# Iron-catalyzed carboazidation of alkenes and alkynes

**DOI:** 10.1038/s41467-018-07985-2

**Published:** 2019-01-10

**Authors:** Haigen Xiong, Nagarajan Ramkumar, Mong-Feng Chiou, Wujun Jian, Yajun Li, Ji-Hu Su, Xinhao Zhang, Hongli Bao

**Affiliations:** 1 Key Laboratory of Coal to Ethylene Glycol and Its Related Technology, State Key Laboratory of Structural Chemistry, Center for Excellence in Molecular Synthesis, Fujian Institute of Research on the Structure of Matter, Chinese Academy of Sciences, 155 Yangqiao Road West, 350002 Fujian, China; 2 University of Chinese Academy of Sciences, 100049 Beijing, China; 3 Hefei National Laboratory for Physical Sciences at the Microscale and Department of Modern Physics, CAS Key Laboratory of Microscale Magnetic Resonance, University of Science and Technology of China, 96 Jinzhai Road, 230026 Hefei, China; 4 Lab of Computational Chemistry and Drug Design, Key Laboratory of Chemical Genomics, Peking University Shenzhen Graduate School, 518055 Shenzhen, China

## Abstract

Carboazidation of alkenes and alkynes holds the promise to construct valuable molecules directly from chemical feedstock therefore is significantly important. Although a few examples have been developed, there are still some unsolved problems and lack of universal methods for carboazidation of both alkenes and alkynes. Here we describe an iron-catalyzed rapid carboazidation of alkenes and alkynes, enabled by the oxidative radical relay precursor *t*-butyl perbenzoate. This strategy enjoys success with a broad scope of alkenes under mild conditions, and it can also work with aryl alkynes which are challenging substrates for carboazidation. A large number of diverse structures, including many kinds of amino acid precursors, fluoroalkylated vinyl azides, other specific organoazides, and 2*H*-azirines can be easily produced.

## Abstract (web summary)

Carboazidation of alkenes and alkynes holds the promise to construct valuable, functionalized molecules. Here, the authors report a general and efficient iron-catalyzed carboazidation of both alkenes and alkynes enabled by *t*-butyl perbenzoate.

## Introduction

**Fig. 1:**
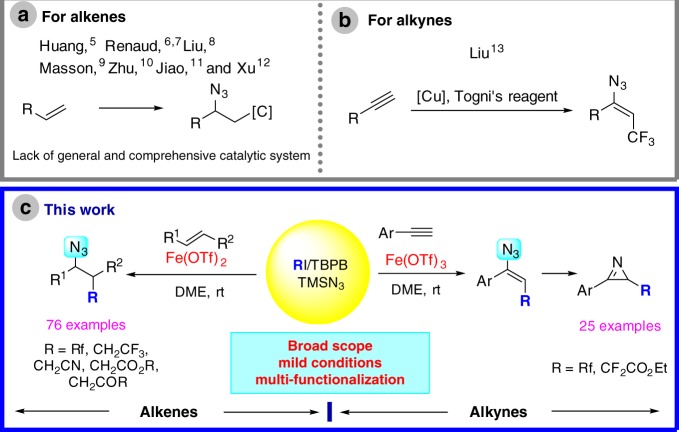
Carboazidation of alkenes and alkynes. **a** Previous arts on carboazidation of alkenes. **b** Previous arts on carboazidation of alkynes. **c** This work: carboazidation of alkenes and alkynes

Amino acids, the basic building blocks of proteins are being used increasingly in bio-relevant modification of proteins and pharmaceutical applications. Development of more versatile methods to provide useful but synthetically challenging amino acid frameworks from chemical feedstocks is always highly desired^1–4^. Carboazidation of alkenes and alkynes holds the promise to construct valuable molecules including amino acid precursors and has therefore attracted much attention recently. Although several carboazidations of alkenes have been developed by Huang^5^, Renaud^6,7^, Liu^8^, Masson^9^, Zhu^10^, Jiao^11^ and Xu^12^, there are some unsolved problems in this field. How to realize the carboazidation reaction using nontoxic, inexpensive and readily available reagents with a broad scope of olefins remains a question. In addition, the carboazidation of alkynes is even more challenging than carboazidation of alkenes (Fig. 1a). There is only one successful carboazidation of alkynes reported by Liu^13^ which works for single carbon functionality, i.e., a trifluoromethyl group using Togni’s reagent (Fig. 1b). The reason for the lack of methods for carboazidation of alkynes might be attributed to the relative lower efficiency of incorporation of azido species compared to other competing reactions. The development of carboazidation of alkenes and alkynes is significantly important from the synthetic point of view.

*t*-Butyl perbenzoate (TBPB) is a commercially available and inexpensive oxidant frequently used as a precursor of the *t*-butoxyl radical^14–22^. Lately, TBPB has been proved to be a good source of methyl radical by Yu^23^ and our group^4,24,25^. Although our understanding of the selective formation of methyl radicals is limited, we found previously that in the presence of Fe(OTf)_2_ or Fe(OTf)_3_, the methyl radical is formed exclusively. We envisioned that TBPB could serve as a polyfunctional reagent for the carboazidation of alkenes and alkynes. Herein, we report our development of a versatile iron-catalyzed rapid carboazidation of both alkenes and alkynes, enabled by TBPB (Fig. 1c).

## Results

### Carboazidation of alkenes

**Fig. 2:**
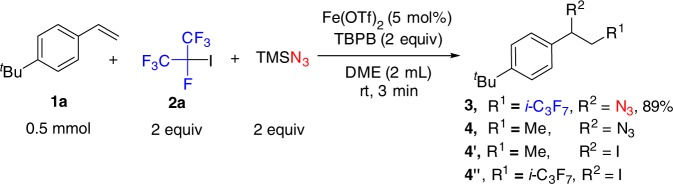
Optimized conditions for carboazidation of alkenes. Fe(OTf)_2_ (5 mol%), **1a** (0.5 mmol), **2a** (1.0 mmol), TMSN_3_ (1.0 mmol), TBPB (1.0 mmol) in DME (2 mL) at rt for 3 min under an N_2_ atmosphere

We investigated the reaction parameters for carboazidation in the presence of TBPB and found that ferrous trifluoromethanesulfonate (Fe(OTf)_2_, ferrous triflate) is optimal (Fig. 2, see details in Supplementary Table 1 and Supplementary Figures 2–4), delivering the corresponding product **3** in 89% yield at rt with DME (dimethoxyethane) as the solvent and azidotrimethylsilane (TMSN_3_) as the azidation reagent. Possible by-products **4**, **4**′, and **4**″ were not observed.

With the optimized conditions in hand, we studied the scope of the reaction with alkyl iodides (Fig. 3 and Supplementary Figures 5–34). Fluoroalkyl iodides were examined first and the corresponding fluoroalkyl-azidation products (**5**–**10**) were obtained in high yields^26^. The reaction of styrene with iodoacetonitrile proceeds smoothly, affording the corresponding product (**11**) in 86% yield. Reactions with ethyl iodoacetates affords products (**12–14)** with the yield ranging from 71–85%. With 1-iodo-3,3-dimethylbutan-2-one the reaction delivers the azide (**15**) in 61% yield. Three electron rich alkyl iodides, i.e., 1-chloro-4-iodobutane, 1-iododecane and 2-iodobutane are not effective in this reaction as the direct azidation of alkyl iodides to form alkyl azides occurs. It should be noted that the reactions with perfluoroalkyl iodides are very fast, completing in 10 min in many cases.

**Fig. 3:**
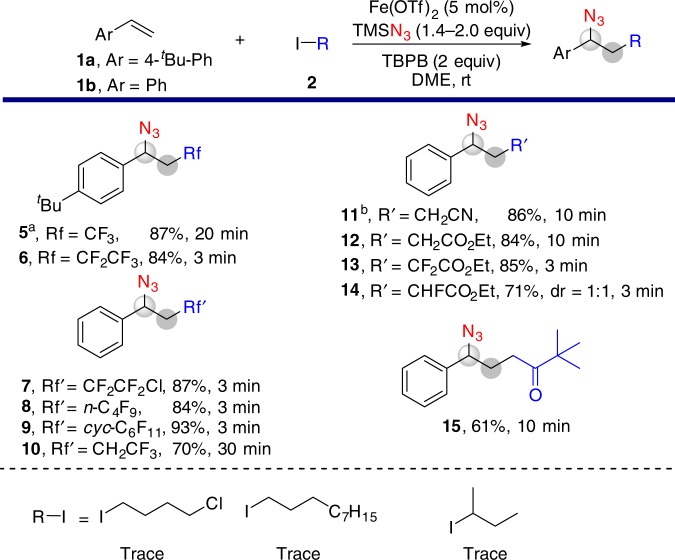
Scope of alkyl iodides. General reaction conditions: Fe(OTf)_2_ (3–5 mol%), **1a** or **1b** (0.5 mmol), **2** (0.65–1.0 mmol), TMSN_3_ (0.7–1.0 mmol), TBPB (0.75–1.0 mmol) in DME (2 mL) at rt under an N_2_ atmosphere. ^a^ Instead of TBPB, lauroyl peroxides (LPO, 0.75 mmol) was applied. ^b^ 50 °C

Subsequently, we studied the substrate scope of olefins (Fig. 4 and Supplementary Figs. 35–189). As examples, *α*-azido esters (**16–27** in Fig. 4a), *β-*azido esters (**28–37** in Fig. 4b), *γ-*azido esters (**38–63** in Fig. 4c), other azido acid derivatives (**64–69** in Fig. 4d–g) and organoazides (**70–75** in Fig. 4h) were obtained. The functional group compatibility of this reaction is good: a series of functional groups, such as halogen, ester, carboxylic acid (**69**), and free hydroxyl group (**74**) are tolerated under the reaction conditions. Both terminal and internal alkenes (**28–37, 58**, and **65**) are compatible with the reaction. The carboazidation reactions of 1-octene with iodomethane and iodobutane are not successful under the reaction conditions.

**Fig. 4:**
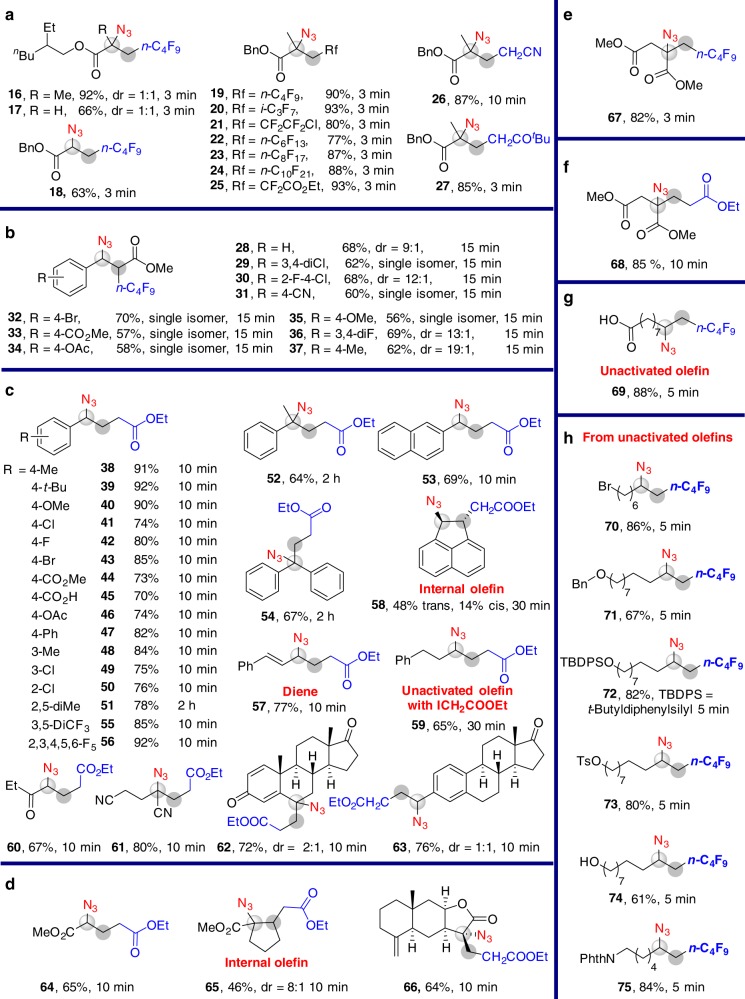
Scope of alkenes. **a** Synthesis of α-azido esters. **b** Synthesis of β-azido esters. **c** Synthesis of γ-azido esters. **d** Synthesis of α,γ-azido esters. **e** Synthesis of an α,β-azido ester. **f** Synthesis of an α,β,γ-azido ester. **g** Synthesis of an azido acid. **h** Synthesis of other organoazides. General reaction conditions: Fe(OTf)_2_ (5 mol%), **1** (0.5 mmol), **2** (0.65–1.0 mmol), TMSN_3_ (1.0 mmol), TBPB (1.0 mmol) in DME (2 mL) at rt under an N_2_ atmosphere. Isolated yields. dr values were determined by ^1^H NMR

To highlight the synthetic applications, **8**, **19** and **78** were reduced to amine **76**^11^, amino acid **77**^26^ and pyrrolidinone **79**, respectively (Fig. 5 and Supplementary Figs. 190–199).

**Fig. 5:**
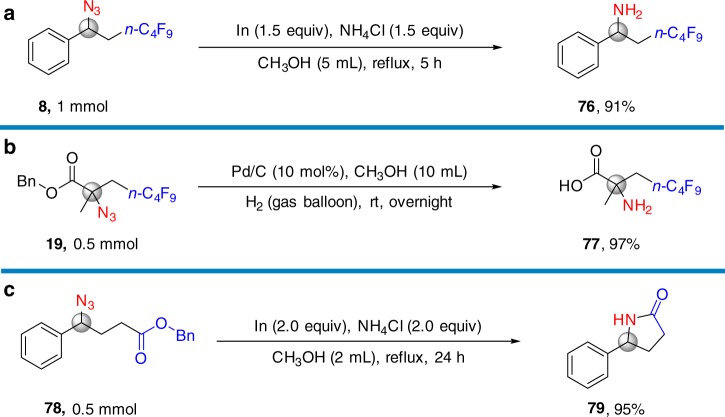
Applications of carboazidation products. **a** Reduction to amine. **b** Reduction to α-amino acid. **c** Cyclization to pyrrolidinones

### Carboazidation of alkynes

**Fig. 6:**
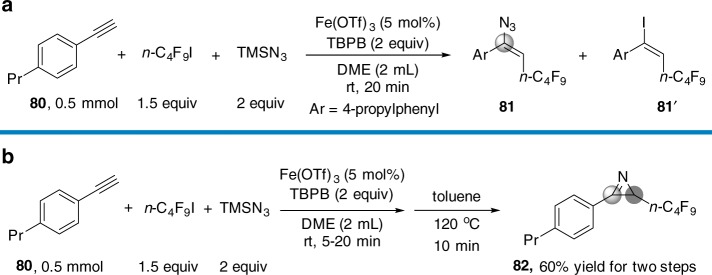
Carboazidation of alkynes. **a** Optimized reaction conditions. **b** Cascade transformation of vinyl azide to 2*H*-azirine

Vinyl azides (1-azidoalkenes)^27,28^ are versatile building blocks in organic synthesis and have been used in many transformations to synthesize bioactive alkaloids and heterocycles^29–36^. Although the carboazidation of alkynes can difunctionalize alkynes, affording 1-azidoalkenes which can be subsequently converted to 2*H*-azirines, reports of such efficient methods are rare^13^, and accordingly, we studied the carboazidation of alkynes. After carefully screening the reaction conditions, Fe(OTf)_3_ was found to be the best catalyst, producing a carboazidation product (**81**) while avoiding the formation of the atom-transfer radical addition (ATRA) product (**81**′) (Fig. 6a, see details in Supplementary Table 2 and Supplementary Figs. 200–208). In view of the broad synthetic utilities of 2*H*-azirines, the conversion of vinyl azides to 2*H*-azirines was studied. It was found that compound **81** could be converted into a 2*H*-azirine (**82**) in toluene at 120 °C (Fig. 6b).

With these conditions identified, we studied the substrate scope regarding alkyl iodides and alkynes. The results are shown in Fig. 7 and Supplementary Figs. 209–283. Fluoroalkyl iodides and aryl alkynes react well in these transformations. Reaction of 1-iododecane with ethynylbenzene does not deliver the desired product. As an example, reaction of 1-octyne delivers only the ATRA product **(107**)^37^ in 42% yield.

**Fig. 7:**
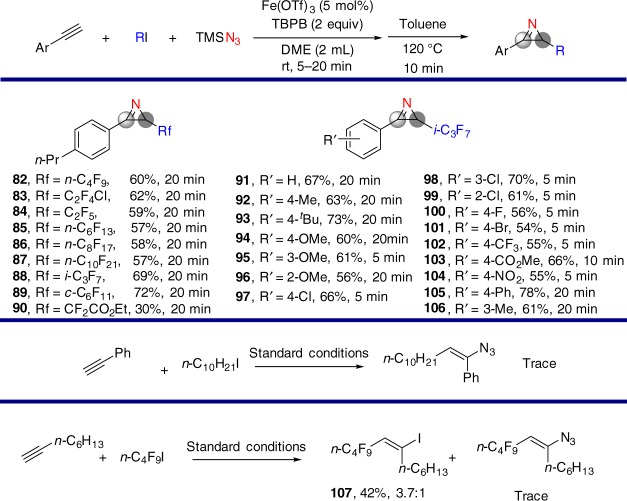
Substrate scope of carboazidation of alkynes and further transformation to 2*H*-azirines. General reaction conditions: Fe(OTf)_3_ (0.025 mmol), alkyne (0.5 mmol), RI (0.75 mmol), TMSN_3_ (1.0 mmol), TBPB (1.0 mmol) in DME (2 mL) at rt for 5–20 min and then in toluene at 120 °C for 10 min under an N_2_ atmosphere. Isolated yields

To highlight the synthetic applications of this method further, vinyl azides and 2*H*-azirine were converted to **108**^38^ **109**^39^ and **110**^40^ in high yields (Fig. 8 and Supplementary Figs. 284–295). The geometry of vinyl azides was confirmed by X-ray crystallographic analysis of product **109** (see details in Supplementary Figure 1 and Supplementary Table 3).

**Fig. 8:**
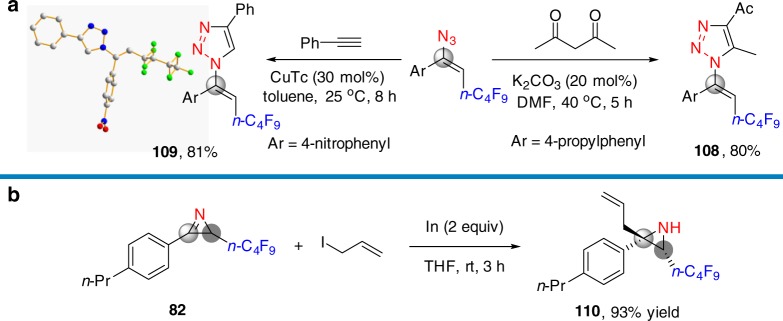
Further synthetic transformations. **a** Synthesis of 1,2,3-triazoles. **b** Synthesis of aziridine

## Discussion

In summary, we have developed a carboazidation of alkenes and alkynes enabled by TBPB. This key transformation has been successfully used to afford various valuable structural skeletons, including many amino acid precursors, vinyl azides and 2*H*-azirines. It is noteworthy that this carboazidation works for both alkenes and alkynes with multiple carbon functionalities.

## Methods

### Typical procedure for carboazidation of alkenes

Fe(OTf)_2_ (9 mg, 0.025 mmol) was added to a dried Schlenk tube equipped with a magnetic bar. This tube was then flushed with N_2_ gas (3 times) and an N_2_ atmosphere was maintained using an N_2_ balloon. A thoroughly mixed solution of alkene (0.5 mmol), alkyl iodide (0.65–1.5 mmol), TMSN_3_ (0.7–1.7 mmol) and TBPB (0.75–1.75 mmol) in DME (2 mL) was added to the catalyst by syringe and the mixture was stirred vigorously for 3–120 min at the appropriate temperature. After completion of the reaction, judged by TLC, the solvent was evaporated and the residue was purified by flash chromatography on silica gel using petroleum ether and EtOAc to give the corresponding product.

### Typical procedure for carboazidation of alkynes

Fe(OTf)_3_ (12.7 mg, 0.025 mmol) was added to a dried Schlenk tube equipped with a magnetic bar. Then this tube was flushed with N_2_ (3 times) and an N_2_ atmosphere was maintained using an N_2_ balloon. A thoroughly mixed solution of alkyne (0.5 mmol), R_f_I (0.75 mmol), TMSN_3_ (1.0 mmol) and TBPB (1.0 mmol) in DME (2 mL) was added to the catalyst by syringe and the mixture was stirred vigorously for 5–20 min at rt. After completion of the reaction, judged by TLC, the volatile compounds were removed by pump and the residue was dissolved in toluene (3 mL). The resulting mixture was then stirred at 120 °C for 10 min. The solvent was then evaporated and the residue was purified by flash chromatography on silica gel using petroleum ether and EtOAc to give the corresponding product.

## Supplementary information

Supplementary Information

## Data Availability

Detailed experimental procedures and characterization of compounds can be found in the Supplementary Information. The X-ray crystallographic coordinates for structures reported in this article have been deposited at the Cambridge Crystallographic Data Centre (109: CCDC 1864994). These data could be obtained free of charge from The Cambridge Crystallographic Data Centre via www.ccdc.cam.ac.uk/data_request/cif. All data are available from the authors upon request.
