# Supplementary material for: Iron-catalyzed carboazidation of alkenes and alkynes
Source: Nat Commun. 2019 Jan 10;10:122. doi: 10.1038/s41467-018-07985-2 (PMC6328574; doi:10.1038/s41467-018-07985-2)
Supplement: Supplementary file 1 — Supplementary Information [file 41467_2018_7985_MOESM1_ESM.pdf]

Supplementary Information

Iron-Catalyzed Carboazidation of Alkenes and Alkynes

Xiong *et al.*

Table of contents

Supplementary information 3

Optimization of the reaction conditions 4

  Optimization for carboazidation of alkenes 4

    Supplementary Table 1 Optimization for carboazidation of alkenes 4

  Optimization for carboazidation of alkynes 6

    Supplementary Table 2 Optimization for carboazidation of alkynes 6

General procedure for carboazidation of alkenes and alkynes 8

  General procedure A: Carboazidation of alkenes 8

  General procedure B: Carboazidation of alkynes for the synthesis of 2H-azirines 8

Characterization data of carboazidation of alkenes 9

Reduction of azido group to synthesize amine, amino acid and pyrrolidinone 40

  (a) Synthesis of amine 40

  (b) Synthesis of amino acid 40

  (c) Synthesis of pyrrolidinone 41

Characterization data of carboazidation of alkynes 42

Applications of the products from carbonazidation of alkynes 54

  (a) Synthesis of 1,2,3-triazole 108 54

  (b) Synthesis of 1,2,3-triazole 109 55

  (c) Transformation of 2H-azirine 57

Single crystal data of compound 109 58

  Supplementary Table 3 Crystal data and structure refinement for compound 109. 58

Copies of ^{1}H, ^{19}F and ^{13}C NMR spectra 59

Supplementary References 207

Supplementary information

All reactions were carried out under an atmosphere of nitrogen in dried glassware with magnetic stirring unless otherwise indicated. Unless otherwise noted, materials obtained from commercial suppliers were used without further purification. Solvents were dried by Innovative Technology Solvent Purification System. Liquids and solutions were transferred via syringe. All reactions were monitored by thin-layer chromatography. GC and GC-MS data were recorded on Thermo Trace 1300 and Thermo ISQ QD, respectively. ^{1}H, ^{19}F and ^{13}C NMR spectra were recorded on Bruker-BioSpin AVANCE III HD-400 Hz. Data for ^{1}H NMR spectra are reported relative to chloroform as an internal standard (7.26 ppm) and are reported as follows: chemical shift (ppm), multiplicity, coupling constant (Hz), and integration. Data for ^{13}C NMR spectra were reported relative to chloroform as an internal standard (77.00 ppm) and are reported in terms of chemical shift (ppm). IR data were obtained from Bruker VERTEX 70. All melting points were determined on a Beijing Science Instrument Dianguang Instrument Factory XT4B melting point apparatus and are uncorrected. HRMS(ESI) data were recorded on Bruker Impact II UHR-TOF; HRMS(EI) data were recorded on Waters Micromass GCT Premier.

Optimization of the reaction conditions

Optimization for carboazidation of alkenes

To a dried Schlenk tube equipped with a magnetic bar, catalyst (x mmol) was added, then this tube was flushed with nitrogen gas (3 times) and maintained a nitrogen atmosphere using a nitrogen balloon. A thoroughly mixed solution of vinylarene **1a** (0.5 mmol), alkyl iodide **2a** (1.0 mmol), $TMSN_3$ (1.0 mmol) and TBPB (1.0 mmol) in solvent (2 mL) was added to the catalyst via syringe and stirred vigorously for 3 h at 50 °C. The yield of the product **3** was determined by ^1H NMR analysis using CH_2Br_2 as an internal standard.

Supplementary Table 1 Optimization for carboazidation of alkenes

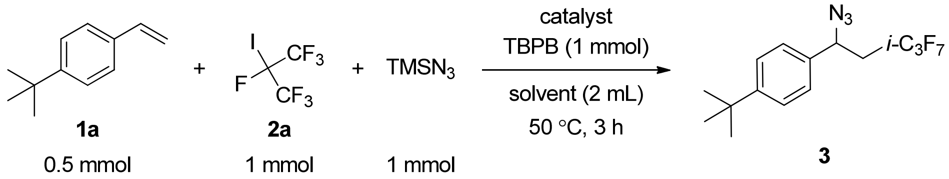

Entry	Catalyst [a]	Solvent	Yield **3** (%) [b]
1	CuI	THF	trace
2	CuTc	THF	trace
3	$Cu(CH_3CN)_4PF_6$	THF	trace
4	$Cu(acac)_2$	THF	trace
5	$CuCl_2$	THF	trace
6	$Fe(OAc)_2$	THF	11
7	$Fe(acac)_3$	THF	14
8	$Fe(OTs)_3$	THF	39
9	$FeCl_3$	THF	trace
10	$FeBr_3$	THF	trace
11	$FeBr_2$	DME	trace
12	$Fe(OTf)_2$	THF	72
13	$Fe(OTf)_3$	THF	72

14	$Ni(acac)_2$	THF	trace
15	$NiBr_2$	THF	trace
16	Ag_2CO_3	THF	trace
17	$Pd(OAc)_2$	THF	trace
18	$PdCl_2$	THF	trace
19	$MnCl_2\cdot 4H_2O$	THF	17
20	$CoCl_2$	THF	trace
21	$In(OTf)_3$	THF	32
22	/	THF	trace
23	TfOH (50 mol %)	THF	trace
24	$Fe(OTf)_2$	THF	73[c]
25	$Fe(OTf)_2$	Dioxane	74[c]
26	$Fe(OTf)_2$	DME	81[c]
27	$Fe(OTf)_2$	CH_3CN	70[c]
28	$Fe(OTf)_2$	Toluene	18[c]
29	$Fe(OTf)_2$	DCM	11[c]
30	$Fe(OTf)_2$	DMF	trace[c]
31	$Fe(OTf)_2$	MTBE	trace[c]
**32**	**$Fe(OTf)_2$ (5 mol%)**	**DME**	**91 (89)**[c,d]
33	$Fe(OTf)_2$ (5 mol%)	DME	12[c] (BPO was used instead of TBPB)
34	$Fe(OTf)_2$ (5 mol%)	DME	17[c] (H_2O_2 was used instead of TBPB)
35	$Fe(OTf)_2$ (5 mol%)	DME	79[c] ($CH_3CO_3C(CH_3)_3$ was used instead of TBPB)
36	$Fe(OTf)_2$ (5 mol%)	DME	90[c] (LPO was used instead of TBPB)

[a]: Catalyst (10 mol%) was used.
[b]: Yield was determined by GC analysis.
[c]: At room temperature.
[d]: Isolated yield was given in parentheses.

Optimization for carboazidation of alkynes

To a dried Schlenk tube equipped with a magnetic bar, catalyst (0.025 mmol) was added, then this tube was flushed with nitrogen gas (3 times) and maintained a nitrogen atmosphere using a nitrogen balloon. A thoroughly mixed solution of **80** (0.5 mmol), R_fI (0.7 mmol), $TMSN_3$ (1.0 mmol) and TBPB (1.0 mmol) in solvent (2 mL) was added to the catalyst via syringe and stirred vigorously for 4 h at room temperature. The yield of the product **81** and **81'** was determined by ^1H NMR analysis using CH_2Br_2 as an internal standard.

Supplementary Table 2 Optimization for carboazidation of alkynes

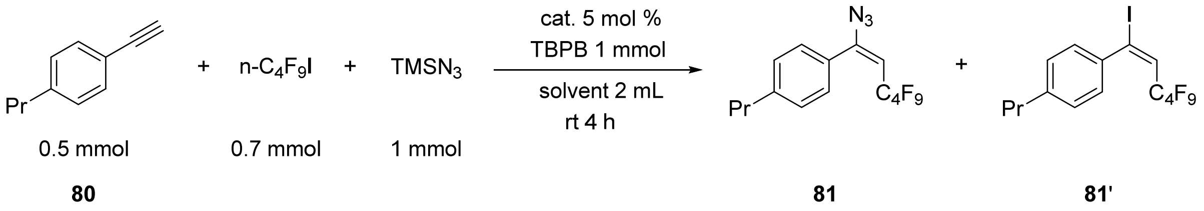

entry	Catalyst/mol%	solvent	Yield **81** (%)[a]	Yield **81'** (%)[a]
1	$NiCl_2$	CH_3CN	trace	trace
2	$CoCl_2$	CH_3CN	trace	21
3	CuBr	CH_3CN	trace	trace
4	$Cu(OAc)_2$	CH_3CN	trace	trace
5	CuTc	CH_3CN	trace	trace
6	$Cu(CH_3CN)_4PF_6$	CH_3CN	6	11
7	$Fe(Cp)_2$	CH_3CN	trace	trace
8	$Fe(acac)_2$	CH_3CN	4	28
9	$Fe(OAc)_2$	CH_3CN	26	31
10	$Fe(OTs)_3$	CH_3CN	29	32
11	$Fe(OTf)_2$	CH_3CN	47	13
12	$Fe(OTf)_3$	CH_3CN	54	10
13	$Fe(OTf)_3$	CH_2Cl_2	13	48
14	$Fe(OTf)_3$	1,4-dioxane	56	27
15	$Fe(OTf)_3$	DME	67	trace

16	$Fe(OTf)_3$	THF	50	11
17	$Fe(OTf)_3$	Toluene	11	35
18	$Fe(OTf)_3$	MTBE	18	48
19[b]	$Fe(OTf)_3$	DME	75(74)[c]	trace
20[d]	$Fe(OTf)_3$	DME	73	trace

[a]: Yield was determined by ^{1}H NMR analysis.

[b]: 1.5 equivalents of R_fI was used and the reaction ran for 20 minutes.

[c]: Isolated yield was given in parentheses.

[d]: LPO was used instead of TBPB, 1.5 equivalents of R_fI was used and the reaction ran for 20 minutes.

General procedure for carboazidation of alkenes and alkynes

General procedure A: Carboazidation of alkenes

To a dried Schlenk tube equipped with a magnetic bar, $Fe(OTf)_2$ (9 mg, 0.025 mmol) was added, Then this tube was flushed with nitrogen gas (3 times) and maintained a nitrogen atmosphere using a nitrogen balloon. A thoroughly mixed solution of alkene (0.5 mmol), alkyl iodide (0.65-1.5 mmol), $TMSN_3$ (0.7-1.7 mmol) and TBPB (0.75-1.75 mmol) in DME (2 mL) was added to the catalyst via syringe and the mixture was stirred vigorously for 3 - 120 minutes at appropriate temperature. After completion (TLC), the solvent was evaporated and the residue was purified by flash chromatography on silica gel using petroleum ether and ethyl acetate to give the corresponding product.

General procedure B: Carboazidation of alkynes for the synthesis of 2H-azirines

To a dried Schlenk tube equipped with a magnetic bar, $Fe(OTf)_3$ (12.7 mg, 0.025 mmol) was added, Then this tube was flushed with nitrogen gas (3 times) and maintained a nitrogen atmosphere using a nitrogen balloon. A thoroughly mixed solution of alkyne (0.5 mmol), R_fI (0.75 mmol), $TMSN_3$ (1.0 mmol) and TBPB (1.0 mmol) in DME (2 mL) was added to the catalyst via syringe and the mixture was stirred vigorously for 5 – 20 minutes at room temperature. After completion (TLC), the volatile compounds were removed by pump and the residue was dissolved in 3 mL of toluene. The resulting mixture was then stirred at 120 ^{o}C for 10 minutes. The solvent was evaporated and the residue was purified by flash chromatography on silica gel using petroleum ether and ethyl acetate to give the corresponding product.

Characterization data of carboazidation of alkenes

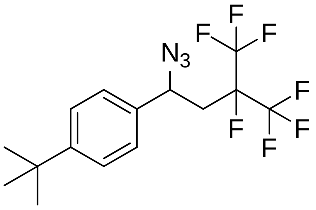

3

Following the **general procedure A**, the mixture of vinylarene (0.5 mmol), alkyl iodide (1.0 mmol), $TMSN_3$ (1.0 mmol) and TBPB (1.0 mmol) in DME (2 mL) was added to $Fe(OTf)_2$ (0.025 mmol) at room temperature for 3 minutes to afford **3**. Yield: 165 mg, 89%; clear oil; IR (KBr): υ 2967, 2114, 1465, 1225, 1162, 1110, 727 cm^{-1}; ^1H NMR (400 MHz, $CDCl_3$) δ 7.45 (d, J = 8.4 Hz, 2H), 7.27 (d, J = 8.3 Hz, 2H), 4.85 (dd, J = 8.9, 3.5 Hz, 1H), 2.69 – 2.37 (m, 2H), 1.35 (s, 9H); ^{19}F NMR (376 MHz, $CDCl_3$) δ -72.98 – -80.44 (m, 6F), -183.68 – -188.16 (m, 1F); ^{13}C NMR (100 MHz, $CDCl_3$) δ 152.33, 135.88, 126.33, 126.28, 120.96 (dd, J = 285.7, 27.6 Hz), 90.93 (dm, J = 207.0 Hz), 59.84, 35.80 (d, J = 18.9 Hz), 34.83, 31.37; HRMS (EI) calcd for $[C_{15}H_{16}F_7N_3]^+([M]^+)$: 371.1232, found: 371.1238.

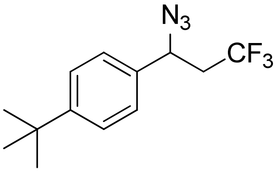

5

Following **the general procedure A**, the mixture of vinylarene (0.5 mmol), $TMSN_3$ (0.75 mmol) in DME (2 mL) was added to $Fe(OTf)_2$ (0.015 mmol) and LPO (0.75 mmol) at room temperature for 20 minutes (with a CF_3I balloon) to afford **5**. Yield: 118 mg, 87%; clear oil; IR (KBr): υ 2966, 2872, 2112, 1513, 1384, 1256, 1142, 831, 744 cm^{-1}; ^1H NMR (400 MHz, $CDCl_3$) δ 7.43 (d, J = 8.5 Hz, 2H), 7.24 (d, J = 8.3 Hz, 2H), 4.75 (dd, J = 8.9, 4.7 Hz, 1H), 2.70 – 2.40 (m, 2H), 1.32 (s, 9H); ^{19}F NMR (376 MHz, $CDCl_3$) δ -64.15(s, 3F); ^{13}C NMR (100 MHz, $CDCl_3$) δ 152.12, 134.70, 126.32, 126.06, 59.62 (q, J = 3.4 Hz), 40.34 (q, J = 28.3 Hz), 34.66, 31.22. The NMR data is consistent with the reported value.[1]

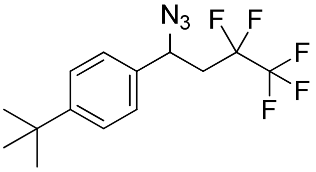

6

Following the **general procedure A**, the mixture of vinylarene (0.5 mmol), alkyl iodide (1.0 mmol), $TMSN_3$ (0.7 mmol) and TBPB (0.75 mmol) in DME (2 mL) was added to $Fe(OTf)_2$ (0.025 mmol) at room temperature for 3 minutes to afford **6**. Yield: 135 mg, 84%; clear oil; IR (KBr): υ 2966, 2114, 1345, 1198, 1110, 835 cm^{-1}; ^1H NMR (400 MHz, $CDCl_3$) δ 7.41 (d, *J* = 7.6 Hz, 2H), 7.24 (d, *J* = 7.5 Hz, 2H), 4.83 (dd, *J* = 8.5, 4.0 Hz, 1H), 2.64 – 2.29 (m, 2H), 1.30 (s, 9H); ^{19}F NMR (376 MHz, $CDCl_3$) δ -85.57 – -85.98 (m, 3F), -117.09 – -117.70 (m, 2F); ^{13}C NMR (100 MHz, $CDCl_3$) δ 152.18, 135.13, 126.30, 126.13,130.45 – 110.71 (m, CF_2CF_3), 58.67, 37.27 (t, *J* = 20.8 Hz, CH_2-CF_2), 34.68, 31.23; HRMS (EI) calcd for $[C_{14}H_{16}F_5N_3]^+([M]^+)$: 321.1264, found: 321.1261.

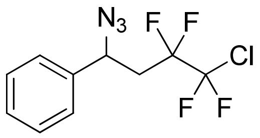

7

Following the **general procedure A**, the mixture of vinylarene (0.5 mmol), alkyl iodide (0.65 mmol), $TMSN_3$ (0.7 mmol) and TBPB (0.75 mmol) in DME (2 mL) was added to $Fe(OTf)_2$ (0.025 mmol) at room temperature for 3 minutes to afford **7**. Yield: 123 mg, 87%; clear oil; IR (KBr): υ 3035, 2926, 2111, 1456, 1379, 1247, 1151, 1088, 700 cm^{-1}; ^1H NMR (400 MHz, $CDCl_3$) δ 7.46 – 7.30 (m, 5H), 4.88 (dd, *J* = 7.8, 5.0 Hz, 1H), 2.71 – 2.39 (m, 2H); ^{19}F NMR (376 MHz, $CDCl_3$) δ-70.97 – -72.27 (m, 2F), -112.59 – -113.60 (m, 2F); ^{13}C NMR (100 MHz, $CDCl_3$) δ 169.09, 138.21, 129.23, 129.06, 123.34 (tt, *J* = 298.6, 37.1 Hz), 115.98 (tt, *J* = 256.7, 33.7 Hz), 59.30, 37.24 (t, *J* = 21.2 Hz, CH_2-CF_2); HRMS (EI) calcd for $[C_{10}H_8ClF_4N_3]^+([M]^+)$: 281.0343, found: 281.0344.

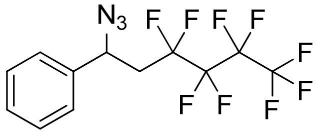

8

Following the **general procedure A**, the mixture of vinylarene (0.5 mmol), alkyl iodide (0.65 mmol), $TMSN_3$ (0.7 mmol) and TBPB (0.75 mmol) in DME (2 mL) was added to $Fe(OTf)_2$ (0.025 mmol) at room temperature for 3 minutes to afford **8**. Yield: 154 mg, 84%; clear oil; IR (KBr): υ 3036, 2927, 2110, 1456, 1236, 1134, 1067, 700 cm^{-1}; ^1H NMR (400 MHz, $CDCl_3$) δ 7.47 – 7.30 (m, 5H), 4.90 (dd, *J* = 8.1, 4.8 Hz, 1H), 2.71 – 2.37 (m, 2H); ^{19}F NMR (376 MHz, $CDCl_3$) δ -78.08 – -84.18 (m, 3F), -111.65 – -115.38 (m, 2F), -123.68 – -125.20 (m, 2F), -125.38 – -126.48 (m, 2F); ^{13}C NMR (100 MHz, $CDCl_3$) δ 138.12, 129.25, 129.09,

126.64, 122.16 – 104.11 (m, (CF$_2$)$_3$$CF_3$), 58.95, 37.40 (t, J = 20.9 Hz, CH$_2$-CF$_2$); HRMS (EI) calcd for $[C_{12}H_8F_9N_3]^+$($[M]^+$): 365.0575, found: 365.0580.

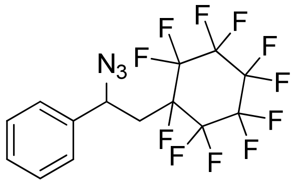

9

Following the **general procedure A**, the mixture of vinylarene (0.5 mmol), alkyl iodide (0.65 mmol), $TMSN_3$ (0.7 mmol) and TBPB (0.75 mmol) in DME (2 mL) was added to $Fe(OTf)_2$ (0.025 mmol) at room temperature for 3 minutes to afford **9**. Yield: 198 mg, 93%; clear oil; IR (KBr): υ 3036, 2930, 2109, 1456, 1316, 1233, 1180, 1032, 700 cm^{-1}; ^{1}H NMR (400 MHz, $CDCl_3$) δ 7.48 – 7.30 (m, 5H), 4.92 (dd, J = 8.0, 3.9 Hz, 1H), 2.76 – 2.49 (m, 2H); ^{19}F NMR (376 MHz, $CDCl_3$) δ -109.73 – -128.04 (m, 5F), -128.58 – -150.45 (m, 5F), -183.16 – -187.72 (m, 1F); ^{13}C NMR (100 MHz, $CDCl_3$) δ 138.63, 129.29, 129.10, 126.46, 112.99 (m), 110.35 (m), 107.89 (m), 105.36 (m), 91.74 (m), 89.70 (m), 59.79, 32.70 (d, J =19.2 Hz, CH$_2$-CF); HRMS (EI) calcd for $[C_{14}H_8F_{11}N_3]^+$($[M]^+$): 427.0543, found: 427.0536.

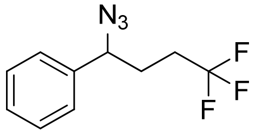

10

Following the **general procedure A**, the mixture of alkene (0.5 mmol), alkyl iodide (1.5 mmol), $TMSN_3$ (1.5 mmol) and TBPB (1.5 mmol) in DME (2 mL) was added to $Fe(OTf)_2$ (0.025 mmol) at room temperature for 30 minutes to afford **10**. Yield: 81 mg, 70%; clear oil; IR (KBr): υ 2953, 2101, 1454, 1252, 1143, 1001, 701 cm^{-1}; ^{1}H NMR (400 MHz, $CDCl_3$) δ 7.48 – 7.26 (m, 5H), 4.51 (t, J = 6.8 Hz, 1H), 2.32 – 1.86 (m, 4H); ^{19}F NMR (376 MHz, $CDCl_3$) δ -66.12 (s, 3F); ^{13}C NMR (100 MHz, $CDCl_3$) δ 138.36, 129.11, 128.80, 126.86 (q, J = 276.3 Hz, CF$_3$), 126.79, 64.83, 30.67 (q, J = 29.3 Hz, CH$_2$-CF$_3$), 28.87 (q, 2.6 Hz, CH$_2$-CH$_2$-CF$_3$); HRMS (EI) calcd for $[C_{10}H_{10}F_3N_3]^+$($[M]^+$): 229.0827, found: 229.0837.

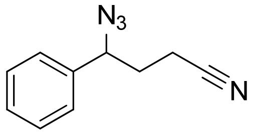

11

Following the **general procedure A**, the mixture of vinylarene (0.5 mmol), alkyl iodide (1.0 mmol), $TMSN_3$ (1.0 mmol) and TBPB (1.0 mmol) in DME (2 mL) was added to $Fe(OTf)_2$ (0.025 mmol) at 50 °C for 10 minutes to afford **11**. Yield: 80 mg, 86%; clear oil; IR (KBr): υ

3033, 2936, 2247, 2097, 1454, 1248, 1027, 701 cm^{-1}; ^{1}H NMR (400 MHz, $CDCl_3$) δ 7.46 – 7.35 (m, 3H), 7.35 – 7.28 (m, 2H), 4.63 (dd, *J* = 8.4, 6.0 Hz, 1H), 2.54 – 2.31 (m, 2H), 2.16 – 1.96 (m, 2H); ^{13}C NMR (100 MHz, $CDCl_3$) δ 137.76, 129.23, 129.03, 126.84, 118.75, 64.37, 32.02, 14.33; HRMS (ESI) calcd for $[C_{10}H_{10}N_4Na]^+$($[M+Na]^+$): 209.0798, found: 209.0803.

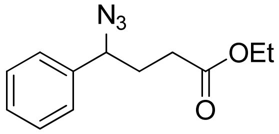

12

Following the **general procedure A**, the mixture of vinylarene (0.5 mmol), alkyl iodide (1.0 mmol), $TMSN_3$ (1.0 mmol) and TBPB (1.0 mmol) in DME (2 mL) was added to $Fe(OTf)_2$ (0.025 mmol) at room temperature for 10 minutes to afford **12**. Yield: 98 mg, 84%; clear oil; IR (KBr): υ 2982, 2098, 1734, 1453, 1250, 701 cm^{-1}; ^{1}H NMR (400 MHz, $CDCl_3$) δ 7.42 – 7.27 (m, 5H), 4.53 (t, *J* = 7.1 Hz, 1H), 4.12 (q, *J* = 7.1 Hz, 2H), 2.37 (t, *J*= 7.3 Hz, 2H), 2.18 – 2.00 (m, 2H), 1.25 (t, *J* = 7.1 Hz, 3H); ^{13}C NMR (100 MHz, $CDCl_3$) δ 172.72, 139.02, 128.90, 128.46, 126.89, 65.35, 60.56, 31.38, 30.81, 14.22; HRMS (ESI) calcd for $[C_{12}H_{15}N_3NaO_2]^+$($[M+Na]^+$):256.1056, found: 256.1059.

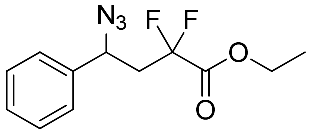

13

Following the **general procedure A**, the mixture of vinylarene (0.5 mmol), alkyl iodide (0.75 mmol), $TMSN_3$ (0.85 mmol) and TBPB (0.85 mmol) in DME (2 mL) was added to $Fe(OTf)_2$ (0.025 mmol) at room temperature for 3 minutes to afford **13**. Yield: 114 mg, 85%; clear oil; IR (KBr): υ 2928, 2108, 1763, 1455, 1231, 1194, 1101, 700 cm^{-1}; ^{1}H NMR (400 MHz, $CDCl_3$) δ 7.46 – 7.27 (m, 5H), 4.75 (dd, *J* = 9.5, 4.4 Hz, 1H), 4.31 – 4.20 (m, 2H), 2.77 – 2.58 (m, 1H), 2.52 – 2.37 (m, 1H), 1.34 (t, *J* = 7.2 Hz, 3H); ^{19}F NMR (376 MHz, $CDCl_3$) δ -102.47 (d, *J* = 264.5 Hz, 1F), -107.01 (d, *J* = 264.5 Hz, 1F); ^{13}C NMR (100 MHz, $CDCl_3$) δ 163.54 (dd, *J* = 32.5, 31.7 Hz), 137.87, 129.12, 129.01, 126.86, 114.54 (dd, *J* = 252.7, 249.8 Hz), 63.13, 60.03 (dd, *J* = 7.2, 3.9 Hz), 41.07 (t, *J* = 23.8 Hz), 13.84; HRMS (ESI) calcd for $[C_{12}H_{13}F_2N_3NaO_2]^+$($[M+Na]^+$): 292.0868, found:292.0865.

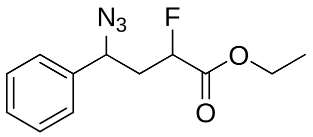

14

Following the **general procedure A**, the mixture of vinylarene (0.5 mmol), alkyl iodide (1.0 mmol), $TMSN_3$ (1.0 mmol) and TBPB (1.0 mmol) in DME (2 mL) was added to $Fe(OTf)_2$ (0.025 mmol) at room temperature for 3 minutes to afford **14**. Yield: 90 mg, 71% (dr = 1:1); clear oil; IR (KBr): υ 2983, 2102, 1762, 1455, 1247, 1096, 701 cm^{-1}; ^1H NMR (400 MHz, $CDCl_3$) δ 7.48 – 7.30 (m, 10H), 5.26 – 5.05 (m, 1H), 4.85 – 4.66 (m, 3H), 4.34 – 4.12 (m, 4H), 2.53 – 2.07 (m, 4H), 1.34 – 1.25 (m, 6H); ^{19}F NMR (376 MHz, $CDCl_3$) δ -193.77 (s, 1F), -194.47 (s, 1F); ^{13}C NMR (100 MHz, $CDCl_3$) δ 169.25 (d, J = 23.1 Hz), 169.13 (d, J = 23.3 Hz), 138.47, 137.90, 129.15, 129.07, 128.90, 128.80, 127.09, 126.87, 86.03 (d, J = 185.5 Hz), 86.01 (d, J = 184.6 Hz), 61.83, 61.78, 61.56 (d, J = 3.7 Hz), 61.45 (d, J = 2.6 Hz), 39.32 (d, J = 20.7 Hz), 38.61 (d, J = 20.5 Hz), 14.09; HRMS (ESI) calcd for $[C_{12}H_{14}FN_3NaO_2]^+$($[M+Na]^+$): 274.0962, found:274.0962.

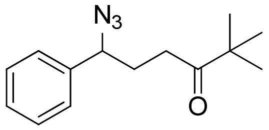

15

Following the **general procedure A**, the mixture of vinylarene (0.5 mmol), alkyl iodide (1.0 mmol), $TMSN_3$ (1.0 mmol) and TBPB (1.0 mmol) in DME (2 mL) was added to $Fe(OTf)_2$ (0.025 mmol) at room temperature for 10 minutes to afford **15**. Yield: 75mg, 61%; clear oil; IR (KBr): υ 2968, 2096, 1705, 1477, 1247, 700 cm^{-1}; ^1H NMR (400 MHz, $CDCl_3$) δ 7.41 – 7.28 (m, 5H), 4.49 (t, J= 7.3 Hz, 1H), 2.65 – 2.49 (m, 2H), 2.03 (m, 2H), 1.12 (s, 9H); ^{13}C NMR (100 MHz, $CDCl_3$) δ 215.00, 139.37, 128.86, 128.37, 126.87, 65.40, 44.16, 32.80, 30.28, 26.43; HRMS (ESI) calcd for $[C_{14}H_{19}N_3NaO]^+$($[M+Na]^+$): 268.1420, found: 268.1422.

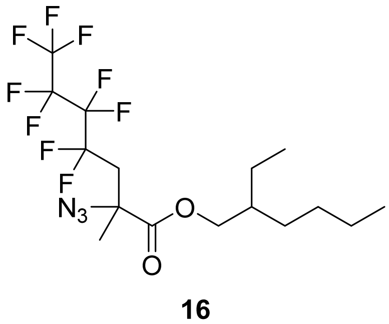

16

Following the **general procedure A**, the mixture of alkene (0.5 mmol), alkyl iodide (0.75 mmol), $TMSN_3$ (0.75mmol) and TBPB (0.8 mmol) in DME (2 mL) was added to $Fe(OTf)_2$ (0.025 mmol) at room temperature for 3 minutes to afford **16**. Yield: 212 mg, 92% (dr = 1:1); clear oil; IR (KBr): υ 2964, 2121, 1746, 1463, 1354, 1235, 1160, 737 cm^{-1}; ^1H NMR (400 MHz, $CDCl_3$) δ 4.21 – 4.10 (m, 2H), 2.78 – 2.46 (m, 2H), 1.70 – 1.58 (m, 4H), 1.43 – 1.26 (m, 8H), 0.95 – 0.86 (m, 6H). ^{19}F NMR (376 MHz, $CDCl_3$) δ -78.24 – -83.94 (m, 3F), -

109.17 – -111.28 (m, 1F), -112.37 – -114.06 (m, 1F), -124.05 – -125.28 (m, 2F), -125.38 – -126.52 (m, 2F); ^{13}C NMR (100 MHz, CDCl3) δ 170.74, 121.46 – 104.60 (m, ($CF_2)_3CF_3$), 68.92, 62.36, 38.73, 37.53 (t, J = 20.3 Hz, CH_2-CF_2), 30.29, 28.83, 23.70, 23.24, 22.85, 13.86, 10.79. HRMS (ESI) calcd for $[C_{16}H_{22}F_9N_3NaO_2]^+$($[M+Na]^+$): 482.1461, found: 482.1461.

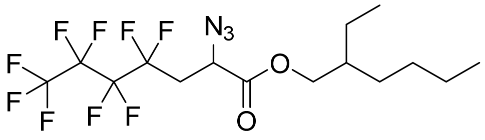

17

Following the **general procedure A**, the mixture of alkene (0.5 mmol), alkyl iodide (0.75 mmol), $TMSN_3$ (0.75 mmol) and TBPB (0.8 mmol) in DME (2 mL) was added to $Fe(OTf)_2$ (0.025 mmol) at room temperature for 3 minutes to afford **17**. Yield: 147 mg, 66% (dr = 1:1); clear oil; IR (KBr): υ 2965, 2934, 2125, 1749, 1465, 1236, 1135, 881 cm^{-1}; ^{1}H NMR (400 MHz, CDCl3) δ 4.30 (dd, J = 8.2, 4.5 Hz, 1H), 4.23 – 4.13 (m, 2H), 2.88 – 2.69 (m, 1H), 2.53 – 2.34 (m, 1H), 1.65 (m, 1H), 1.44 – 1.27 (m, 8H), 0.96 – 0.86 (m, 6H); ^{19}F NMR (376 MHz, CDCl3) δ -80.58 – -81.82 (m, 3F), -112.18 – -114.93 (m, 2F), -124.22 – -124.82 (m, 2F), -125.35 – -126.70 (m, 2F); ^{13}C NMR (100 MHz, CDCl3) δ 168.56, 120.62 – 103.69 (m, ($CF_2)_3CF_3$), 69.13, 55.61, 38.72, 32.44 (t, J = 21.2 Hz, CH_2-CF_2), 30.28, 28.84, 23.67, 22.86, 13.87, 10.83; HRMS (ESI) calcd for $[C_{15}H_{20}F_9N_3NaO_2]^+$($[M+Na]^+$): 468.1304, found: 468.1302.

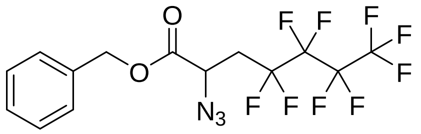

18

Following the **general procedure A**, the mixture of alkene (0.5 mmol), alkyl iodide (0.75 mmol), $TMSN_3$ (0.75 mmol) and TBPB (0.8 mmol) in DME (2 mL) was added to $Fe(OTf)_2$ (0.025 mmol) at room temperature for 3 minutes to afford **18**. Yield: 133 mg, 63%; clear oil; IR (KBr): υ 2964, 2125, 1749, 1457, 1234, 1134, 881 cm^{-1}; ^{1}H NMR (400 MHz, CDCl3) δ 7.34 – 7.25 (m, 5H), 5.18 (s, 2H), 4.24 (dd, J = 8.3, 4.5 Hz, 1H), 2.77 – 2.56 (m, 1H), 2.45 – 2.26 (m, 1H); ^{19}F NMR (376 MHz, CDCl3) δ -80.61 – -81.59 (m, 3F), -111.96 – -115.31 (m, 2F), -123.66 – -125.14 (m, 2F), -125.61 – -126.42 (m, 2F); ^{13}C NMR (100 MHz, CDCl3) δ 167.24, 133.47, 127.89, 127.77, 127.56, 121.79 – 103.39 (m, ($CF_2)_3CF_3$), 67.47, 54.55, 31.43 (t, J = 21.2 Hz, CH_2-CF_2); HRMS (ESI) calcd for $[C_{14}H_{10}F_9N_3NaO_2]^+$($[M+Na]^+$): 446.0522, found: 446.0526.

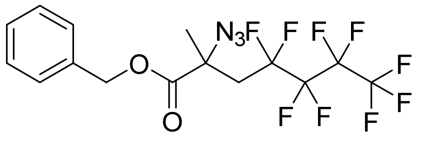

19

Following the **general procedure A**, the mixture of alkene (0.5 mmol), alkyl iodide (0.75 mmol), $TMSN_3$ (0.75 mmol) and TBPB (0.8 mmol) in DME (2 mL) was added to $Fe(OTf)_2$ (0.025 mmol) at room temperature for 3 minutes to afford **19**. Yield: 197 mg, 90%; clear oil; IR (KBr): υ 3038, 2963, 2126, 1749, 1355, 1235, 698 cm^{-1}; ^1H NMR (400 MHz, $CDCl_3$) δ 7.42 – 7.30 (m, 5H), 5.24 (s, 2H), 2.78 – 2.45 (m, 2H), 1.65 (s, 3H); ^{19}F NMR (376 MHz, $CDCl_3$) δ -79.23 – -83.17 (m, 3F), -108.62 – -111.15 (m, 1F), -111.90 – -114.26 (m, 1F), -124.02 – -125.12 (m, 2F), -125.37 – -126.53 (m, 2F); ^{13}C NMR (100 MHz, $CDCl_3$) δ 170.36, 134.54, 128.75, 128.67, 128.51, 68.42, 62.25, 37.59 (t, J = 20.4 Hz), 23.17; HRMS (ESI) calcd for $[C_{15}H_{12}F_9N_3NaO_2]^+$($[M+Na]^+$): 460.0678, found: 460.0683.

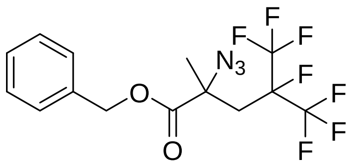

20

Following the **general procedure A**, the mixture of alkene (0.5 mmol), alkyl iodide (0.75 mmol), $TMSN_3$ (0.75 mmol) and TBPB (0.8 mmol) in DME (2 mL) was added to $Fe(OTf)_2$ (0.025 mmol) at room temperature for 3 minutes to afford **20**. Yield: 181 mg, 93%; clear oil; IR (KBr): υ 3038, 2963, 2124, 1748, 1458, 1230, 698 cm^{-1}; ^1H NMR (400 MHz, $CDCl_3$) δ 7.41 – 7.32 (m, 5H), 5.23 (dd, J = 30.4, 12.1 Hz, 2H), 2.60 (dt, J = 33.2, 16.0 Hz, 2H), 1.64 (s, 3H); ^{19}F NMR (376 MHz, $CDCl_3$) δ -76.17 – -77.45 (m, 6F), -185.54 – -186.38 (m, 1F); ^{13}C NMR (100 MHz, $CDCl_3$) δ 170.32, 134.54, 128.74, 128.68, 128.51, 68.37, 63.01 (d, J = 2.2 Hz), 35.04 (d, J = 18.3 Hz), 23.64 (d, J = 1.8 Hz); HRMS (ESI) calcd for $[C_{14}H_{12}F_7N_3NaO_2]^+$($[M+Na]^+$): 410.0710, found: 410.0713.

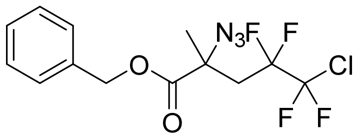

21

Following the **general procedure A**, the mixture of alkene (0.5 mmol), alkyl iodide (0.75 mmol), $TMSN_3$ (0.75 mmol) and TBPB (0.8 mmol) in DME (2 mL) was added to $Fe(OTf)_2$ (0.025 mmol) at room temperature for 3 minutes to afford **21**. Yield: 142 mg, 80%; clear oil; IR (KBr): υ 3037, 2961, 2125, 1748, 1458, 1260, 1152, 963, 750 cm^{-1}; ^1H NMR (400 MHz,

CDCl3) δ 7.43 – 7.29 (m, 5H), 5.23 (s, 2H), 2.78 – 2.47 (m, 2H), 1.64 (s, 3H); ^{19}F NMR (376 MHz, CDCl3) δ -71.33 – -73.24 (m, 2F), -109.05 – -110.59 (m, 1F), -112.03 – -113.68 (m, 1F); ^{13}C NMR (100 MHz, CDCl3) δ 170.38, 134.57, 128.72, 128.66, 128.47, 123.09 (tt, *J* = 299.1, 36.9 Hz), 115.82 (tt, *J* = 258.2, 33.4 Hz), 68.36, 62.39, 37.50 (t, *J* = 20.7 Hz), 23.10; HRMS (ESI) calcd for $[C_{13}H_{12}ClF_4N_3NaO_2]^+$($[M+Na]^+$): 376.0446, found: 376.0448.

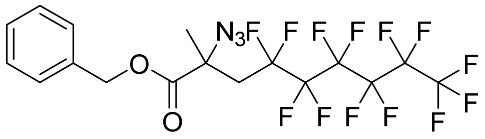

22

Following the **general procedure A**, the mixture of alkene (0.5 mmol), alkyl iodide (0.75 mmol), $TMSN_3$ (0.75 mmol) and TBPB (0.8 mmol) in DME (2 mL) was added to $Fe(OTf)_2$ (0.025 mmol) at room temperature for 3 minutes to afford **22**. Yield: 208 mg, 77%; clear oil; IR (KBr): υ 3038, 2964, 2127, 1749, 1365, 1245, 699 cm^{-1}; ^{1}H NMR (400 MHz, CDCl3) δ 7.42 – 7.31 (m, 5H), 5.24 (s, 2H), 2.79 – 2.45 (m, 2H), 1.65 (s, 3H); ^{19}F NMR (376 MHz, CDCl3) δ -79.00 – -83.01 (m, 3F), -108.18 – -110.97 (m, 1F), -111.32 – -114.23 (m, 1F), -119.29 – -125.27 (m, 6F), -125.66 – -128.32 (m, 2F); ^{13}C NMR (100 MHz, CDCl3) δ 170.38, 134.56, 128.75, 128.66, 128.52, 68.42, 62.27, 37.70 (t, *J* = 20.4 Hz), 23.16; HRMS (ESI) calcd for $[C_{17}H_{12}F_{13}N_3NaO_2]^+$($[M+Na]^+$): 560.0614, found: 560.0614.

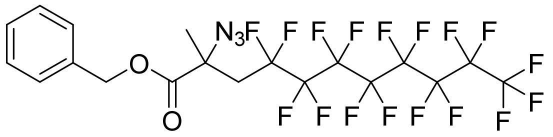

23

Following the **general procedure A**, the mixture of alkene (0.5 mmol), alkyl iodide (0.75 mmol), $TMSN_3$ (0.75 mmol) and TBPB (0.8 mmol) in DME (2 mL) was added to $Fe(OTf)_2$ (0.025 mmol) at room temperature for 3 minutes to afford **23**. Yield: 278 mg, 87%; white solid; mp 38-39 °C; IR (KBr): υ 3038, 2963, 2127, 1749, 1369, 1258, 700 cm^{-1}; ^{1}H NMR (400 MHz, CDCl3) δ 7.45 – 7.28 (m, 5H), 5.24 (s, 2H), 2.80 – 2.44 (m, 2H), 1.65 (s, 3H); ^{19}F NMR (376 MHz, CDCl3) δ -80.08 – -81.81 (m, 3F), -108.99 – -110.56 (m, 1F), -111.79 – -113.70 (m, 1F), -119.89 – -125.38 (m, 10F), -125.85 – -126.64 (m, 2F); ^{13}C NMR (100 MHz, CDCl3) δ 170.38, 134.56, 128.75, 128.66, 128.52, 68.43, 62.29, 37.72 (t, *J* = 20.2 Hz), 23.17; HRMS (ESI) calcd for $[C_{19}H_{12}F_{17}N_3NaO_2]^+$($[M+Na]^+$): 660.0550, found: 660.0546.

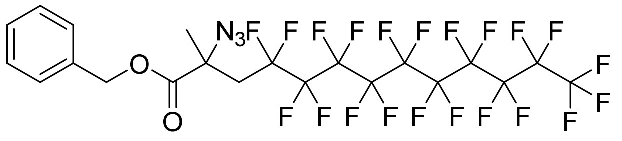

24

Following the **general procedure A**, the mixture of alkene (0.5 mmol), alkyl iodide (0.75 mmol), $TMSN_3$ (0.75 mmol) and TBPB (0.8 mmol) in DME (2 mL) was added to $Fe(OTf)_2$ (0.025 mmol) at room temperature for 3 minutes to afford **24**. Yield: 325 mg, 88%; white solid; mp 50-51 °C; IR (KBr): υ 3038, 2964, 2124, 1747, 1206, 1151, 747 cm^{-1}; ^1H NMR (400 MHz, $CDCl_3$) δ 7.42 – 7.30 (m, 5H), 5.24 (s, 2H), 2.78 – 2.45 (m, 2H), 1.65 (s, 3H); ^{19}F NMR (376 MHz, $CDCl_3$) δ -79.42 – -82.82 (m, 3F), -108.65 – -110.65 (m, 1F), -111.49 – -113.66 (m, 1F), -118.35 – -125.74 (m, 14F), -125.94 – -127.56 (m, 2F); ^{13}C NMR (100 MHz, $CDCl_3$) δ 170.40, 134.59, 128.75, 128.67, 128.53, 68.43, 62.29, 37.71 (t, J = 20.4 Hz), 23.12; HRMS (ESI) calcd for $[C_{21}H_{12}F_{21}N_3NaO_2]^+$($[M+Na]^+$): 760.0486, found: 760.0489.

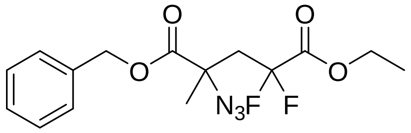

25

Following the **general procedure A**, the mixture of alkene (0.5 mmol), alkyl iodide (0.75 mmol), $TMSN_3$ (0.75 mmol) and TBPB (0.8 mmol) in DME (2 mL) was added to $Fe(OTf)_2$ (0.025 mmol) at room temperature for 3 minutes to afford **25**. Yield: 159 mg, 93%; clear oil; IR (KBr): υ 3036, 2986, 2127, 1748, 1457, 1261, 699 cm^{-1}; ^1H NMR (400 MHz, $CDCl_3$) δ 7.46 – 7.28 (m, 5H), 5.22 (s, 2H), 4.32 (q, J = 7.1 Hz, 2H), 2.80 – 2.51 (m, 2H), 1.62 (s, 3H), 1.34 (t, J = 7.1 Hz, 3H); ^{19}F NMR (376 MHz, $CDCl_3$) δ -100.34 (d, J = 270.0 Hz, 1F), -104.43 (d, J = 270.0 Hz, 1F); ^{13}C NMR (100 MHz, $CDCl_3$) δ 170.86, 163.27 (t, J = 31.8 Hz), 134.71, 128.60, 128.58, 128.27, 114.29 (dd, J = 253.2, 250.4 Hz), 68.13, 63.05, 62.39, 41.38 (t, J = 24.2 Hz), 22.95, 13.72; HRMS (ESI) calcd for $[C_{15}H_{17}F_2N_3NaO_4]^+$($[M+Na]^+$): 364.1079, found: 364.1083.

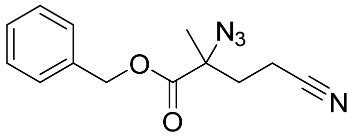

26

Following the **general procedure A**, the mixture of alkene (0.5 mmol), alkyl iodide (1.0 mmol), $TMSN_3$ (1.15 mmol) and TBPB (1.25 mmol) in DME (2 mL) was added to $Fe(OTf)_2$ (0.025 mmol) at room temperature for 10 minutes to afford **26**. Yield: 113 mg, 87%; clear oil;

IR (KBr): υ 3036, 2943, 2250, 2113, 1739, 1456, 1385, 1261, 1115, 699 cm^{-1}; ^1H NMR (400 MHz, $CDCl_3$) δ 7.43 – 7.32 (m, 5H), 5.24 (s, 2H), 2.48 – 2.36 (m, 1H), 2.33 – 2.15 (m, 2H), 1.98 – 1.86 (m, 1H), 1.58 (s, 3H); ^{13}C NMR (100 MHz, $CDCl_3$) δ 171.05, 134.66, 128.72, 128.70, 128.34, 118.66, 68.02, 64.81, 33.65, 22.57, 12.28; HRMS (ESI) calcd for $[C_{13}H_{14}N_4NaO_2]^+$($[M+Na]^+$): 281.1009, found: 281.1011.

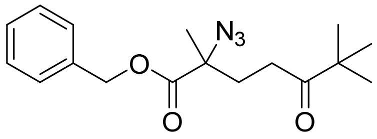

27

Following the **general procedure A**, the mixture of alkene (0.5 mmol), alkyl iodide (1.0 mmol), $TMSN_3$ (1.15 mmol) and TBPB (1.25 mmol) in DME (2 mL) was added to $Fe(OTf)_2$ (0.025 mmol) at room temperature for 3 minutes to afford **27**. Yield: 135 mg, 85%; clear oil; IR (KBr): υ 2970, 2872, 2105, 1739, 1707, 1458, 1261, 1172, 751, 699 cm^{-1}; ^1H NMR (400 MHz, $CDCl_3$) δ 7.41 – 7.29 (m, 5H), 5.21 (dd, J = 30.9, 12.1 Hz, 2H), 2.59 – 2.48 (m, 1H), 2.37 – 2.25 (m, 1H), 2.12 – 2.01 (m, 1H), 1.96 – 1.84 (m, 1H), 1.53 (s, 3H), 1.06 (s, 9H); ^{13}C NMR (100 MHz, $CDCl_3$) δ 214.13, 171.93, 135.09, 128.58, 128.49, 128.33, 67.44, 65.88, 43.99, 32.24, 31.05, 26.25, 22.80; HRMS (ESI) calcd for $[C_{17}H_{23}N_3NaO_3]^+$($[M+Na]^+$): 340.1632, found: 340.1633.

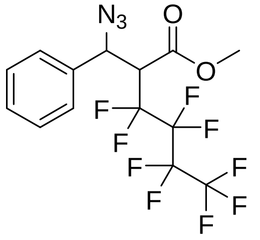

28

Following the **general procedure A**, the mixture of alkene (0.5 mmol), alkyl iodide (1.5 mmol), $TMSN_3$ (1.7 mmol) and TBPB (1.75 mmol) in DME (2 mL) was added to $Fe(OTf)_2$ (0.025 mmol) at room temperature for 15 minutes to afford **28**. Yield: 145 mg, 68% (dr = 9:1); clear oil; IR (KBr): υ 2959, 2109, 1757, 1438, 1237, 1137, 1022, 700 cm^{-1}; ^1H NMR (400 MHz, $CDCl_3$) δ 7.46 – 7.32 (m, 5H), 5.17 (d, J = 9.8 Hz, 1H), 3.83 (s, 3H), 3.69 – 3.56 (m, 1H); ^{19}F NMR (376 MHz, $CDCl_3$) δ -80.06 – -82.14 (m, 3F), -106.19 – -108.68 (m, 1F), -114.67 – -117.16 (m, 1F), -120.69 – -123.31 (m, 2F), -123.79 – -128.54 (m, 2F), ^{13}C NMR (100 MHz, $CDCl_3$) δ 165.39 (d, J = 11.5 Hz), 135.70 (d, J = 1.8 Hz), 129.47, 129.10, 127.43 (d, J = 1.4 Hz), 62.93 (d, J = 3.0 Hz), 53.06, 53.01 (t, J = 19.7 Hz); HRMS (ESI) calcd for $[C_{14}H_{10}F_9N_3NaO_2]^+$($[M+Na]^+$): 446.0522, found: 446.0520.

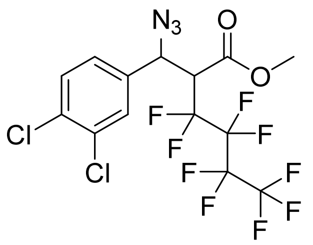

29

Following the **general procedure A**, the mixture of alkene (0.5 mmol), alkyl iodide (1.5 mmol), $TMSN_3$ (1.7 mmol) and TBPB (1.75 mmol) in DME (2 mL) was added to $Fe(OTf)_2$ (0.025 mmol) at room temperature for 15 minutes to afford **29**. Yield: 153 mg, 62%; clear oil; IR (KBr): υ 2960, 2112, 1756, 1471, 1237, 1034, 889, 822, 709 cm^{-1}; ^{1}H NMR (400 MHz, $CDCl_3$) δ 7.53 – 7.47 (m, 2H), 7.22 (dd, J = 8.3, 2.1 Hz, 1H), 5.14 (d, J = 9.7 Hz, 1H), 3.84 (s, 3H), 3.60 – 3.49 (m, 1H); ^{19}F NMR (376 MHz, $CDCl_3$) δ -80.22 – -81.86 (m, 3F), -105.54 – -108.02 (m, 1F), -114.46 – -116.91 (m, 1F), -120.68 – -123.25 (m, 2F), -123.70 – -128.01 (m, 2F); ^{13}C NMR (100 MHz, $CDCl_3$) δ 164.92 (d, J = 11.6 Hz), 136.14 (d, J = 1.7 Hz), 133.82, 133.47, 131.18, 129.40 (d, J = 1.5 Hz), 126.66 (d, J = 1.5 Hz), 61.87 (d, J = 3.2 Hz), 53.26, 53.07(t, J = 19.7 Hz); HRMS (ESI) calcd for $[C_{14}H_8Cl_2F_9N_3NaO_2]^+$($[M+Na]^+$): 513.9742, found: 513.9739.

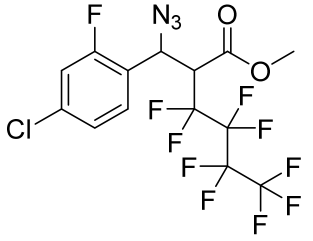

30

Following the **general procedure A**, the mixture of alkene (0.5 mmol), alkyl iodide (1.5 mmol), $TMSN_3$ (1.7 mmol) and TBPB (1.75 mmol) in DME (2 mL) was added to $Fe(OTf)_2$ (0.025 mmol) at room temperature for 15 minutes to afford **30**. Yield: 163 mg, 68% (dr = 12:1); clear oil; IR (KBr): υ 2960, 2111, 1758, 1613, 1491, 1237, 1023, 902, 724 cm^{-1}; ^{1}H NMR (400 MHz, $CDCl_3$) δ 7.37 – 7.30 (m, 1H), 7.26 – 7.15 (m, 2H), 5.43 (d, J = 9.5 Hz, 1H), 3.82 (s, 3H), 3.80 – 3.69 (m, 1H); ^{19}F NMR (376 MHz, $CDCl_3$) δ -80.38 – -81.79 (m, 3F), -108.14 – -109.67 (m, 1F), -114.38 (s, 1F), -115.24 – -116.82 (m, 1F), -120.83 – -123.49 (m, 2F), -124.09 – -127.83 (m, 2F); ^{13}C NMR (100 MHz, $CDCl_3$) δ 164.95 (d, J = 11.4 Hz), 159.97 (d, J = 252.5 Hz), 136.46 (d, J = 10.5 Hz), 129.94 (d, J = 3.7 Hz), 125.37 (d, J = 3.6 Hz), 121.85 (d, J = 13.2 Hz), 117.10 (d, J = 25.1 Hz), 57.11, 53.18, 51.53 (t, J = 19.8 Hz); HRMS (ESI) calcd for $[C_{14}H_8ClF_{10}N_3NaO_2]^+$($[M+Na]^+$): 498.0038, found: 498.0034.

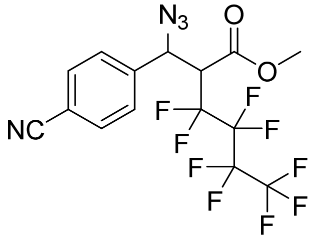

31

Following the **general procedure A**, the mixture of alkene (0.5 mmol), alkyl iodide (1.5 mmol), $TMSN_3$ (1.7 mmol) and TBPB (1.75 mmol) in DME (2 mL) was added to $Fe(OTf)_2$ (0.025 mmol) at room temperature for 15 minutes to afford **31**. Yield: 135 mg, 60%; clear oil; IR (KBr): υ 2961, 2233, 2113, 1756, 1351, 1237, 1138, 749 cm^{-1}; ^{1}H NMR (400 MHz, $CDCl_3$) δ 7.75 (d, *J* = 8.4 Hz, 2H), 7.51 (d, *J* = 8.3 Hz, 2H), 5.25 (d, *J* = 9.7 Hz, 1H), 3.85 (s, 3H), 3.65 – 3.53 (m, 1H); ^{19}F NMR (376 MHz, $CDCl_3$) δ -80.41 – -81.74 (m, 3F), -105.38 – -107.83 (m, 1F), -114.21 – -116.62 (m, 1F), -120.78 – -123.47 (m, 2F), -123.85 – -127.92 (m, 2F); ^{13}C NMR (100 MHz, $CDCl_3$) δ 164.78 (d, *J* = 11.6 Hz), 141.00 (d, *J* = 1.6 Hz), 132.90, 128.25 (d, *J* = 1.6 Hz), 117.93, 113.51, 62.36 (d, *J* = 3.1 Hz), 53.28, 53.04 (t, *J* = 19.8 Hz); HRMS (EI) calcd for $[C_{15}H_9F_9N_2O_2]^+([M-N_2]^+)$: 420.0520, found: 420.0527.

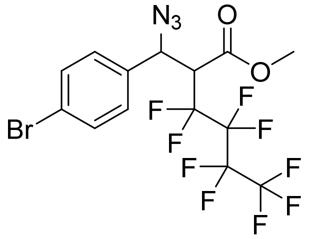

32

Following the **general procedure A**, the mixture of alkene (0.5 mmol), alkyl iodide (1.5 mmol), $TMSN_3$ (1.7 mmol) and TBPB (1.75 mmol) in DME (2 mL) was added to $Fe(OTf)_2$ (0.025 mmol) at room temperature for 15 minutes to afford **32**. Yield: 177 mg, 70%; clear oil; IR (KBr): υ 2959, 2111, 1756, 1438, 1237, 1014, 879, 821, 733 cm^{-1}; ^{1}H NMR (400 MHz, $CDCl_3$) δ 7.57 (d, *J* = 8.5 Hz, 2H), 7.25 (d, *J* = 8.5 Hz, 2H), 5.15 (d, *J* = 9.8 Hz, 1H), 3.83 (s, 3H), 3.63 – 3.50 (m, 1H); ^{19}F NMR (376 MHz, $CDCl_3$) δ -80.38 – -81.67 (m, 3F), -106.09 – -107.98 (m, 1F), -114.92 – -116.78 (m, 1F), -120.92 – -123.22 (m, 2F), -123.91 – -127.72 (m, 2F); ^{13}C NMR (100 MHz, $CDCl_3$) δ 165.13 (d, *J* = 11.5 Hz), 134.86 (d, *J* = 1.8 Hz), 132.37, 129.05 (d, *J* = 1.5 Hz), 123.62, 62.38 (d, *J* = 3.2 Hz), 53.16, 53.02 (t, *J* = 19.7 Hz); HRMS (ESI) calcd for $[C_{14}H_9BrF_9N_3NaO_2]^+([M+Na]^+)$: 523.9627, found: 523.9626.

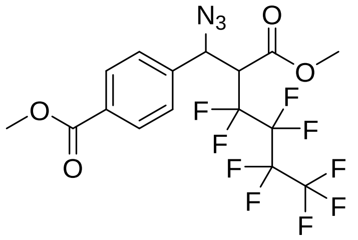

33

Following the **general procedure A**, the mixture of alkene (0.5 mmol), alkyl iodide (1.5 mmol), $TMSN_3$ (1.7 mmol) and TBPB (1.75 mmol) in DME (2 mL) was added to $Fe(OTf)_2$ (0.025 mmol) at room temperature for 15 minutes to afford **33**. Yield: 138 mg, 57%; clear oil; IR (KBr): υ 2959, 2112, 1730, 1614, 1438, 1236, 1021, 883, 705 cm^{-1}; ^{1}H NMR (400 MHz, $CDCl_3$) δ 8.11 (d, J = 8.4 Hz, 2H), 7.46 (d, J = 8.3 Hz, 2H), 5.24 (d, J = 9.7 Hz, 1H), 3.94 (s, 3H), 3.84 (s, 3H), 3.69 – 3.57 (m, 1H); ^{19}F NMR (376 MHz, $CDCl_3$) δ -80.75 – -81.41 (m, 3F), -105.72 – -108.23 (m, 1F), -114.46 – -117.11 (m, 1F), -120.70 – -123.39 (m, 2F), -123.90 – -128.00 (m, 2F); ^{13}C NMR (100 MHz, $CDCl_3$) δ 166.26, 165.04 (d, J = 11.5 Hz), 140.55 (d, J = 1.6 Hz), 131.23, 130.36, 127.51 (d, J = 1.3 Hz), 62.50 (d, J = 2.9 Hz), 53.16, 52.98 (t, J = 19.8 Hz), 52.26; HRMS (ESI) calcd for $[C_{16}H_{12}F_9N_3NaO_4]^+$($[M+Na]^+$): 504.0576, found: 504.0575.

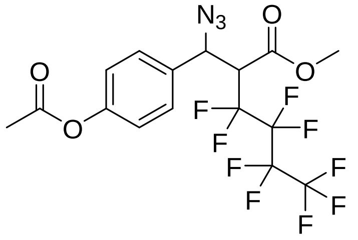

34

Following the **general procedure A**, the mixture of alkene (0.5 mmol), alkyl iodide (1.5 mmol), $TMSN_3$ (1.7 mmol) and TBPB (1.75 mmol) in DME (2 mL) was added to $Fe(OTf)_2$ (0.025 mmol) at room temperature for 15 minutes to afford **34**. Yield: 140 mg, 58%; clear oil; IR (KBr): υ 2960, 2112, 1758, 1509, 1438, 1205, 1019, 913, 744 cm^{-1}; ^{1}H NMR (400 MHz, $CDCl_3$) δ 7.38 (d, J = 8.6 Hz, 2H), 7.18 (d, J = 8.6 Hz, 2H), 5.18 (d, J = 9.6 Hz, 1H), 3.82 (s, 3H), 3.65 – 3.53 (m, 1H), 2.30 (s, 3H); ^{19}F NMR (376 MHz, $CDCl_3$) δ -80.75 – -81.38 (m, 3F), -106.01 – -108.59 (m, 1F), -114.58 – -117.21 (m, 1F), -120.74 – -123.33 (m, 2F), -123.74 – -128.05 (m, 2F); ^{13}C NMR (100 MHz, $CDCl_3$) δ 168.94, 165.21 (d, J = 11.5 Hz), 151.32, 133.26 (d, J = 1.7 Hz), 128.49 (d, J = 1.3 Hz), 122.24, 62.33 (d, J = 3.0 Hz), 53.10 (t, J = 19.7 Hz), 53.07, 21.05; HRMS (ESI) calcd for $[C_{16}H_{12}F_9N_3NaO_4]^+$($[M+Na]^+$): 504.0576, found: 504.0574.

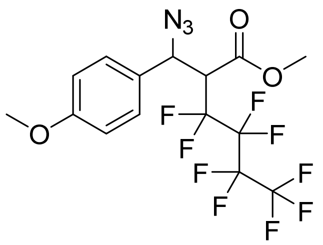

35

Following the **general procedure A**, the mixture of vinylarene (0.5 mmol), alkyl iodide (1.5 mmol), $TMSN_3$ (1.7 mmol) and TBPB (1.75 mmol) in DME (2 mL) was added to $Fe(OTf)_2$ (0.025 mmol) at room temperature for 15 minutes to afford **35**. Yield: 128 mg, 56%; clear oil; IR (KBr): υ 2960, 2843, 2110, 1756, 1613, 1516, 1439, 1239, 876 cm^{-1}; ^1H NMR (400 MHz, $CDCl_3$) δ 7.29 (d, *J* = 8.7 Hz, 2H), 6.94 (d, *J* = 8.7 Hz, 2H), 5.14 (d, *J* = 9.9 Hz, 1H), 3.86 – 3.79 (m, 6H), 3.66 – 3.52 (m, 1H); ^{19}F NMR (376 MHz, $CDCl_3$) δ -78.95 – -83.04 (m, 3F), -106.09 – -109.03 (m, 1F), -114.43 – -117.51 (m, 1F), -120.61 – -123.39 (m, 2F), -124.27 – -127.51 (m, 2F); ^{13}C NMR (100 MHz, $CDCl_3$) δ 165.55 (d, *J* = 11.4 Hz), 160.31, 128.76 (d, *J* = 1.2 Hz), 127.58 (d, *J* = 1.8 Hz), 114.43, 119.86 – 106.20 (m, ($CF_2)_3CF_3$), 62.59 (d, *J* = 3.4 Hz), 55.25, 53.07 (t, *J* = 19.6 Hz), 53.04; HRMS (ESI) calcd for $[C_{15}H_{12}F_9N_3NaO_3]^+$($[M+Na]^+$): 476.0627, found:476.0628.

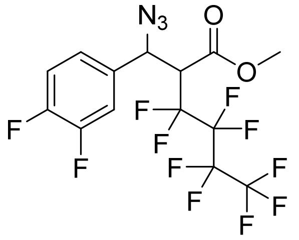

36

Following the **general procedure A**, the mixture of vinylarene (0.5 mmol), alkyl iodide (1.5 mmol), $TMSN_3$ (1.7 mmol) and TBPB (1.75 mmol) in DME (2 mL) was added to $Fe(OTf)_2$ (0.025 mmol) at room temperature for 15 minutes to afford **36**. Yield: 159 mg, 69% (dr = 13:1); clear oil; IR (KBr): υ 2962, 2112, 1755, 1614, 1522, 1440, 1260, 1022, 886, 747 cm^{-1}; ^1H NMR (400 MHz, $CDCl_3$) δ 7.27 – 7.18 (m, 2H), 7.17 – 7.10 (m, 1H), 5.16 (d, *J* = 9.8 Hz, 1H), 3.84 (s, 3H), 3.59 – 3.48 (m, 1H); ^{19}F NMR (376 MHz, $CDCl_3$) δ -80.13 – -82.12 (m, 3F), -105.56 – -108.58 (m, 1F), -114.31 – -116.98 (m, 1F), -120.84 – -123.23 (m, 2F), -123.56 – -128.41 (m, 2F), -135.41 (d, *J* = 21.1 Hz, 1F), -135.69 (d, *J* = 21.1 Hz, 1F); ^{13}C NMR (100 MHz, $CDCl_3$) δ 165.02 (d, *J* = 11.5 Hz), 150.87 (dd, *J* = 259.7, 20.8 Hz), 150.61 (dd, *J* = 253.5, 15.7 Hz), 132.95, 123.88, 118.08 (d, *J* = 17.6 Hz), 116.58 (d, *J* = 18.2 Hz),

62.03 (d, J = 3.2 Hz), 53.22 (t, J = 19.7 Hz), 53.19; HRMS (EI) calcd for $[C_{14}H_8F_{11}NO_2]^+$($[M-N_2]^+$): 431.0379, found: 431.0376.

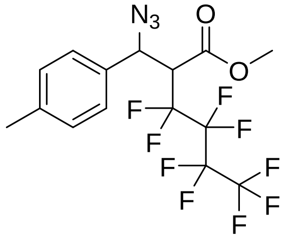

37

Following the **general procedure A**, the mixture of vinylarene (0.5 mmol), alkyl iodide (1.5 mmol), $TMSN_3$ (1.7 mmol) and TBPB (1.75 mmol) in DME (2 mL) was added to $Fe(OTf)_2$ (0.025 mmol) at room temperature for 15 minutes to afford **37**. Yield: 136 mg, 62% (dr = 19:1); clear oil; IR (KBr): υ 2959, 2110, 1757, 1517, 1438, 1236, 1022, 878, 720 cm^{-1}; ^1H NMR (400 MHz, $CDCl_3$) δ 7.33 – 7.25 (m, 4H), 5.19 (d, J = 9.8 Hz, 1H), 3.88 (s, 3H), 3.73 – 3.61 (m, 1H), 2.42 (s, 3H); ^{19}F NMR (376 MHz, $CDCl_3$) δ -79.93 – -82.13 (m, 3F), -106.09 – -108.76 (m, 1F), -114.84 – -117.45 (m, 1F), -120.91 – -123.29 (m, 2F), -124.01 – -127.72 (m, 2F); ^{13}C NMR (100 MHz, $CDCl_3$) δ 165.48 (d, J = 11.5 Hz), 139.44, 132.63 (d, J = 1.8 Hz), 129.77, 127.33 (d, J = 1.4 Hz), 62.75 (d, J = 3.3 Hz), 53.02, 52.96 (t, J = 19.6 Hz), 21.17; HRMS (ESI) calcd for $[C_{15}H_{12}F_9N_3NaO_2]^+$($[M+Na]^+$): 460.0678, found: 460.0681.

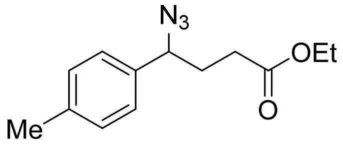

38

Following the **general procedure A**, the mixture of vinylarene (0.5 mmol), alkyl iodide (1.0 mmol), $TMSN_3$ (1.0 mmol) and TBPB (1.0 mmol) in DME (2 mL) was added to $Fe(OTf)_2$ (0.025 mmol) at room temperature for 10 minutes to afford **38**. Yield: 112 mg, 91%; clear oil; IR (KBr): υ 2982, 2097, 1734, 1446, 1250, 1181, 1024, 816 cm^{-1}; ^1H NMR (400 MHz, $CDCl_3$) δ 7.23 – 7.15 (m, 4H), 4.49 (t, J = 7.2 Hz, 1H), 4.12 (q, J = 7.1 Hz, 2H), 2.41 – 2.30 (m, 5H), 2.15 – 1.99 (m, 2H), 1.25 (t, J = 7.1 Hz, 3H); ^{13}C NMR (100 MHz, $CDCl_3$) δ 172.75, 138.24, 135.92, 129.55, 126.83, 65.17, 60.53, 31.30, 30.87, 21.15, 14.21; HRMS (ESI) calcd for $[C_{13}H_{17}N_3NaO_2]^+$($[M+Na]^+$): 270.1213, found: 270.1213.

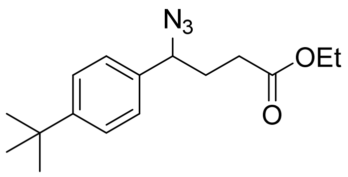

39

Following the **general procedure A**, the mixture of vinylarene (0.5 mmol), alkyl iodide (1.0 mmol), $TMSN_3$ (1.0 mmol) and TBPB (1.0 mmol) in DME (2 mL) was added to $Fe(OTf)_2$ (0.025 mmol) at room temperature for 10 minutes to afford **39**. Yield: 133 mg, 92%; clear oil; IR (KBr): υ 2964, 2099, 1735, 1463, 1251, 1179, 1025, 831 cm^{-1}; ^1H NMR (400 MHz, $CDCl_3$) δ 7.39 (d, J = 7.6 Hz, 2H), 7.23 (d, J = 7.6 Hz, 2H), 4.50 (t, J = 7.1 Hz, 1H), 4.12 (q, J = 7.0 Hz, 2H), 2.37 (t, J = 7.3 Hz, 2H), 2.09 (m, 2H), 1.32 (s, 9H), 1.25 (t, J = 7.0 Hz, 3H); ^{13}C NMR (100 MHz, $CDCl_3$) δ 172.77, 151.37, 135.94, 126.55, 125.76, 65.12, 60.53, 34.61, 31.30, 30.90, 14.22; HRMS (ESI) calcd for $[C_{16}H_{23}N_3NaO_2]^+$($[M+Na]^+$): 312.1682, found: 312.1679.

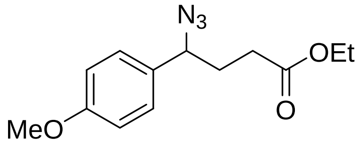

40

Following the **general procedure A**, the mixture of vinylarene (0.5 mmol), alkyl iodide (1.0 mmol), $TMSN_3$ (1.0 mmol) and TBPB (1.0 mmol) in DME (2 mL) was added to $Fe(OTf)_2$ (0.025 mmol) at room temperature for 10 minutes to afford **40**. Yield: 119 mg, 90%; pale yellow oil; IR (KBr): υ 2936, 2098, 1733, 1514, 1463, 1251, 1177, 1033, 831 cm^{-1}; ^1H NMR (400 MHz, $CDCl_3$) δ 7.26 (d, J = 8.3 Hz, 2H), 6.93 (d, J = 8.3 Hz, 2H), 4.50 (t, J = 7.2 Hz, 1H), 4.15 (q, J = 7.1 Hz, 2H), 3.84 (s, 3H), 2.37 (t, J = 7.3 Hz, 2H), 2.17 – 2.00 (m, 2H), 1.27 (t, J = 7.1 Hz, 3H); ^{13}C NMR (100 MHz, $CDCl_3$) δ 172.77, 159.63, 130.94, 128.16, 114.22, 64.92, 60.54, 55.30, 31.28, 30.91, 14.22; HRMS (ESI) calcd for $[C_{13}H_{17}N_3NaO_3]^+$($[M+Na]^+$): 286.1162, found: 286.1163.

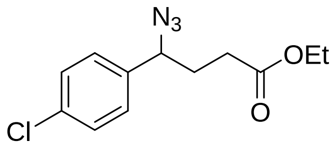

41

Following the **general procedure A**, the mixture of vinylarene (0.5 mmol), alkyl iodide (1.0 mmol), $TMSN_3$ (1.0 mmol) and TBPB (1.0 mmol) in DME (2 mL) was added to $Fe(OTf)_2$ (0.025 mmol) at 50 ^oC for 10 minutes to afford **41**. Yield: 99 mg, 74%; pale yellow oil; IR

(KBr): υ 2981, 2100, 1733, 1375, 1249, 1184, 1092, 826 cm^{-1}; ^{1}H NMR (400 MHz, $CDCl_3$) δ 7.37 (d, *J* = 7.7 Hz, 2H), 7.25 (d, *J* = 7.9 Hz, 2H), 4.53 (t, *J* = 7.1 Hz, 1H), 4.13 (q, *J* = 7.0 Hz, 2H), 2.36 (t, *J* = 7.3 Hz, 2H), 2.06 (m, 2H), 1.25 (t, *J* = 7.0 Hz, 3H); ^{13}C NMR (100 MHz, $CDCl_3$) δ 172.57, 137.60, 134.26, 129.11, 128.22, 64.60, 60.65, 31.38, 30.62, 14.20; HRMS (ESI) calcd for $[C_{12}H_{14}ClN_3NaO_2]^+$($[M+Na]^+$): 290.0667, found: 290.0664.

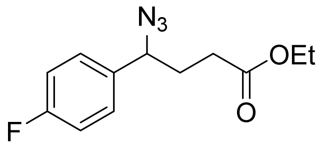

42

Following the **general procedure A**, the mixture of vinylarene (0.5 mmol), alkyl iodide (1.0 mmol), $TMSN_3$ (1.0 mmol) and TBPB (1.0 mmol) in DME (2 mL) was added to $Fe(OTf)_2$ (0.025 mmol) at 50 °C for 10 minutes to afford **42**. Yield: 101 mg, 80%; yellow oil; IR (KBr): υ 2983, 2100, 1733, 1446, 1228, 1160, 1026, 836 cm^{-1}; ^{1}H NMR (400 MHz, $CDCl_3$) δ 7.35 – 7.27 (m, 2H), 7.15 – 7.06 (m, 2H), 4.55 (t, *J* = 7.1 Hz, 1H), 4.15 (q, *J* = 7.1 Hz, 2H), 2.39 (t, *J* = 7.3 Hz, 2H), 2.16 – 2.00 (m, 2H), 1.28 (t, *J* = 7.1 Hz, 3H); ^{19}F NMR (376 MHz, $CDCl_3$) δ -113.40 (s, 1F). ^{13}C NMR (100 MHz, $CDCl_3$) δ 172.60, 162.59 (d, *J* = 247.2 Hz), 134.88 (d, *J* = 3.3 Hz), 128.59 (d, *J* = 8.2 Hz), 115.83 (d, *J* = 21.6 Hz), 64.61, 60.61, 31.45, 30.70, 14.19; HRMS (ESI) calcd for $[C_{12}H_{14}FN_3NaO_2]^+$($[M+Na]^+$): 274.0962, found: 274.0962.

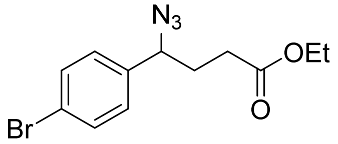

43

Following the **general procedure A**, the mixture of vinylarene (0.5 mmol), alkyl iodide (1.0 mmol), $TMSN_3$ (1.0 mmol) and TBPB (1.0 mmol) in DME (2 mL) was added to $Fe(OTf)_2$ (0.025 mmol) at 50 °C for 10 minutes to afford **43**. Yield: 132 mg, 85%; yellow oil; IR (KBr): υ 2981, 2099, 1732, 1489, 1249, 1184, 1010, 822 cm^{-1}; ^{1}H NMR (400 MHz, $CDCl_3$) δ 7.54 (d, *J* = 8.2 Hz, 2H), 7.21 (d, *J* = 8.2 Hz, 2H), 4.54 (t, *J* = 7.1 Hz, 1H), 4.15 (q, *J* = 7.1 Hz, 2H), 2.50 – 2.23 (m, 2H), 2.11 – 1.97 (m, 2H), 1.27 (t, *J* = 7.1 Hz, 3H); ^{13}C NMR (100 MHz, $CDCl_3$) δ 172.56, 138.13, 132.07, 128.54, 122.38, 64.65, 60.65, 31.35, 30.60, 14.21; HRMS (ESI) calcd for $[C_{12}H_{14}BrN_3NaO_2]^+$($[M+Na]^+$): 334.0162, found: 334.0159.

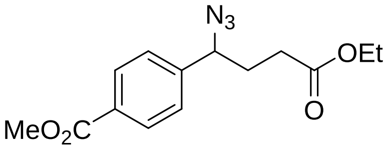

44

Following the **general procedure A**, the mixture of vinylarene (0.5 mmol), alkyl iodide (1.0 mmol), $TMSN_3$ (1.0 mmol) and TBPB (1.0 mmol) in DME (2 mL) was added to $Fe(OTf)_2$ (0.025 mmol) at 50 °C for 10 minutes to afford **44**. Yield: 105 mg, 73%; pale yellow semi-solid; IR (KBr): υ 2983, 2954, 2100, 1728, 1436, 1281, 1182, 1020, 708 cm^{-1}; ^1H NMR (400 MHz, $CDCl_3$) δ 8.06 (d, J = 7.8 Hz, 2H), 7.39 (d, J = 7.8 Hz, 2H), 4.62 (t, J = 7.0 Hz, 1H), 4.13 (q, J = 7.0 Hz, 2H), 3.93 (s, 3H), 2.39 – 2.35 (m, 2H), 2.13 – 1.99 (m, 2H), 1.26 (t, J = 7.1 Hz, 3H); ^{13}C NMR (100 MHz, $CDCl_3$) δ 172.54, 166.57, 144.12, 130.25, 130.22, 126.84, 64.86, 60.66, 52.22, 31.39, 30.55, 14.20; HRMS (ESI) calcd for $[C_{14}H_{17}N_3NaO_4]^+$($[M+Na]^+$): 314.1111, found: 314.1112.

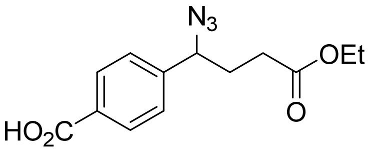

45

Following the **general procedure A**, the mixture of vinylarene (0.5 mmol), alkyl iodide (1.0 mmol), $TMSN_3$ (1.0 mmol) and TBPB (1.0 mmol) in DME (2 mL) was added to $Fe(OTf)_2$ (0.025 mmol) at 50 °C for 10 minutes to afford **45**. Yield: 97 mg, 70%; pale yellow solid; mp 96 °C; IR (KBr): υ 2983, 2101, 1731, 1695, 1422, 1287, 1180, 1019, 857 cm^{-1}; ^1H NMR (400 MHz, $CDCl_3$) δ 8.15 (d, J = 8.0 Hz, 2H), 7.44 (d, J = 8.0 Hz, 2H), 4.66 (t, J = 7.0 Hz, 1H), 4.14 (q, J = 7.1 Hz, 2H), 2.46 – 2.33 (m, 2H), 2.14 – 2.03 (m, 2H), 1.26 (t, J = 7.2 Hz, 3H) Note: –COO***H*** was not observed; ^{13}C NMR (100 MHz, $CDCl_3$) δ 172.61, 171.39, 145.15, 130.87, 129.39, 126.97, 64.85, 60.73, 31.42, 30.54, 14.20; HRMS (ESI) calcd for $[C_{13}H_{15}N_3NaO_4]^+$($[M+Na]^+$): 300.0955, found: 300.0956.

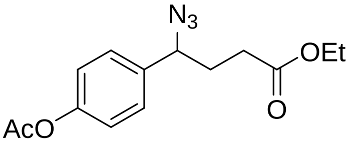

46

Following the **general procedure A**, the mixture of vinylarene (0.5 mmol), alkyl iodide (1.0 mmol), $TMSN_3$ (1.0 mmol) and TBPB (1.0 mmol) in DME (2 mL) was added to $Fe(OTf)_2$ (0.025 mmol) at room temperature for 10 minutes to afford **46**. Yield: 105 mg, 74%;

colourless thick liquid; IR (KBr): υ 2982, 2931, 2099, 1768, 1733, 1508, 1197, 1017, 849 cm^{-1}; ^{1}H NMR (400 MHz, $CDCl_3$) δ 7.32 (d, *J* = 8.6 Hz, 2H), 7.12 (d, *J* = 8.6 Hz, 2H), 4.55 (dd, *J* = 7.8, 6.5 Hz, 1H), 4.13 (q, *J* = 7.1 Hz, 2H), 2.42 – 2.34 (m, 2H), 2.30 (s, 3H), 2.13 – 1.99 (m, 2H), 1.25 (t, *J* = 7.1 Hz, 3H); ^{13}C NMR (100 MHz, $CDCl_3$) δ 172.64, 169.28, 150.57, 136.67, 127.91, 122.02, 64.74, 60.61, 31.48, 30.71, 21.14, 14.21; HRMS (ESI) calcd for $[C_{14}H_{17}N_3NaO_4]^+$($[M+Na]^+$): 314.1111, found: 314.1111.

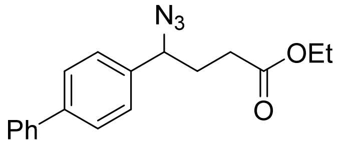

47

Following the **general procedure A**, the mixture of vinylarene (0.5 mmol), alkyl iodide (1.0 mmol), $TMSN_3$ (1.0 mmol) and TBPB (1.0 mmol) in DME (2 mL) was added to $Fe(OTf)_2$ (0.025 mmol) at room temperature for 10 minutes to afford **47**. Yield: 127 mg, 82%; clear oil; IR (KBr): υ 2980, 2098, 1732, 1487, 1249, 1116, 1026, 765 cm^{-1}; ^{1}H NMR (400 MHz, $CDCl_3$) δ 7.64 – 7.56 (m, 4H), 7.48 – 7.41 (m, 2H), 7.40 – 7.33 (m, 3H), 4.63 – 4.54 (m, 1H), 4.13 (q, *J* = 7.1 Hz, 2H), 2.40 (t, *J* = 7.4 Hz, 2H), 2.19 – 2.05 (m, 2H), 1.26 (t, *J* = 7.1 Hz, 3H); ^{13}C NMR (100 MHz, $CDCl_3$) δ 172.73, 141.38, 140.47, 137.98, 128.84, 127.61, 127.52, 127.32, 127.11, 65.09, 60.61, 31.38, 30.83, 14.23; HRMS (ESI) calcd for $[C_{18}H_{19}N_3NaO_2]^+$($[M+Na]^+$): 332.1369, found: 332.1370.

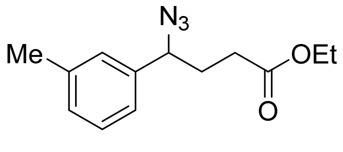

48

Following the **general procedure A**, the mixture of vinylarene (0.5 mmol), alkyl iodide (1.0 mmol), $TMSN_3$ (1.0 mmol) and TBPB (1.0 mmol) in DME (2 mL) was added to $Fe(OTf)_2$ (0.025 mmol) at room temperature for 10 minutes to afford **48**. Yield: 103 mg, 84%; clear oil; IR (KBr): υ 2980, 2098, 1734, 1447, 1375, 1253, 1179, 1097, 705 cm^{-1}; ^{1}H NMR (400 MHz, $CDCl_3$) δ 7.30 – 7.23 (m, 1H), 7.17 – 7.07 (m, 3H), 4.49 (t, *J* = 7.1 Hz, 1H), 4.13 (q, *J* = 7.0 Hz, 2H), 2.40 – 2.32 (m, 5H), 2.15 – 1.99 (m 2H), 1.25 (t, *J* = 7.1 Hz, 3H); ^{13}C NMR (100 MHz, $CDCl_3$) δ 172.77, 138.93, 138.61, 129.20, 128.75, 127.53, 123.92, 65.38, 60.55, 31.35, 30.86, 21.47, 14.22; HRMS (ESI) calcd for $[C_{13}H_{17}N_3NaO_2]^+$($[M+Na]^+$): 270.1213, found: 270.1213.

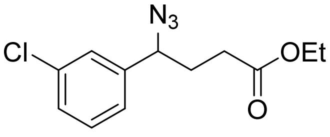

49

Following the **general procedure A**, the mixture of vinylarene (0.5 mmol), alkyl iodide (1.0 mmol), $TMSN_3$ (1.0 mmol) and TBPB (1.0 mmol) in DME (2 mL) was added to $Fe(OTf)_2$ (0.025 mmol) at 50 °C for 10 minutes to afford **49**. Yield: 101 mg, 75%; pale yellow oil; IR (KBr): υ 2982, 2101, 1733, 1575, 1477, 1249, 1183, 1026, 787 cm^{-1}; ^1H NMR (400 MHz, $CDCl_3$) δ 7.35 – 7.29 (m, 3H), 7.23 – 7.17 (m, 1H), 4.53 (t, *J* = 7.1 Hz, 1H), 4.13 (q, *J* = 7.1 Hz, 2H), 2.44 – 2.31 (m, 2H), 2.15 – 1.97 (m, 2H), 1.26 (t, *J* = 7.1 Hz, 3H); ^{13}C NMR (100 MHz, $CDCl_3$) δ 172.54, 141.22, 134.82, 130.20, 128.62, 127.01, 125.01, 64.69, 60.66, 31.41, 30.59, 14.21; HRMS (ESI) calcd for $[C_{12}H_{14}ClN_3NaO_2]^+([M+Na]^+)$: 290.0667, found: 290.0665.

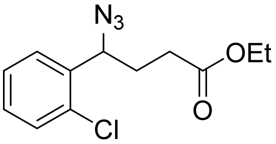

50

Following the **general procedure A**, the mixture of vinylarene (0.5 mmol), alkyl iodide (1.0 mmol), $TMSN_3$ (1.0 mmol) and TBPB (1.0 mmol) in DME (2 mL) was added to $Fe(OTf)_2$ (0.025 mmol) at 50 °C for 10 minutes to afford **50**. Yield: 102 mg, 76%; yellow thick liquid; IR (KBr): υ 2981, 2100, 1734, 1442, 1251, 1182, 1036, 757 cm^{-1}; ^1H NMR (400 MHz, $CDCl_3$) δ 7.45 (d, *J* = 7.6 Hz, 1H), 7.40 (d, *J* = 7.9 Hz, 1H), 7.33 (t, *J* = 7.5 Hz, 1H), 7.30 – 7.23 (m, 1H), 5.15 – 5.08 (m, 1H), 4.13 (q, *J* = 7.1 Hz, 2H), 2.42 (t, *J* = 7.5 Hz, 2H), 2.15 – 2.02 (m, 2H), 1.26 (t, *J* = 7.1 Hz, 3H); ^{13}C NMR (100 MHz, $CDCl_3$) δ 172.57, 136.91, 132.92, 129.87, 129.36, 127.76, 127.46, 61.40, 60.63, 30.67, 30.49, 14.21; HRMS (ESI) calcd for $[C_{12}H_{14}ClN_3NaO_2]^+([M+Na]^+)$: 290.0667, found: 290.0668.

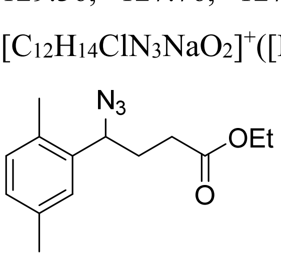

51

Following the **general procedure A**, the mixture of vinylarene (0.5 mmol), alkyl iodide (1.0 mmol), $TMSN_3$ (1.0 mmol) and TBPB (1.0 mmol) in DME (2 mL) was added to $Fe(OTf)_2$ (0.025 mmol) at -10 °C to room temperature for 2 hours to afford **51**. Yield: 103 mg, 78%;

clear oil; IR (KBr): υ 2978, 2928, 2098, 1734, 1456, 1374, 1253, 1178, 1026, 812 cm^{-1}; ^{1}H NMR (400 MHz, $CDCl_3$) δ 7.15 (s, 1H), 7.07 (d, *J* = 7.7 Hz, 1H), 7.02 (d, *J* = 7.7 Hz, 1H), 4.77 (t, *J* = 7.1 Hz, 1H), 4.14 (q, *J* = 7.1 Hz, 2H), 2.42 (td, J = 7.2, 2.1 Hz, 2H), 2.33 (s, 3H), 2.32 (s, 3H), 2.08 – 2.01 (m, 2H), 1.26 (t, *J* = 7.1 Hz, 3H); ^{13}C NMR (100 MHz, $CDCl_3$) δ 172.85, 136.97, 136.04, 132.27, 130.74, 128.80, 126.77, 61.62, 60.58, 30.88, 30.57, 21.13, 18.75, 14.23; HRMS (ESI) calcd for $[C_{14}H_{19}N_3NaO_2]^+$($[M+Na]^+$): 284.1369, found: 284.1369.

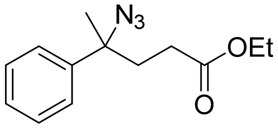

52

Following the **general procedure A**, the mixture of vinylarene (0.5 mmol), alkyl iodide (1.0 mmol), $TMSN_3$ (1.0 mmol) and TBPB (1.0 mmol) in DME (2 mL) was added to $Fe(OTf)_2$ (0.025 mmol) at -10 °C to room temperature for 2 hours to afford **52**. Yield: 79 mg, 64%; clear oil; IR (KBr): υ 2981, 2107, 1735, 1446, 1381, 1254, 1180, 1095, 763 cm^{-1}; ^{1}H NMR (400 MHz, $CDCl_3$) δ 7.42 – 7.32 (m, 4H), 7.32 – 7.23 (m, 1H), 4.06 (q, *J* = 6.7 Hz, 2H), 2.36 – 2.24 (m, 1H), 2.22 – 2.05 (m, 3H), 1.71 (s, 3H), 1.21 (t, *J* = 7.1 Hz, 3H); ^{13}C NMR (100 MHz, $CDCl_3$) δ 172.99, 142.56, 128.67, 127.51, 125.50, 66.18, 60.50, 37.15, 29.57, 25.82, 14.16; HRMS (ESI) calcd for $[C_{13}H_{17}N_3NaO_2]^+$($[M+Na]^+$): 270.1213, found: 270.1212.

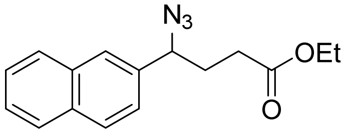

53

Following the **general procedure A**, the mixture of vinylarene (0.5 mmol), alkyl iodide (1.0 mmol), $TMSN_3$ (1.0 mmol) and TBPB (1.0 mmol) in DME (2 mL) was added to $Fe(OTf)_2$ (0.025 mmol) at 50 °C for 10 minutes to afford **53**. Yield: 142 mg, 69%; pale yellow oil; IR (KBr): υ 2980, 2099, 1732, 1445, 1374, 1250, 1180, 1025, 749 cm^{-1}; ^{1}H NMR (400 MHz, $CDCl_3$) δ 7.95 – 7.84 (m, 3H), 7.79 (s, 1H), 7.63 – 7.50 (m, 2H), 7.46 (d, *J* = 8.5 Hz, 1H), 4.74 (t, *J* = 7.1 Hz, 1H), 4.15 (q, *J* = 7.1 Hz, 2H), 2.43 (t, *J* = 7.4 Hz, 2H), 2.28 – 2.12 (m, 2H), 1.27 (t, *J* = 7.1 Hz, 3H); ^{13}C NMR (100 MHz, $CDCl_3$) δ 172.73, 136.34, 133.26, 133.17, 128.98, 128.03, 127.75, 126.52, 126.40, 126.24, 124.25, 65.55, 60.59, 31.30, 30.80, 14.21; HRMS (ESI) calcd for $[C_{16}H_{17}N_3NaO_2]^+$($[M+Na]^+$): 306.1213, found: 306.1212.

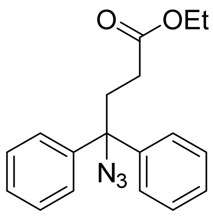

54

Following the **general procedure A**, the mixture of vinylarene (0.5 mmol), alkyl iodide (1.0 mmol), $TMSN_3$ (1.0 mmol) and TBPB (1.0 mmol) in DME (2 mL) was added to $Fe(OTf)_2$ (0.025 mmol) at -20 °C to room temperature for 2 hours to afford **54**. Yield: 104 mg, 67%; clear oil; IR (KBr): υ 2981, 2102, 1734, 1446, 1252, 1181, 1095, 700 cm^{-1}; ^1H NMR (400 MHz, $CDCl_3$) δ 7.37 – 7.29 (m, 8H), 7.29 – 7.22 (m, 2H), 4.07 (q, J= 7.1 Hz, 2H), 2.80 – 2.69 (m, 2H), 2.28 – 2.16 (m, 2H), 1.21 (t, J = 7.1 Hz, 3H); ^{13}C NMR (100 MHz, $CDCl_3$) δ 173.10, 142.28, 128.49, 127.75, 127.08, 71.96, 60.58, 33.89, 29.50, 14.20; HRMS (ESI) calcd for $[C_{18}H_{19}N_3NaO_2]^+$($[M+Na]^+$): 332.1369, found: 332.1371.

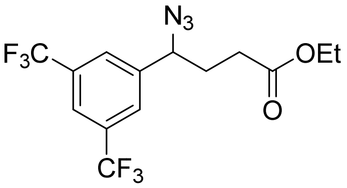

55

Following the **general procedure A**, the mixture of vinylarene (0.5 mmol), alkyl iodide (1.0 mmol), $TMSN_3$ (1.0 mmol) and TBPB (1.0 mmol) in DME (2 mL) was added to $Fe(OTf)_2$ (0.025 mmol) at 50 °C for 10 minutes to afford **55**. Yield: 157 mg, 85%; clear oil; IR (KBr): υ 2986, 2104, 1735, 1448, 1380, 1280, 1136, 707 cm^{-1}; ^1H NMR (400 MHz, $CDCl_3$) δ 7.87 (s, 1H), 7.80 (s, 2H), 4.77 (t, J = 7.0 Hz, 1H), 4.16 (q, J = 7.1 Hz, 2H), 2.54 – 2.35 (m, 2H), 2.16 – 2.05 (m, 2H), 1.27 (t, J = 7.1 Hz, 3H); ^{19}F NMR (376 MHz, $CDCl_3$) δ -63.01 (s, 6F); ^{13}C NMR (100 MHz, $CDCl_3$) δ 172.27, 142.35, 132.31 (q, J = 33.5 Hz, C-CF_3), 126.99 – 126.85 (m), 123.06 (q, J = 272.8 Hz, $C$$F_3$), 122.35 (hept, J = 3.7 Hz), 64.24, 60.80, 31.78, 30.25, 14.07; HRMS (ESI) calcd for $[C_{14}H_{13}F_6N_3NaO_2]^+$($[M+Na]^+$): 392.0804, found: 392.0805.

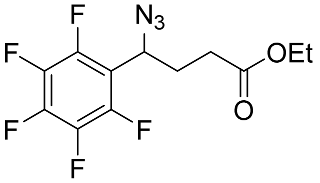

56

Following the **general procedure A**, the mixture of vinylarene (0.5 mmol), alkyl iodide (1.0 mmol), $TMSN_3$ (1.0 mmol) and TBPB (1.0 mmol) in DME (2 mL) was added to $Fe(OTf)_2$

(0.025 mmol) at 50 °C for 10 minutes to afford **56**. Yield: 149 mg, 92%; clear oil; IR (KBr): υ 2932, 2103, 1737, 1652, 1504, 1256, 1126, 803 cm^{-1}; ^{1}H NMR (400 MHz, $CDCl_3$) δ 5.01 – 4.93 (m, 1H), 4.15 (q, *J*= 7.1 Hz, 2H), 2.47 – 2.40 (m, 2H), 2.40 – 2.31 (m, 1H), 2.21 – 2.10 (m, 1H), 1.27 (t, *J* = 7.2 Hz, 3H); ^{19}F NMR (376 MHz, $CDCl_3$) δ -140.07 – -142.72 (m, 2F), --151.80 – -153.91 (m, 1F), -159.27 – -162.11 (m, 2F); ^{13}C NMR (100 MHz, $CDCl_3$) δ 171.93, 145.04 (dm, *J* = 250.4 Hz), 141.24 (dm, *J* = 256.0 Hz), 137.72 (dm, *J* = 254.4 Hz), 112.49 (td, J = 15.6, 4.1 Hz), 60.87, 55.21, 30.69, 28.50, 14.10; HRMS (ESI) calcd for $[C_{12}H_{10}F_5N_3NaO_2]^+$($[M+Na]^+$): 346.0585, found: 346.0584.

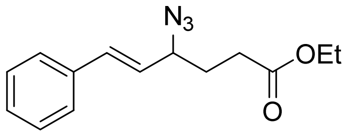

57

Following the **general procedure A**, the mixture of diene (0.5 mmol), alkyl iodide (1.0 mmol), $TMSN_3$ (1.0 mmol) and TBPB (1.0 mmol) in DME (2 mL) was added to $Fe(OTf)_2$ (0.025 mmol) at 50 °C for 10 minutes to afford **57**. Yield: 99 mg, 77%; yellow oil; IR (KBr): υ 2981, 2099, 1733, 1449, 1241, 1179, 968, 751 cm^{-1}; ^{1}H NMR (400 MHz, $CDCl_3$) δ 7.42 – 7.38 (m, 2H), 7.37 – 7.24 (m, 3H), 6.63 (d, *J* = 15.8 Hz, 1H), 6.09 (dd, *J* = 15.8, 8.1 Hz, 1H), 4.16 – 4.07 (m, 3H), 2.42 (t, *J* = 7.3 Hz, 2H), 1.99 – 1.89 (m, 2H), 1.24 (t, *J* = 7.1 Hz, 3H); ^{13}C NMR (100 MHz, $CDCl_3$) δ 172.79, 135.78, 133.93, 128.68, 128.29, 126.70, 126.24, 63.98, 60.59, 30.51, 29.84, 14.21; HRMS (ESI) calcd for $[C_{14}H_{17}N_3NaO_2]^+$($[M+Na]^+$): 282.1213, found: 282.1213.

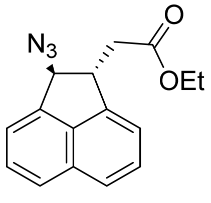

58
trans isomer

Following the **general procedure A**, the mixture of vinylarene (0.5 mmol), alkyl iodide (1.0 mmol), $TMSN_3$ (1.0 mmol) and TBPB (1.0 mmol) in DME (2 mL) was added to $Fe(OTf)_2$ (0.025 mmol) at room temperature for 30 minutes to afford **58** (*trans* and *cis* isomers). *trans isomer*: Yield: 67 mg, 48%; yellow oil; IR (KBr): υ 2981, 2095, 1733, 1496, 1371, 1252, 1172, 1029, 778 cm^{-1}; ^{1}H NMR (400 MHz, $CDCl_3$) δ 7.79 (d, *J* = 8.0 Hz, 1H), 7.71 (d, *J* = 8.1 Hz, 1H), 7.62 – 7.48 (m, 3H), 7.31 (d, *J* = 6.9 Hz, 1H), 5.13 (s, 1H), 4.20 (q, *J* = 7.0 Hz, 2H), 4.03 (t, *J* = 6.5 Hz, 1H), 2.95 – 2.67 (m, 2H), 1.25 (t, *J* = 7.0 Hz, 3H); ^{13}C NMR (100

MHz, $CDCl_3$) δ 171.52, 143.31, 139.79, 136.73, 131.30, 128.39, 128.19, 125.51, 123.85, 120.90, 119.69, 69.31, 60.92, 48.32, 39.29, 14.19; HRMS (ESI) calcd for $[C_{16}H_{15}N_3NaO_2]^+$($[M+Na]^+$): 304.1056, found: 304.1056.

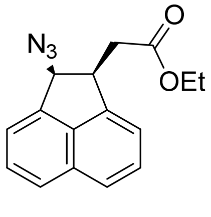

58
cis isomer

cis isomer: Yield: 20 mg, 14%; yellow oil; IR (KBr): υ 3051, 2982, 2926, 2098, 1732, 1463, 1372, 1265, 1176, 1029, 782 cm^{-1}; ^1H NMR (400 MHz, $CDCl_3$) δ 7.80 (d, J = 7.8 Hz, 1H), 7.70 (d, J = 7.7 Hz, 1H), 7.62 – 7.46 (m, 3H), 7.28 – 7.23 (m, 1H), 5.52 (d, J = 7.0 Hz, 1H), 4.34 – 4.24 (m, 3H), 2.95 (d, J = 7.4 Hz, 2H), 1.34 (t, J = 7.1 Hz, 3H); ^{13}C NMR (100 MHz, $CDCl_3$) δ 172.55, 143.75, 140.27, 136.66, 131.32, 128.33, 128.08, 125.60, 123.72, 120.82, 119.29, 65.46, 60.87, 44.21, 34.75, 14.31 (**Note**: trace amount of trans isomer's peaks were found in ^1H and ^{13}C NMR spectra); HRMS (ESI) calcd for $[C_{16}H_{15}N_3NaO_2]^+$($[M+Na]^+$): 304.1056, found: 304.1052.

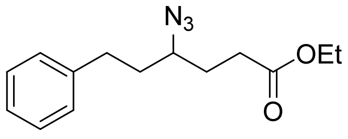

59

The mixture of alkene (0.5 mmol), alkyl iodide (1.0 mmol), and TBPB (0.375 mmol) in DME (2 mL) was added to $Fe(OTf)_2$ (0.05 mmol) at 50 °C for 10 minutes. Then the mixture of $TMSN_3$ (1.5 mmol) and TBPB (1.5 mmol) in DME (1 mL) was added and stirred for further 20 minutes at the same temperature to give the compound **59**. Yield: 85 mg, 65%; clear oil; IR (KBr): υ 2935, 2100, 1735, 1454, 1251, 1181, 1030, 700 cm^{-1}; ^1H NMR (400 MHz, $CDCl_3$) δ 7.33 – 7.25 (m, 2H), 7.23 – 7.15 (m, 3H), 4.13 (q, J = 7.1 Hz, 2H), 3.37 – 3.26 (m, 1H), 2.84 – 2.74 (m, 1H), 2.74 – 2.63 (m, 1H), 2.50 – 2.33 (m, 2H), 1.98 – 1.73 (m, 4H), 1.24 (t, J = 7.1 Hz, 3H); ^{13}C NMR (100 MHz, $CDCl_3$) δ 172.87, 140.97, 128.57, 128.41, 126.17, 61.45, 60.60, 36.14, 32.29, 30.83, 29.63, 14.22; HRMS (ESI) calcd for $[C_{14}H_{19}N_3NaO_2]^+$($[M+Na]^+$): 284.1369, found: 284.1371.

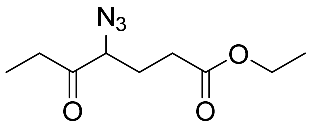

60

Following the **general procedure A**, the mixture of alkene (0.5 mmol), alkyl iodide (1.0 mmol), $TMSN_3$ (1.0 mmol) and TBPB (1.0 mmol) in DME (2 mL) was added to $Fe(OTf)_2$ (0.025 mmol) at 50 °C for 10 minutes to afford **60**. Yield: 72 mg, 67%; clear oil; IR (KBr): υ 2982, 2106, 1730, 1448, 1260, 1179, 1030, 699 cm^{-1}; ^{1}H NMR (400 MHz, $CDCl_3$) δ 4.15 (q, *J* = 7.1 Hz, 2H), 3.96 (dd, *J* = 9.0, 4.6 Hz, 1H), 2.66 – 2.53 (m, 2H), 2.51 – 2.41 (m, 2H), 2.22 – 2.10 (m, 1H), 1.99 – 1.86 (m, 1H), 1.27 (t, *J* = 7.1 Hz, 3H), 1.10 (t, *J* = 7.2 Hz, 3H); ^{13}C NMR (100 MHz, $CDCl_3$) δ 207.37, 172.35, 66.84, 60.75, 33.01, 30.09, 25.85, 14.15, 7.36; HRMS (ESI) calcd for $[C_9H_{15}N_3NaO_3]^+$($[M+Na]^+$): 236.1006, found: 236.1002.

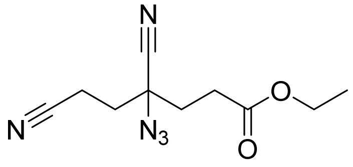

61

Following the **general procedure A**, the mixture of alkene (0.5 mmol), alkyl iodide (1.0 mmol), $TMSN_3$ (1.0 mmol) and TBPB (1.0 mmol) in DME (2 mL) was added to $Fe(OTf)_2$ (0.025 mmol) at 50 °C for 10 minutes to afford **61**. Yield: 94 mg, 80%; clear oil; IR (KBr): υ 2984, 2252, 2107, 1732, 1447, 895 cm^{-1}; ^{1}H NMR (400 MHz, $CDCl_3$) δ 4.18 (q, *J* = 7.1 Hz, 2H), 2.68 – 2.54 (m, 4H), 2.32 – 2.07 (m, 4H), 1.29 (t, *J* = 7.1 Hz, 3H); ^{13}C NMR (100 MHz, $CDCl_3$) δ 171.01, 117.55, 115.80, 61.36, 61.30, 33.60, 33.03, 29.10, 14.12, 12.96; HRMS (ESI) calcd for $[C_{10}H_{13}N_5NaO_2]^+$($[M+Na]^+$): 258.0961, found: 258.0962.

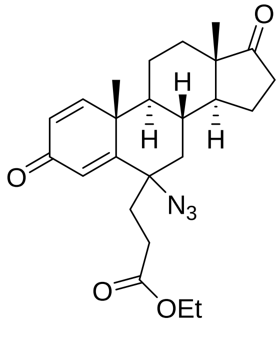

62

Following the **general procedure A**, the mixture of alkene (0.5 mmol), alkyl iodide (1.0 mmol), $TMSN_3$ (1.0 mmol) and TBPB (1.0 mmol) in DME (2 mL) was added to $Fe(OTf)_2$ (0.025 mmol) at 50 °C for 10 minutes to afford **62**. Yield: 152 mg, 72% (dr = 2:1); white solid; mp 77-78 °C; IR (KBr): υ 3054, 2946, 2101, 1732, 1665, 1625, 1455, 1244, 1182, 1088, 735 cm^{-1}; ^{1}H NMR (400 MHz, $CDCl_3$) δ 7.11 (dd, *J* = 10.2, 1.4 Hz, 2H), 7.07 (dd, *J* = 10.1, 1.4 Hz, 1H), 6.50 – 6.47 (m, 1H), 6.40 – 6.36 (m, 2H), 6.30-6.22 (m, 3H), 4.22 – 4.09 (m, 6H), 2.59 – 2.33 (m, 12H), 2.26 – 1.99 (m, 12H), 2.01 – 1.86 (m, 10H), 1.84 – 1.54 (m, 8H),

1.40 – 1.11 (m, 25H), 1.09 – 0.81 (m, 13H); ^{13}C NMR (100 MHz, $CDCl_3$) δ 219.32, 219.00, 185.54, 185.32, 172.77, 172.44, 161.52, 160.97, 156.44, 155.17, 127.52, 126.71, 126.53, 126.24, 66.88, 65.01, 60.94, 60.80, 50.30, 50.21, 50.17, 49.78, 47.51, 47.50, 43.60, 43.48, 41.36, 39.85, 35.55, 32.92, 31.87, 31.07, 31.00, 30.75, 29.15, 29.00, 21.81, 21.75, 21.48, 21.32, 21.21, 20.32, 14.22, 14.16, 13.88, 13.81; HRMS (ESI) calcd for $[C_{24}H_{31}N_3NaO_4]^+$($[M+Na]^+$): 448.2207, found: 448.2207.

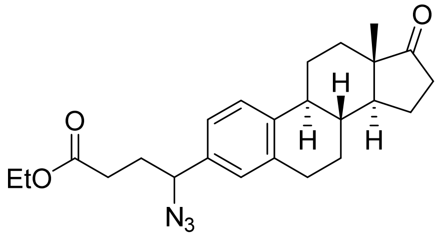

63

Following the **general procedure A**, the mixture of vinylarene (0.5 mmol), alkyl iodide (1.0 mmol), $TMSN_3$ (1.0 mmol) and TBPB (1.0 mmol) in DME (2 mL) was added to $Fe(OTf)_2$ (0.025 mmol) at 50 °C for 10 minutes to afford **63**. Yield: 156 mg, 76% (dr = 1:1); colorless semi-solid; IR (KBr): υ 2930, 2098, 1737, 1454, 1374, 1255, 1178, 1083, 823 cm^{-1}; ^{1}H NMR (400 MHz, $CDCl_3$)) δ 7.30 (d, *J* = 8.0 Hz, 1H), 7.08 (d, *J* = 8.0 Hz, 1H), 7.03 (s, 1H), 4.46 (t, *J* = 7.0 Hz, 1H), 4.13 (q, *J* = 7.1 Hz, 2H), 2.99 – 2.85 (m, 2H), 2.57 – 2.46 (m, 1H), 2.46 – 2.25 (m, 4H), 2.22 – 1.93 (m, 6H), 1.68 – 1.41 (m, 6H), 1.25 (t, *J* = 7.0 Hz, 3H), 0.92 (s, 3H); ^{13}C NMR (100 MHz, $CDCl_3$) δ 220.75, 172.78, 140.03, 137.08, 136.43, 127.43, 127.39, 125.85, 124.24, 124.19, 65.17, 65.11, 60.55, 50.52, 47.97, 44.38, 38.03, 35.85, 31.59, 31.31, 31.27, 30.91, 29.44, 29.43, 26.43, 25.66, 21.60, 14.23, 13.85. HRMS (ESI) calcd for $[C_{24}H_{31}N_3NaO_3]^+$($[M+Na]^+$): 432.2258, found: 432.2260.

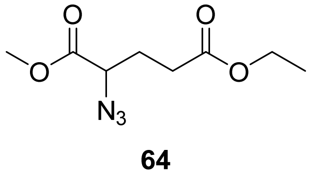

64

Following the **general procedure A**, the mixture of alkene (0.5 mmol), alkyl iodide (1.0 mmol), $TMSN_3$ (1.0 mmol) and TBPB (1.0 mmol) in DME (2 mL) was added to $Fe(OTf)_2$ (0.025 mmol) at 50 °C for 10 minutes to afford **64**. Yield: 70 mg, 65%; clear oil; IR (KBr): υ 2984, 2109, 1739, 1439, 1030, 859 cm^{-1}; ^{1}H NMR (400 MHz, $CDCl_3$) δ 4.15 (q, *J* = 7.1 Hz, 2H), 4.02 (dd, *J* = 8.4, 5.2 Hz, 1H), 3.81 (s, 3H), 2.46 (t, *J* = 7.3 Hz, 2H), 2.26 – 2.13 (m, 1H), 2.09 – 1.95 (m, 1H), 1.27 (t, *J* = 7.0 Hz, 3H); ^{13}C NMR (100 MHz, $CDCl_3$) δ 172.21, 170.47,

61.06, 60.72, 52.71, 30.07, 26.51, 14.16; HRMS (ESI) calcd for $[C_8H_{13}N_3NaO_4]^+$($[M+Na]^+$): 238.0798, found: 238.0799.

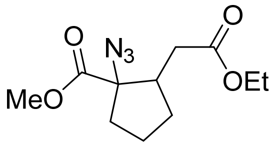

65

Following the **general procedure A**, the mixture of alkene (0.5 mmol), alkyl iodide (1.0 mmol), $TMSN_3$ (1.0 mmol) and TBPB (1.0 mmol) in DME (2 mL) was added to $Fe(OTf)_2$ (0.025 mmol) at 50 °C for 10 minutes to afford **65**. **Major isomer**: Yield: 53 mg, 41%; clear oil; IR (KBr): υ 2956, 2117, 1738, 1436, 1261, 1193, 1028, 802 cm^{-1}; ^1H NMR (400 MHz, $CDCl_3$) δ 4.13 (q, *J* = 7.2, Hz, 2H), 3.80 (s, 3H), 2.61 – 2.53 (m, 1H), 2.47 (dd, *J* = 15.7, 5.2 Hz, 1H), 2.37 – 2.06 (m, 3H), 1.99 – 1.75 (m, 3H), 1.57 – 1.48 (m, 1H), 1.26 (t, *J* = 7.1 Hz, 3H); ^{13}C NMR (100 MHz, $CDCl_3$) δ 171.85, 171.76, 75.17, 60.67, 52.63, 45.70, 35.91, 35.33, 30.67, 22.23, 14.19; HRMS (ESI) calcd for $[C_{11}H_{17}N_3NaO_4]^+$($[M+Na]^+$): 278.1111, found: 278.1111. **Minor isomer**: Yield: 6 mg, 5%; clear oil; IR (KBr): υ 2957, 2108, 1737, 1436, 1255, 1195, 1030, 800 cm^{-1}; ^1H NMR (400 MHz, $CDCl_3$) δ 4.10 (q, *J* = 7.1 Hz, 2H), 3.80 (s, 3H), 2.78 – 2.70 (m, 1H), 2.53 – 2.44 (m, 1H), 2.36 – 2.28 (m, 1H), 2.28 – 1.99 (m, 3H), 1.88 – 1.80 (m, 2H), 1.52 – 1.42 (m, 1H), 1.24 (t, *J* = 7.1 Hz, 3H); ^{13}C NMR (100 MHz, $CDCl_3$) δ 172.53, 172.34, 74.87, 60.45, 52.85, 44.24, 35.95, 34.16, 30.10, 21.86, 14.18; HRMS (ESI) calcd for $[C_{11}H_{17}N_3NaO_4]^+$($[M+Na]^+$): 278.1111, found: 278.1111.

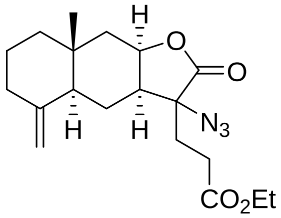

66

Following the **general procedure A**, the mixture of alkene (0.5 mmol), alkyl iodide (1.0 mmol), $TMSN_3$ (1.0 mmol) and TBPB (1.0 mmol) in DME (2 mL) was added to $Fe(OTf)_2$ (0.025 mmol) at 50 °C for 10 minutes to afford **66**. Yield: 116 mg, 64% (single isomer); clear oil; IR (KBr): υ 2932, 2103, 1771, 1735, 1444, 1221, 1173, 982 cm^{-1}; ^1H NMR (400 MHz, $CDCl_3$) δ 4.83 – 4.75 (m, 2H), 4.46 (s, 1H), 4.22 – 4.13 (m, 2H), 2.66 – 2.56 (m, 1H), 2.57 – 2.28 (m, 3H), 2.21 (dd, *J* = 15.7, 2.1 Hz, 1H), 2.16 – 2.04 (m, 2H), 2.04 – 1.93 (m, 1H), 1.79 (d, *J* = 12.3Hz, 1H), 1.66 – 1.51 (m, 4H), 1.46 (dd, *J* = 15.7, 4.4 Hz, 1H), 1.29 (t, *J* = 7.2 Hz, 3H), 1.25 – 1.15 (m, 2H), 0.79 (s, 3H); ^{13}C NMR (100 MHz, $CDCl_3$) δ 172.60, 172.05,

148.75, 106.73, 77.29, 70.55, 60.98, 46.47, 43.43, 42.04, 41.11, 36.66, 34.49, 28.82, 23.84, 22.62, 20.87, 17.80, 14.17; HRMS (ESI) calcd for $[C_{19}H_{27}N_3NaO_4]^+$($[M+Na]^+$): 384.1894, found: 384.1895.

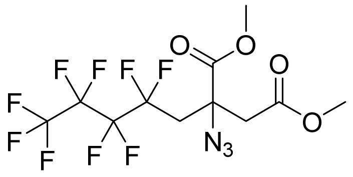

67

Following the **general procedure A**, the mixture of alkene (0.5 mmol), alkyl iodide (1.0 mmol), $TMSN_3$ (1.0 mmol) and TBPB (1.0 mmol) in DME (2 mL) was added to $Fe(OTf)_2$ (0.025 mmol) at room temperature for 3 minutes to afford **67**. Yield: 172 mg, 82%: clear oil; IR (KBr): υ 2961, 2123, 1746, 1440, 1222, 1134, 739 cm^{-1}; ^1H NMR (400 MHz, $CDCl_3$) δ 3.88 (s, 3H), 3.73 (s, 3H), 3.10 – 2.94 (m, 2H), 2.94 – 2.70 (m, 2H); ^{19}F NMR (376 MHz, $CDCl_3$) δ -79.94 – -82.26 (m, 3F), -109.70 – -111.95 (m, 1F), -112.33 – -115.42 (m, 1F), -124.04 – -124.97 (m, 2F), -125.32 – -126.46 (m, 2F); ^{13}C NMR (100 MHz, $CDCl_3$) δ 169.41, 169.14, 122.79 – 103.47 (m, $(CF_2)_3CF_3$), 62.77, 53.61, 52.24, 40.19, 34.99 (t, J = 20.0 Hz, CH_2-CF_2); HRMS (ESI) calcd for $[C_{11}H_{10}F_9N_3NaO_4]^+$($[M+Na]^+$): 442.0420, found: 442.0421.

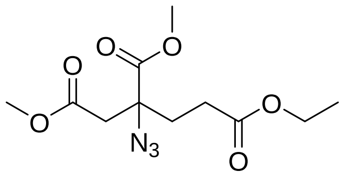

68

Following the **general procedure A**, the mixture of alkene (0.5 mmol), alkyl iodide (1.0 mmol), $TMSN_3$ (1.0 mmol) and TBPB (1.0 mmol) in DME (2 mL) was added to $Fe(OTf)_2$ (0.025 mmol) at 50 °C for 10 minutes to afford **68**. Yield: 123 mg, 85%; clear oil; IR (KBr): υ 2957, 2116, 1739, 1439, 1202, 1076, 699 cm^{-1}; ^1H NMR (400 MHz, $CDCl_3$) δ 4.14 (q, J = 7.1 Hz, 2H), 3.83 (s, 3H), 3.71 (s, 3H), 3.01 (d, J = 16.5 Hz, 1H), 2.73 (d, J = 16.5 Hz, 1H), 2.54 – 2.28 (m, 2H), 2.22 – 2.00 (m, 2H), 1.26 (t, J = 7.1 Hz, 3H); ^{13}C NMR (100 MHz, $CDCl_3$) δ 172.08, 170.87, 169.70, 66.07, 60.75, 53.05, 52.11, 41.53, 32.35, 28.80, 14.10; HRMS (ESI) calcd for $[C_{11}H_{17}N_3NaO_6]^+$($[M+Na]^+$): 310.1010, found: 310.1010.

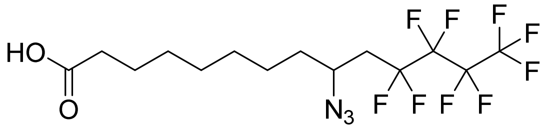

69

Following the **general procedure A**, the mixture of alkene (0.5 mmol), alkyl iodide (0.65 mmol), $TMSN_3$ (1.0 mmol) and TBPB (1.0 mmol) in DME (2 mL) was added to $Fe(OTf)_2$ (0.025 mmol) at room temperature for 5 minutes to afford **69**. Yield: 190 mg, 88%; clear oil; IR (KBr): υ 2936, 2111, 1712, 1414, 1233, 1134, 880 cm^{-1}; ^1H NMR (400 MHz, $CDCl_3$) δ 3.79 – 3.69 (m, 1H), 2.43 – 2.12 (m, 4H), 1.72 – 1.56 (m, 4H), 1.55 – 1.26 (m, 8H); ^{19}F NMR (376 MHz, $CDCl_3$) δ -79.15 – -83.38 (m, 3F), -112.11 – -115.03 (m, 2F), -123.76 – -125.28 (m, 2F), -125.38 – -126.68 (m, 2F); ^{13}C NMR (100 MHz, $CDCl_3$) δ 180.41, 123.00 – 103.47 (m, $(CF_2)_3CF_3$), 55.85, 35.47 (t, J = 21.3 Hz, CH_2-CF_2), 35.33, 33.99, 28.96, 28.87, 28.82, 25.56, 24.51; HRMS (ESI) calcd for $[C_{14}H_{18}F_9N_3NaO_2]^+$($[M+Na]^+$): 454.1148, found: 454.1148.

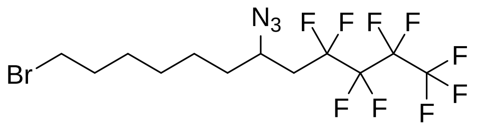

70

Following the **general procedure A**, the mixture of alkene (0.5 mmol), alkyl iodide (0.65 mmol), $TMSN_3$ (1.0 mmol) and TBPB (1.0 mmol) in DME (2 mL) was added to $Fe(OTf)_2$ (0.025 mmol) at room temperature for 5 minutes to afford **70**. Yield: 194 mg, 86%; clear oil; IR (KBr): υ 2938, 2862, 2111, 1356, 1234, 1134, 880, 722 cm^{-1}; ^1H NMR (400 MHz, $CDCl_3$) δ 3.79 – 3.70 (m, 1H), 3.42 (t, J = 6.7 Hz, 2H), 2.39 – 2.14 (m, 2H), 1.93 – 1.82 (m, 2H), 1.68 – 1.58 (m, 2H), 1.56 – 1.32 (m, 6H); ^{19}F NMR (376 MHz, $CDCl_3$) δ -75.63 – -86.30 (m, 3F), -110.49 – -116.56 (m, 2F), -123.08 – -125.19 (m, 2F), -125.25 – -127.53 (m, 2F); ^{13}C NMR (100 MHz, $CDCl_3$) δ 125.18 – 102.63 (m, $(CF_2)_3CF_3$), 55.84, 35.52 (t, J = 21.3 Hz), 35.29, 33.66, 32.54, 28.28, 27.91, 25.53. HRMS (EI) calcd for $[C_{12}H_{14}BrF_9N]^+$($[M-N_2-H]^+$): 422.0166, found: 422.0168.

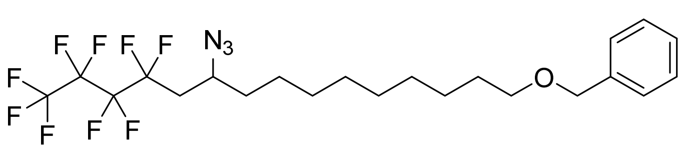

71

Following the **general procedure A**, the mixture of alkene (0.5 mmol), alkyl iodide (0.65 mmol), $TMSN_3$ (1.0 mmol) and TBPB (1.0 mmol) in DME (2 mL) was added to $Fe(OTf)_2$ (0.025 mmol) at room temperature for 5 minutes to afford **71**. Yield: 175 mg, 67%; clear oil; IR (KBr): υ 2932, 2858, 2110, 1357, 1235, 1134, 880, 736 cm^{-1}; ^1H NMR (400 MHz, $CDCl_3$) δ 7.29 – 7.23 (m, 4H), 7.22 – 7.16 (m, 1H), 4.42 (s, 2H), 3.70 – 3.60 (m, 1H), 3.39 (t, J = 6.6 Hz, 2H), 2.29 – 2.04 (m, 2H), 1.59 – 1.47 (m, 4H), 1.42 – 1.20 (m, 12H); ^{19}F NMR (376

MHz, $CDCl_3$) δ -78.80 – -83.55 (m, 3F), -111.86 – -115.26 (m, 2F), -122.67 – -125.15 (m, 2F), -125.34 – -127.75 (m, 2F); ^{13}C NMR (100 MHz, $CDCl_3$) δ 138.74, 128.34, 127.62, 127.47, 120.83 – 105.65 (m, $(CF_2)_3CF_3$), 72.88, 70.50, 55.89, 35.51 (t, *J* = 21.2 Hz, CH_2CF_2), 35.41, 29.78, 29.43, 29.41, 29.35, 29.12, 26.18, 25.68; HRMS (ESI) calcd for $[C_{22}H_{28}F_9N_3NaO]^+$($[M+Na]^+$): 544.1990, found: 544.1986.

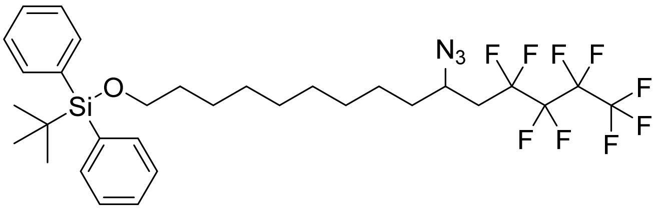

72

Following the **general procedure A**, the mixture of alkene (0.5 mmol), alkyl iodide (0.65 mmol), $TMSN_3$ (1.0 mmol) and TBPB (1.0 mmol) in DME (2 mL) was added to $Fe(OTf)_2$ (0.025 mmol) at room temperature for 5 minutes to afford **72**. Yield: 275 mg, 82%; clear oil; IR (KBr): υ 2932, 2858, 2109, 1356, 1236, 1111, 702 cm^{-1}; ^1H NMR (400 MHz, $CDCl_3$) δ 7.72 – 7.63 (m, 4H), 7.45 – 7.33 (m, 6H), 3.78 – 3.69 (m, 1H), 3.66 (t, *J* = 6.5 Hz, 2H), 2.38 – 2.12 (m, 2H), 1.66 – 1.51 (m, 4H), 1.44 – 1.20 (m, 12H), 1.05 (s, 9H); ^{19}F NMR (376 MHz, $CDCl_3$) δ -78.20 – -83.76 (m, 3F), -111.47 – -115.46 (m, 2F), -122.97 – -125.09 (m, 2F), -125.21 – -127.20 (m, 2F); ^{13}C NMR (100 MHz, $CDCl_3$) δ 135.62, 134.23, 129.51, 127.59, 120.83 – 105.02 (m, $(CH_2)_3CH_3$), 64.01, 55.91, 35.54 (t, *J* = 21.3 Hz, CH_2CF_2), 35.44, 32.59, 29.47, 29.37, 29.32, 29.15, 26.89, 25.77, 25.72, 19.25; HRMS (EI) calcd for $[C_{27}H_{31}F_9NOSi]^+$($[M-N_2-C_4H_9]^+$): 584.2031, found: 584.2028.

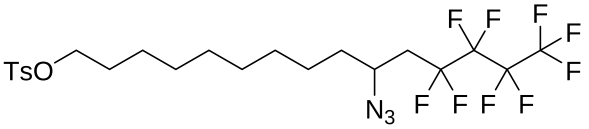

73

Following the **general procedure A**, the mixture of alkene (0.5 mmol), alkyl iodide (0.65 mmol), $TMSN_3$ (1.0 mmol) and TBPB (1.0 mmol) in DME (2 mL) was added to $Fe(OTf)_2$ (0.025 mmol) at room temperature for 5 minutes to afford **73**. Yield: 234 mg, 80%; clear oil; IR (KBr): υ 2931, 2859, 2111, 1360, 1236, 1134, 664 cm^{-1}; ^1H NMR (400 MHz, $CDCl_3$) δ 7.79 (d, *J* = 8.2 Hz, 2H), 7.35 (d, *J* = 8.1 Hz, 2H), 4.02 (t, *J* = 6.5 Hz, 2H), 3.79 – 3.68 (m, 1H), 2.45 (s, 3H), 2.37 – 2.13 (m, 2H), 1.67 – 1.58 (m, 4H), 1.52 – 1.36 (m, 2H), 1.33 – 1.21 (m, 10H); ^{19}F NMR (376 MHz, $CDCl_3$) δ -80.13 – -81.97 (m, 3F), -112.97 – -114.16 (m, 2F), -123.89 – -125.17 (m, 2F), -125.42 – -126.80 (m, 2F); ^{13}C NMR (100 MHz, $CDCl_3$) δ 144.65, 133.24, 129.80, 127.87, 122.37 – 107.20 (m, $(CF_2)_3CF_3$), 70.66, 55.87, 35.49 (t, *J* = 21.2 Hz,

CH_2CF_2), 35.37, 29.22, 29.20, 29.04, 28.84, 28.80, 25.64, 25.30, 21.58; HRMS (ESI) calcd for $[C_{22}H_{28}F_9N_3NaO_3S]^+$($[M+Na]^+$): 608.1602, found: 608.1601.

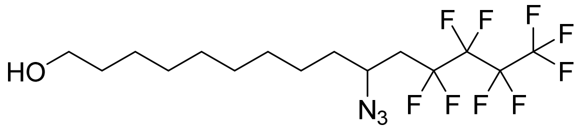

74

Following the **general procedure A**, the mixture of alkene (0.5 mmol), alkyl iodide (0.65 mmol), $TMSN_3$ (1.0 mmol) and TBPB (1.0 mmol) in DME (2 mL) was added to $Fe(OTf)_2$ (0.025 mmol) at room temperature for 5 minutes to afford **74**. Yield: 132 mg, 61%; clear oil; IR (KBr): υ 3351, 2931, 2858, 2110, 1465, 1355, 1234, 736 cm^{-1}; ^1H NMR (400 MHz, $CDCl_3$) δ 3.79 – 3.70 (m, 1H), 3.64 (t, J = 6.6 Hz, 2H), 2.39 – 2.13 (m, 2H), 1.66 – 1.29 (m, 16H); ^{19}F NMR (376 MHz, $CDCl_3$) δ -80.43 – -81.89 (m, 3F), -112.89 – -114.30 (m, 2F), -123.88 – -125.11 (m, 2F), -125.42 – -126.67 (m, 2F); ^{13}C NMR (100 MHz, $CDCl_3$) δ 123.00 – 103.26 (m, ($CF_2)_3CF_3$) 62.96, 55.87, 35.48 (t, J = 21.3 Hz, CH_2-CF_2), 35.37, 32.74, 29.41, 29.33, 29.31, 29.08, 25.69, 25.65; HRMS (ESI) calcd for $[C_{15}H_{22}F_9N_3NaO]^+$($[M+Na]^+$): 454.1511, found: 454.1513.

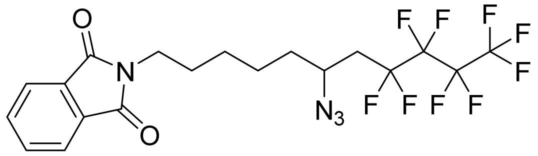

75

Following the **general procedure A**, the mixture of alkene (0.5 mmol), alkyl iodide (0.65 mmol), $TMSN_3$ (1.0 mmol) and TBPB (1.0 mmol) in DME (2 mL) was added to $Fe(OTf)_2$ (0.025 mmol) at room temperature for 5 minutes to afford **75**. Yield: 212 mg, 84%; yellow oil; IR (KBr): υ 2941, 2111, 1715, 1234, 1134, 880, 721 cm^{-1}; ^1H NMR (400 MHz, $CDCl_3$) δ 7.85 (dd, J = 5.4, 3.0 Hz, 2H), 7.72 (dd, J = 5.5, 3.0 Hz, 2H), 3.78 – 3.67 (m, 3H), 2.39 – 2.13 (m, 2H), 1.78 – 1.68 (m, 2H), 1.67 – 1.58 (m, 2H), 1.57 – 1.51 (m, 1H), 1.50 – 1.36 (m, 3H); ^{19}F NMR (376 MHz, $CDCl_3$) δ -79.87 – -82.21 (m, 3F), -112.51 – -114.52 (m, 2F), -123.60 – -125.18 (m, 2F), -125.37 – -126.62 (m, 2F); ^{13}C NMR (100 MHz, $CDCl_3$) δ 168.43, 133.91, 132.11, 123.19, 121.25 – 104.39 (m, ($CF_2)_3CF_3$), 55.77, 37.67, 35.44 (t, J = 21.2 Hz, CH_2CF_2), 35.24, 28.35, 26.36, 25.22; HRMS (ESI) calcd for $[C_{19}H_{17}F_9N_4NaO_2]^+$($[M+Na]^+$): 527.1098, found: 527.1102.

Reduction of azido group to synthesize amine, amino acid and pyrrolidinone

(a) Synthesis of amine

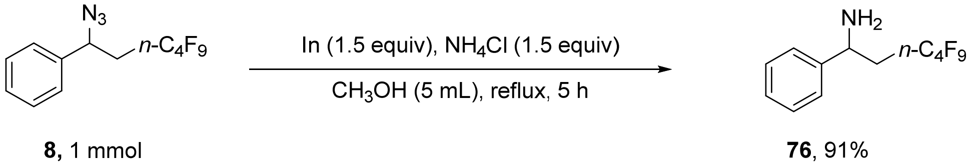

The mixture of azide **8** (1.0 mmol), NH_4Cl (1.5 mmol), and indium powder (1.5 mmol) was refluxed in MeOH (5 mL) for 5 hours under N_2 atmosphere. After completion (TLC), the solvent was evaporated and the residue was purified by flash chromatography on neutral aluminium oxide using petroleum ether and ethyl acetate to give the corresponding product **76** (yield: 309 mg, 91%).[2]

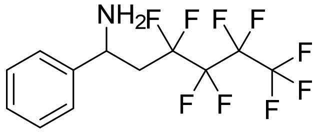

76

Clear oil; IR (KBr): υ 3034, 1355, 1235, 1134, 880, 764, 701 cm^{-1}; ^{1}H NMR (400 MHz, $CDCl_3$) δ 7.42 – 7.26 (m, 5H), 4.54 – 4.47 (m, 1H), 2.57 – 2.31 (m, 2H); ^{19}F NMR (376 MHz, $CDCl_3$) δ -79.78 – -82.45 (m, 3F), -111.22 – -115.49 (m, 2F), -123.98 – -125.20 (m, 2F), -125.36 – -126.49 (m, 2F); ^{13}C NMR (100 MHz, $CDCl_3$) δ 144.31, 128.87, 127.85, 126.14, 49.71, 40.10 (t, J = 20.4 Hz); HRMS (ESI) calcd for $[C_{12}H_{11}F_9N]^+$($[M+H]^+$): 340.0742, found: 340.0737.

(b) Synthesis of amino acid

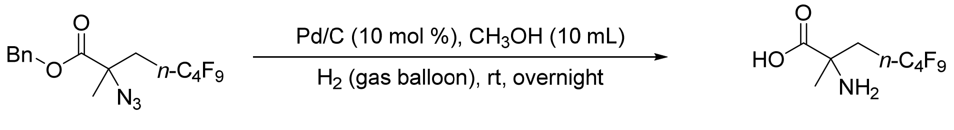

To a solution of the azide **19** (0.50 mmol) in MeOH (10 ml) were added 10% Pd/C (10 mol%). After stirring overnight at room temperature under H_2 atmosphere, the mixture was filtered with a pad of Celite®. Concentration of the solvent in vacuo gave the desired amino acid **77** (yield: 156 mg, 97%).[3]

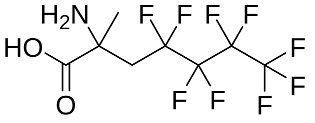

77

White solid; mp 278-279 °C; ^{1}H NMR (400 MHz, CD_3OD) δ 3.24 – 3.05 (m, 1H), 2.93 – 2.73 (m, 1H), 1.73 (s, 3H); ^{19}F NMR (376 MHz, CD_3OD) δ -81.92 – -83.39 (m, 3F), -108.91 – -112.31 (m, 1F), -113.50 – -116.57 (m, 1F), -124.73 – -126.22 (m, 2F), -126.42 – -127.90 (m, 2F); ^{13}C NMR (100 MHz, CD_3OD + CF_3COOH) δ 172.12, 57.16 (d, *J* = 1.7 Hz), 37.56 (t, *J* = 20.2 Hz), 24.66, HRMS (ESI) calcd for $[C_8H_{10}F_9NO_2]^+$($[M+H]^+$): 322.0484, found: 322.0481.

(c) Synthesis of pyrrolidinone

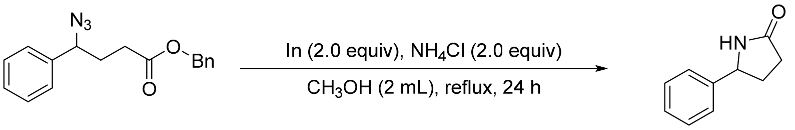

78, 0.5 mmol

79, 95%

The reaction of **78** (0.5 mmol), NH_4Cl (1.0 mmol), indium powder (1.0 mmol) in MeOH (2 mL) at reflux for 24 hours. After completion (TLC), the solvent was evaporated and the residue was purified by flash chromatography on silica gel using dichloromethane and methanol to give the corresponding product **79** (Yield: 77 mg, 95%).

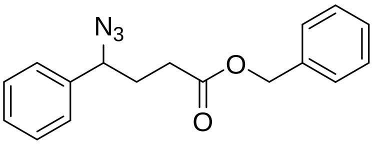

78

Following the **general procedure A**, the mixture of alkene (0.5 mmol), alkyl iodide (1.0 mmol), $TMSN_3$ (1.0 mmol) and TBPB (1.0 mmol) in DME (2 mL) was added to $Fe(OTf)_2$ (0.025 mmol) at room temperature for 15 minutes to afford **78**. Yield: 94 mg, 63%; clear oil; IR (KBr): υ 3033, 2956, 2098, 1733, 1455, 1160, 1003, 751, 700 cm^{-1}; ^{1}H NMR (400 MHz, $CDCl_3$) δ 7.40 – 7.23 (m, 10H), 5.10 (d, *J* = 2.0 Hz, 2H), 4.50 (dd, *J* = 7.9, 6.5 Hz, 1H), 2.42 (dd, *J* = 10.8, 4.6 Hz, 2H), 2.18 – 2.00 (m, 2H); ^{13}C NMR (100 MHz, $CDCl_3$) δ 172.44, 138.84, 135.75, 128.83, 128.53, 128.39, 128.26, 128.23, 126.80, 66.35, 65.17, 31.26, 30.69, HRMS (ESI) calcd for $[C_{17}H_{17}N_3NaO_2]^+$($[M+Na]^+$): 318.1213, found: 318.1213.

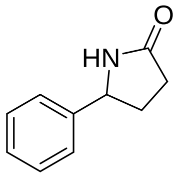

79

Clear oil; ^{1}H NMR (400 MHz, $CDCl_3$) δ 7.42 – 7.23 (m, 5H), 6.70 (s, 1H), 4.75 (t, *J* = 7.1 Hz, 1H), 2.63 – 2.33 (m, 3H), 2.04 – 1.88 (m, 1H); ^{13}C NMR (100 MHz, $CDCl_3$) δ 178.73, 142.45, 128.82, 127.80, 125.55, 58.08, 31.24, 30.27. The NMR data is consistent with the reported value.[4]

Characterization data of carboazidation of alkynes

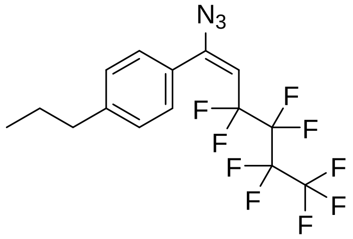

81

To a dried Schlenk tube equipped with a magnetic bar, $Fe(OTf)_3$ (12.7 mg, 0.025 mmol) was added, Then this tube was flushed with nitrogen gas (3 times) and maintained a nitrogen atmosphere using a nitrogen balloon. A thoroughly mixed solution of alkynes (0.5 mmol), R_fI (0.75 mmol), $TMSN_3$ (1.0 mmol) and TBPB (1.0 mmol) in DME (2 mL) was added to the catalyst via syringe and stirred vigorously for 20 minutes at room temperature. After completion (TLC), the solvent was evaporated and the residue was purified by flash chromatography on silica gel using petroleum ether and ethyl acetate to give the corresponding product **81**. Yield: 151 mg, 74%; clear oil; IR (KBr): υ 2965, 2936, 2876, 2118, 1655, 1354, 1235, 1133, 886, 736 cm^{-1}; ^{1}H NMR (400 MHz, $CDCl_3$) δ 7.27 – 7.22 (m, 4H), 5.44 (t, *J* = 14.1 Hz, 1H), 2.66 – 2.59 (m, 2H), 1.72 – 1.61 (m, 2H), 0.94 (t, *J* = 7.3 Hz, 3H). ^{19}F NMR (376 MHz, $CDCl_3$) δ -78.19 – -84.49 (m, 3F), -101.26 – -104.80 (m, 2F), -122.86 – -124.74 (m, 2F), -124.84 – -126.86 (m, 2F). ^{13}C NMR (100 MHz, $CDCl_3$) δ 151.82 (t, *J* = 5.0 Hz), 145.11, 128.55, 127.94 (t, *J* = 2.5 Hz), 101.55 (t, *J* = 22.6 Hz), 37.83, 24.15, 13.70. HRMS (EI) calcd for $[C_{15}H_{12}F_9N]^+$($[M-N_2]^+$): 377.0826, found: 377.0829.

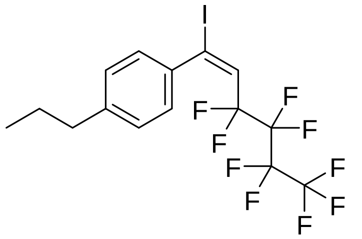

81'

81'; clear oil; IR (KBr): υ 2964, 2934, 2875, 1636, 1505, 1352, 1235, 1134, 874, 748 cm^{-1}; ^{1}H NMR (400 MHz, $CDCl_3$) δ 7.21 (d, *J* = 8.1 Hz, 2H), 7.12 (d, *J* = 8.2 Hz, 2H), 6.56 (t, *J* = 13.5 Hz, 1H), 2.63 – 2.52 (m, 2H), 1.70 – 1.58 (m, 2H), 0.94 (t, *J* = 7.3 Hz, 3H). ^{19}F NMR (376 MHz, $CDCl_3$) δ -79.68 – -82.63 (m, 3F), -104.32 – -106.30 (m, 2F), -122.66 – -124.69 (m, 2F), -125.17 – -127.30 (m, 2F). ^{13}C NMR (100 MHz, $CDCl_3$) δ 144.25, 138.64, 128.01, 126.92 (t, *J* = 2.4 Hz), 126.49 (t, *J* = 21.9 Hz), 113.55 (t, *J* = 6.4 Hz), 37.82, 24.19, 13.76. HRMS (EI) calcd for $[C_{15}H_{12}F_9I]^+$($[M]^+$): 489.9840, found: 489.9838.

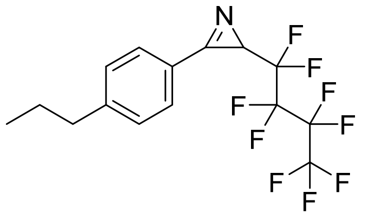

82

Following the **general procedure B**, the mixture of alkyne (0.5 mmol), alkyl iodide (0.75 mmol), $TMSN_3$ (1.0 mmol) and TBPB (1.0 mmol) in DME (2 mL) was added to $Fe(OTf)_3$ (0.025 mmol) at room temperature for 20 minutes and then in toluene at 120°C for 10 minutes to afford **82**. Yield: 114 mg, 60%; clear oil; IR (KBr): υ 2966, 2937, 2877, 1758, 1608, 1355, 1232, 1135, 885, 738 cm^{-1}; ^{1}H NMR (400 MHz, $CDCl_3$) δ 7.84 (d, *J* = 8.2 Hz, 2H), 7.41 (d, *J* = 8.2 Hz, 2H), 2.78 – 2.63 (m, 3H), 1.77 – 1.63 (m, 2H), 0.97 (t, *J* = 7.4 Hz, 3H). ^{19}F NMR (376 MHz, $CDCl_3$) δ -78.94 – -82.75 (m, 3F), -111.48 – -116.29 (m, 2F), -117.59 – -125.28 (m, 6F), -125.68 – -127.94 (m, 2F). ^{13}C NMR (100 MHz, $CDCl_3$) δ 159.87 (d, *J* = 4.0 Hz), 150.30, 130.36, 129.61, 119.60, 38.29, 28.55 (t, *J* = 29.5 Hz), 24.17, 13.65. HRMS (ESI) calcd for $[C_{15}H_{13}F_9N]^+$($[M+H]^+$): 378.0899, found: 378.0895.

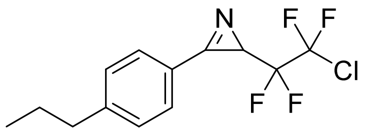

83

Following the **general procedure B**, the mixture of alkyne (0.5 mmol), alkyl iodide (0.75 mmol), $TMSN_3$ (1.0 mmol) and TBPB (1.0 mmol) in DME (2 mL) was added to $Fe(OTf)_3$

(0.025 mmol) at room temperature for 20 minutes and then in toluene at 120°C for 10 minutes to afford **83**. Yield: 92 mg, 62%; clear oil; IR (KBr): υ 2964, 2935, 2875, 1756, 1607, 1419, 1267, 1154, 945, 738 cm^{-1}; ^{1}H NMR (400 MHz, $CDCl_3$) δ 7.84 (d, *J* = 8.2 Hz, 2H), 7.41 (d, *J* = 8.2 Hz, 2H), 2.74 – 2.65 (m, 3H), 1.76 – 1.64 (m, 2H), 0.97 (t, *J* = 7.3 Hz, 3H). ^{19}F NMR (376 MHz, $CDCl_3$) δ -69.09 – -71.42 (m, 2F), -111.20 – -116.27 (m, 2F). ^{13}C NMR (100 MHz, $CDCl_3$) δ 160.11 (d, *J* = 3.9 Hz), 150.19, 130.37, 129.56, 119.64, 38.26, 28.32 (dd, *J* = 31.3, 29.0 Hz), 24.16, 13.69. HRMS (ESI) calcd for $[C_{13}H_{12}ClF_4NNa]^+$($[M+Na]^+$): 316.0487, found: 316.0486.

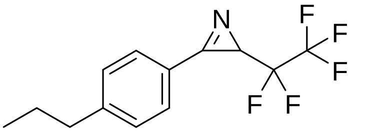

84

Following the **general procedure B**, the mixture of alkyne (0.5 mmol), alkyl iodide (0.75 mmol), $TMSN_3$ (1.0 mmol) and TBPB (1.0 mmol) in DME (2 mL) was added to $Fe(OTf)_3$ (0.025 mmol) at room temperature for 20 minutes and then in toluene at 120°C for 10 minutes to afford **84**. Yield: 82 mg, 59%; clear oil; IR (KBr): υ 2966, 2937, 2876, 1758, 1608, 1336, 1201, 1123, 838, 724 cm^{-1}; ^{1}H NMR (400 MHz, $CDCl_3$) δ 7.83 (d, *J* = 8.1 Hz, 2H), 7.41 (d, *J* = 8.1 Hz, 2H), 2.70 (t, *J* = 7.6 Hz, 2H), 2.62 (t, *J* = 9.2 Hz, 1H), 1.76 – 1.64 (m, 2H), 0.97 (t, *J* = 7.3 Hz, 3H). ^{19}F NMR (376 MHz, $CDCl_3$) δ -83.49 – -84.90 (m, 3F), -116.03 – -120.40 (m, 2F). ^{13}C NMR (100 MHz, $CDCl_3$) δ 159.87 (d, *J* = 3.7 Hz), 150.29, 130.37, 129.59, 119.53, 38.26, 27.91 (dd, *J* = 31.2, 28.7 Hz), 24.15, 13.65. HRMS (ESI) calcd for $[C_{13}H_{12}F_5NNa]^+$($[M+Na]^+$): 300.0782, found: 300.0780.

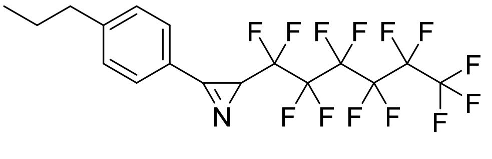

85

Following the **general procedure B**, the mixture of alkyne (0.5 mmol), alkyl iodide (0.75 mmol), $TMSN_3$ (1.0 mmol) and TBPB (1.0 mmol) in DME (2 mL) was added to $Fe(OTf)_3$ (0.025 mmol) at room temperature for 20 minutes and then in toluene at 120°C for 10 minutes to afford **85**. Yield: 137 mg, 57%; clear oil; IR (KBr): υ 2967, 2938, 2877, 1758, 1608, 1364, 1239, 1067, 840, 708 cm^{-1}; ^{1}H NMR (400 MHz, $CDCl_3$) δ 7.84 (d, *J* = 8.2 Hz, 2H), 7.41 (d, *J* = 8.2 Hz, 2H), 2.78 – 2.63 (m, 3H), 1.77 – 1.63 (m, 2H), 0.97 (t, *J* = 7.4 Hz, 3H). ^{19}F NMR (376 MHz, $CDCl_3$) δ -78.94 – -82.75 (m, 3F), -111.48 – -116.29 (m, 2F), -117.59 – -125.28 (m, 6F), -125.68 – -127.94 (m, 2F). ^{13}C NMR (100 MHz, $CDCl_3$) δ 159.87 (d, *J* = 4.0 Hz),

150.30, 130.36, 129.61, 119.60, 38.29, 28.55 (t, *J* = 29.5 Hz), 24.17, 13.65. HRMS (ESI) calcd for $[C_{17}H_{13}F_{13}N]^+([M+H]^+)$: 478.0835, found: 478.0833.

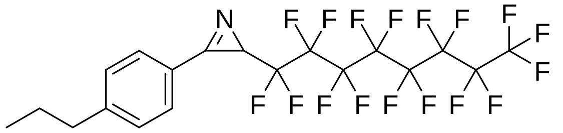

86

Following the **general procedure B**, the mixture of alkyne (0.5 mmol), alkyl iodide (0.75 mmol), $TMSN_3$ (1.0 mmol) and TBPB (1.0 mmol) in DME (2 mL) was added to $Fe(OTf)_3$ (0.025 mmol) at room temperature for 20 minutes and then in toluene at 120°C for 10 minutes to afford **86**. Yield: 169 mg, 58%; clear oil; IR (KBr): υ 2967, 2938, 2878, 1758, 1608, 1208, 1151, 988, 839, 706 cm^{-1}; ^1H NMR (400 MHz, $CDCl_3$) δ 7.84 (d, *J* = 8.1 Hz, 2H), 7.41 (d, *J* = 7.9 Hz, 2H), 2.75 – 2.64 (m, 3H), 1.76 – 1.64 (m, 2H), 0.97 (t, *J* = 7.3 Hz, 3H). ^{19}F NMR (376 MHz, $CDCl_3$) δ -79.76 – -82.46 (m, 3F), -112.30 – -116.04 (m, 2F), -116.23 – -125.71 (m, 10F), -125.94 – -127.56 (m, 2F). ^{13}C NMR (100 MHz, $CDCl_3$) δ 159.90 (d, *J* = 4.0 Hz), 150.31, 130.35, 129.60, 119.66, 38.29, 28.57 (t, *J* = 29.6 Hz), 24.17, 13.58. HRMS (ESI) calcd for $[C_{19}H_{12}F_{17}NNa]^+([M+Na]^+)$: 600.0590, found: 600.0599.

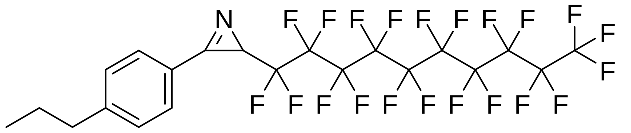

87

Following the **general procedure B**, the mixture of alkyne (0.5 mmol), alkyl iodide (0.75 mmol), $TMSN_3$ (1.0 mmol) and TBPB (1.0 mmol) in DME (2 mL) was added to $Fe(OTf)_3$ (0.025 mmol) at room temperature for 20 minutes and then in toluene at 120°C for 10 minutes to afford **87**. Yield: 194 mg, 57%; white solid; mp 50-51 °C; IR (KBr): υ 2967, 2938, 2878, 1758, 1608, 1208, 1151, 988, 839, 706 cm^{-1}; ^1H NMR (400 MHz, $CDCl_3$) δ 7.83 (d, *J* = 8.2 Hz, 2H), 7.41 (d, *J* = 8.2 Hz, 2H), 2.75 – 2.63 (m, 3H), 1.75 – 1.65 (m, 2H), 0.97 (t, *J* = 7.3 Hz, 3H). ^{19}F NMR (376 MHz, $CDCl_3$) δ -79.58 – -82.78 (m, 3F), -112.51 – -115.92 (m, 2F), -116.25 – -125.87 (m, 14F), -126.07 – -127.53 (m, 2F). ^{13}C NMR (100 MHz, $CDCl_3$) δ 159.91 (d, *J* = 4.0 Hz), 150.30, 130.35, 129.59, 119.67, 38.29, 28.58 (t, *J* = 29.6 Hz), 24.17, 13.58. HRMS (ESI) calcd for $[C_{21}H_{12}F_{21}NNa]^+([M+Na]^+)$: 700.0527, found: 700.0531.

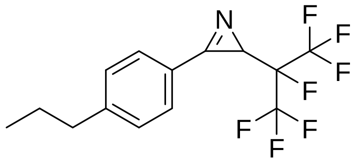

88

Following the **general procedure B**, the mixture of alkyne (0.5 mmol), alkyl iodide (0.75 mmol), $TMSN_3$ (1.0 mmol) and TBPB (1.0 mmol) in DME (2 mL) was added to $Fe(OTf)_3$ (0.025 mmol) at room temperature for 20 minutes and then in toluene at 120°C for 10 minutes to afford **88**. Yield: 114 mg, 69%; clear oil; IR (KBr): υ 2966, 2937, 2877, 1752, 1608, 1306, 1177, 997, 837, 723 cm^{-1}; ^{1}H NMR (400 MHz, $CDCl_3$) δ 7.84 (d, *J* = 8.2 Hz, 2H), 7.40 (d, *J* = 8.4 Hz, 2H), 2.74 – 2.60 (m, 3H), 1.76 – 1.63 (m, 2H), 0.97 (t, *J* = 7.3 Hz, 3H). ^{19}F NMR (376 MHz, $CDCl_3$) δ -73.61 – -77.32 (m, 6F), -185.25 – -188.91 (m, 1F). ^{13}C NMR (100 MHz, $CDCl_3$) δ 160.85 (d, *J* = 2.7 Hz), 150.18, 130.07, 129.54, 119.86, 38.26, 27.71 (d, *J* = 22.4 Hz), 24.16, 13.67. HRMS (ESI) calcd for $[C_{14}H_{12}F_7NNa]^+$($[M+Na]^+$): 350.0750, found: 350.0746.

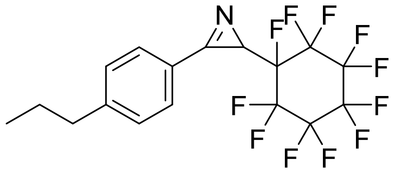

89

Following the **general procedure B**, the mixture of alkyne (0.5 mmol), alkyl iodide (0.75 mmol), $TMSN_3$ (1.0 mmol) and TBPB (1.0 mmol) in DME (2 mL) was added to $Fe(OTf)_3$ (0.025 mmol) at room temperature for 20 minutes and then in toluene at 120°C for 10 minutes to afford **89**. Yield: 159 mg, 72%; light yellow solid; mp 37-38 °C; IR (KBr): υ 2966, 2937, 2877, 1753, 1608, 1317, 1225, 1029, 964, 741 cm^{-1}; ^{1}H NMR (400 MHz, $CDCl_3$) δ 7.86 (d, *J* = 8.0 Hz, 2H), 7.41 (d, *J* = 8.2 Hz, 2H), 2.79 (dt, *J* = 16.6, 2.1 Hz, 1H), 2.70 (t, *J* = 7.6 Hz, 2H), 1.76 – 1.64 (m, 2H), 0.97 (t, *J* = 7.3 Hz, 3H). ^{19}F NMR (376 MHz, $CDCl_3$) δ -106.39 – -152.97 (m, 10F), -185.60(s, 1F). ^{13}C NMR (100 MHz, $CDCl_3$) δ 160.43 (d, *J* = 2.9 Hz), 150.29, 130.15 (d, *J* = 2.0 Hz), 129.57, 119.81, 38.28, 25.26 (d, *J* = 22.6 Hz), 24.16, 13.69. HRMS (ESI) calcd for $[C_{17}H_{12}F_{11}NNa]^+$($[M+Na]^+$): 462.0686, found: 462.0685.

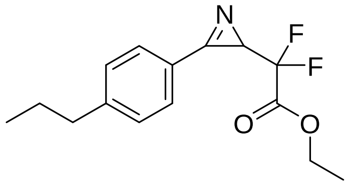

90

Following the **general procedure B**, the mixture of alkyne (0.5 mmol), alkyl iodide (0.75 mmol), $TMSN_3$ (1.0 mmol) and TBPB (1.0 mmol) in DME (2 mL) was added to $Fe(OTf)_3$ (0.025 mmol) at room temperature for 20 minutes and then in toluene at 120 °C for 10 minutes to afford **90**. Yield: 43 mg, 30%; clear oil; IR (KBr): υ 2964, 2935, 2874, 1769, 1607, 1467, 1306, 1077, 837, 740 cm^{-1}; ^1H NMR (400 MHz, $CDCl_3$) δ 7.84 (d, *J* = 8.2 Hz, 2H), 7.39 (d, *J* = 8.3 Hz, 2H), 4.34 (q, *J* = 7.1 Hz, 2H), 2.75 – 2.65 (m, 3H), 1.76 – 1.62 (m, 2H), 1.31 (t, *J* = 7.2 Hz, 3H), 0.96 (t, *J* = 7.3 Hz, 3H). ^{19}F NMR (376 MHz, $CDCl_3$) δ -104.73 (d, *J* = 262.2 Hz, 1F), -107.43 (d, *J* = 262.2 Hz, 1F). ^{13}C NMR (100 MHz, $CDCl_3$) δ 163.13 (t, *J* = 33.7 Hz), 161.81 (t, *J* = 2.1 Hz), 149.85, 130.35, 129.43, 120.21 (t, *J* = 1.4 Hz), 113.71 (dd, *J* = 251.9, 249.2 Hz), 63.02, 38.21, 31.27 (dd, *J* = 35.7, 32.4 Hz), 24.15, 13.83, 13.65. HRMS (ESI) calcd for $[C_{15}H_{17}F_2NNaO_2]^+$($[M+Na]^+$): 304.1120, found: 304.1122.

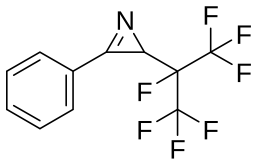

91

Following the **general procedure B**, the mixture of alkyne (0.5 mmol), alkyl iodide (0.75 mmol), $TMSN_3$ (1.0 mmol) and TBPB (1.0 mmol) in DME (2 mL) was added to $Fe(OTf)_3$ (0.025 mmol) at room temperature for 20 minutes and then in toluene at 120°C for 10 minutes to afford **91**. Yield: 96 mg, 67%; clear oil; IR (KBr): υ 3068, 2929, 1754, 1600, 1454, 1307, 1175, 997, 728 cm^{-1}; ^1H NMR (400 MHz, $CDCl_3$) δ 7.97 – 7.89 (m, 2H), 7.71 – 7.64 (m, 1H), 7.64 – 7.56 (m, 2H), 2.69 (d, *J* = 16.8 Hz, 1H). ^{19}F NMR (376 MHz, $CDCl_3$) δ -75.02 – -75.59 (m, 3F), -75.69 – -76.26 (m, 3F), -186.71 – -187.36 (m, 1F). ^{13}C NMR (100 MHz, $CDCl_3$) δ 161.42 (d, *J* = 2.7 Hz), 134.32, 130.03, 129.38, 122.44, 27.94 (d, *J* = 22.4 Hz). HRMS (EI) calcd for $[C_{11}H_6F_7N]^+$($[M]^+$): 285.0388, found: 285.0391.

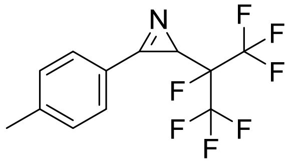

92

Following the **general procedure B**, the mixture of alkyne (0.5 mmol), alkyl iodide (0.75 mmol), $TMSN_3$ (1.0 mmol) and TBPB (1.0 mmol) in DME (2 mL) was added to $Fe(OTf)_3$ (0.025 mmol) at room temperature for 20 minutes and then in toluene at 120°C for 10 minutes to afford **92**. Yield: 95 mg, 63%; clear oil; IR (KBr): υ 3039, 2929, 1751, 1609, 1306, 1177, 998, 724 cm^{-1}; ^1H NMR (400 MHz, $CDCl_3$) δ 7.81 (d, *J* = 8.2 Hz, 2H), 7.40 (d, *J* = 7.8 Hz,

2H), 2.65 (d, *J* = 16.8 Hz, 1H), 2.47 (s, 3H). ^{19}F NMR (376 MHz, $CDCl_3$) δ -73.67 – -77.78 (m, 6F), -187.08 (p, *J* = 9.0 Hz, 1F). ^{13}C NMR (100 MHz, $CDCl_3$) δ 160.83 (d, *J* = 2.7 Hz), 145.52, 130.12, 130.05, 119.66, 27.70 (d, *J* = 22.5 Hz), 21.93. HRMS (EI) calcd for $[C_{12}H_8F_7N]^+$($[M]^+$): 299.0545, found: 299.0552.

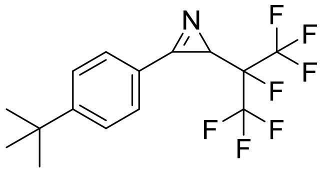

93

Following the **general procedure B**, the mixture of alkyne (0.5 mmol), alkyl iodide (0.75 mmol), $TMSN_3$ (1.0 mmol) and TBPB (1.0 mmol) in DME (2 mL) was added to $Fe(OTf)_3$ (0.025 mmol) at room temperature for 20 minutes and then in toluene at 120°C for 10 minutes to afford **93**. Yield: 125 mg, 73%; clear oil; IR (KBr): υ 2969, 2910, 2874, 1754, 1608, 1306, 1179, 841, 718 cm^{-1}; ^{1}H NMR (400 MHz, $CDCl_3$) δ 7.86 (d, *J* = 8.5 Hz, 2H), 7.62 (d, *J* = 8.7 Hz, 2H), 2.65 (d, *J* = 16.9 Hz, 1H), 1.37 (s, 9H). ^{19}F NMR (376 MHz, $CDCl_3$) δ -74.99 – -76.16 (m, 6F), -186.63 – -187.62 (m, 1F). ^{13}C NMR (100 MHz, $CDCl_3$) δ 160.79 (d, *J* = 2.6 Hz), 158.46, 129.96, 126.45, 119.58, 35.44, 31.00, 27.66 (d, *J* = 22.6 Hz). HRMS (ESI) calcd for $[C_{15}H_{14}F_7NNa]^+$($[M+Na]^+$): 364.0907, found: 364.0901.

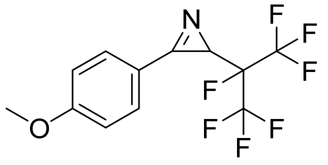

94

Following the **general procedure B**, the mixture of alkyne (0.5 mmol), alkyl iodide (0.75 mmol), $TMSN_3$ (1.0 mmol) and TBPB (1.0 mmol) in DME (2 mL) was added to $Fe(OTf)_3$ (0.025 mmol) at room temperature for 20 minutes and then in toluene at 120°C for 10 minutes to afford **94**. Yield: 95 mg, 60%; clear oil; IR (KBr): υ 2971, 2846, 1749, 1606, 1511, 1307, 838, 723 cm^{-1}; ^{1}H NMR (400 MHz, $CDCl_3$) δ 7.87 (d, *J* = 8.8 Hz, 2H), 7.08 (d, *J* = 8.8 Hz, 2H), 3.90 (s, 3H), 2.63 (d, *J* = 16.9 Hz, 1H). ^{19}F NMR (376 MHz, $CDCl_3$) δ -74.06 – -77.22 (m, 6F), -186.35 – -188.13 (m, 1F). ^{13}C NMR (100 MHz, $CDCl_3$) δ 164.39, 159.78 (d, *J* = 2.8 Hz), 132.12, 114.91, 114.74, 55.55, 27.58 (d, *J* = 22.6 Hz). HRMS (ESI) calcd for $[C_{12}H_8F_7NNaO]^+$($[M+Na]^+$): 338.0386, found: 338.0386.

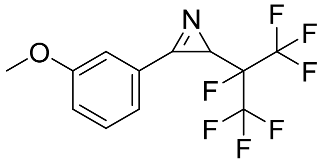

95

Following the **general procedure B**, the mixture of alkyne (0.5 mmol), alkyl iodide (0.75 mmol), $TMSN_3$ (1.0 mmol) and TBPB (1.0 mmol) in DME (2 mL) was added to $Fe(OTf)_3$ (0.025 mmol) at room temperature for 5 minutes and then in toluene at 120°C for 10 minutes to afford **95**. Yield: 97 mg, 61%; pale yellow oil; IR (KBr): υ 2966, 2841, 1754, 1601, 1487, 1306, 793, 724 cm^{-1}; ^{1}H NMR (400 MHz, $CDCl_3$) δ 7.54 – 7.47 (m, 2H), 7.46 – 7.42 (m, 1H), 7.25 – 7.17 (m, 1H), 3.89 (s, 3H), 2.69 (d, *J* = 16.9 Hz, 1H). ^{19}F NMR (376 MHz, $CDCl_3$) δ -74.65 – -76.55 (m, 6F), -186.16 – -187.94 (m, 1F). ^{13}C NMR (100 MHz, $CDCl_3$) δ 161.54 (d, *J* = 2.8 Hz), 160.09, 130.48, 123.49, 122.68, 120.99, 113.82, 55.49, 28.18 (d, *J* = 22.8 Hz). HRMS (ESI) calcd for $[C_{12}H_9F_7NO]^+$($[M+H]^+$): 316.0567, found: 316.0567.

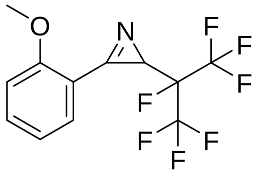

96

Following the **general procedure B**, the mixture of alkyne (0.5 mmol), alkyl iodide (0.75 mmol), $TMSN_3$ (1.0 mmol) and TBPB (1.0 mmol) in DME (2 mL) was added to $Fe(OTf)_3$ (0.025 mmol) at room temperature for 20 minutes and then in toluene at 120°C for 10 minutes to afford **96**. Yield: 89 mg, 56%; white solid; mp 62-63 ^{o}C; IR (KBr): υ 2951, 2846, 1753, 1601, 1494, 1306, 1227, 757, 725 cm^{-1}; ^{1}H NMR (400 MHz, $CDCl_3$) δ 7.69 (dd, *J* = 7.7, 1.7 Hz, 1H), 7.66 – 7.60 (m, 1H), 7.16 – 7.10 (m, 1H), 7.07 (d, *J* = 8.6 Hz, 1H), 4.00 (s, 3H), 2.52 (d, *J* = 16.8 Hz, 1H). ^{19}F NMR (376 MHz, $CDCl_3$) δ -72.90 – -78.39 (m, 6F), -185.45 – -188.86 (m, 1F). ^{13}C NMR (100 MHz, $CDCl_3$) δ 160.20, δ 157.66 (d, *J* = 2.7 Hz), 135.90, 132.67, 132.65, 120.79, 111.34, 55.86, 25.30 (d, *J* = 22.5 Hz). HRMS (ESI) calcd for $[C_{12}H_8F_7NNaO]^+$($[M+Na]^+$): 338.0386, found: 338.0386.

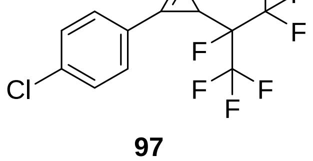

97

Following the **general procedure B**, the mixture of alkyne (0.5 mmol), alkyl iodide (0.75 mmol), $TMSN_3$ (1.0 mmol) and TBPB (1.0 mmol) in DME (2 mL) was added to $Fe(OTf)_3$

(0.025 mmol) at room temperature for 5 minutes and then in toluene at 120ºC for 10 minutes to afford **97**. Yield: 106 mg, 66%; white solid; mp 43-44 ºC; IR (KBr): υ 3420, 1755, 1596, 1489, 1307, 1227, 834, 733 cm^{-1}; ^{1}H NMR (400 MHz, $CDCl_3$) δ 7.87 (d, *J* = 8.5 Hz, 2H), 7.59 (d, *J* = 8.5 Hz, 2H), 2.71 (d, *J* = 16.8 Hz, 1H). ^{19}F NMR (376 MHz, $CDCl_3$) δ -74.50 – -76.72 (m, 6F), -186.74 – -187.36 (m, 1F). ^{13}C NMR (100 MHz, $CDCl_3$) δ 160.81 (d, *J* = 2.8 Hz), 140.98, 131.16, 129.93, 120.89, 28.14 (d, *J* = 22.4 Hz). HRMS (EI) calcd for $[C_{11}H_5ClF_7N]^+$($[M]^+$): 318.9999, found: 318.9998.

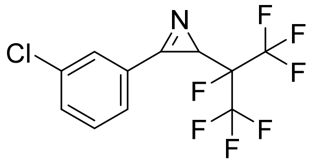

98

Following the **general procedure B**, the mixture of alkyne (0.5 mmol), alkyl iodide (0.75 mmol), $TMSN_3$ (1.0 mmol) and TBPB (1.0 mmol) in DME (2 mL) was added to $Fe(OTf)_3$ (0.025 mmol) at room temperature for 5 minutes and then in toluene at 120ºC for 10 minutes to afford **98**. Yield: 113 mg, 70%; pale yellow oil; IR (KBr): υ 3424, 3071, 2930, 1755, 1573, 1428, 1307, 1182, 1000, 719 cm^{-1}; ^{1}H NMR (400 MHz, $CDCl_3$) δ 7.94 – 7.89 (m, 1H), 7.84 – 7.79 (m, 1H), 7.69 – 7.63 (m, 1H), 7.59 – 7.52 (m, 1H), 2.73 (d, *J* = 16.7 Hz, 1H). ^{19}F NMR (376 MHz, $CDCl_3$) δ -73.87 – -77.25 (m, 6F), -184.51 – -189.02 (m, 1F). ^{13}C NMR (100 MHz, $CDCl_3$) δ 161.20 (d, *J* = 2.9 Hz), 135.65, 134.36, 130.74, 129.67, 128.05, 124.11, 28.40 (d, *J* = 22.4 Hz). HRMS (EI) calcd for $[C_{11}H_5ClF_7N]^+$($[M]^+$): 318.9999, found: 319.0007.

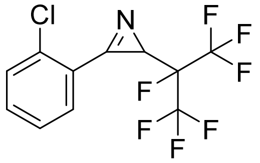

99

Following the **general procedure B**, the mixture of alkyne (0.5 mmol), alkyl iodide (0.75 mmol), $TMSN_3$ (1.0 mmol) and TBPB (1.0 mmol) in DME (2 mL) was added to $Fe(OTf)_3$ (0.025 mmol) at room temperature for 5 minutes and then in toluene at 120ºC for 10 minutes to afford **99**. Yield: 98 mg, 61%; clear oil; IR (KBr): υ 3421, 3076, 2931, 1755, 1591, 1440, 1306, 1181, 997, 714 cm^{-1}; ^{1}H NMR (400 MHz, Chloroform-*d*) δ 7.77 (d, *J* = 7.4 Hz, 1H), 7.65 – 7.56 (m, 2H), 7.55 – 7.47 (m, 1H), 2.67 (d, *J* = 16.2 Hz, 1H). ^{19}F NMR (376 MHz, Chloroform-*d*) δ -74.17 – -76.70 (m), -185.07 – -188.57 (m). ^{13}C NMR (100 MHz, $CDCl_3$) δ 159.90 (d, *J* = 3.3 Hz), 136.71, 134.75, 133.10 (d, *J* = 1.5 Hz), 130.98, 127.38, 121.37, 26.79 (d, *J* = 22.6 Hz). HRMS (EI) calcd for $[C_{11}H_5ClF_7N]^+$($[M]^+$): 318.9999, found: 319.0005.

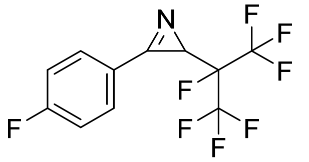

100

Following the **general procedure B**, the mixture of alkyne (0.5 mmol), alkyl iodide (0.75 mmol), $TMSN_3$ (1.0 mmol) and TBPB (1.0 mmol) in DME (2 mL) was added to $Fe(OTf)_3$ (0.025 mmol) at room temperature for 5 minutes and then in toluene at 120°C for 10 minutes to afford **100**. Yield: 85 mg, 56%; clear oil; IR (KBr): υ 3080, 1753, 1602, 1509, 1307, 1182, 998, 724 cm^{-1}; ^{1}H NMR (400 MHz, $CDCl_3$) δ 8.00 – 7.91 (m, 2H), 7.34 – 7.27 (m, 2H), 2.70 (d, *J* = 16.8 Hz, 1H). ^{19}F NMR (376 MHz, $CDCl_3$) δ -74.09 – -77.04 (m, 6F), -101.72(s, 1F) , -185.53 – -188.62 (m, 1F). ^{13}C NMR (100 MHz, $CDCl_3$) δ 166.31 (d, *J* = 257.8 Hz), 160.45 (d, *J* = 2.8 Hz), 132.54 (d, *J* = 9.6 Hz), 118.86 (d, *J* = 3.3 Hz), 117.02 (d, *J* = 22.6 Hz), 28.07 (d, *J* = 22.5 Hz). HRMS (ESI) calcd for $[C_{11}H_5F_8N]^+([M+H]^+)$: 304.0367, found: 304.0369.

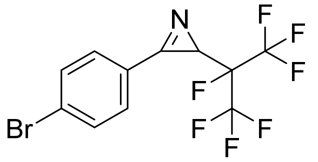

101

Following the **general procedure B**, the mixture of alkyne (0.5 mmol), alkyl iodide (0.75 mmol), $TMSN_3$ (1.0 mmol) and TBPB (1.0 mmol) in DME (2 mL) was added to $Fe(OTf)_3$ (0.025 mmol) at room temperature for 5 minutes and then in toluene at 120°C for 10 minutes to afford **101**. Yield: 99 mg, 54%; white solid; mp 55-56 °C; IR (KBr): υ 3071, 1754, 1590, 1484, 1305, 1227, 997, 732 cm^{-1}; ^{1}H NMR (400 MHz, $CDCl_3$) δ 7.84 – 7.70 (m, 4H), 2.71 (d, *J* = 16.7 Hz, 1H). ^{19}F NMR (376 MHz, $CDCl_3$) δ -73.03 – -78.00 (m, 6F), -184.66 – -189.41 (m, 1F). ^{13}C NMR (100 MHz, $CDCl_3$) δ 161.02 (d, *J* = 2.8 Hz), 132.92, 131.19, 129.62, 121.31, 28.14 (d, *J* = 22.5 Hz). HRMS (EI) calcd for $[C_{11}H_5BrF_7N]^+([M]^+)$: 362.9494, found: 362.9492.

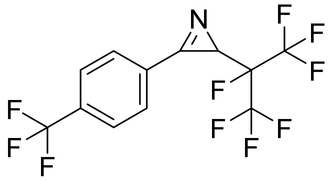

102

Following the **general procedure B**, the mixture of alkyne (0.5 mmol), alkyl iodide (0.75 mmol), $TMSN_3$ (1.0 mmol) and TBPB (1.0 mmol) in DME (2 mL) was added to $Fe(OTf)_3$ (0.025 mmol) at room temperature for 5 minutes and then in toluene at 120°C for 10 minutes

to afford **102**. Yield: 98 mg, 55%; white solid; mp 70-71 °C; IR (KBr): υ 3075, 1754, 1416, 1323, 1251, 1134, 842, 749 cm^{-1}; ^{1}H NMR (400 MHz, $CDCl_3$) δ 8.07 (d, *J* = 8.1 Hz, 2H), 7.88 (d, *J* = 8.1 Hz, 2H), 2.78 (d, *J* = 16.6 Hz, 1H). ^{19}F NMR (376 MHz, $CDCl_3$) δ -63.50(s, 3F) , -74.68 – -76.54 (m, 6F), -185.66 – -188.21 (m, 1F). ^{13}C NMR (100 MHz, $CDCl_3$) δ 161.52 (d, *J* = 2.5 Hz), 135.76 (q, *J* = 33.1 Hz), 130.30, 126.46 (q, *J* = 3.8 Hz), 125.68, 123.21 (q, *J* = 272.7 Hz), 28.48 (d, *J* = 22.6 Hz). HRMS (ESI) calcd for $[C_{12}H_6F_{10}N]^+([M+H]^+)$: 354.0335, found: 354.0336.

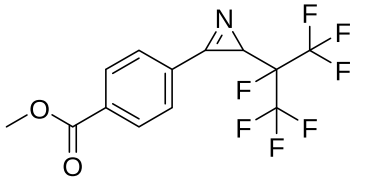

103

Following the **general procedure B**, the mixture of alkyne (0.5 mmol), alkyl iodide (0.75 mmol), $TMSN_3$ (1.0 mmol) and TBPB (1.0 mmol) in DME (2 mL) was added to $Fe(OTf)_3$ (0.025 mmol) at room temperature for 10 minutes and then in toluene at 120°C for 10 minutes to afford **103**. Yield: 114 mg, 66%; light yellow solid; mp 58-59 °C; IR (KBr): υ 3048, 2960, 1722, 1440, 1411, 1307, 1224, 1184, 864, 731 cm^{-1}; ^{1}H NMR (400 MHz, $CDCl_3$) δ 8.30 – 8.23 (m, 2H), 8.01 (dd, *J* = 8.3, 1.6 Hz, 2H), 3.99 (s, 3H), 2.76 (d, *J* = 16.7 Hz, 1H). ^{19}F NMR (376 MHz, $CDCl_3$) δ -71.69 – -79.24 (m, 6F), -182.50 – -191.57 (m, 1F). ^{13}C NMR (100 MHz, $CDCl_3$) δ 165.65, 161.55 (d, *J* = 2.8 Hz), 135.15, 130.41, 129.87, 126.03, 52.65, 28.35 (d, *J* = 22.5 Hz). HRMS (EI) calcd for $[C_{13}H_8F_7NO_2]^+([M]^+)$: 343.0443, found: 343.0440.

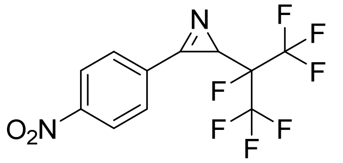

104

Following the **general procedure B**, the mixture of alkyne (0.5 mmol), alkyl iodide (0.75 mmol), $TMSN_3$ (1.0 mmol) and TBPB (1.0 mmol) in DME (2 mL) was added to $Fe(OTf)_3$ (0.025 mmol) at room temperature for 5 minutes and then in toluene at 120°C for 10 minutes to afford **104**. Yield: 92 mg, 55%; light yellow solid; mp 82-83 °C; IR (KBr): υ 3428, 3110, 1753, 1605, 1525, 1348, 1227, 1185, 864, 730 cm^{-1}; ^{1}H NMR (400 MHz, $CDCl_3$) δ 8.48 (d, *J* = 8.8 Hz, 2H), 8.14 (d, *J* = 8.8 Hz, 2H), 2.84 (d, *J* = 16.6 Hz, 1H). ^{19}F NMR (376 MHz, $CDCl_3$) δ -73.18 – -77.99 (m, 6F), -184.29 – -189.32 (m, 1F). ^{13}C NMR (100 MHz, $CDCl_3$) δ 161.50 (d, *J* = 2.7 Hz), 151.06, 130.92, 127.78, 124.58, 28.87 (d, *J* = 22.3 Hz). HRMS (ESI) calcd for $[C_{11}H_6F_7N_2O_2]^+([M+H]^+)$: 331.0312, found: 331.0312.

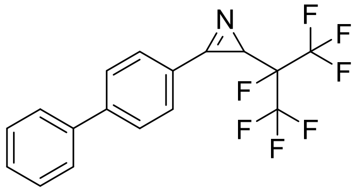

105

Following the **general procedure B**, the mixture of alkyne (0.5 mmol), alkyl iodide (0.75 mmol), $TMSN_3$ (1.0 mmol) and TBPB (1.0 mmol) in DME (2 mL) was added to $Fe(OTf)_3$ (0.025 mmol) at room temperature for 20 minutes and then in toluene at 120°C for 10 minutes to afford **105**. Yield: 142 mg, 78%; light yellow solid; mp 86-87 °C; IR (KBr): υ 3444, 1745, 1605, 1409, 1283, 1223, 1179, 842, 721 cm^{-1}; ^1H NMR (400 MHz, $CDCl_3$) δ 7.98 (d, J = 8.3 Hz, 2H), 7.79 (d, J = 8.4 Hz, 2H), 7.66 – 7.61 (m, 2H), 7.52 – 7.39 (m, 3H), 2.70 (d, J = 16.9 Hz, 1H). ^{19}F NMR (376 MHz, $CDCl_3$) δ -74.45 – -76.68 (m, 6F), -185.74 – -188.24 (m, 1F). ^{13}C NMR (100 MHz, $CDCl_3$) δ 160.96 (d, J = 2.9 Hz), 147.16, 139.36, 130.49, 129.08, 128.67, 127.99, 127.28, 121.05, 27.90 (d, J = 22.4 Hz). HRMS (ESI) calcd for $[C_{17}H_{10}F_7NNa]^+$($[M+Na]^+$): 384.0594, found: 384.0587.

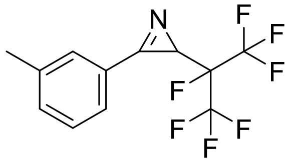

106

Following the **general procedure B**, the mixture of alkyne (0.5 mmol), alkyl iodide (0.75 mmol), $TMSN_3$ (1.0 mmol) and TBPB (1.0 mmol) in DME (2 mL) was added to $Fe(OTf)_3$ (0.025 mmol) at room temperature for 20 minutes and then in toluene at 120°C for 10 minutes to afford **106**. Yield: 92 mg, 61%; clear oil; IR (KBr): υ 3037, 1753, 1607, 1307, 1186, 992, 721 cm^{-1}; ^1H NMR (400 MHz, $CDCl_3$) δ 7.78 – 7.68 (m, 2H), 7.52 – 7.44 (m, 2H), 2.66 (d, J = 16.8 Hz, 1H), 2.46 (s, 3H). ^{19}F NMR (376 MHz, $CDCl_3$) δ -74.41 – -76.83 (m, 6F), -185.40 – -188.68 (m, 1F). ^{13}C NMR (100 MHz, $CDCl_3$) δ 161.40 (d, J = 2.6 Hz), 139.42, 135.17, 130.33, 129.24, 127.29, 122.32, 27.89 (d, J = 22.4 Hz), 21.16. HRMS (EI) calcd for $[C_{12}H_8F_7N]^+$($[M]^+$): 299.0545, found: 299.0549.

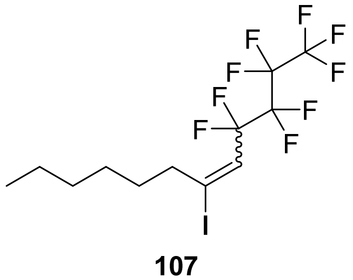

107

To a dried Schlenk tube equipped with a magnetic bar, $Fe(OTf)_3$ (12.7 mg, 0.025 mmol) was added, Then this tube was flushed with nitrogen gas (3 times) and maintained a nitrogen atmosphere using a nitrogen balloon. A thoroughly mixed solution of alkynes (0.5 mmol), R_fI (0.75 mmol), $TMSN_3$ (1.0 mmol) and TBPB (1.0 mmol) in DME (2 mL) was added to the catalyst via syringe and stirred vigorously for 10 minutes at room temperature. After completion (TLC), the solvent was evaporated and the residue was purified by flash chromatography on silica gel using petroleum ether and ethyl acetate to give the corresponding product **107**. Yield: 96 mg, 42%; E/Z = 3.7:1; clear oil; ^{1}H NMR (400 MHz, $CDCl_3$) δ 6.32 (t, J = 14.5 Hz, 0.81H), 6.23 (t, J = 13.0 Hz, 0.19H), 2.72 – 2.57 (m, 2H), 1.65 – 1.50 (m, 2H), 1.40 – 1.22 (m, 6H), 0.90 (t, J = 6.7 Hz, 3H). ^{19}F NMR (376 MHz, $CDCl_3$) δ -76.08 – -86.12 (m, 3F), -102.45 – -110.47 (m, 2F), -119.19 – -131.64 (m, 4F). ^{13}C NMR (100 MHz, $CDCl_3$) δ 126.32 (t, J = 23.8 Hz), 123.14, 121.49 (t, J = 23.7 Hz), 48.41, 41.15, 31.45, 31.41, 30.01, 28.99, 28.09, 27.66, 22.46, 13.97, 13.94. The NMR data is consistent with the reported value.[5]

Applications of the products from carbonazidation of alkynes

(a) Synthesis of 1,2,3-triazole 108

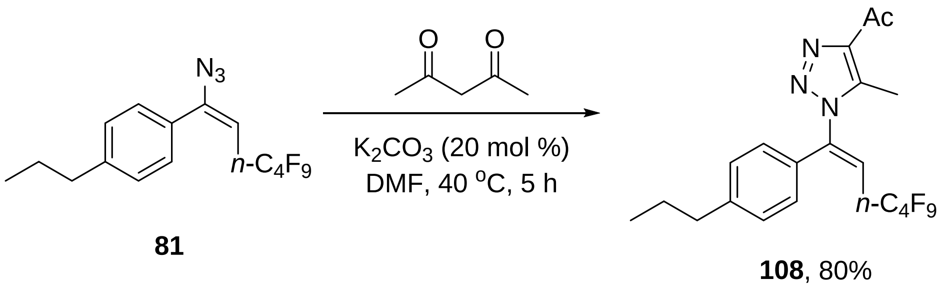

To a solution of vinyl azide **81** (0.5 mmol) and acetylacetone (1.0 mmol) in DMF (5 mL) was added K_2CO_3 (0.1 mmol), and the reaction mixture was stirred at 40 °C for 5 h. The reaction mixture was quenched with water, and then extracted twice with ethyl acetate. The combined organic extracts were washed with water and brine, dried over $MgSO_4$. The solvent was evaporated and the residue was purified by flash chromatography on silica gel using petroleum ether and ethyl acetate to give the corresponding product **108** (yield: 196 mg, 80%).[6]

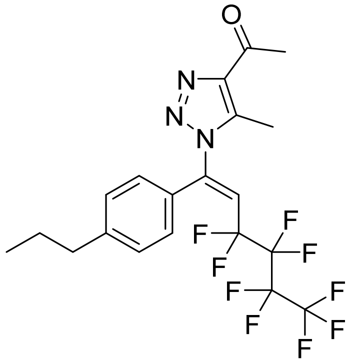

108

Clear oil; IR (KBr): υ 2965, 2875, 1684, 1557, 1417, 1259, 1134, 1025, 881, 741 cm^{-1}; ^{1}H NMR (400 MHz, $CDCl_3$) δ 7.28 – 7.20 (m, 4H), 6.33 (t, *J* = 14.0 Hz, 1H), 2.70 (s, 3H), 2.67 – 2.61 (m, 2H), 2.14 (s, 3H), 1.73 – 1.60 (m, 2H), 0.94 (t, *J* = 7.3 Hz, 3H). ^{19}F NMR (376 MHz, $CDCl_3$) δ -78.89 – -83.45 (m, 3F), -103.88 – -105.75 (m, 2F), -122.25 – -124.60 (m, 2F), -124.81 – -127.31 (m, 2F). ^{13}C NMR (100 MHz, $CDCl_3$) δ 194.08, 146.63, 145.62 (t, *J* = 4.3 Hz), 144.01, 138.26, 128.87 (t, *J* = 2.7 Hz), 128.84, 128.48, 114.23 (t, *J* = 22.3 Hz), 37.76, 27.86, 24.05, 13.57, 9.80. HRMS (ESI) calcd for $[C_{20}H_{18}F_9N_3NaO]^+$($[M+Na]^+$): 510.1198, found: 510.1197.

(b) Synthesis of 1,2,3-triazole 109

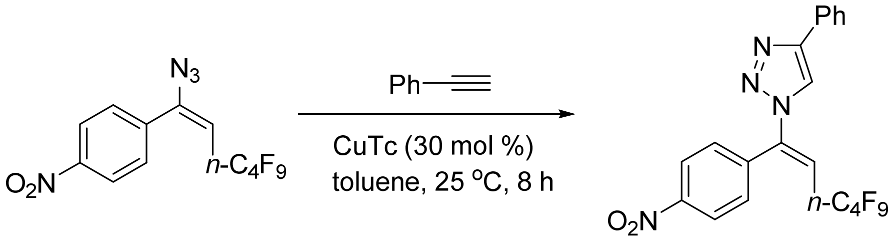

109, 81%

To a solution of vinyl azide (0.5 mmol) and phenylacetylene (1.0 mmol) in toluene (2 mL) was added CuTc (0.15 mmol), and the reaction mixture was stirred at 25°C for 8 h. After completion (TLC), the solvent was evaporated and the residue was purified by flash chromatography on silica gel using petroleum ether and ethyl acetate to give the corresponding product **109** (yield: 207 mg, 81%).[7]

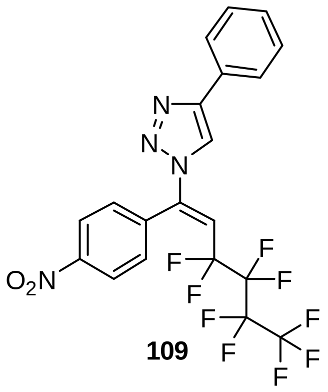

109

White solid; IR (KBr): υ 3087, 1671, 1605, 1526, 1350, 1233, 1134, 1015, 883, 766, 696 cm^{-1}; ^{1}H NMR (600 MHz, $CDCl_3$) δ 8.45 – 8.36 (m, 2H), 7.80 – 7.72 (m, 2H), 7.71 – 7.63 (m, 2H), 7.46 – 7.31 (m, 4H), 7.03 (t, *J* = 13.9 Hz, 1H); ^{19}F NMR (376 MHz, $CDCl_3$) δ -78.81 – -83.41 (m, 3F), -103.41 – -105.63 (m, 2F), -122.58 – -124.34 (m, 2F), -124.55 – -126.70 (m, 2F). ^{13}C NMR (150 MHz, $CDCl_3$) δ 149.29, 148.77, 142.56, 136.53, 130.73, 129.04, 128.97, 128.93, 125.88, 124.10, 118.72, 108.50 (t, J = 22.9 Hz). HRMS (ESI) calcd for $[C_{20}H_{11}F_9N_4NaO_2]^+$($[M+Na]^+$): 533.0631, found: 533.0623.

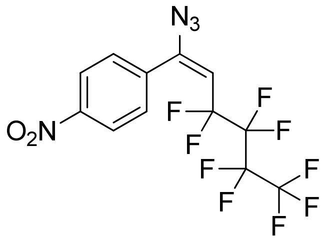

To a dried Schlenk tube equipped with a magnetic bar, $Fe(OTf)_3$ (12.7 mg, 0.025 mmol) was added, Then this tube was flushed with nitrogen gas (3 times) and maintained a nitrogen atmosphere using a nitrogen balloon. A thoroughly mixed solution of alkyne (0.5 mmol), C_4F_9I (0.75 mmol), $TMSN_3$ (1.0 mmol) and TBPB (1.0 mmol) in DME (2 mL) was added to the catalyst via syringe and stirred vigorously for 5 minutes at room temperature. After completion (TLC), the solvent was evaporated and the residue was purified by flash chromatography on silica gel using petroleum ether and ethyl acetate to give the corresponding vinyl azide product. Yield: 131 mg, 64%; pale yellow oil; IR (KBr): υ 2963, 2919, 2866, 2116, 1653, 1528, 1348, 1259, 1095, 1019, 799, 702 cm^{-1}; ^{1}H NMR (400 MHz, $CDCl_3$) δ 8.33 (d, *J* = 8.8 Hz, 2H), 7.56 (d, *J* = 8.7 Hz, 2H), 5.59 (t, *J* = 13.9 Hz, 1H). ^{19}F NMR (376 MHz, $CDCl_3$) δ -78.77 – -83.31 (m, 3F), -101.95 – -104.73 (m, 2F), -122.26 – -124.60 (m, 2F), -124.82 – -126.84 (m, 2F). ^{13}C NMR (100 MHz, $CDCl_3$) δ 149.23 (t, *J* = 5.0

Hz), 148.82, 137.45, 129.45 (t, J = 2.7 Hz), 123.82, 103.22 (t, J = 23.0 Hz). HRMS (EI) calcd for $[C_{12}H_5F_9N_2O_2]^+$($[M-N_2]^+$): 380.0207, found: 380.0204.

(c) Transformation of 2H-azirine

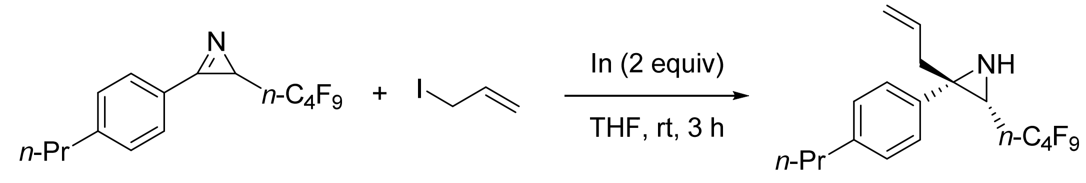

A mixture of 3-iodoprop-1-ene (1.5 mmol) and indium powder (1.0 mmol) in THF (2 mL) was stirred at room temperature for 1 h. To the resulting solution, **82** (0.50 mmol) was added and the mixture was stirred at room temperature for another 3 h. The reaction was quenched with water (10 mL) and the product was extracted with ether. The extracts were washed successively with water, brine, and dried over Na_2SO_4. The solvent was evaporated and the residue was purified by flash chromatography on silica gel using petroleum ether and ethyl acetate to give the corresponding product **110** (yield: 196 mg, 93%).[8]

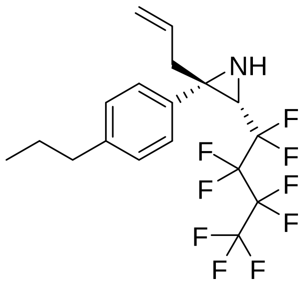

110

Clear oil; IR (KBr): υ 3293, 2963, 2934, 2875, 1642, 1518, 1436, 1356, 1235, 1133, 925, 726 cm^{-1}; ^1H NMR (400 MHz, $CDCl_3$) δ 7.35 (d, J = 7.8 Hz, 2H), 7.13 (d, J = 8.0 Hz, 2H), 5.77 – 5.58 (m, 1H), 5.16 (dd, J = 29.2, 13.6 Hz, 2H), 2.85 – 2.50 (m, 5H), 1.70 – 1.57 (m, 2H), 1.41 (d, J = 9.9 Hz, 1H), 0.92 (t, J = 7.3 Hz, 3H). ^{19}F NMR (376 MHz, $CDCl_3$) δ -80.34 – -81.85 (m, 3F), -113.78 – -120.44 (m, 2F), -122.49 – -125.63 (m, 2F), -125.76 – -126.71 (m, 2F). ^{13}C NMR (100 MHz, $CDCl_3$) δ 141.81, 134.78, 130.99, 128.08, 127.82, 120.91, 44.89, 43.92, 37.70, 36.94 (dd, J = 33.3, 20.9 Hz), 24.34, 13.75. HRMS (ESI) calcd for $[C_{18}H_{18}F_9NNa]^+$($[M+Na]^+$): 442.1188, found: 442.1184.

Single crystal data of compound 109

Crystal data and structure refinements of **109** is listed in Supplementary Table 3. CCDC number is 1864994. These data can be obtained free of charge from the Cambridge Crystallographic Data Centre via www.ccdc.cam.ac.uk/data_request/cif.

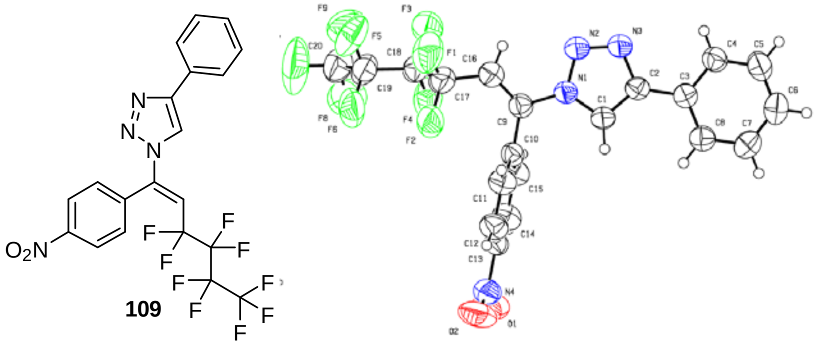

Supplementary Figure 1. X-ray crystal structure of **109**

Supplementary Table 3 Crystal data and structure refinement for compound **109.**

Identification code	109
Empirical formula	C20 H11 F9 N4 O2
Formula weight	510.33
Temperature	293(2) K
Wavelength	0.71073 A
Crystal system, space group	Monoclinic, P 21/n
Unit cell dimensions	a = 9.648(4) A alpha = 90 deg.
	b = 8.528(3) A beta = 92.544(7) deg.
	c = 26.015(10) A gamma = 90 deg.
Volume	2138.4(14) A^3
Z, Calculated density	4, 1.585 Mg/m^3
Absorption coefficient	0.158 mm^-1
F(000)	1024
Crystal size	0.38 x 0.36 x 0.18 mm
Theta range for data collection	2.221 to 27.499 deg.
Limiting indices	-12<=h<=12, -10<=k<=10, -33<=l<=33
Reflections collected / unique	26879 / 4890 [R(int) = 0.0253]
Completeness to theta = 25.242	99.80%
Absorption correction	Semi-empirical from equivalents
Max. and min. transmission	1.0000 and 0.9587
Refinement method	Full-matrix least-squares on F^2
Data / restraints / parameters	4890 / 12 / 316
Goodness-of-fit on F^2	1.168
Final R indices [I>2sigma(I)]	R1 = 0.0646, wR2 = 0.1828
R indices (all data)	R1 = 0.0771, wR2 = 0.1954
Extinction coefficient	n/a
Largest diff. peak and hole	0.350 and -0.339 e.A^-3

Copies of ^{1}H, ^{19}F and ^{13}C NMR spectra

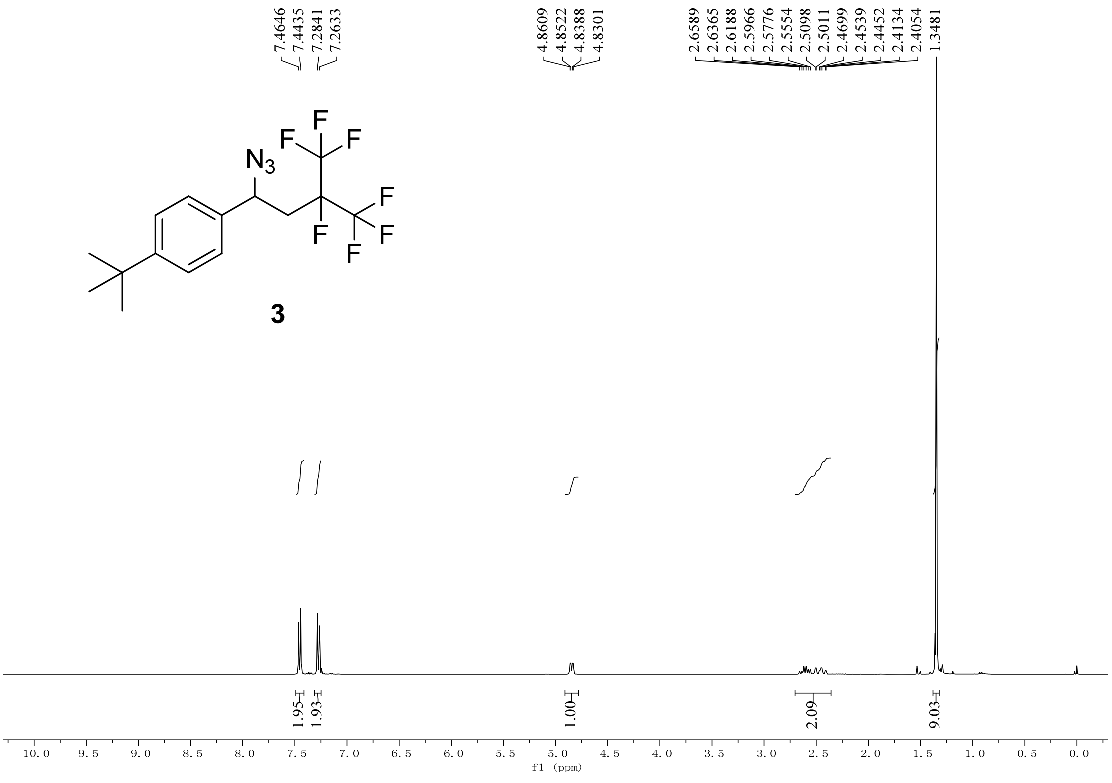

Supplementary Figure 2. ^{1}H NMR spectrum for compound **3**

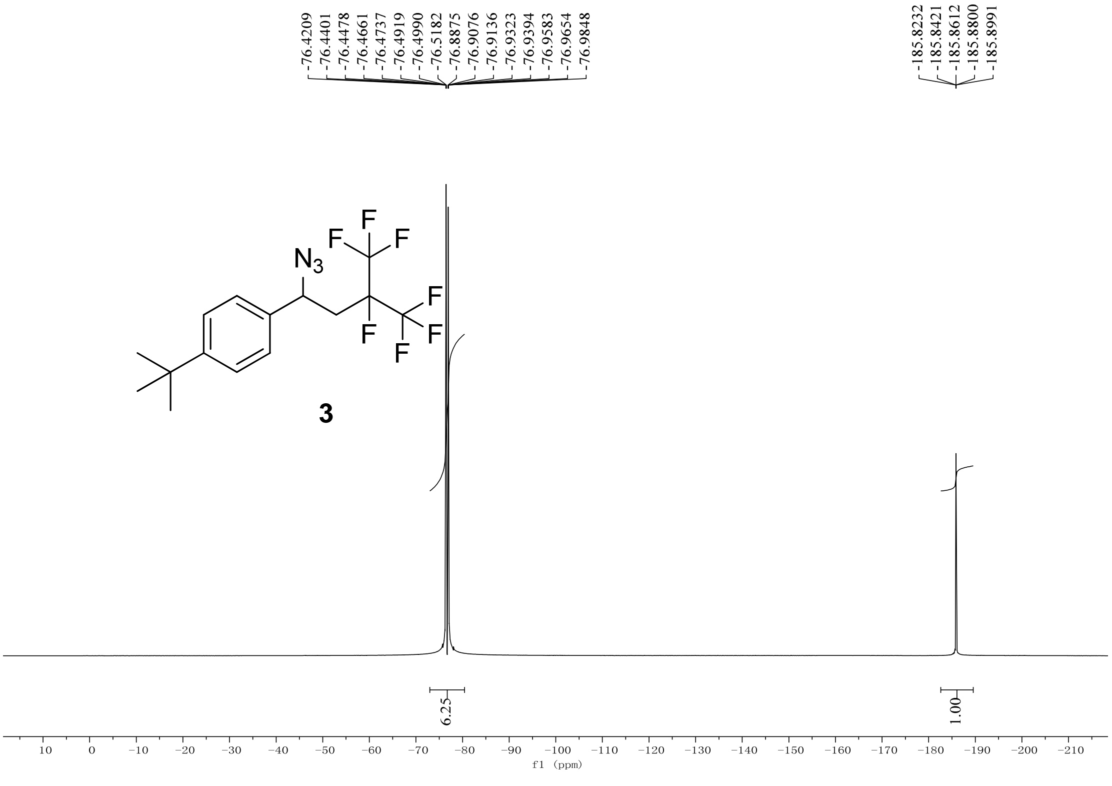

Supplementary Figure 3. ^{19}F NMR spectrum for compound **3**

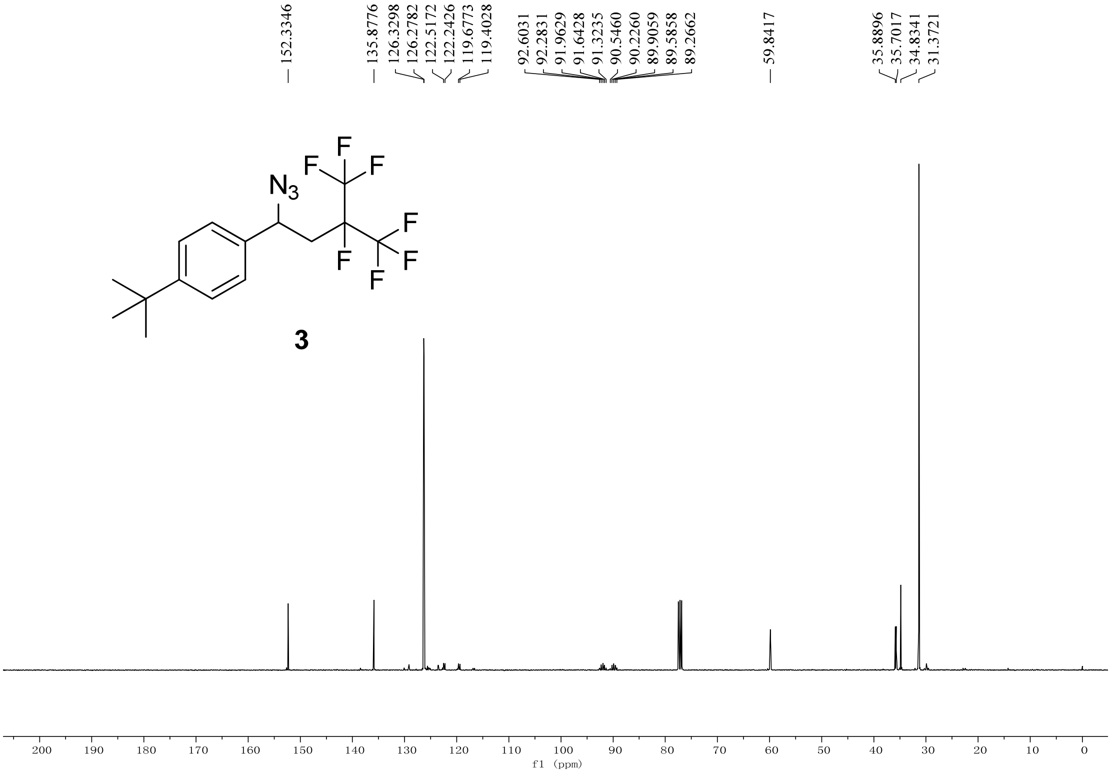

Supplementary Figure 4. ^{13}C NMR spectrum for compound **3**

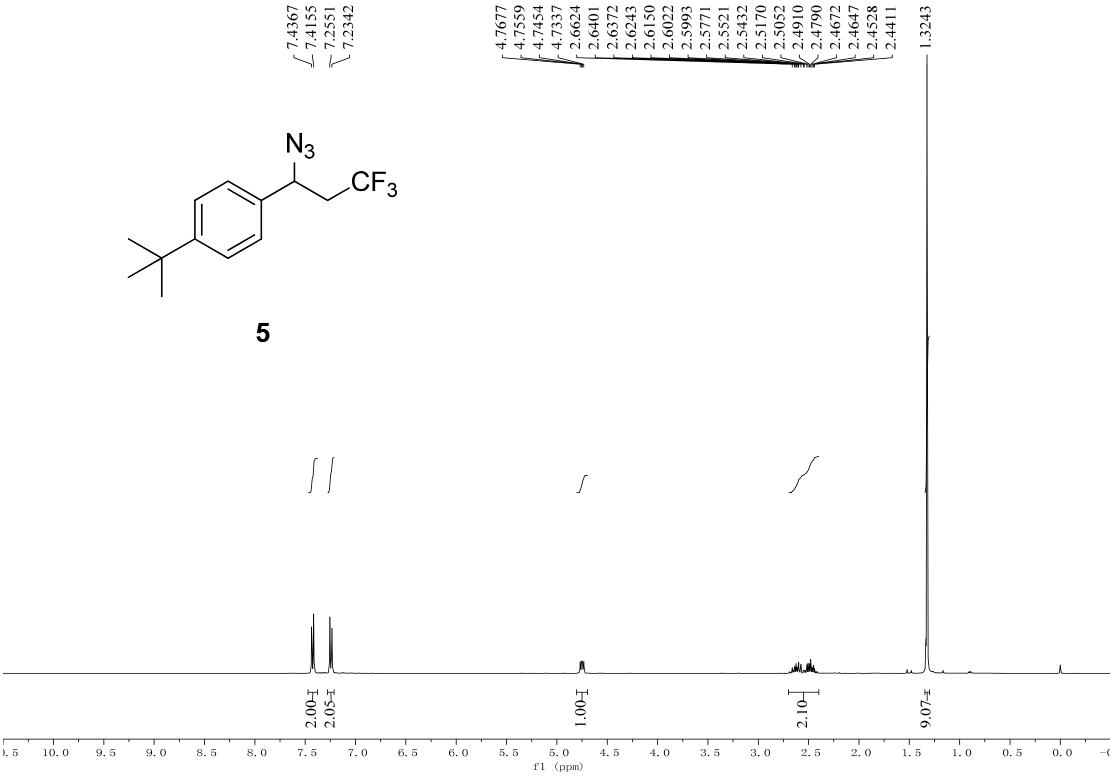

Supplementary Figure 5. ^{1}H NMR spectrum for compound **5**

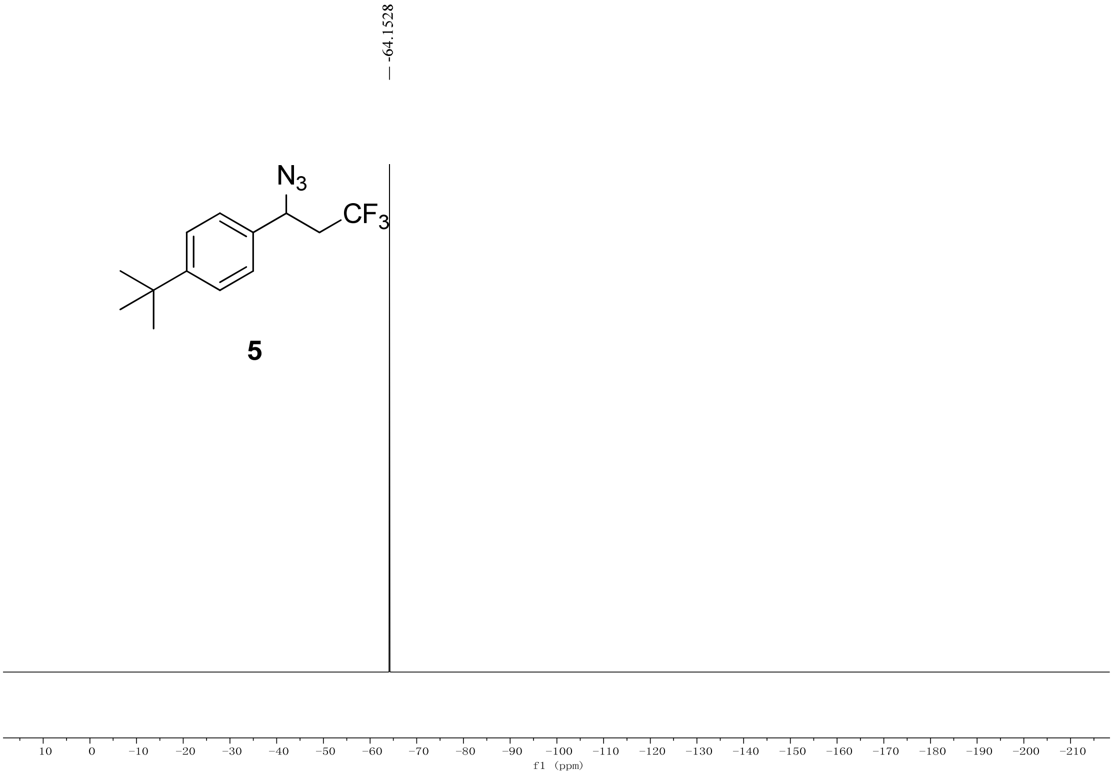

Supplementary Figure 6. ^{19}F NMR spectrum for compound **5**

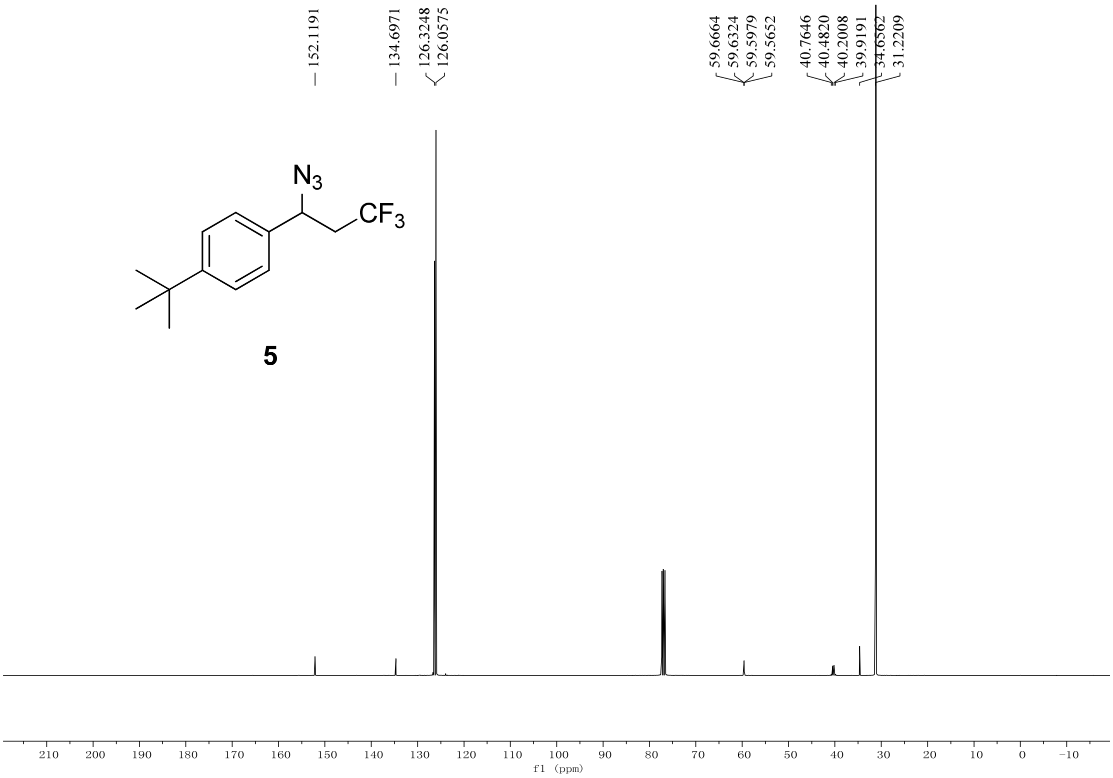

Supplementary Figure 7. ^{13}C NMR spectrum for compound **5**

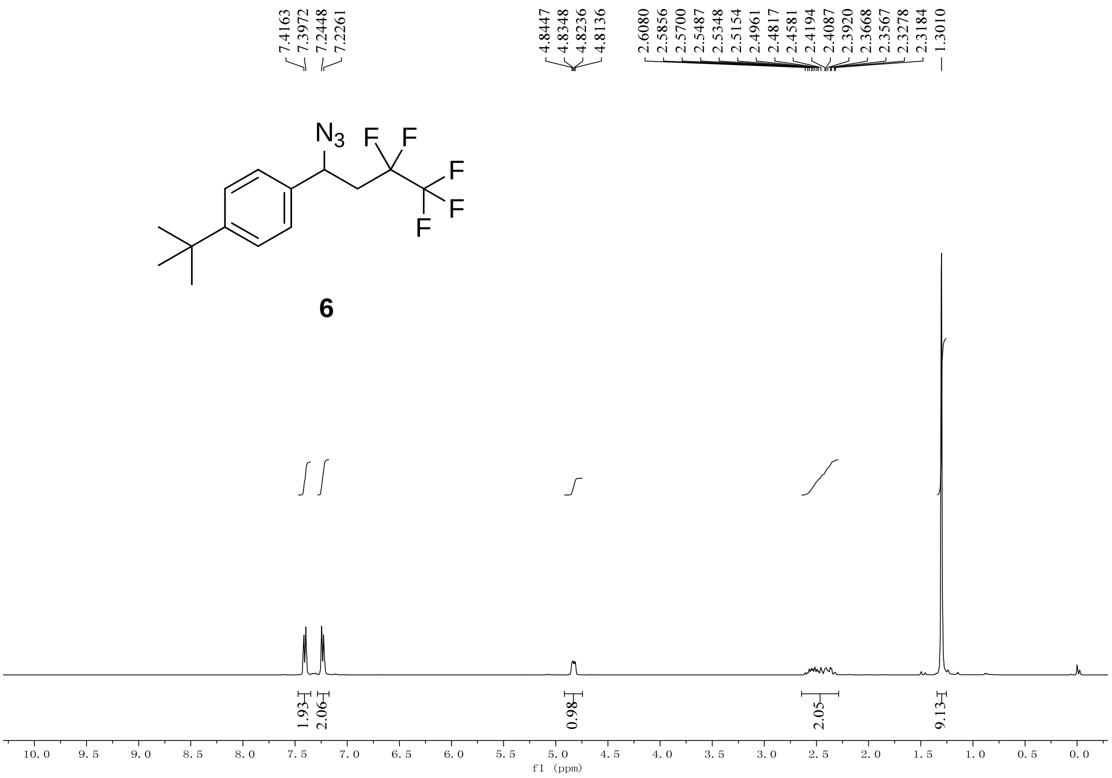

Supplementary Figure 8. ^{1}H NMR spectrum for compound **6**

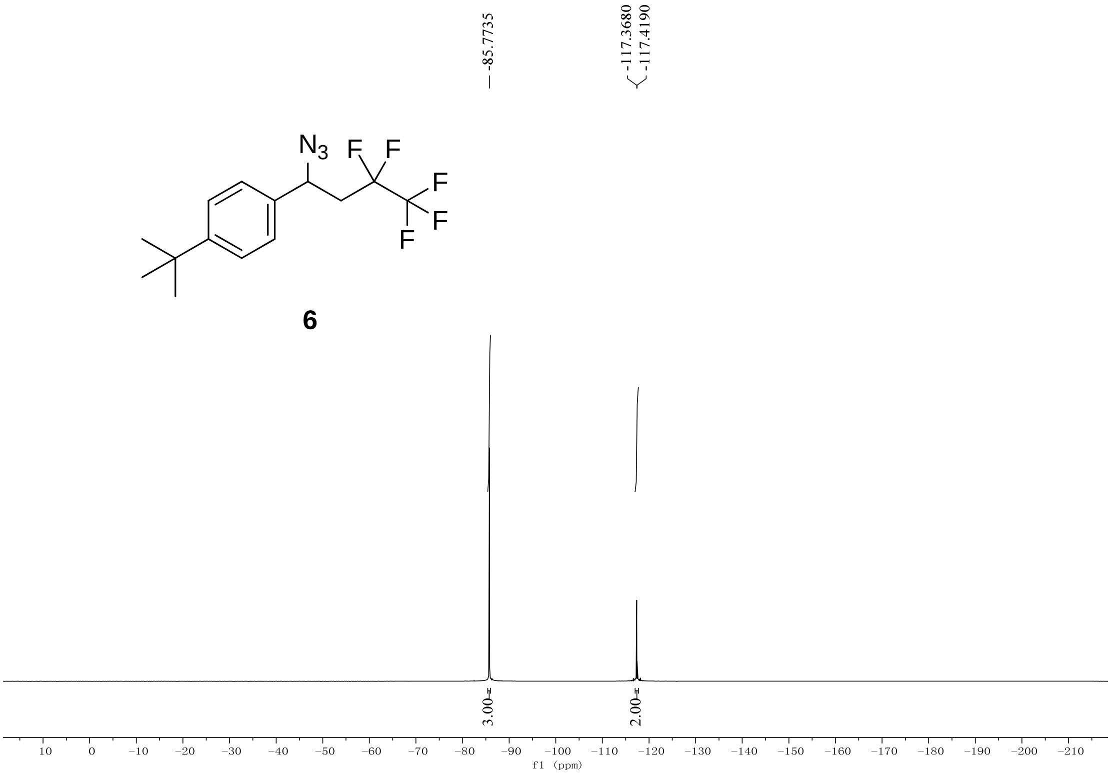

Supplementary Figure 9. ^{19}F NMR spectrum for compound **6**

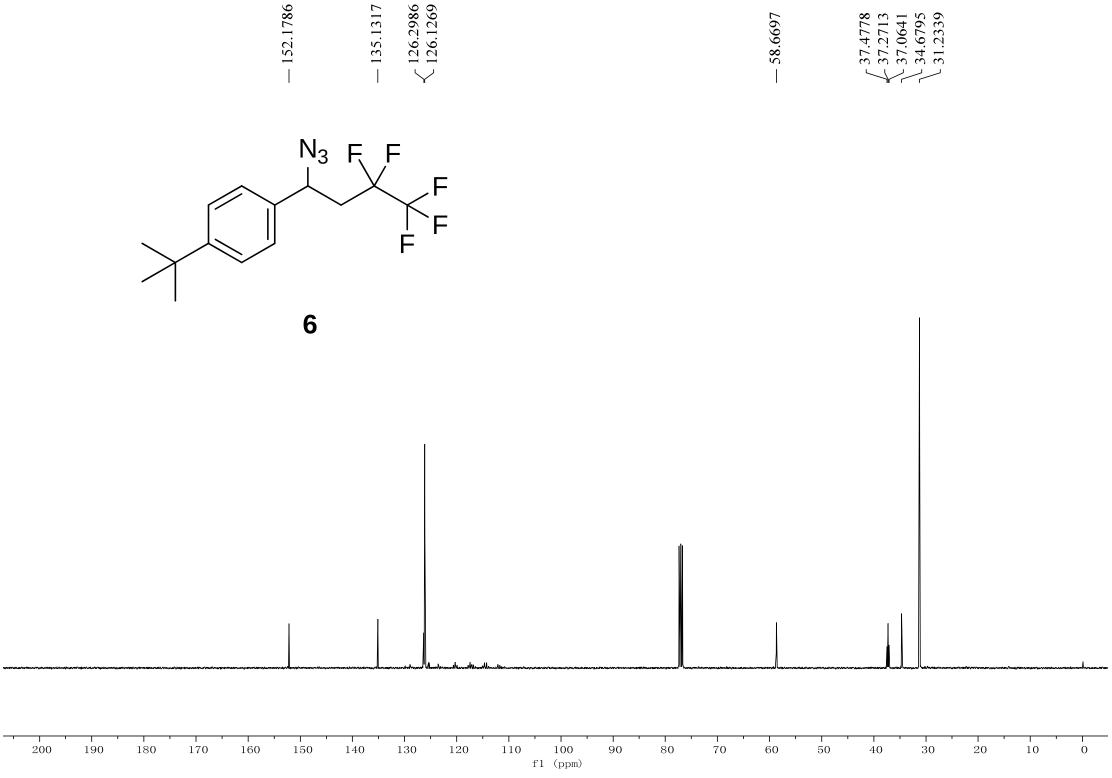

Supplementary Figure 10. ^{13}C NMR spectrum for compound **6**

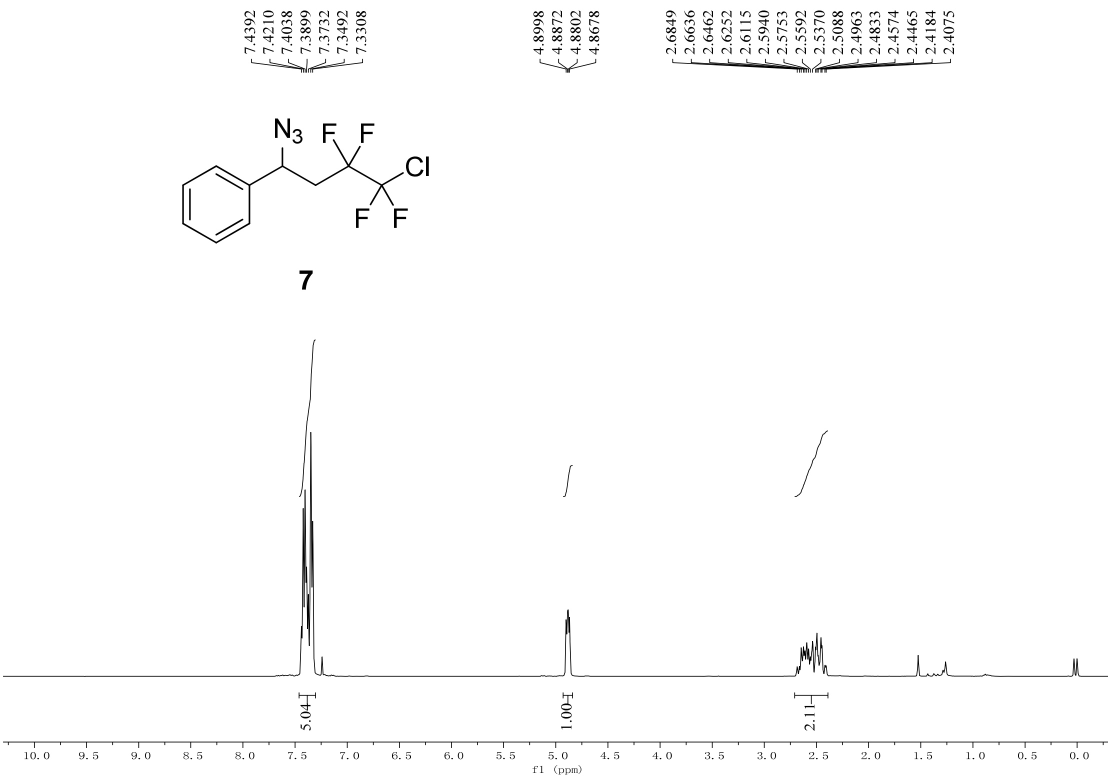

Supplementary Figure 11. ^{1}H NMR spectrum for compound **7**

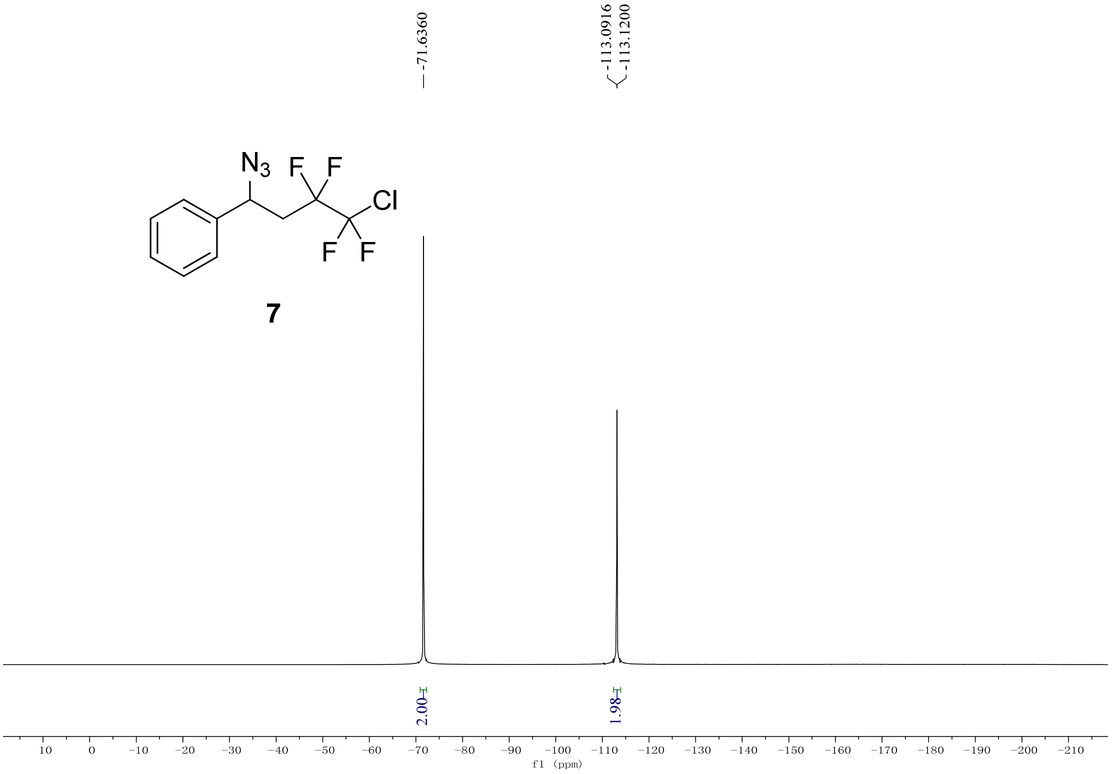

Supplementary Figure 12. ^{19}F NMR spectrum for compound **7**

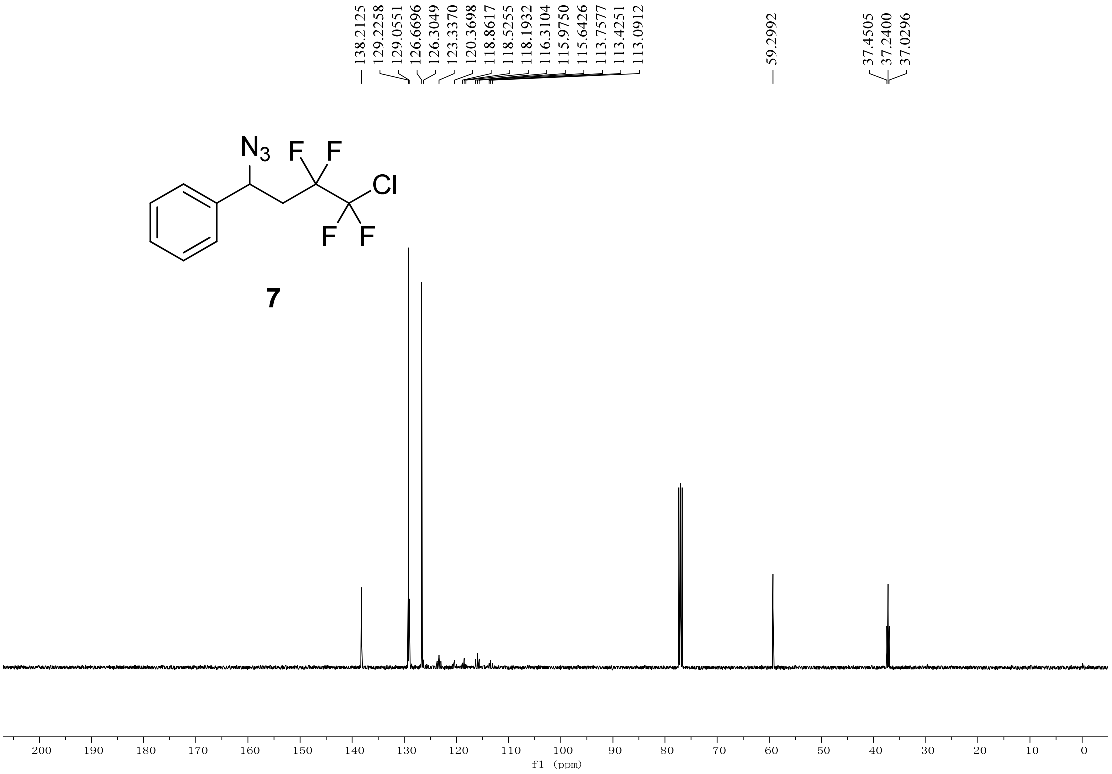

Supplementary Figure 13. ^{13}C NMR spectrum for compound **7**

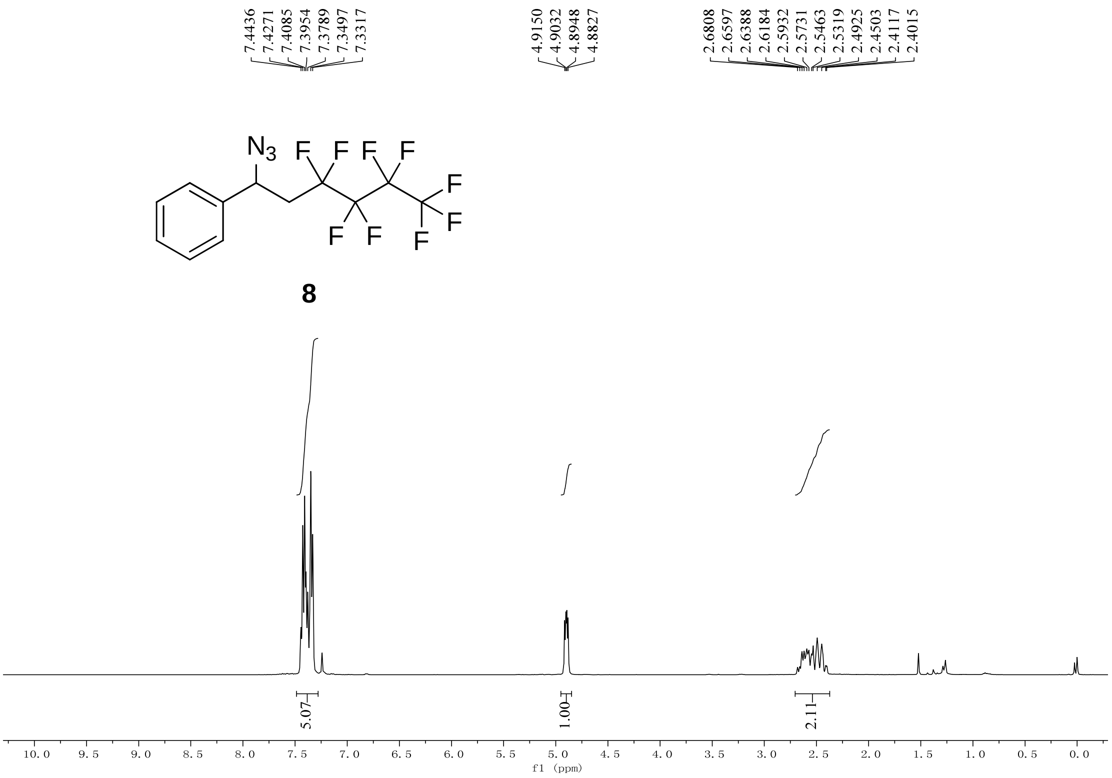

Supplementary Figure 14. ^{1}H NMR spectrum for compound **8**

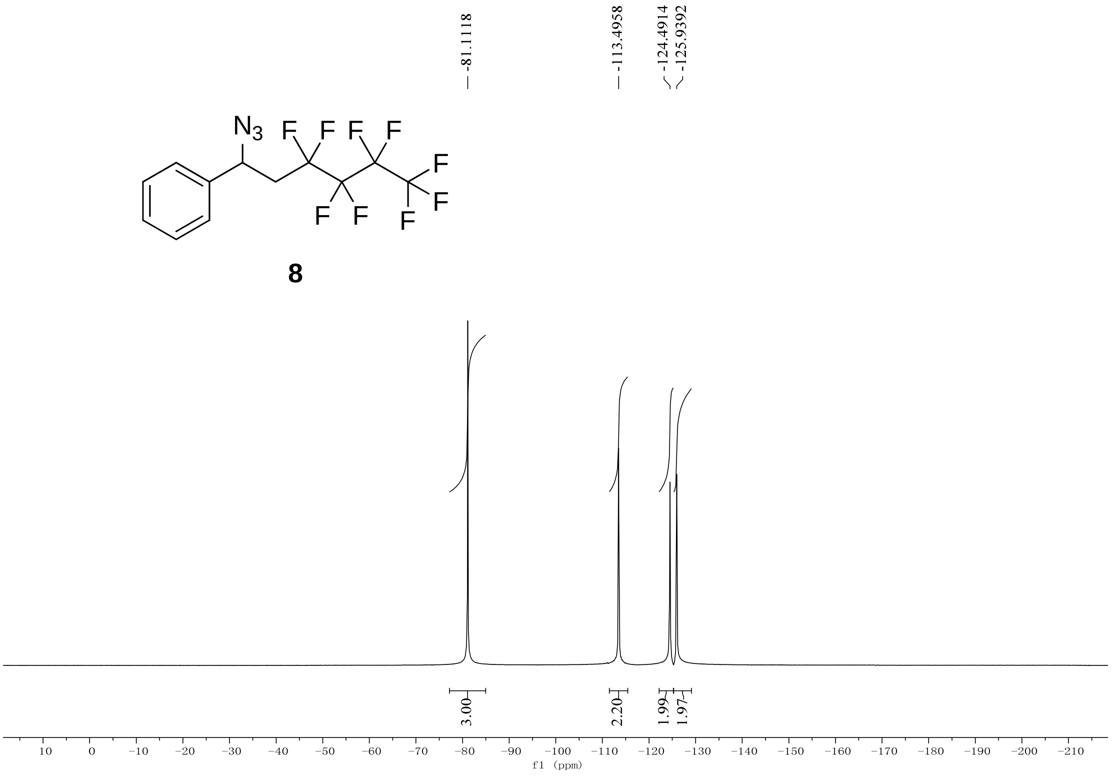

Supplementary Figure 15. ^{19}F NMR spectrum for compound **8**

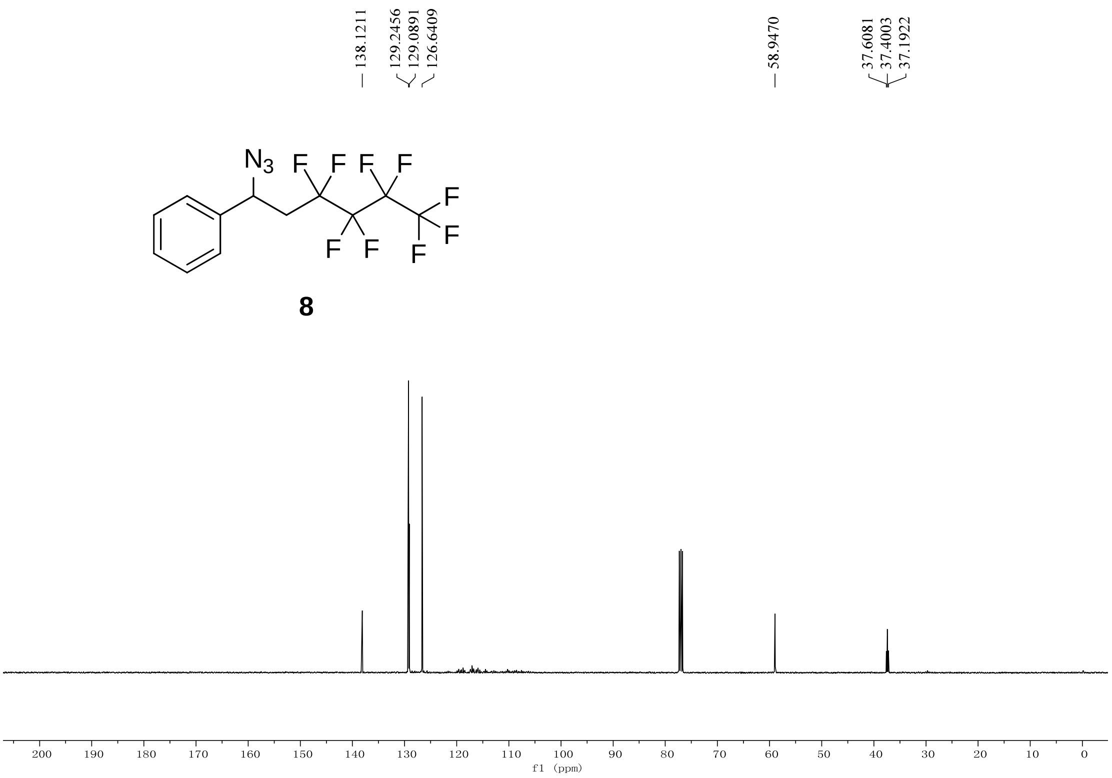

Supplementary Figure 16. ^{13}C NMR spectrum for compound **8**

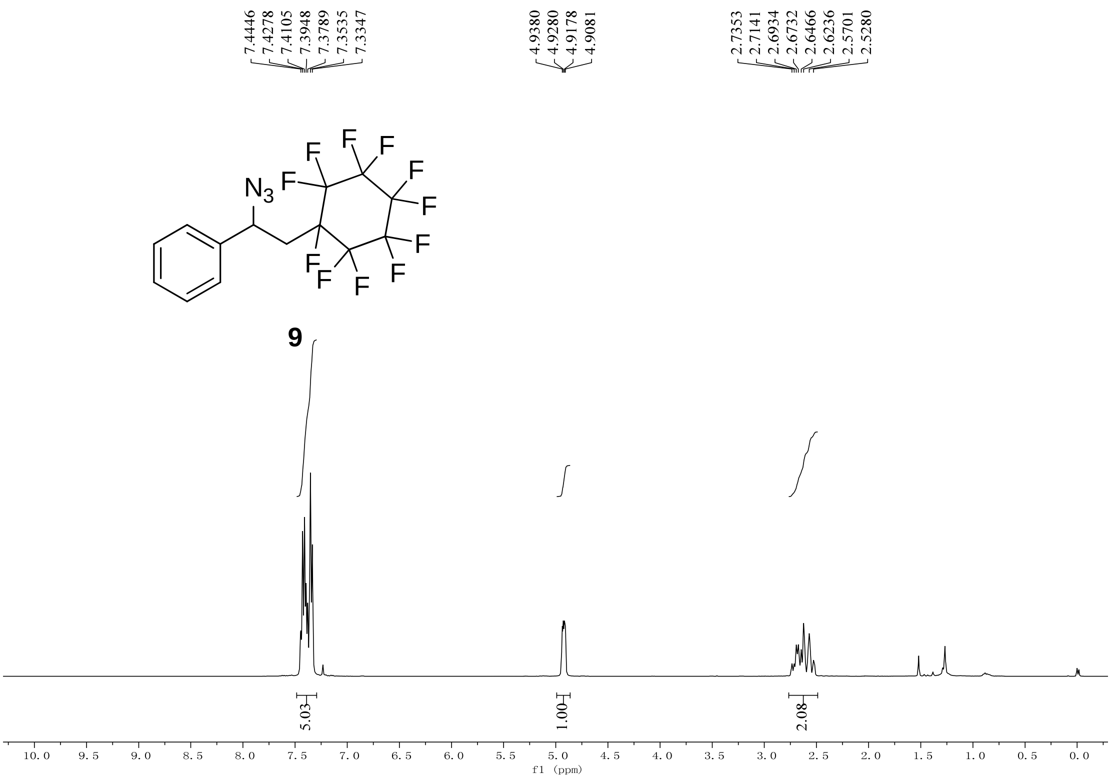

Supplementary Figure 17. ^{1}H NMR spectrum for compound **9**

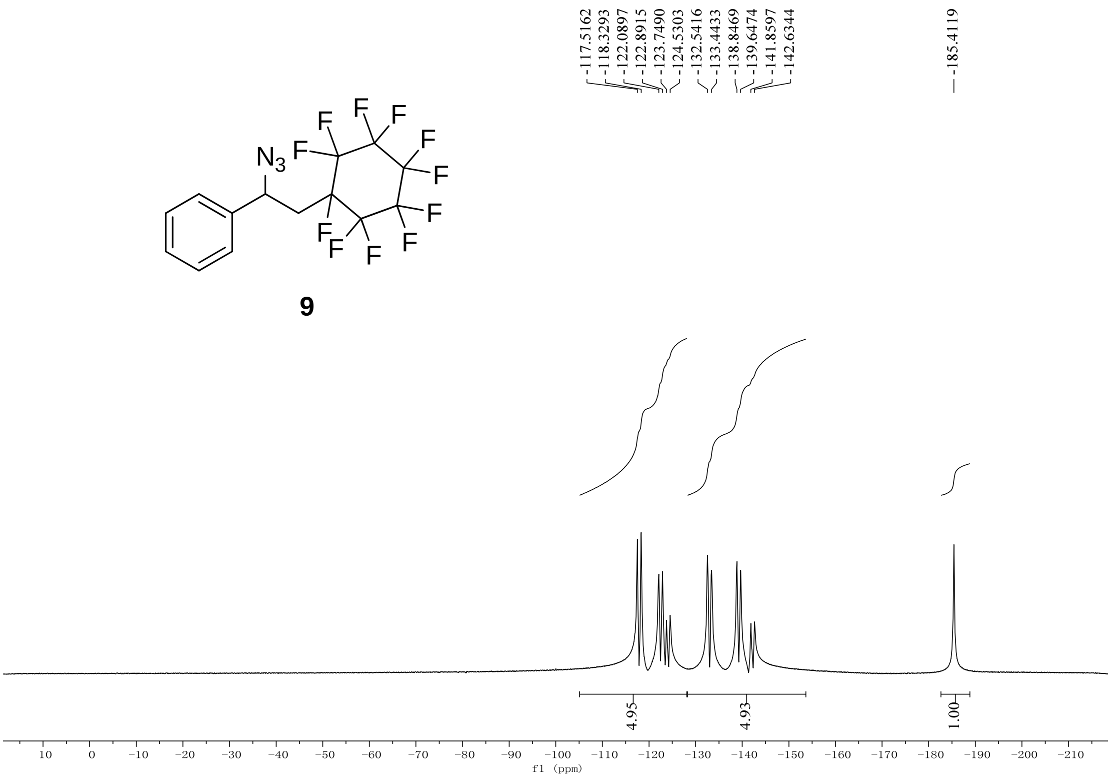

Supplementary Figure 18. ^{19}F NMR spectrum for compound **9**

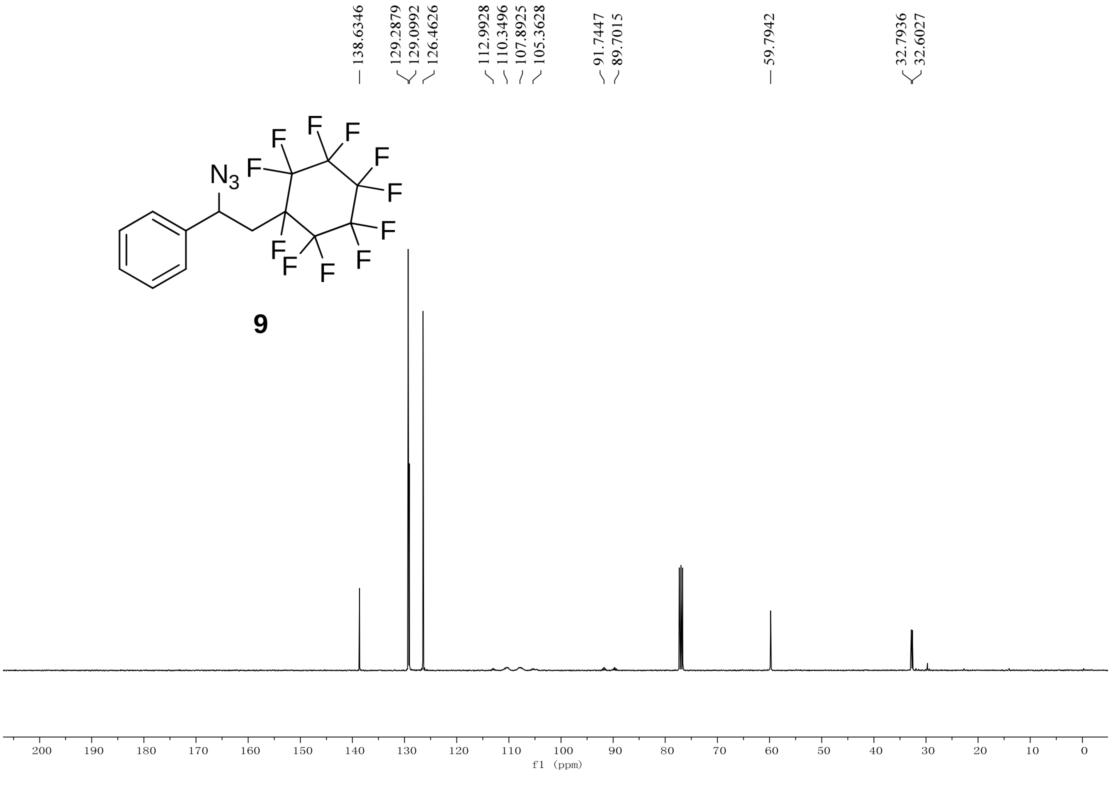

Supplementary Figure 19. ^{13}C NMR spectrum for compound **9**

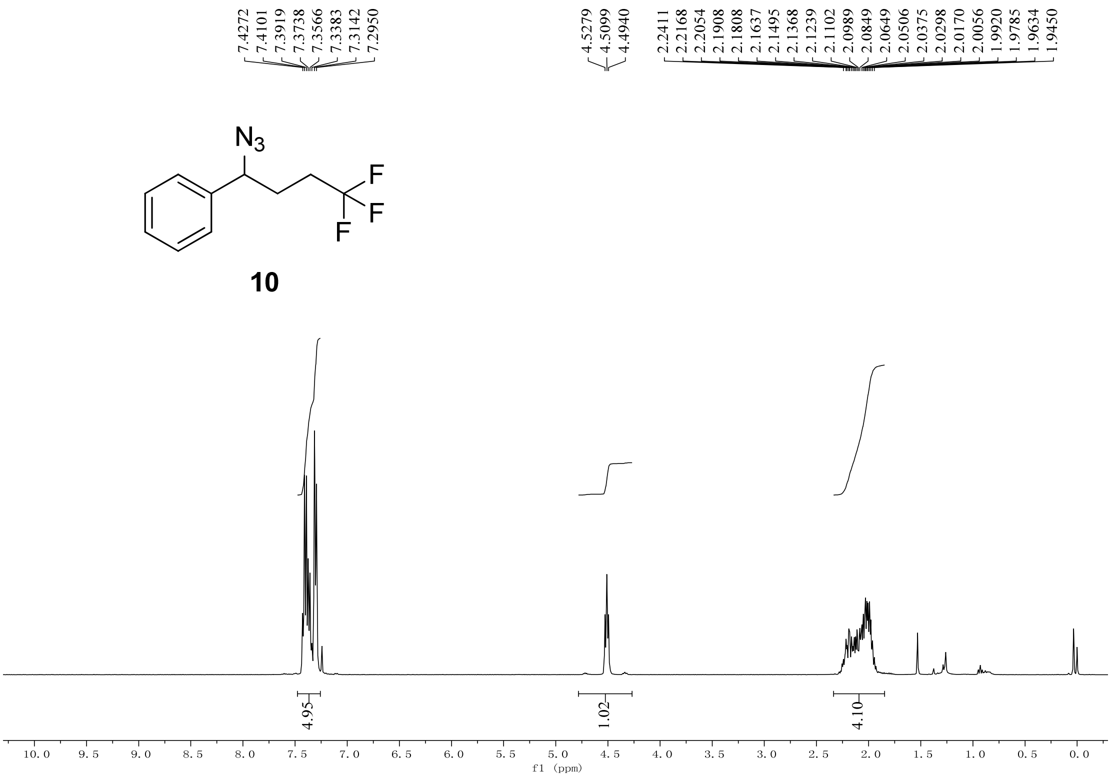

Supplementary Figure 20. ^{1}H NMR spectrum for compound **10**

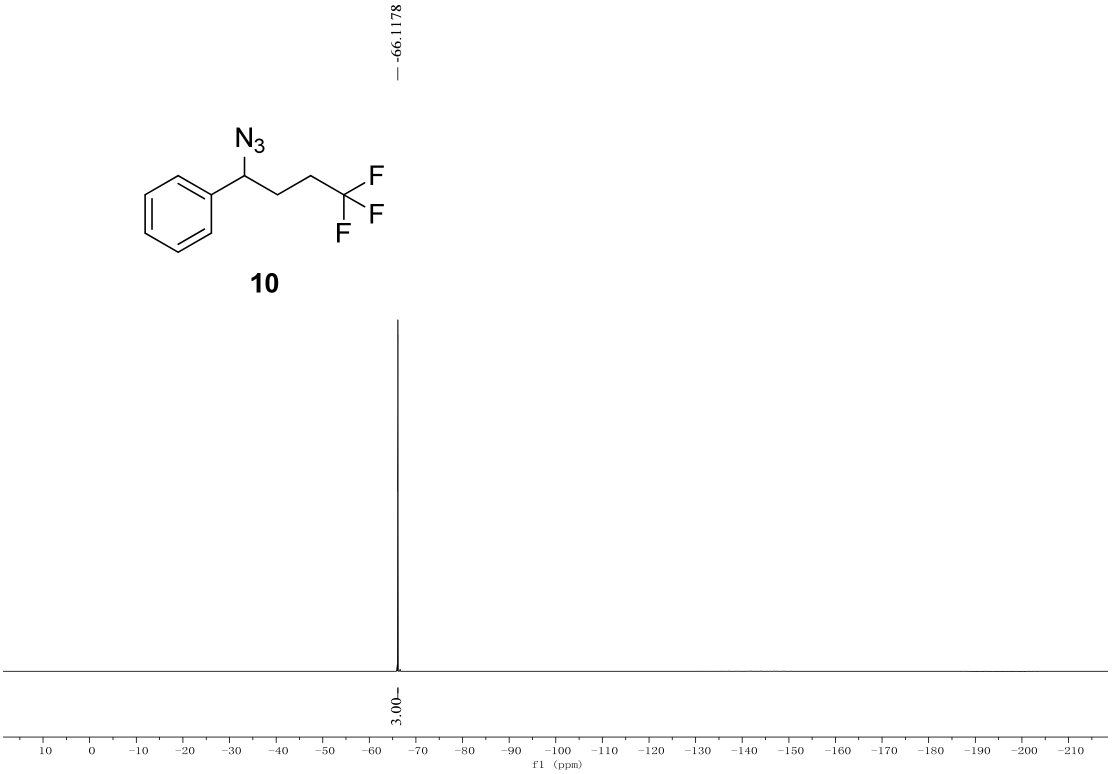

Supplementary Figure 21. ^{19}F NMR spectrum for compound **10**

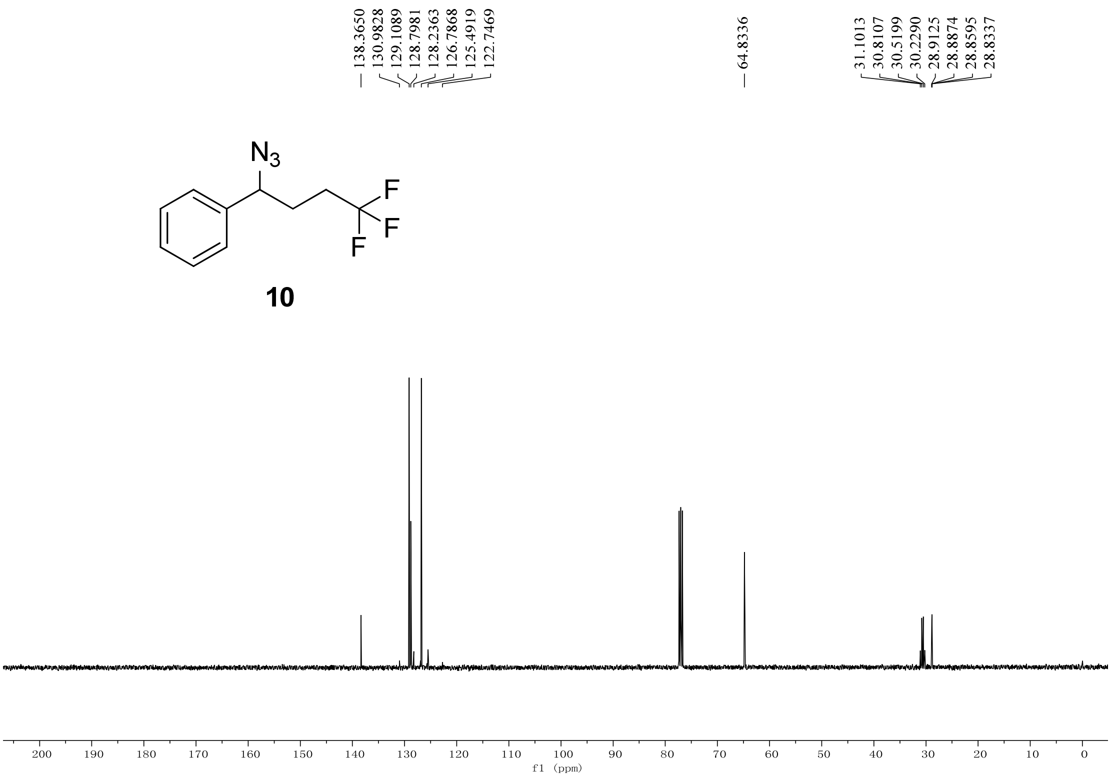

Supplementary Figure 22. ^{13}C NMR spectrum for compound **10**

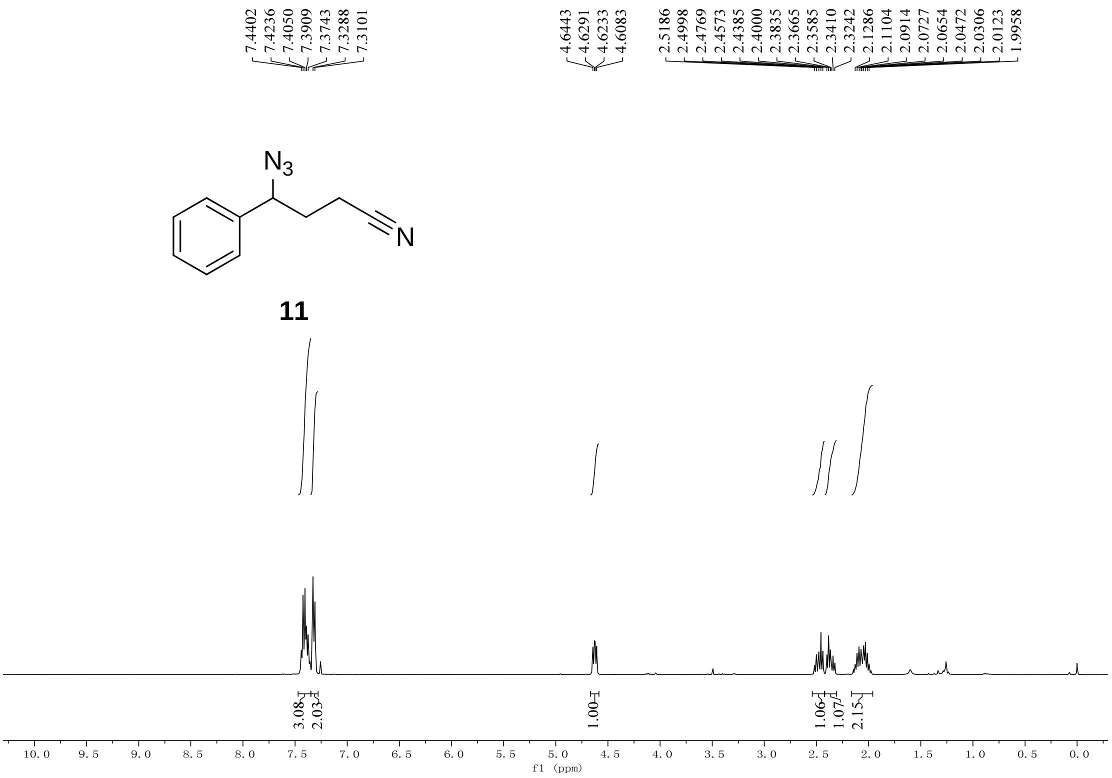

Supplementary Figure 23. ^{1}H NMR spectrum for compound **11**

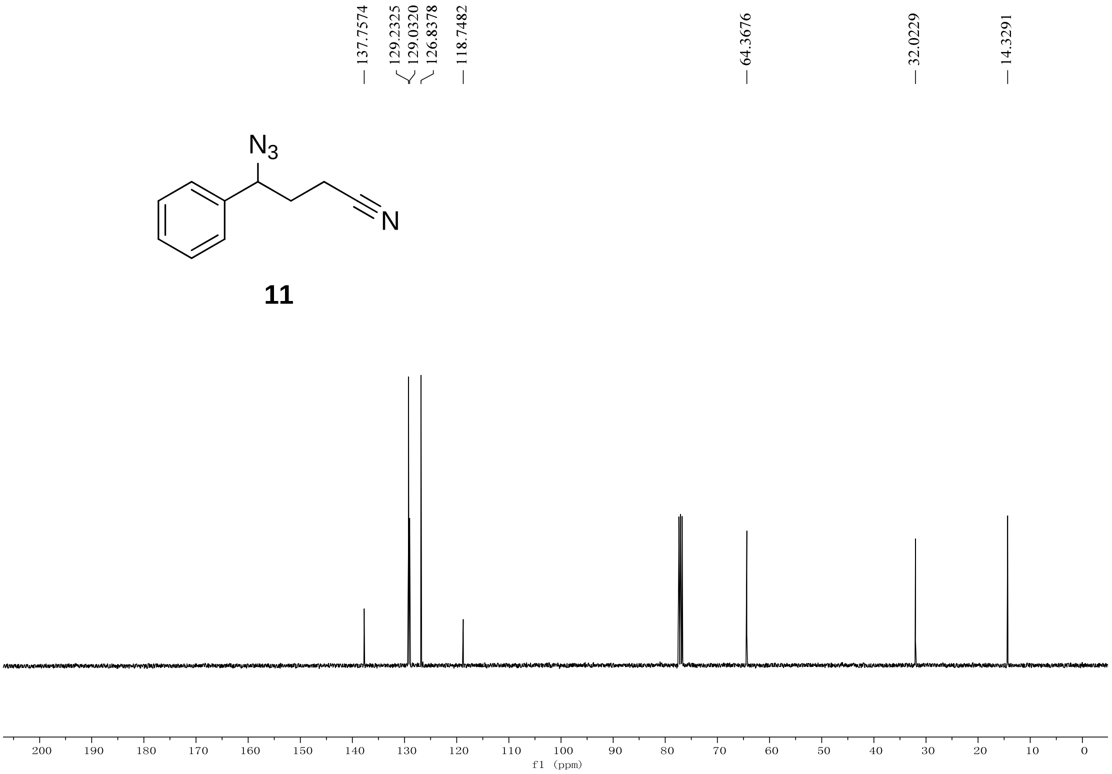

Supplementary Figure 24. ^{13}C NMR spectrum for compound **11**

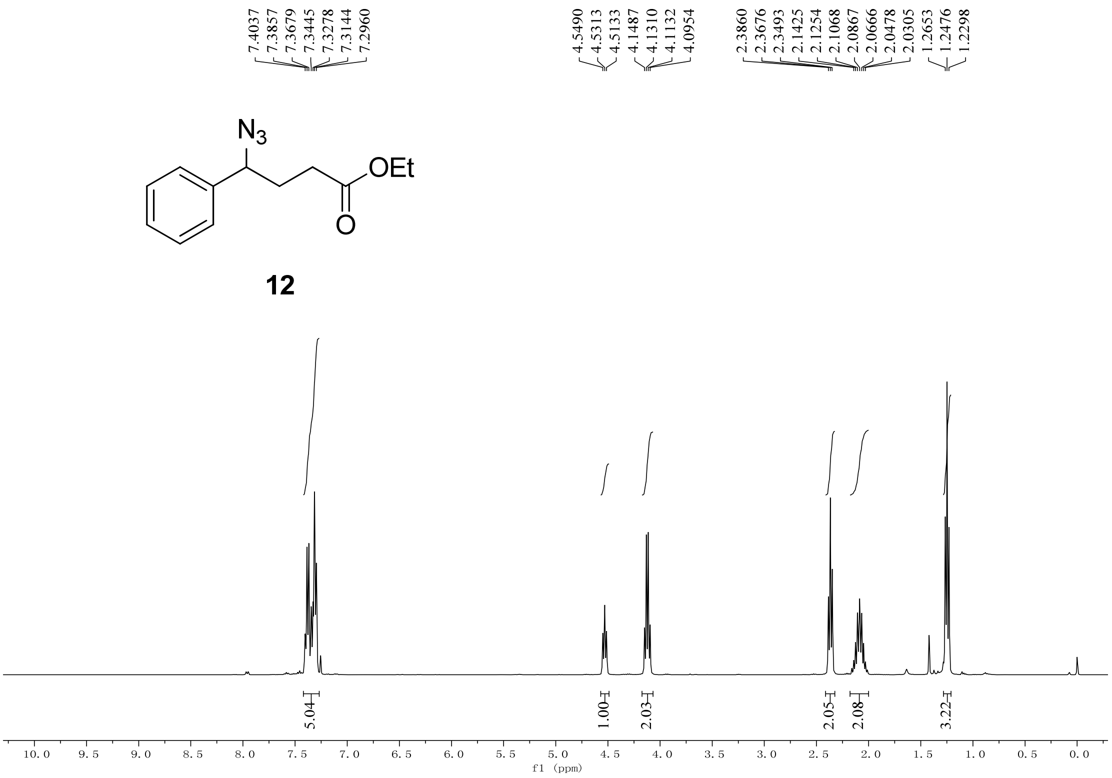

Supplementary Figure 25. ^{1}H NMR spectrum for compound **12**

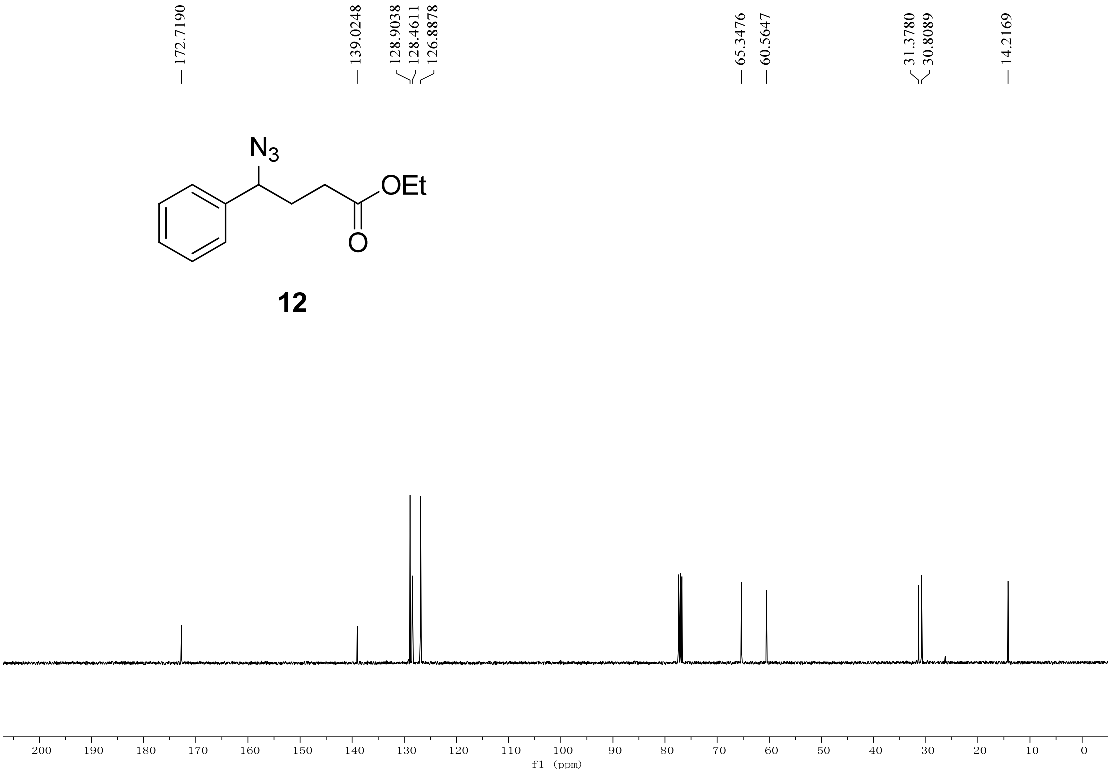

Supplementary Figure 26. ^{13}C NMR spectrum for compound **12**

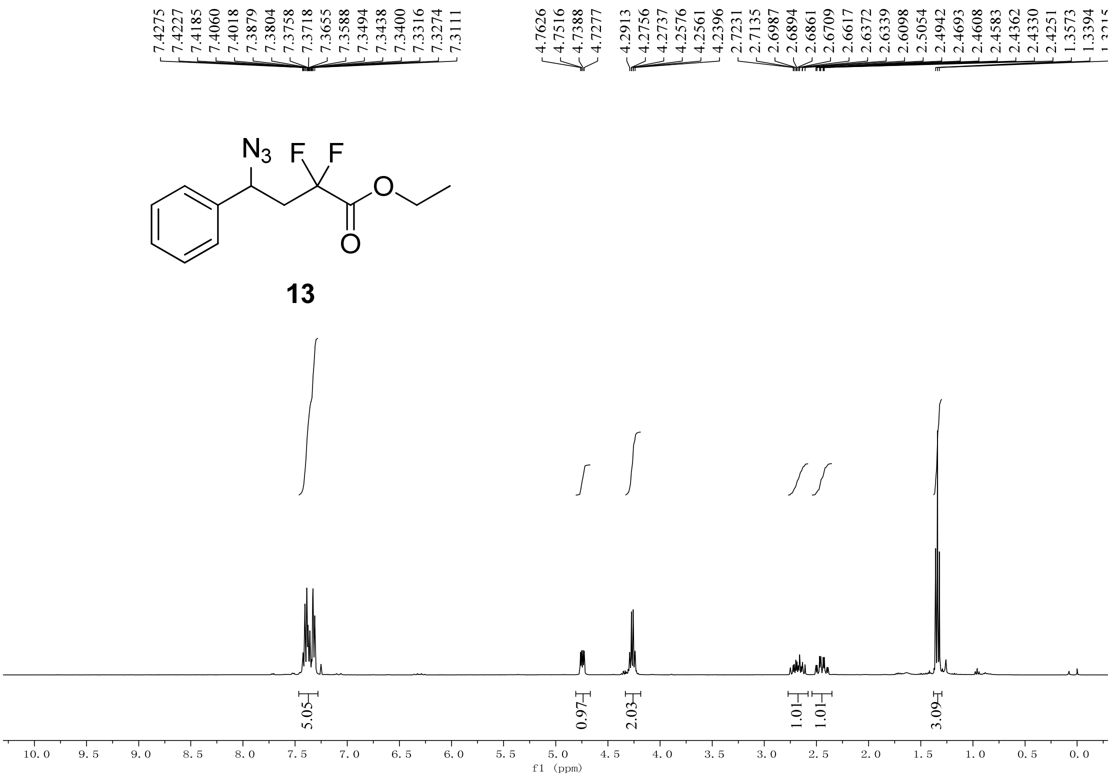

Supplementary Figure 27. ^{1}H NMR spectrum for compound **13**

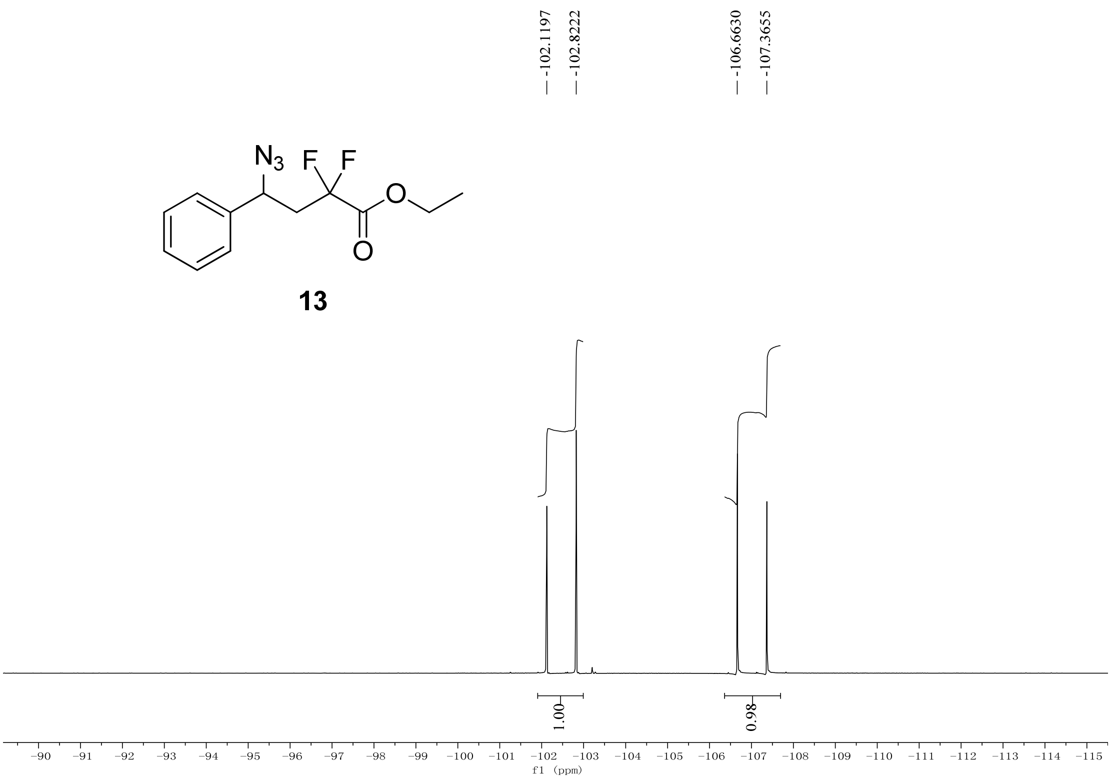

Supplementary Figure 28. ^{19}F NMR spectrum for compound **13**

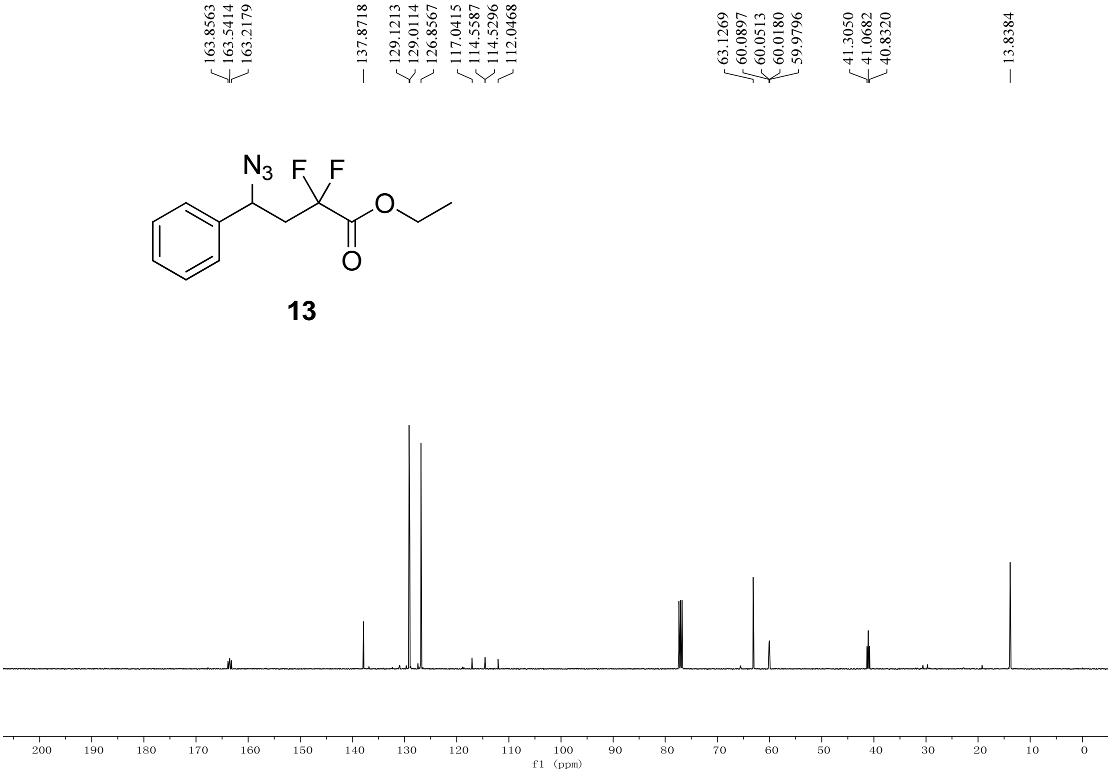

Supplementary Figure 29. ^{13}C NMR spectrum for compound **13**

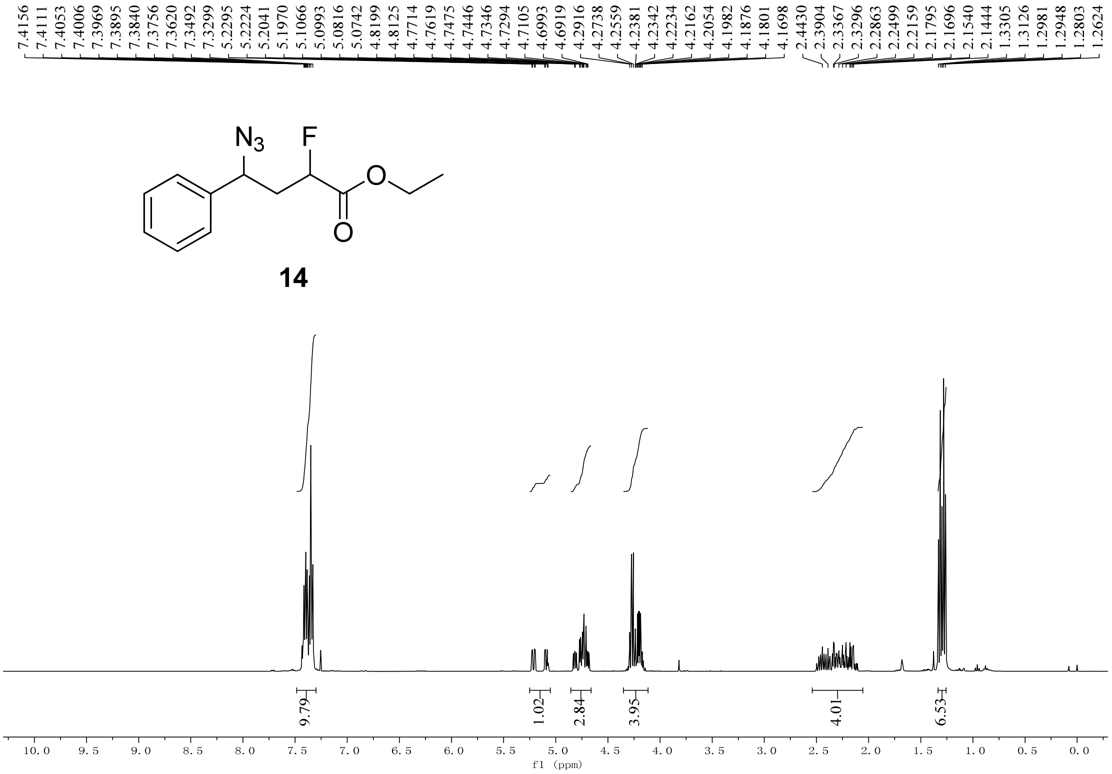

Supplementary Figure 30. ^{1}H NMR spectrum for compound **14**

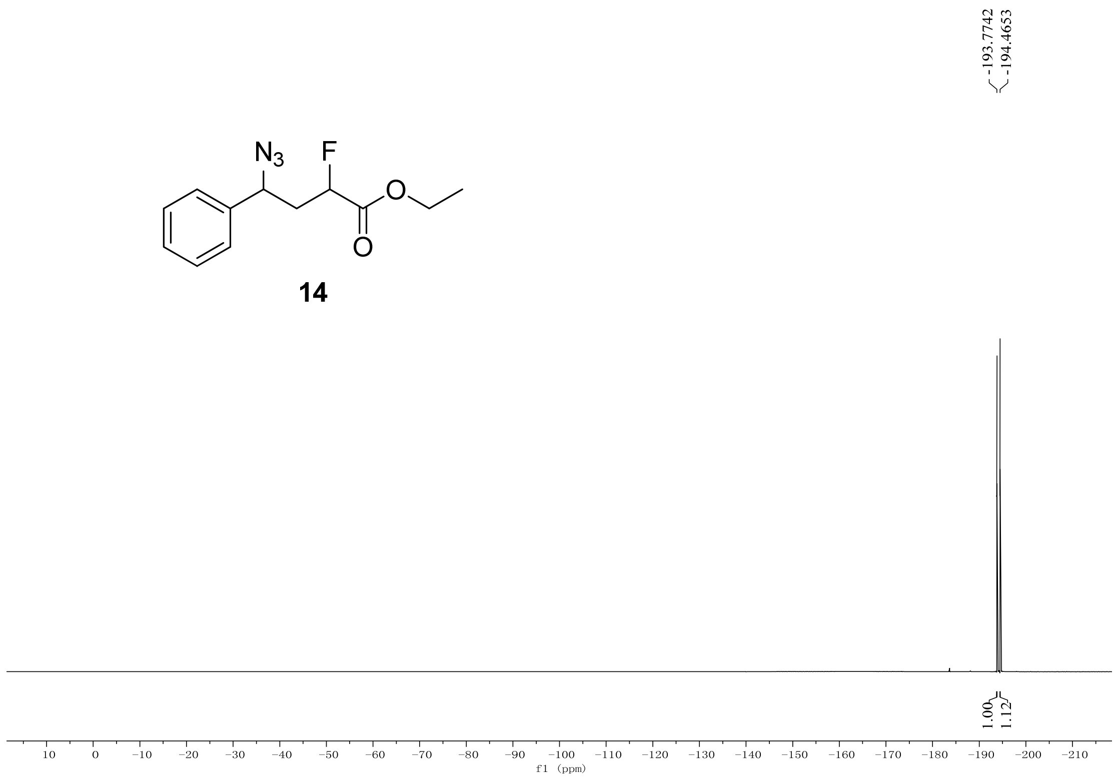

Supplementary Figure 31. ^{19}F NMR spectrum for compound **14**

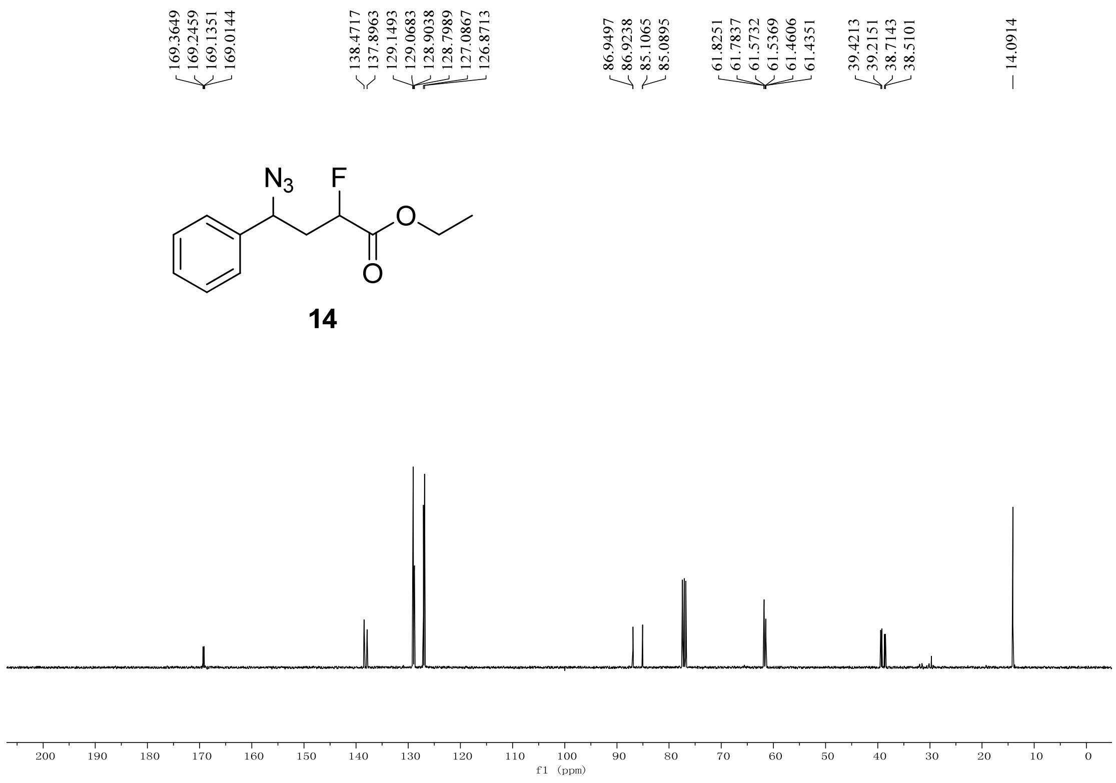

Supplementary Figure 32. ^{13}C NMR spectrum for compound **14**

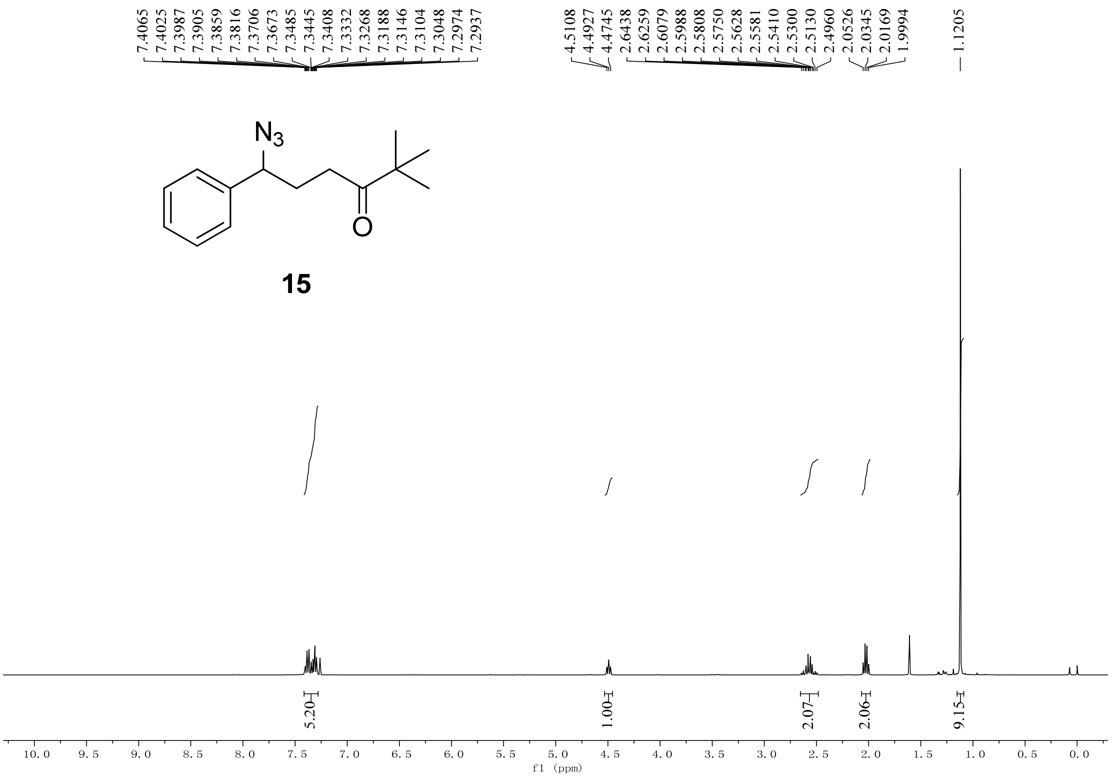

Supplementary Figure 33. ^{1}H NMR spectrum for compound **15**

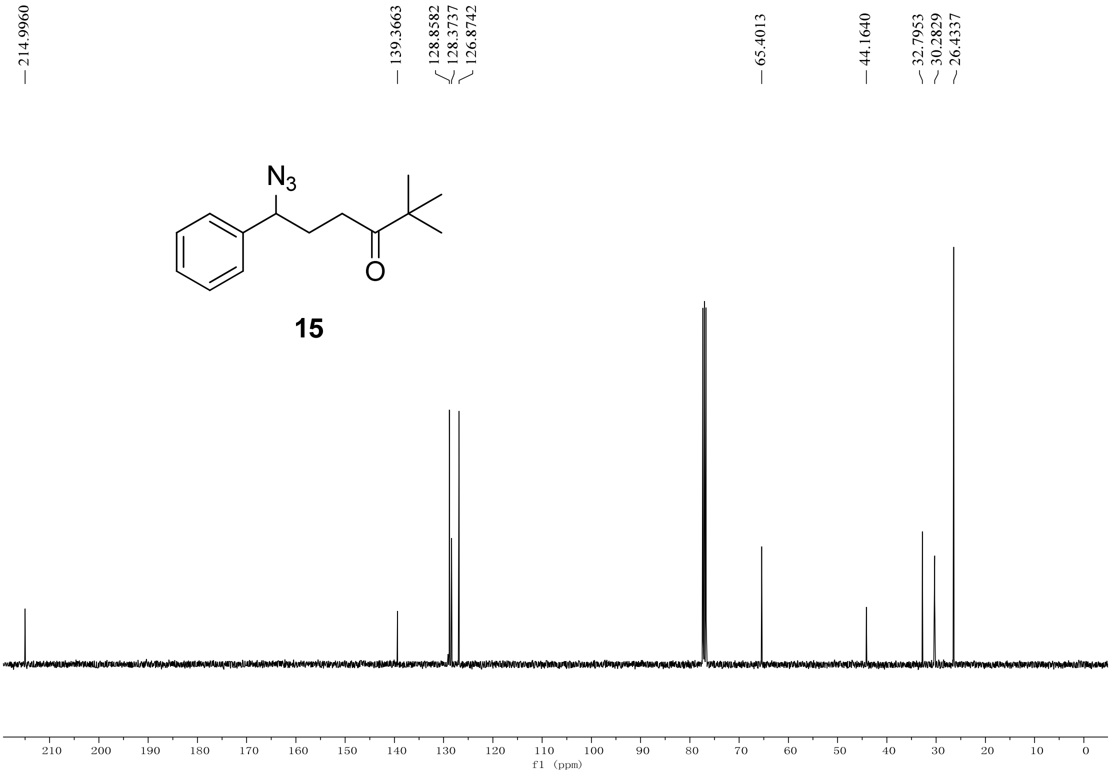

Supplementary Figure 34. ^{13}C NMR spectrum for compound **15**

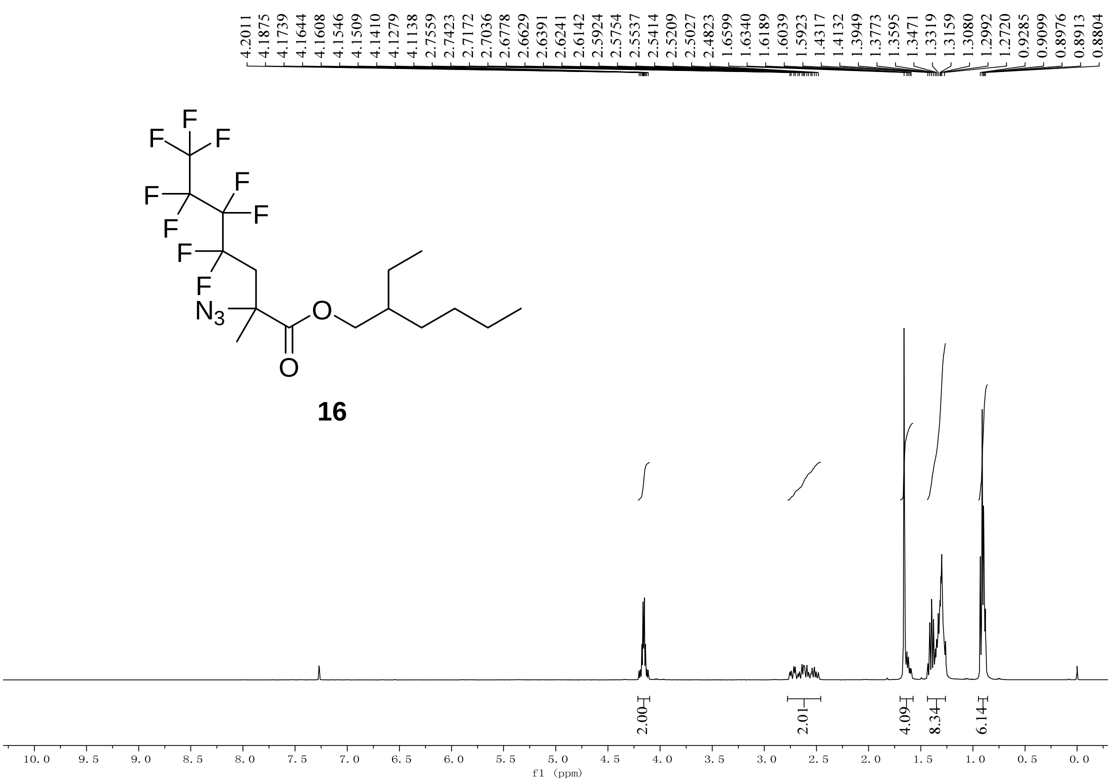

Supplementary Figure 35. ^{1}H NMR spectrum for compound **16**

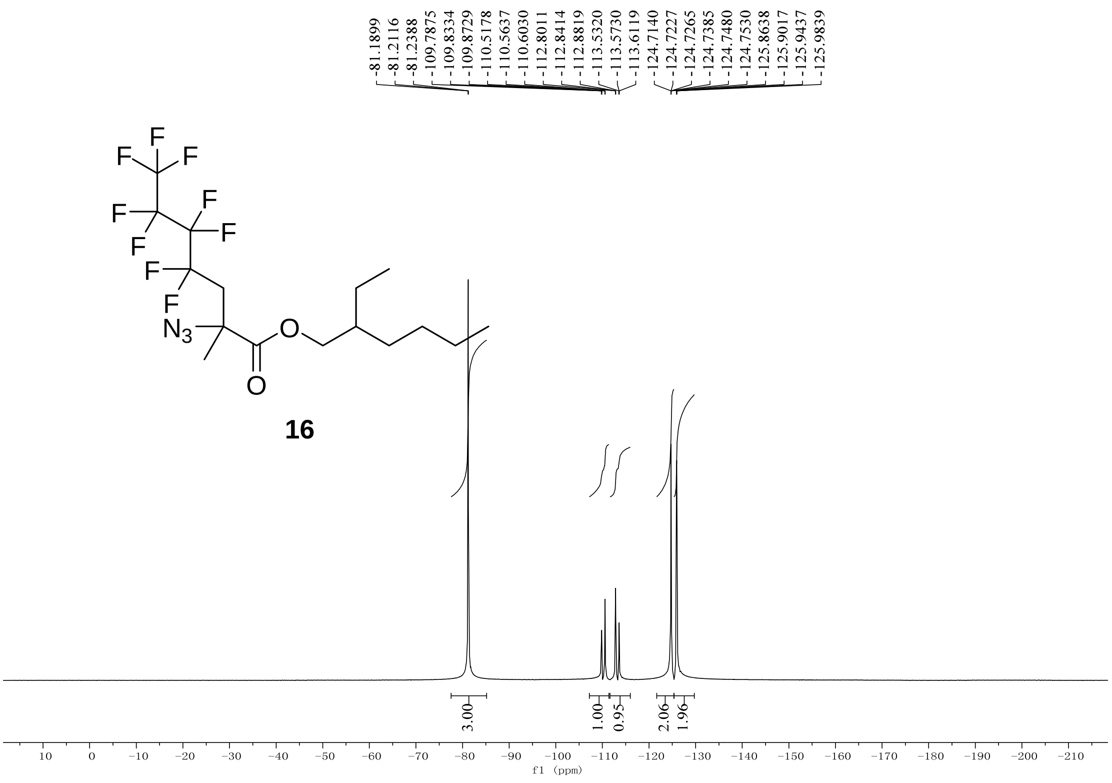

Supplementary Figure 36. ^{19}F NMR spectrum for compound **16**

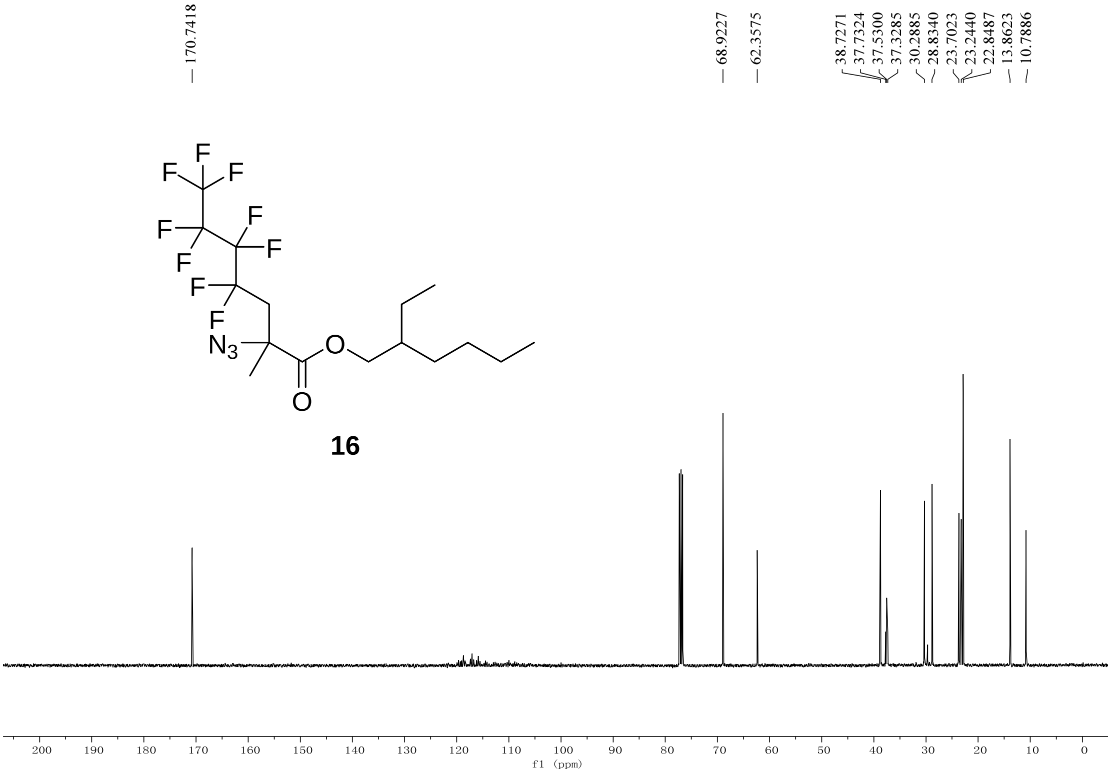

Supplementary Figure 37. ^{13}C NMR spectrum for compound **16**

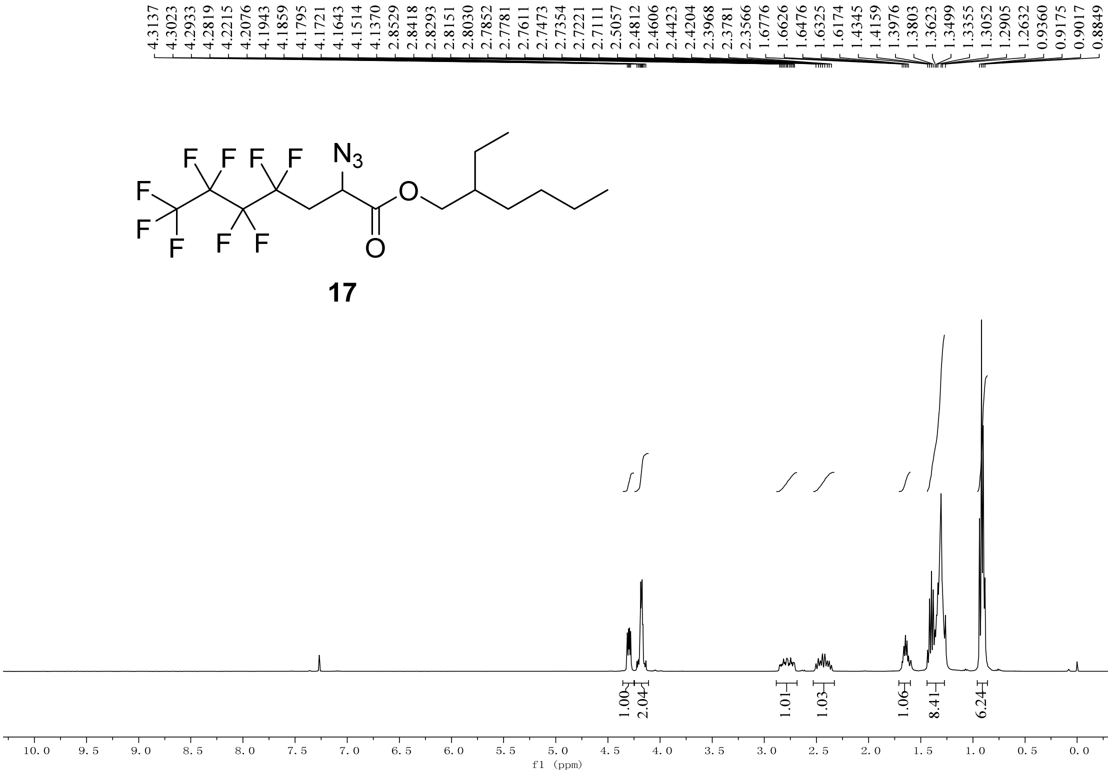

Supplementary Figure 38. ^{1}H NMR spectrum for compound **17**

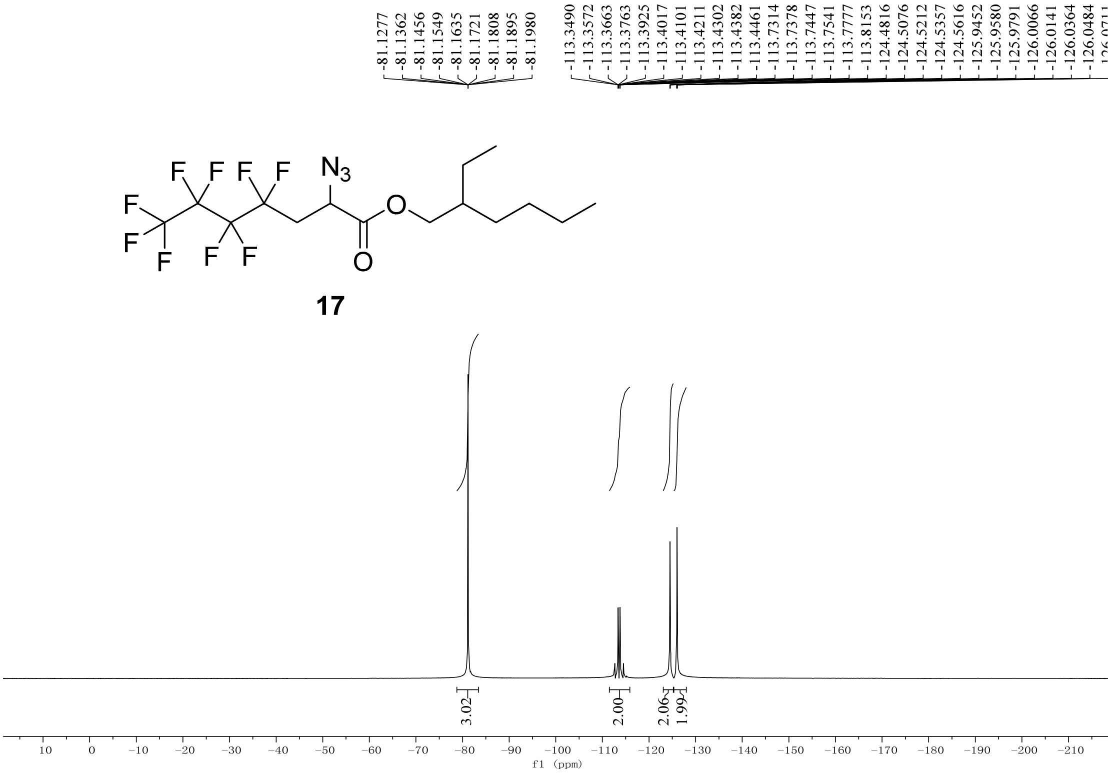

Supplementary Figure 39. ^{19}F NMR spectrum for compound **17**

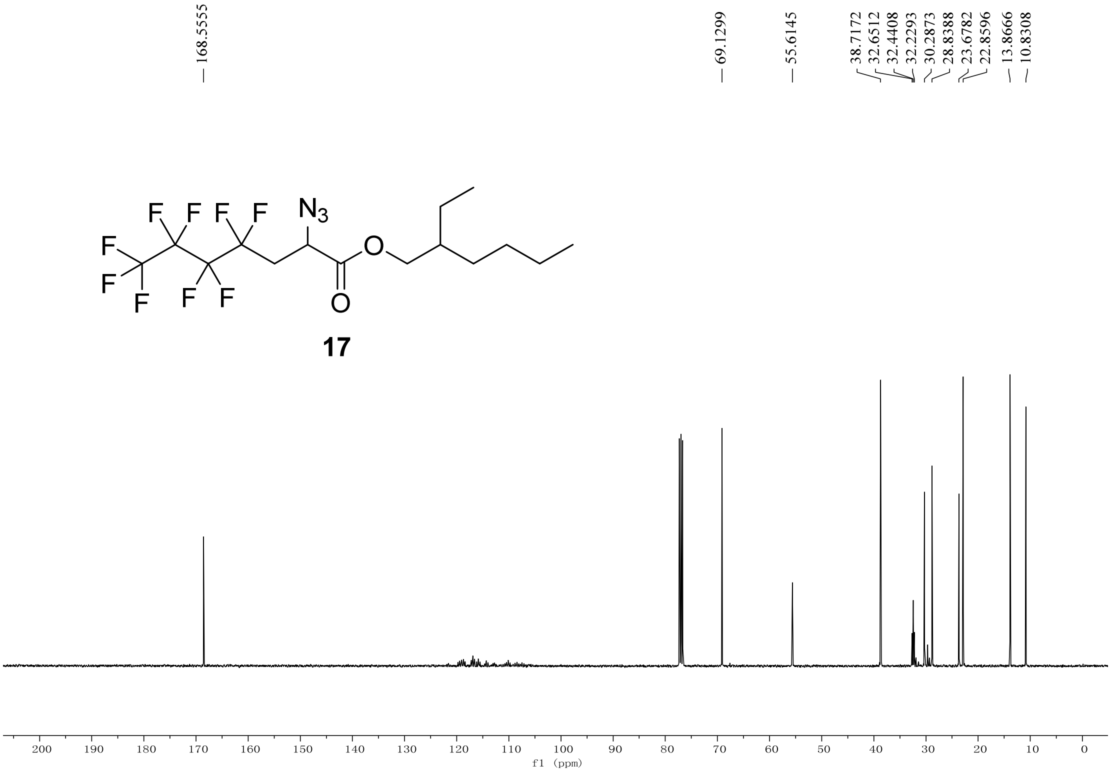

Supplementary Figure 40. ^{13}C NMR spectrum for compound **17**

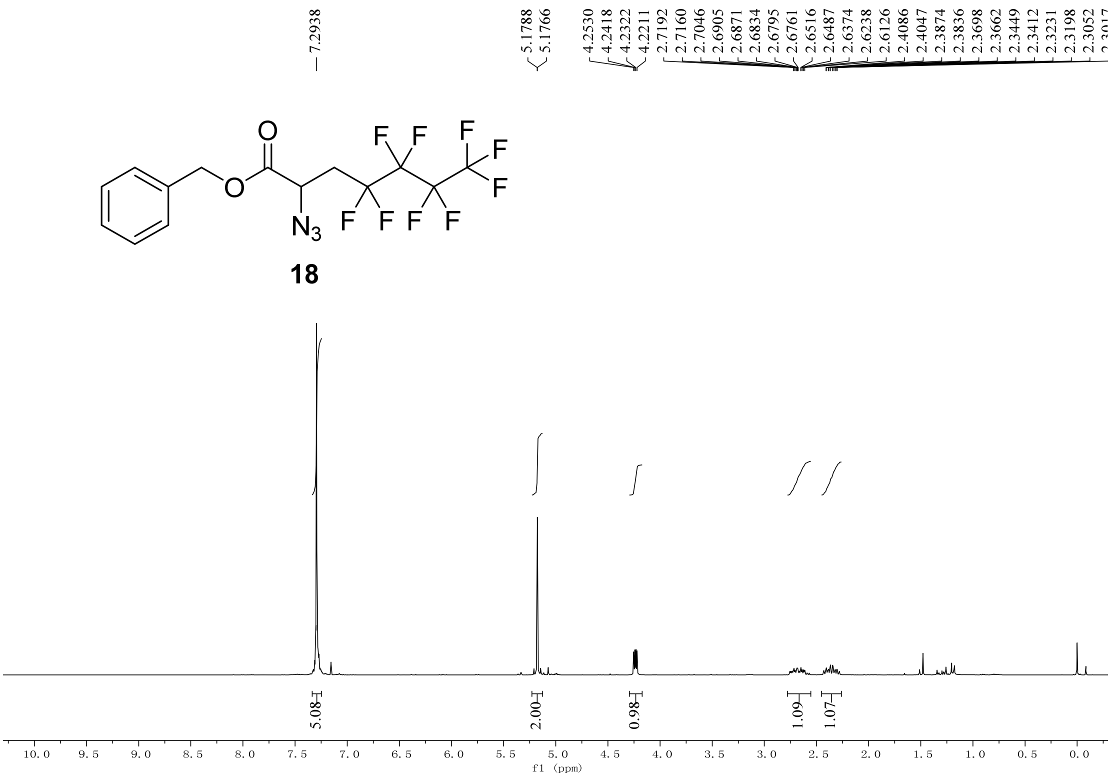

Supplementary Figure 41. ^{1}H NMR spectrum for compound **18**

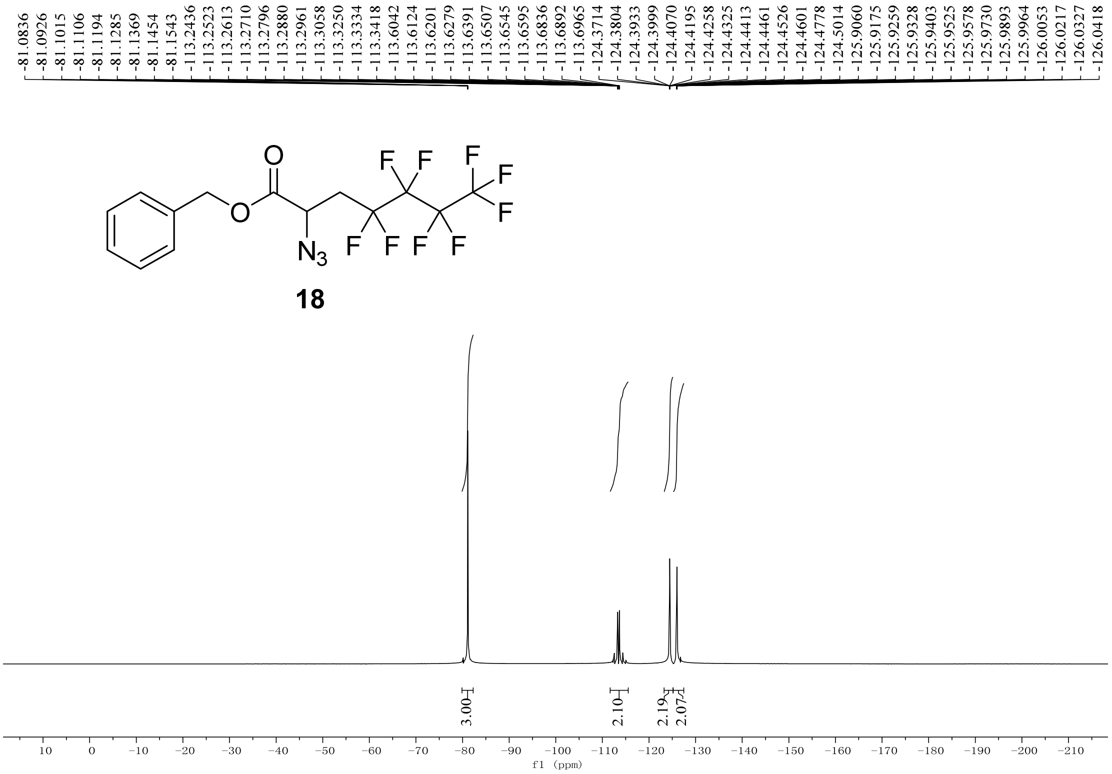

Supplementary Figure 42. ^{19}F NMR spectrum for compound **18**

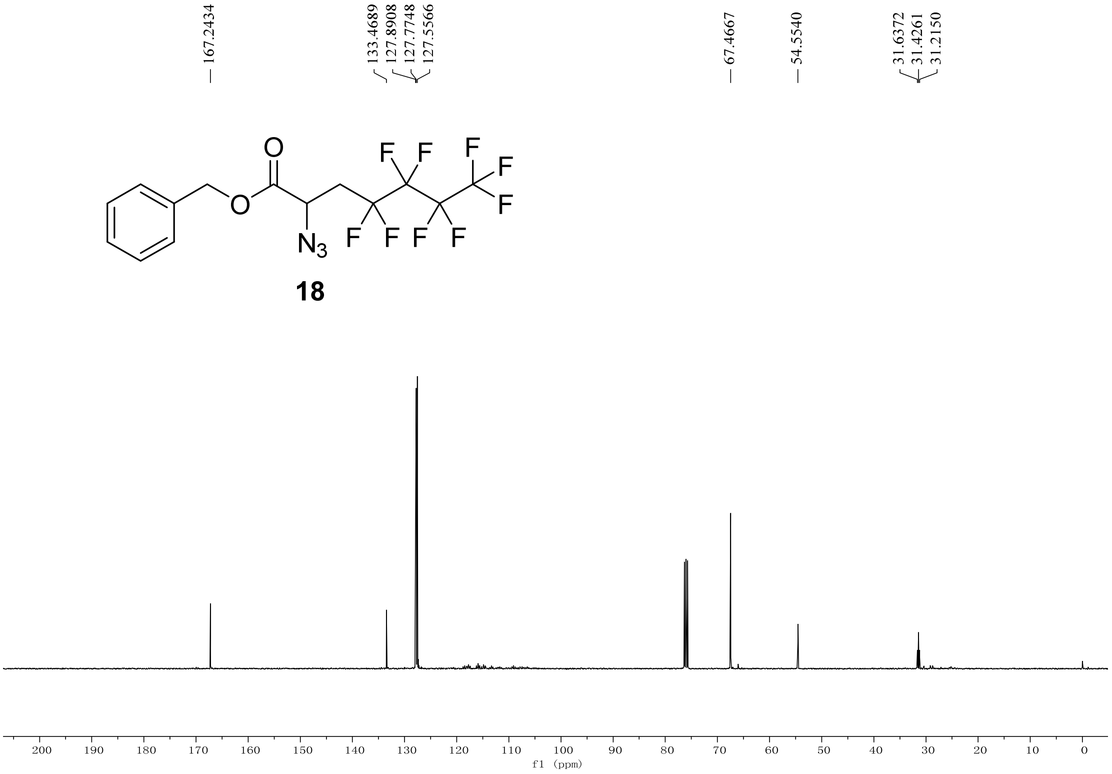

Supplementary Figure 43. ^{13}C NMR spectrum for compound **18**

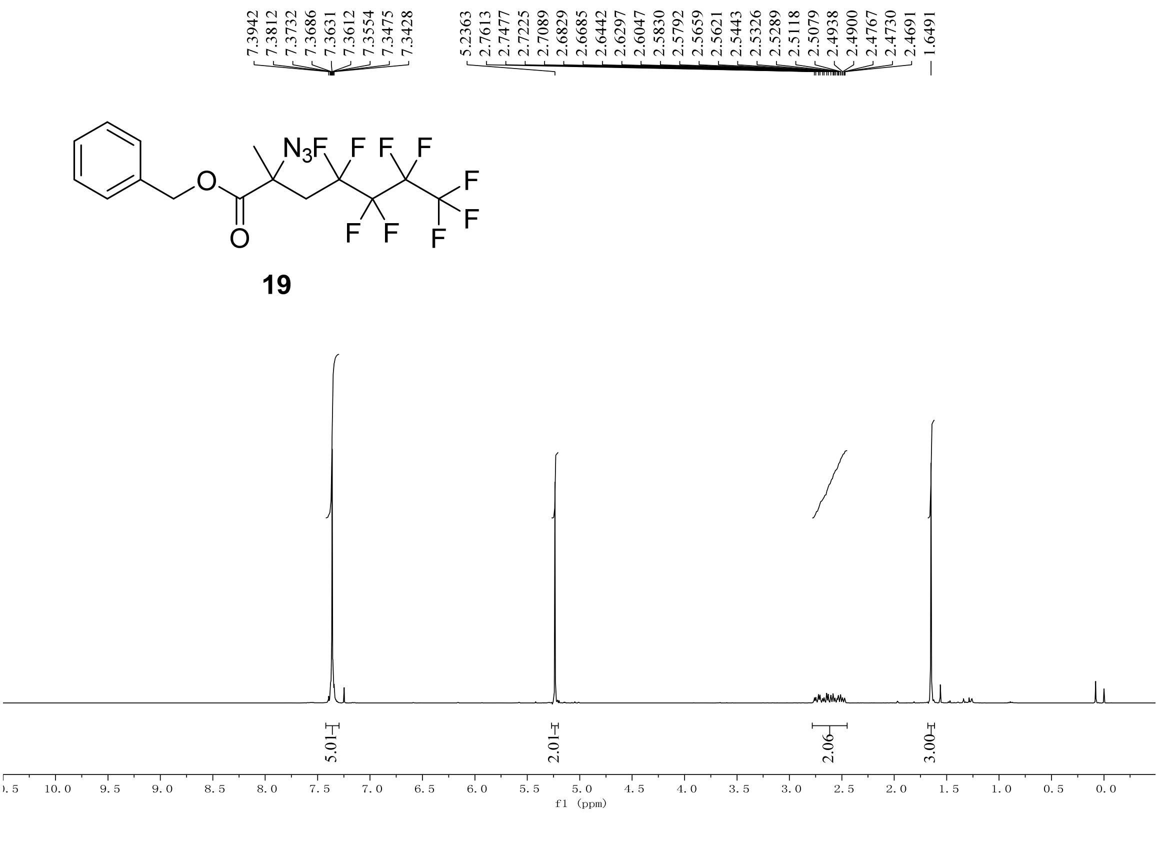

Supplementary Figure 44. ^{1}H NMR spectrum for compound **19**

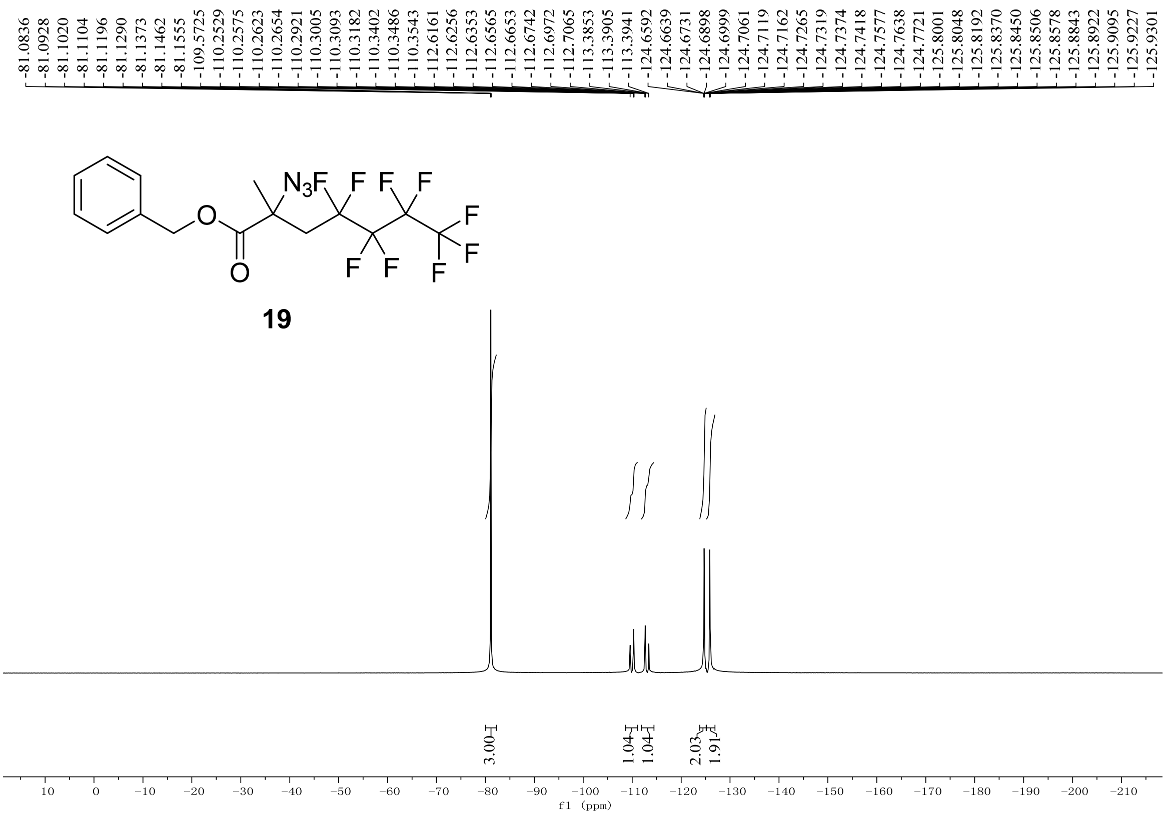

Supplementary Figure 45. ^{19}F NMR spectrum for compound **19**

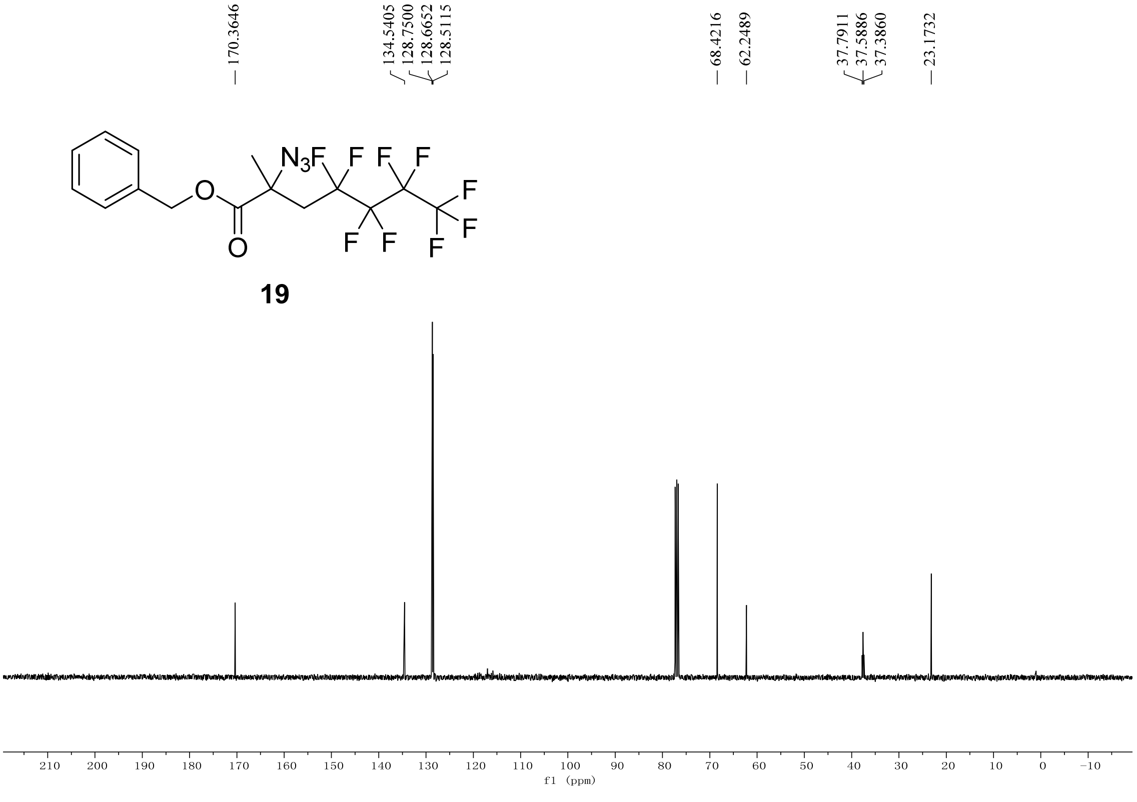

Supplementary Figure 46. ^{13}C NMR spectrum for compound **19**

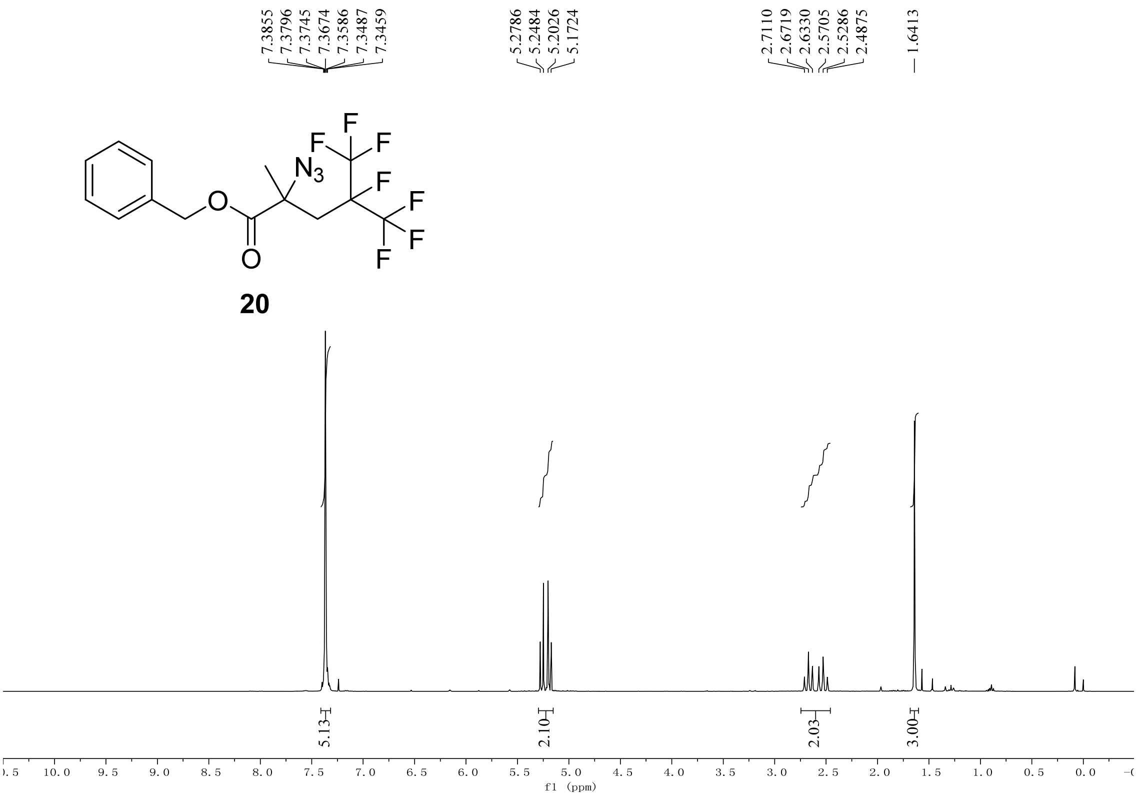

Supplementary Figure 47. ^{1}H NMR spectrum for compound **20**

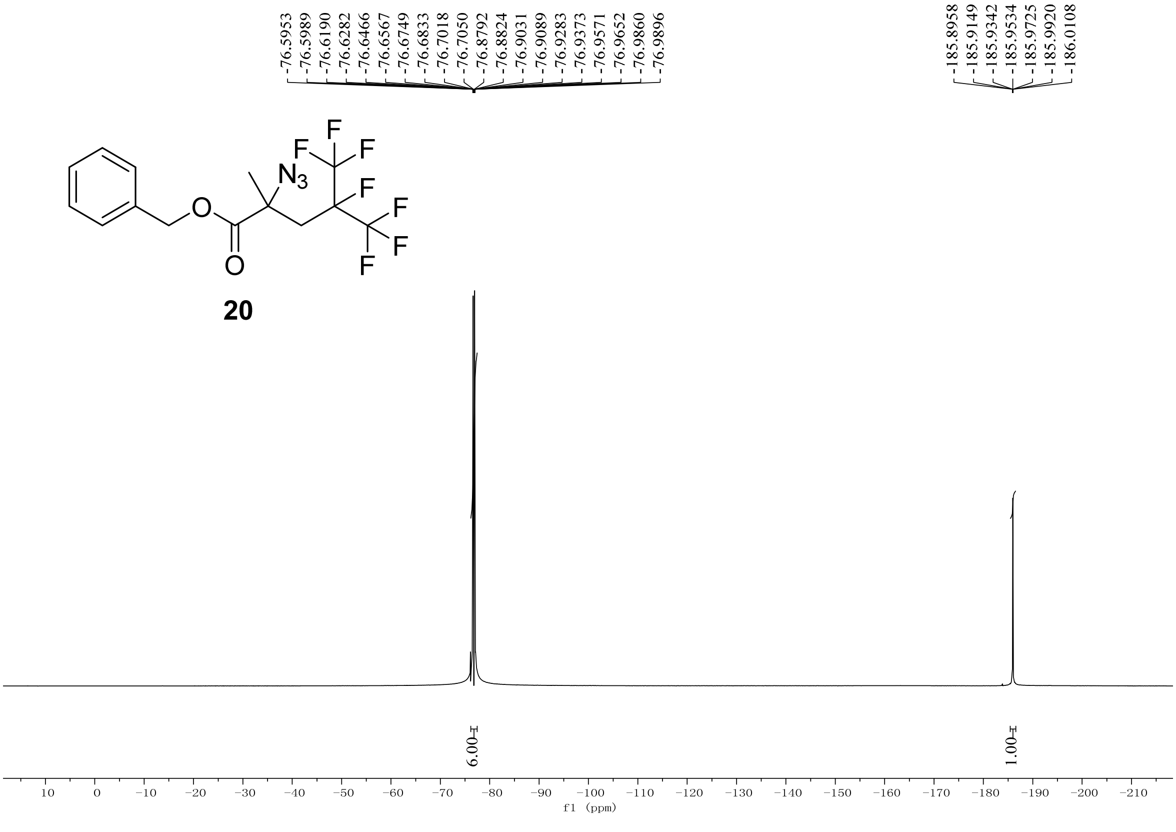

Supplementary Figure 48. ^{19}F NMR spectrum for compound **20**

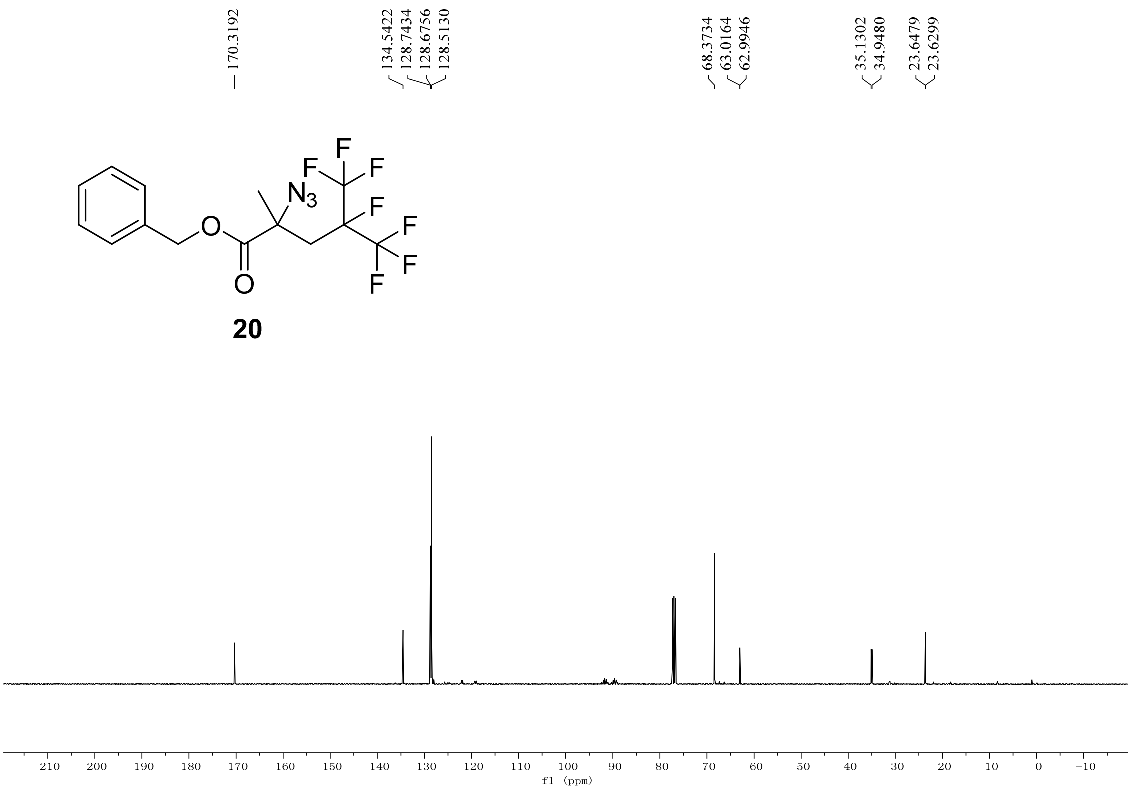

Supplementary Figure 49. ^{13}C NMR spectrum for compound **20**

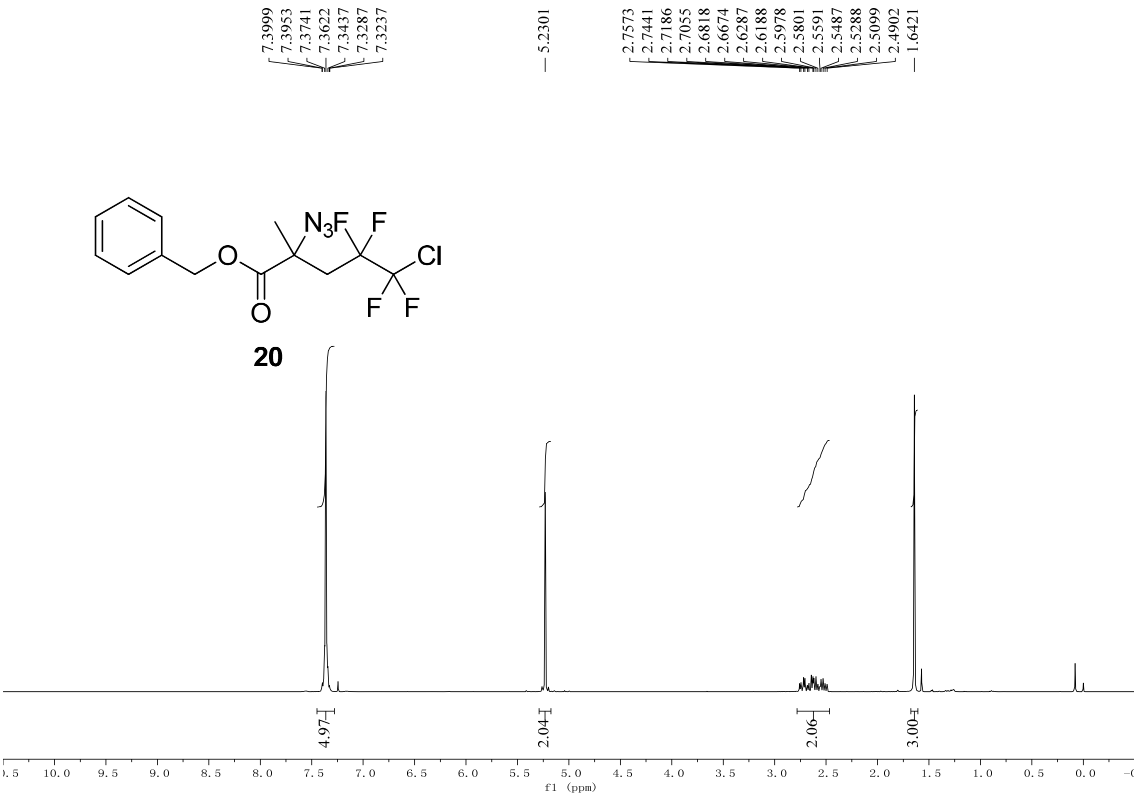

Supplementary Figure 50. ^{1}H NMR spectrum for compound **21**

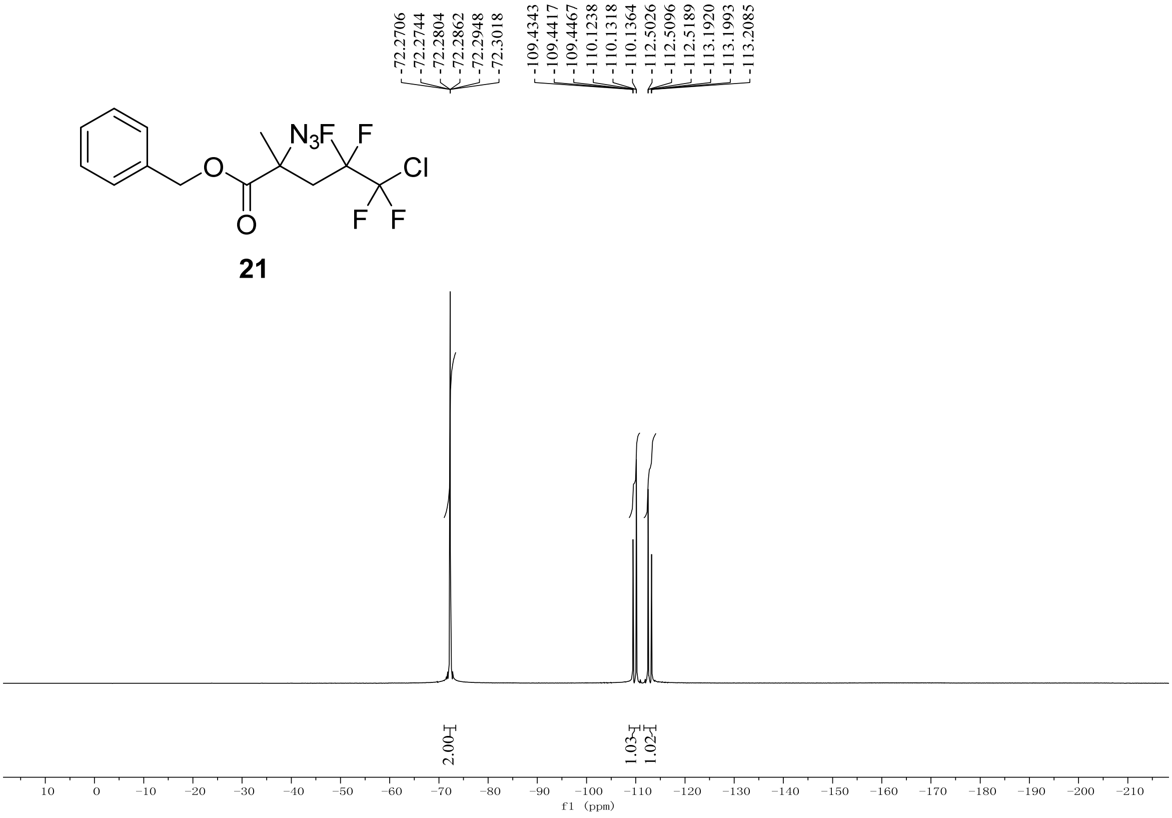

Supplementary Figure 51. ^{19}F NMR spectrum for compound **21**

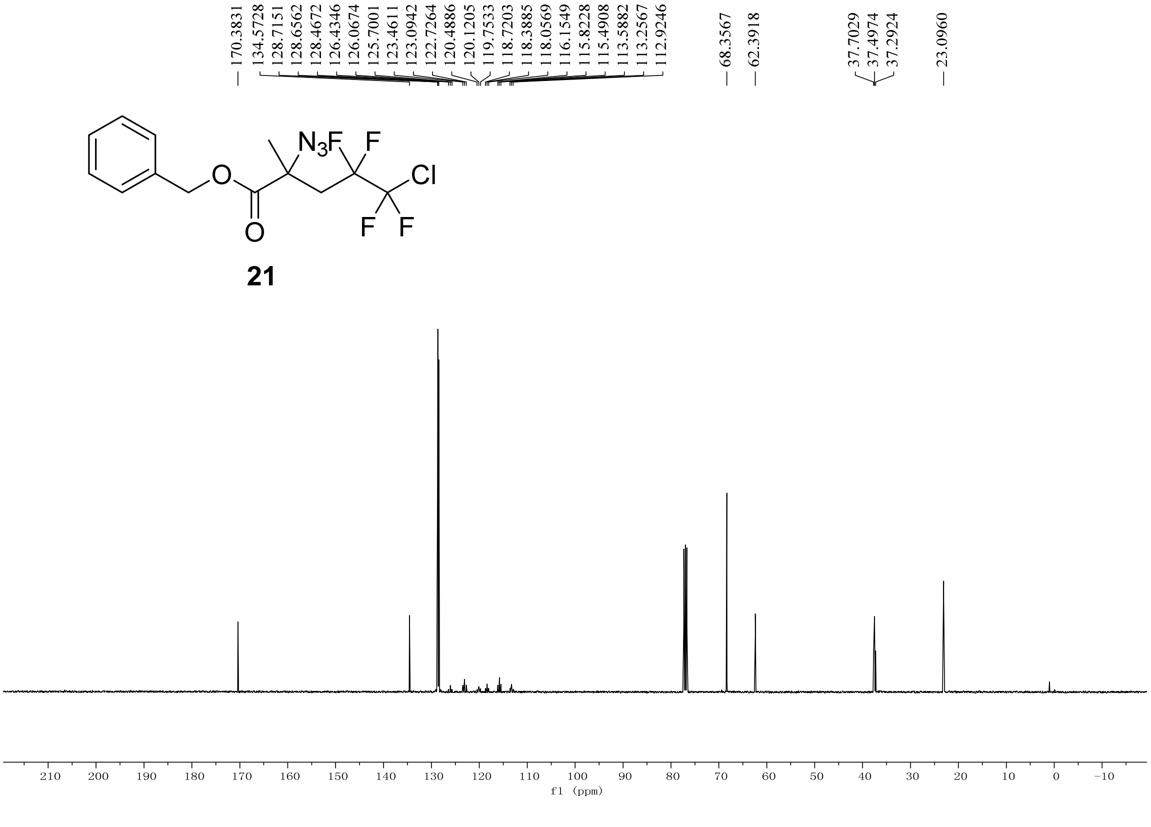

Supplementary Figure 52. ^{13}C NMR spectrum for compound **21**

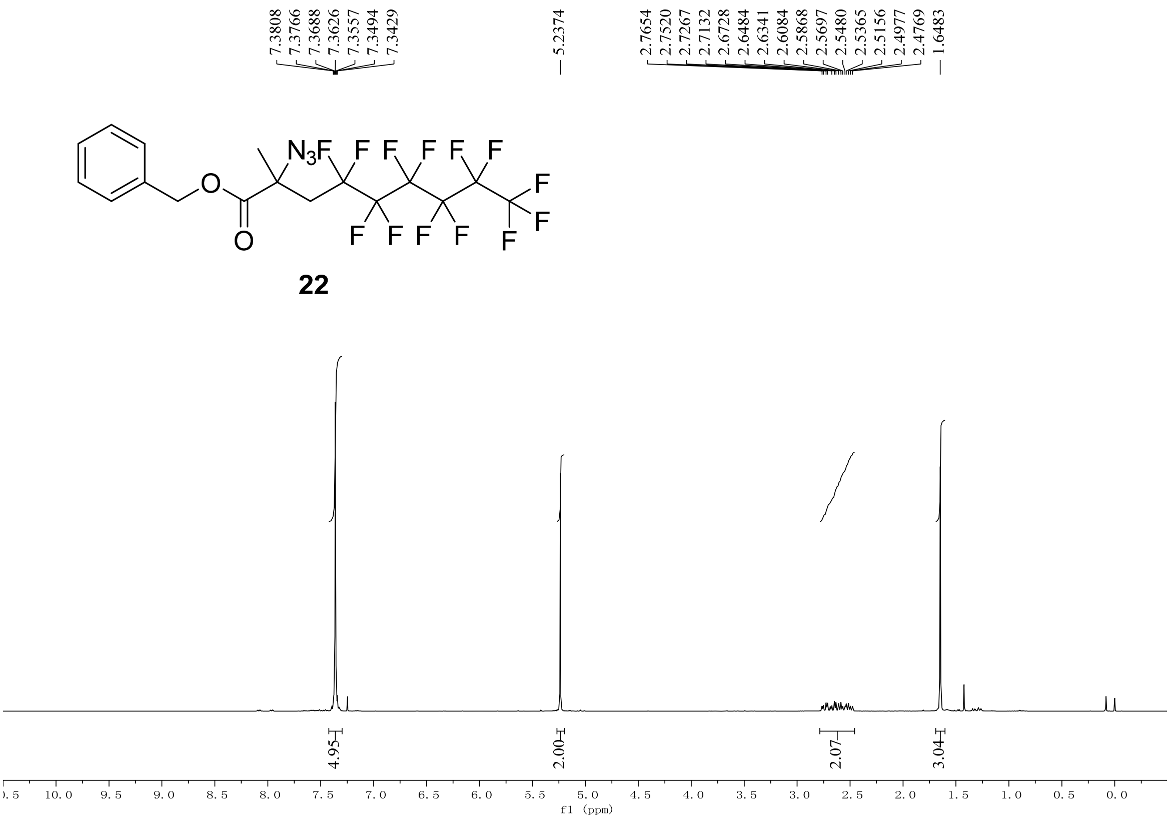

Supplementary Figure 53. ^{1}H NMR spectrum for compound **22**

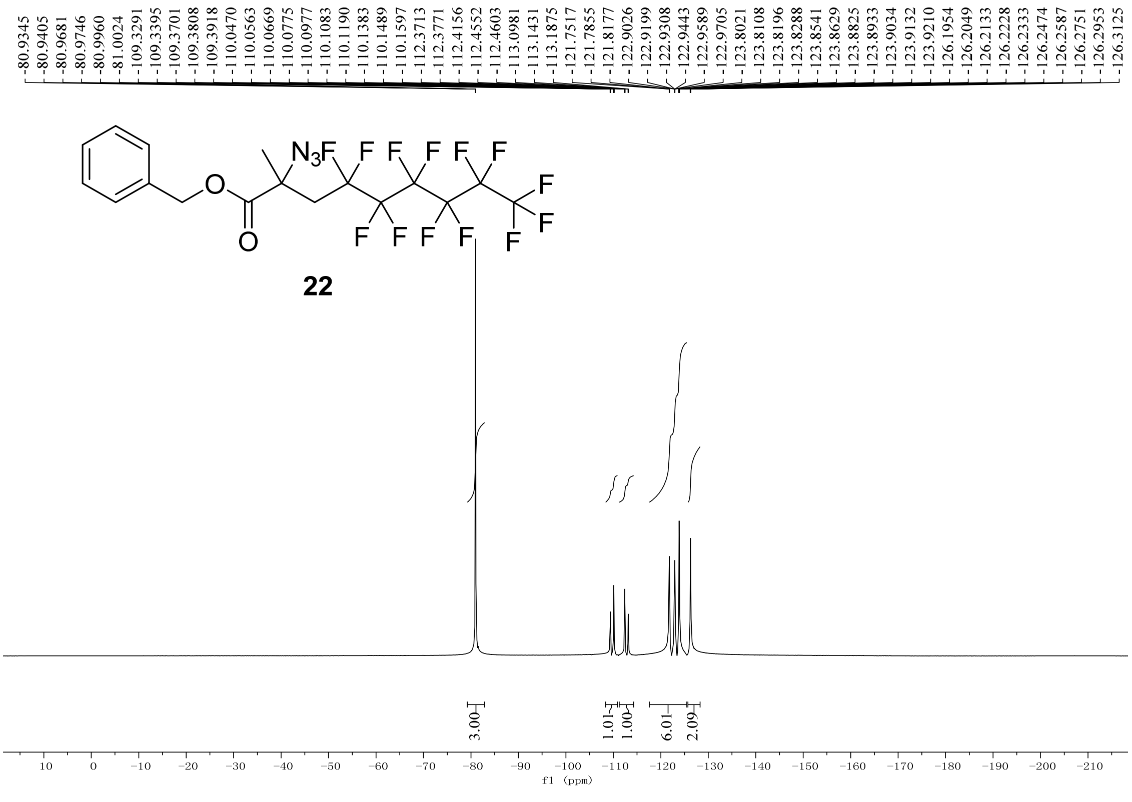

Supplementary Figure 54. ^{19}F NMR spectrum for compound **22**

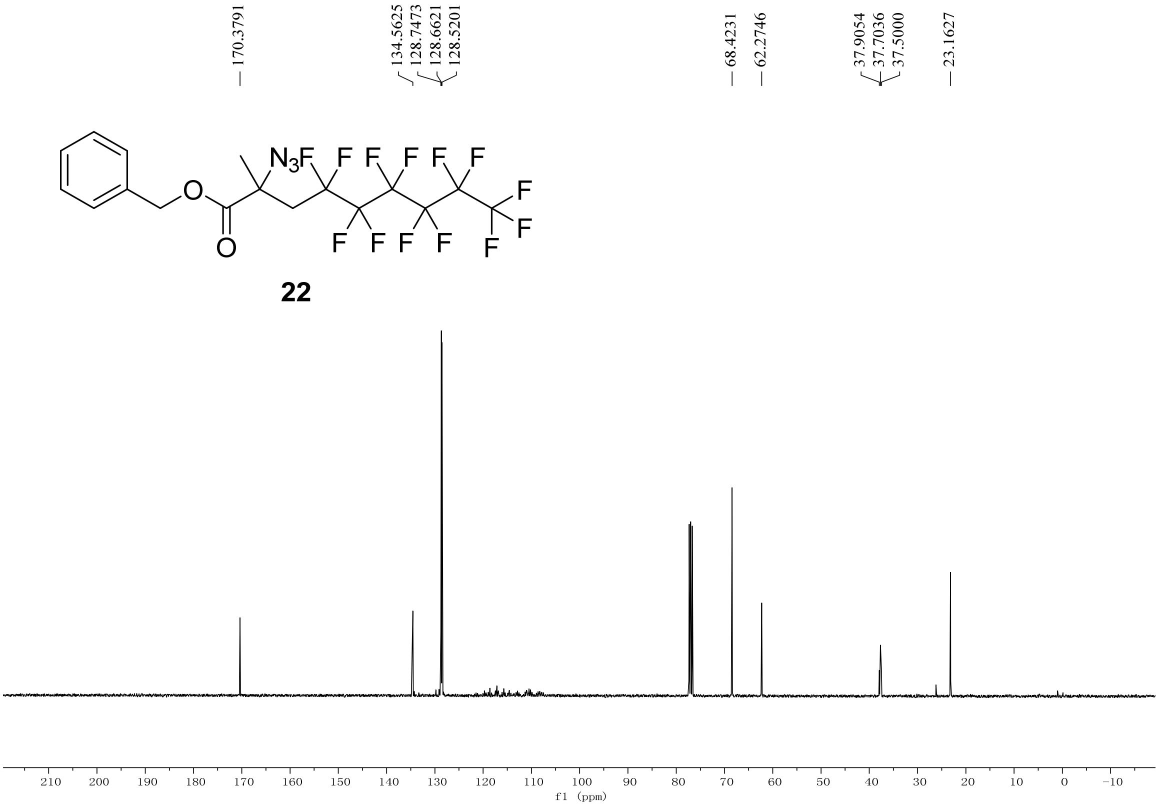

Supplementary Figure 55. ^{13}C NMR spectrum for compound **22**

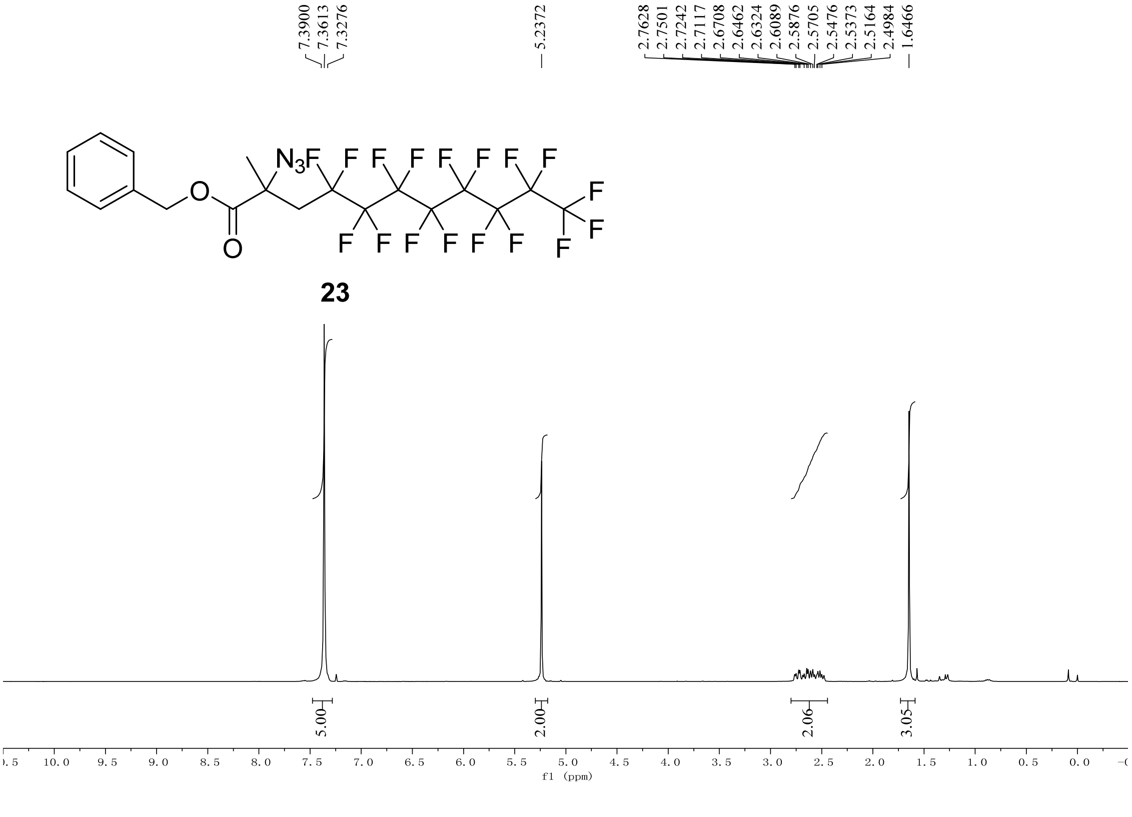

Supplementary Figure 56. ^{1}H NMR spectrum for compound **23**

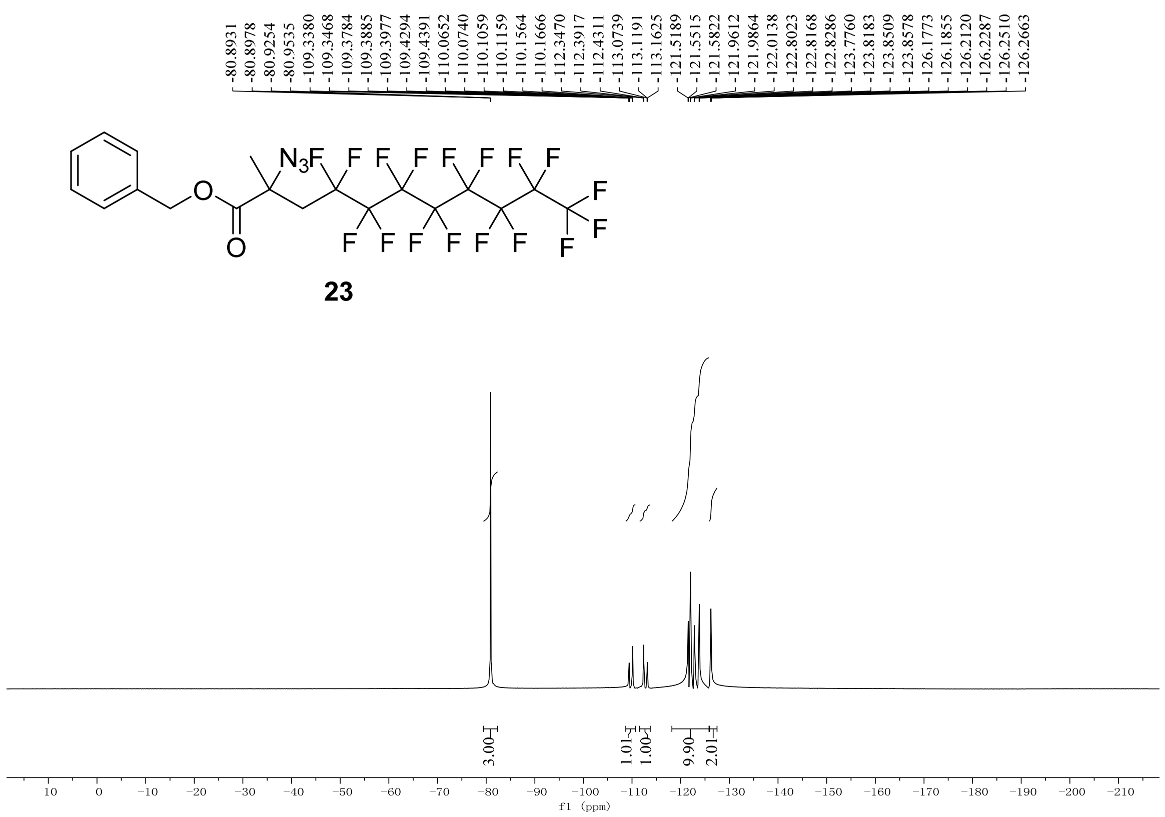

Supplementary Figure 57. ^{19}F NMR spectrum for compound **23**

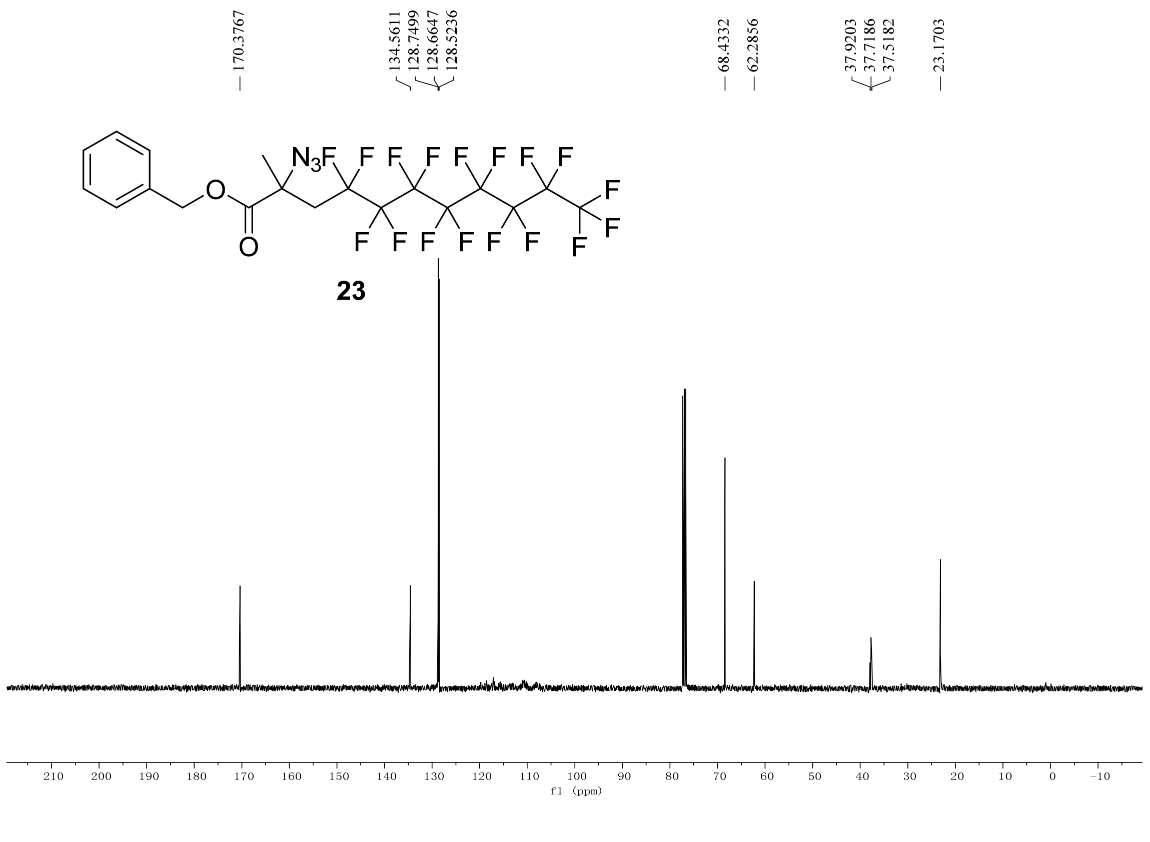

Supplementary Figure 58. ^{13}C NMR spectrum for compound **23**

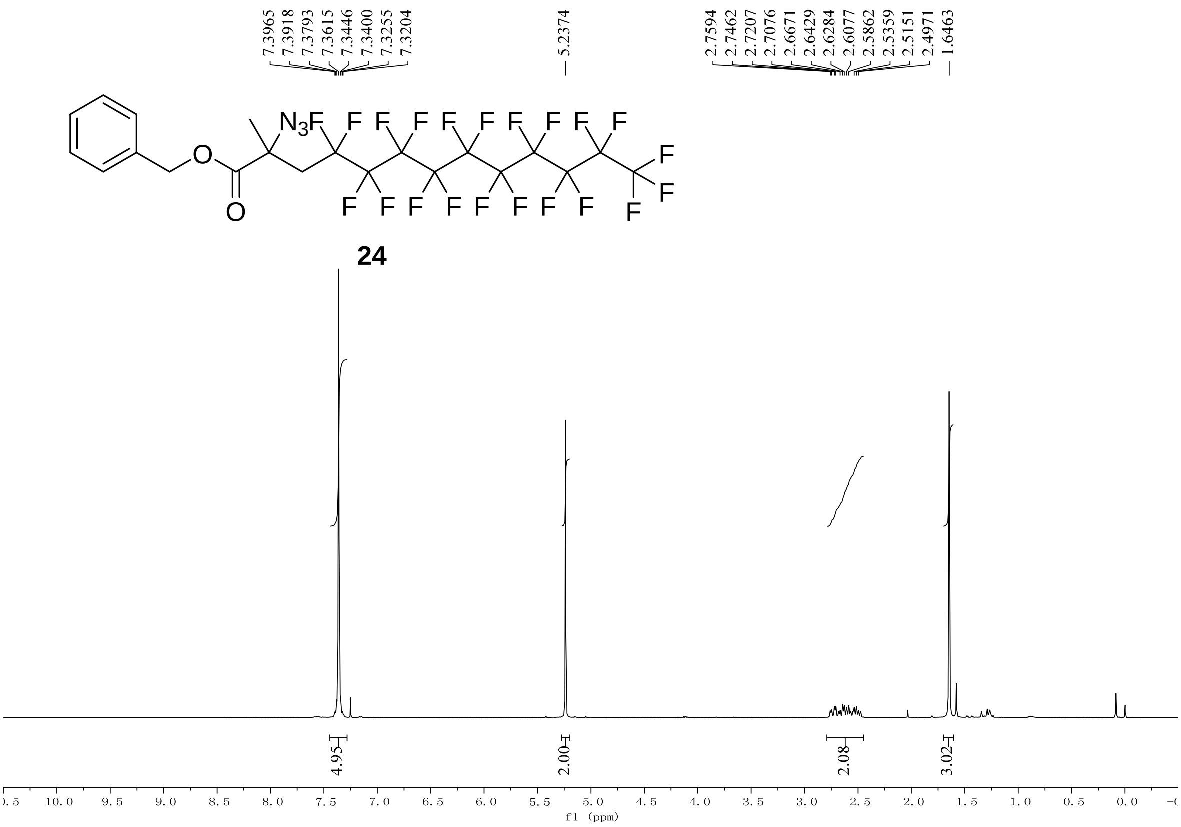

Supplementary Figure 59. ^{1}H NMR spectrum for compound **24**

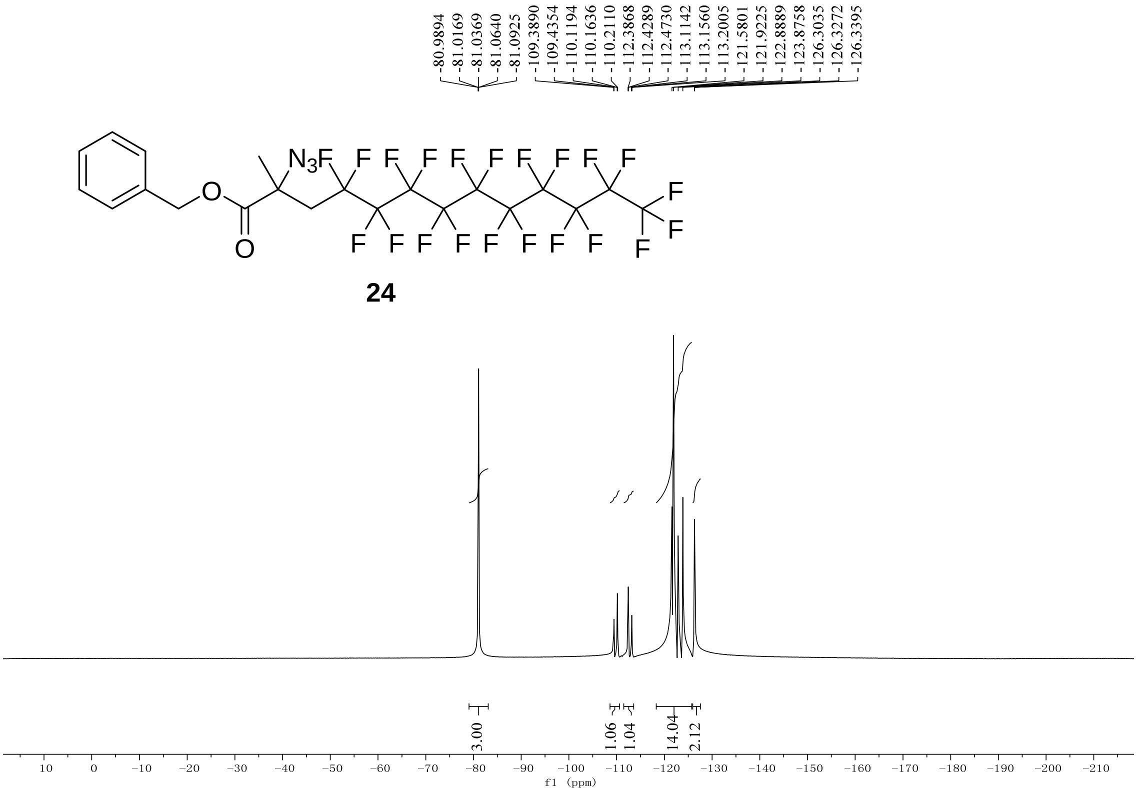

Supplementary Figure 60. ^{19}F NMR spectrum for compound **24**

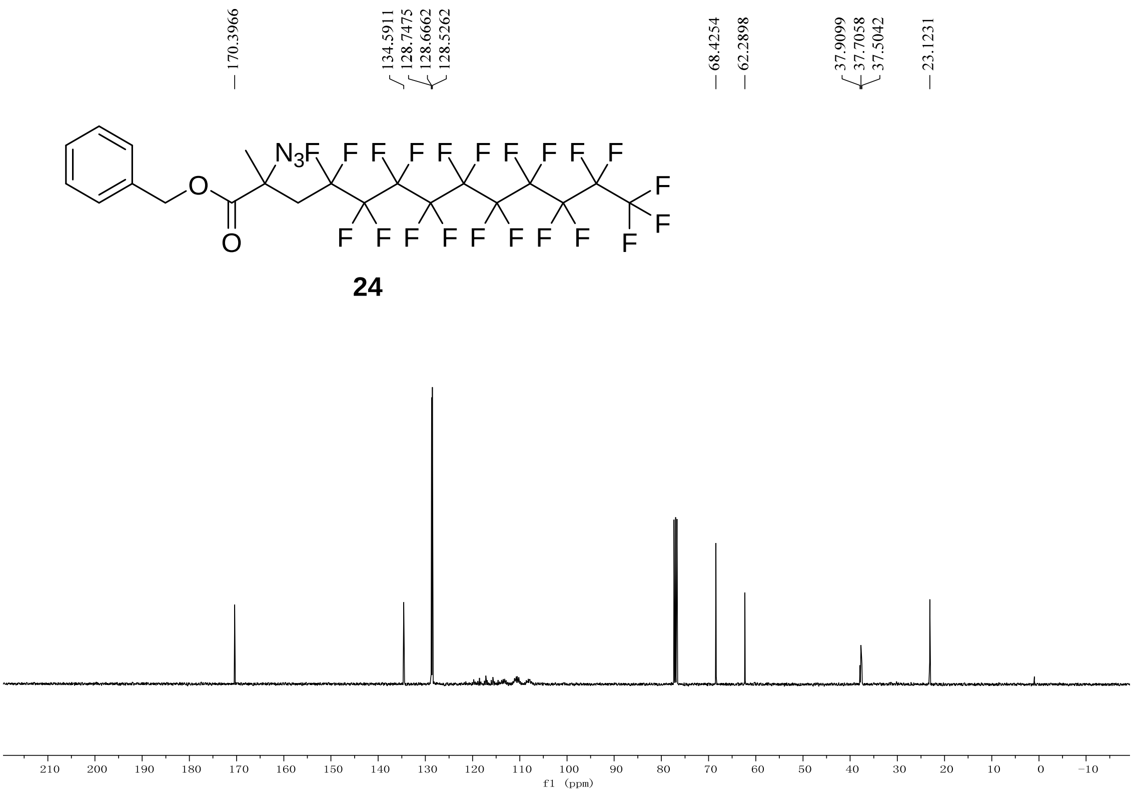

Supplementary Figure 61. ^{13}C NMR spectrum for compound **24**

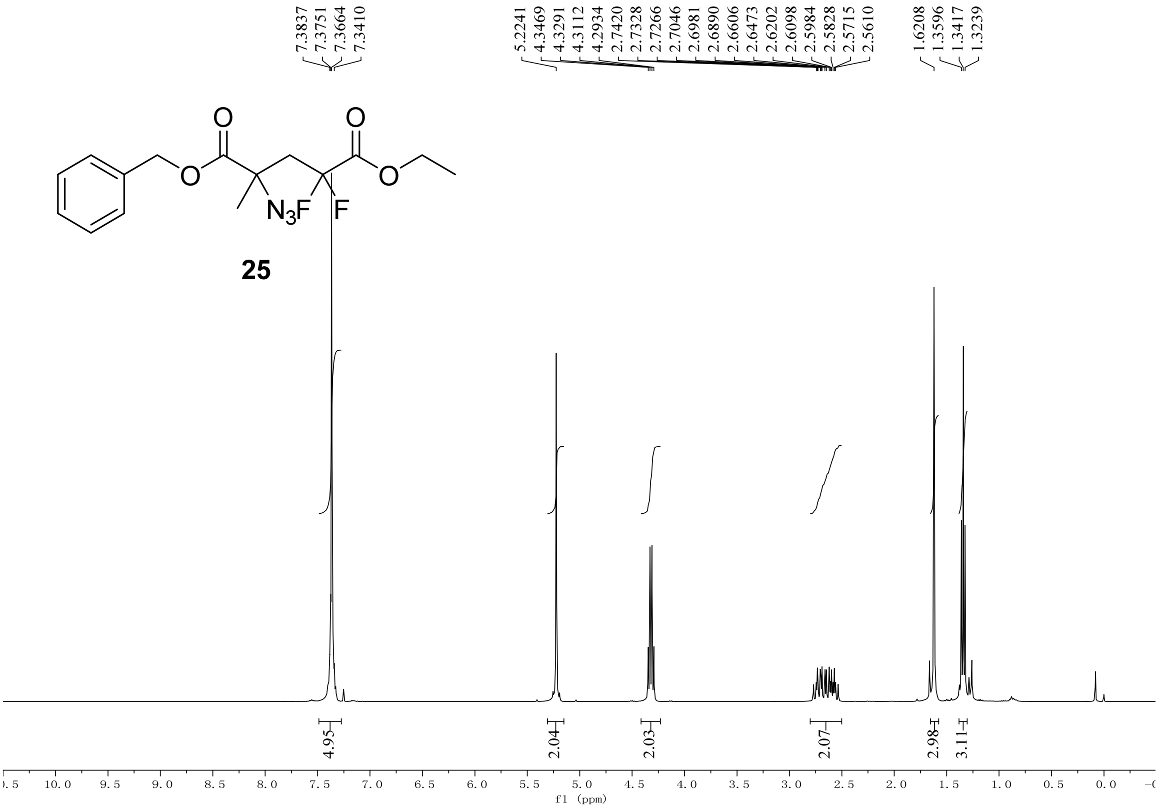

Supplementary Figure 62. ^{1}H NMR spectrum for compound **25**

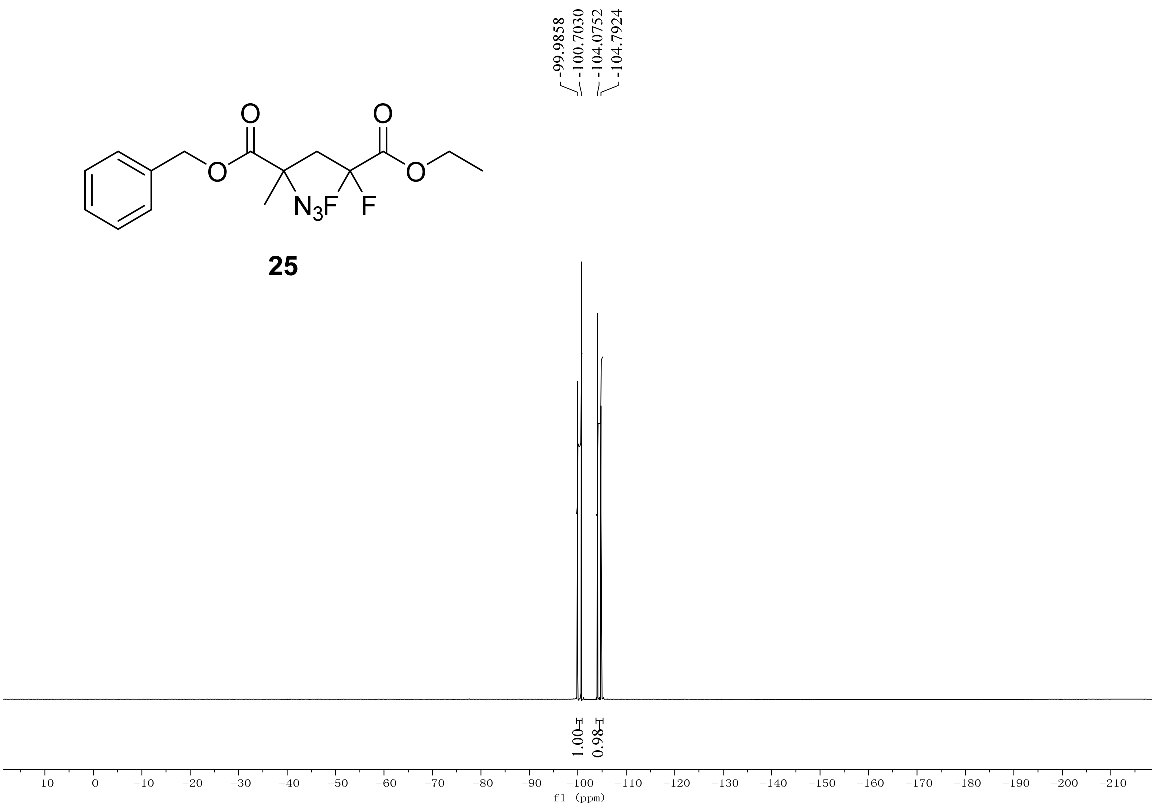

Supplementary Figure 63. ^{19}F NMR spectrum for compound **25**

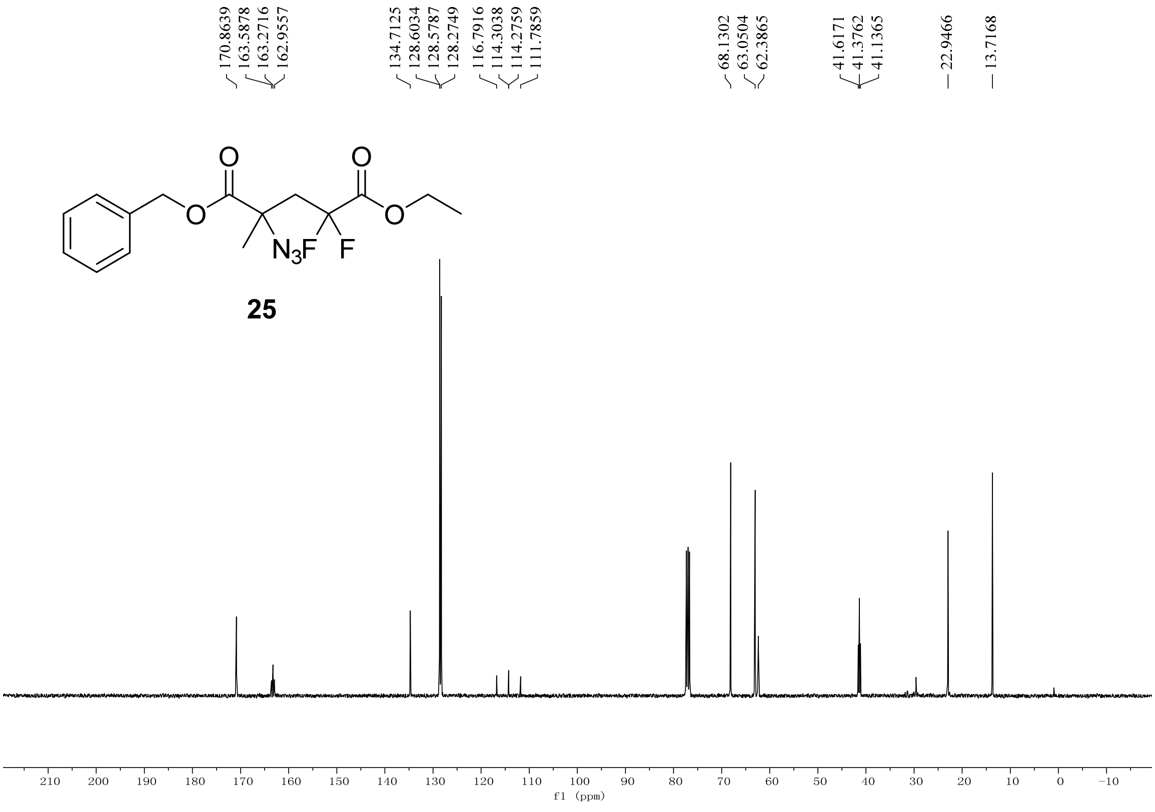

Supplementary Figure 64. ^{13}C NMR spectrum for compound **25**

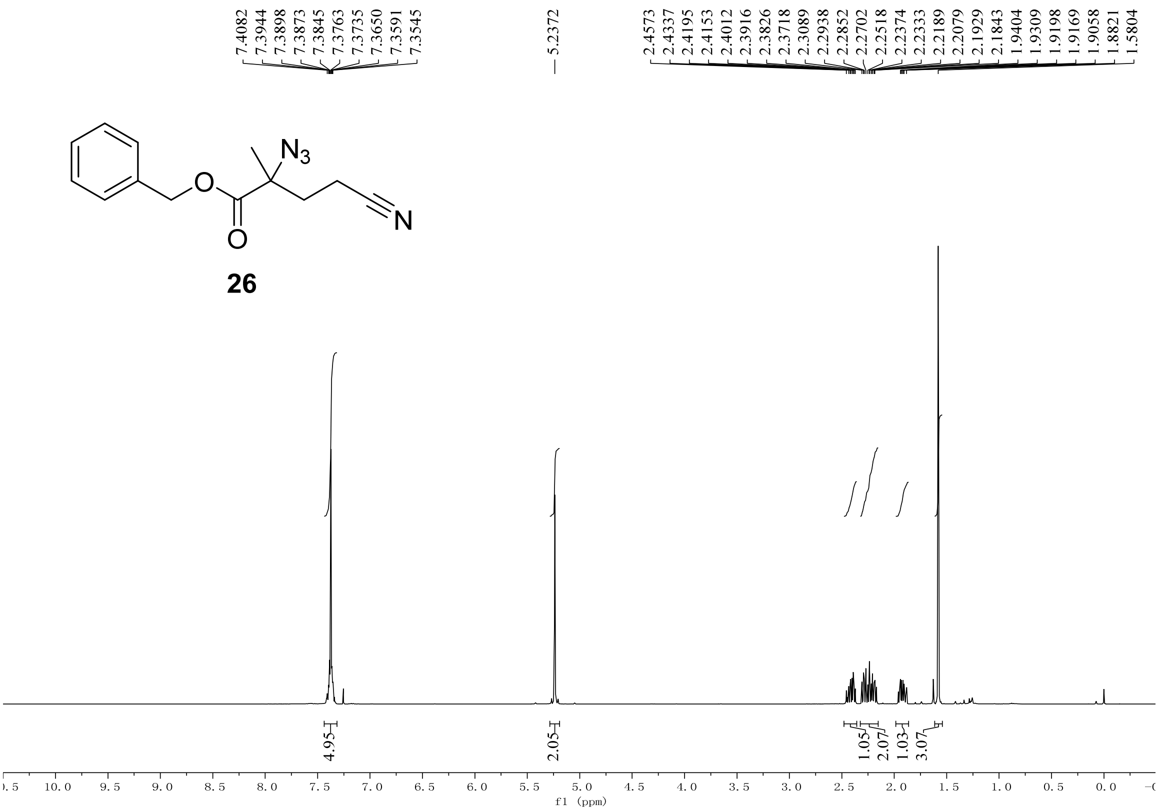

Supplementary Figure 65. ^{1}H NMR spectrum for compound **26**

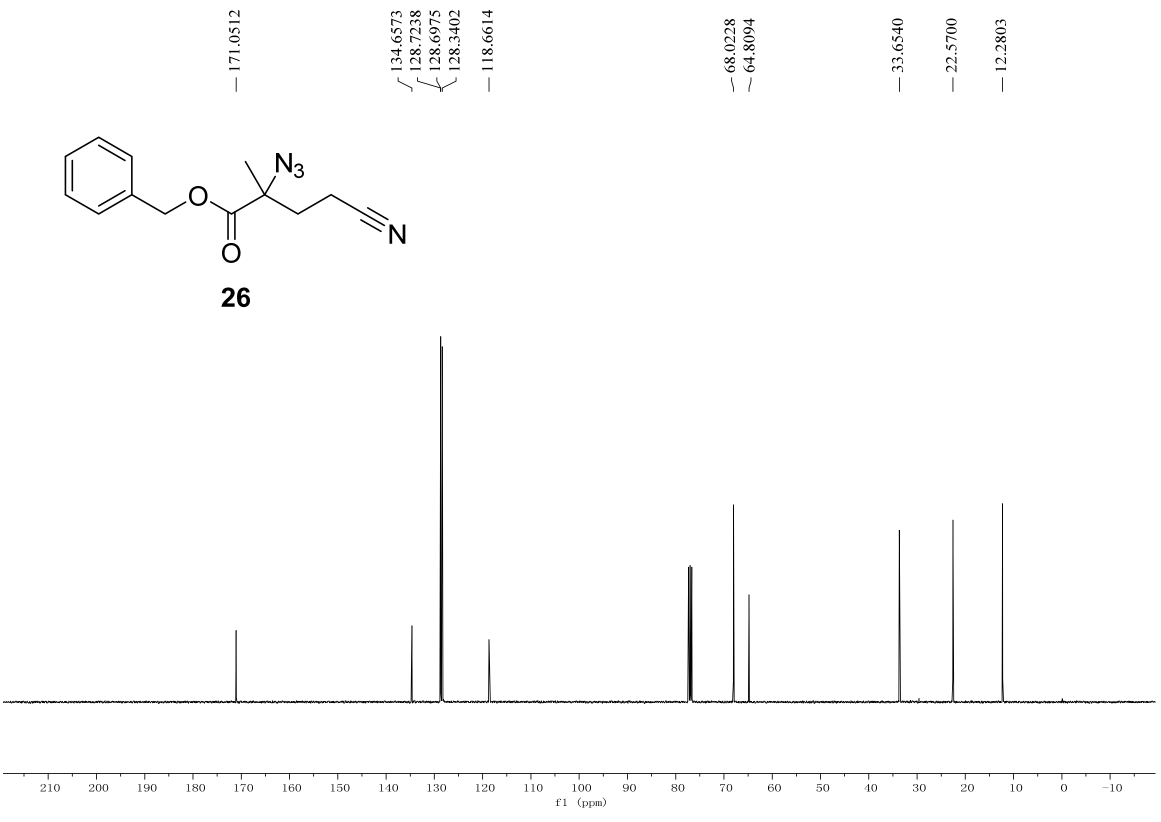

Supplementary Figure 66. ^{13}C NMR spectrum for compound **26**

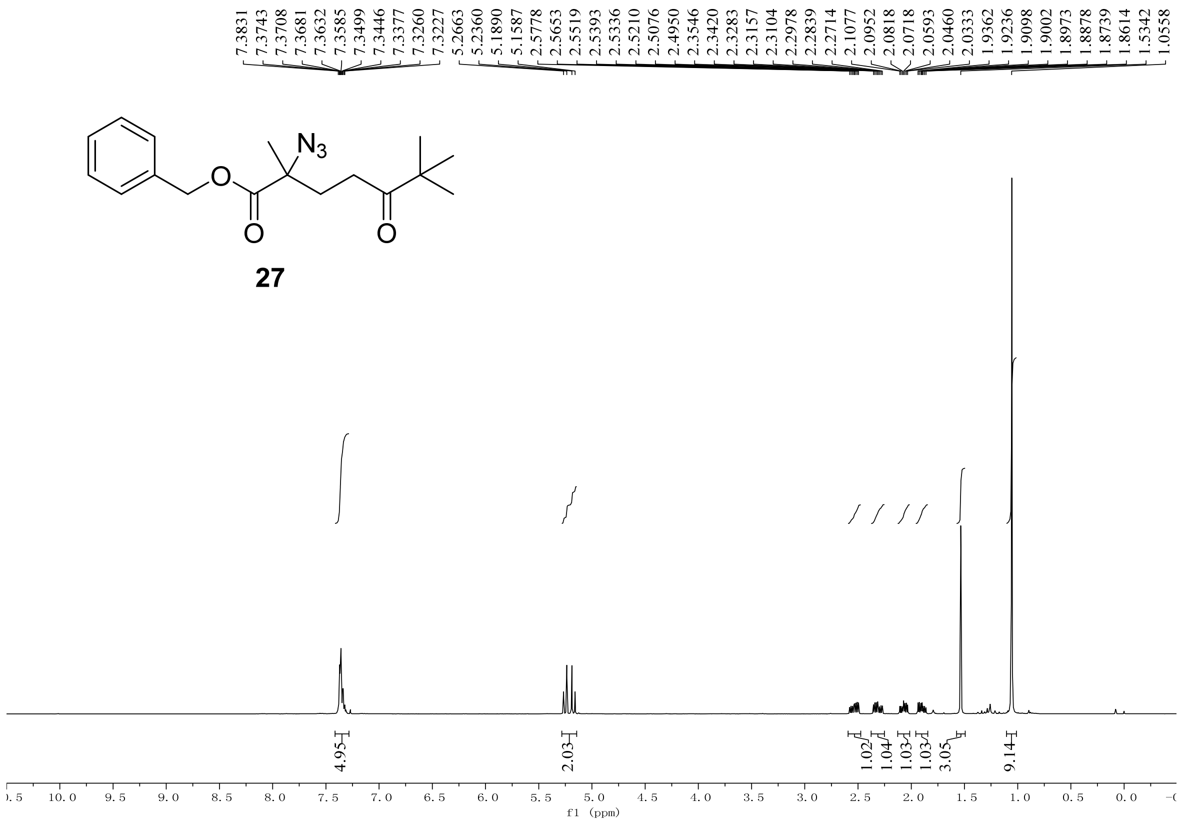

Supplementary Figure 67. ^{1}H NMR spectrum for compound **27**

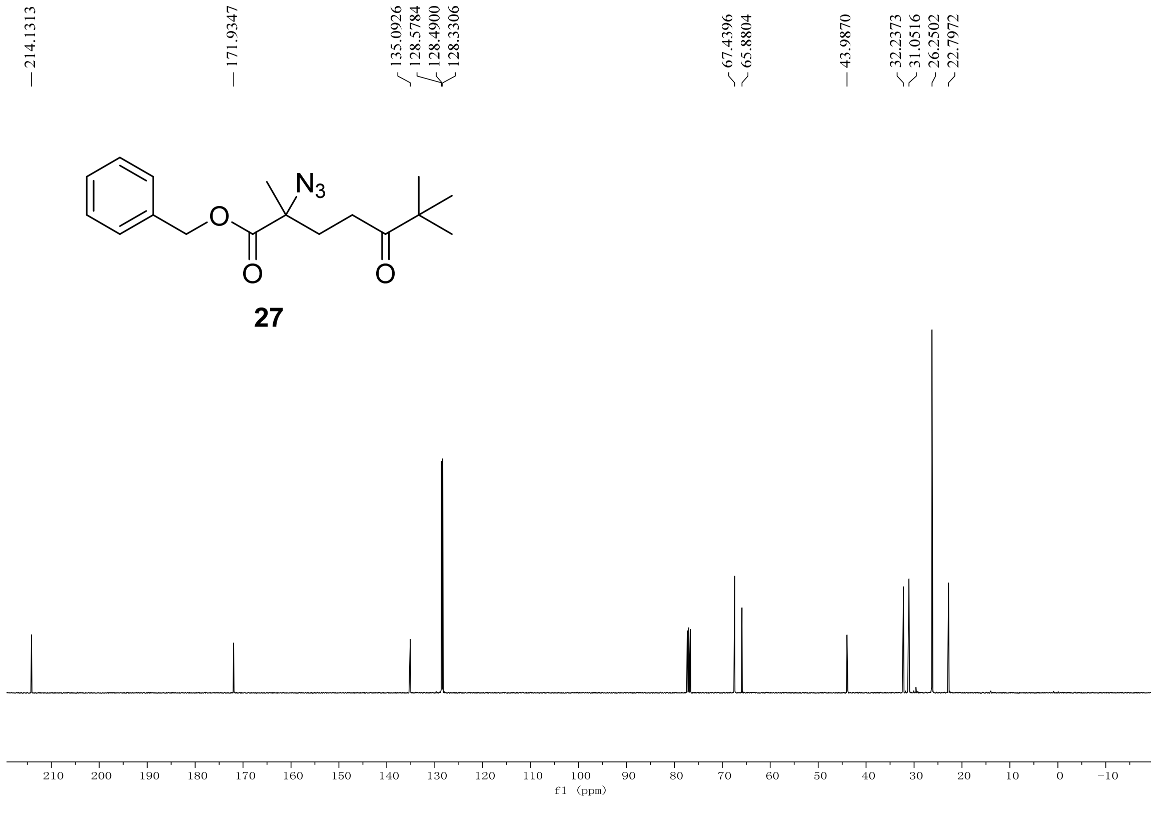

Supplementary Figure 68. ^{13}C NMR spectrum for compound **27**

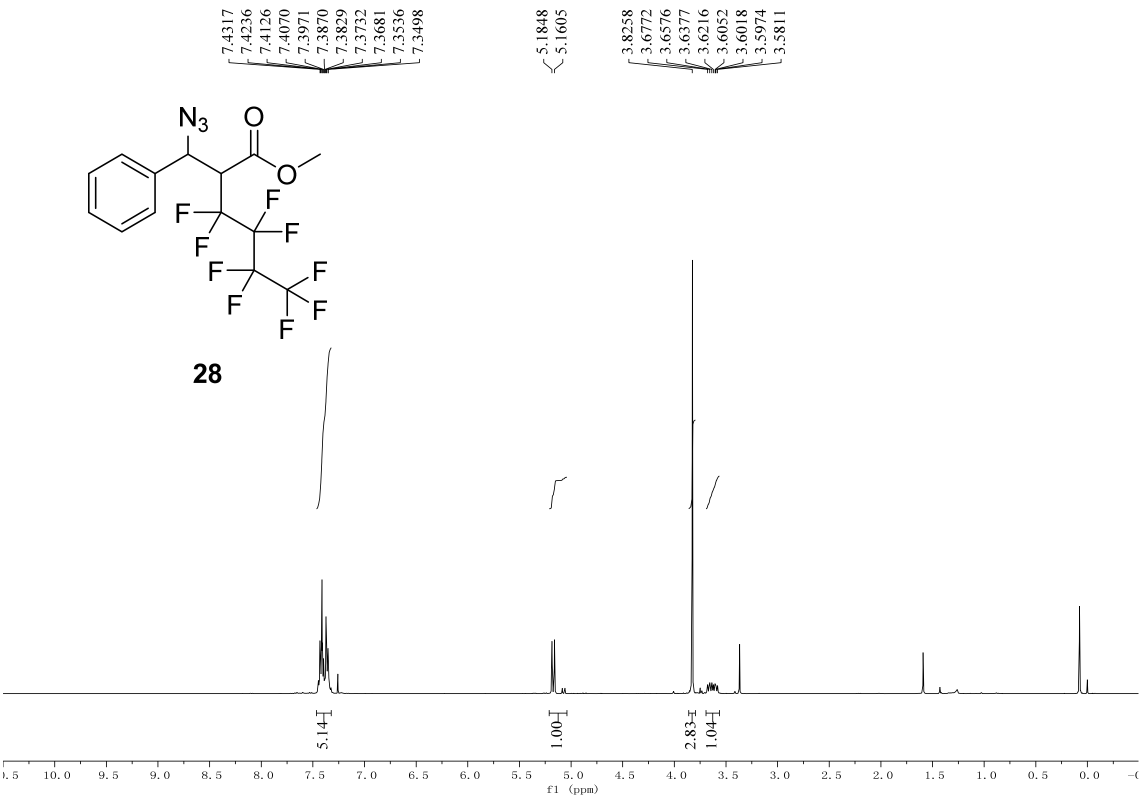

Supplementary Figure 69. ^{1}H NMR spectrum for compound **28**

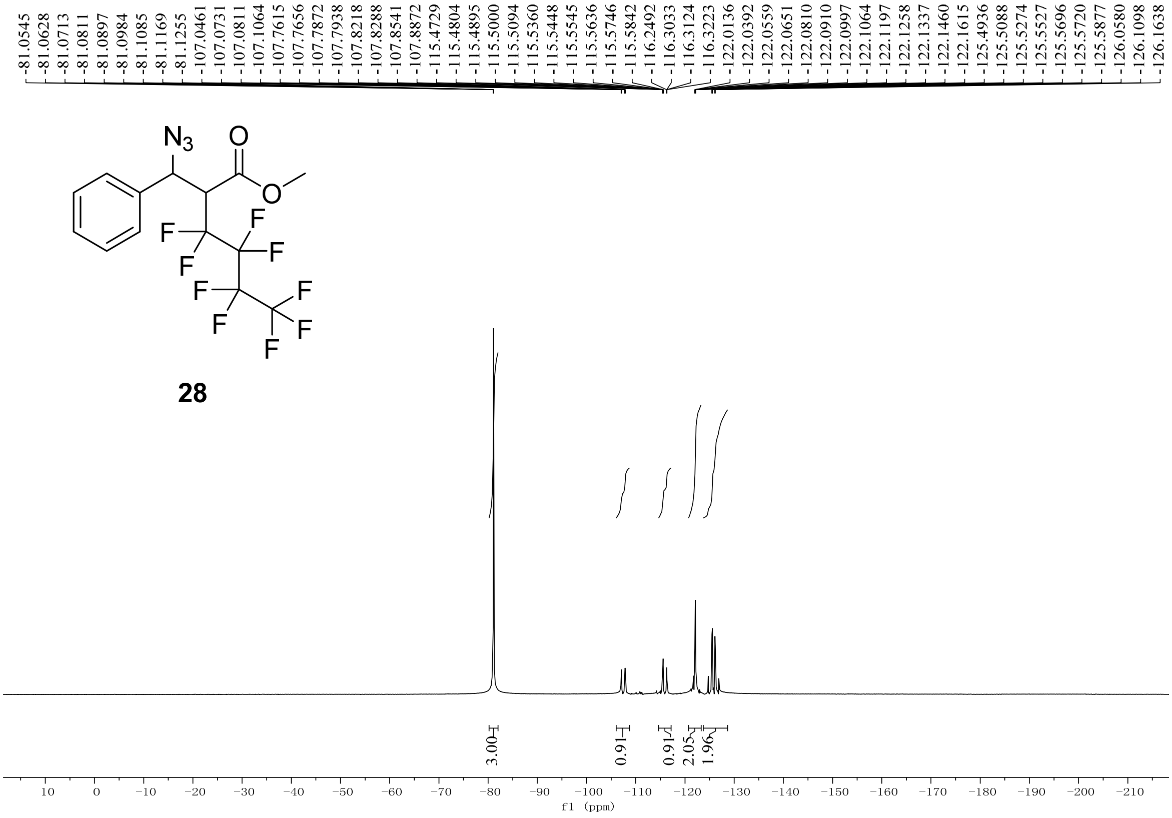

Supplementary Figure 70. ^{19}F NMR spectrum for compound **28**

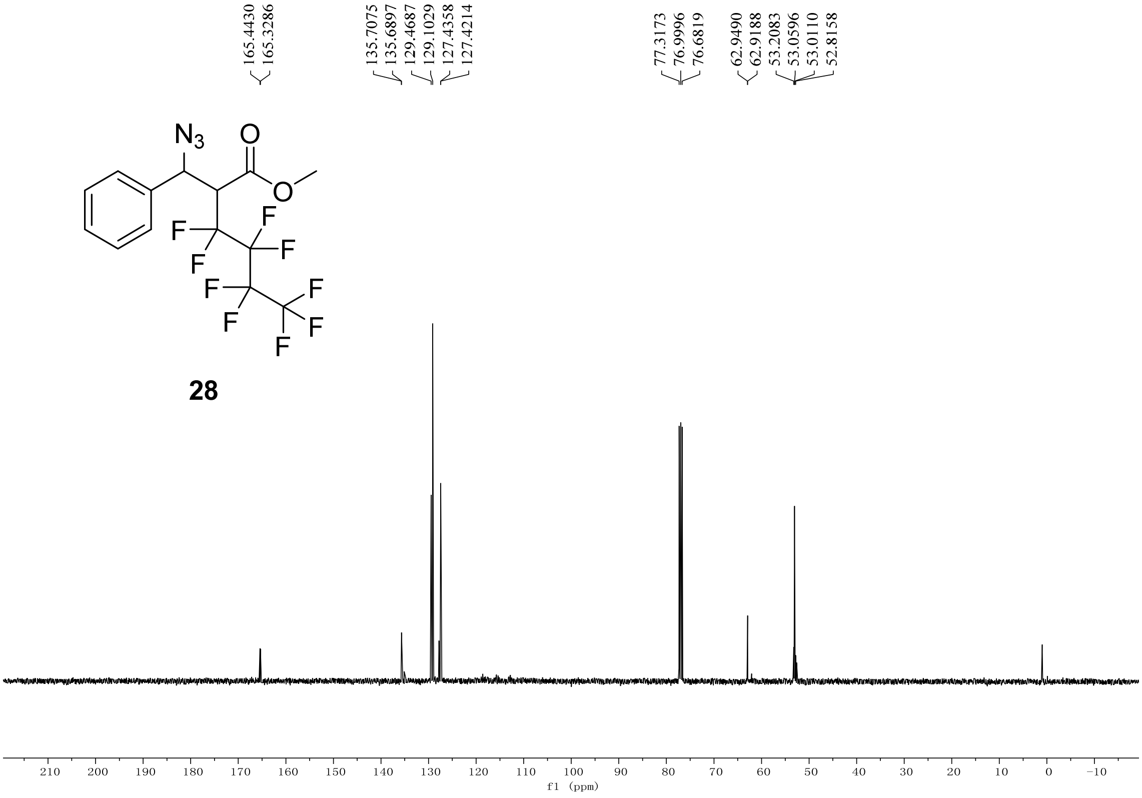

Supplementary Figure 71. ^{13}C NMR spectrum for compound **28**

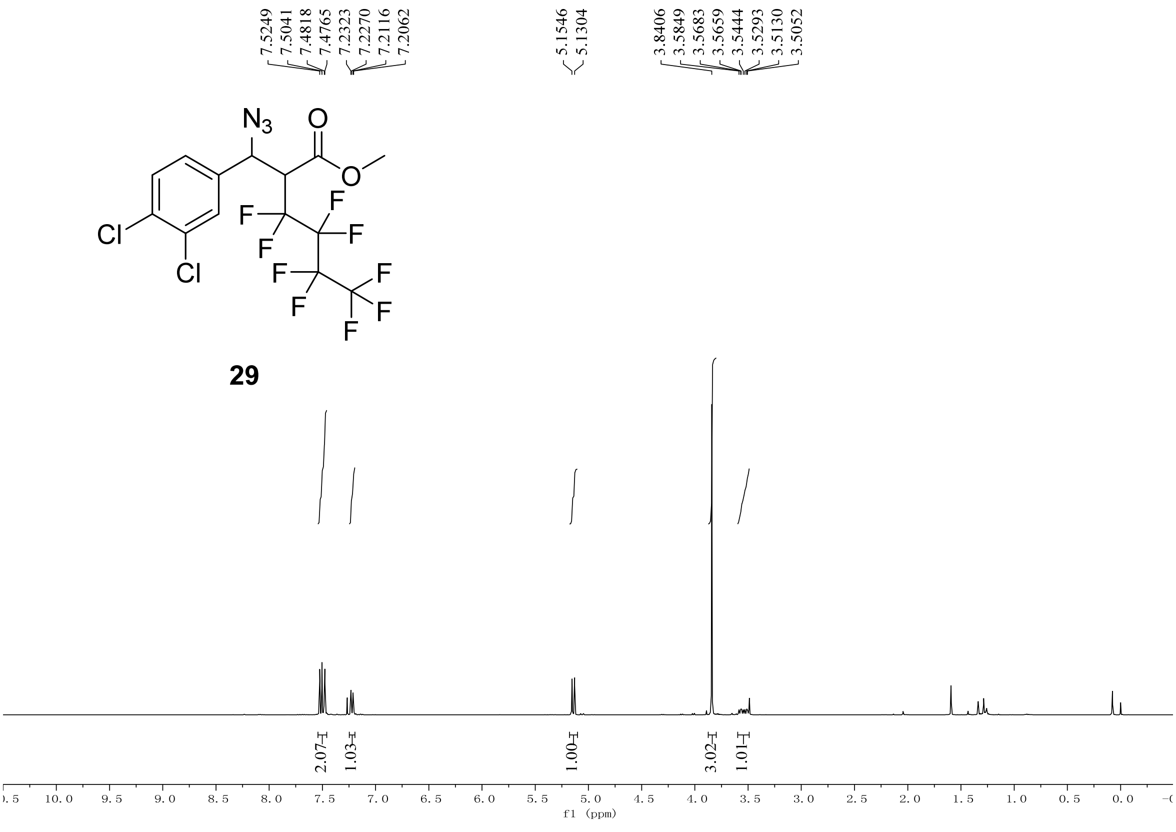

Supplementary Figure 72. ^{1}H NMR spectrum for compound **29**

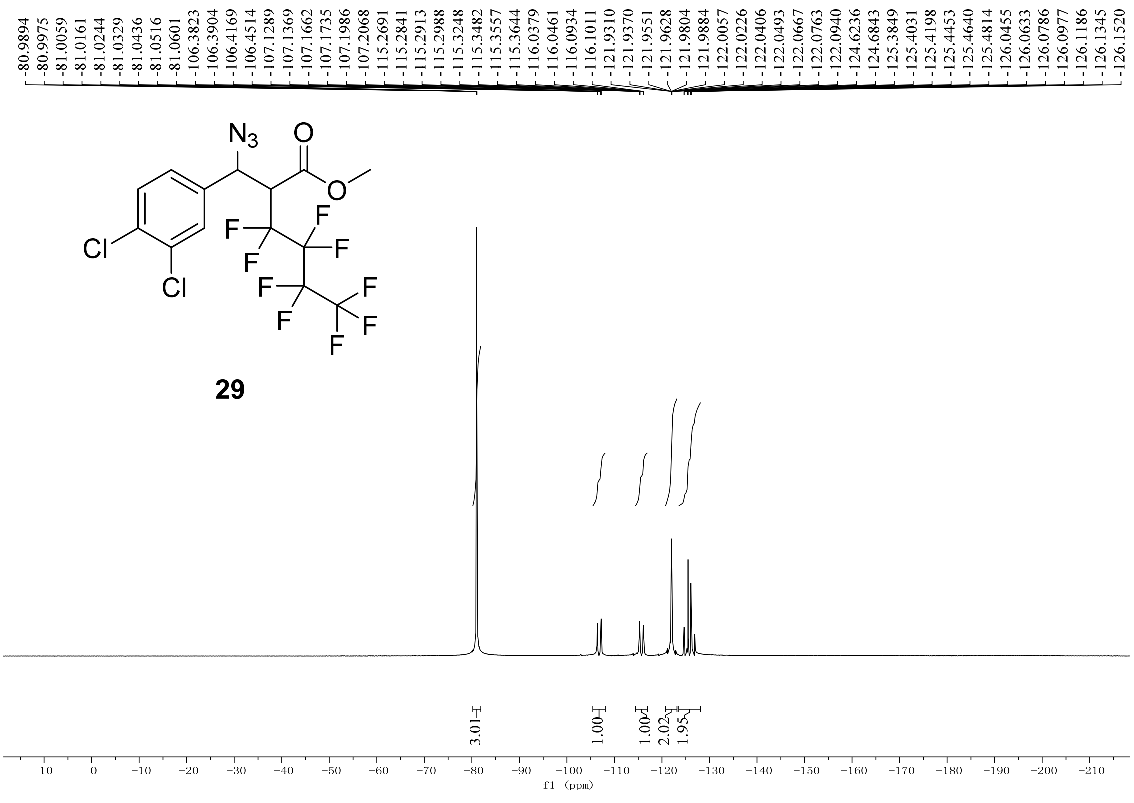

Supplementary Figure 73. ^{19}F NMR spectrum for compound **29**

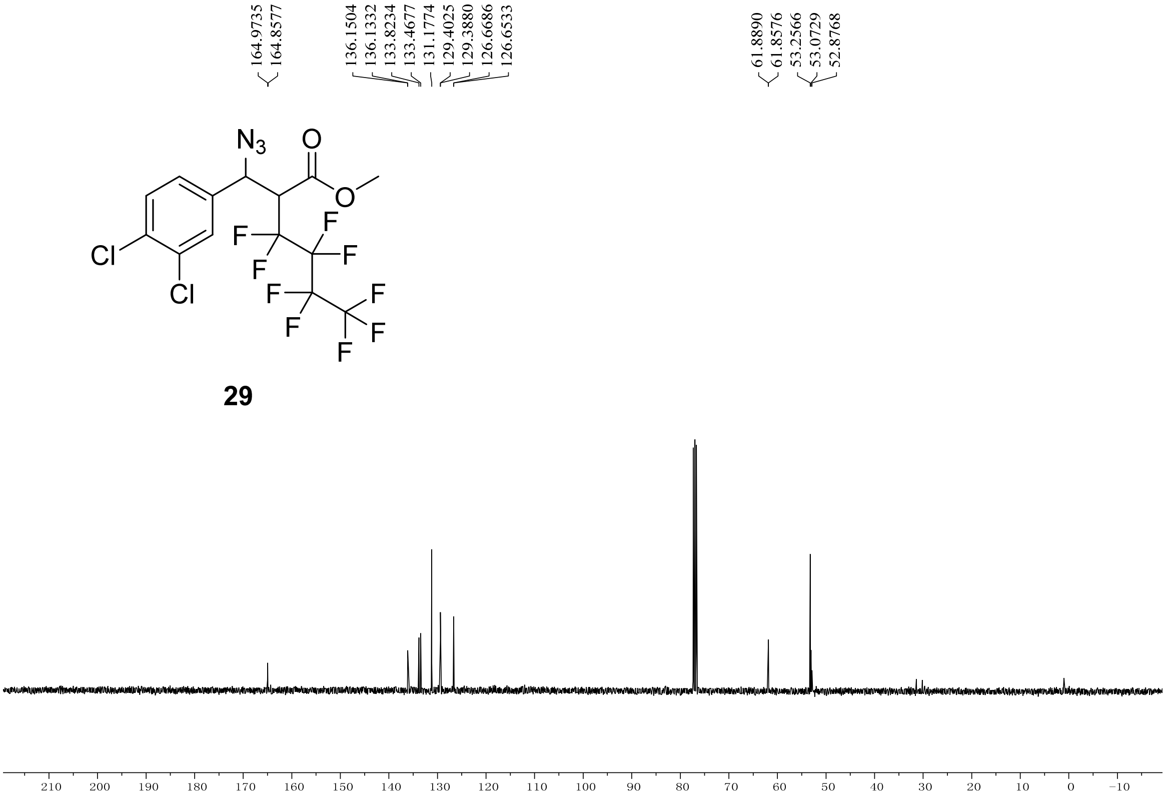

Supplementary Figure 74. ^{13}C NMR spectrum for compound **29**

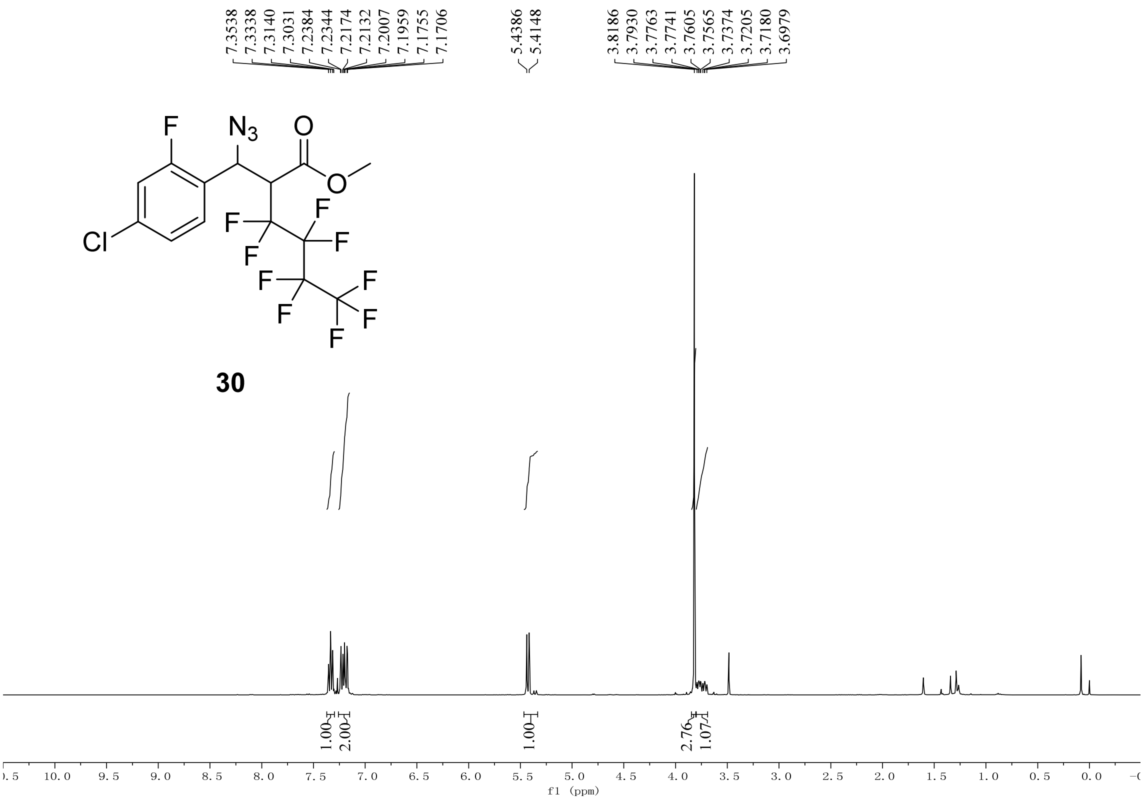

Supplementary Figure 75. ^{1}H NMR spectrum for compound **30**

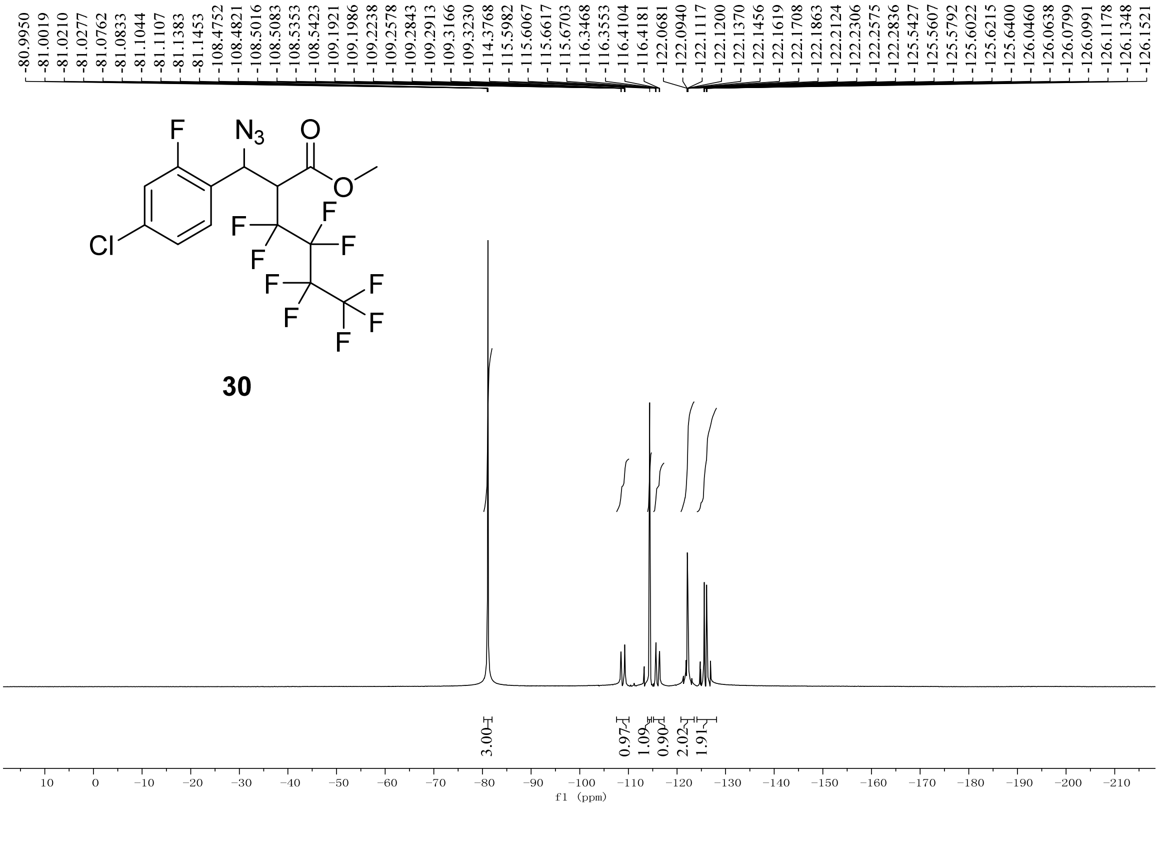

Supplementary Figure 76. ^{19}F NMR spectrum for compound **30**

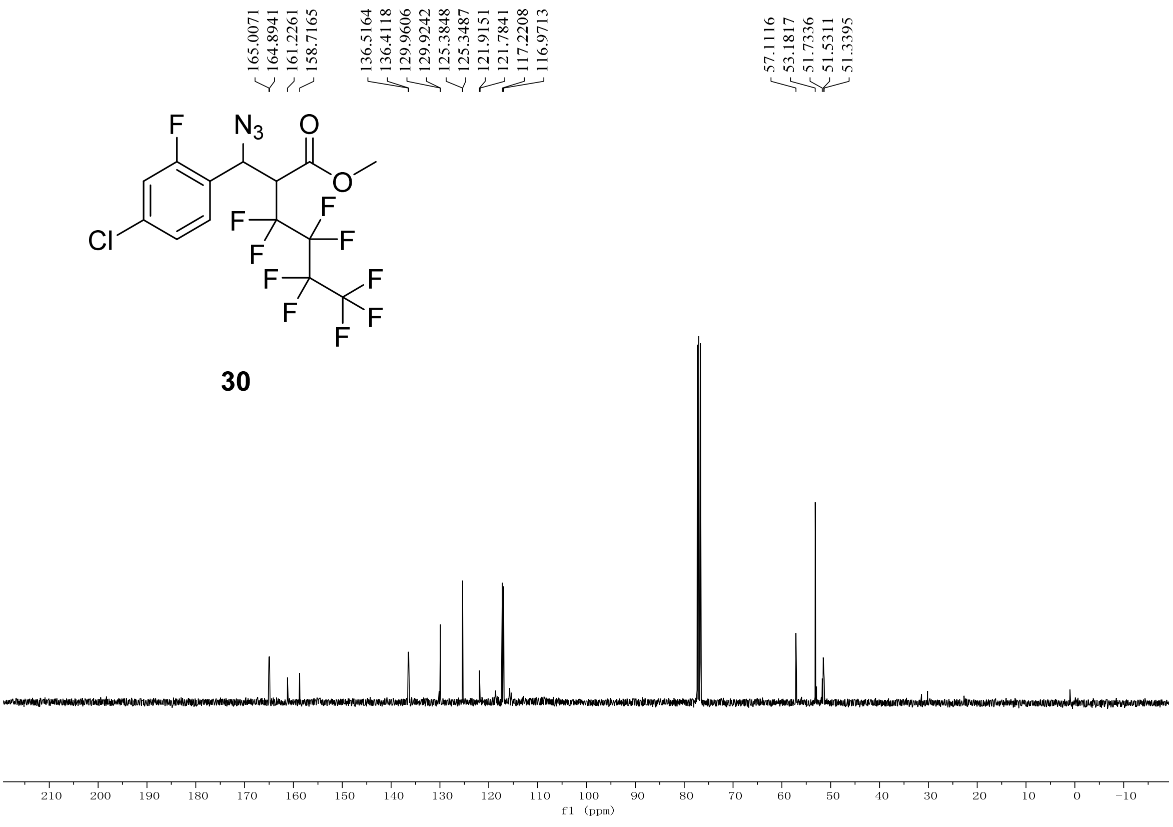

Supplementary Figure 77. ^{13}C NMR spectrum for compound **30**

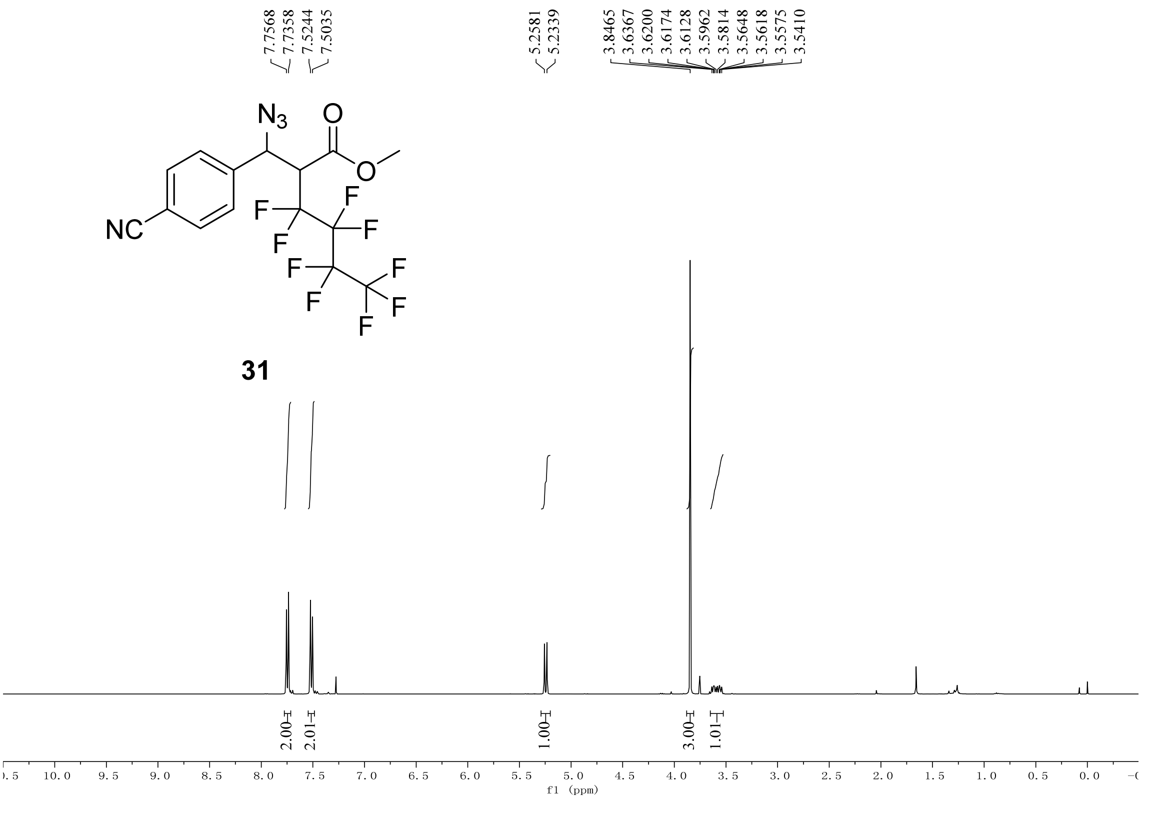

Supplementary Figure 78. ^{1}H NMR spectrum for compound **31**

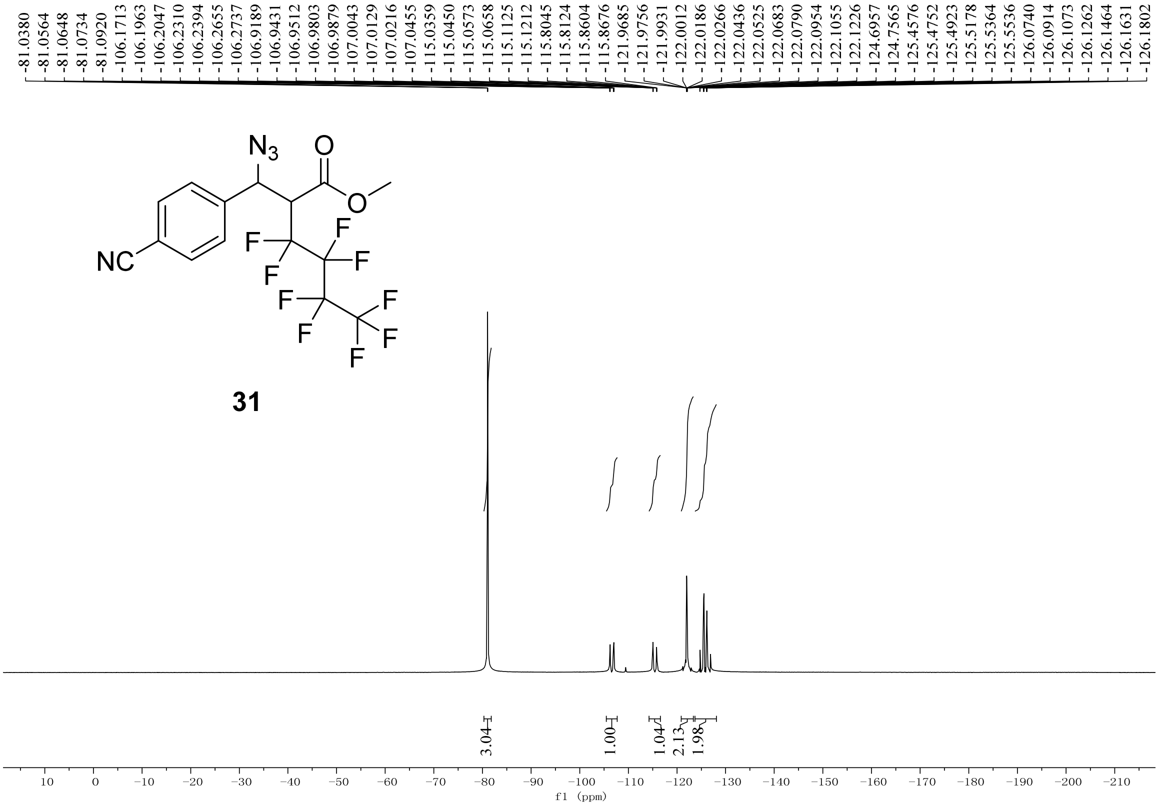

Supplementary Figure 79. ^{19}F NMR spectrum for compound **31**

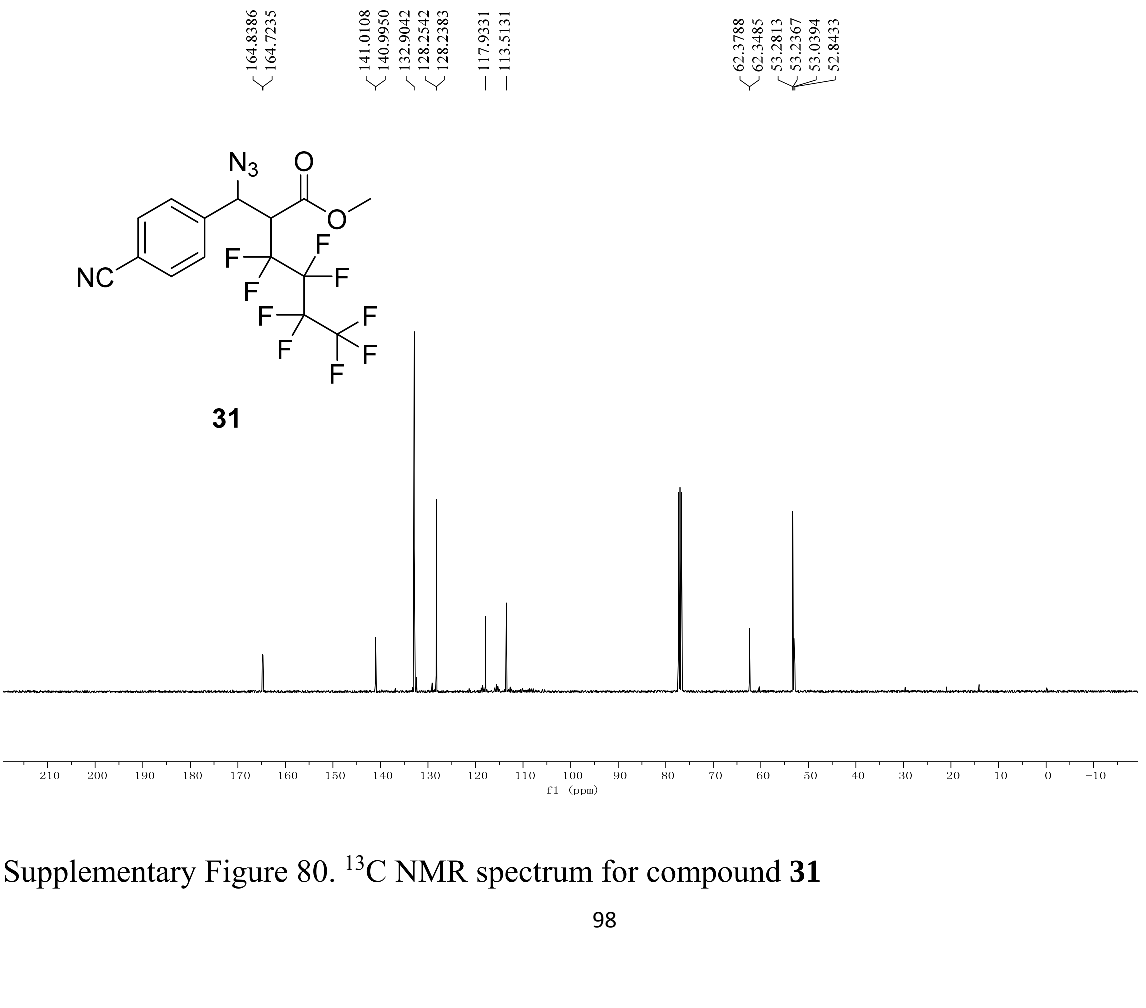

Supplementary Figure 80. ^{13}C NMR spectrum for compound **31**

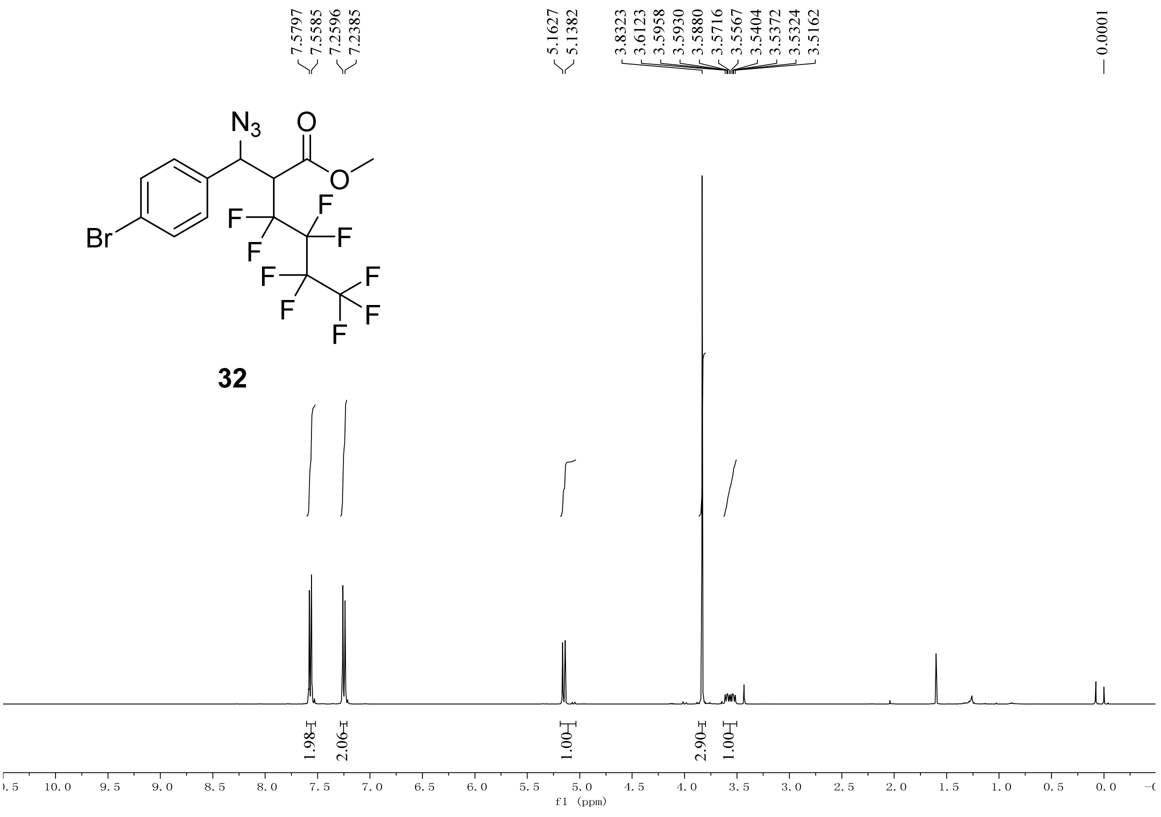

Supplementary Figure 81. ^{1}H NMR spectrum for compound **32**

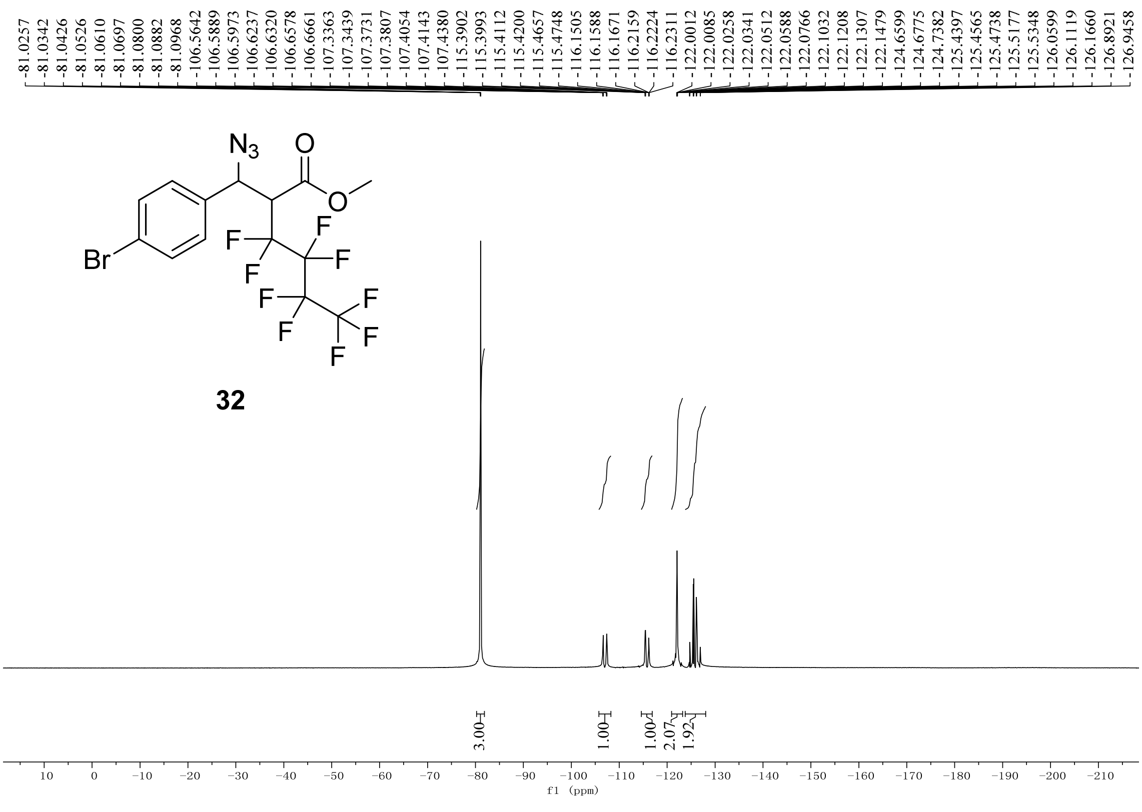

Supplementary Figure 82. ^{19}F NMR spectrum for compound **32**

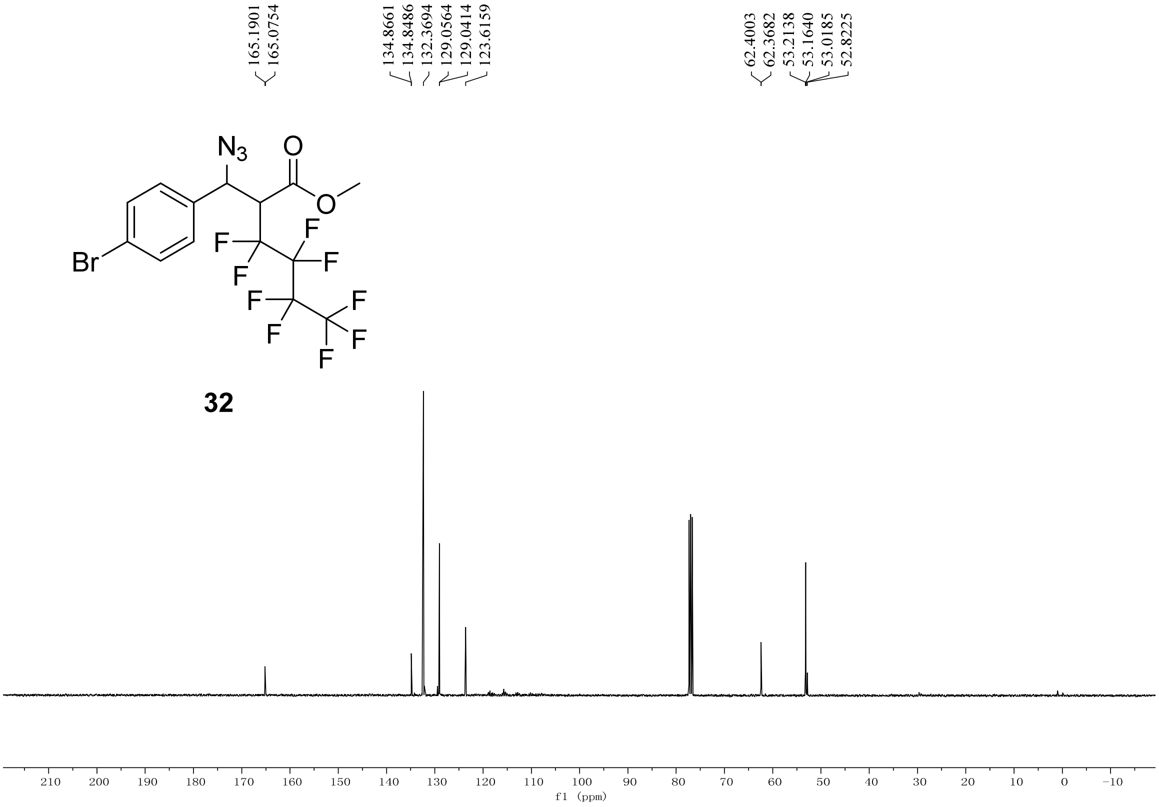

Supplementary Figure 83. ^{13}C NMR spectrum for compound **32**

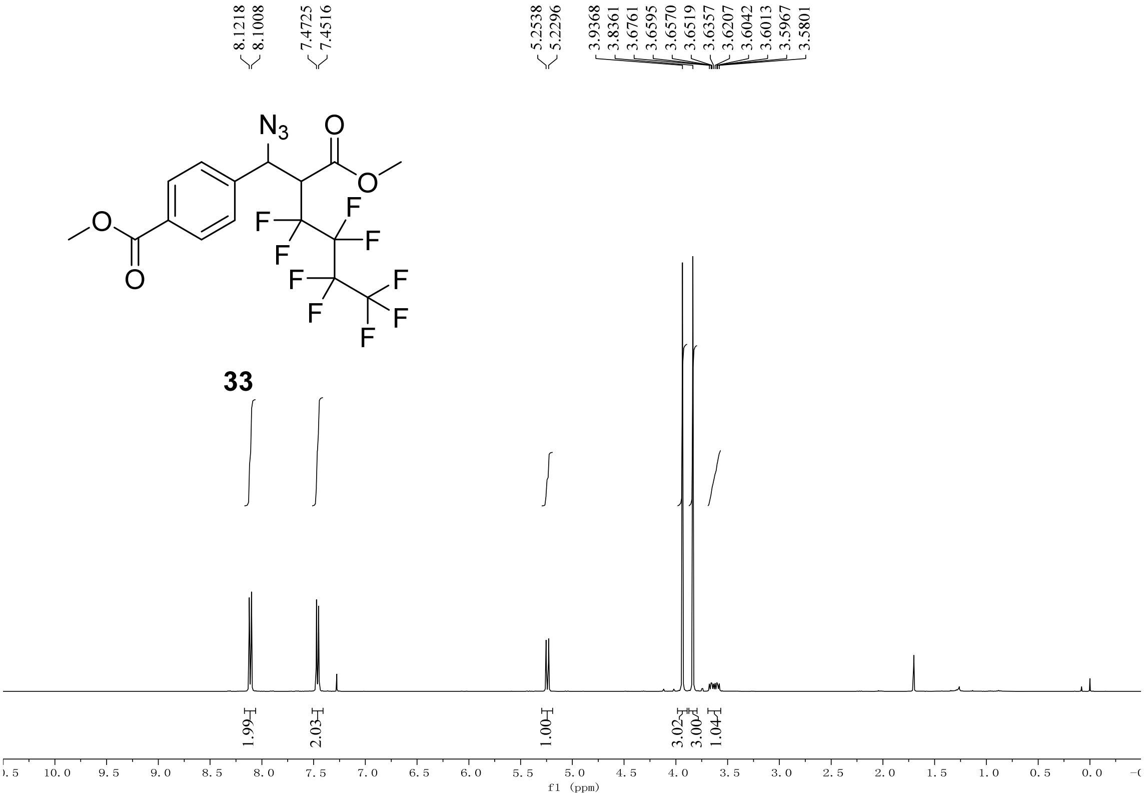

Supplementary Figure 84. ^{1}H NMR spectrum for compound **33**

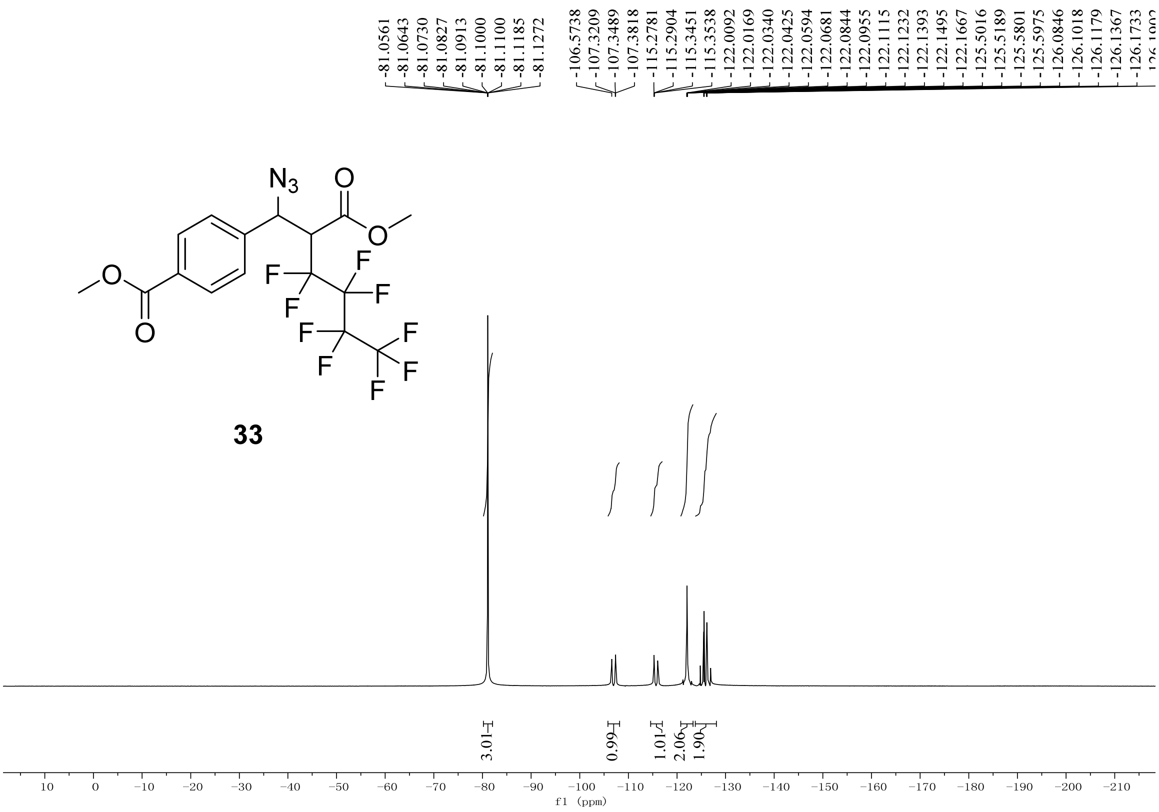

Supplementary Figure 85. ^{19}F NMR spectrum for compound **33**

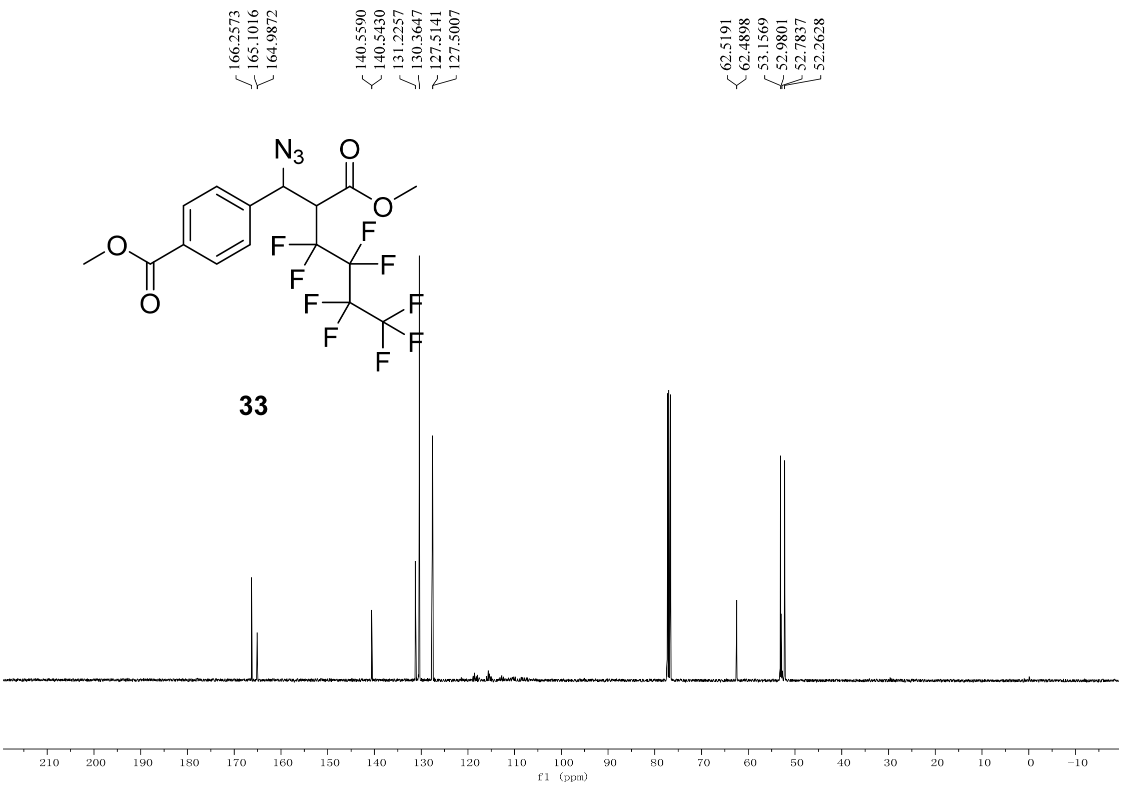

Supplementary Figure 86. ^{13}C NMR spectrum for compound **33**

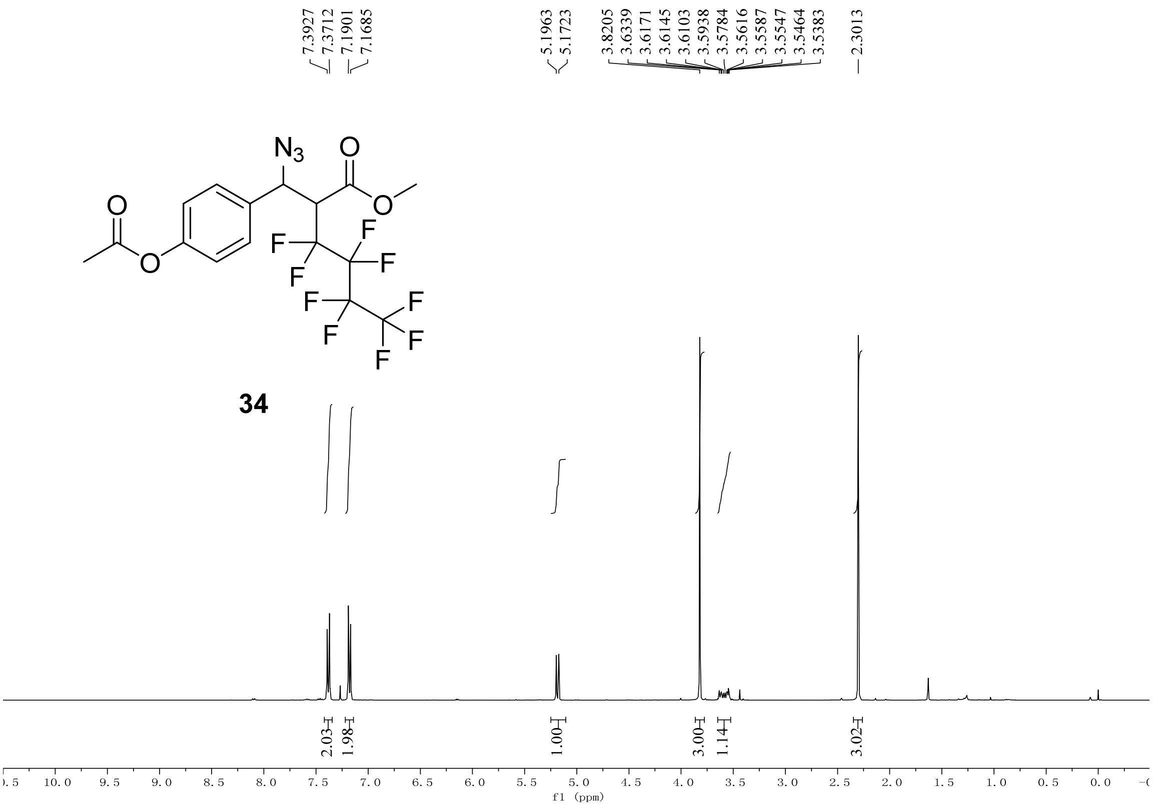

Supplementary Figure 87. ^{1}H NMR spectrum for compound **34**

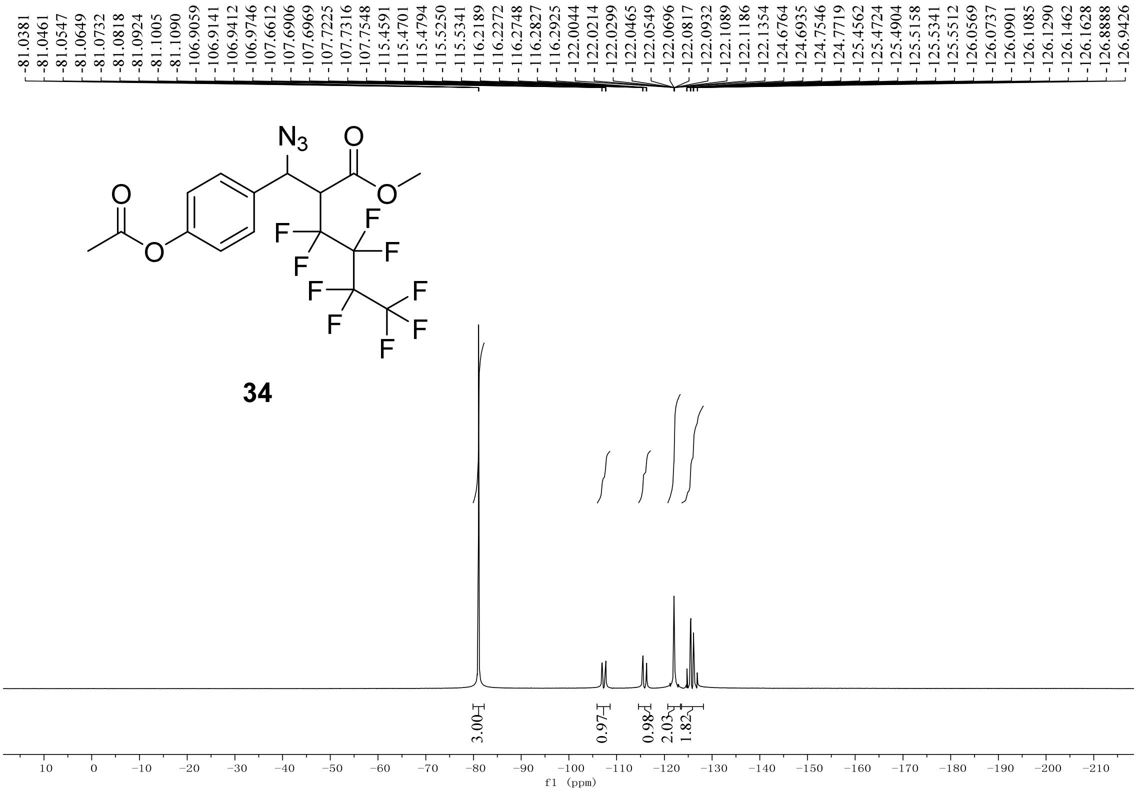

Supplementary Figure 88. ^{19}F NMR spectrum for compound **34**

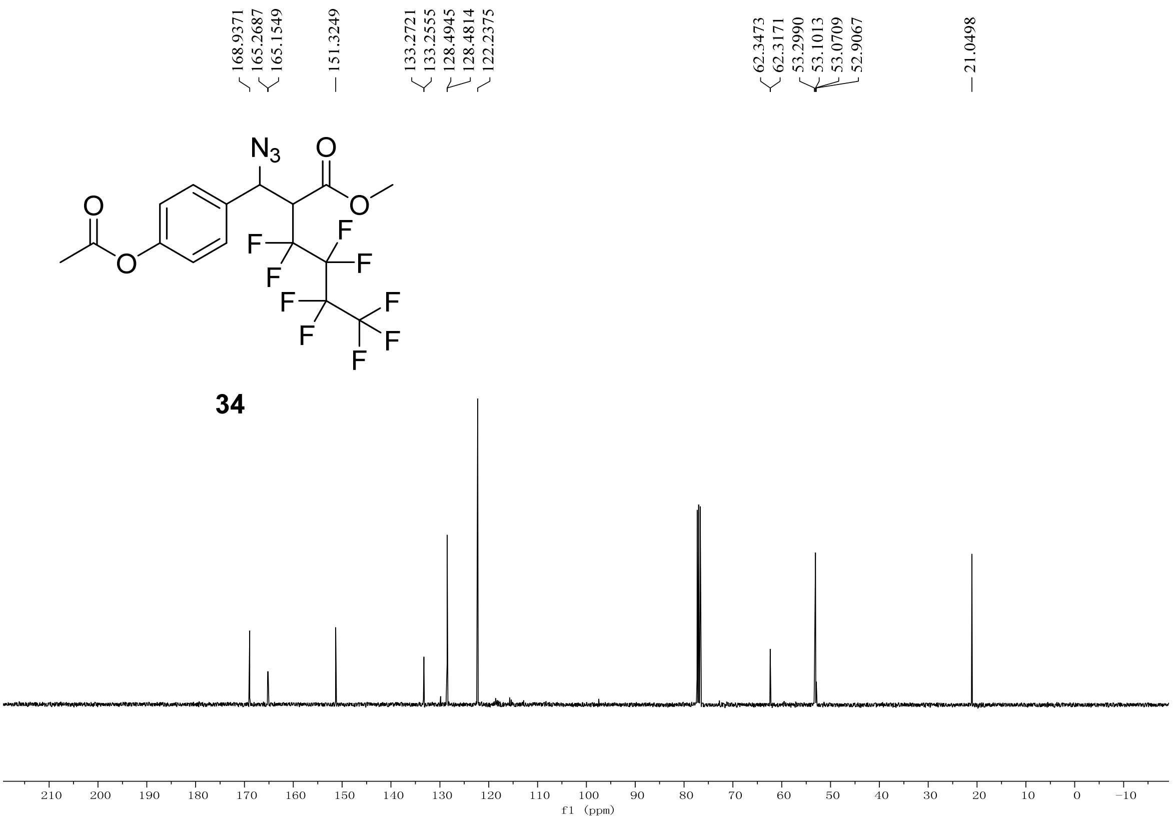

Supplementary Figure 89. ^{13}C NMR spectrum for compound **34**

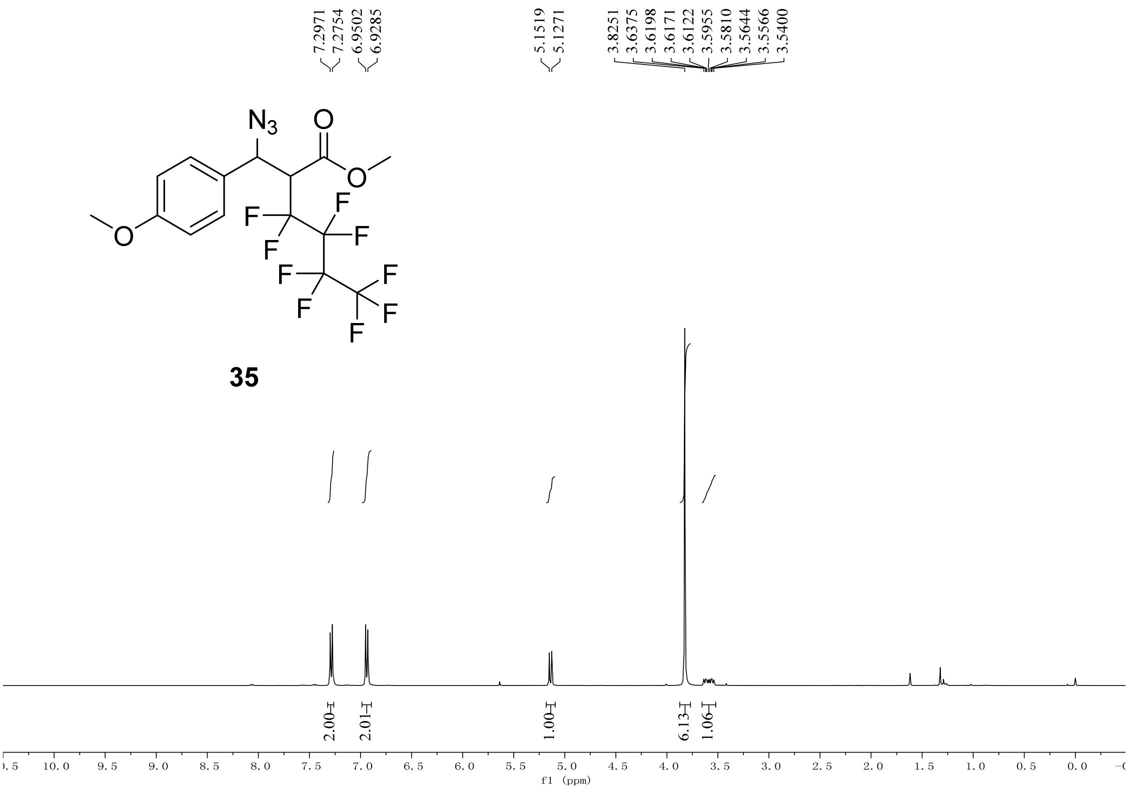

Supplementary Figure 90. ^{1}H NMR spectrum for compound **35**

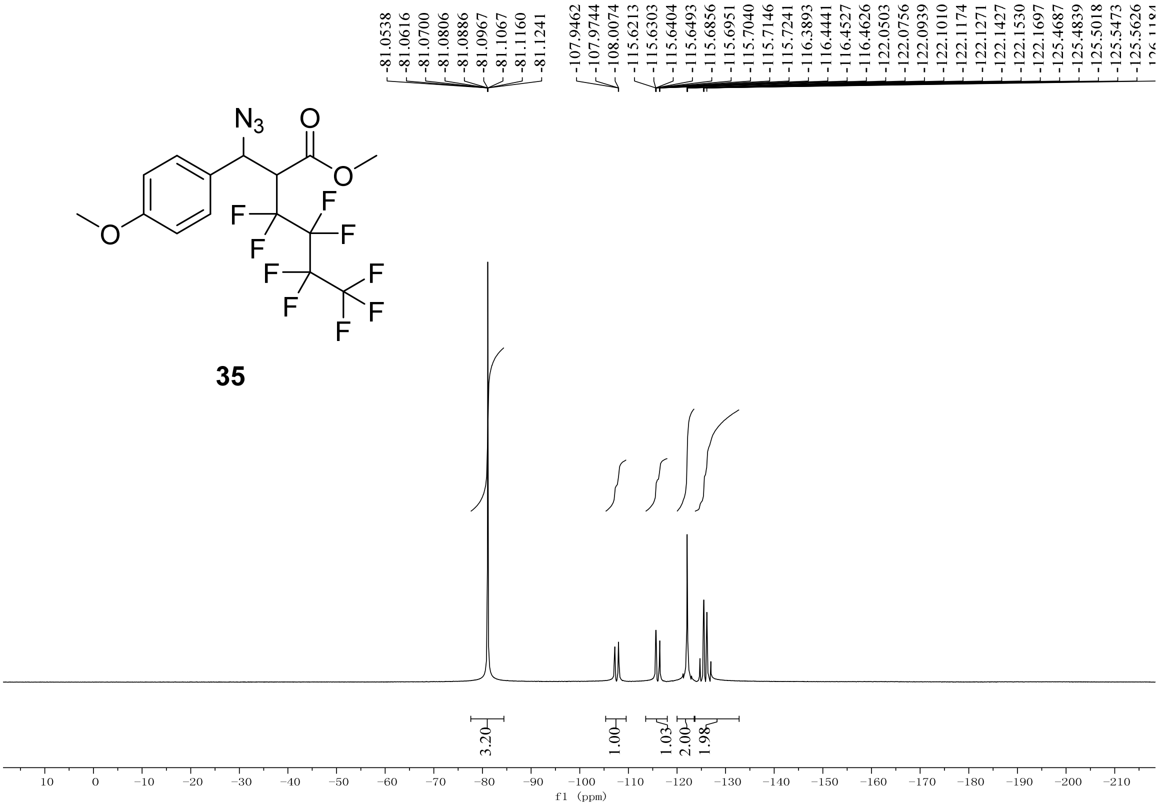

Supplementary Figure 91. ^{19}F NMR spectrum for compound **35**

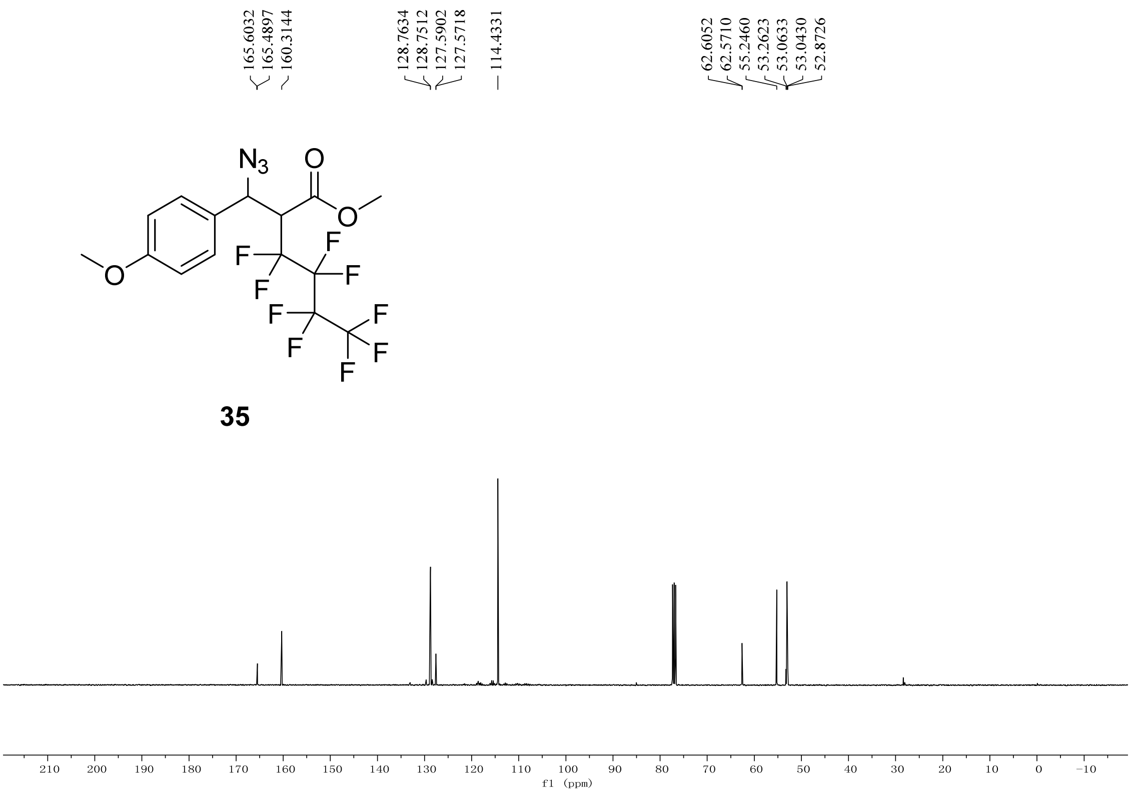

Supplementary Figure 92. ^{13}C NMR spectrum for compound **35**

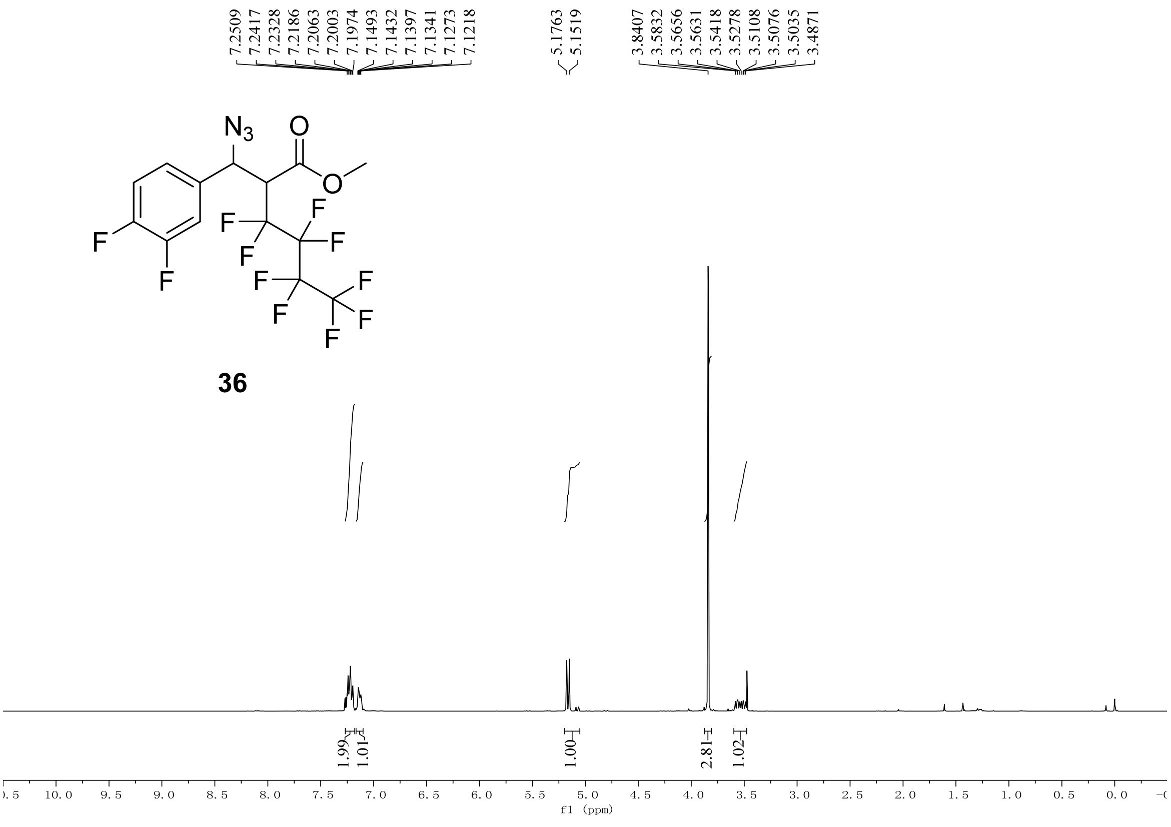

Supplementary Figure 93. ^{1}H NMR spectrum for compound **36**

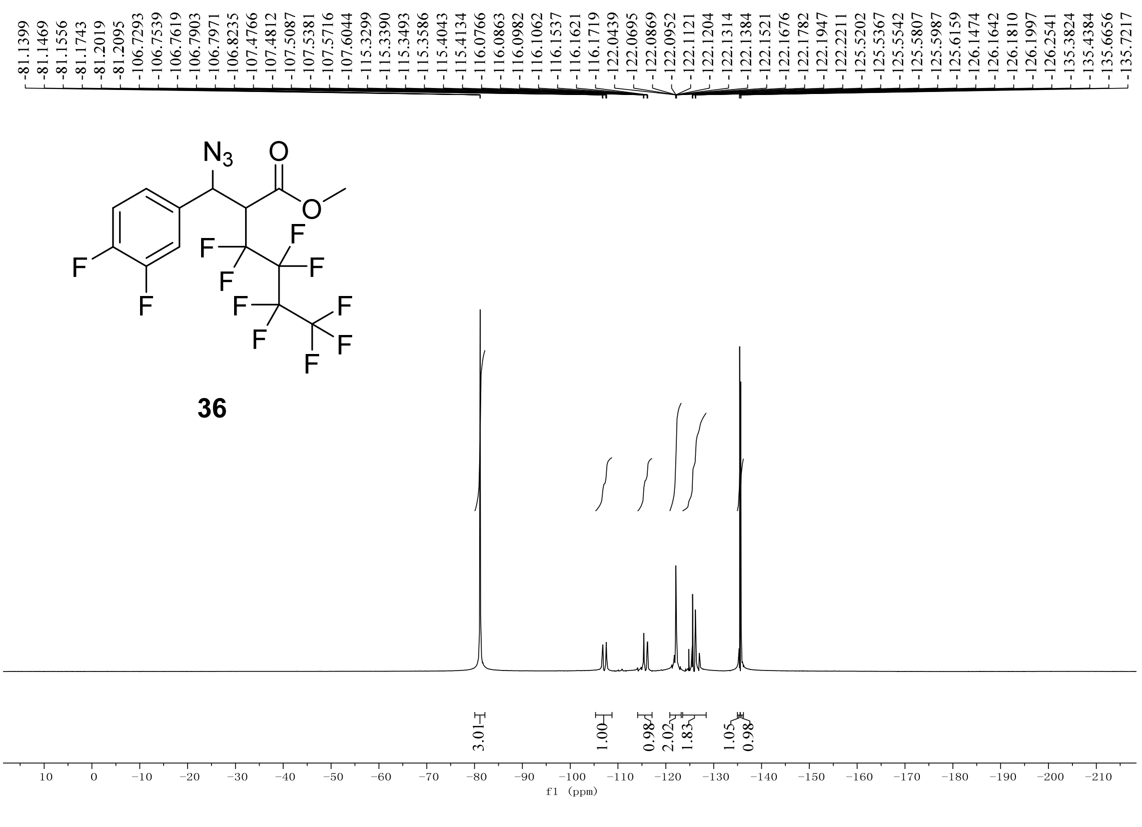

Supplementary Figure 94. ^{19}F NMR spectrum for compound **36**

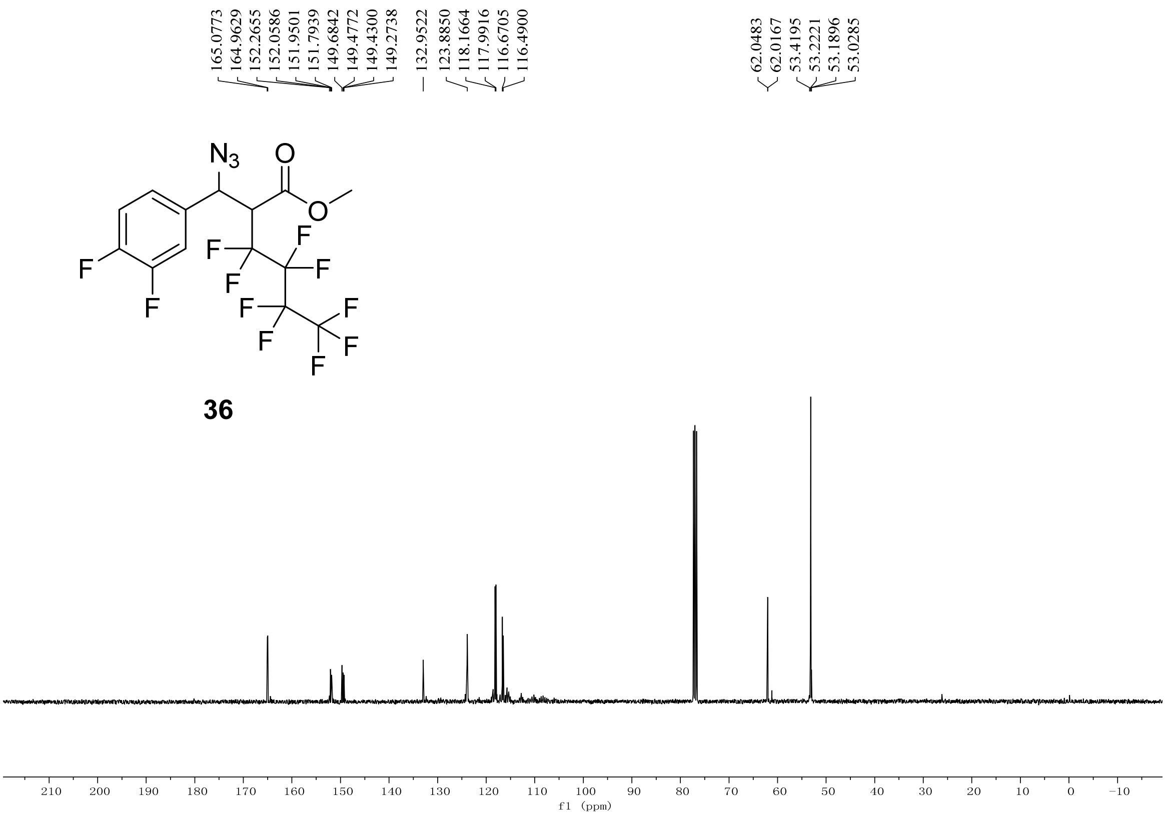

Supplementary Figure 95. ^{13}C NMR spectrum for compound **36**

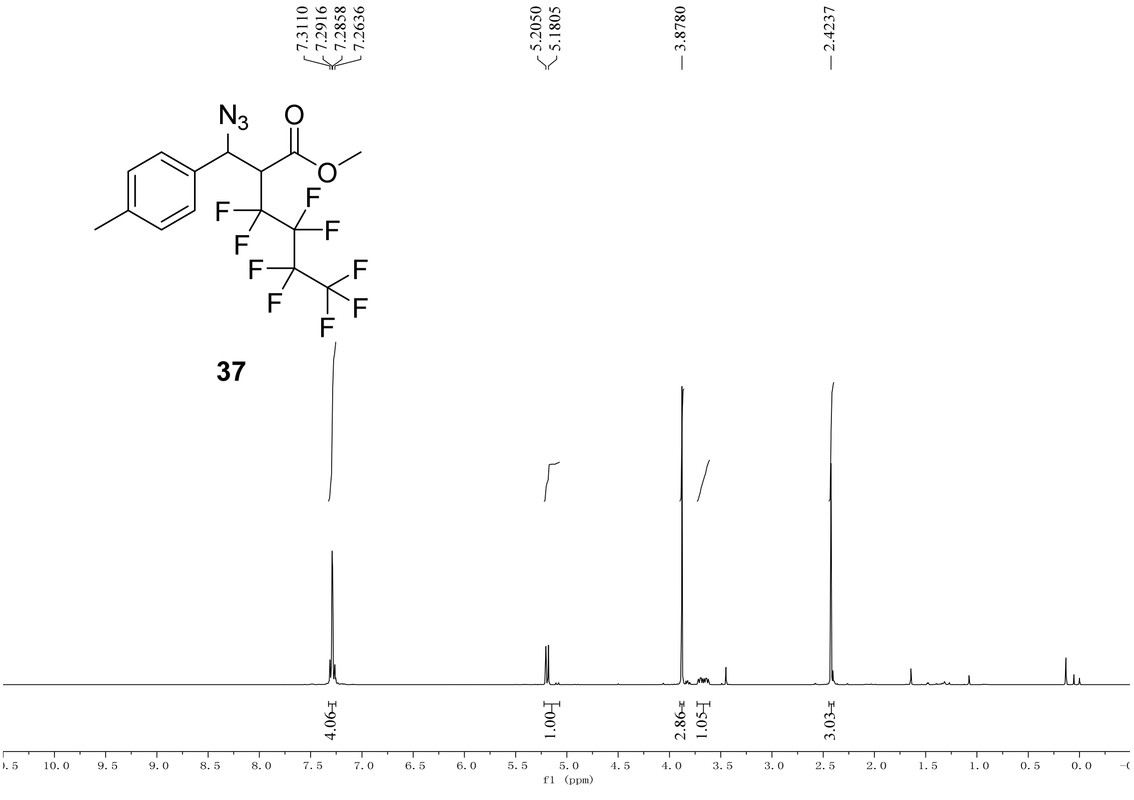

Supplementary Figure 96. ^{1}H NMR spectrum for compound **37**

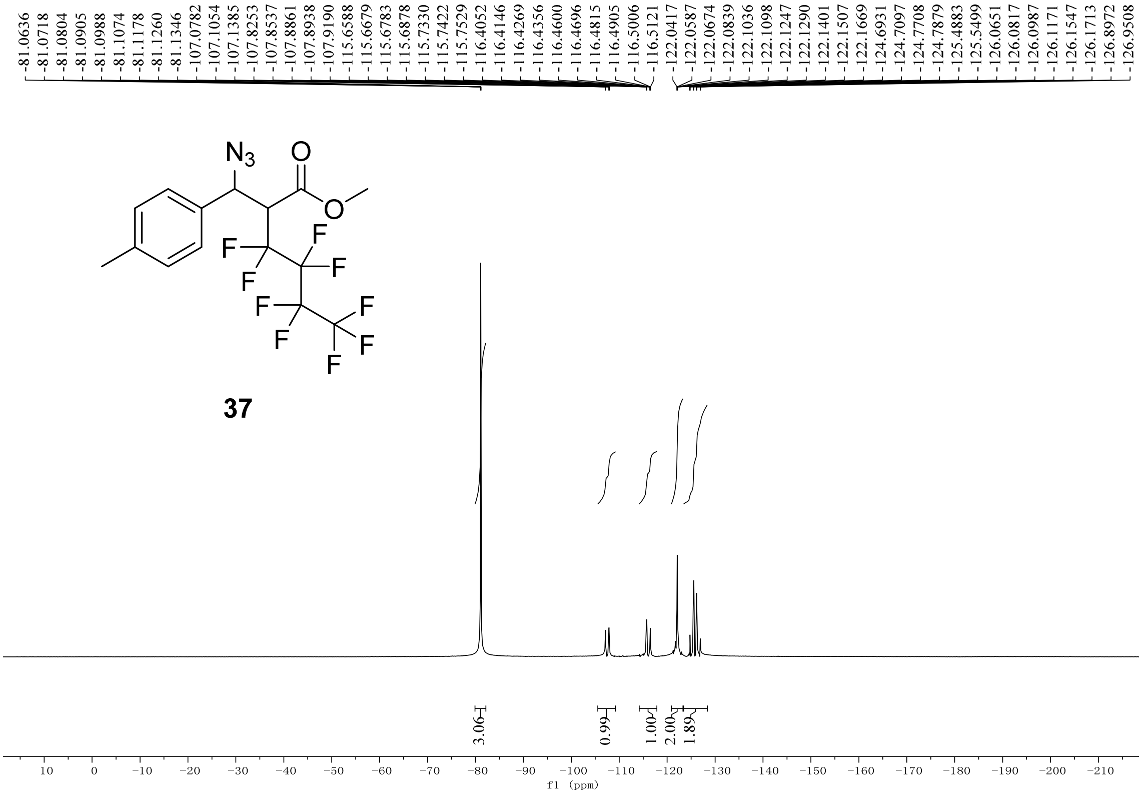

Supplementary Figure 97. ^{19}F NMR spectrum for compound **37**

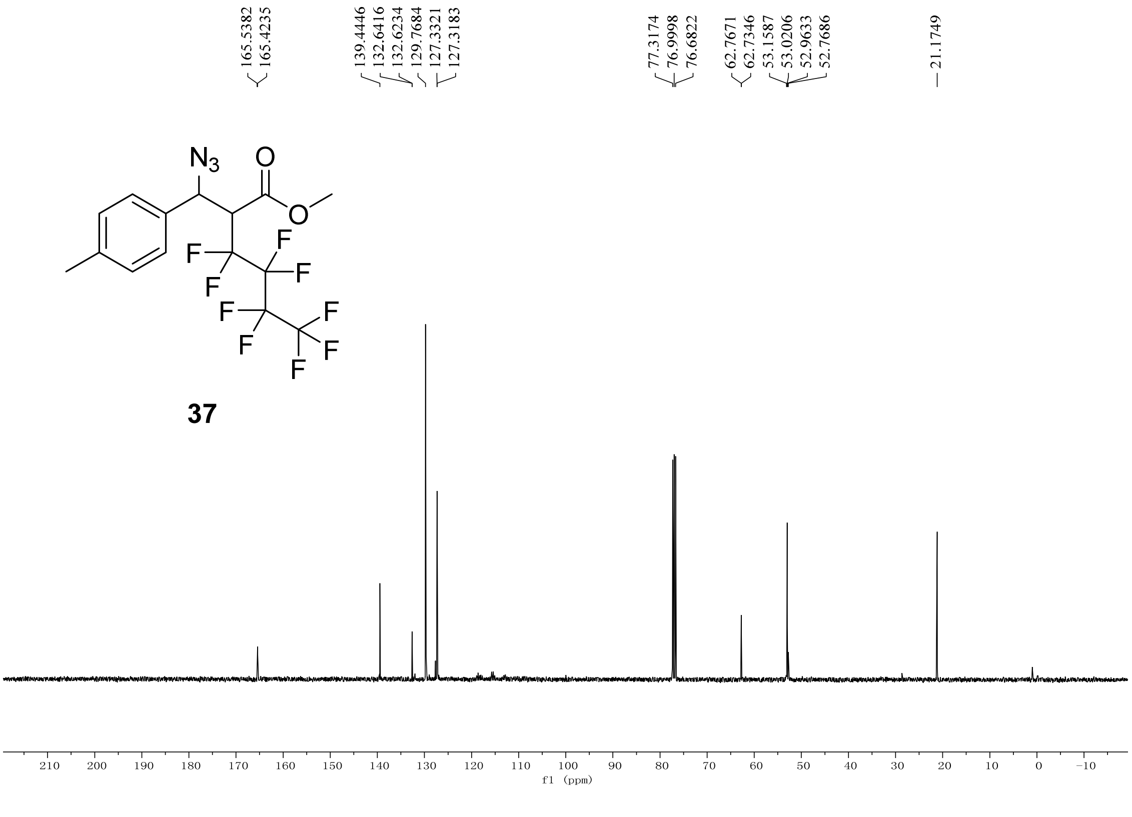

Supplementary Figure 98. ^{13}C NMR spectrum for compound **37**

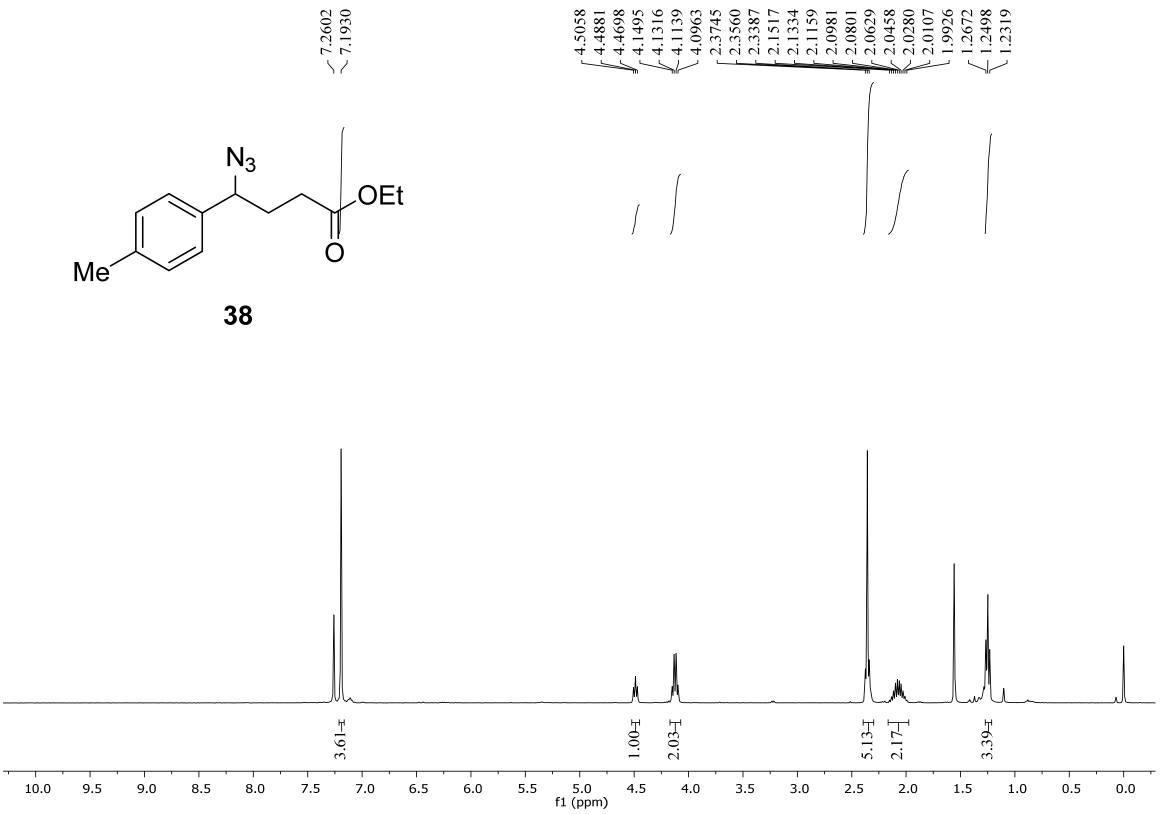

Supplementary Figure 99. ^{1}H NMR spectrum for compound **38**

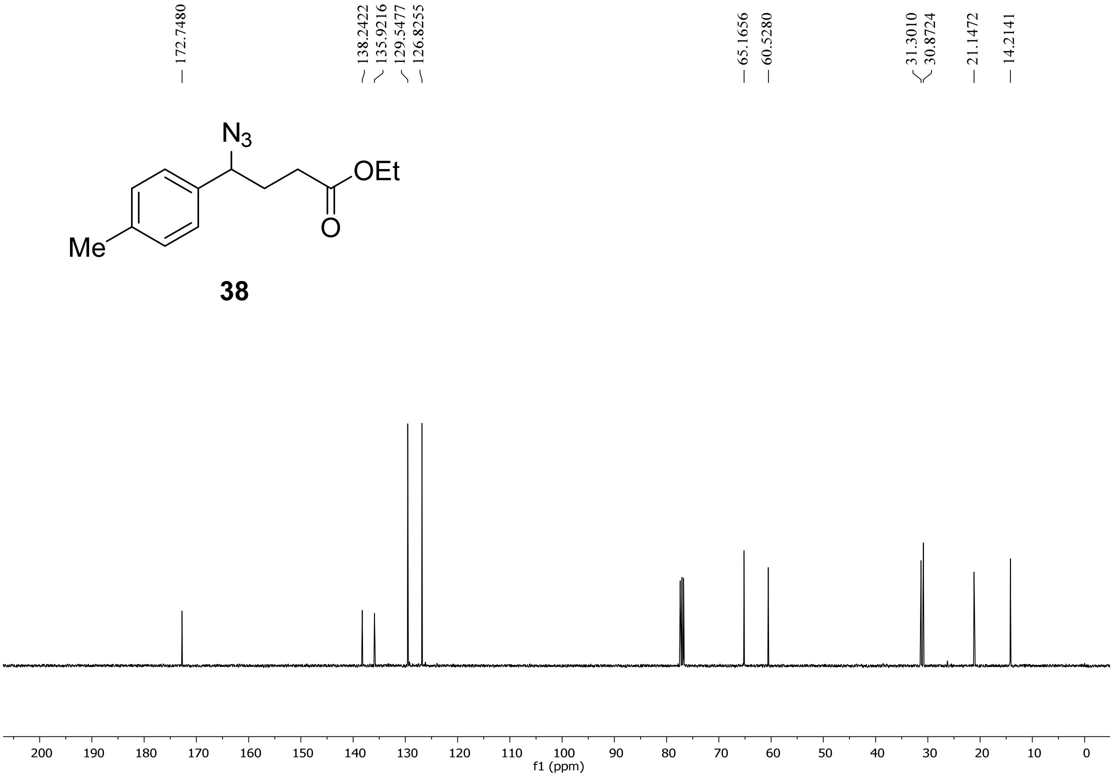

Supplementary Figure 100. ^{13}C NMR spectrum for compound **38**

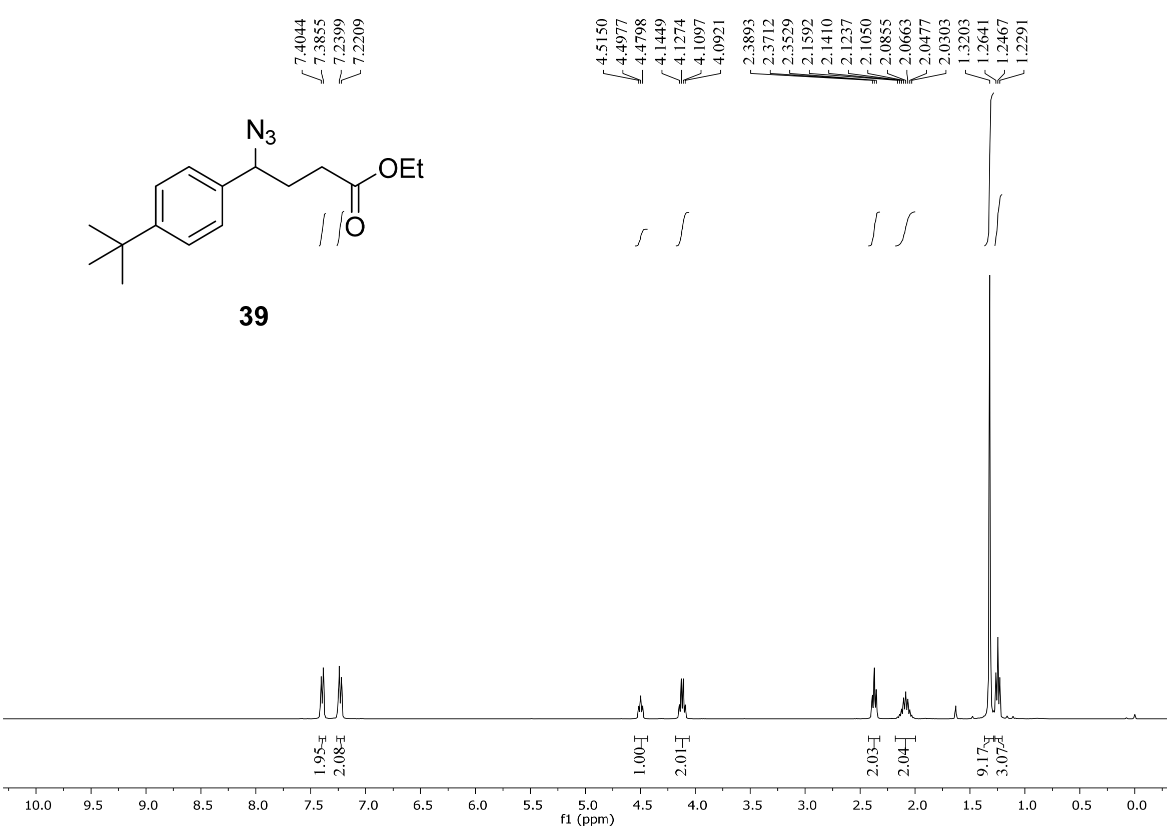

Supplementary Figure 101. ^{1}H NMR spectrum for compound **39**

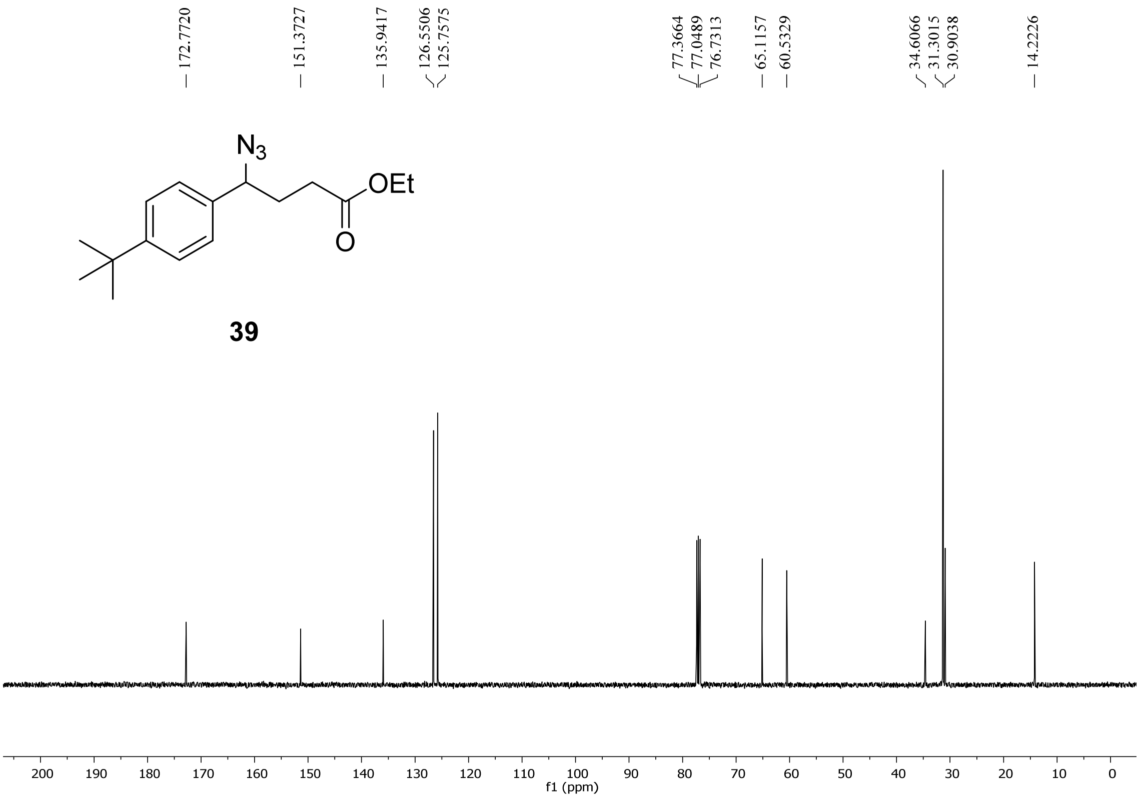

Supplementary Figure 102. ^{13}C NMR spectrum for compound **39**

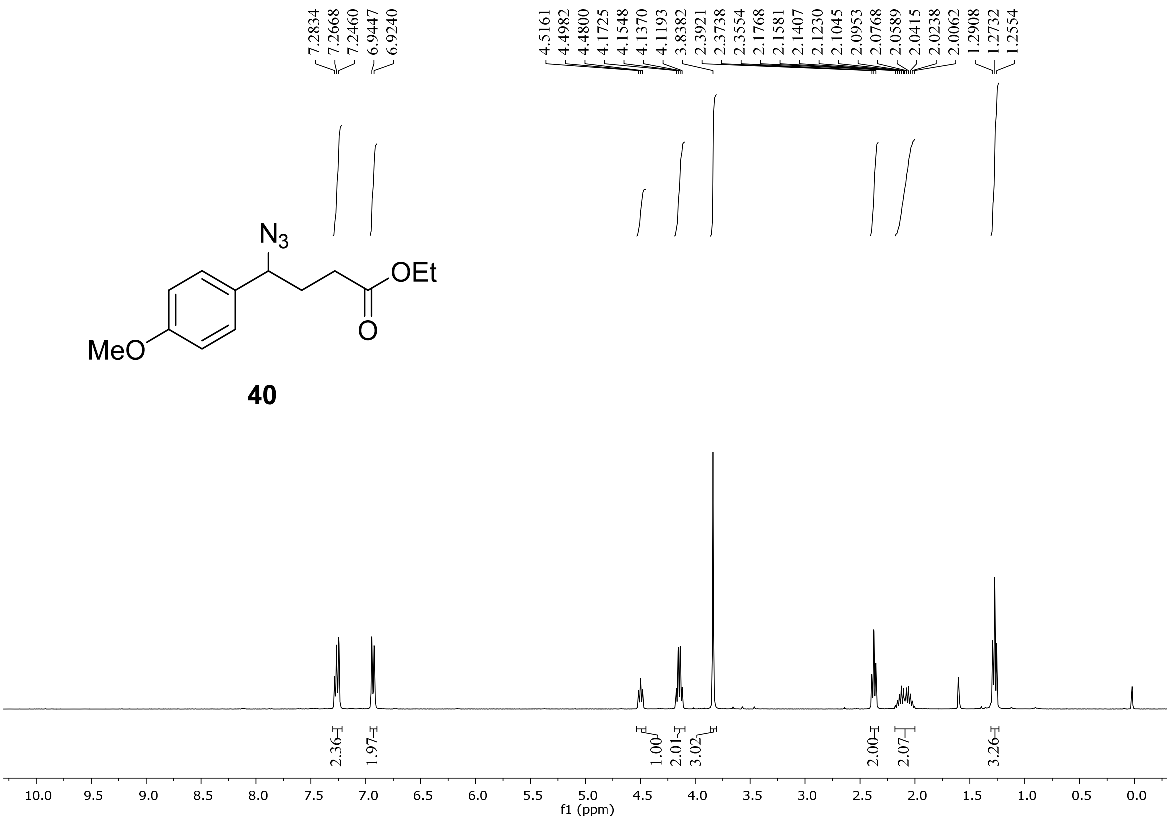

Supplementary Figure 103. ^{1}H NMR spectrum for compound **40**

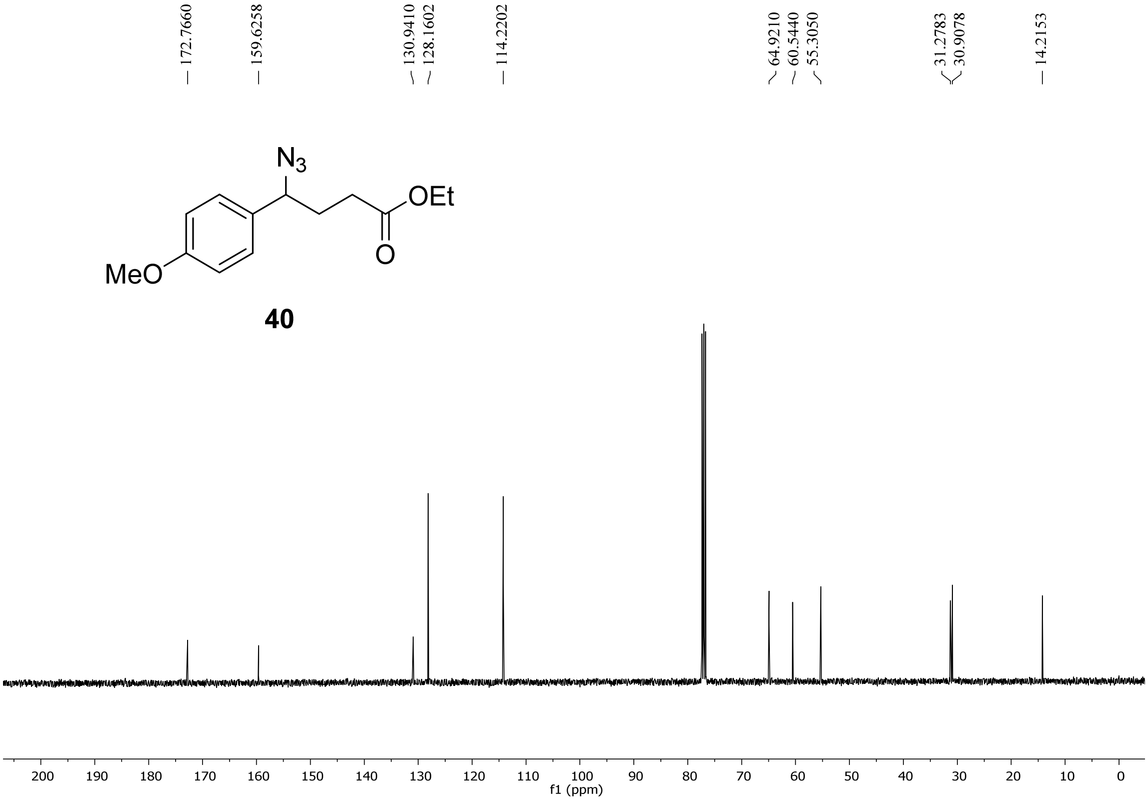

Supplementary Figure 104. ^{13}C NMR spectrum for compound **40**

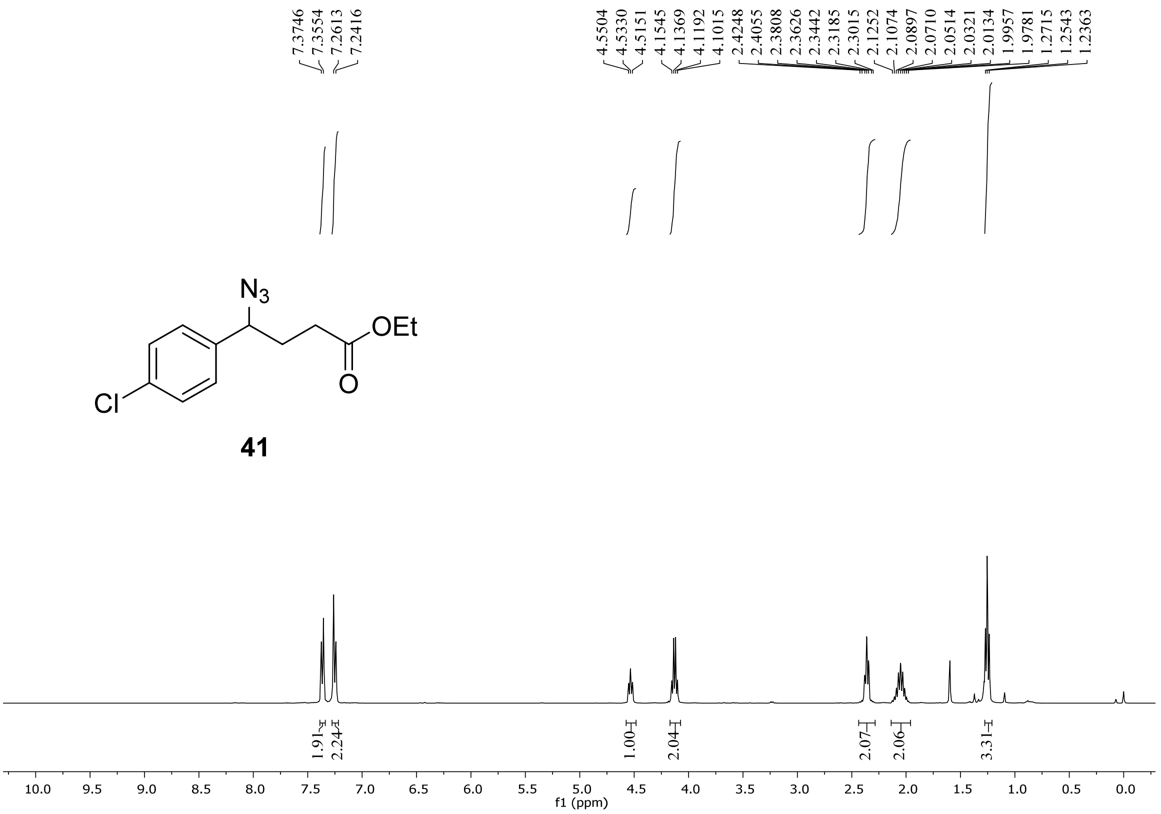

Supplementary Figure 105. ^{1}H NMR spectrum for compound **41**

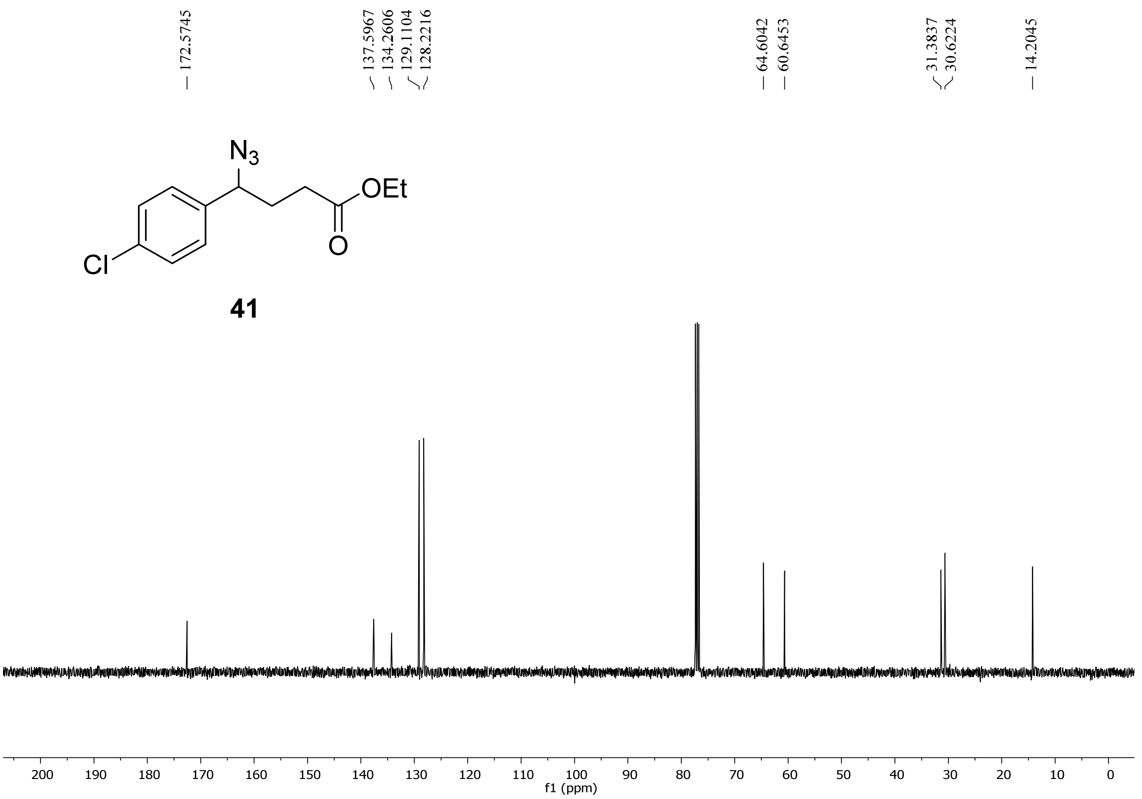

Supplementary Figure 106. ^{13}C NMR spectrum for compound **41**

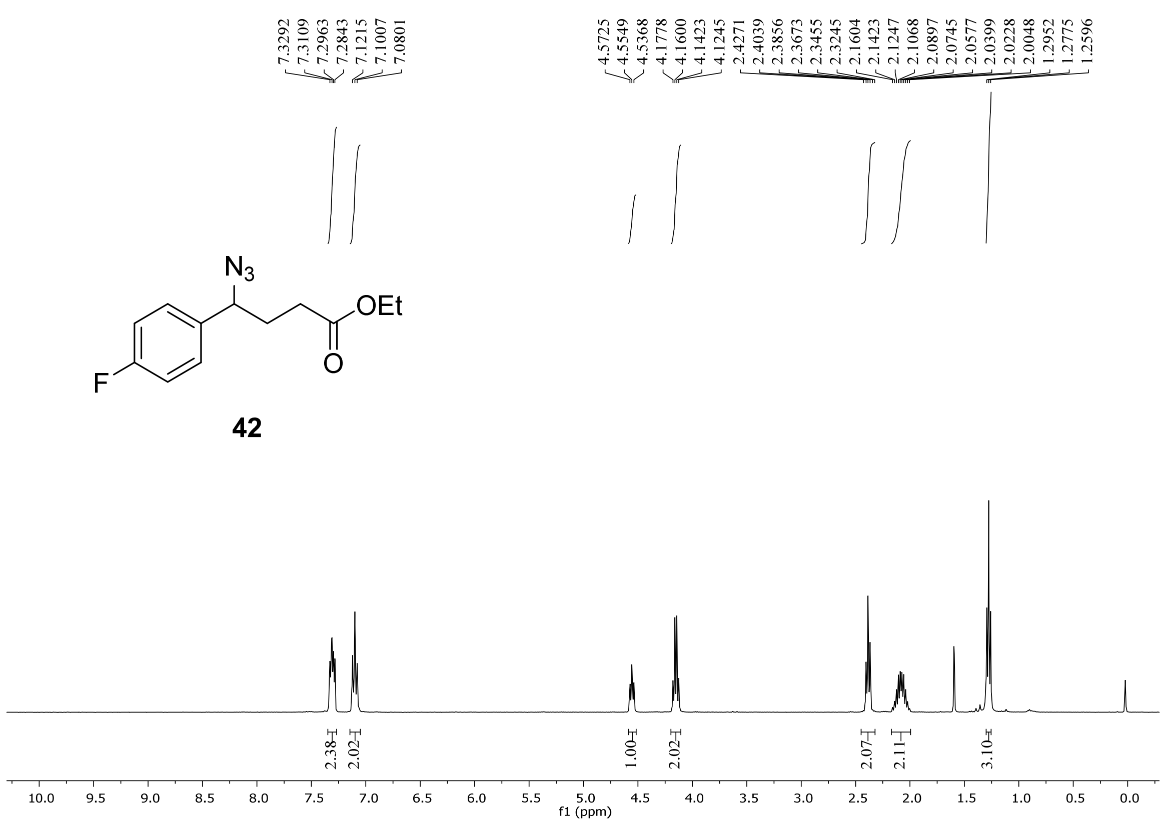

Supplementary Figure 107. ^{1}H NMR spectrum for compound **42**

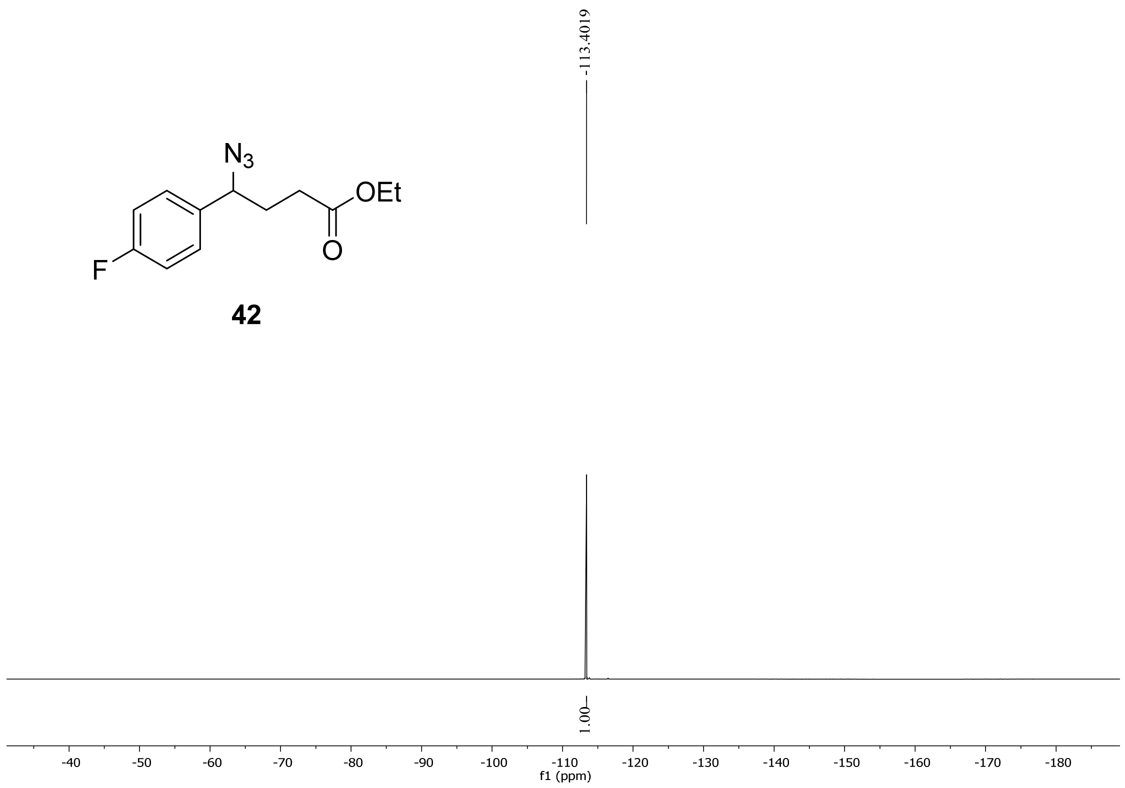

Supplementary Figure 108. ^{19}F NMR spectrum for compound **42**

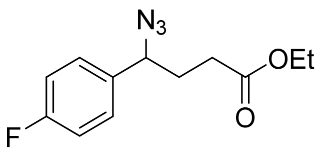

42

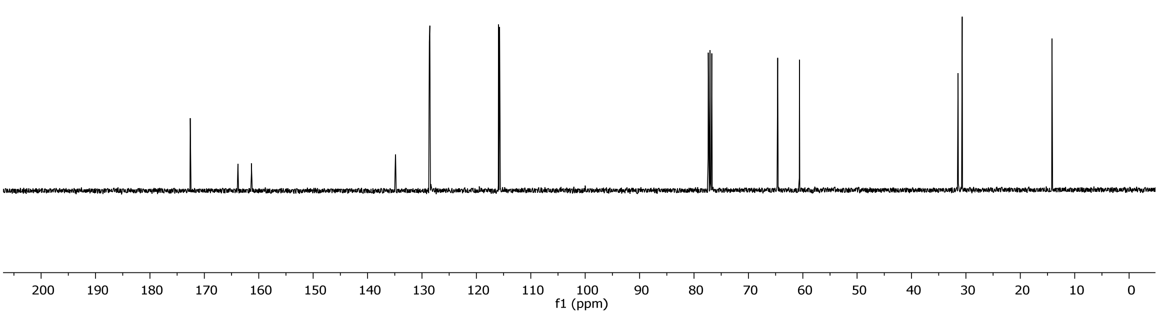

Supplementary Figure 109. ^{13}C NMR spectrum for compound **42**

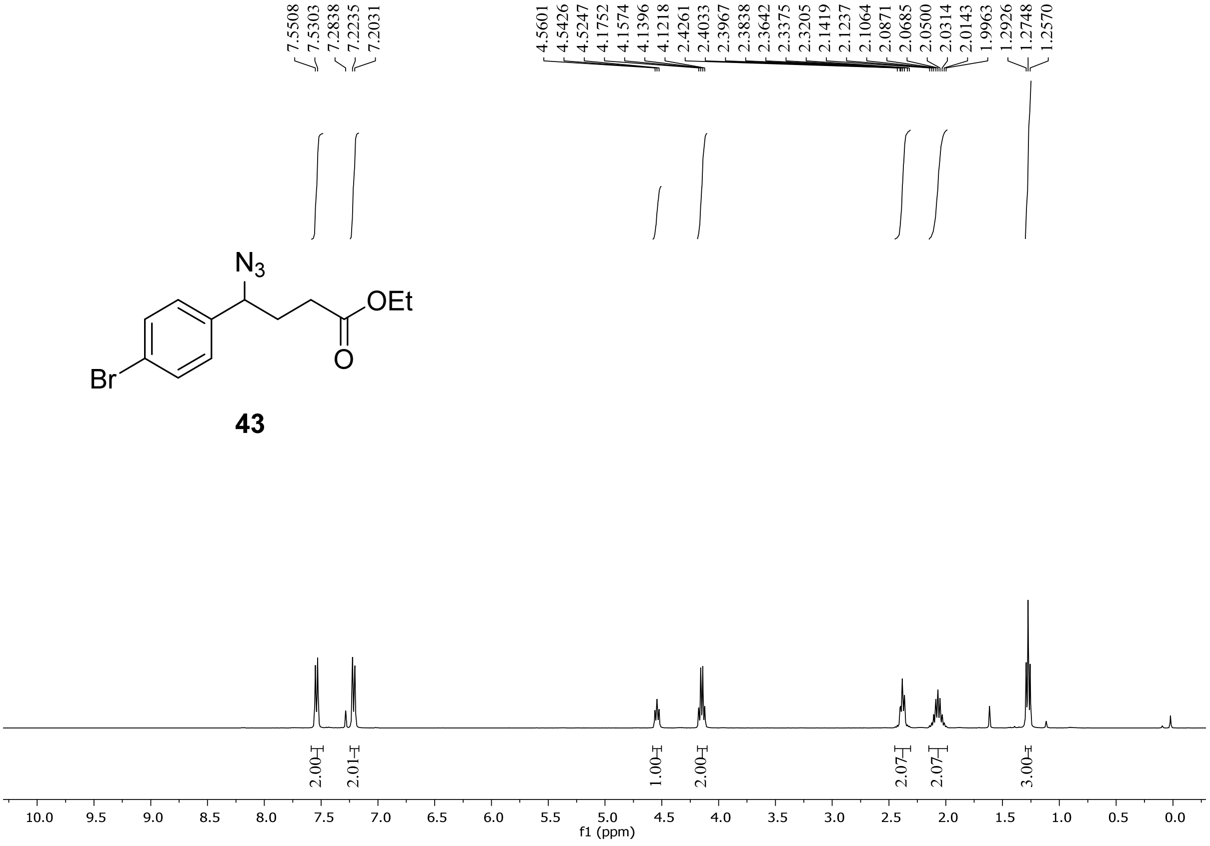

Supplementary Figure 110. ^{1}H NMR spectrum for compound **43**

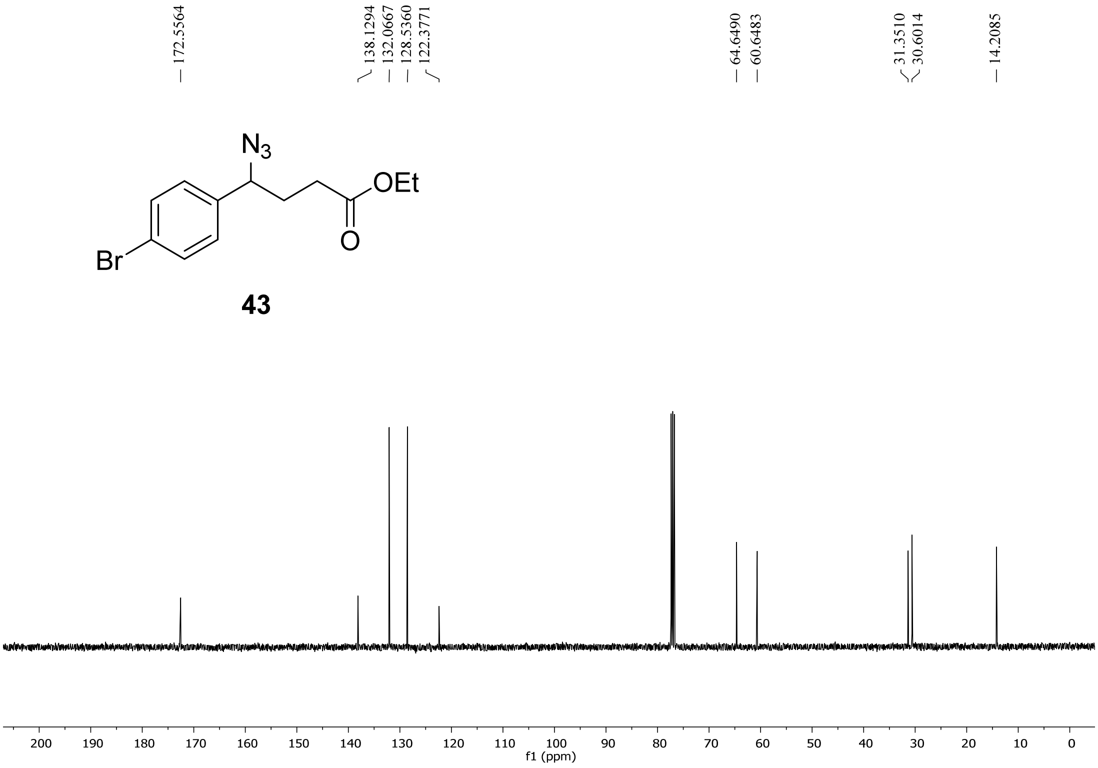

Supplementary Figure 111. ^{13}C NMR spectrum for compound **43**

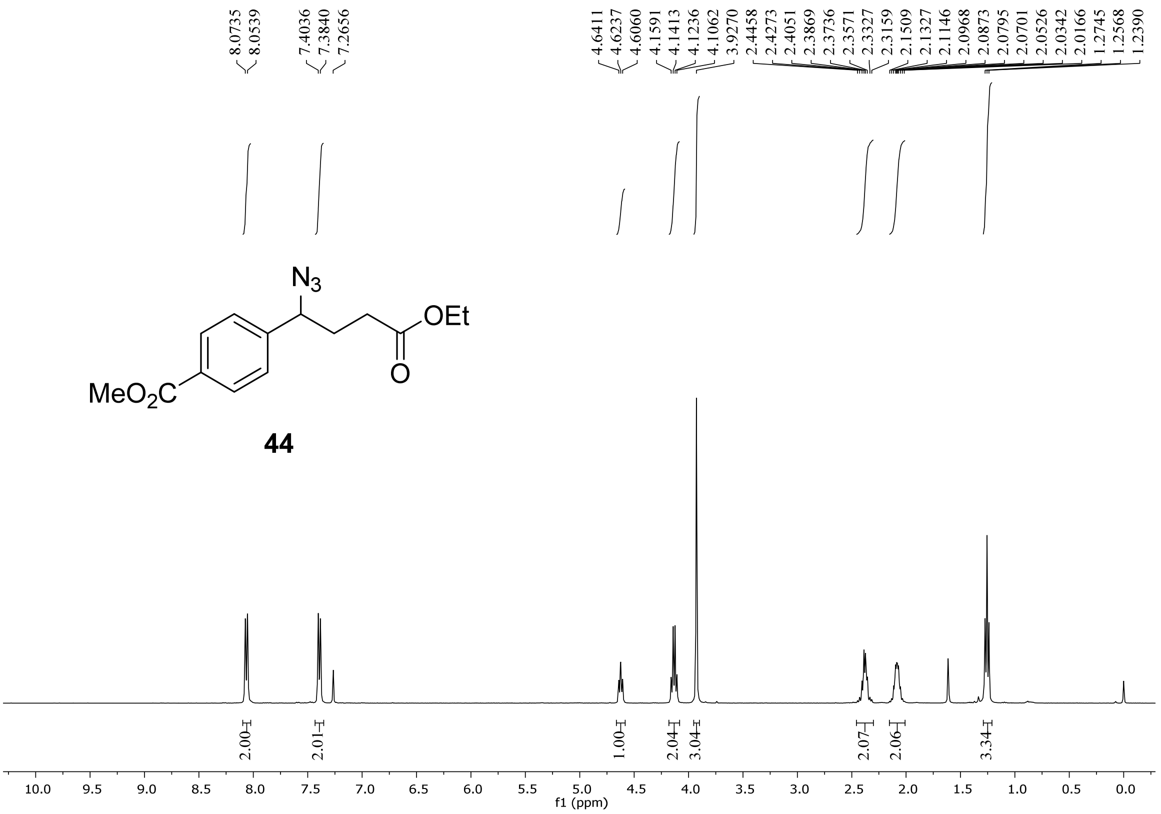

Supplementary Figure 112. ^{1}H NMR spectrum for compound **44**

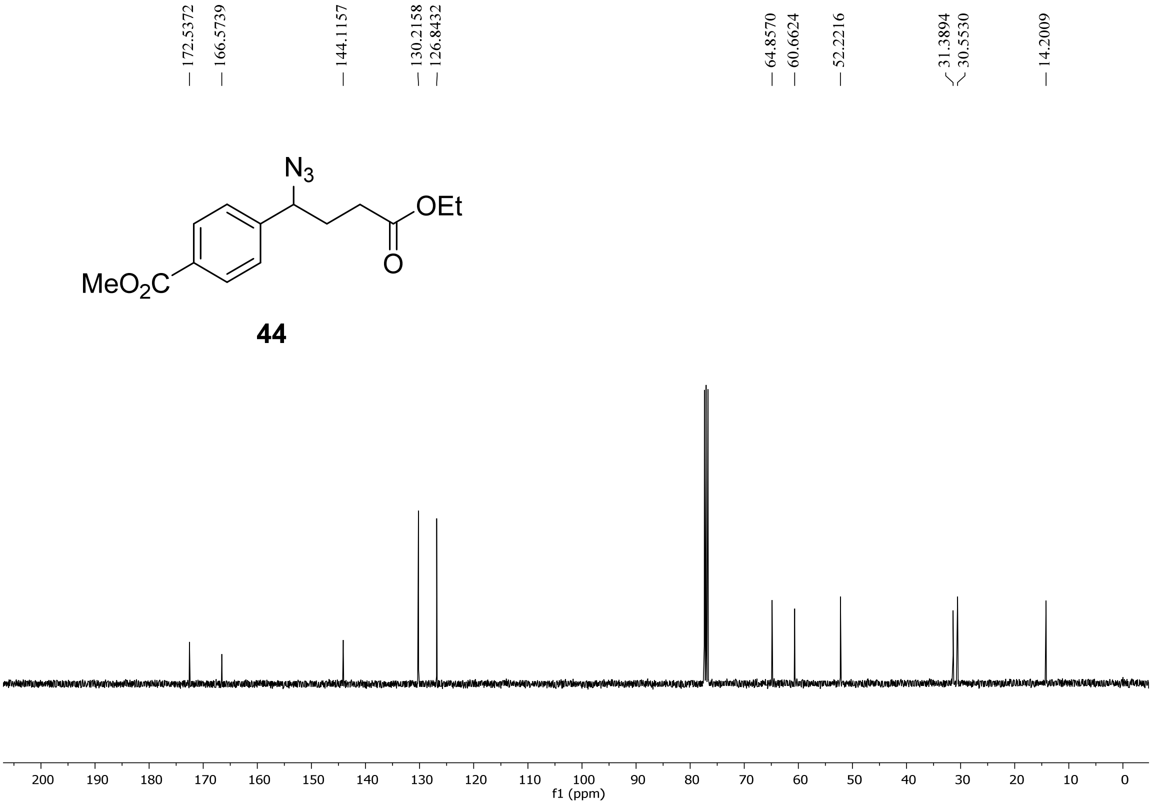

Supplementary Figure 113. ^{13}C NMR spectrum for compound **44**

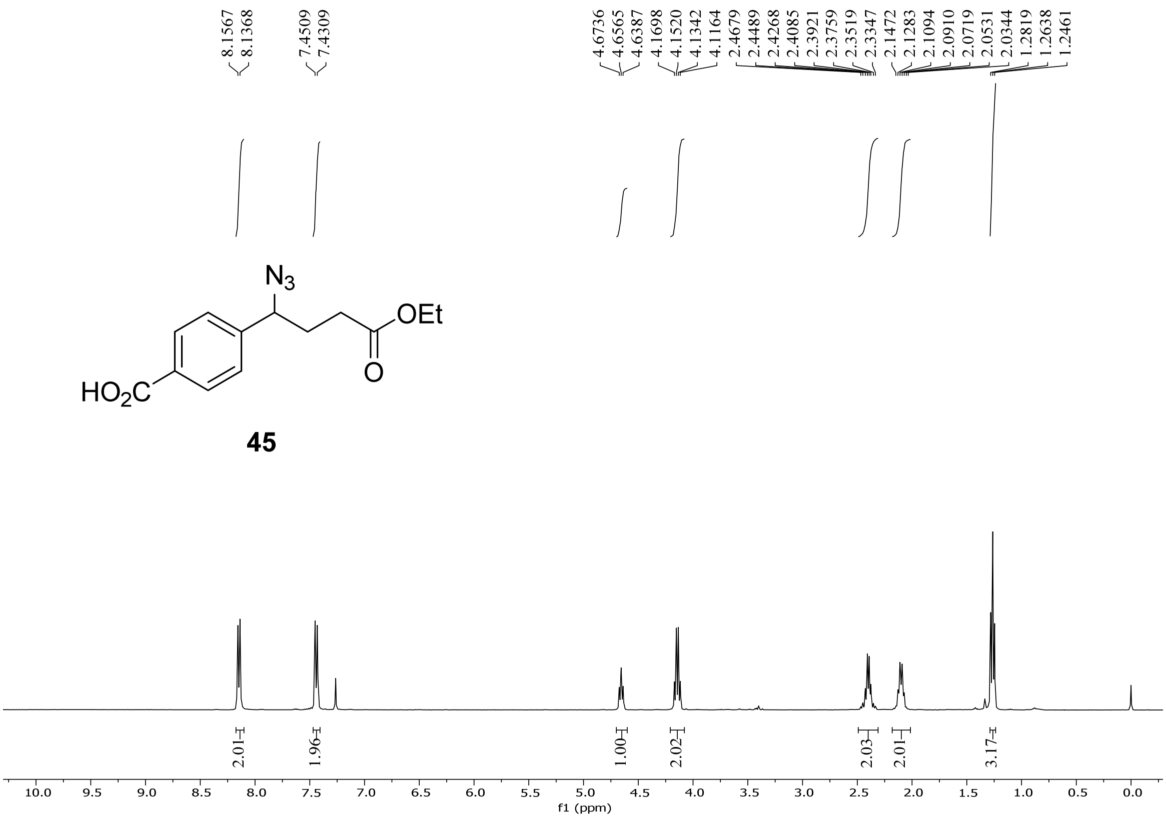

Supplementary Figure 114. ^{1}H NMR spectrum for compound **45**

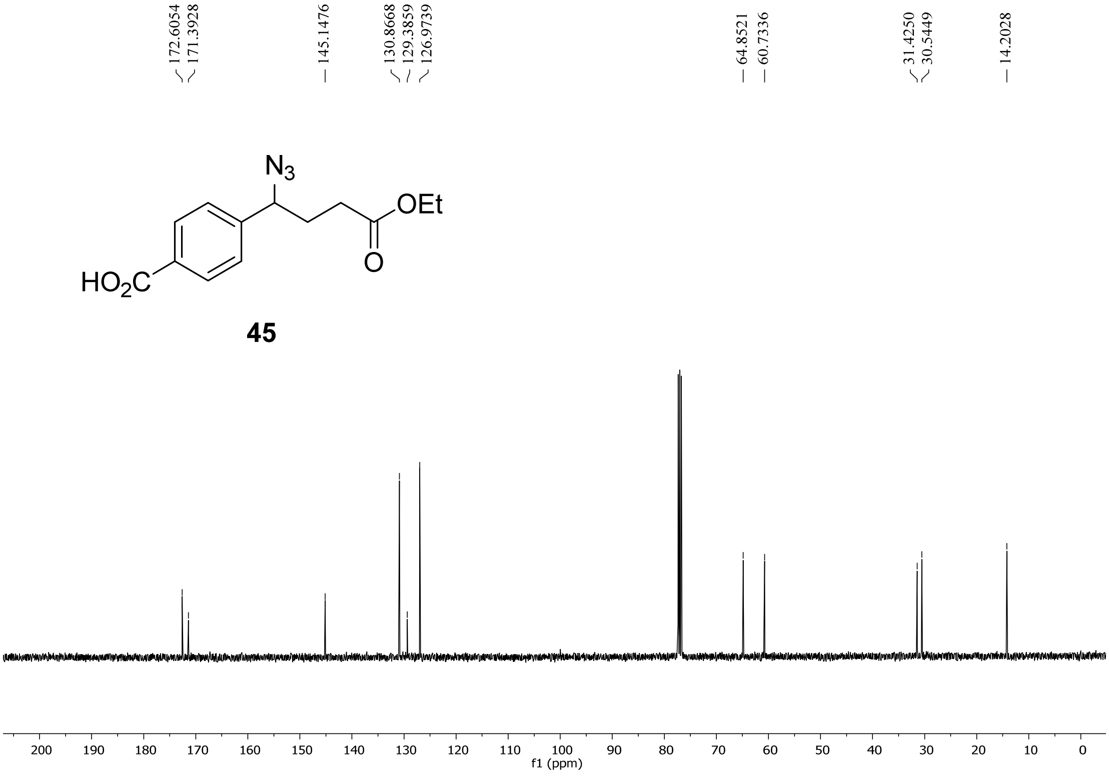

Supplementary Figure 115. ^{13}C NMR spectrum for compound **45**

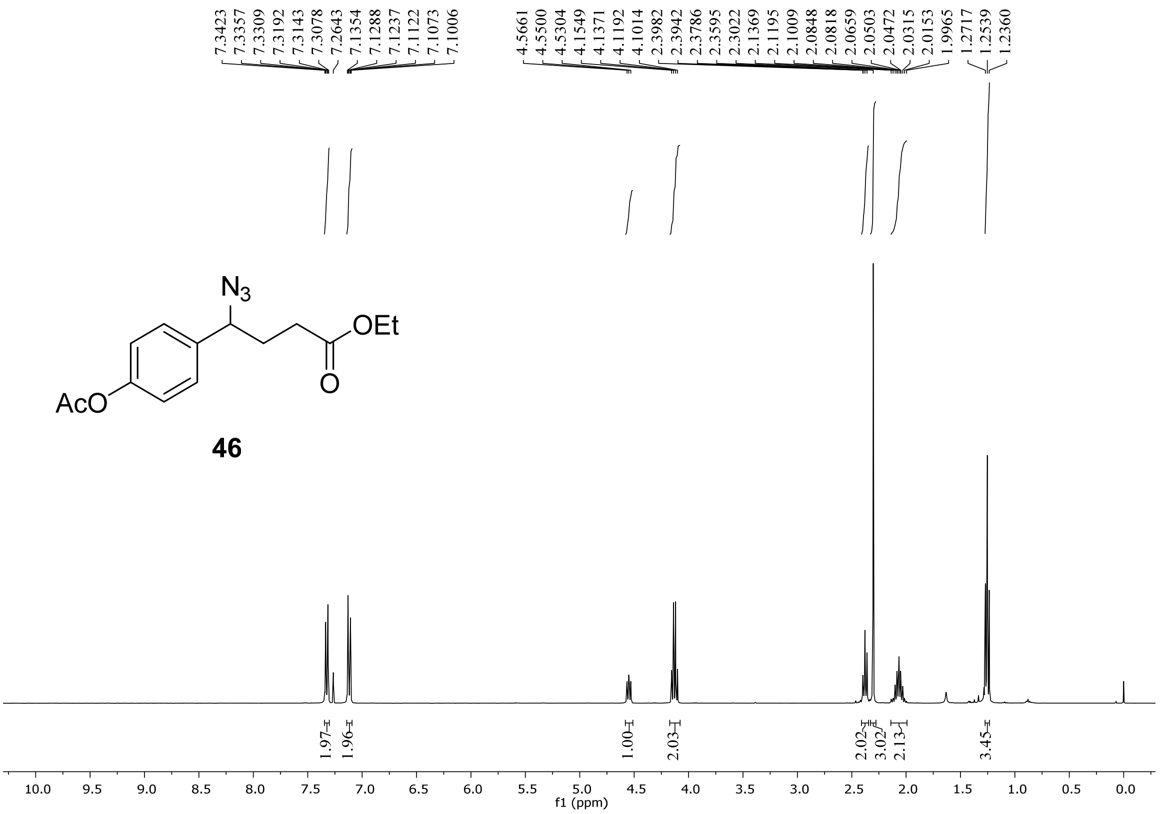

Supplementary Figure 116. ^{1}H NMR spectrum for compound **46**

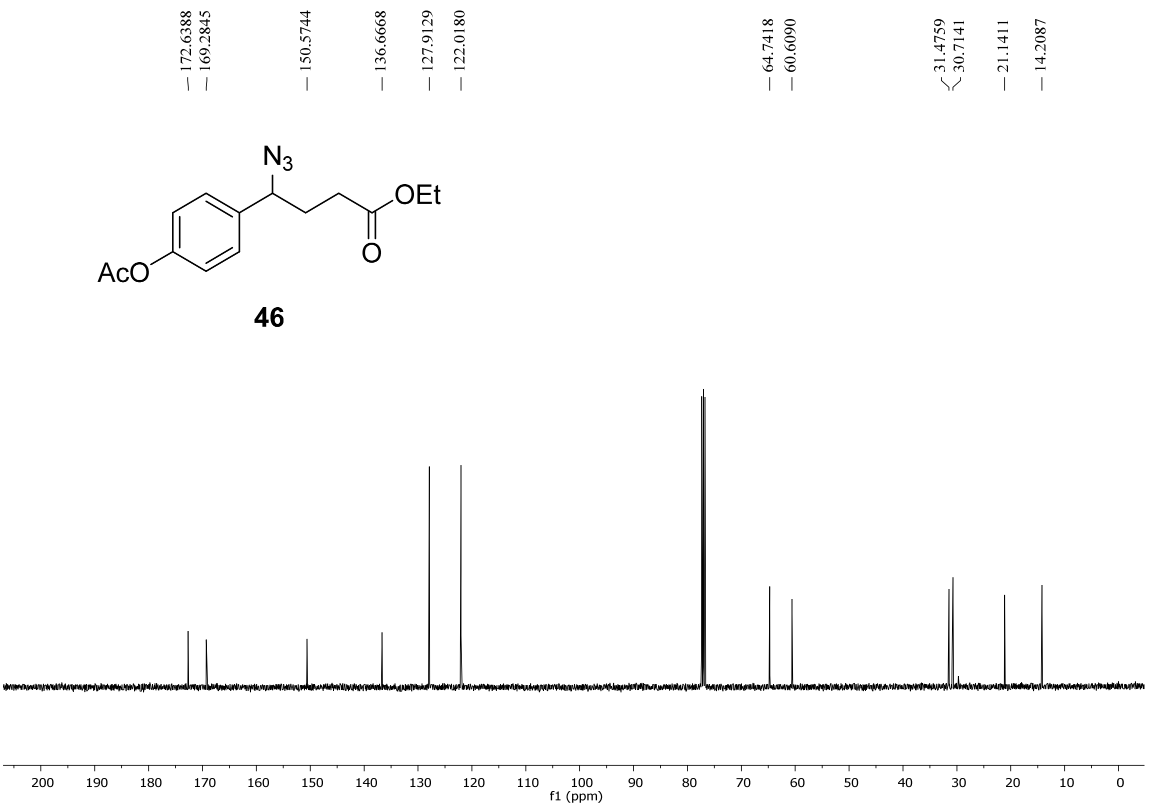

Supplementary Figure 117. ^{13}C NMR spectrum for compound **46**

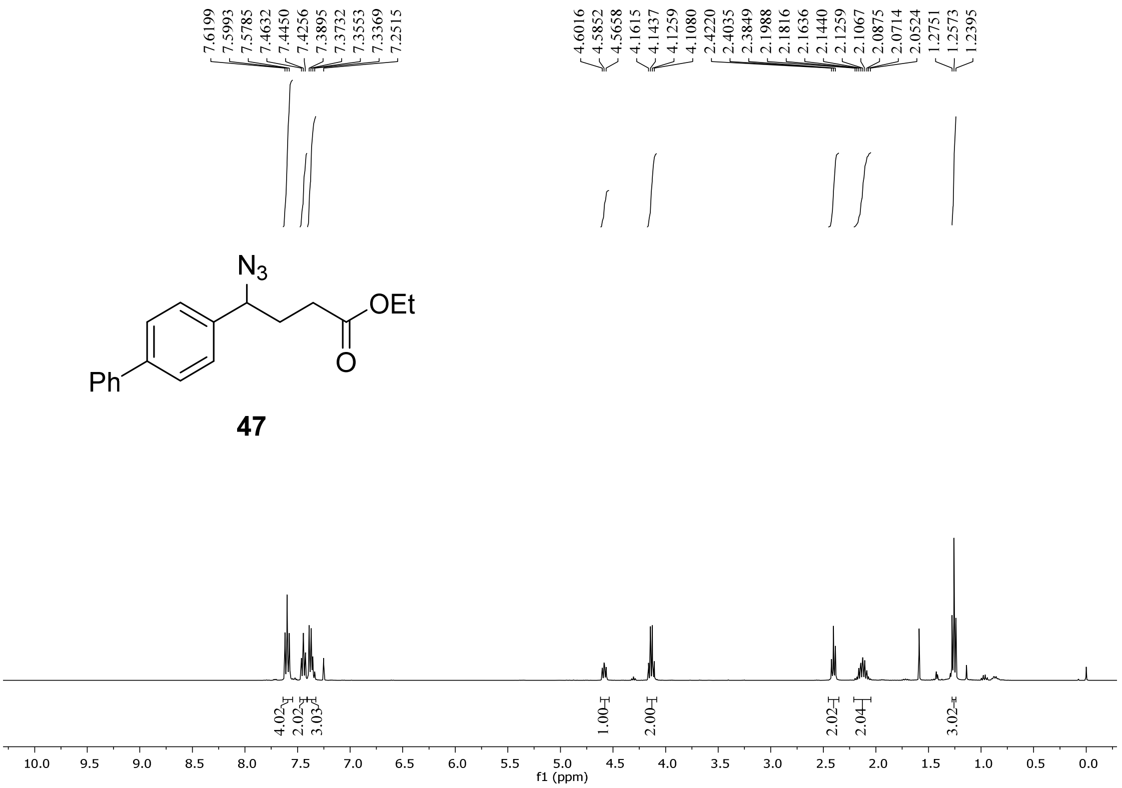

Supplementary Figure 118. ^{1}H NMR spectrum for compound **47**

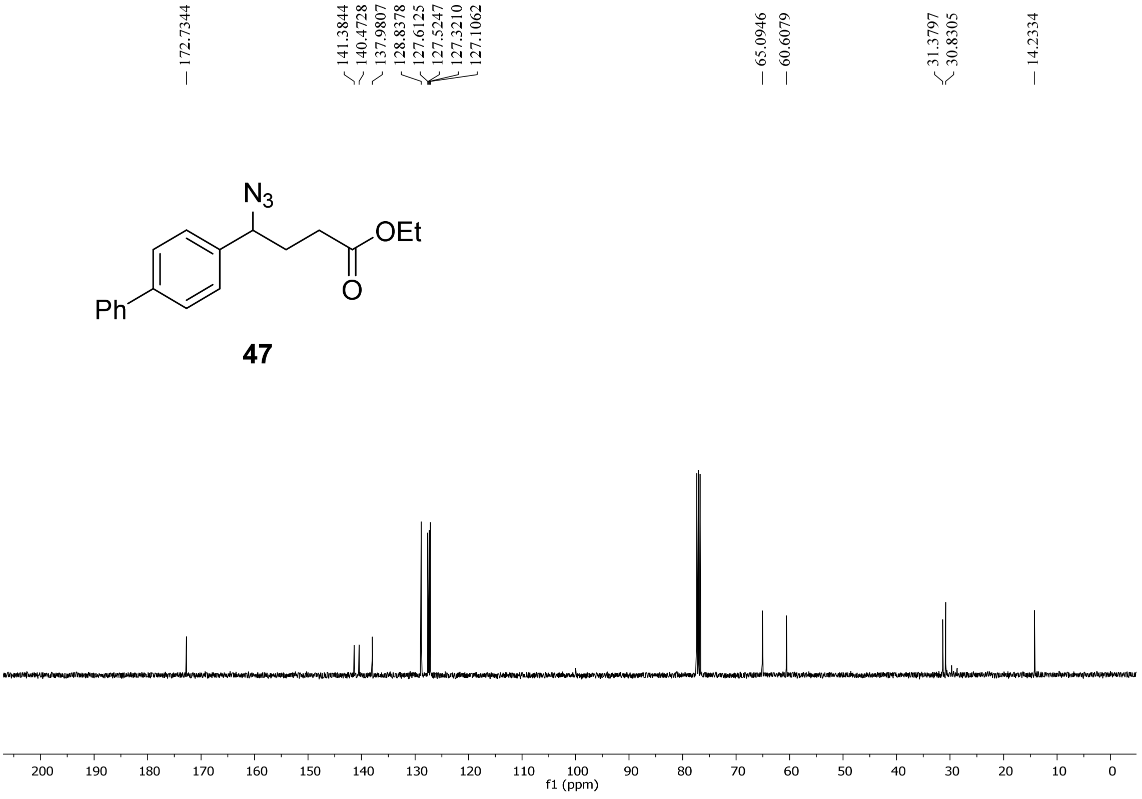

Supplementary Figure 119. ^{13}C NMR spectrum for compound **47**

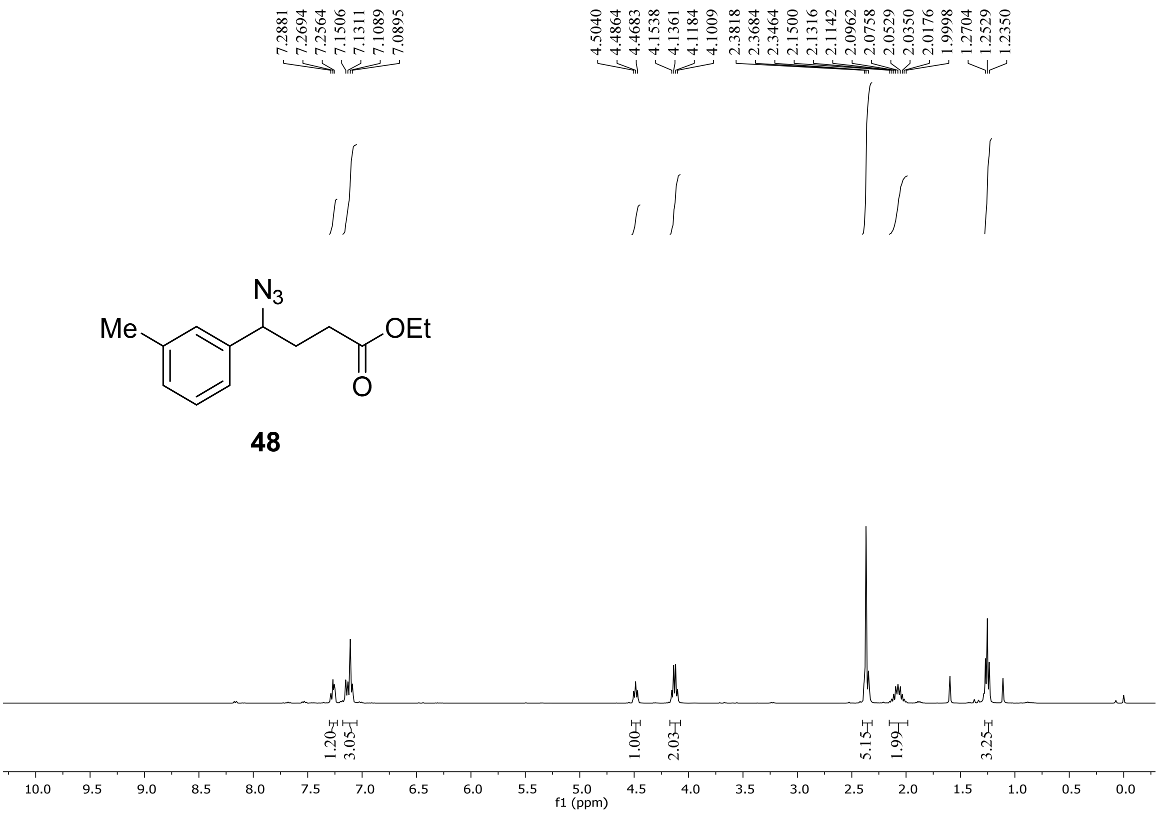

Supplementary Figure 120. ^{1}H NMR spectrum for compound **48**

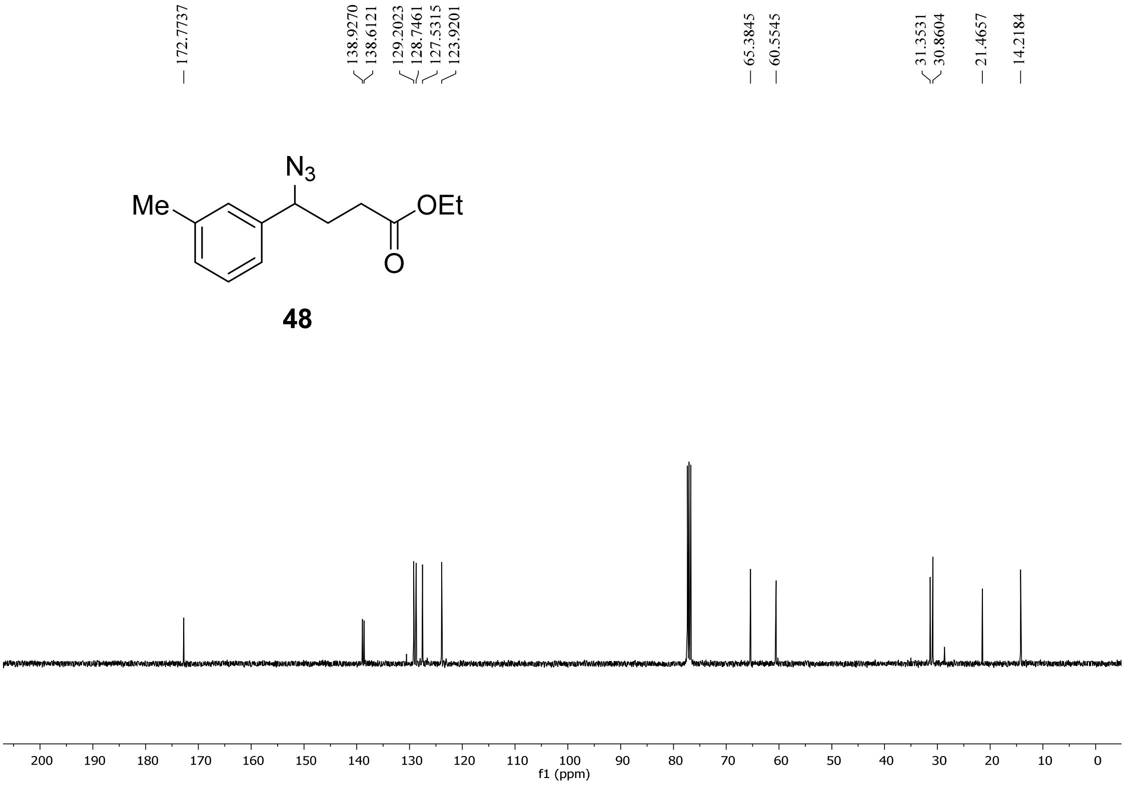

Supplementary Figure 121. ^{13}C NMR spectrum for compound **48**

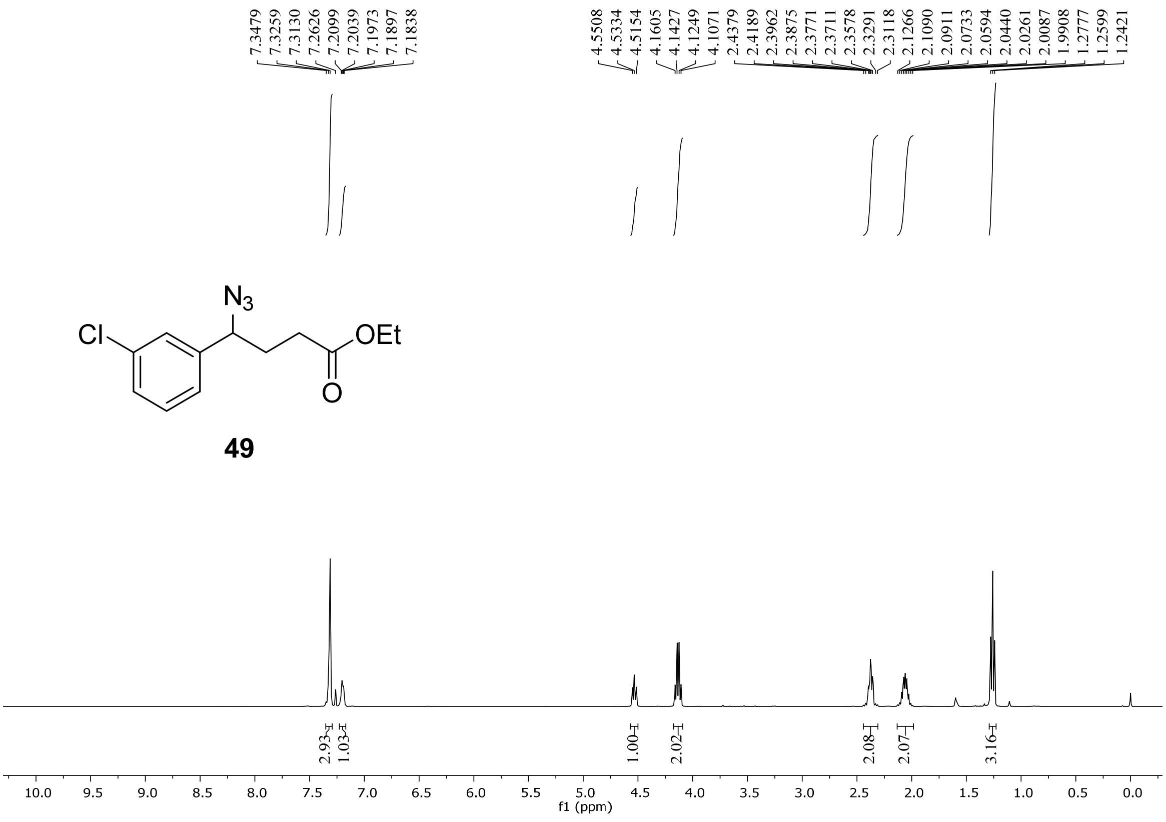

Supplementary Figure 122. ^{1}H NMR spectrum for compound **49**

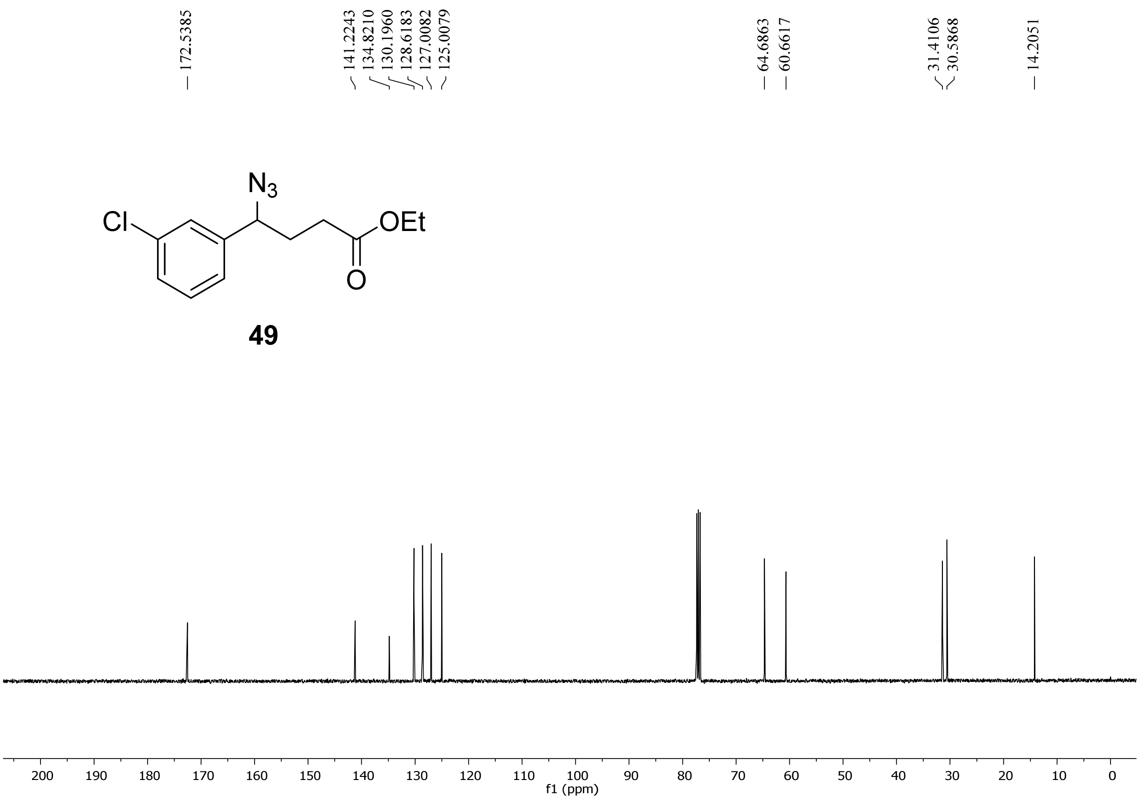

Supplementary Figure 123. ^{13}C NMR spectrum for compound **49**

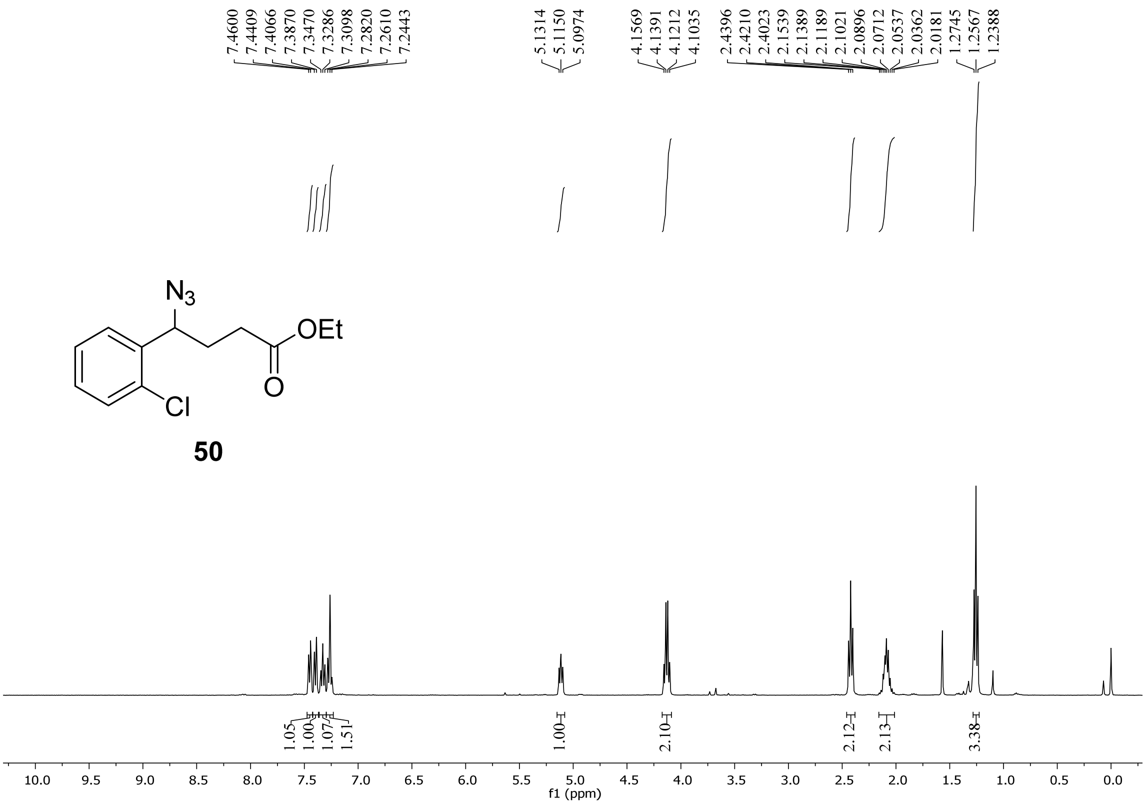

Supplementary Figure 124. ^{1}H NMR spectrum for compound **50**

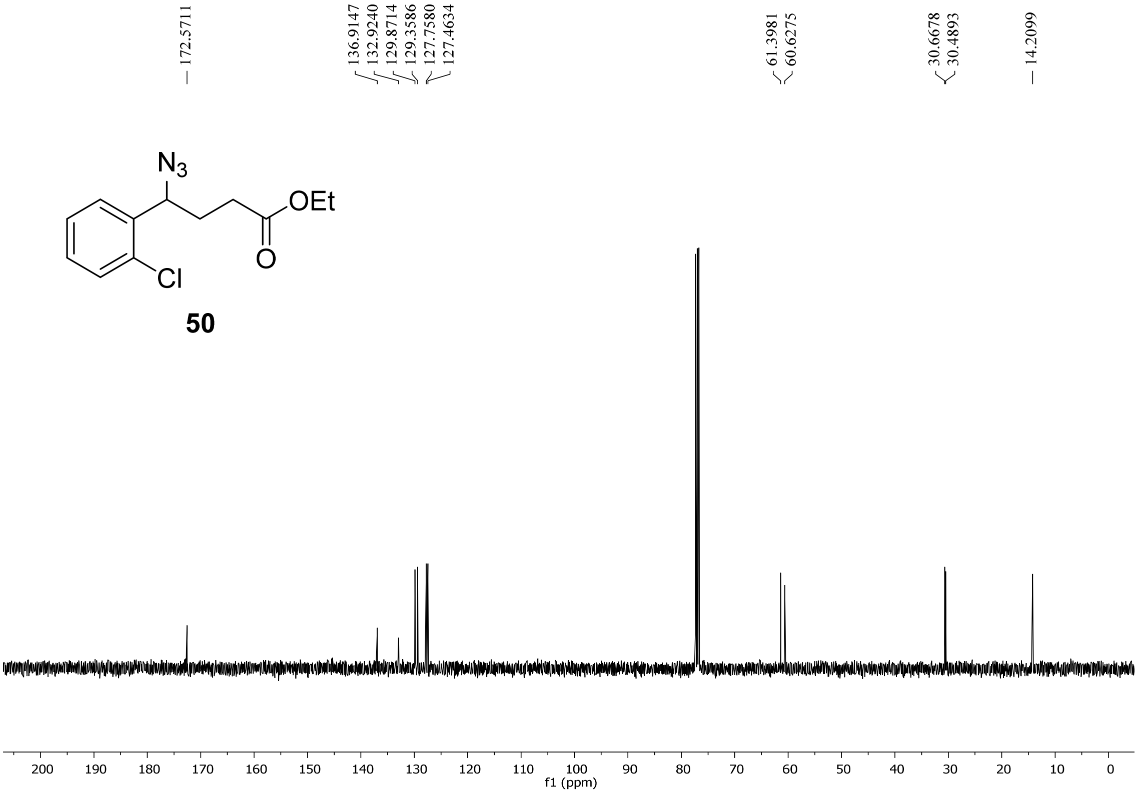

Supplementary Figure 125. ^{13}C NMR spectrum for compound **50**

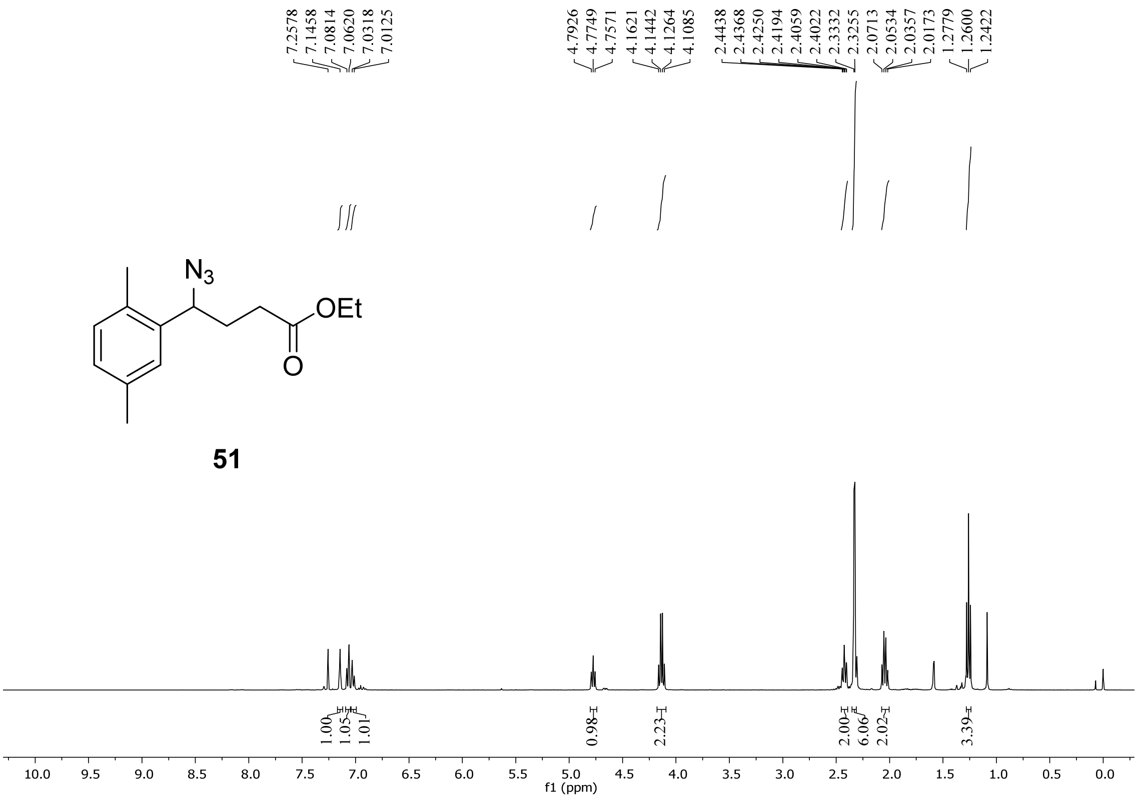

Supplementary Figure 126. ^{1}H NMR spectrum for compound **51**

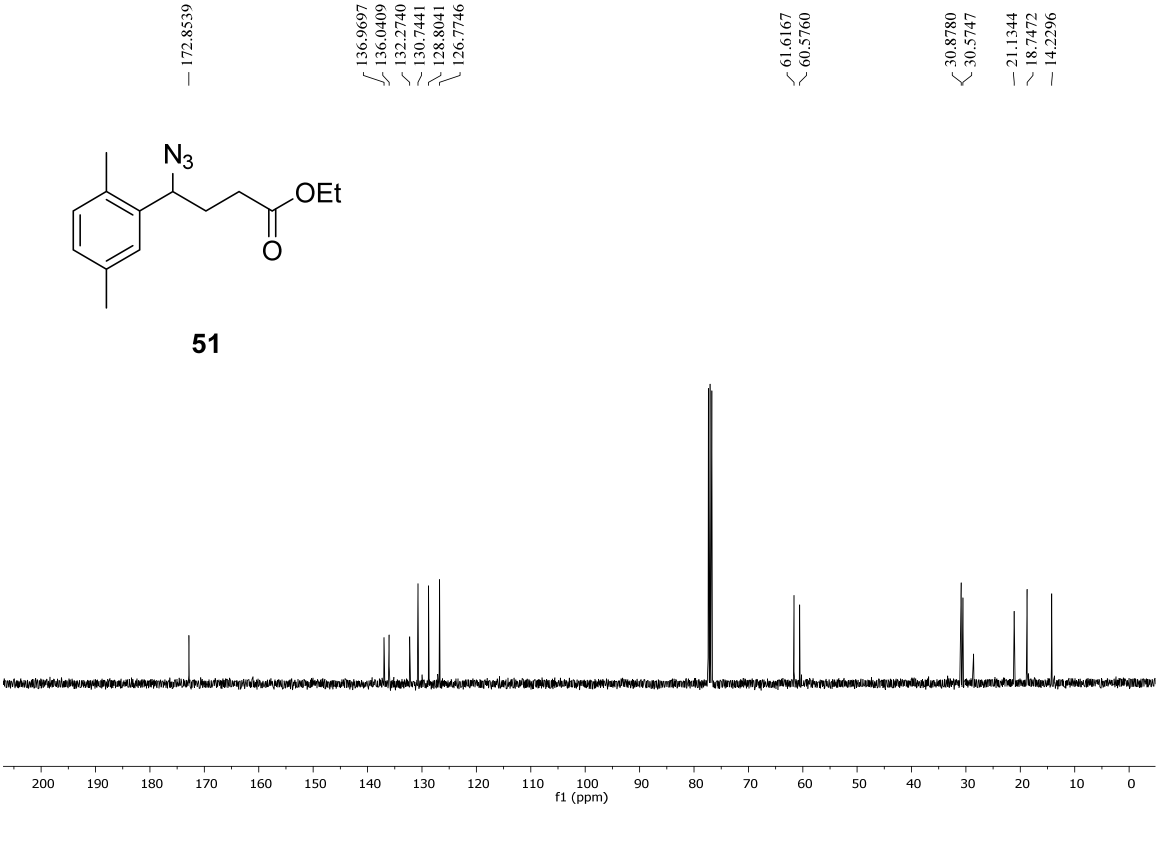

Supplementary Figure 127. ^{13}C NMR spectrum for compound **51**

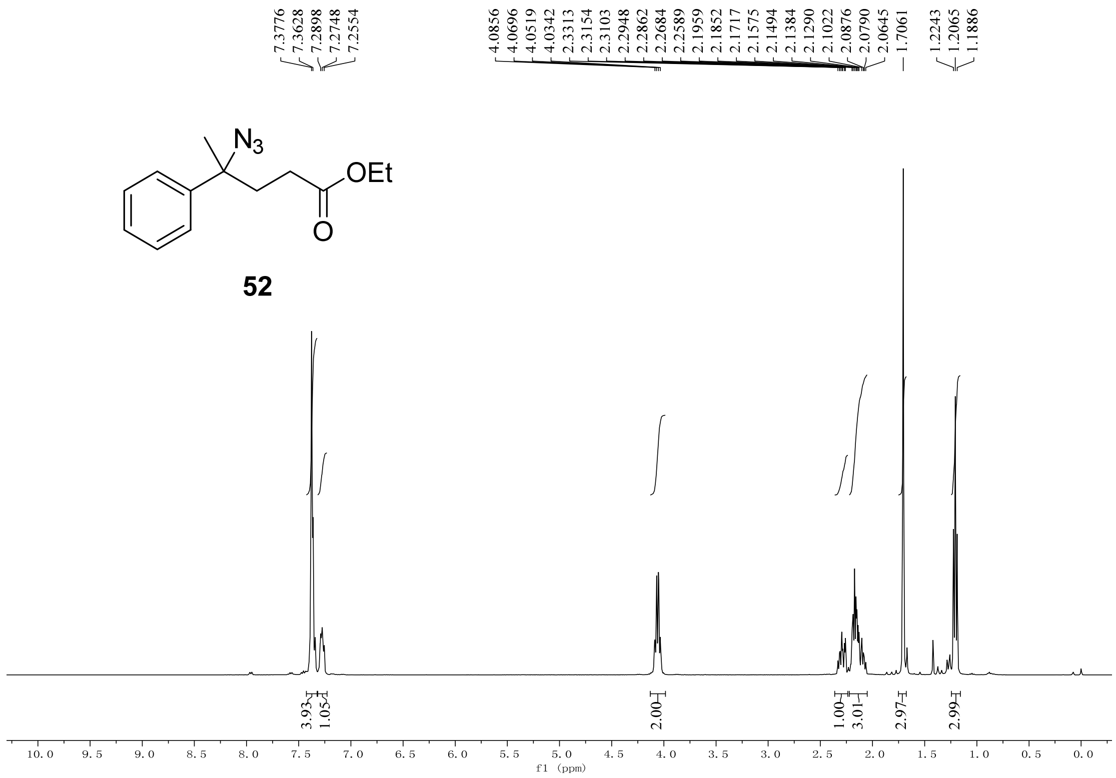

Supplementary Figure 128. ^{1}H NMR spectrum for compound **52**

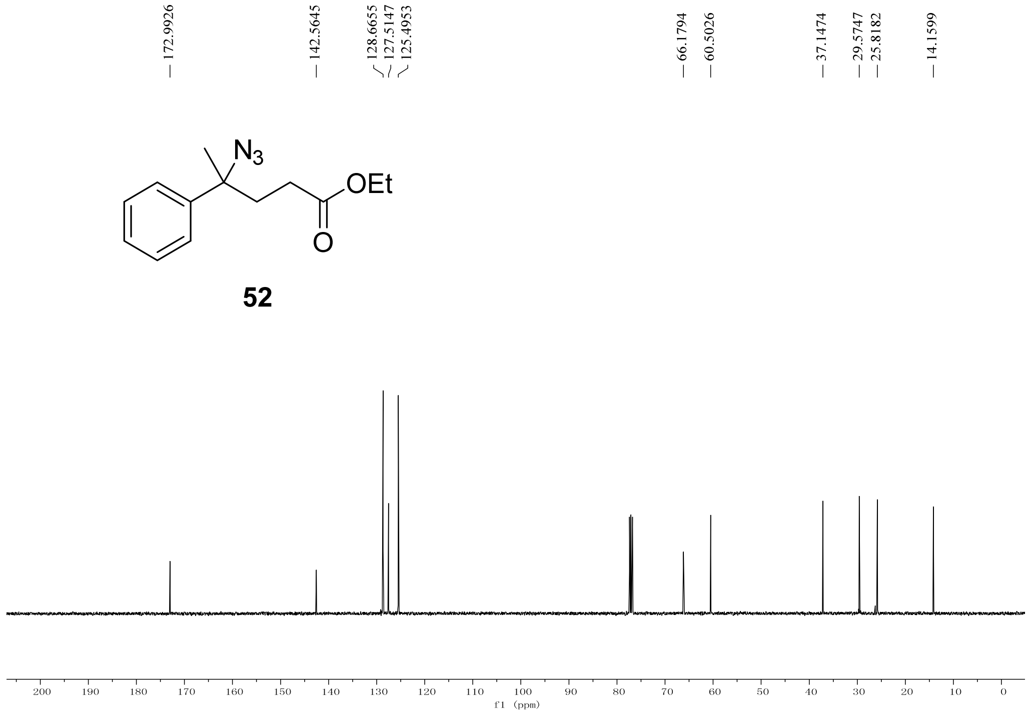

Supplementary Figure 129. ^{13}C NMR spectrum for compound **52**

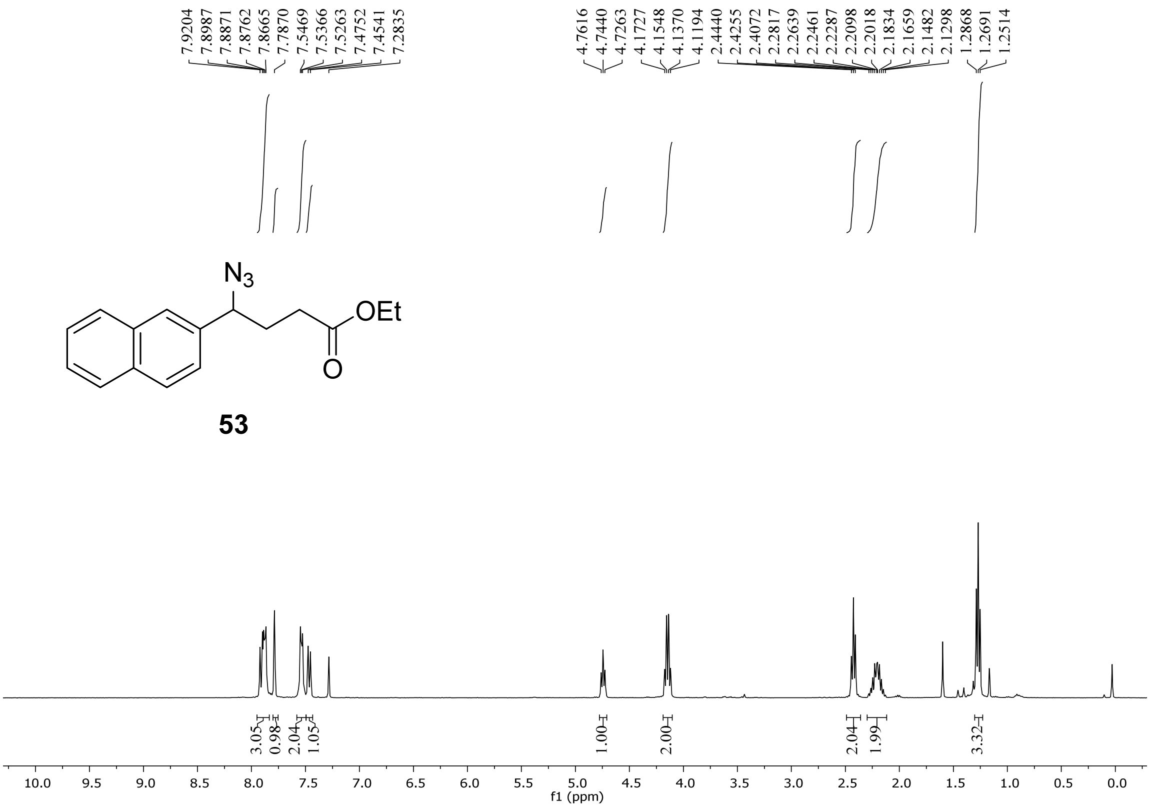

Supplementary Figure 130. ^{1}H NMR spectrum for compound **53**

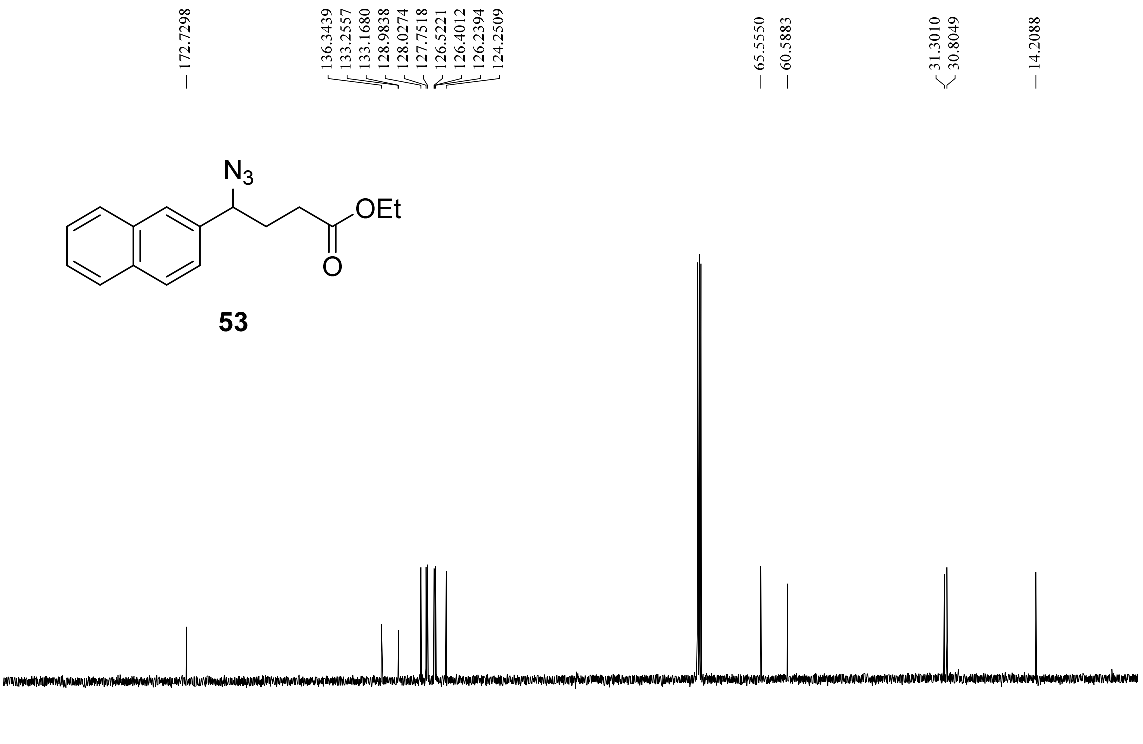

Supplementary Figure 131. ^{13}C NMR spectrum for compound **53**

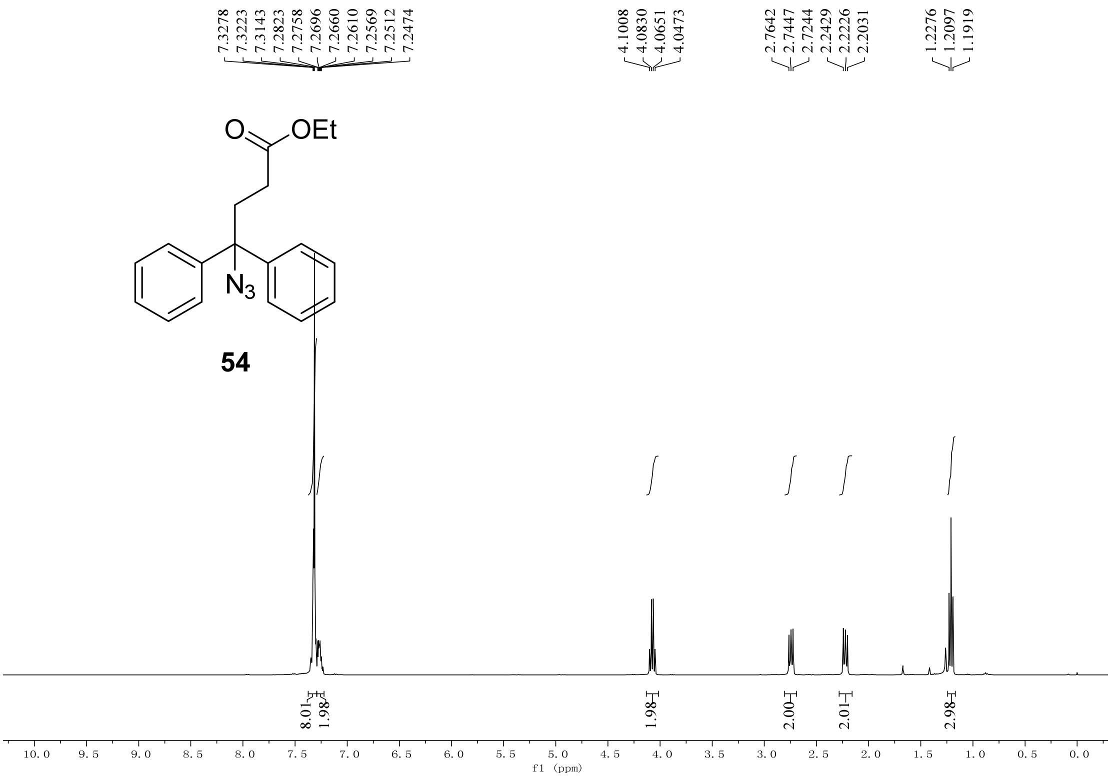

Supplementary Figure 132. ^{1}H NMR spectrum for compound **54**

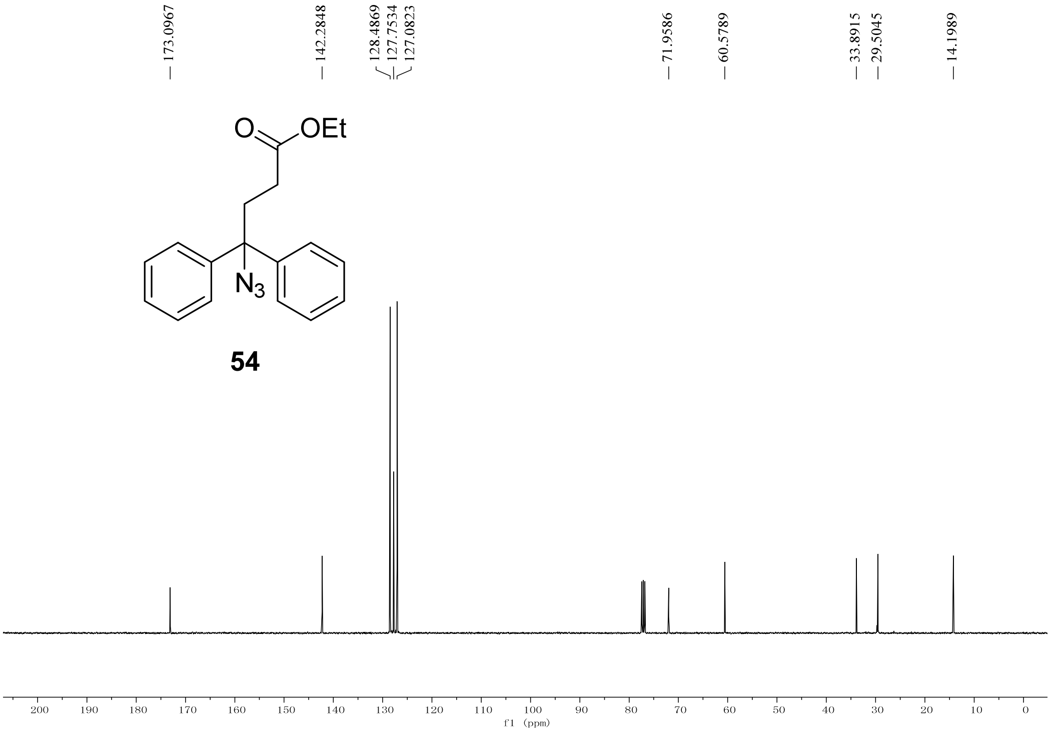

Supplementary Figure 133. ^{13}C NMR spectrum for compound **54**

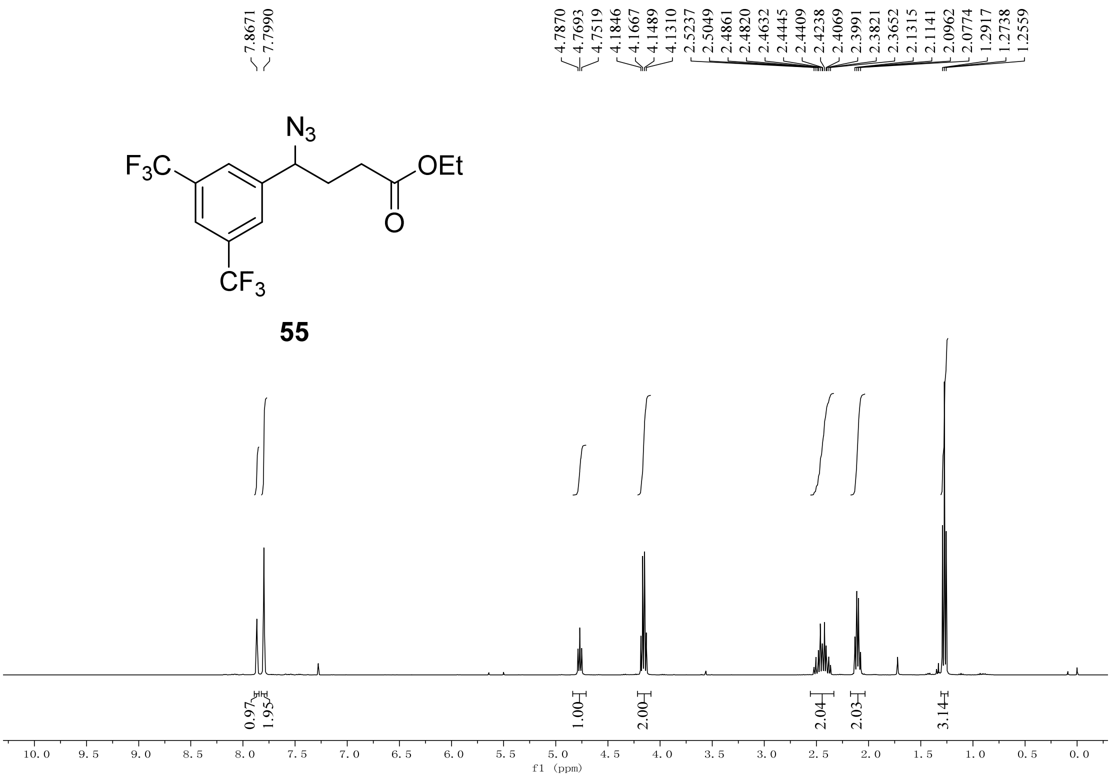

Supplementary Figure 134. ^{1}H NMR spectrum for compound **55**

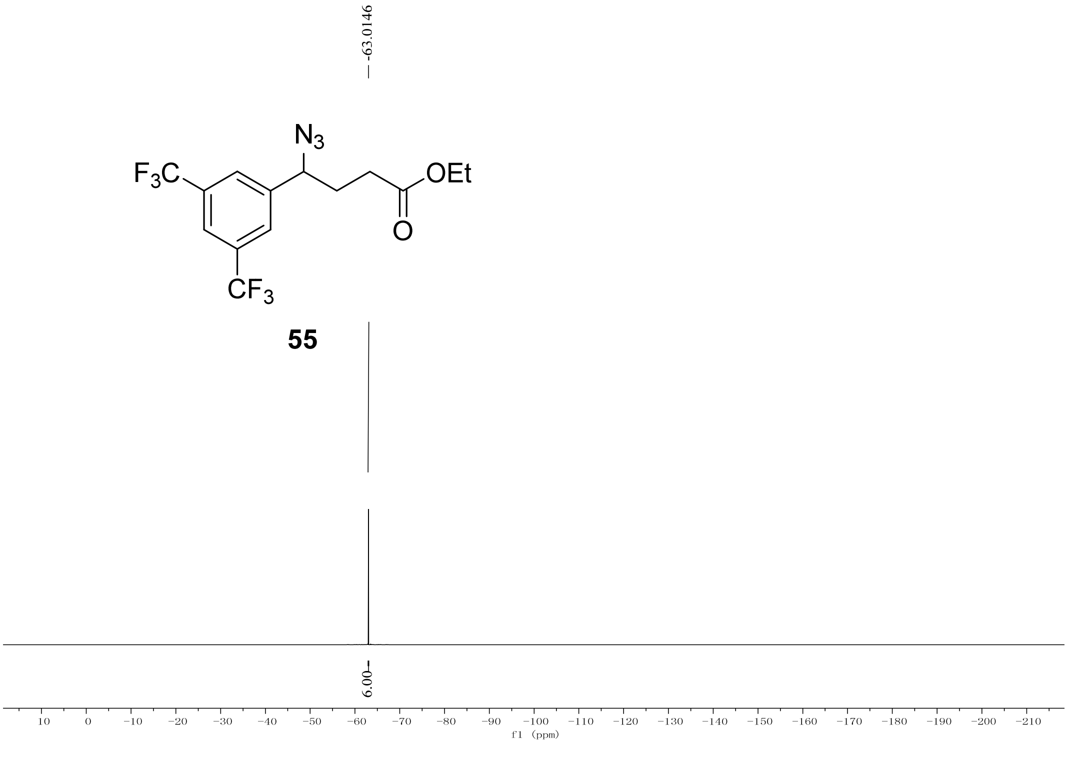

Supplementary Figure 135. ^{19}F NMR spectrum for compound **55**

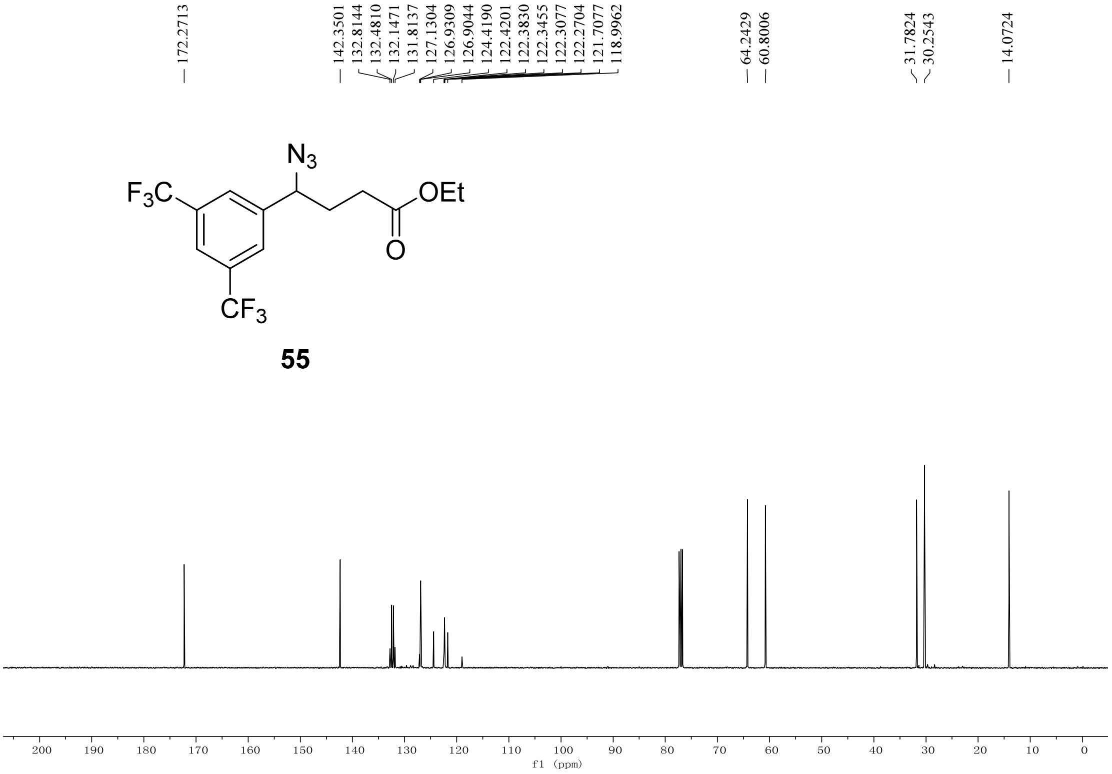

Supplementary Figure 136. ^{13}C NMR spectrum for compound **55**

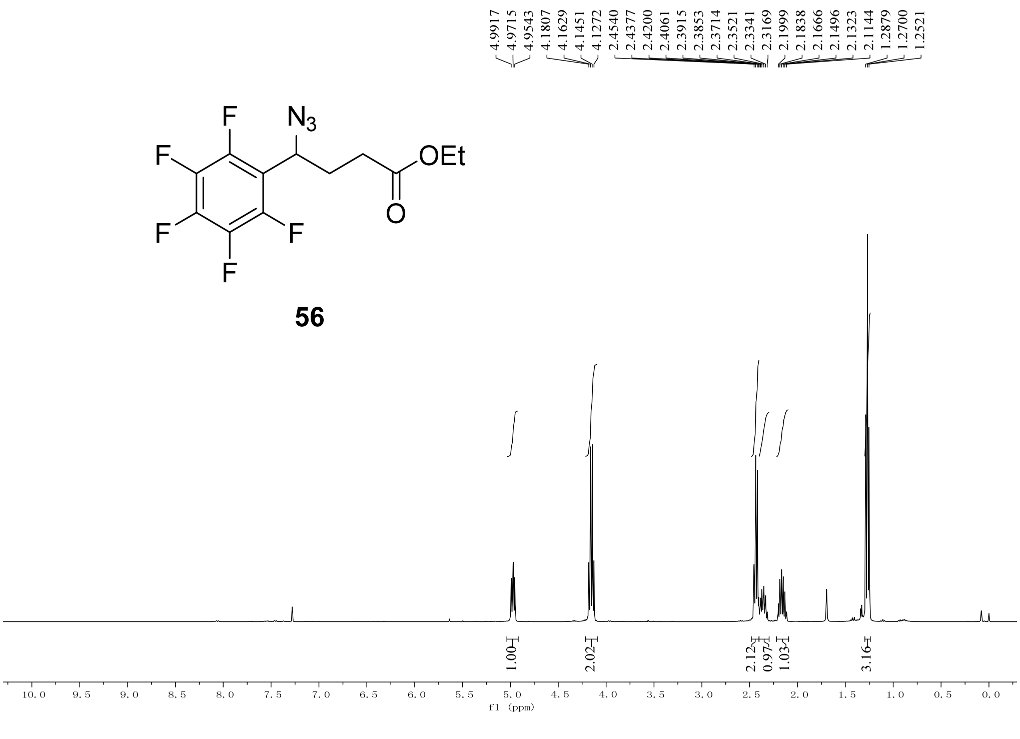

Supplementary Figure 137. ^{1}H NMR spectrum for compound **56**

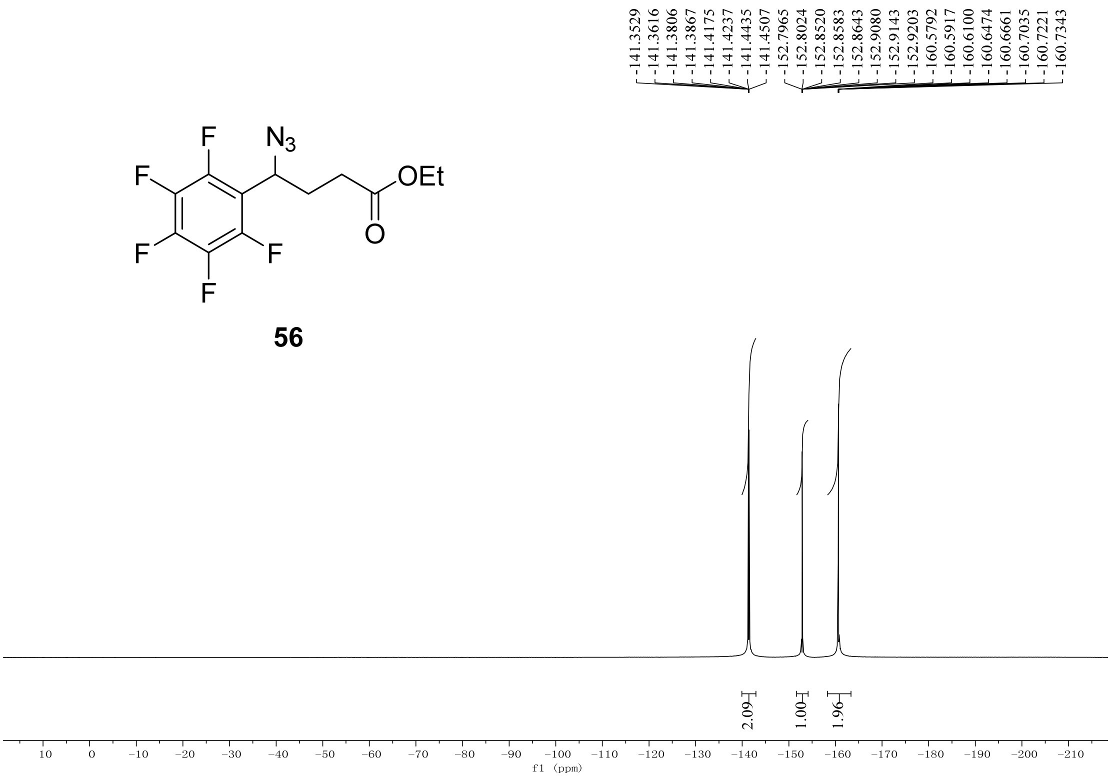

Supplementary Figure 138. ^{19}F NMR spectrum for compound **56**

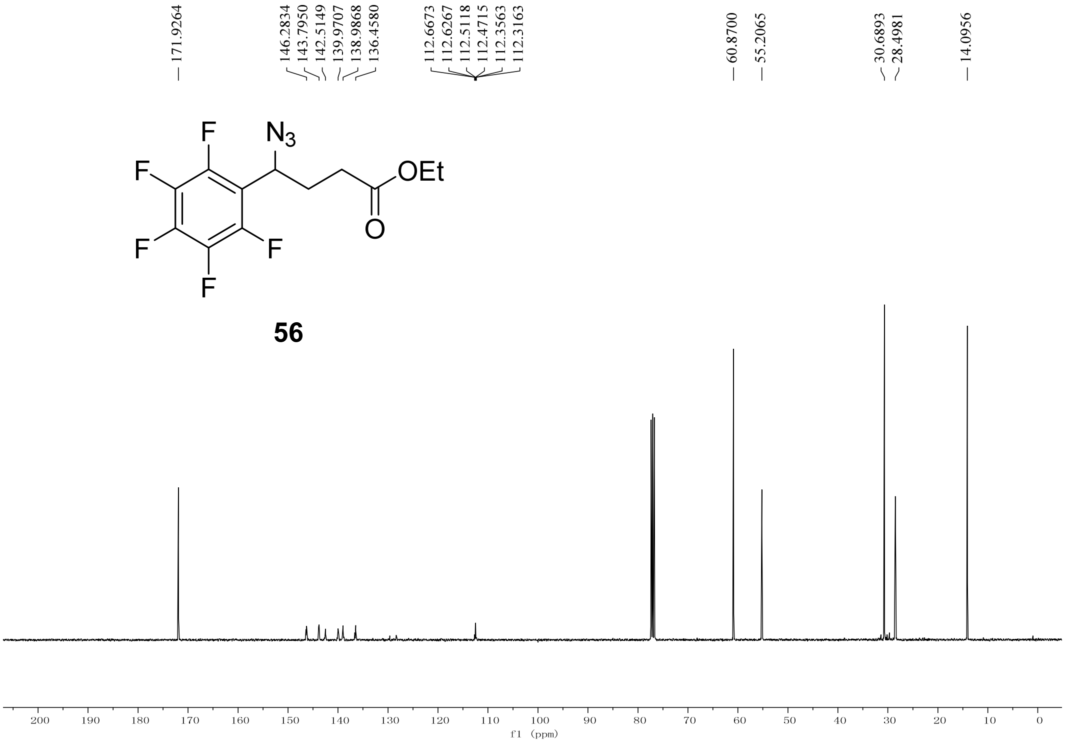

Supplementary Figure 139. ^{13}C NMR spectrum for compound **56**

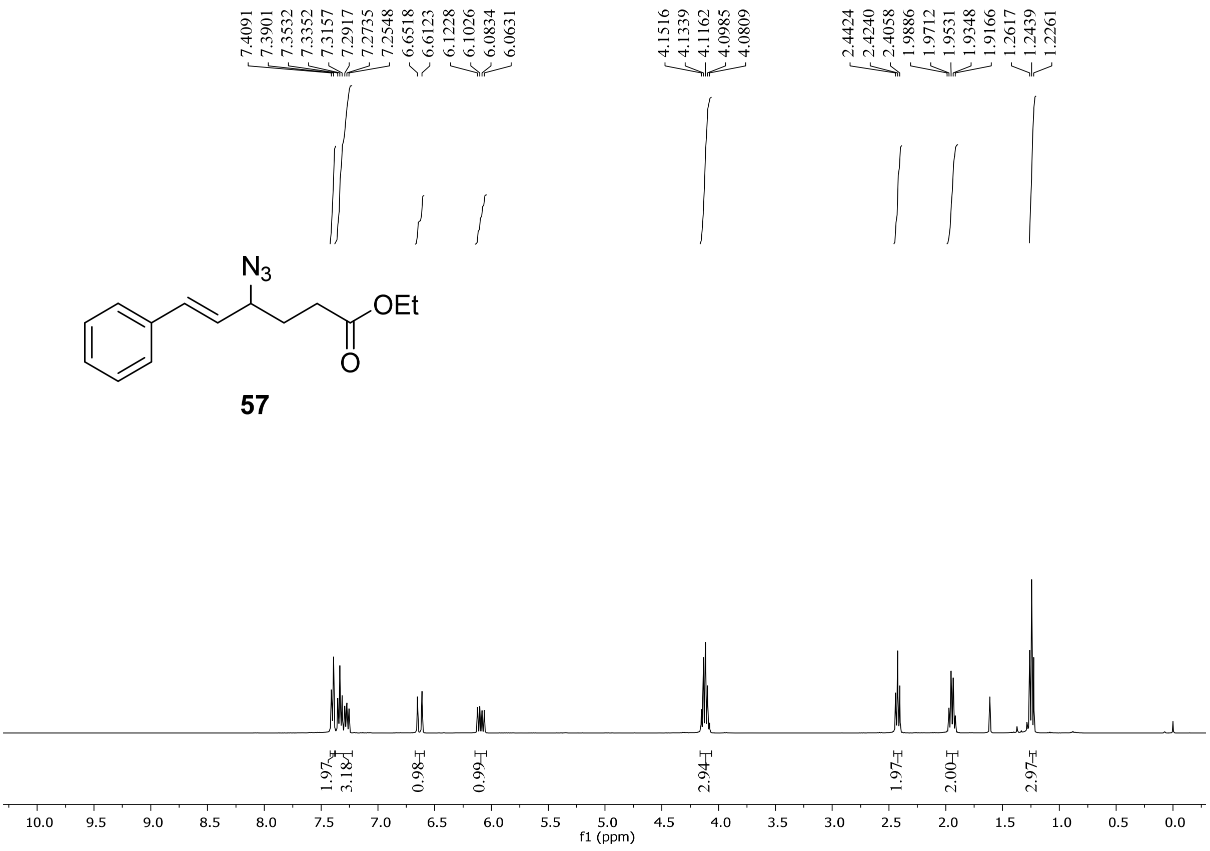

Supplementary Figure 140. ^{1}H NMR spectrum for compound **57**

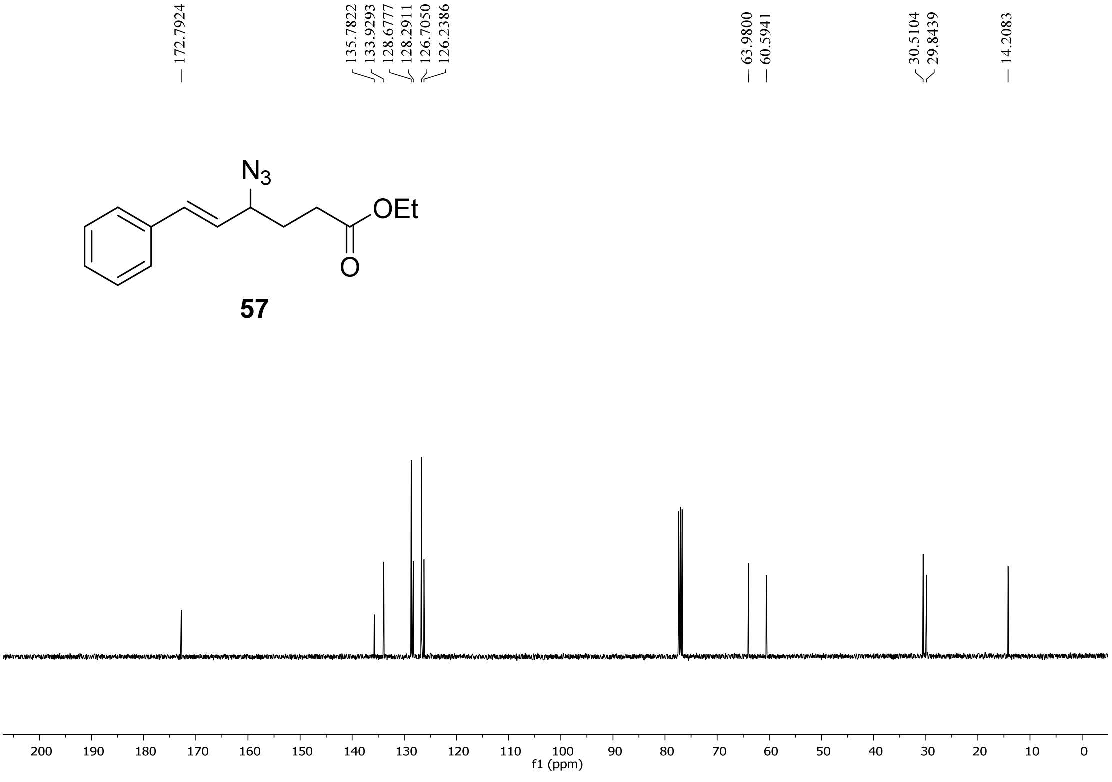

Supplementary Figure 141. ^{13}C NMR spectrum for compound **57**

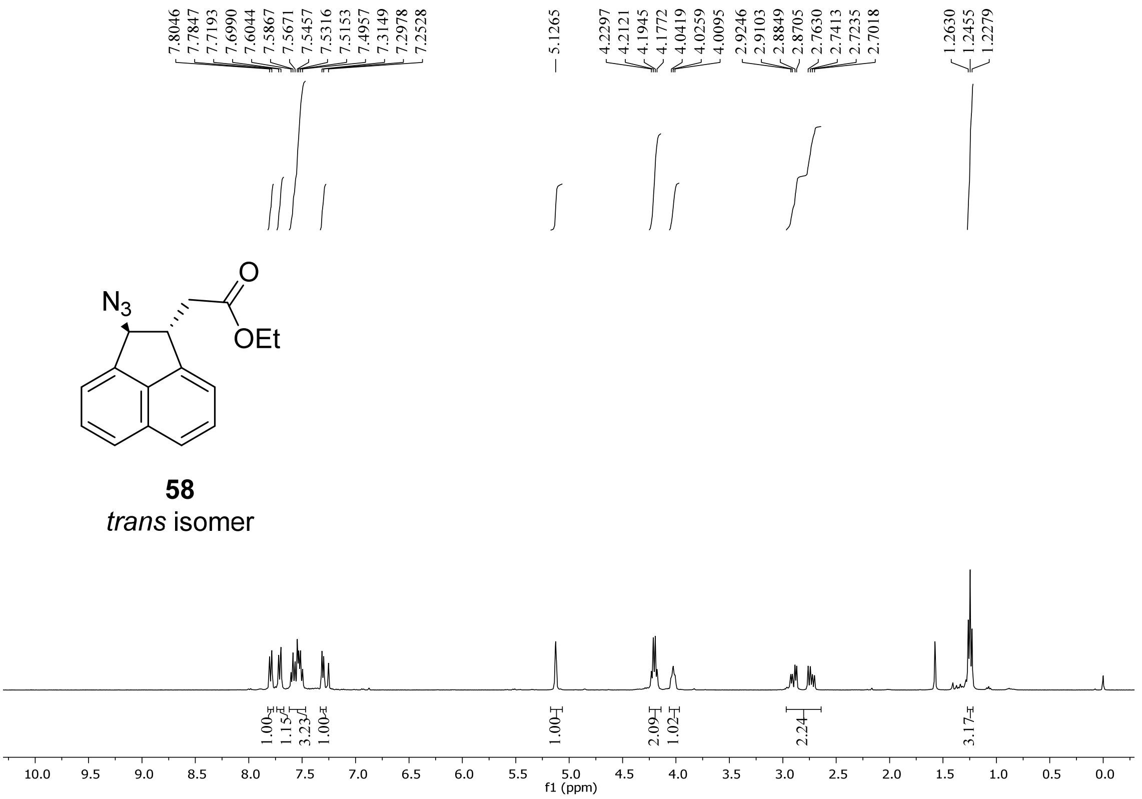

58
trans isomer

Supplementary Figure 142. ^{1}H NMR spectrum for compound *trans*-**58**

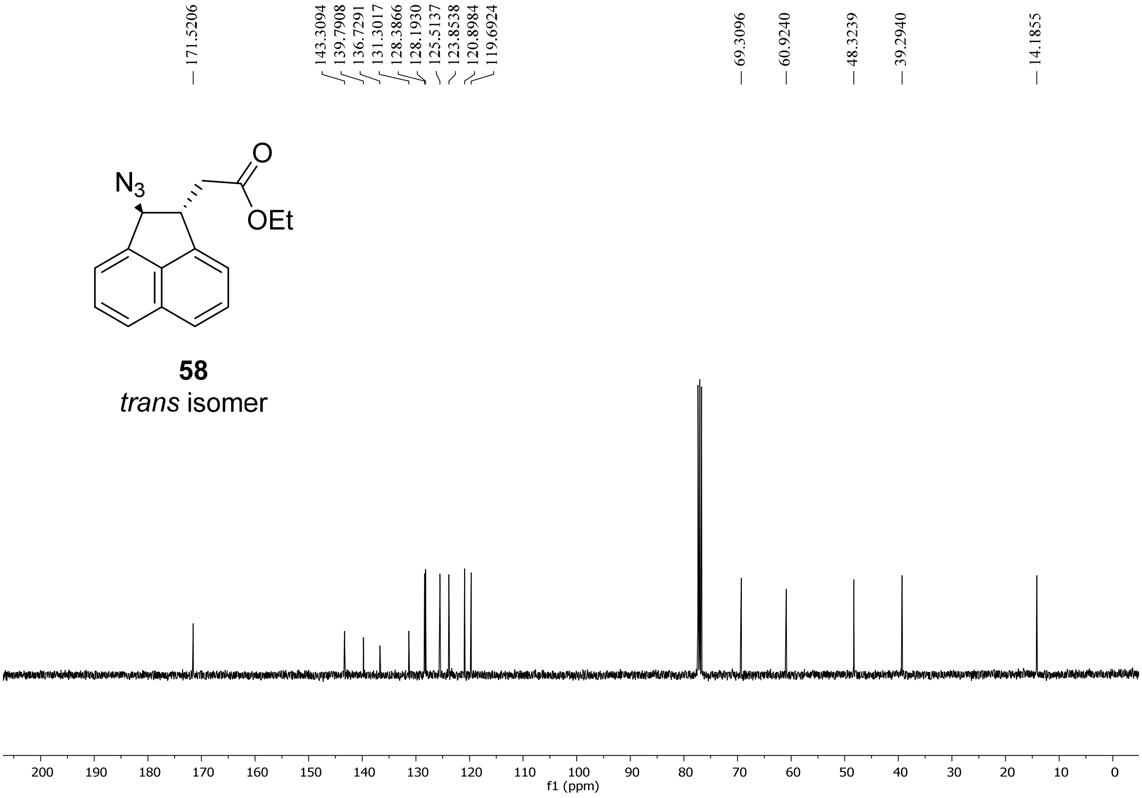

58
trans isomer

Supplementary Figure 143. ^{13}C NMR spectrum for compound *trans*-**58**

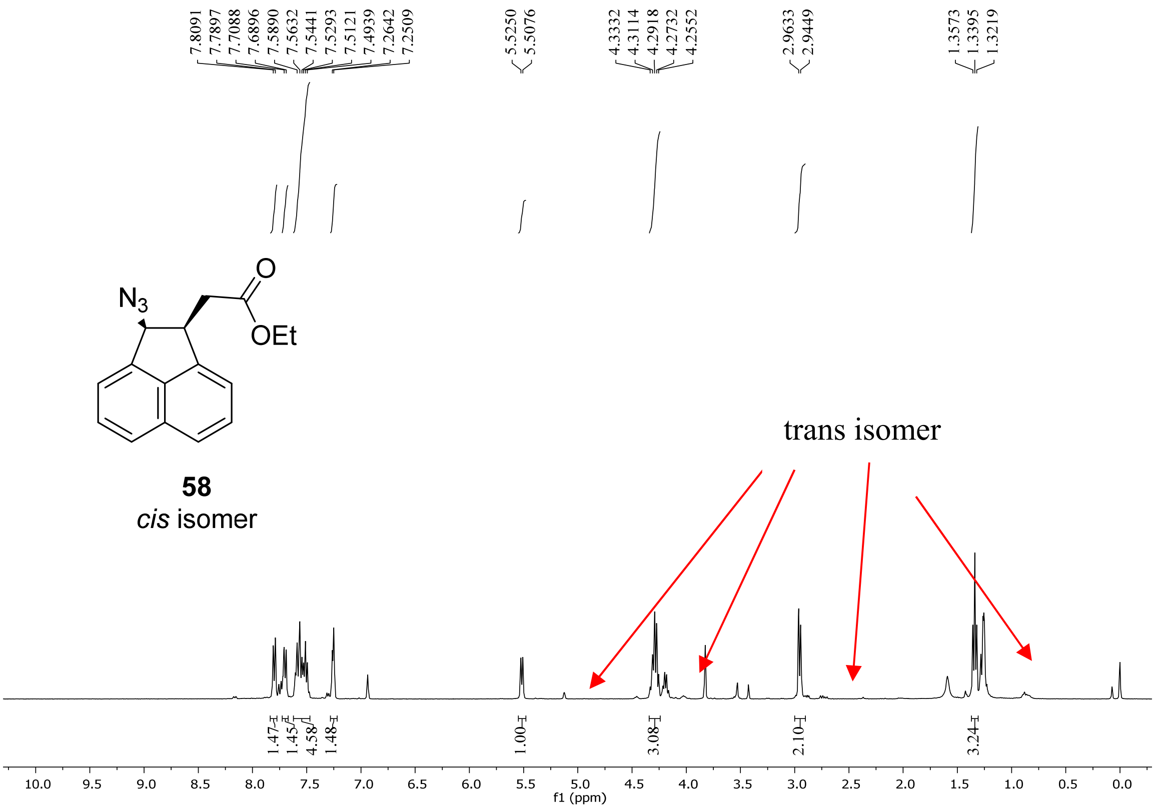

Supplementary Figure 144. ^{1}H NMR spectrum for compound *cis*-**58**

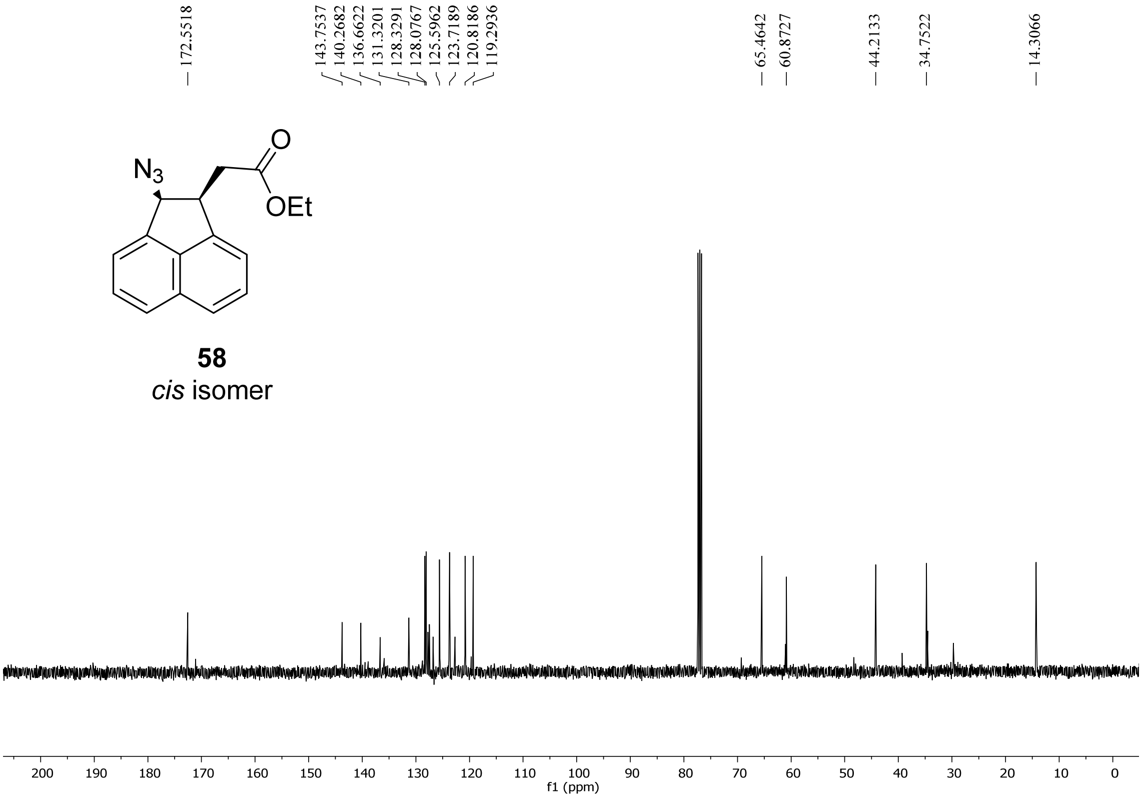

Supplementary Figure 145. ^{13}C NMR spectrum for compound *cis*-**58**

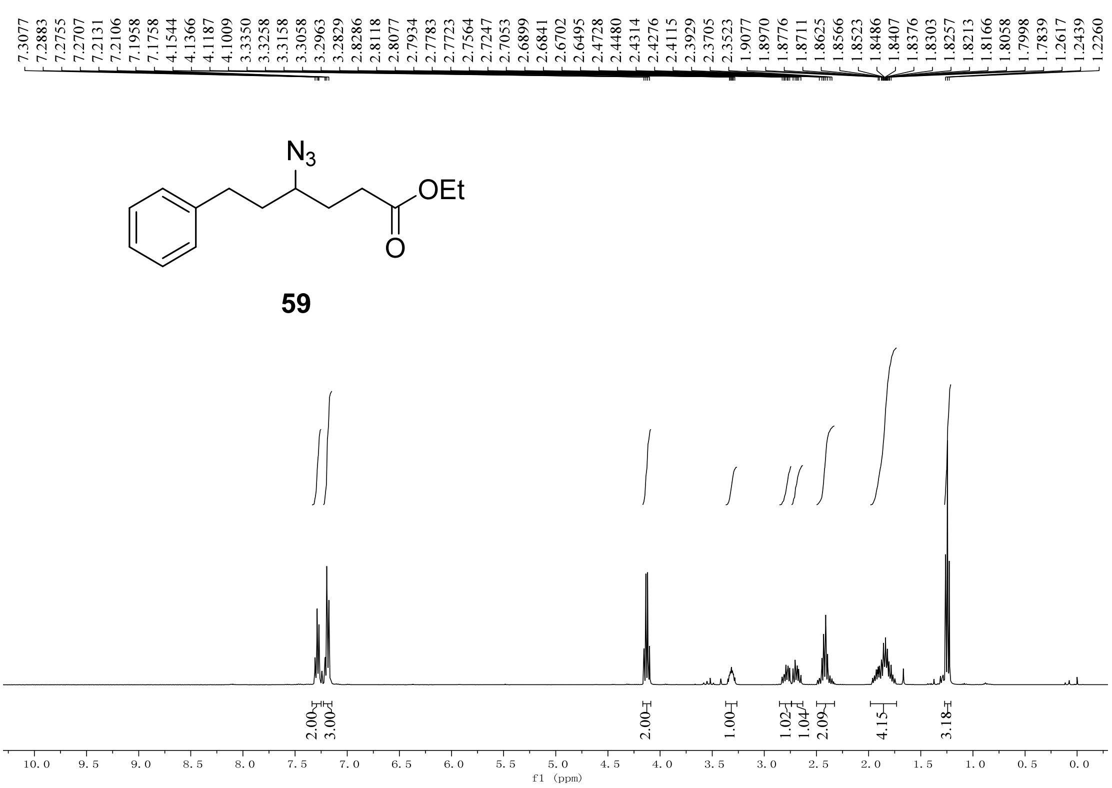

Supplementary Figure 146. ^{1}H NMR spectrum for compound **59**

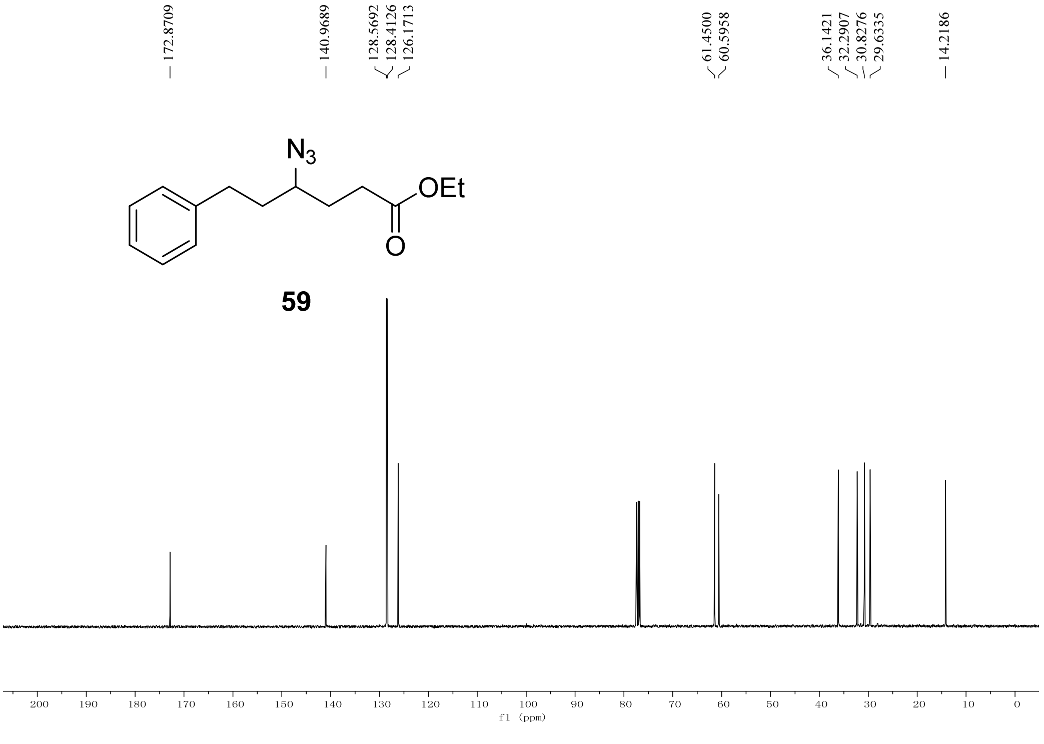

Supplementary Figure 147. ^{13}C NMR spectrum for compound **59**

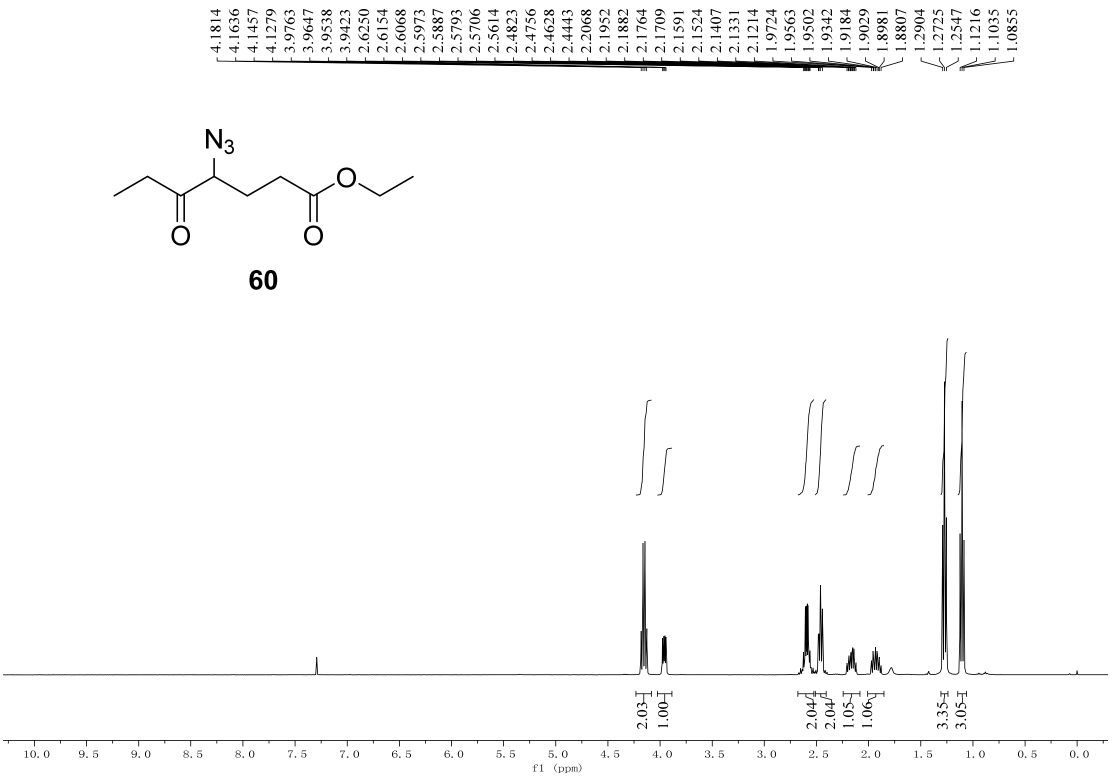

Supplementary Figure 148. ^{1}H NMR spectrum for compound **60**

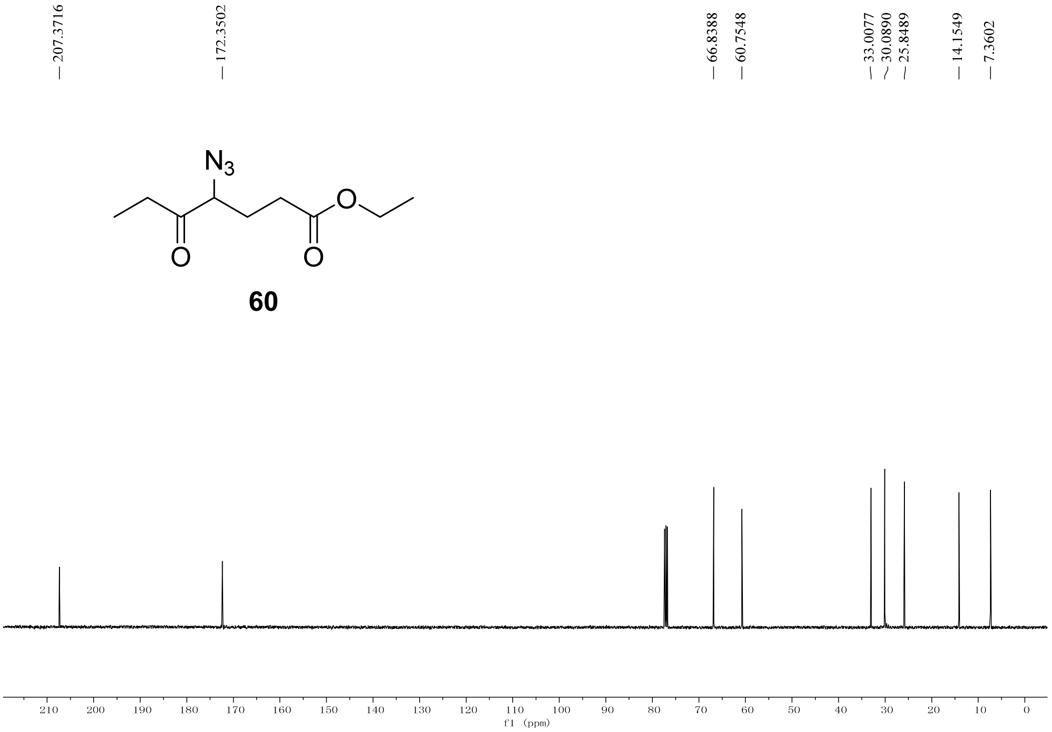

Supplementary Figure 149. ^{13}C NMR spectrum for compound **60**

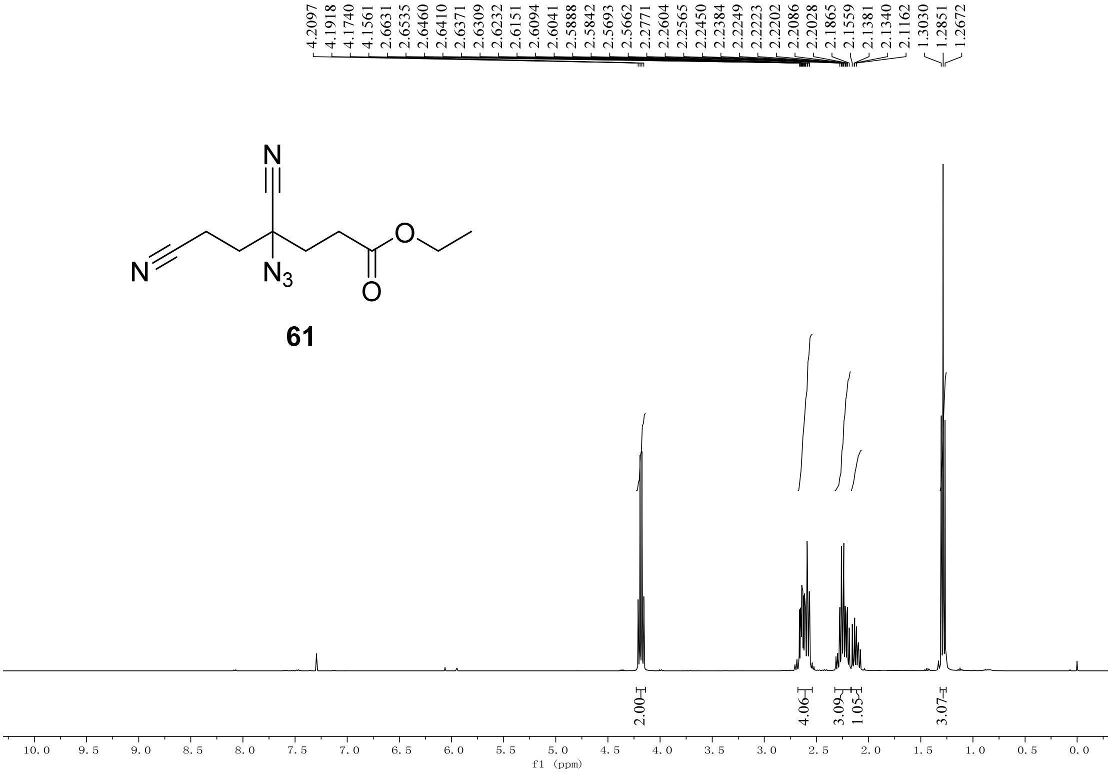

Supplementary Figure 150. ^{1}H NMR spectrum for compound **61**

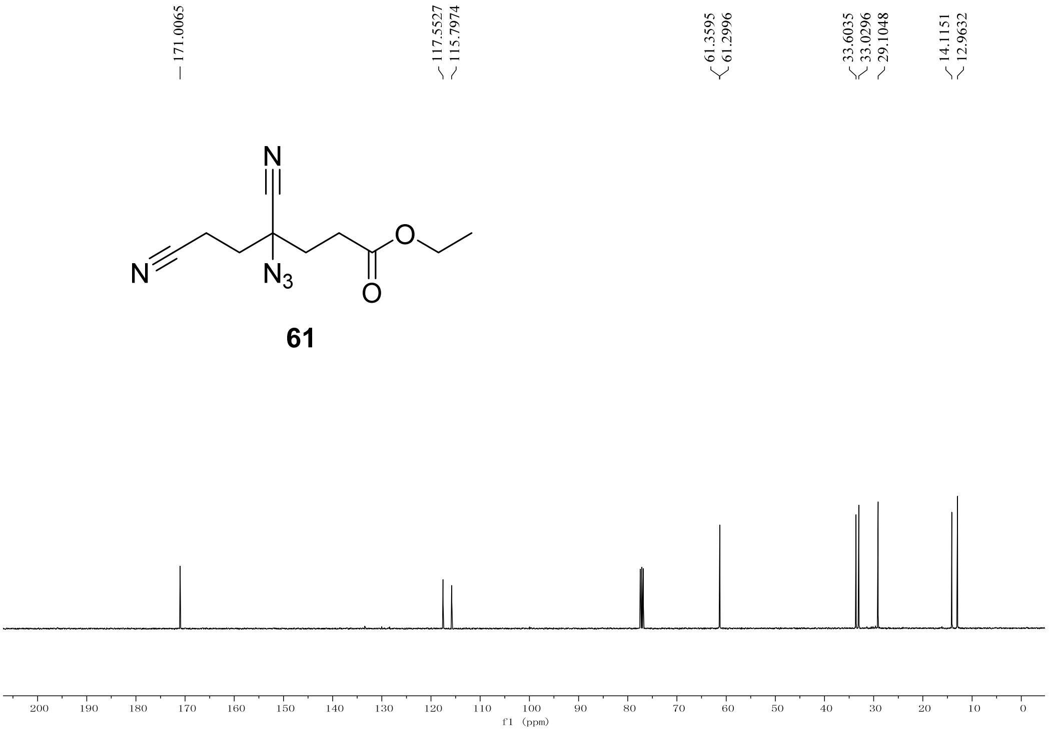

Supplementary Figure 151. ^{13}C NMR spectrum for compound **61**

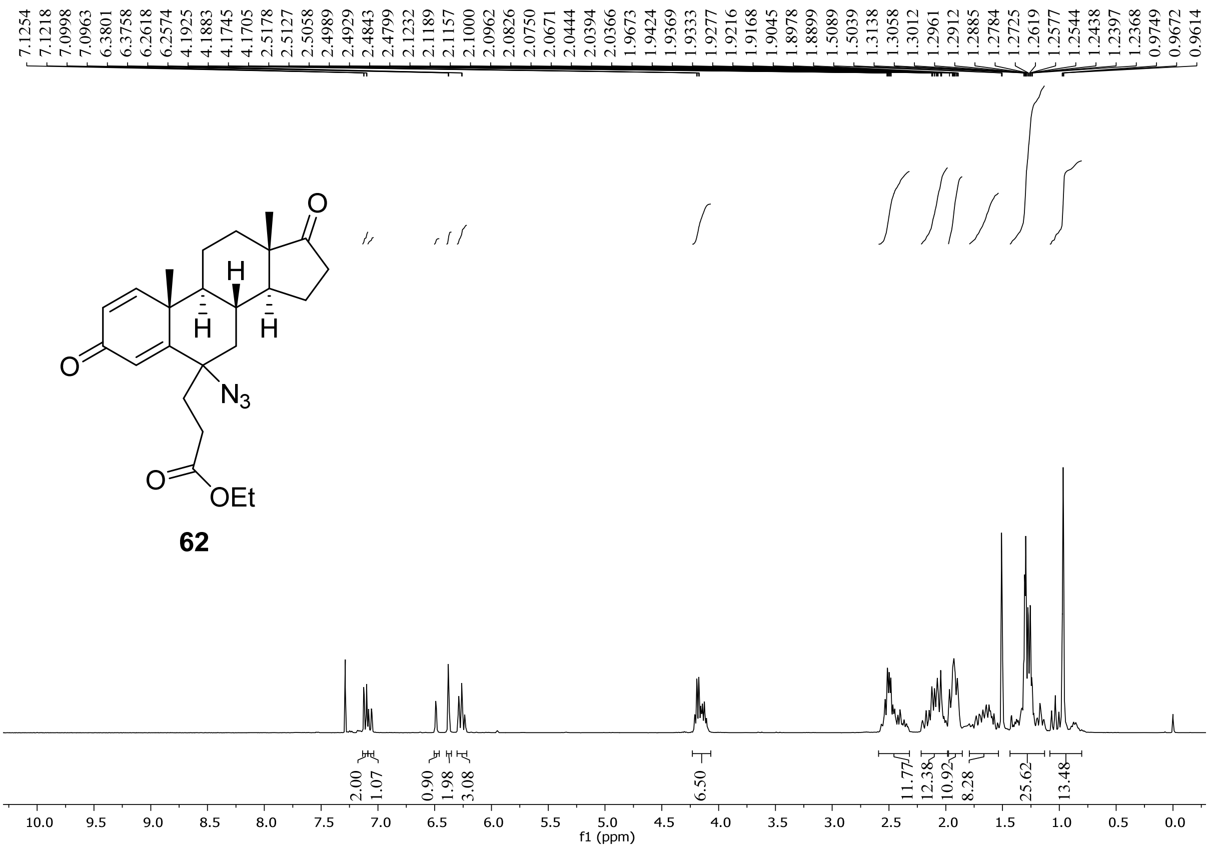

Supplementary Figure 152. ^{1}H NMR spectrum for compound **62**

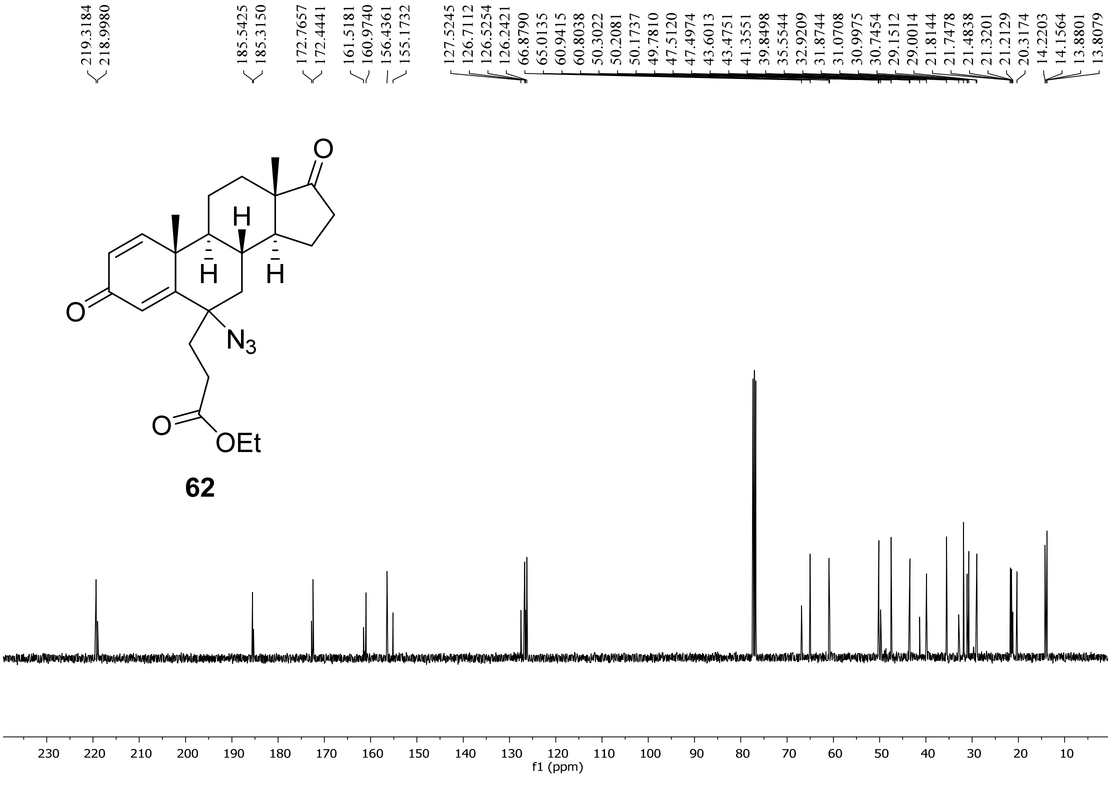

Supplementary Figure 153. ^{13}C NMR spectrum for compound **62**

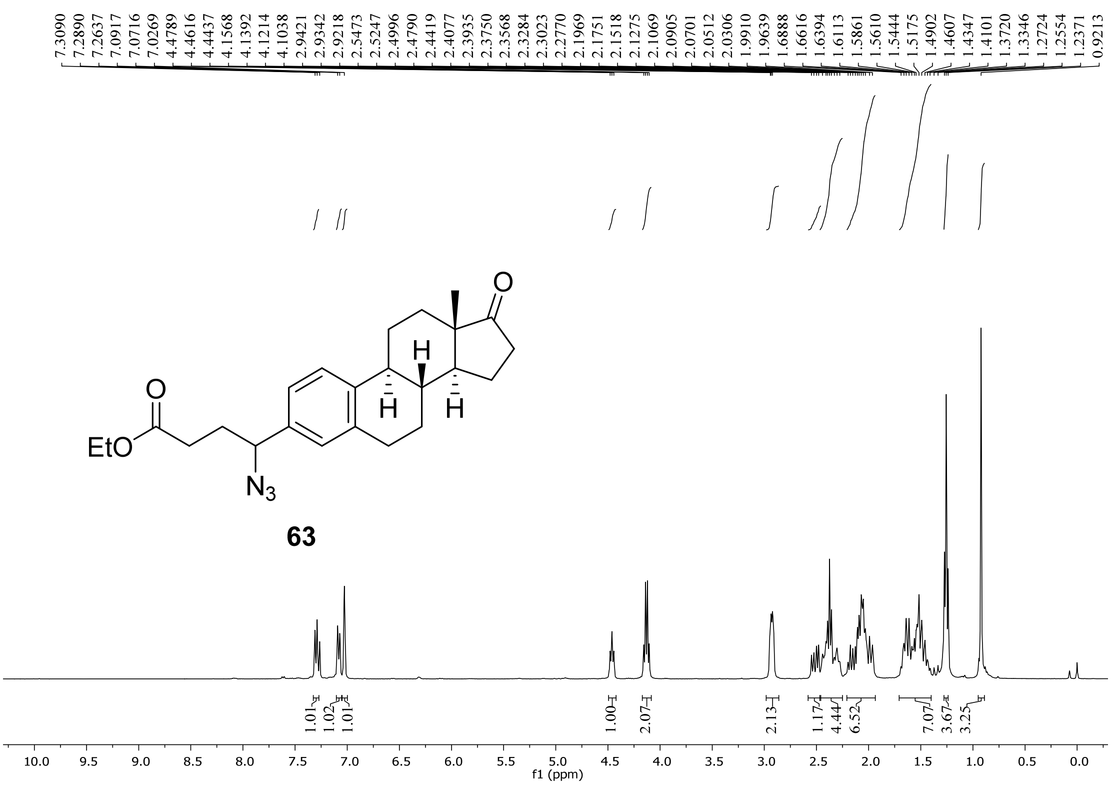

Supplementary Figure 154. ^{1}H NMR spectrum for compound **63**

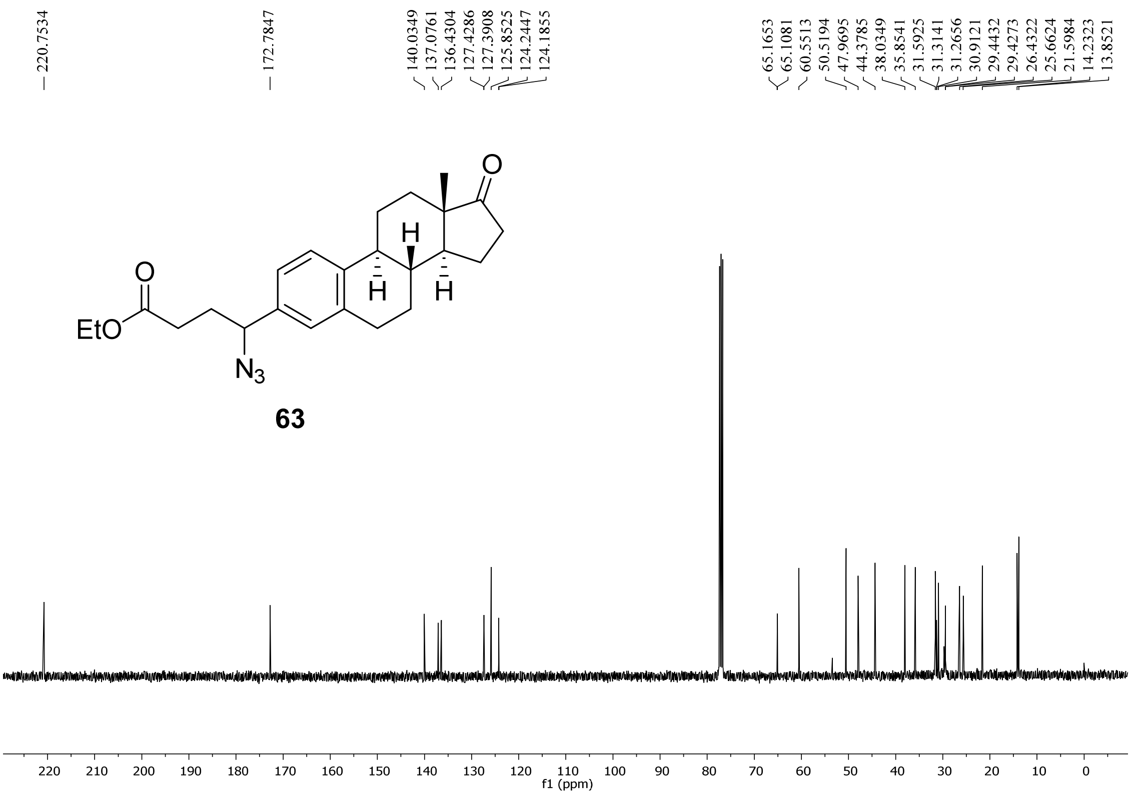

Supplementary Figure 155. ^{13}C NMR spectrum for compound **63**

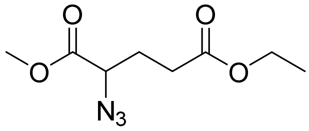

64

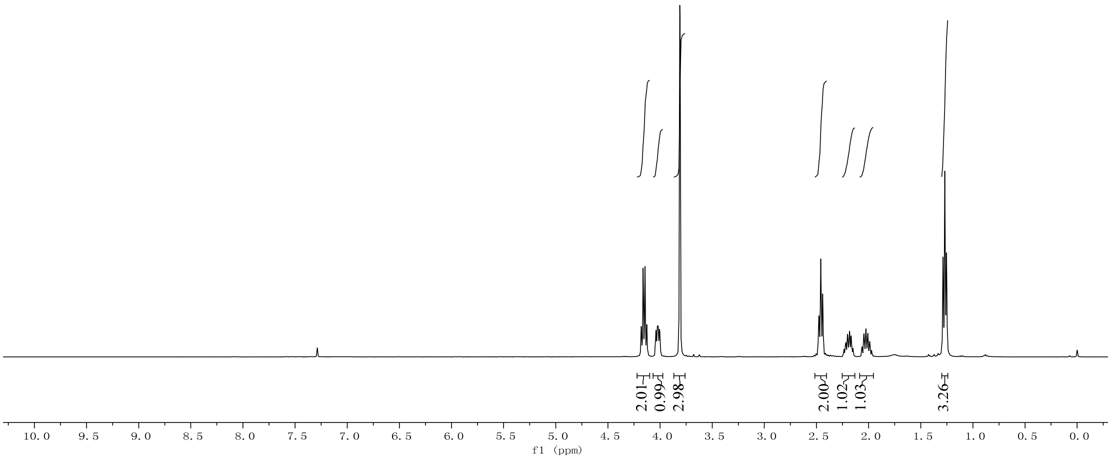

Supplementary Figure 156. ^{1}H NMR spectrum for compound **64**

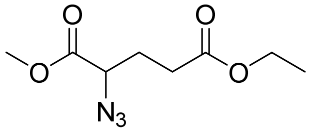

64

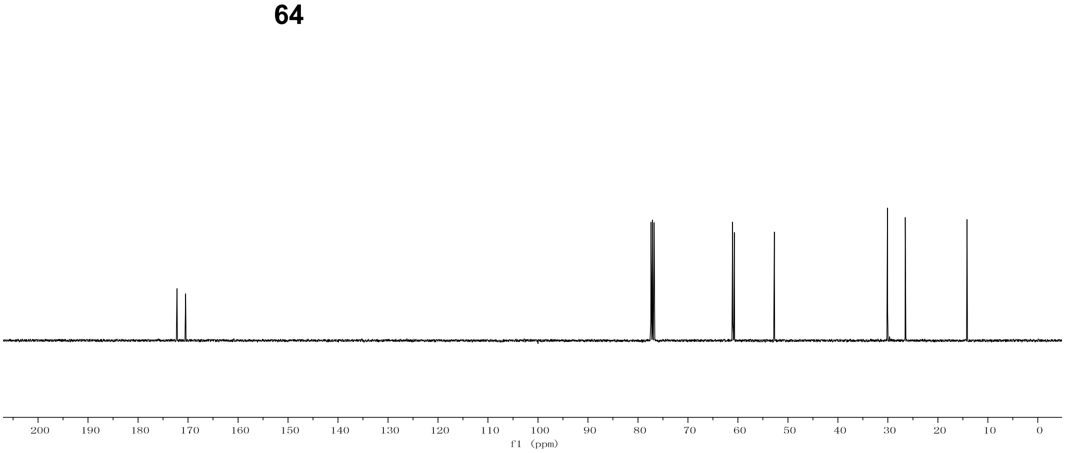

Supplementary Figure 157. ^{13}C NMR spectrum for compound **64**

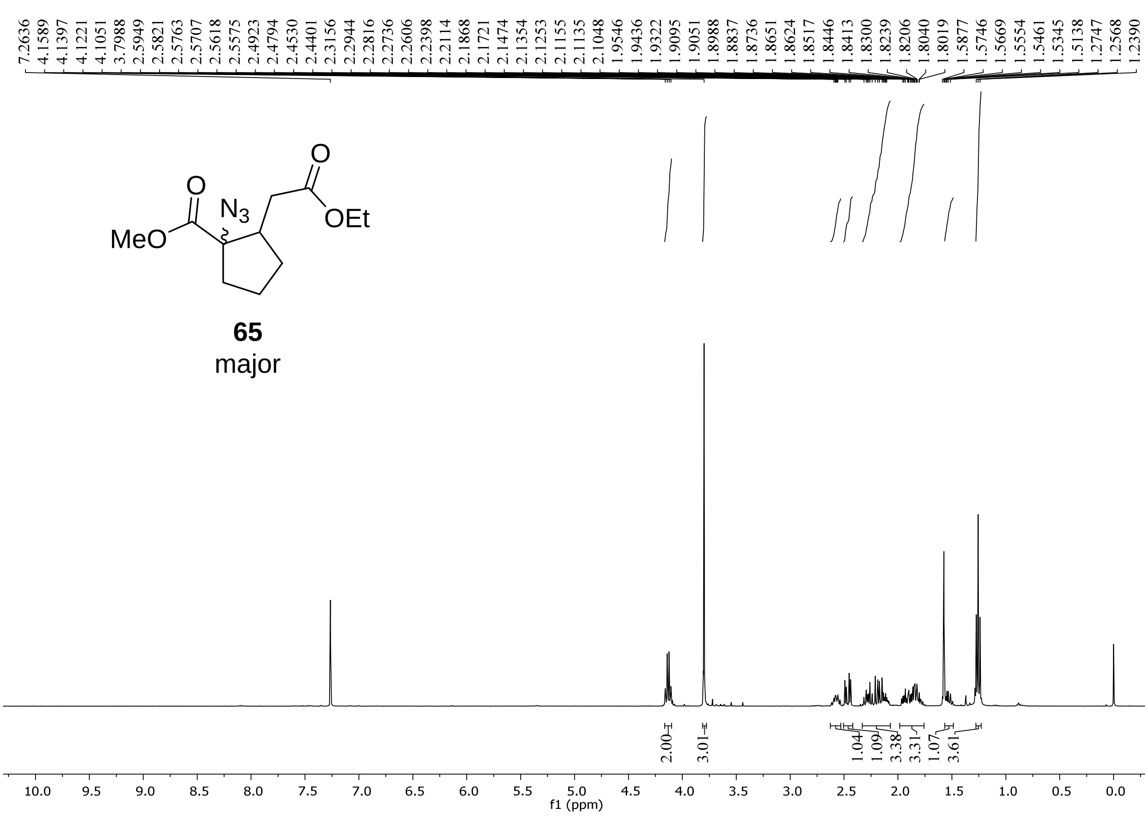

Supplementary Figure 158. ^{1}H NMR spectrum for compound *major*-**65**

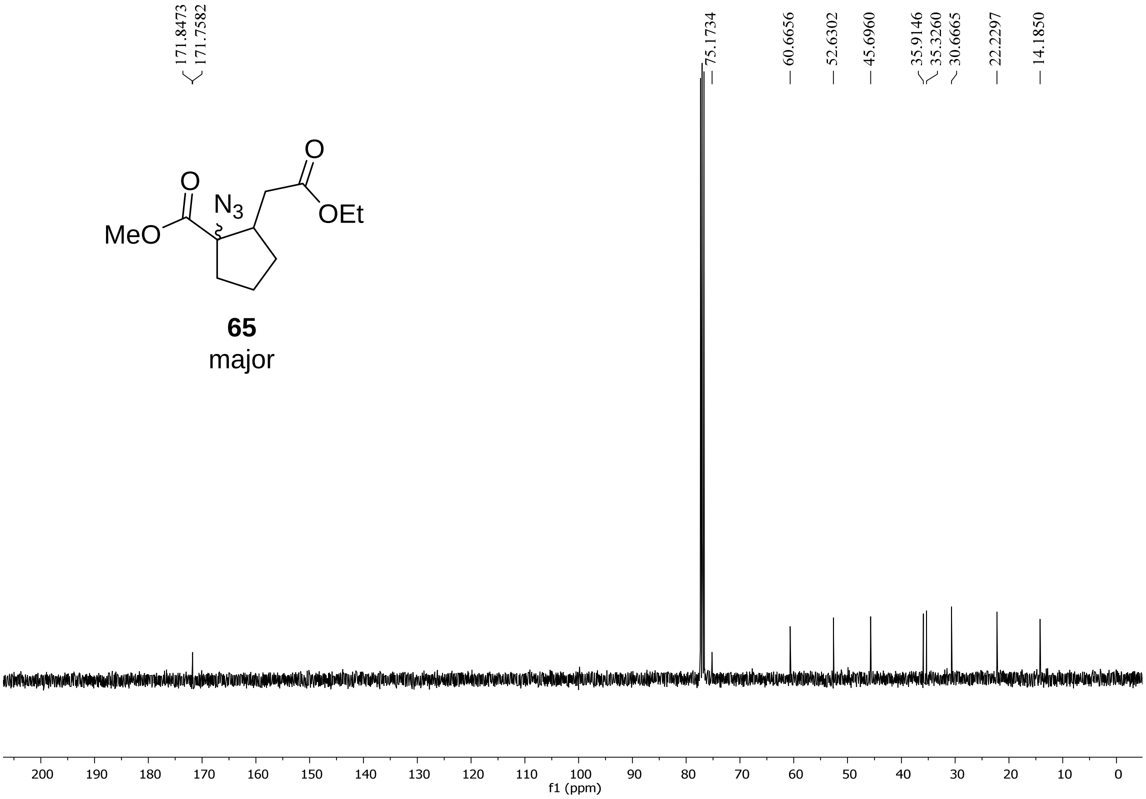

Supplementary Figure 159. ^{13}C NMR spectrum for compound *major*-**65**

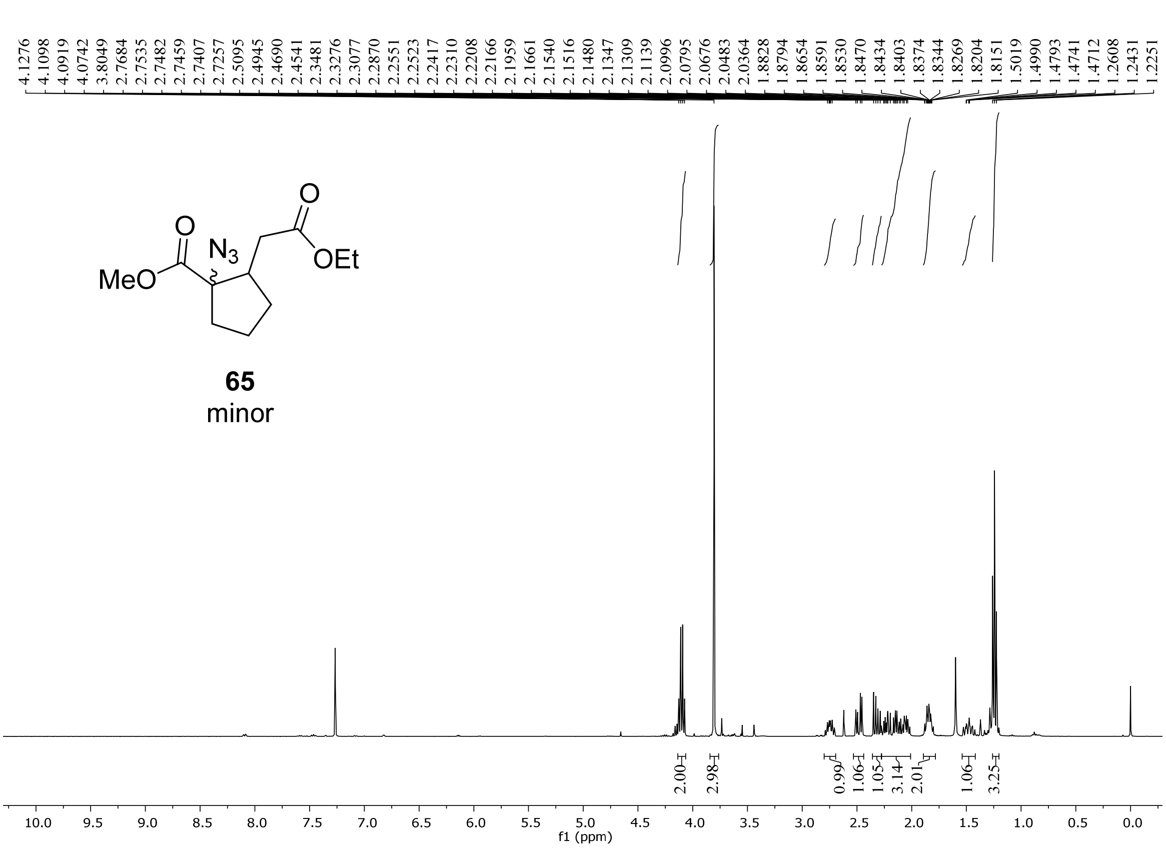

Supplementary Figure 160. ^{1}H NMR spectrum for compound *minor*-**65**

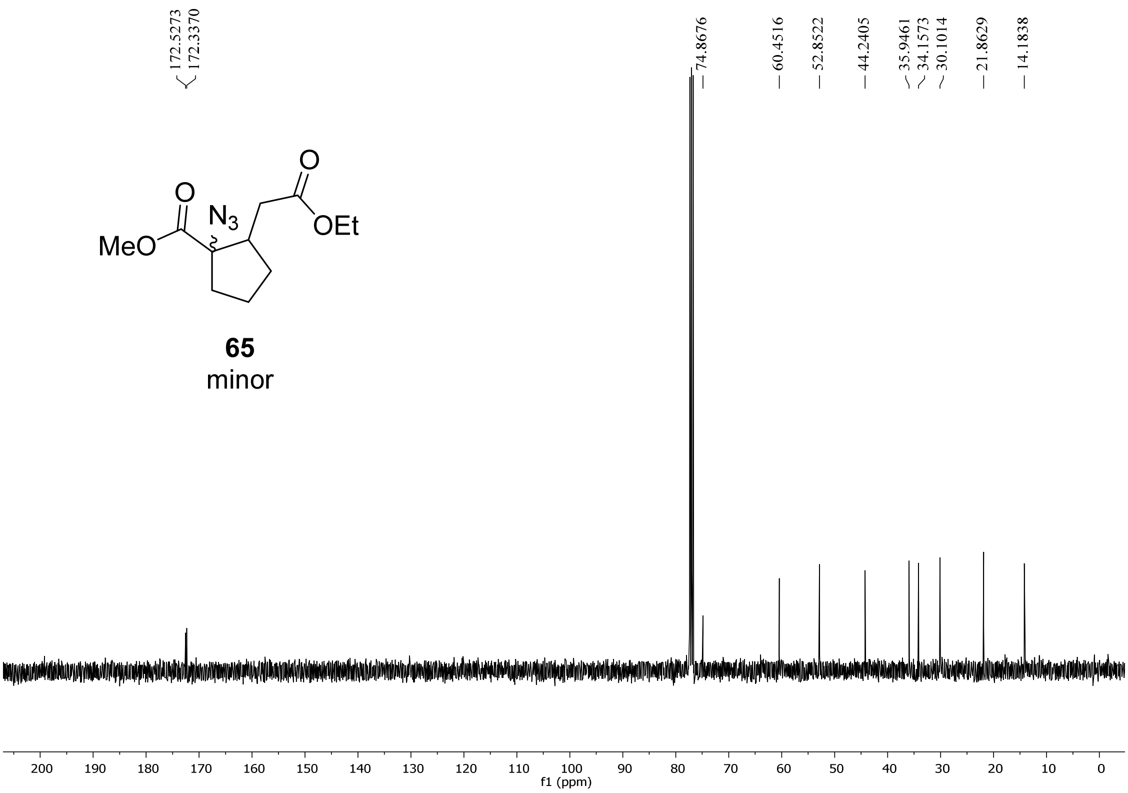

Supplementary Figure 161. ^{13}C NMR spectrum for compound *minor*-**65**

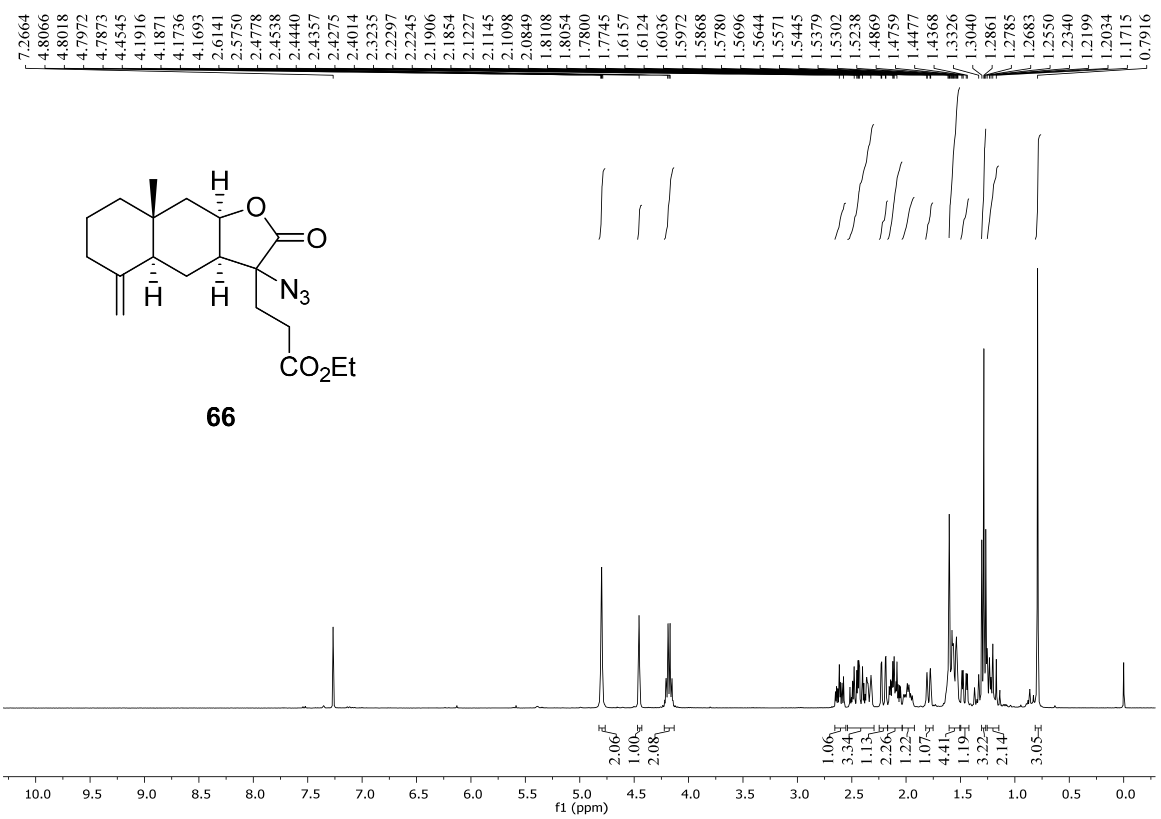

Supplementary Figure 162. ^{1}H NMR spectrum for compound **66**

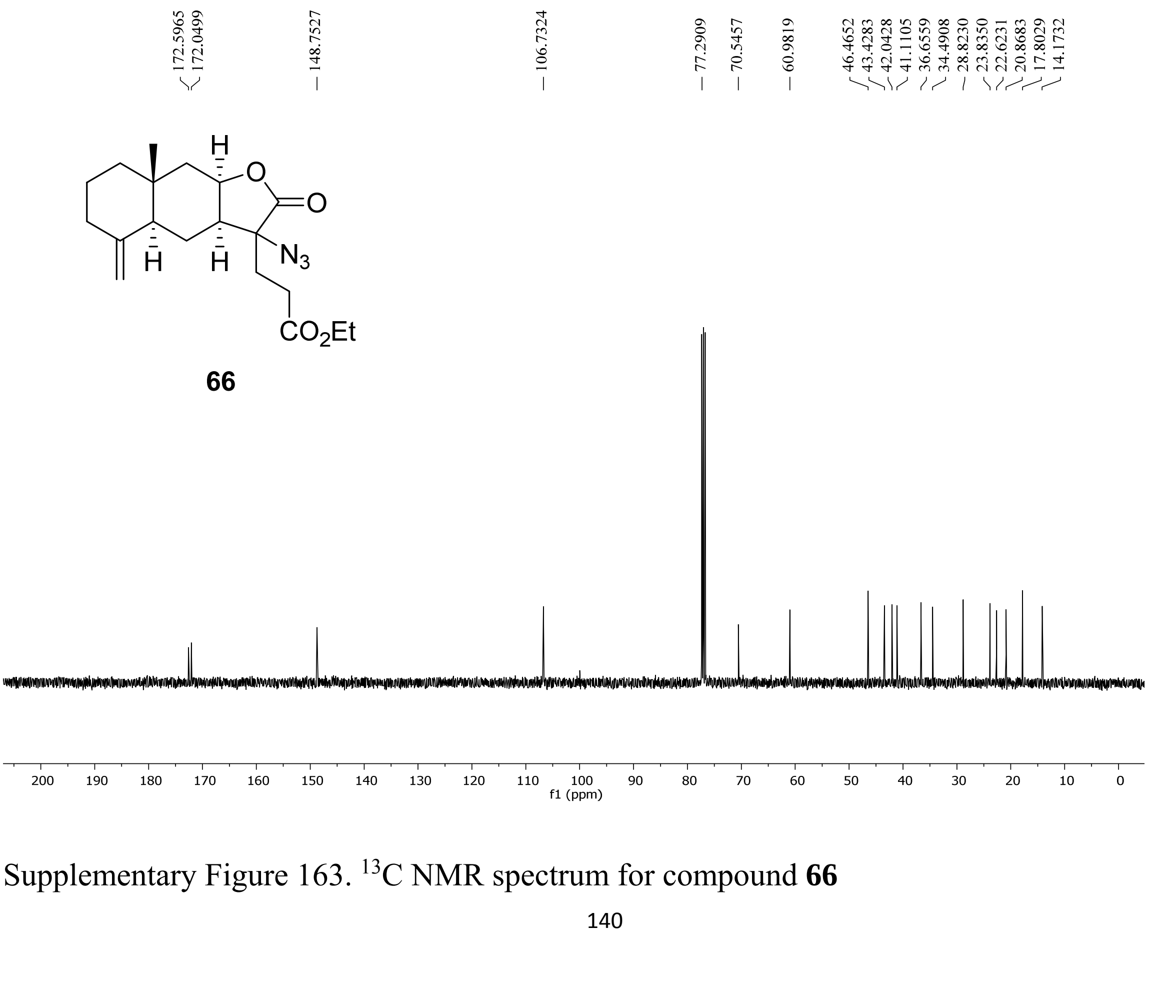

Supplementary Figure 163. ^{13}C NMR spectrum for compound **66**

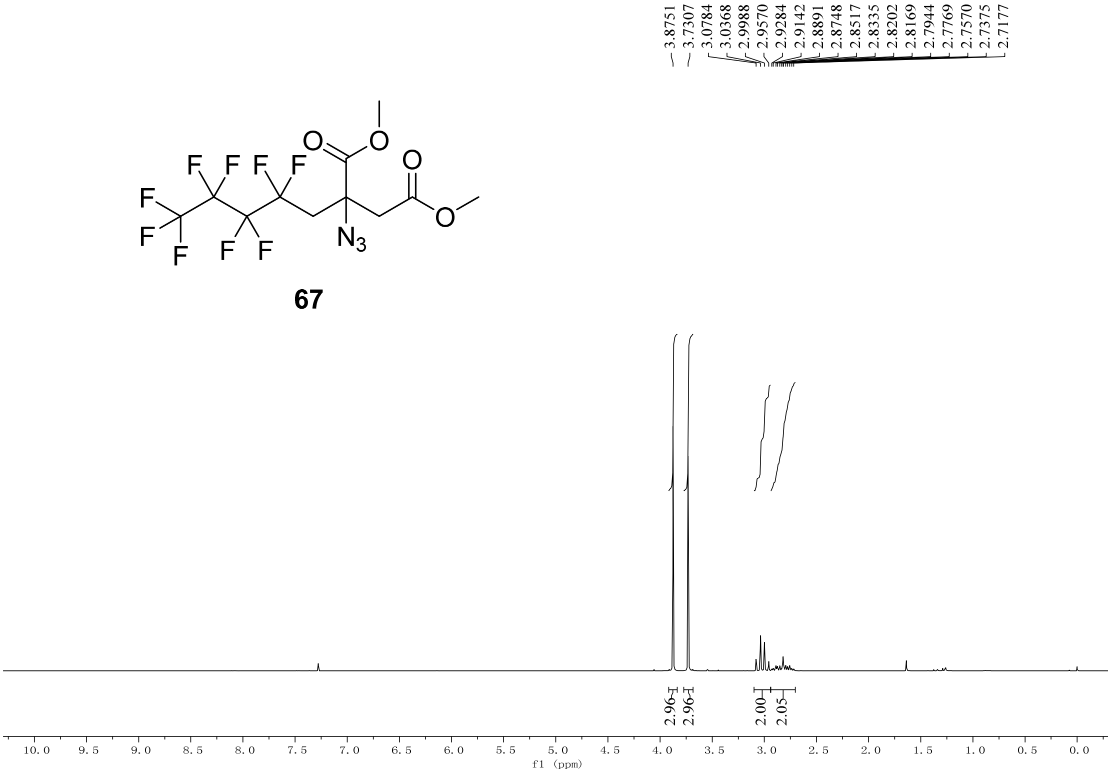

Supplementary Figure 164. ^{1}H NMR spectrum for compound **67**

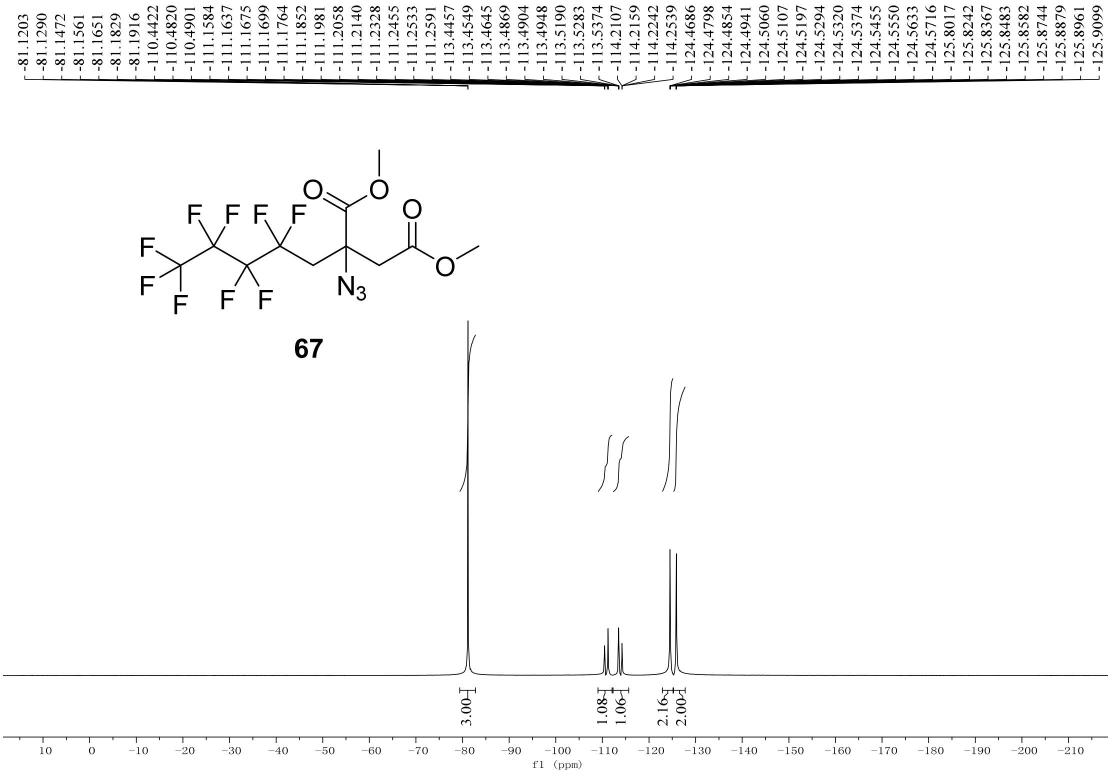

Supplementary Figure 165. ^{19}F NMR spectrum for compound **67**

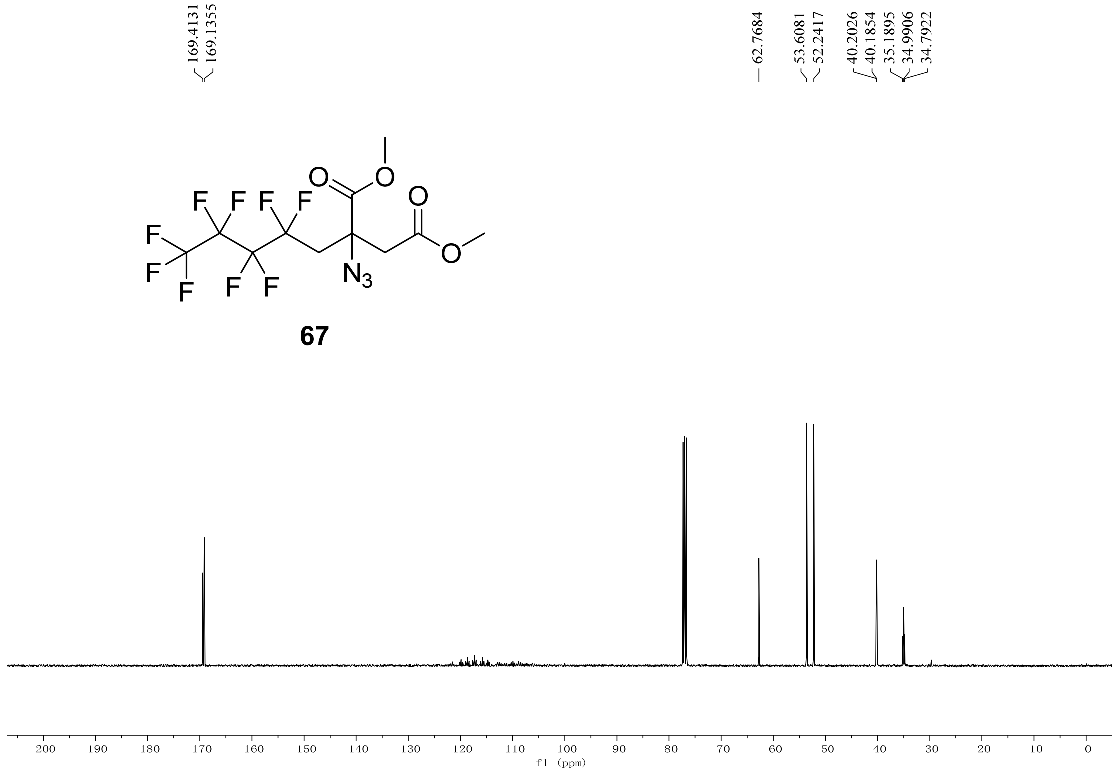

Supplementary Figure 166. ^{13}C NMR spectrum for compound **67**

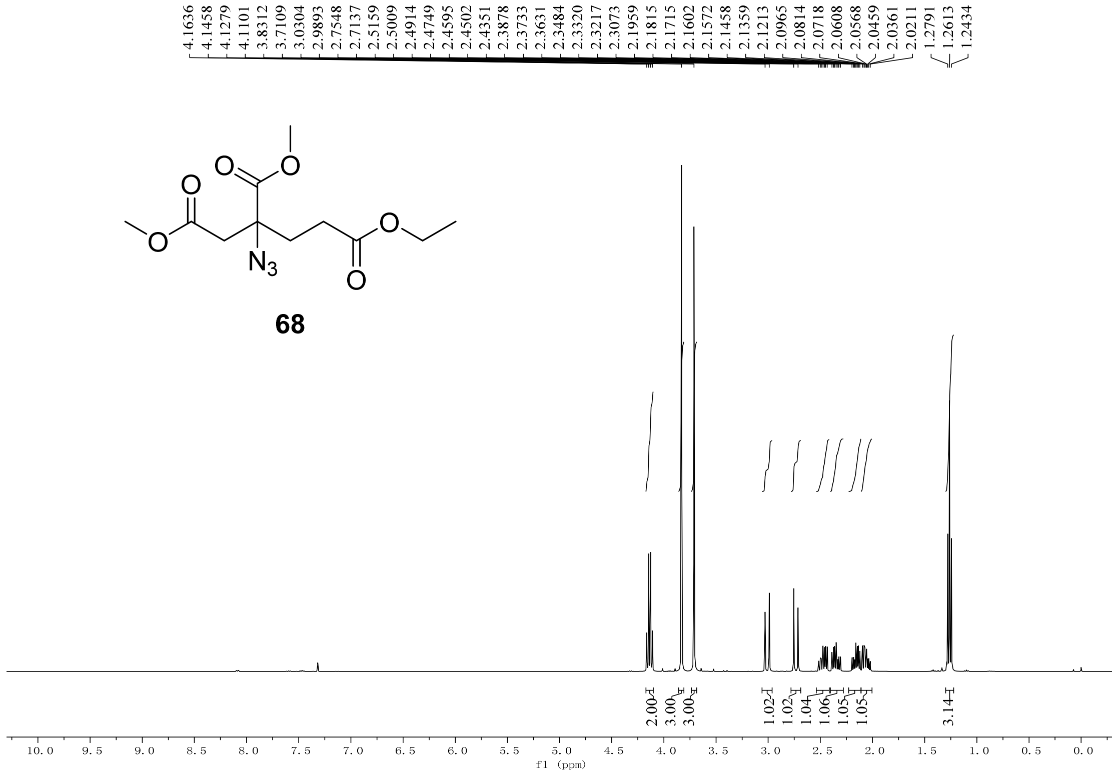

Supplementary Figure 167. ^{1}H NMR spectrum for compound **68**

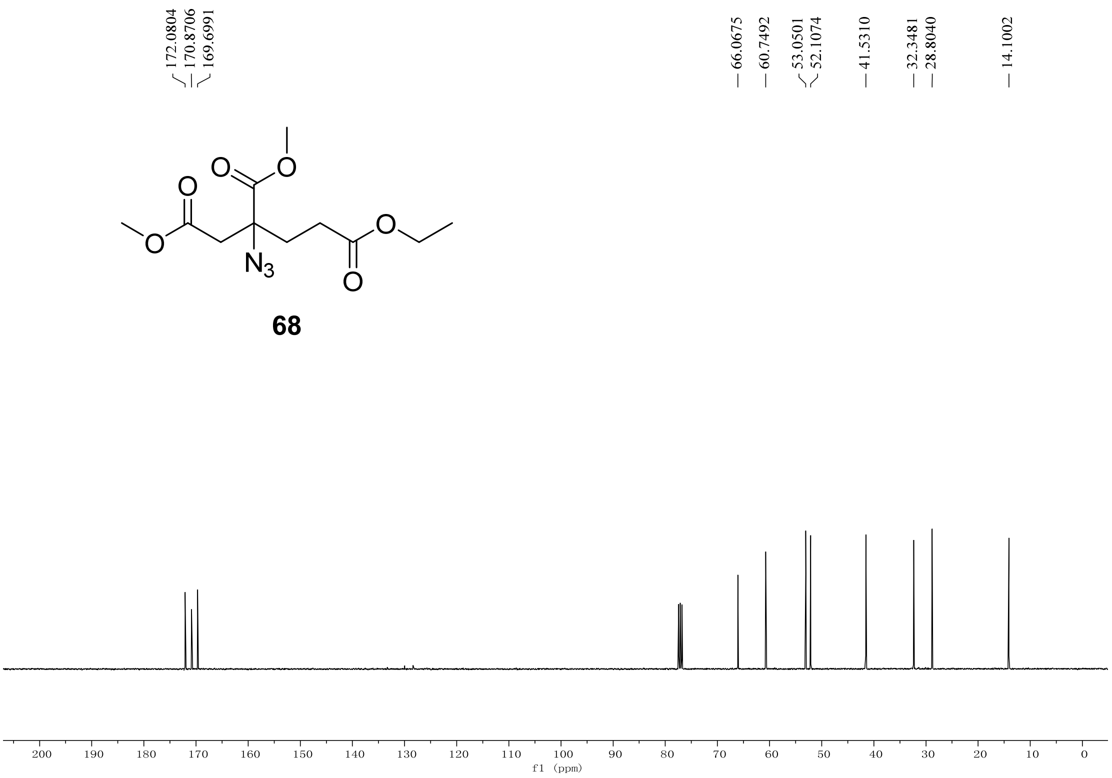

Supplementary Figure 168. ^{13}C NMR spectrum for compound **68**

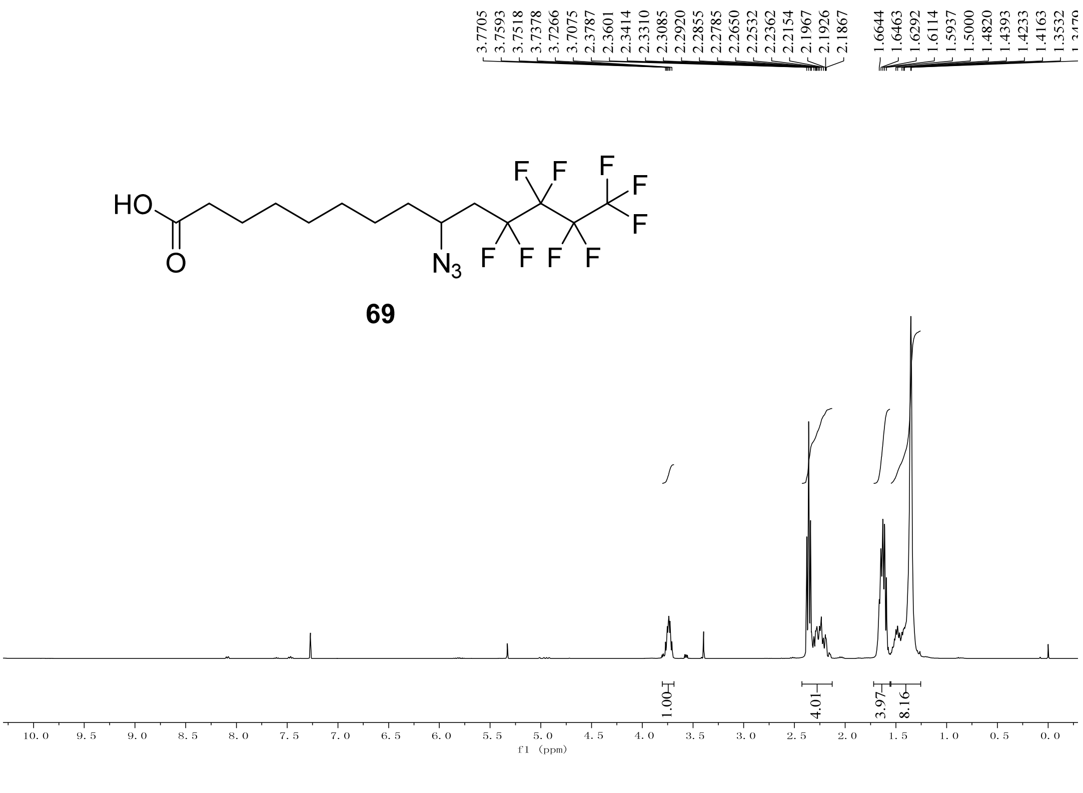

Supplementary Figure 169. ^{1}H NMR spectrum for compound **69**

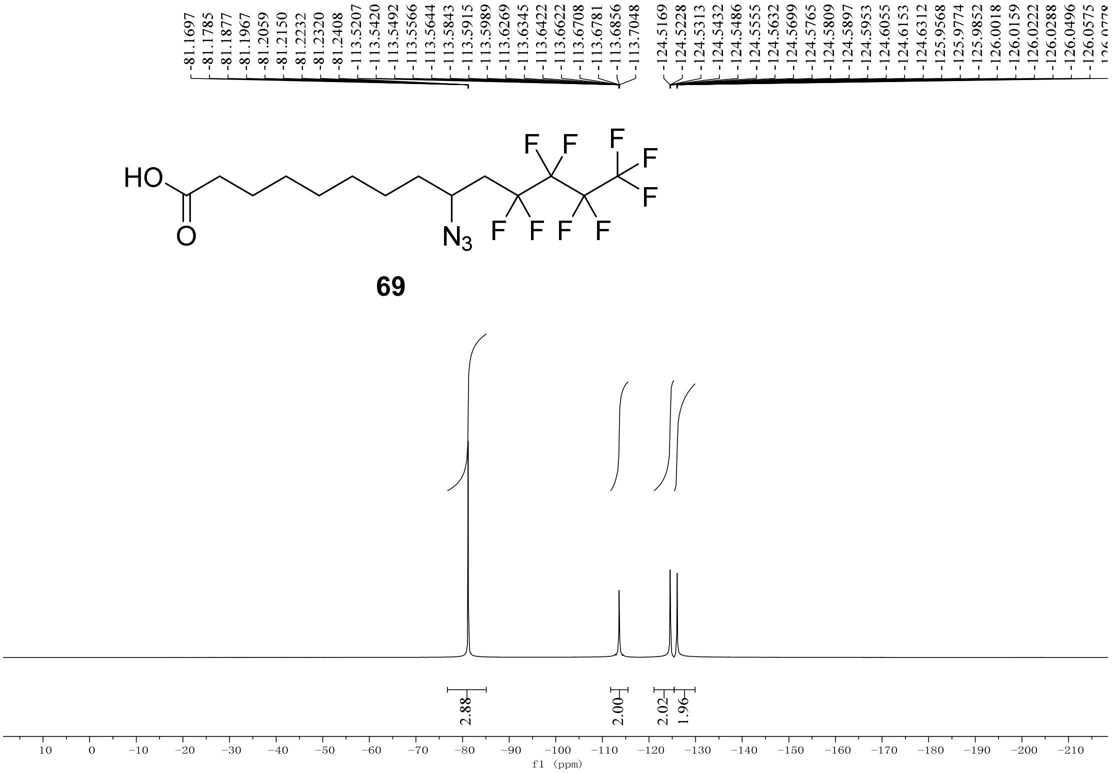

Supplementary Figure 170. ^{19}F NMR spectrum for compound **69**

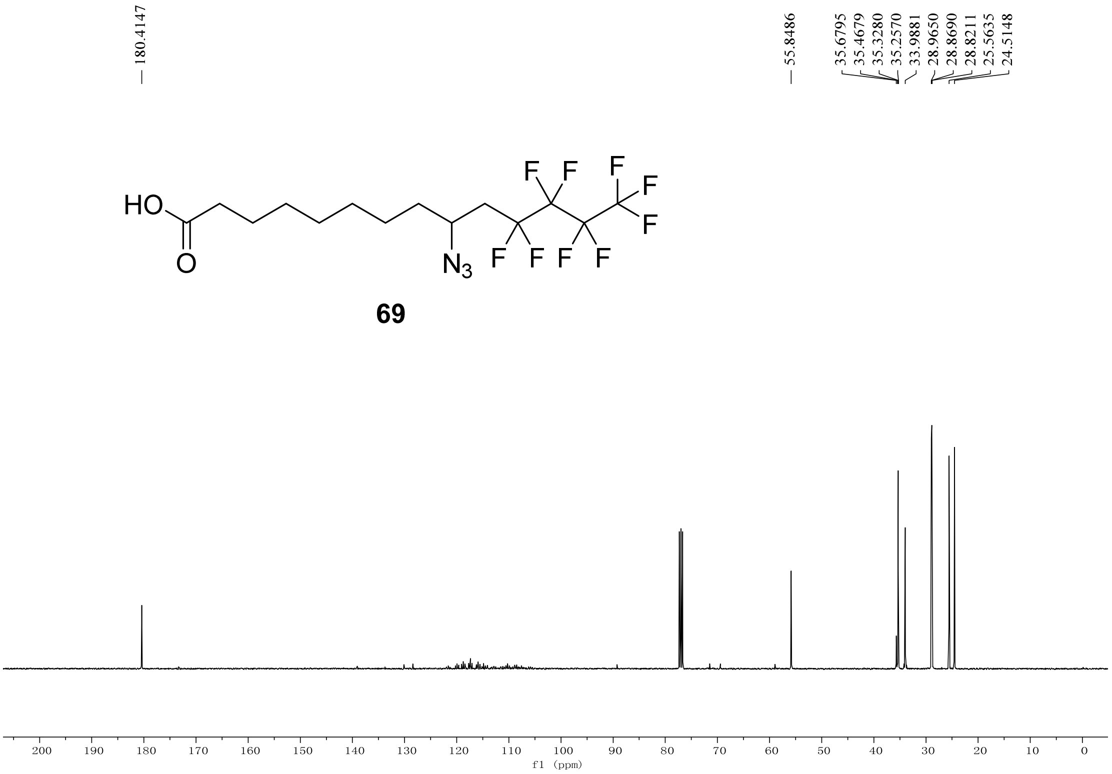

Supplementary Figure 171. ^{13}C NMR spectrum for compound **69**

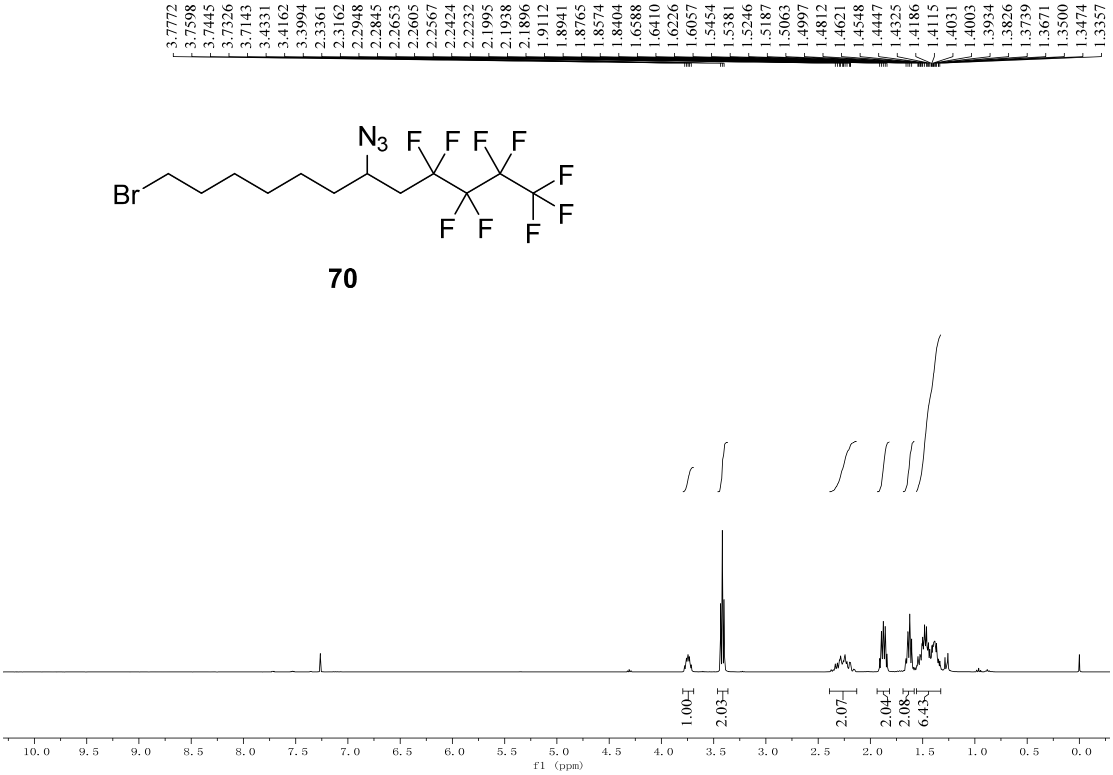

Supplementary Figure 172. ^{1}H NMR spectrum for compound **70**

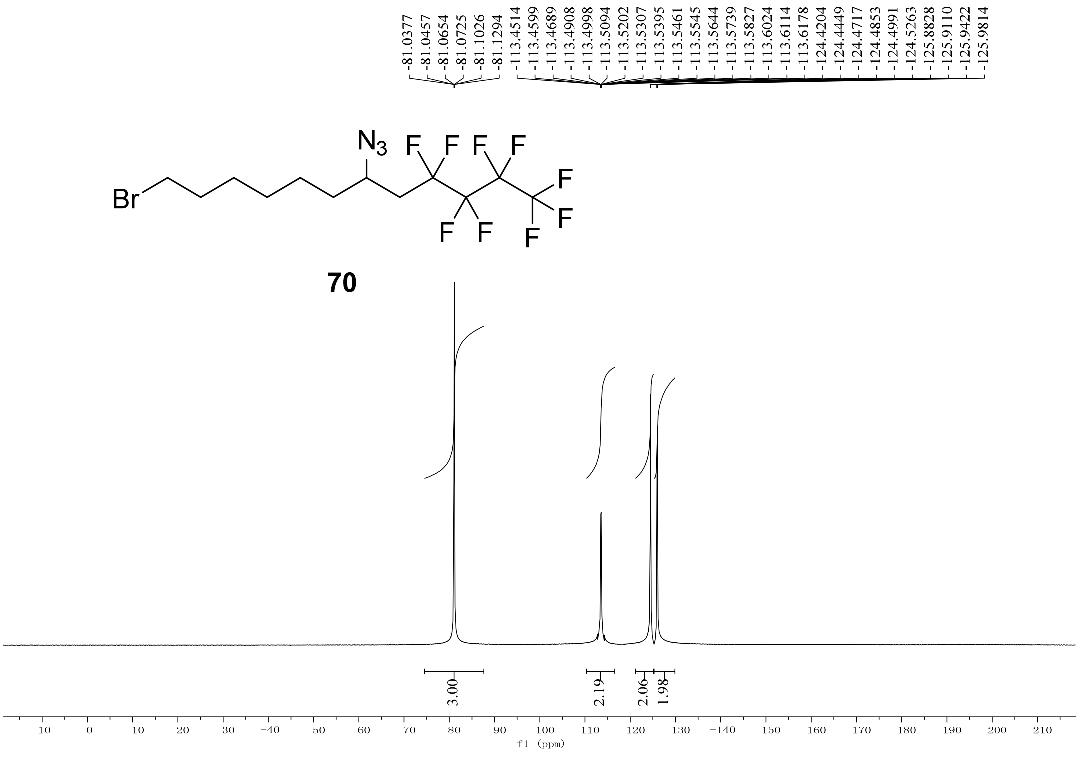

Supplementary Figure 173. ^{19}F NMR spectrum for compound **70**

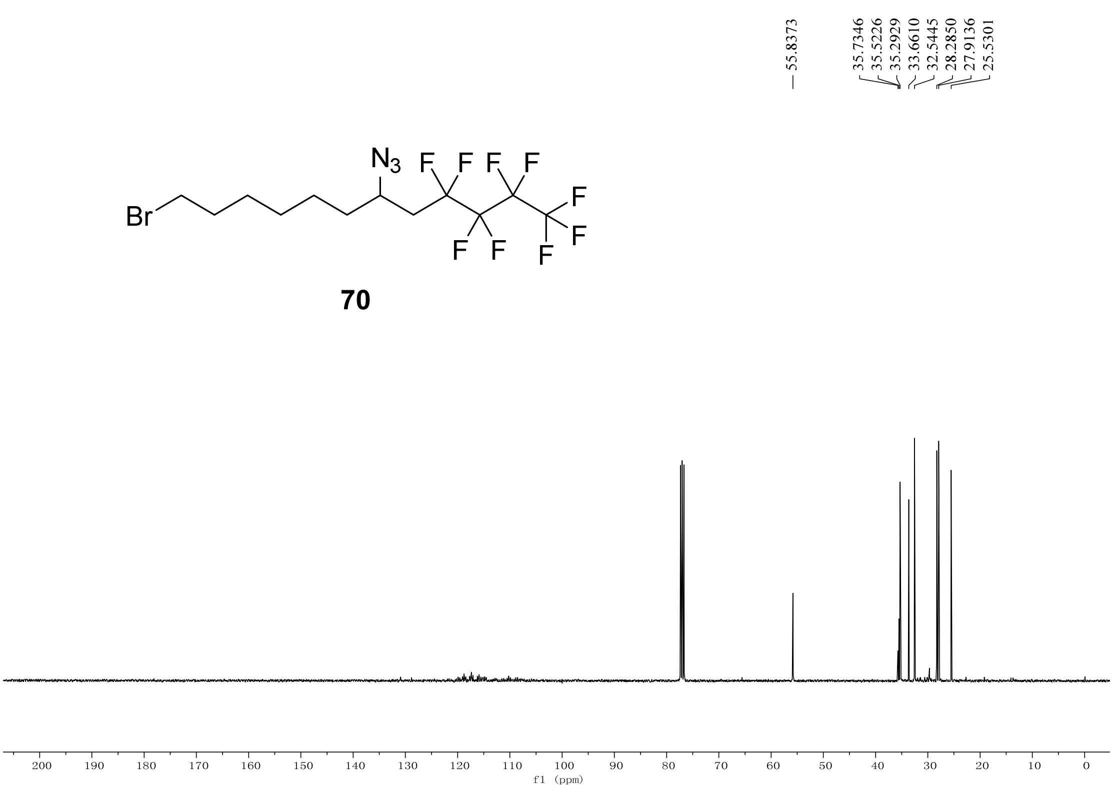

Supplementary Figure 174. ^{13}C NMR spectrum for compound **70**

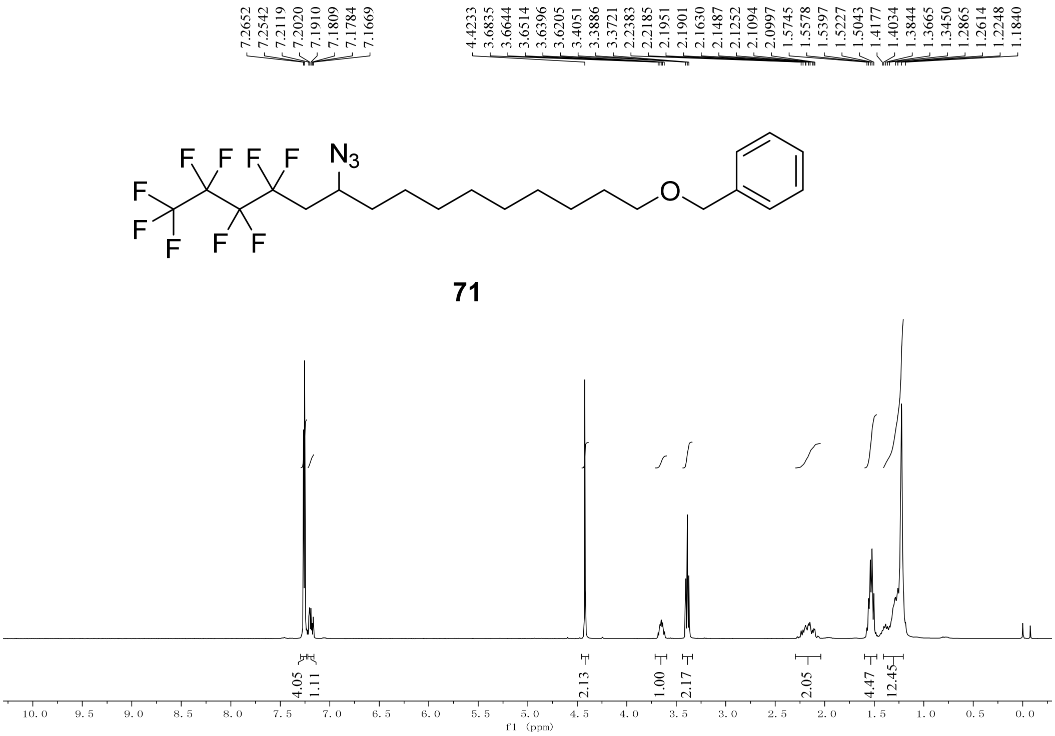

Supplementary Figure 175. ^{1}H NMR spectrum for compound **71**

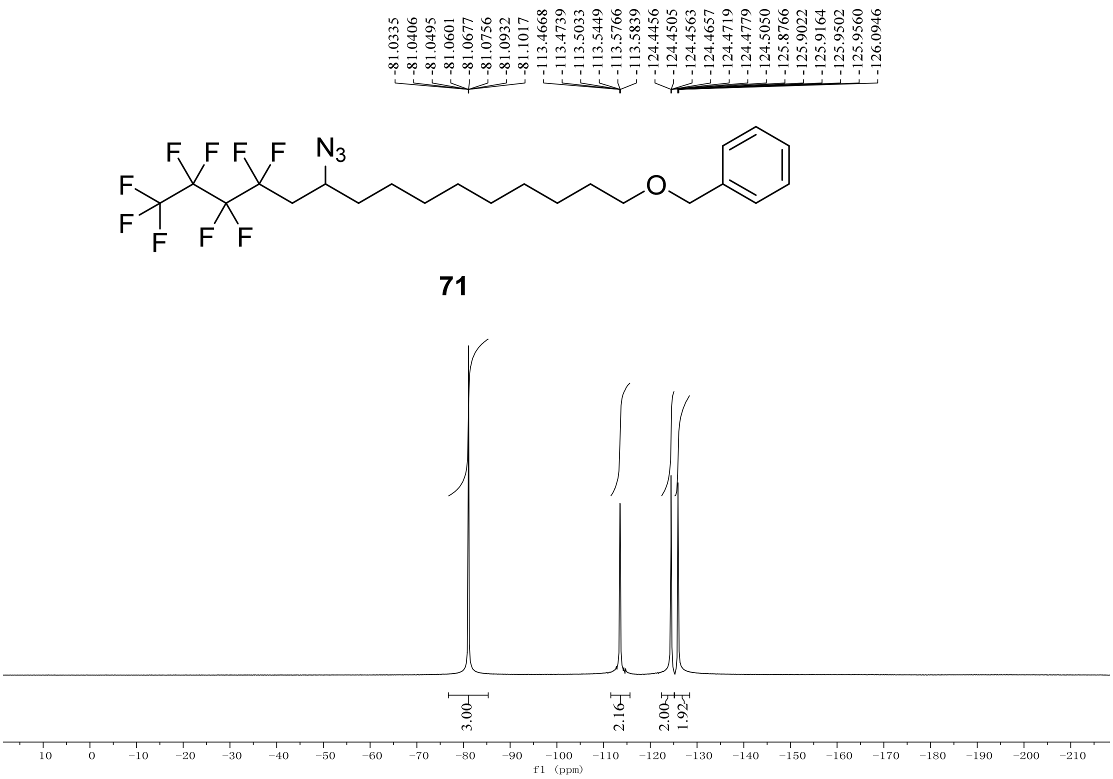

Supplementary Figure 176. ^{19}F NMR spectrum for compound **71**

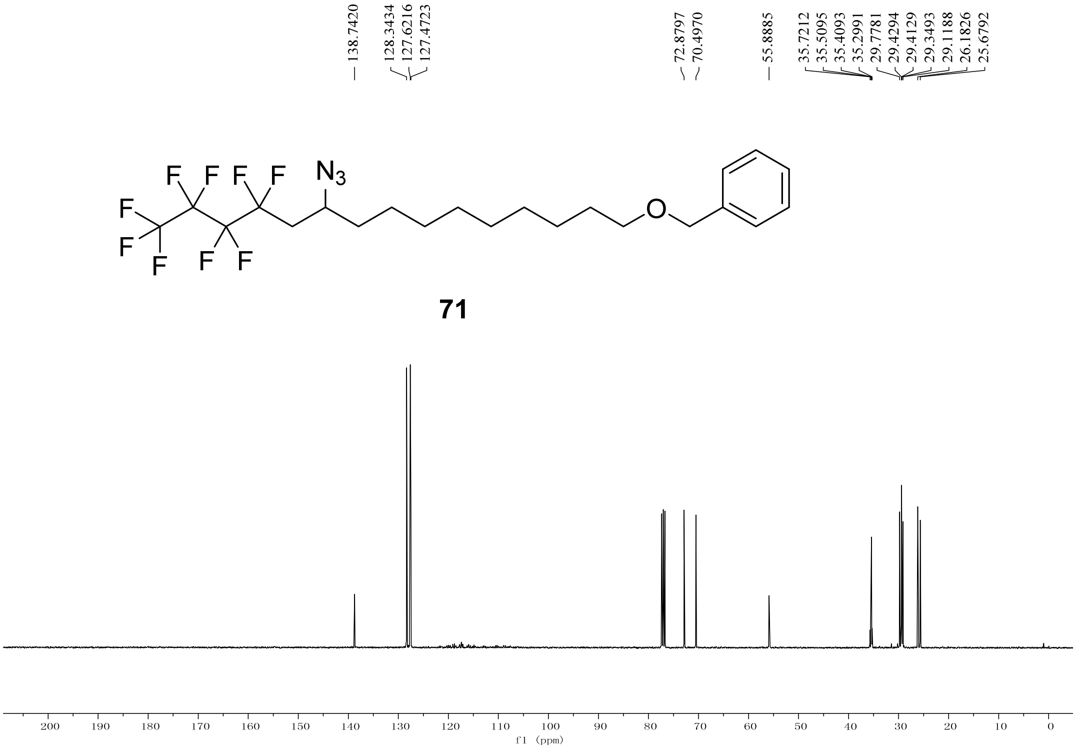

Supplementary Figure 177. ^{13}C NMR spectrum for compound **71**

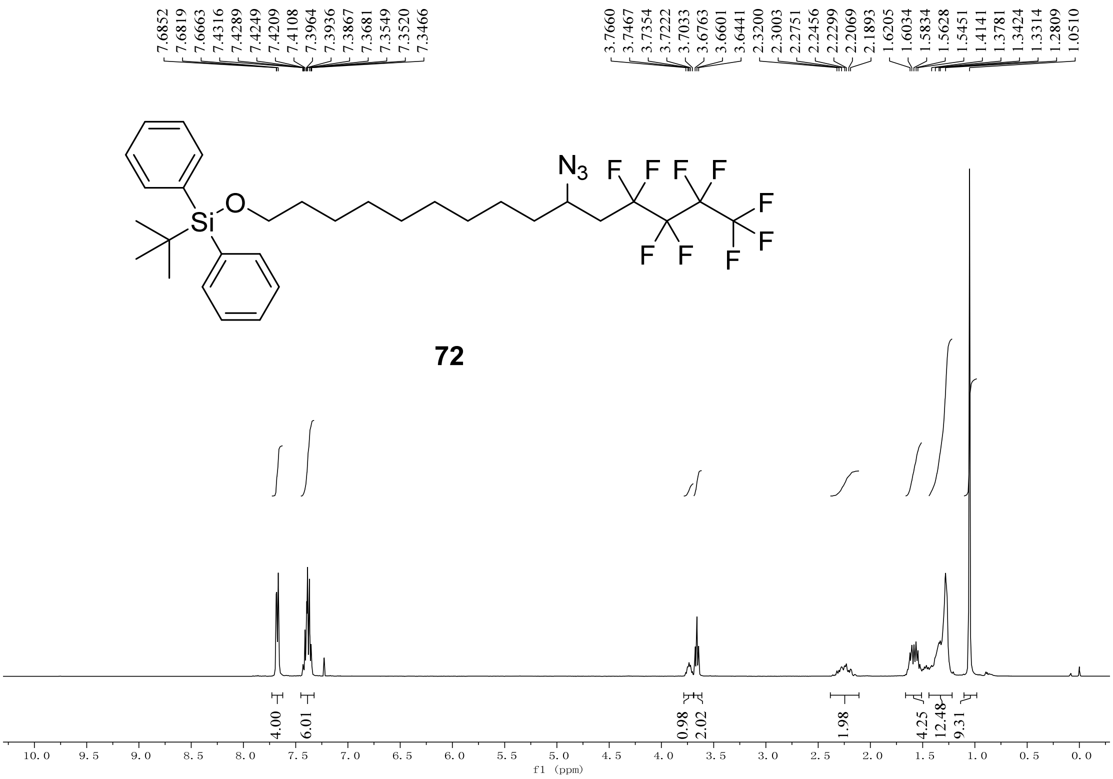

Supplementary Figure 178. ^{1}H NMR spectrum for compound **72**

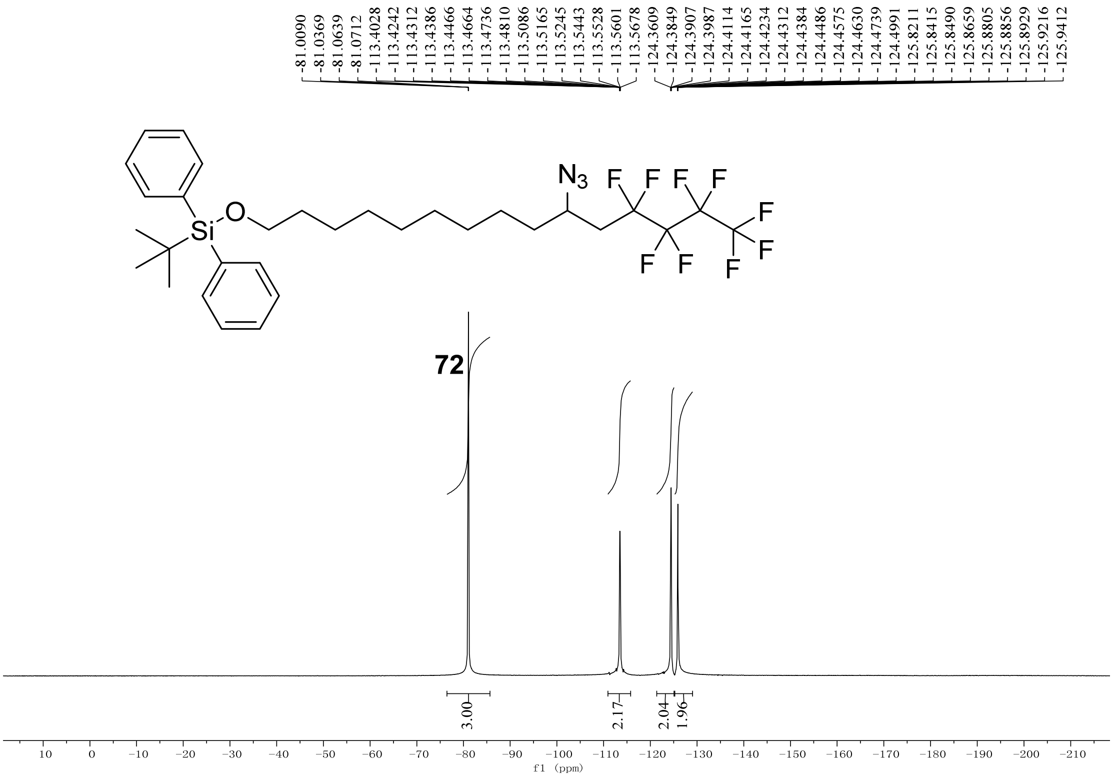

Supplementary Figure 179. ^{19}F NMR spectrum for compound **72**

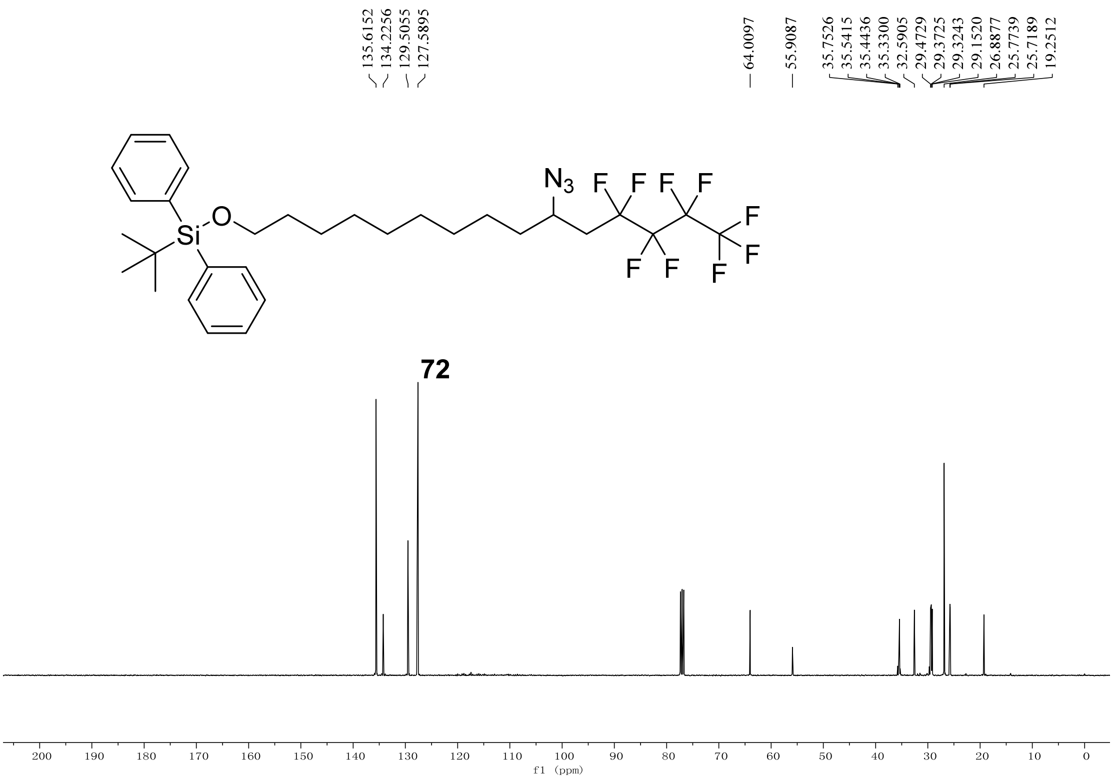

Supplementary Figure 180. ^{13}C NMR spectrum for compound **72**

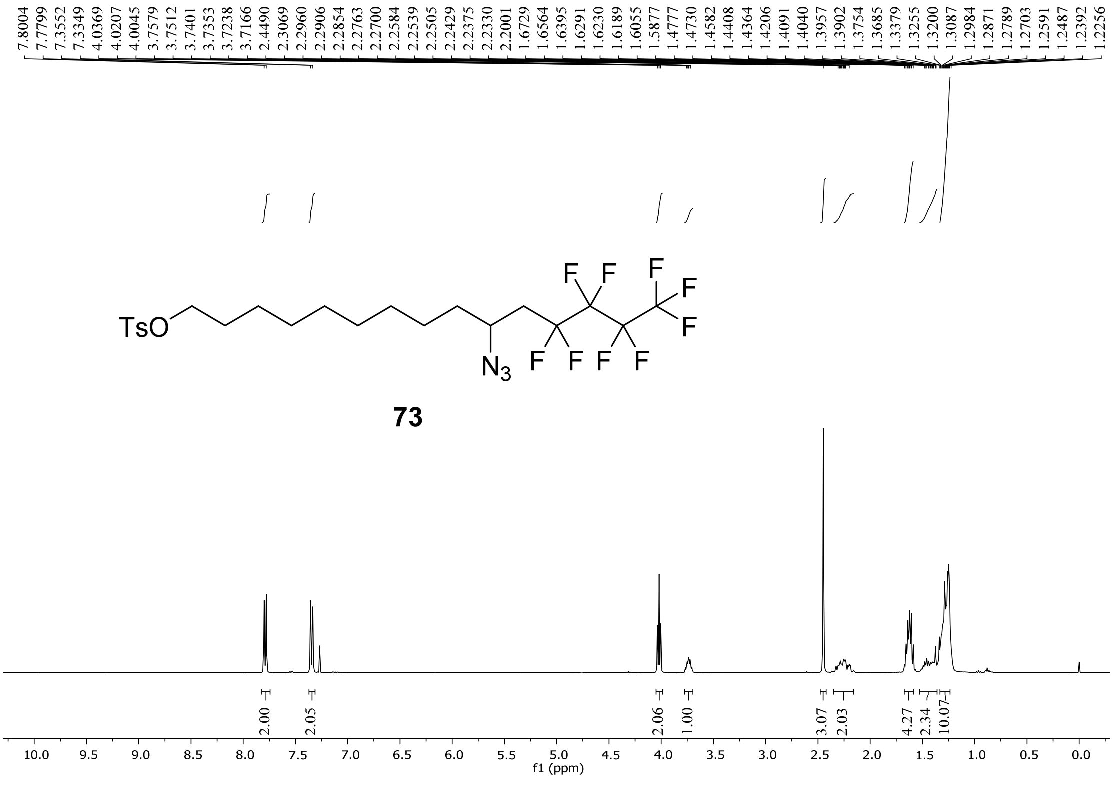

Supplementary Figure 181. ^{1}H NMR spectrum for compound **73**

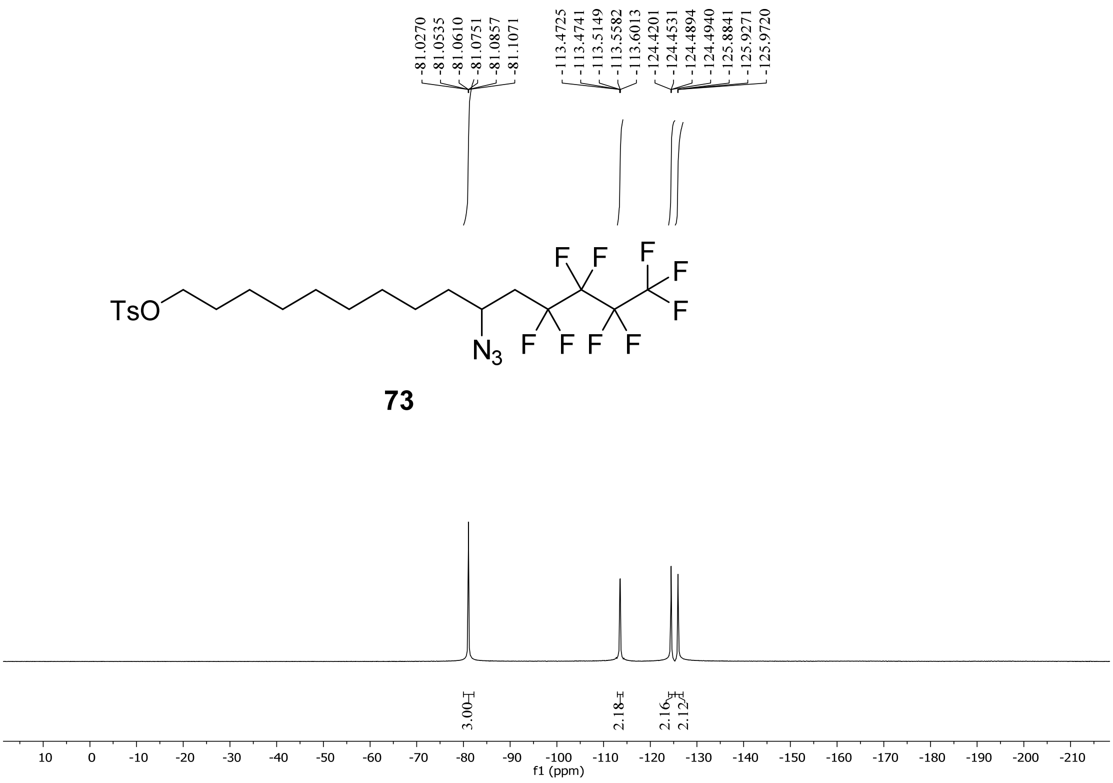

Supplementary Figure 182. ^{19}F NMR spectrum for compound **73**

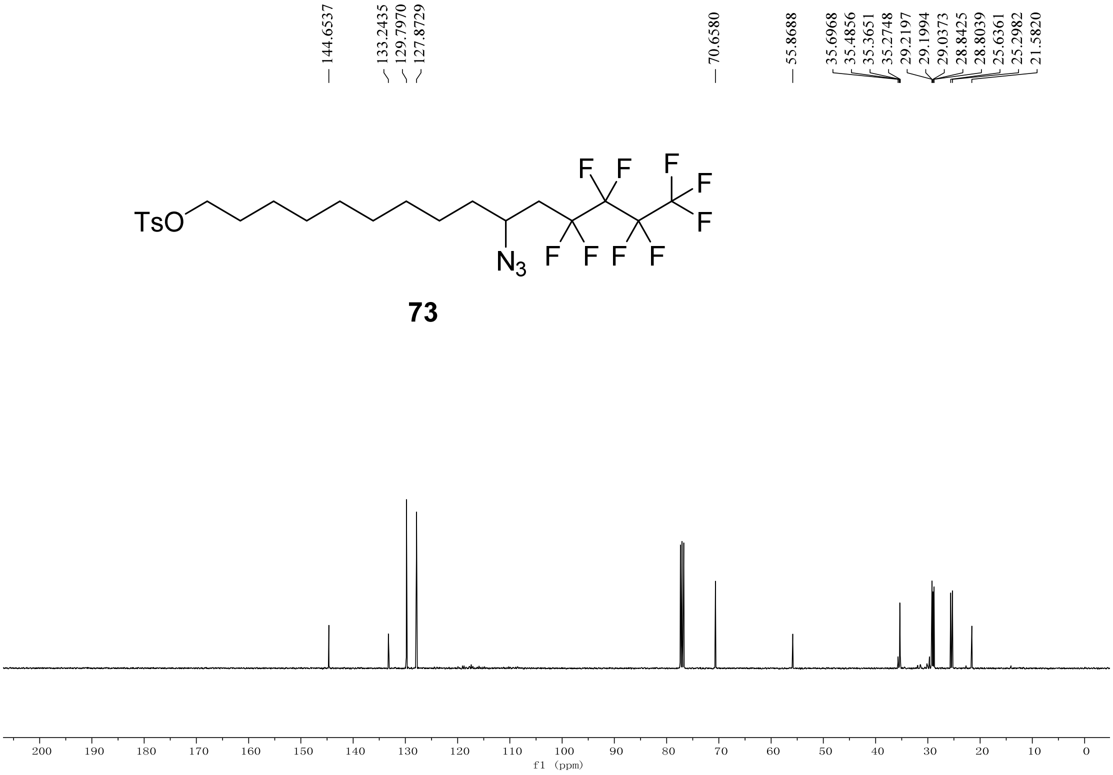

Supplementary Figure 183. ^{13}C NMR spectrum for compound **73**

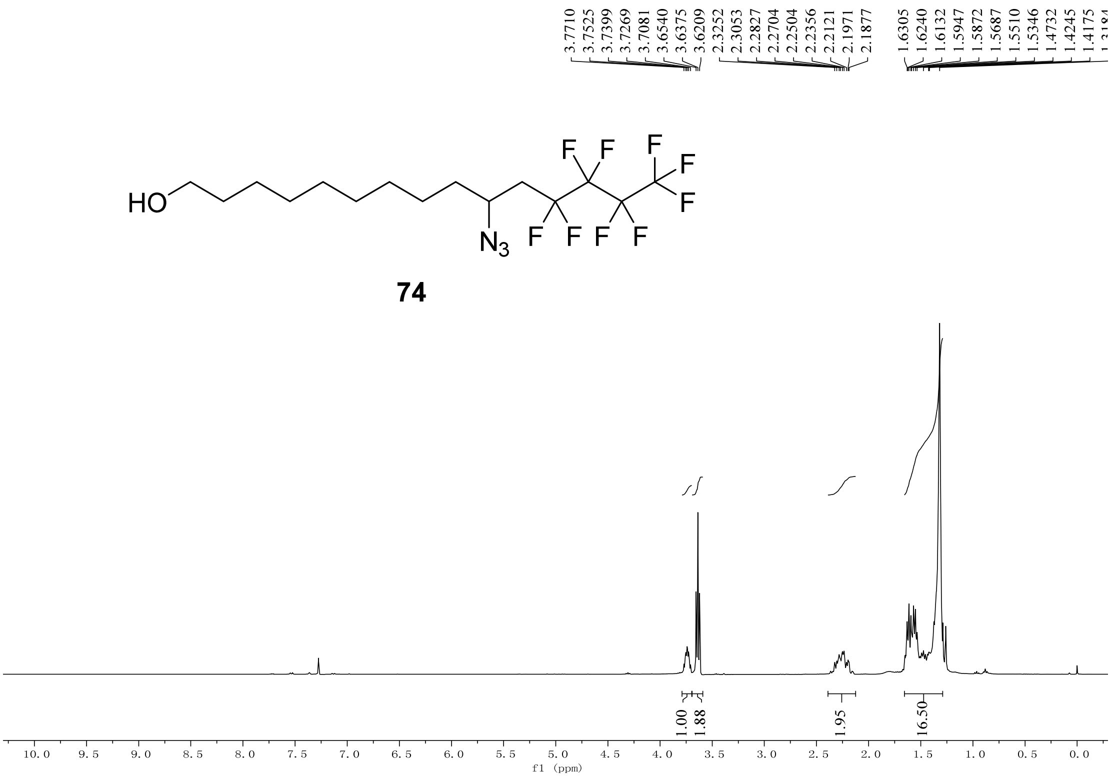

Supplementary Figure 184. ^{1}H NMR spectrum for compound **74**

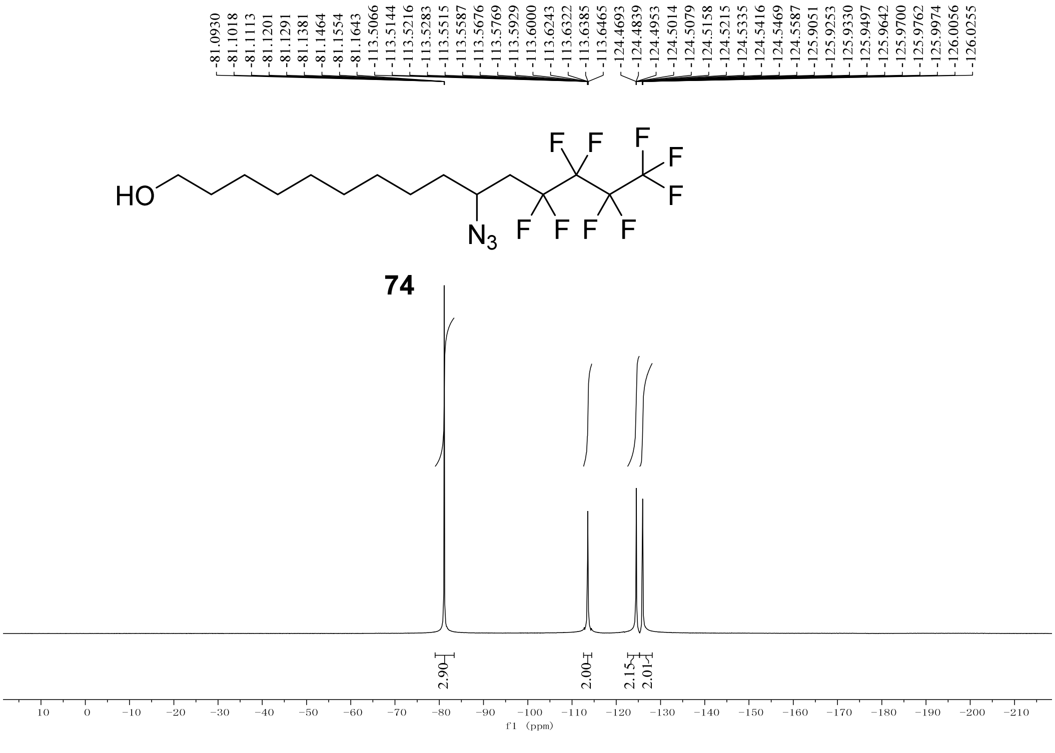

Supplementary Figure 185. ^{19}F NMR spectrum for compound **74**

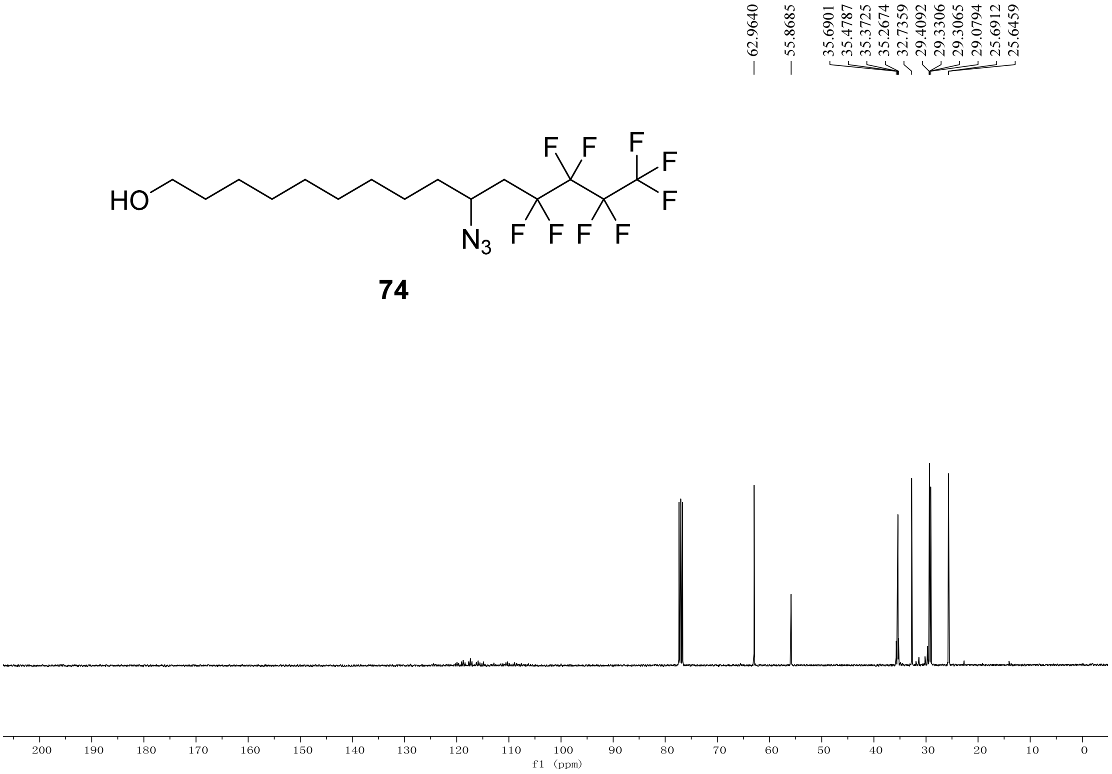

Supplementary Figure 186. ^{13}C NMR spectrum for compound **74**

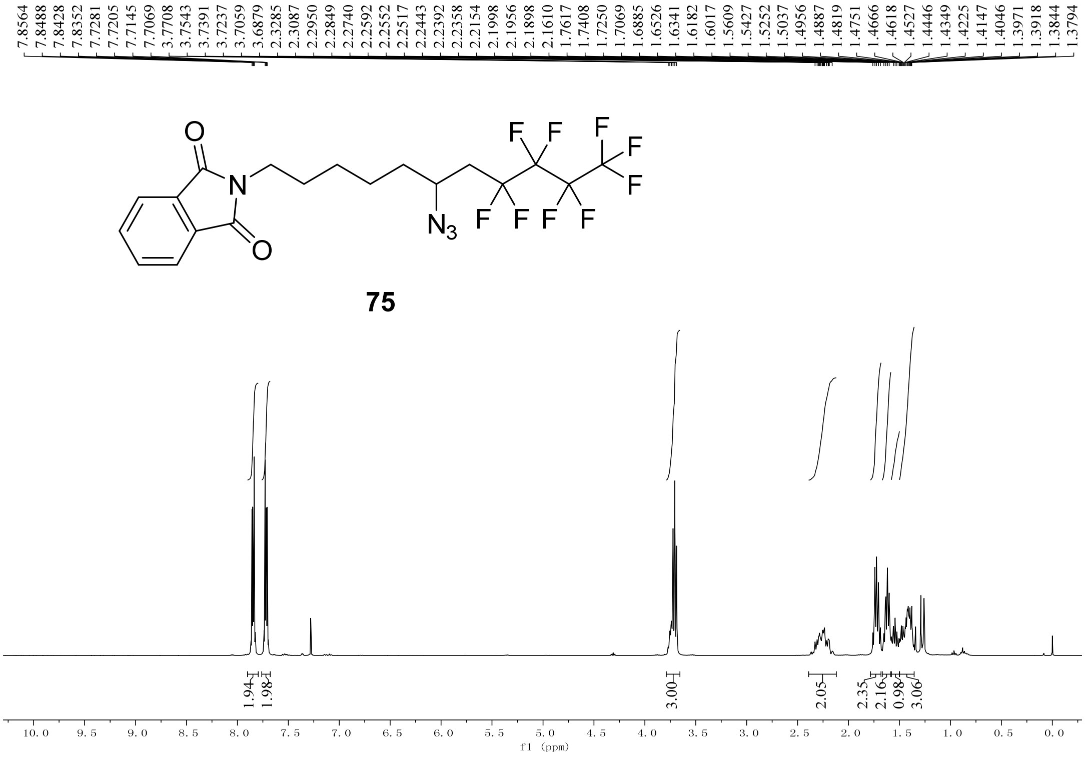

Supplementary Figure 187. ^{1}H NMR spectrum for compound **75**

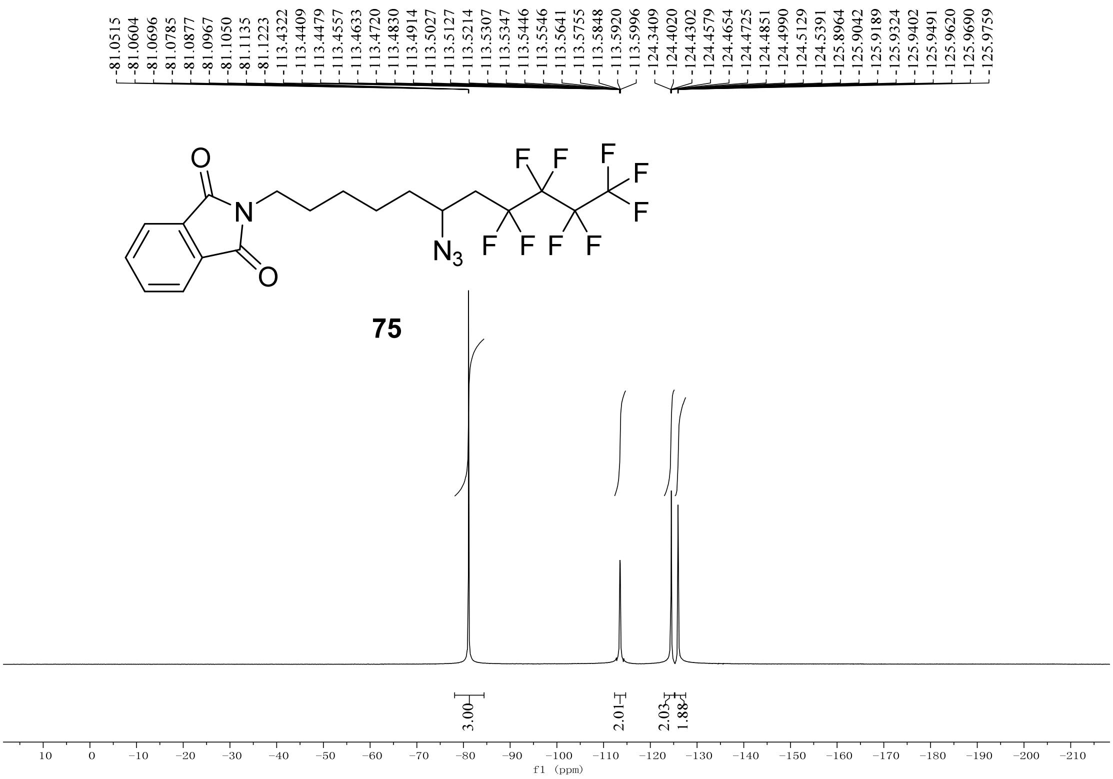

Supplementary Figure 188. ^{19}F NMR spectrum for compound **75**

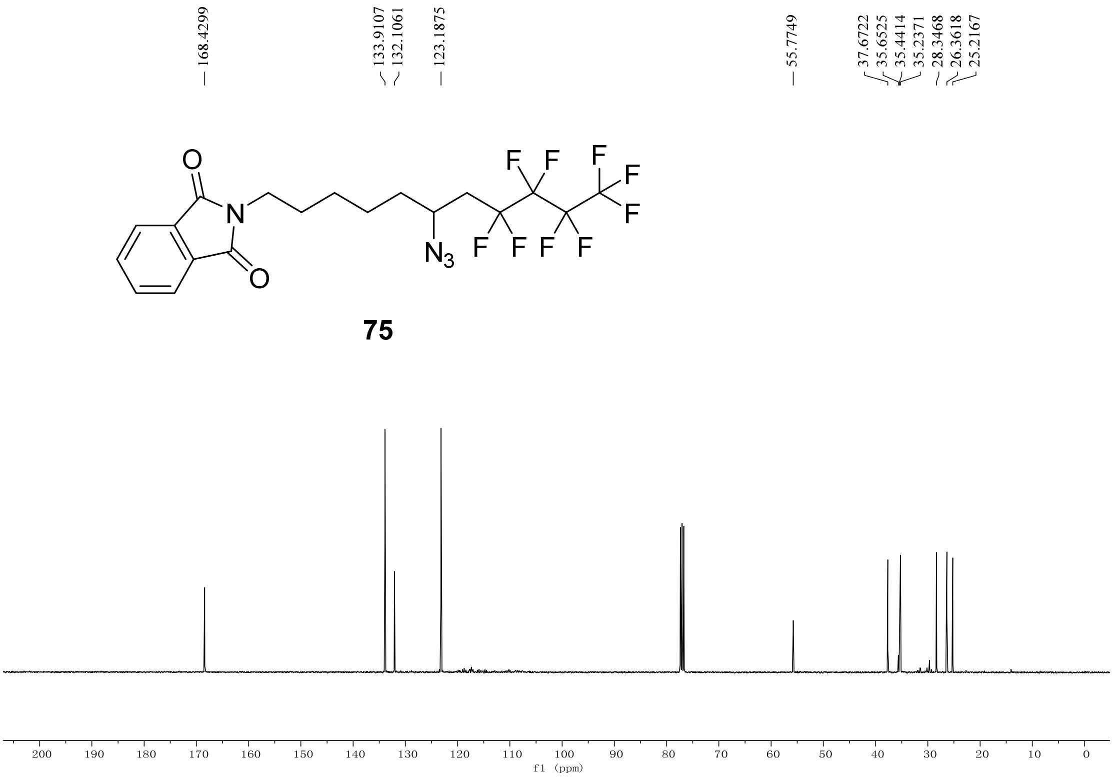

Supplementary Figure 189. ^{13}C NMR spectrum for compound **75**

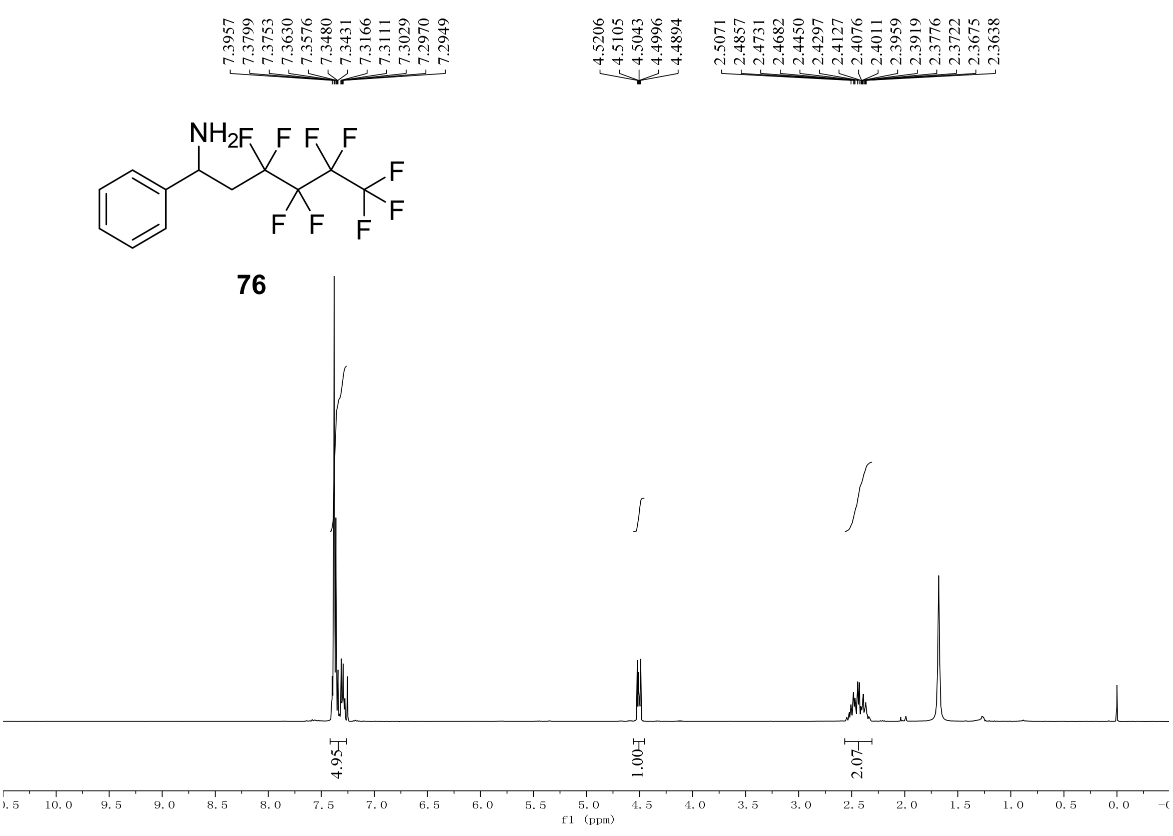

Supplementary Figure 190. ^{1}H NMR spectrum for compound **76**

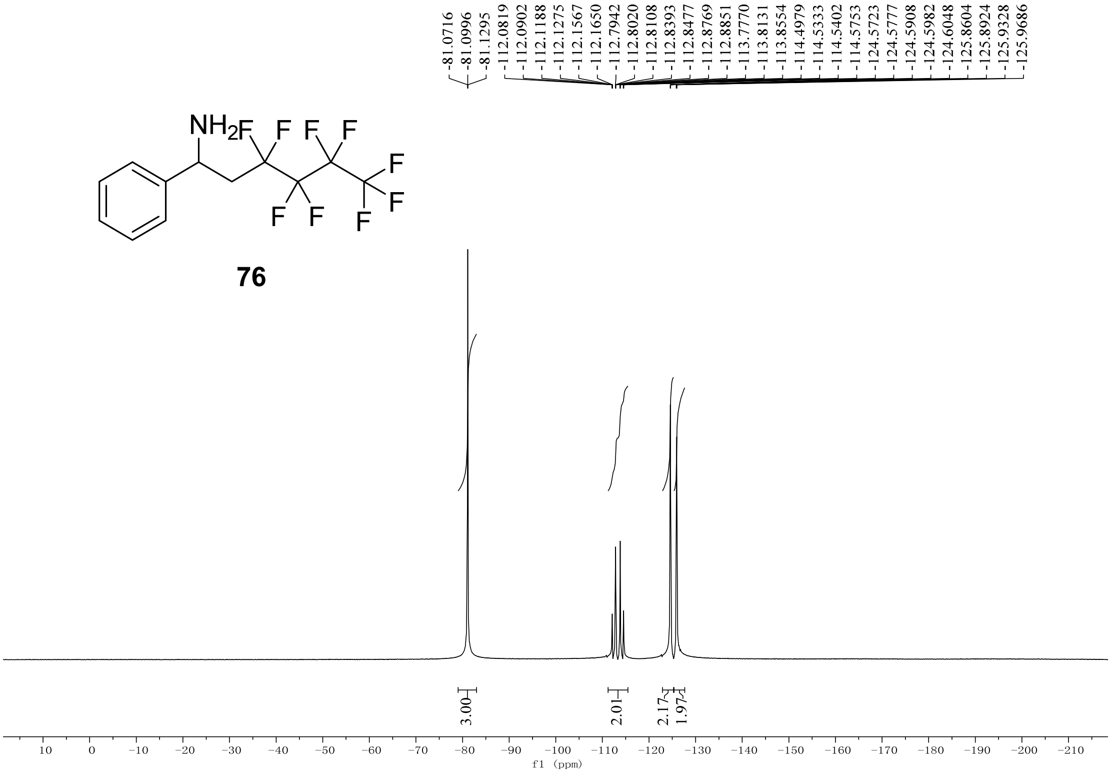

Supplementary Figure 191. ^{19}F NMR spectrum for compound **76**

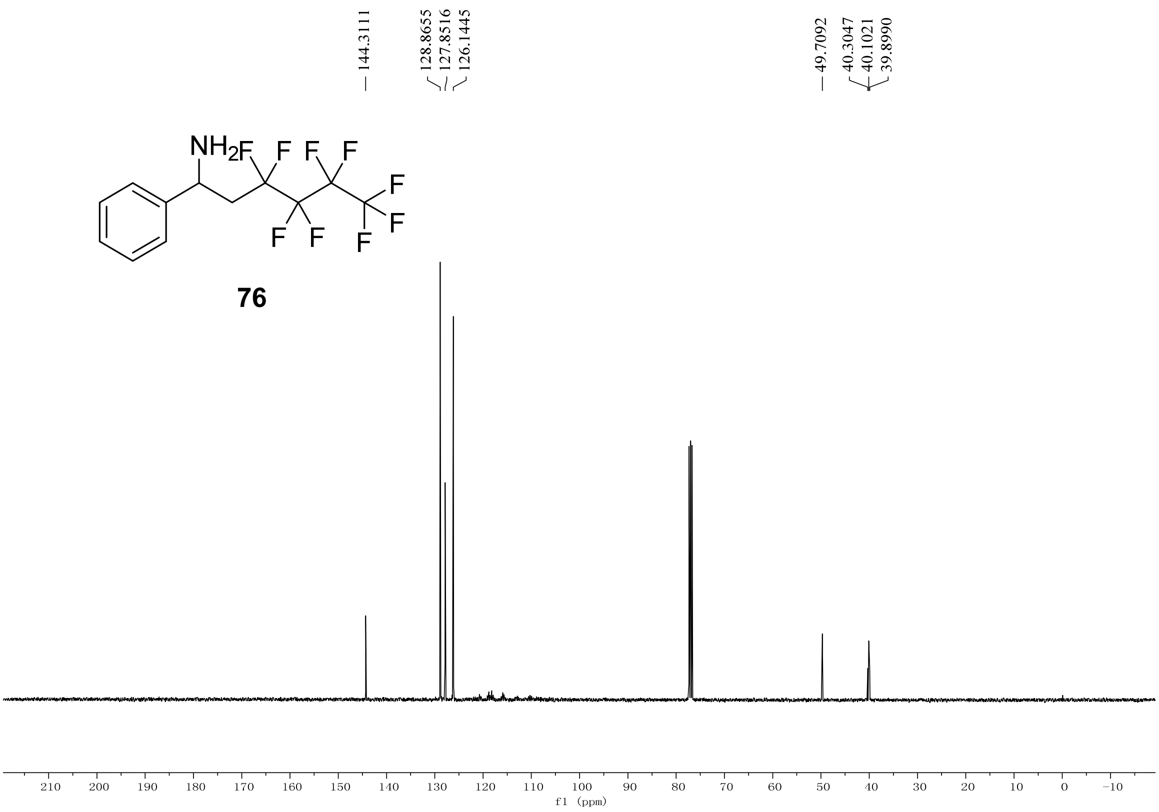

Supplementary Figure 192. ^{13}C NMR spectrum for compound **76**

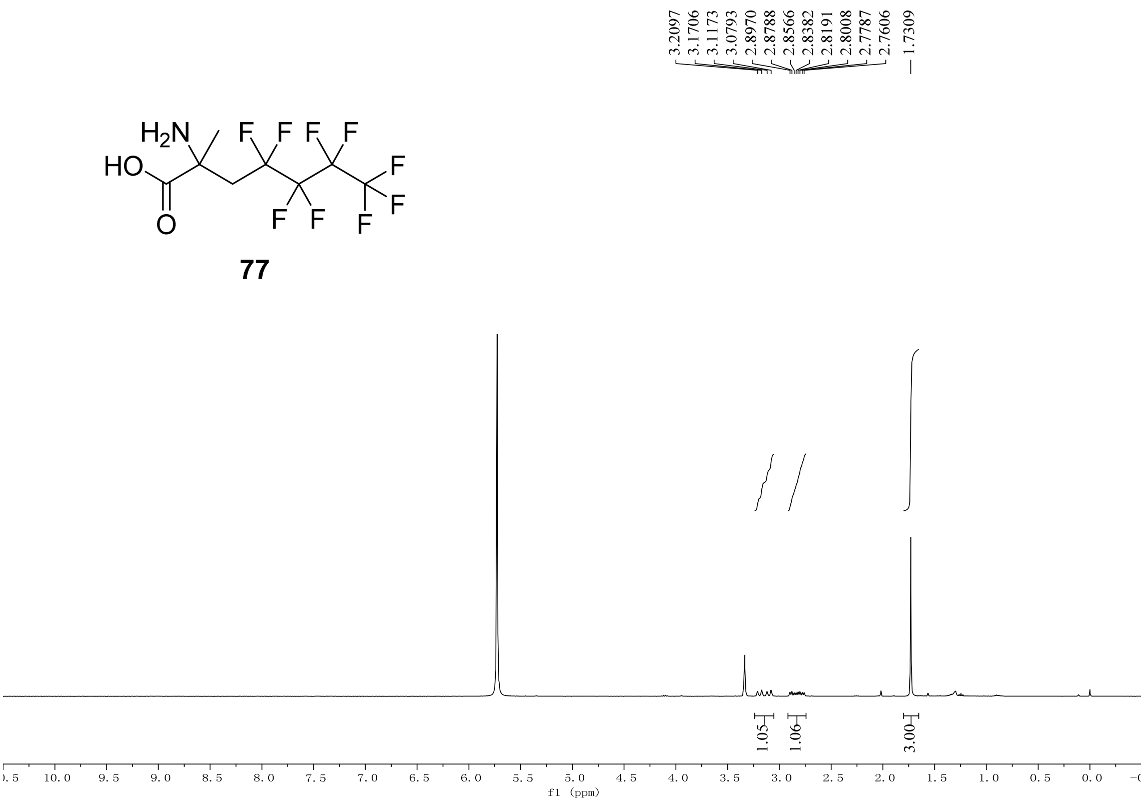

Supplementary Figure 193. ^{1}H NMR spectrum for compound **77**

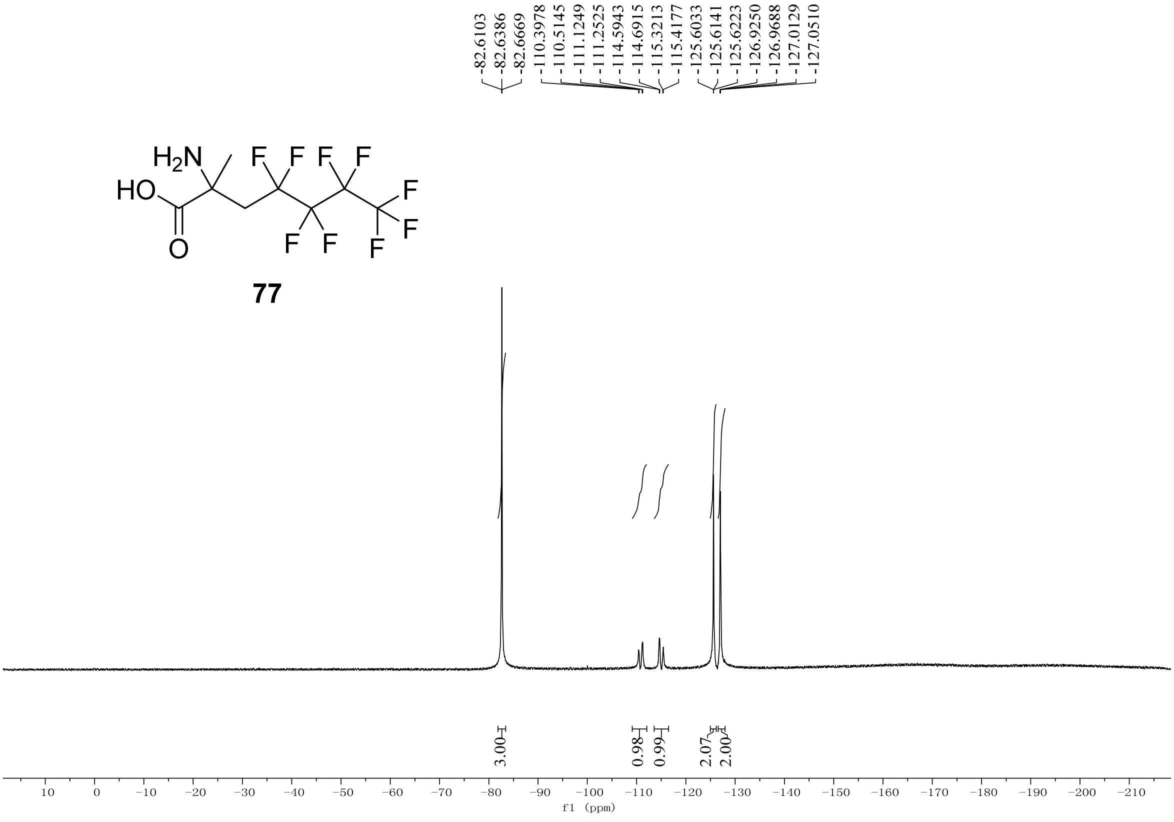

Supplementary Figure 194. ^{19}F NMR spectrum for compound **77**

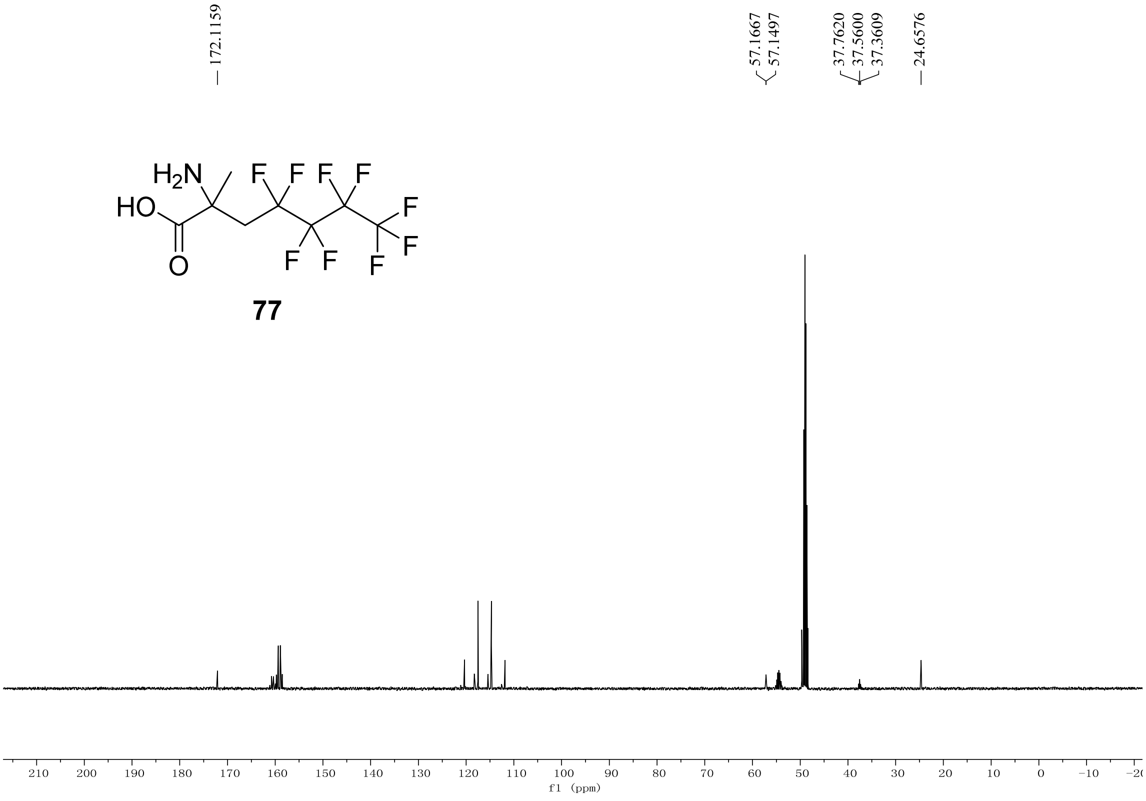

Supplementary Figure 195. ^{13}C NMR spectrum for compound **77**

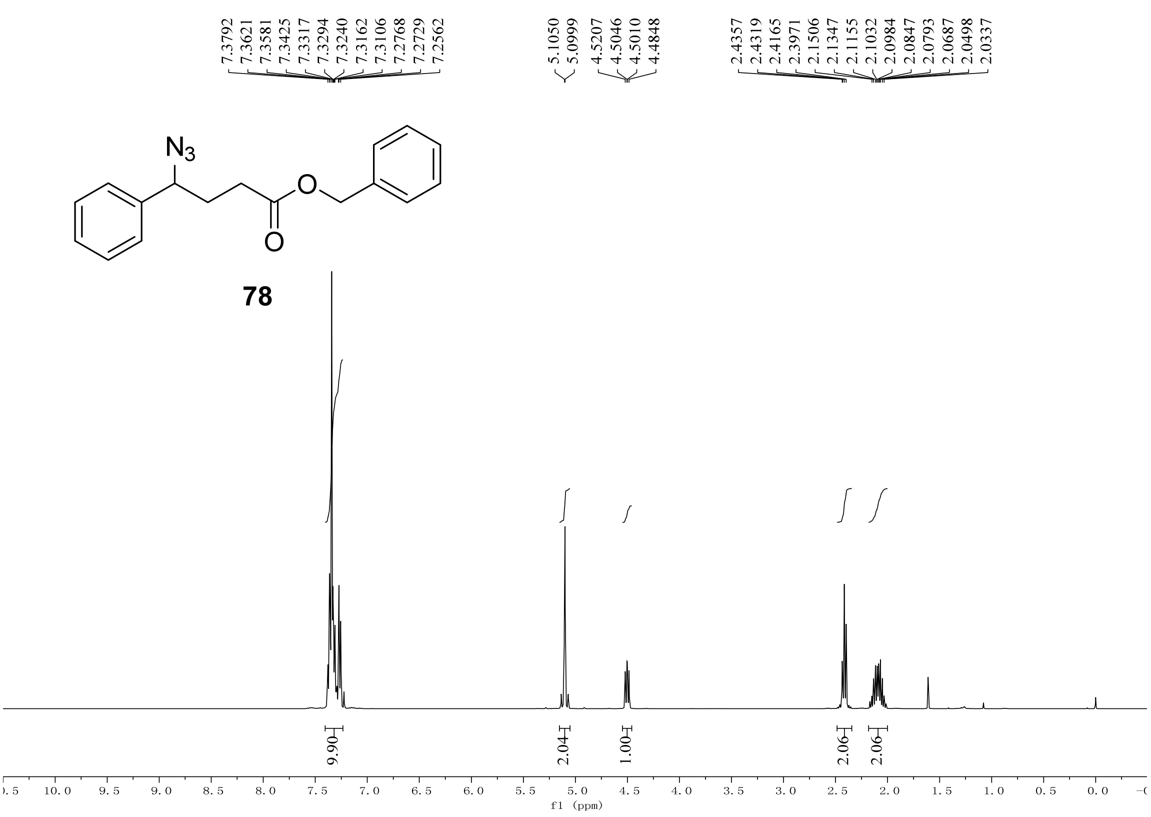

Supplementary Figure 196. ^{1}H NMR spectrum for compound **78**

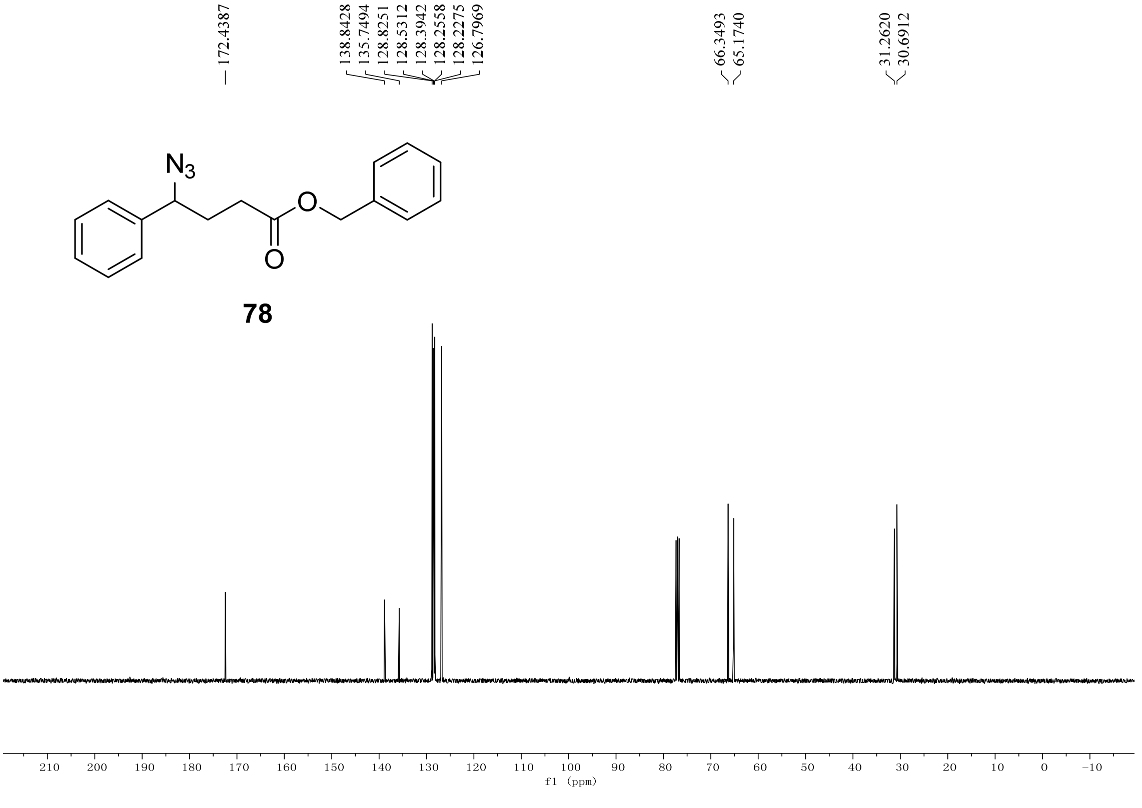

Supplementary Figure 197. ^{13}C NMR spectrum for compound **78**

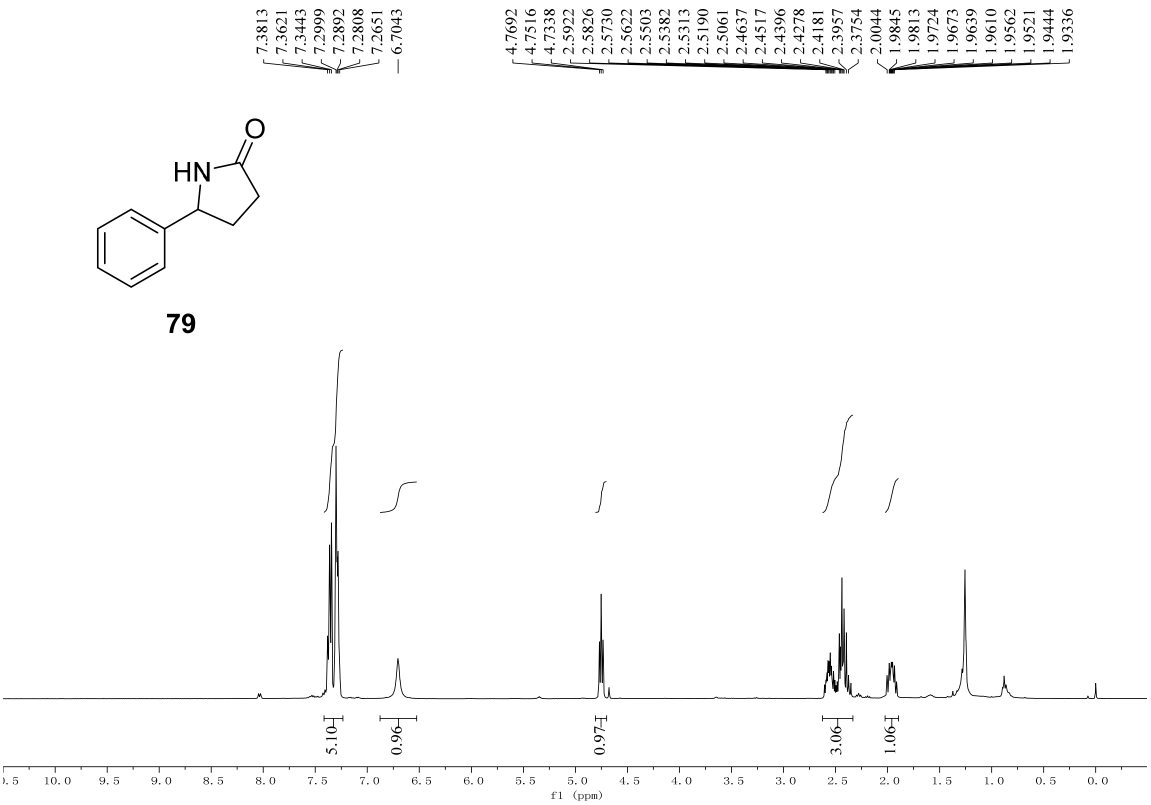

Supplementary Figure 198. ^{1}H NMR spectrum for compound **79**

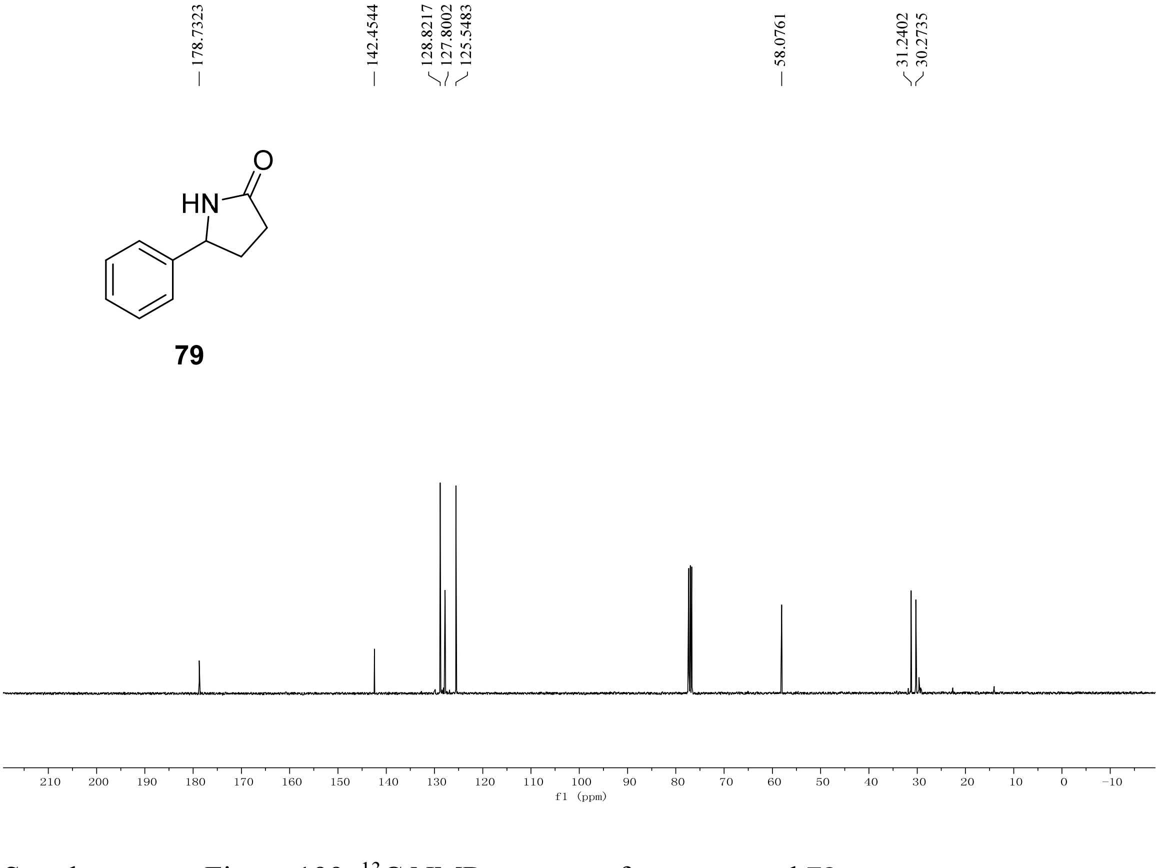

Supplementary Figure 199. ^{13}C NMR spectrum for compound **79**

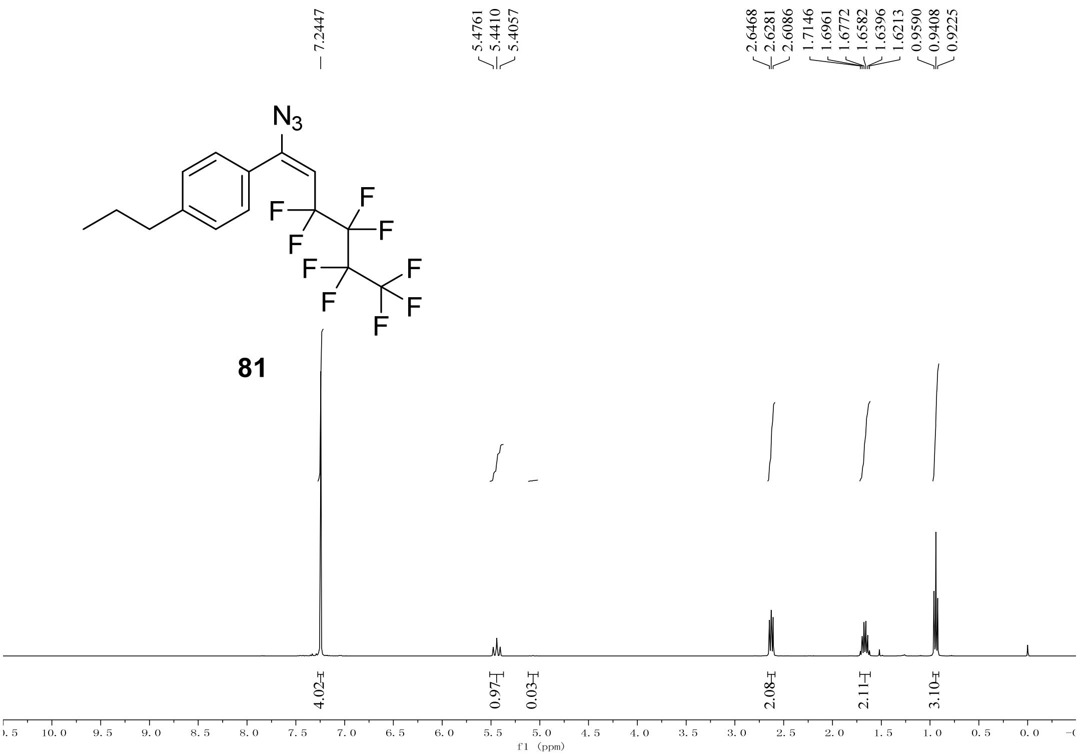

Supplementary Figure 200. ^{1}H NMR spectrum for compound **81**

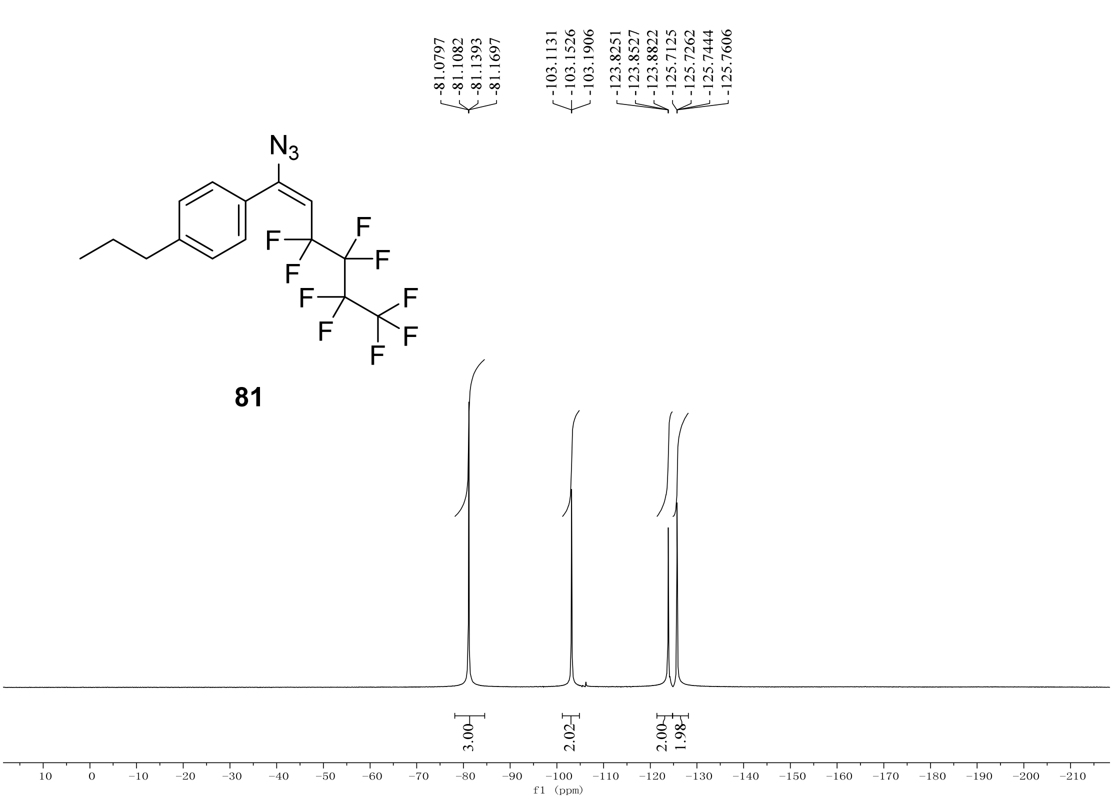

Supplementary Figure 201. ^{19}F NMR spectrum for compound **81**

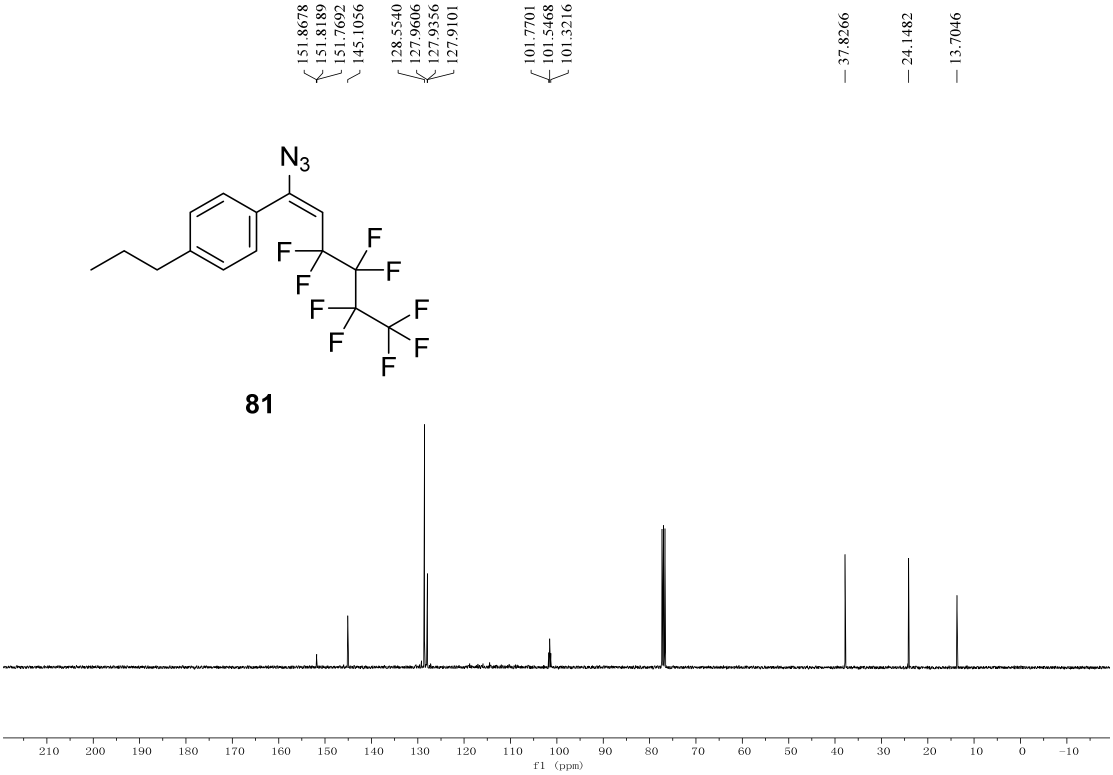

Supplementary Figure 202. ^{13}C NMR spectrum for compound **81**

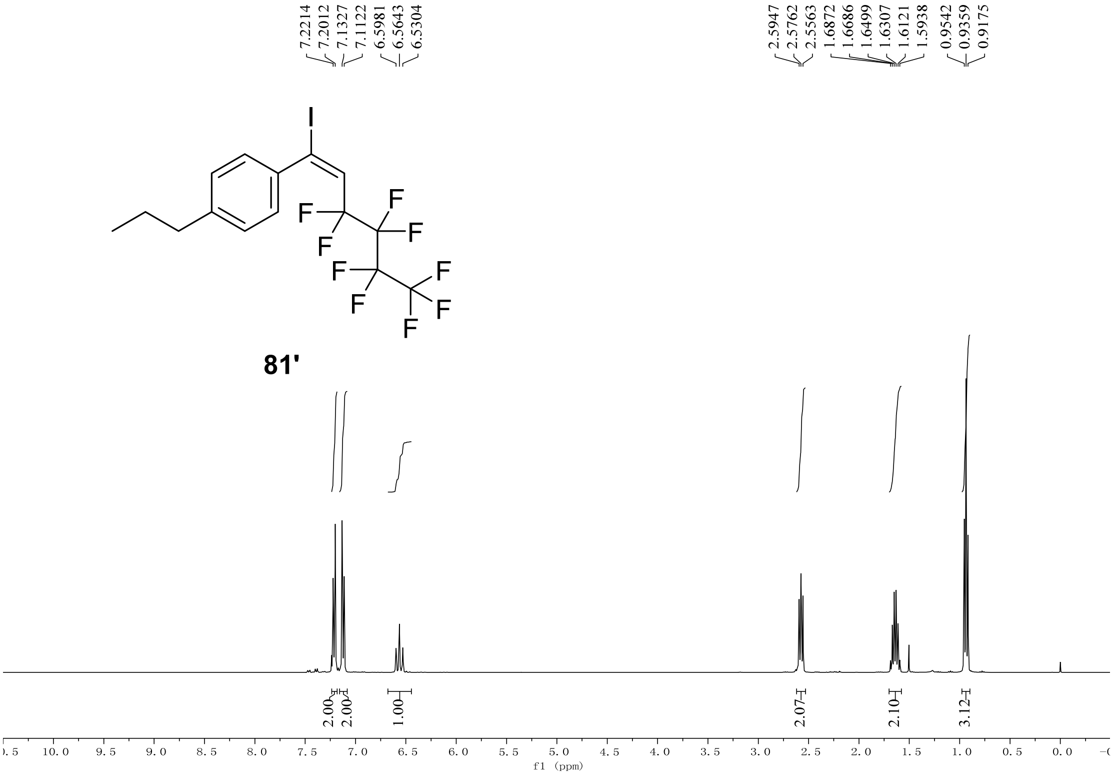

Supplementary Figure 203. ^{1}H NMR spectrum for compound **81’**

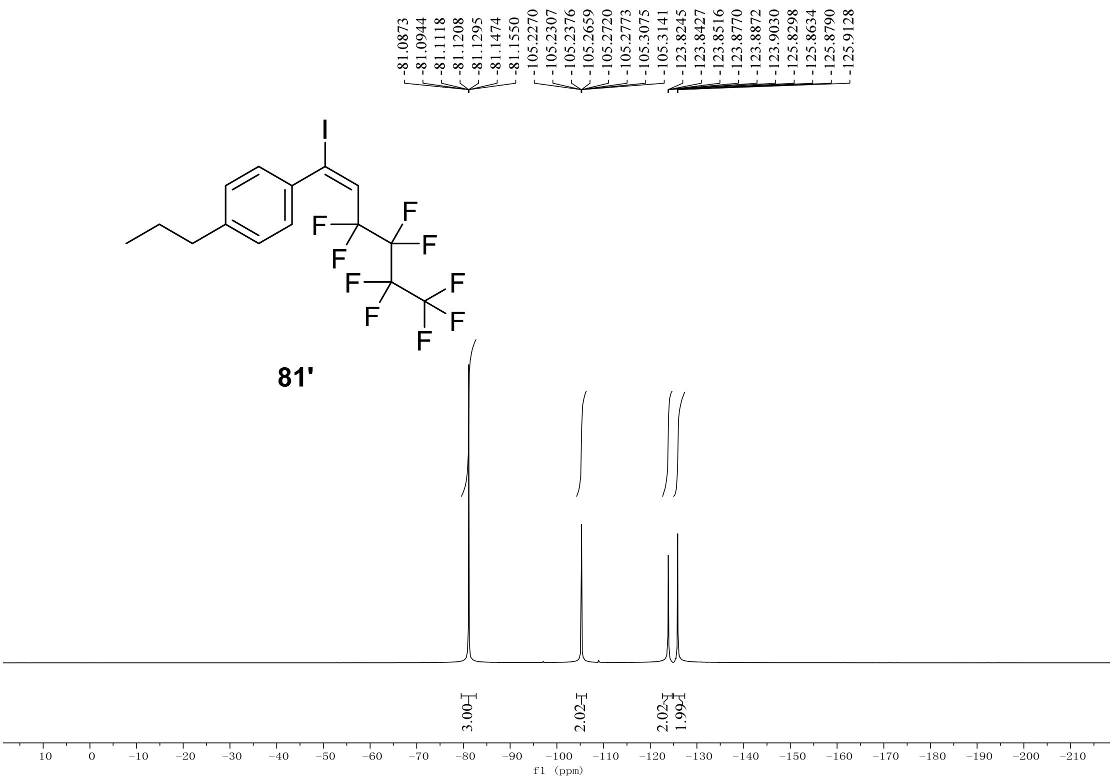

Supplementary Figure 204. ^{19}F NMR spectrum for compound **81’**

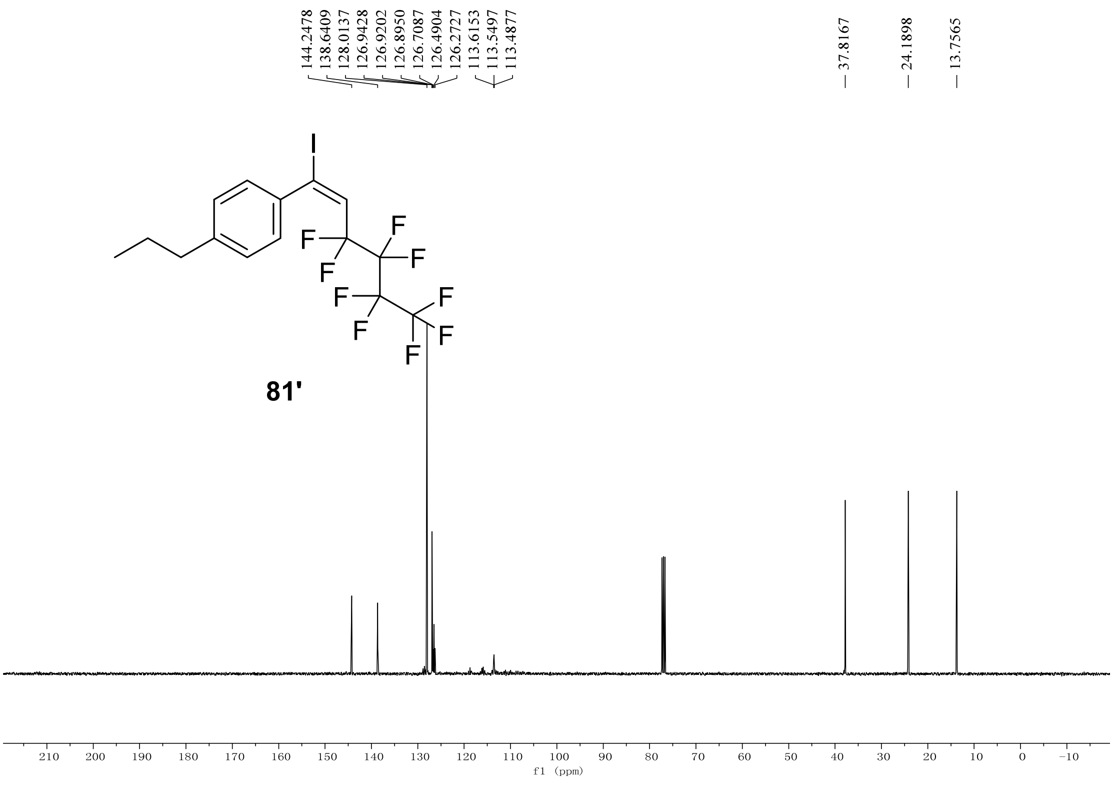

Supplementary Figure 205. ^{13}C NMR spectrum for compound **81’**

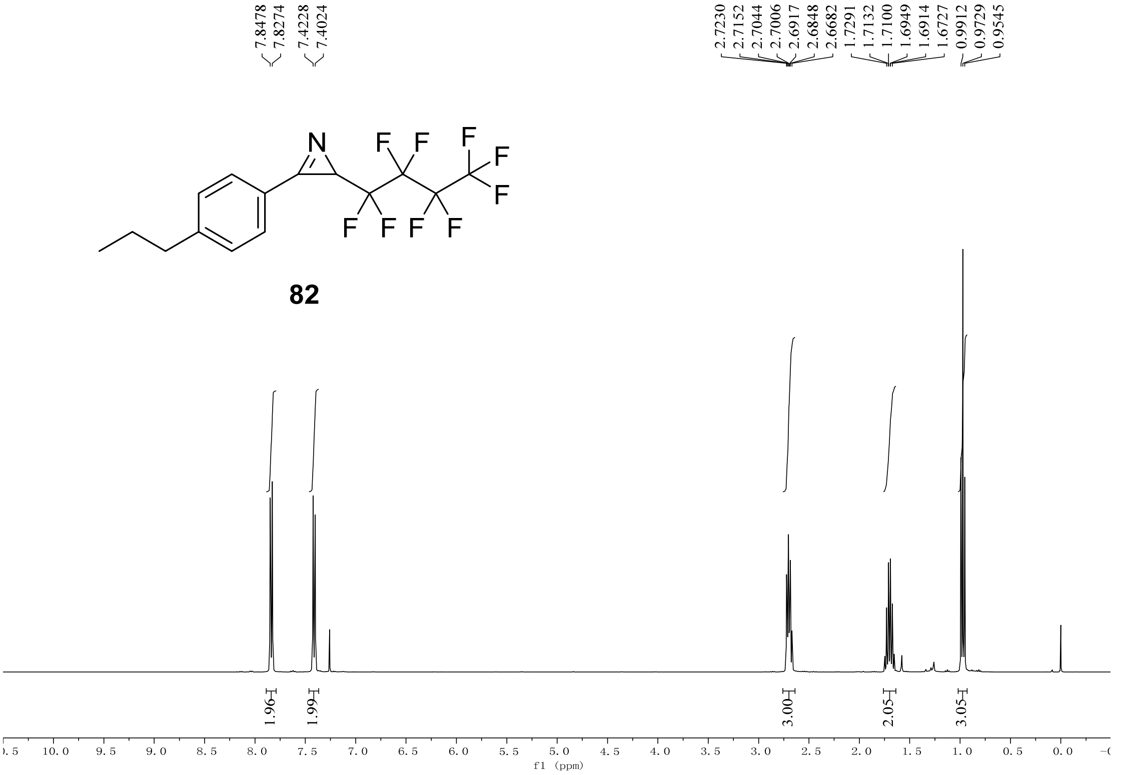

Supplementary Figure 206. ^{1}H NMR spectrum for compound **82**

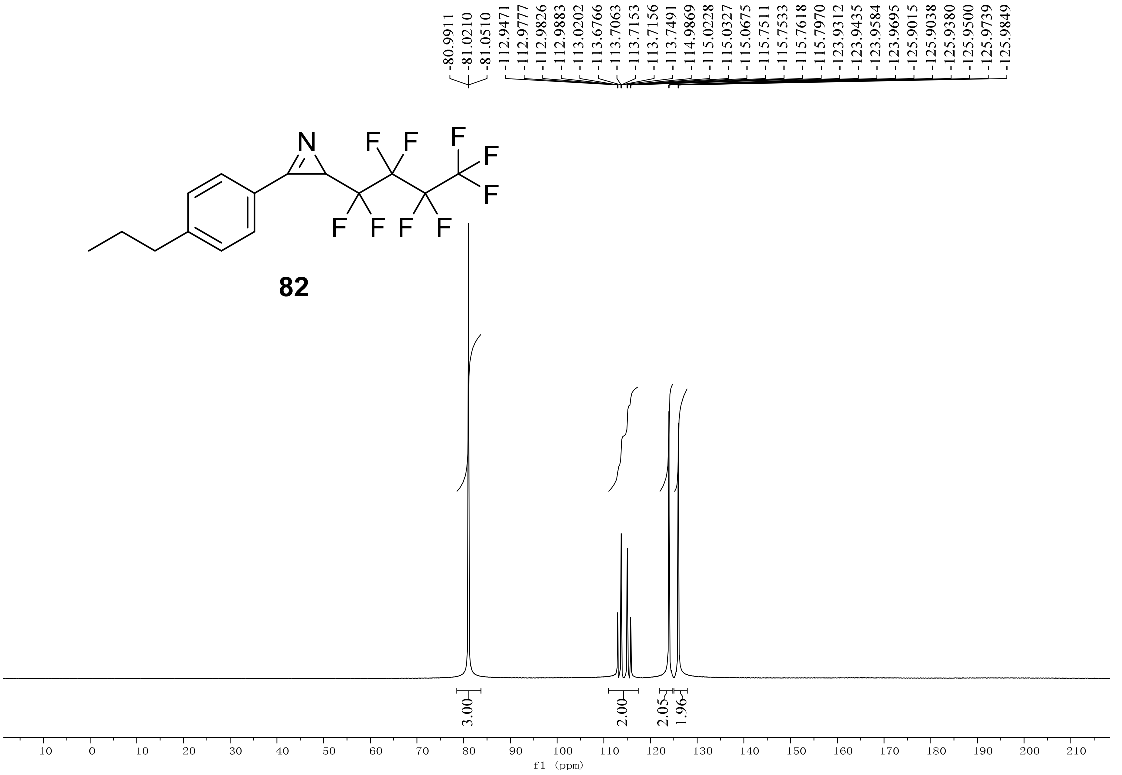

Supplementary Figure 207. ^{19}F NMR spectrum for compound **82**

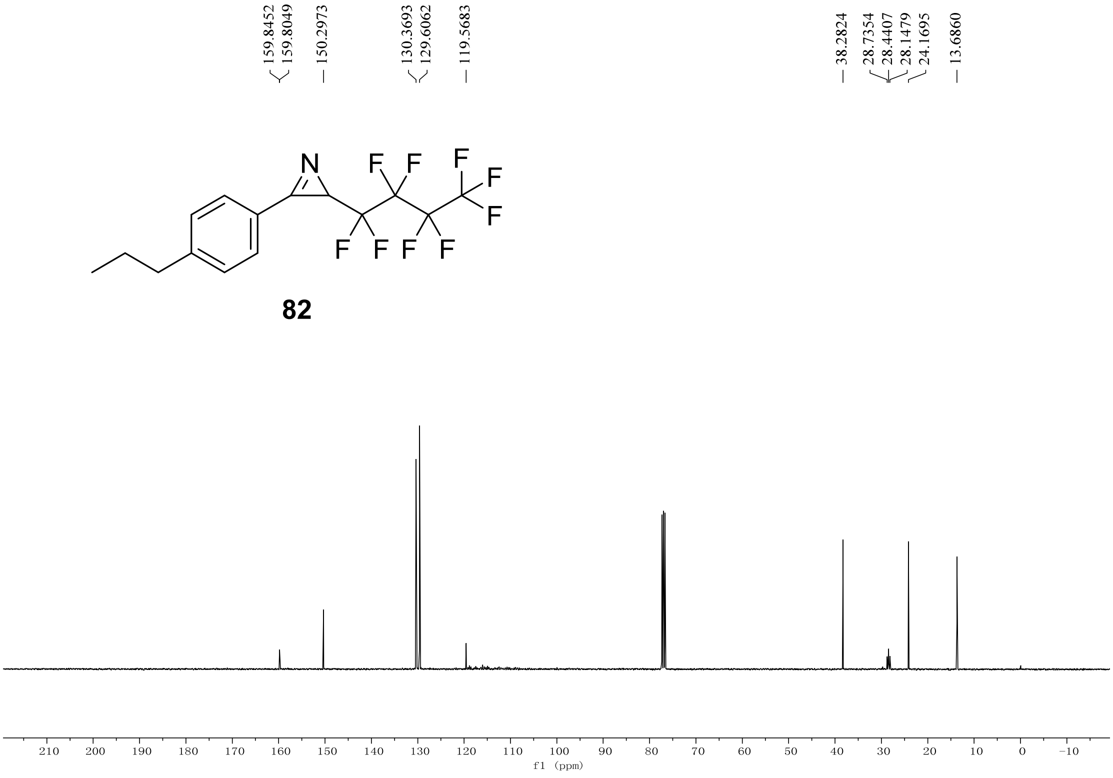

Supplementary Figure 208. ^{13}C NMR spectrum for compound **82**

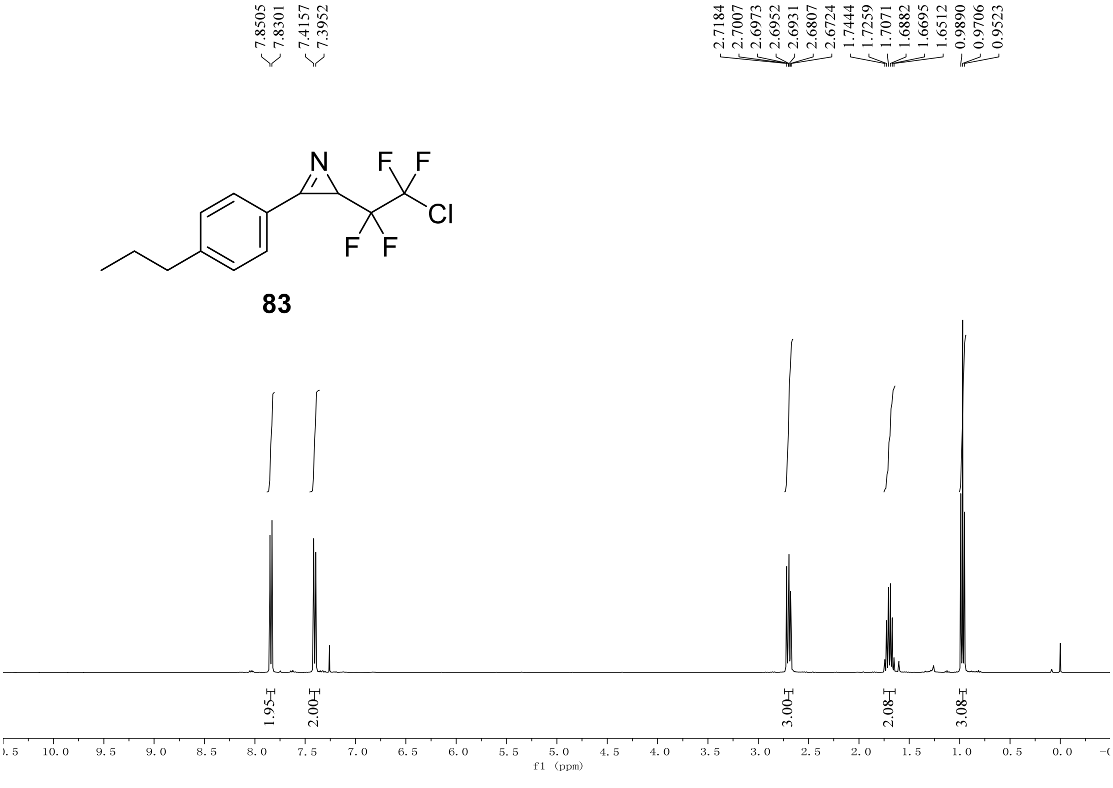

Supplementary Figure 209. ^{1}H NMR spectrum for compound **83**

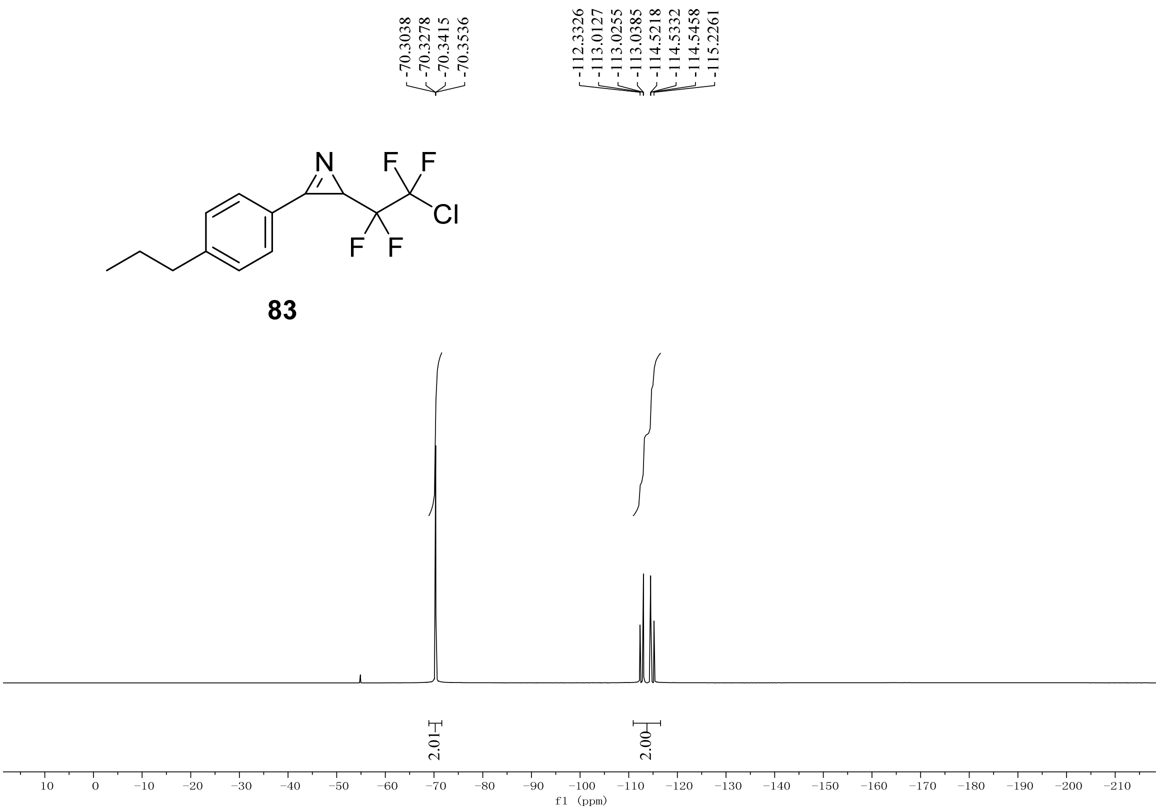

Supplementary Figure 210. ^{19}F NMR spectrum for compound **83**

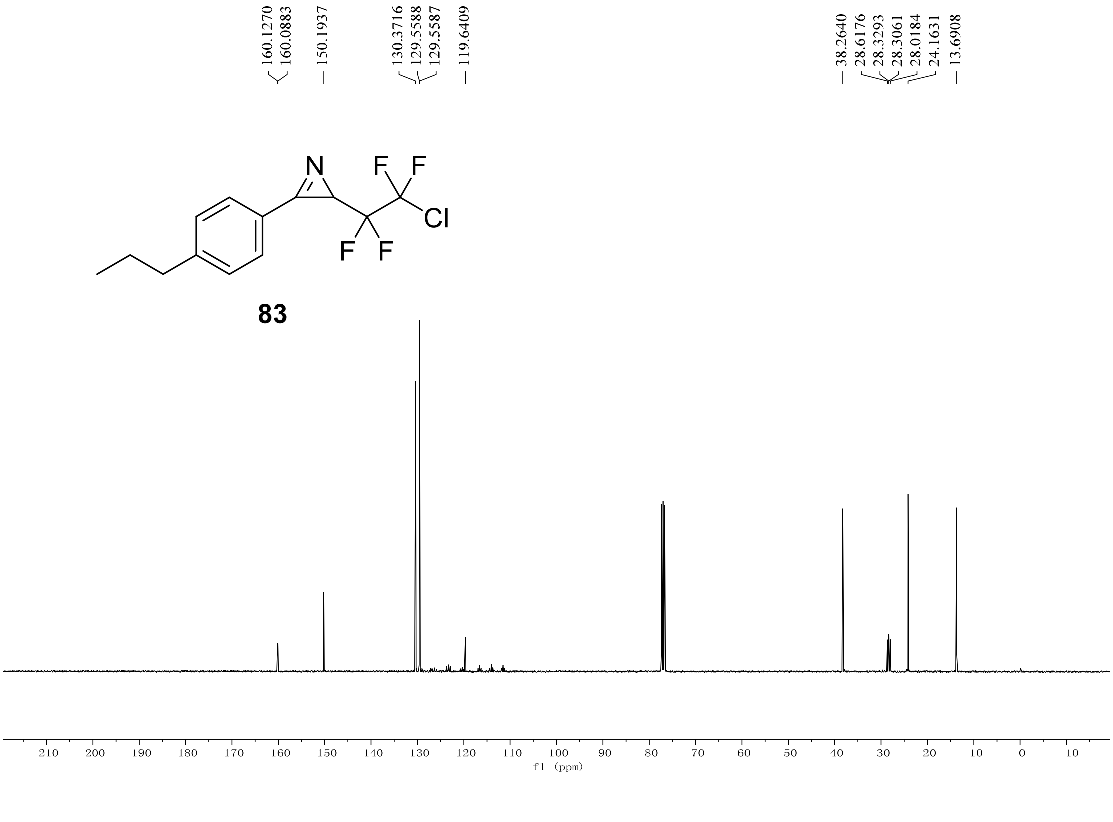

Supplementary Figure 211. ^{13}C NMR spectrum for compound **83**

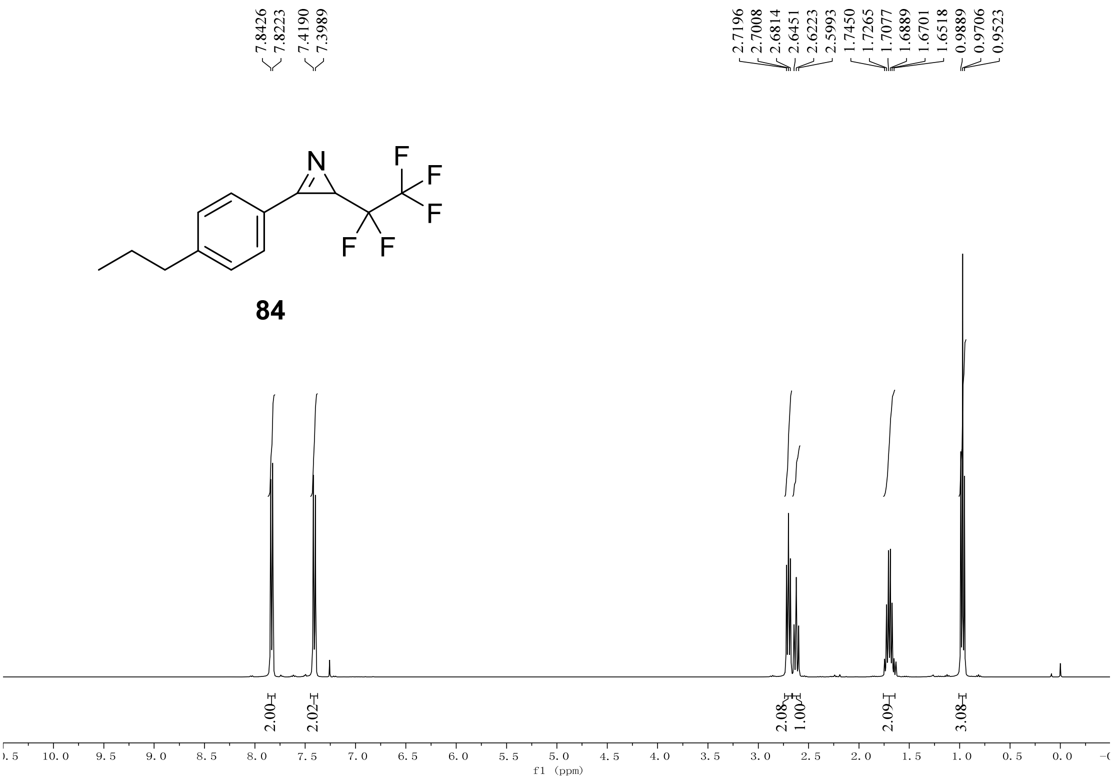

Supplementary Figure 212. ^{1}H NMR spectrum for compound **84**

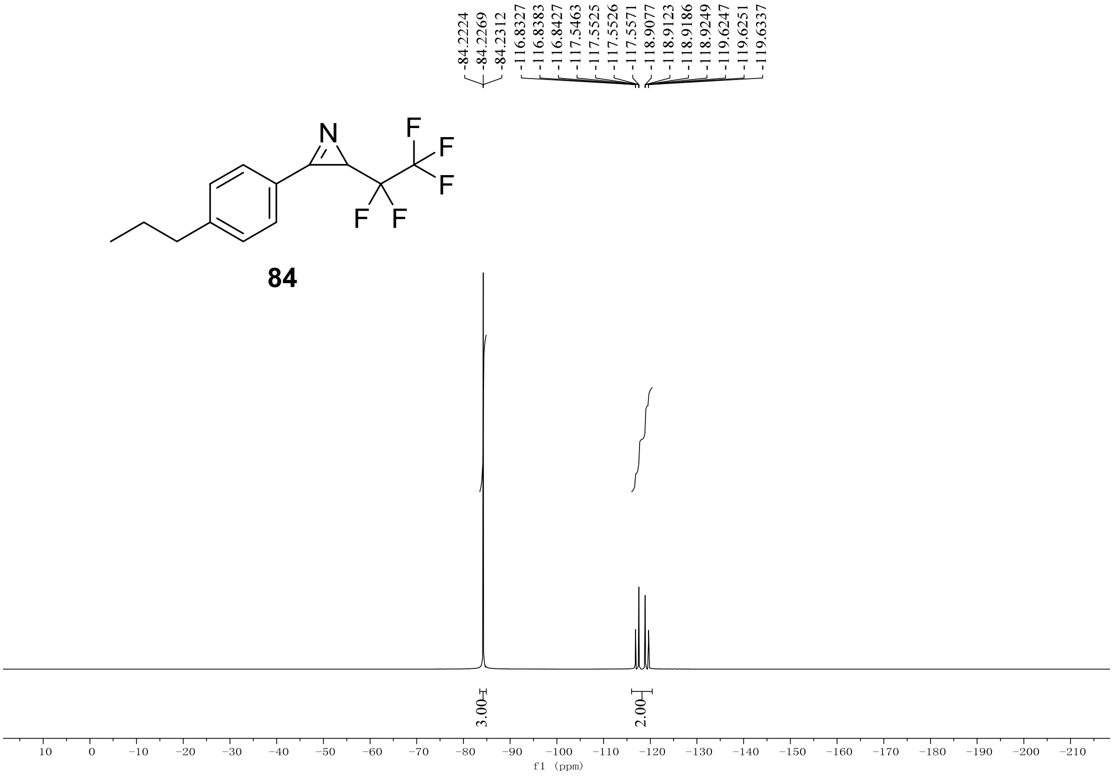

Supplementary Figure 213. ^{19}F NMR spectrum for compound **84**

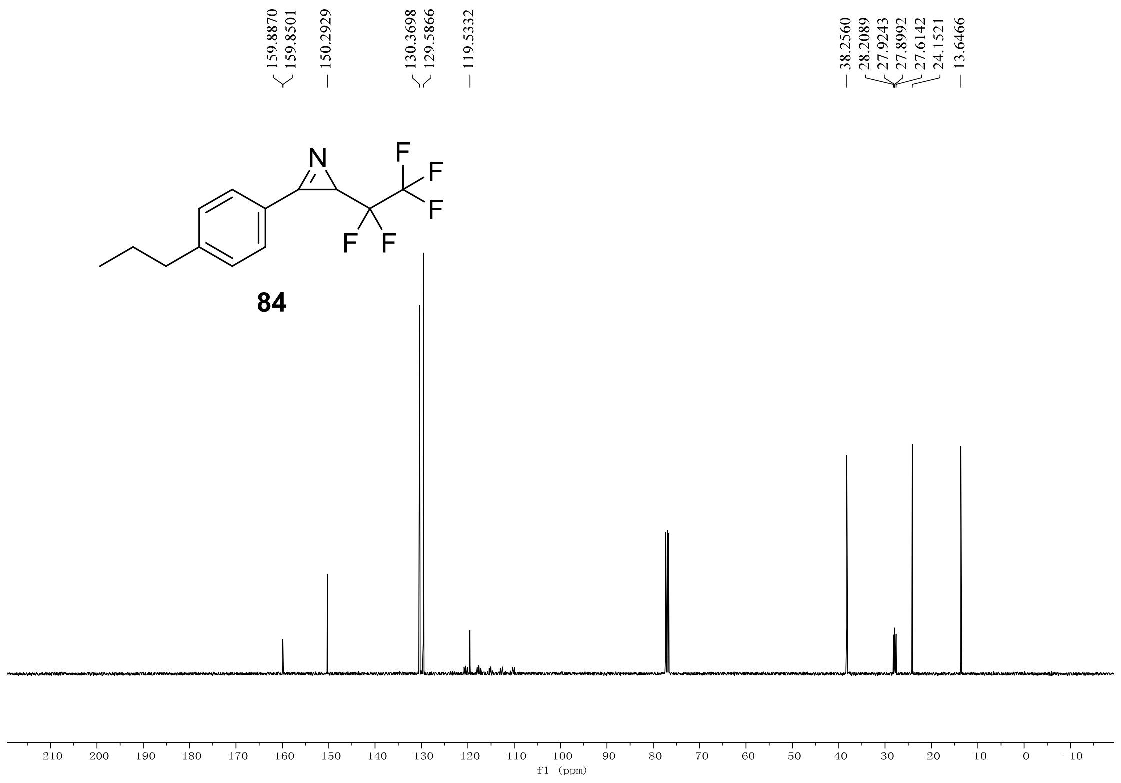

Supplementary Figure 214. ^{13}C NMR spectrum for compound **84**

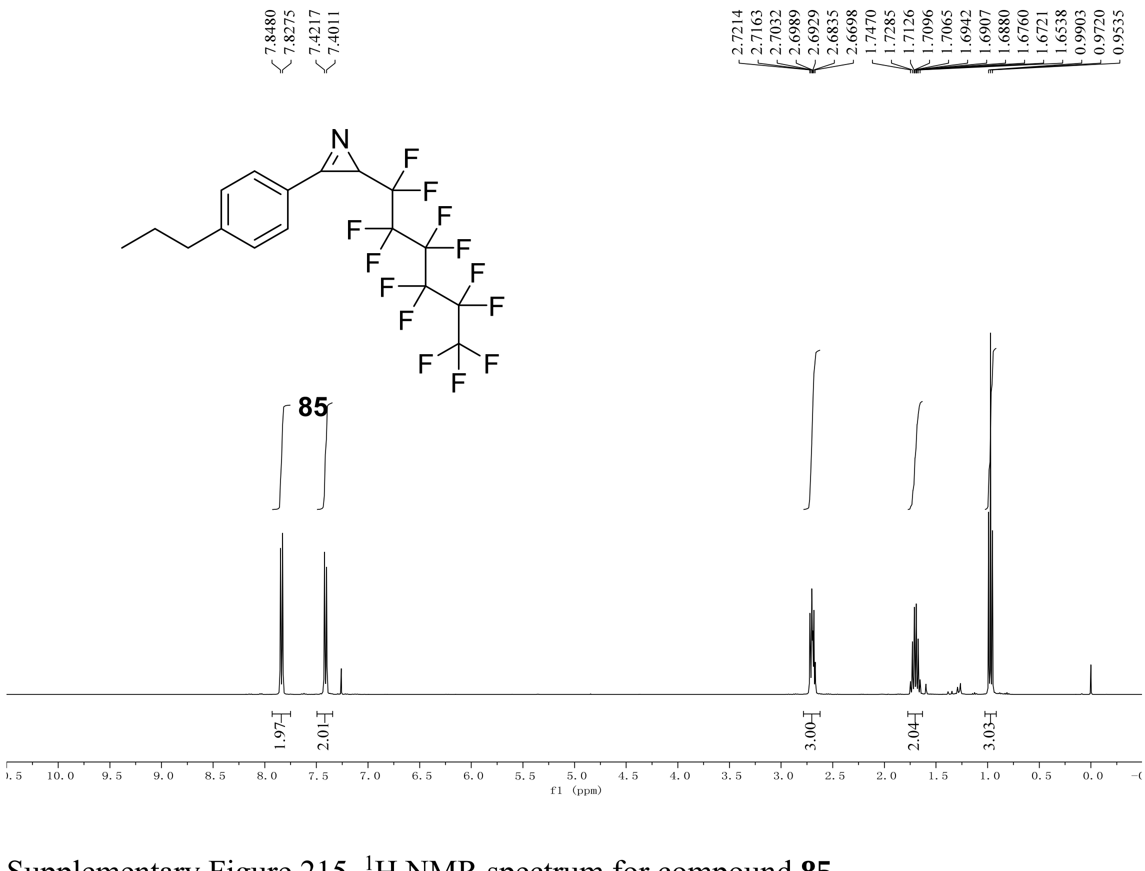

Supplementary Figure 215. ^{1}H NMR spectrum for compound **85**

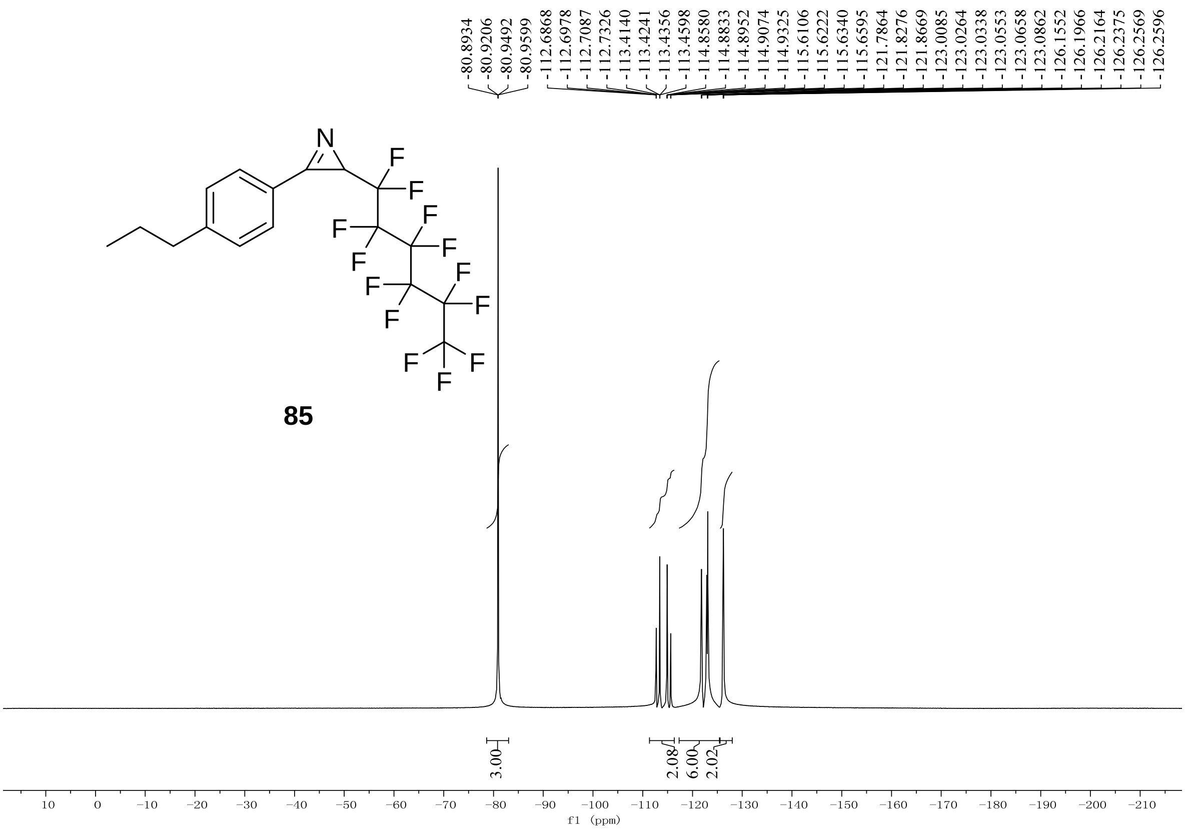

Supplementary Figure 216. ^{19}F NMR spectrum for compound **85**

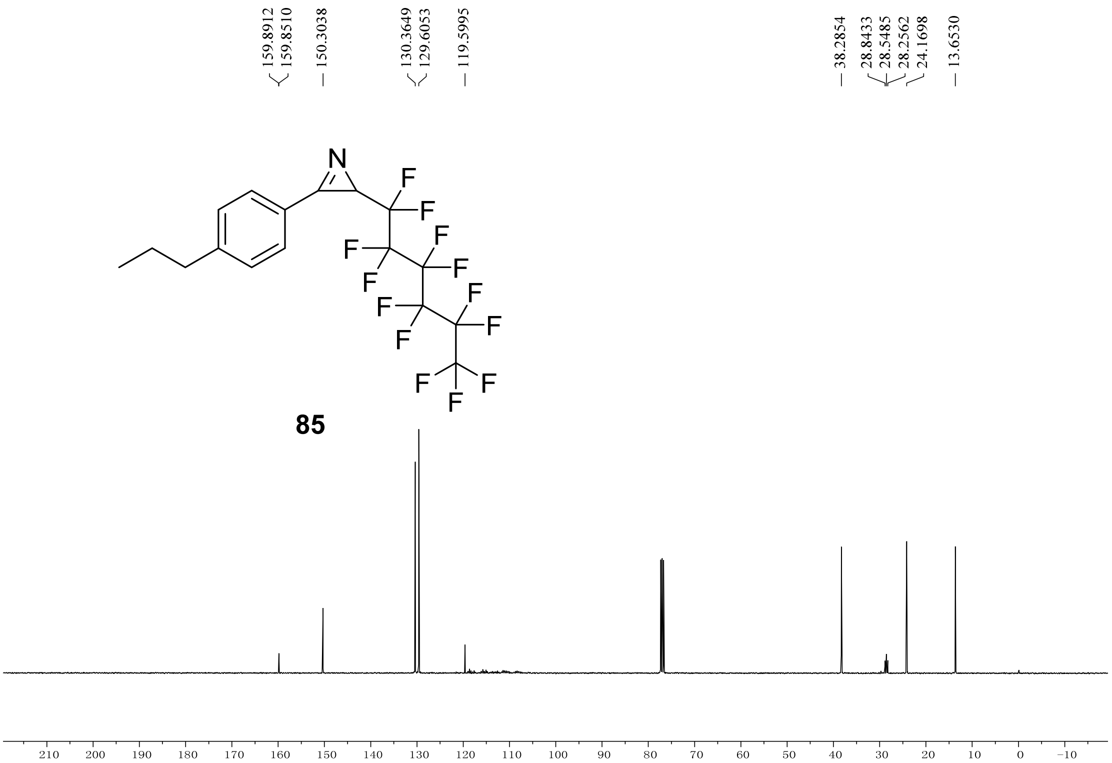

Supplementary Figure 217. ^{13}C NMR spectrum for compound **85**

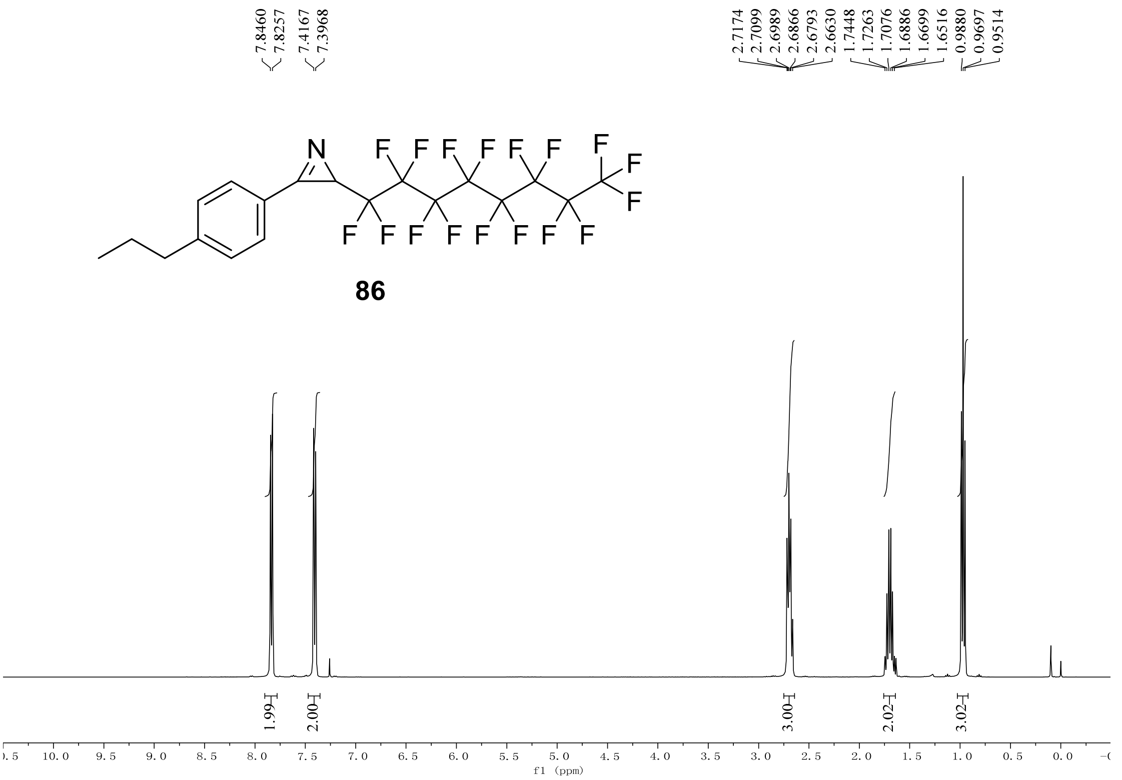

Supplementary Figure 218. ^{1}H NMR spectrum for compound **86**

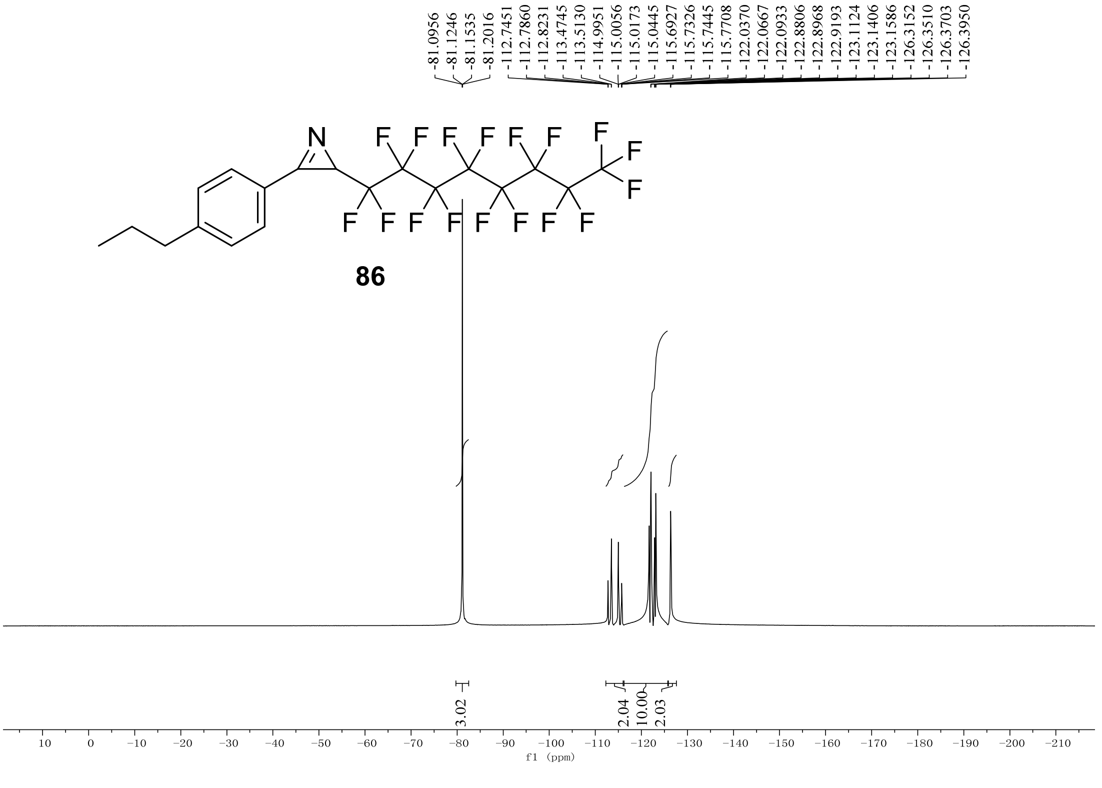

Supplementary Figure 219. ^{19}F NMR spectrum for compound **86**

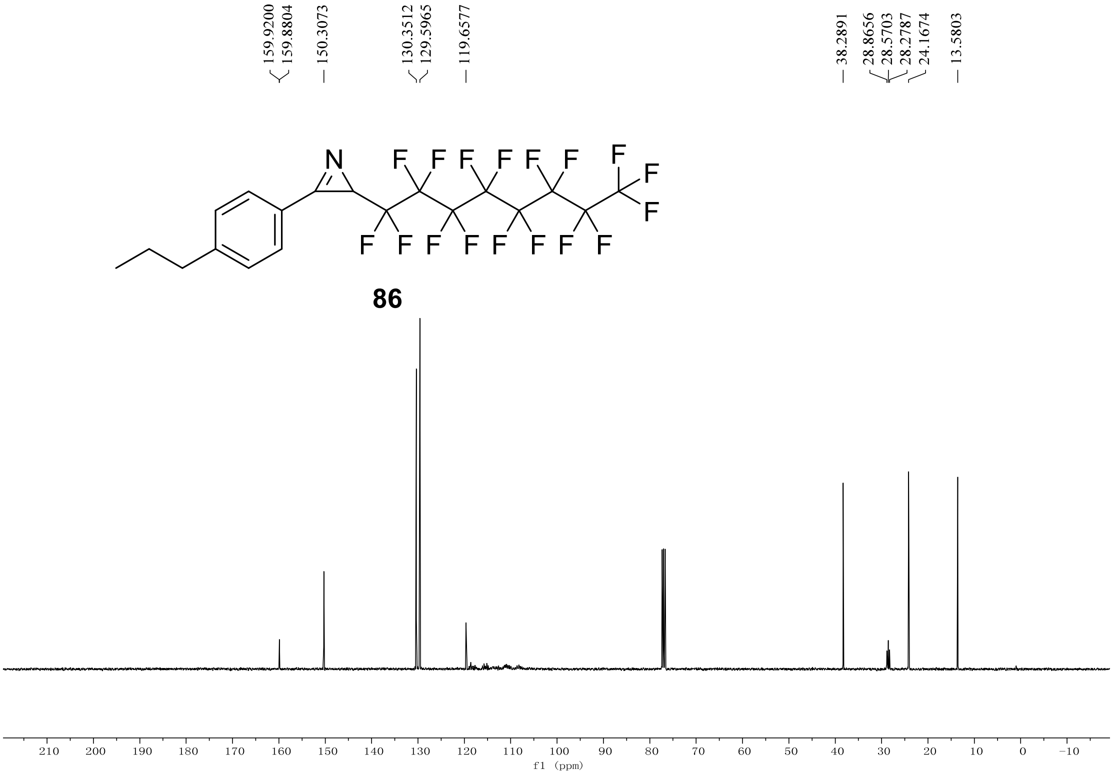

Supplementary Figure 220. ^{13}C NMR spectrum for compound **86**

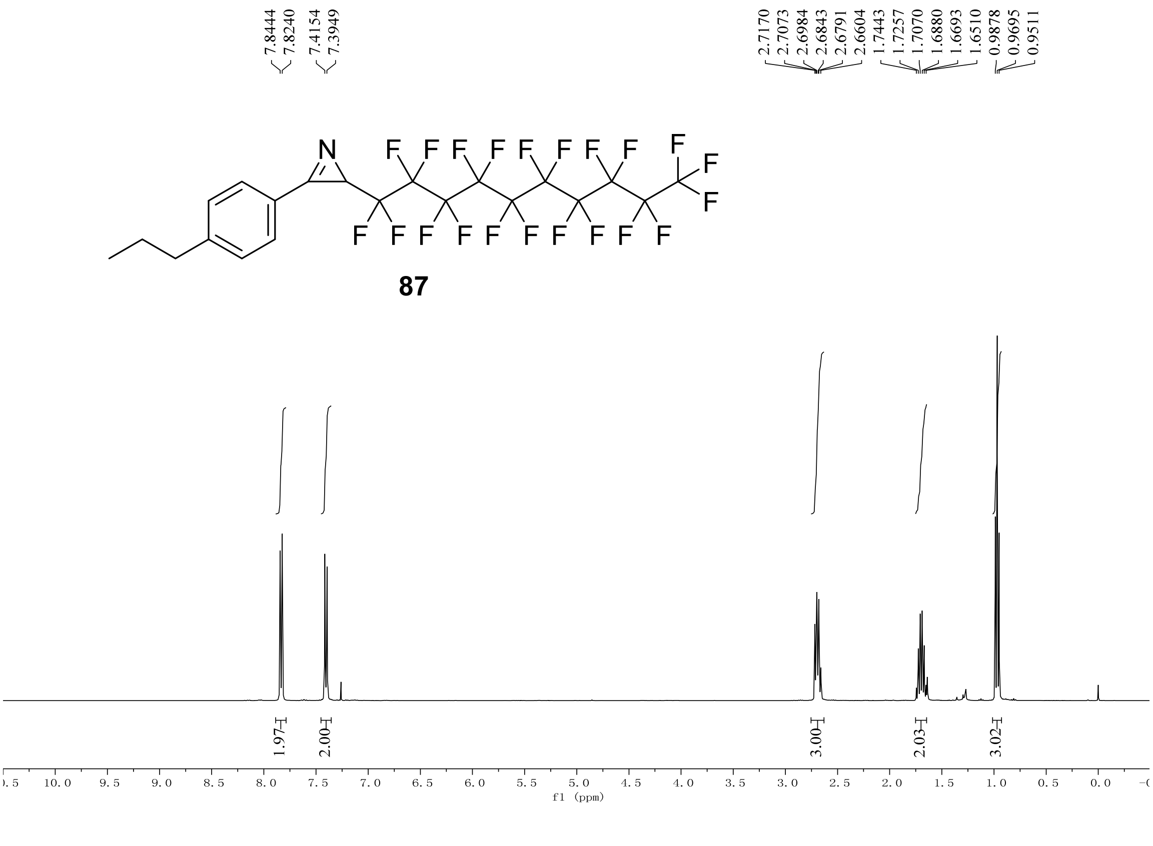

Supplementary Figure 221. ^{1}H NMR spectrum for compound **87**

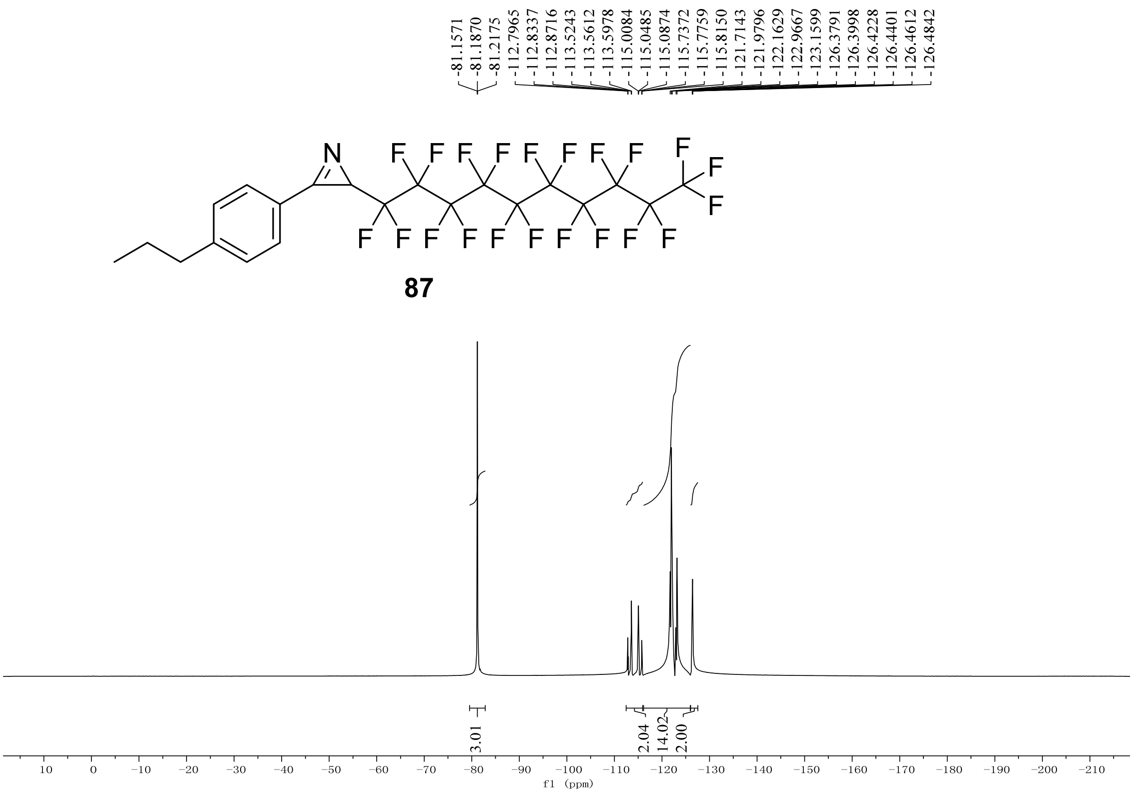

Supplementary Figure 222. ^{19}F NMR spectrum for compound **87**

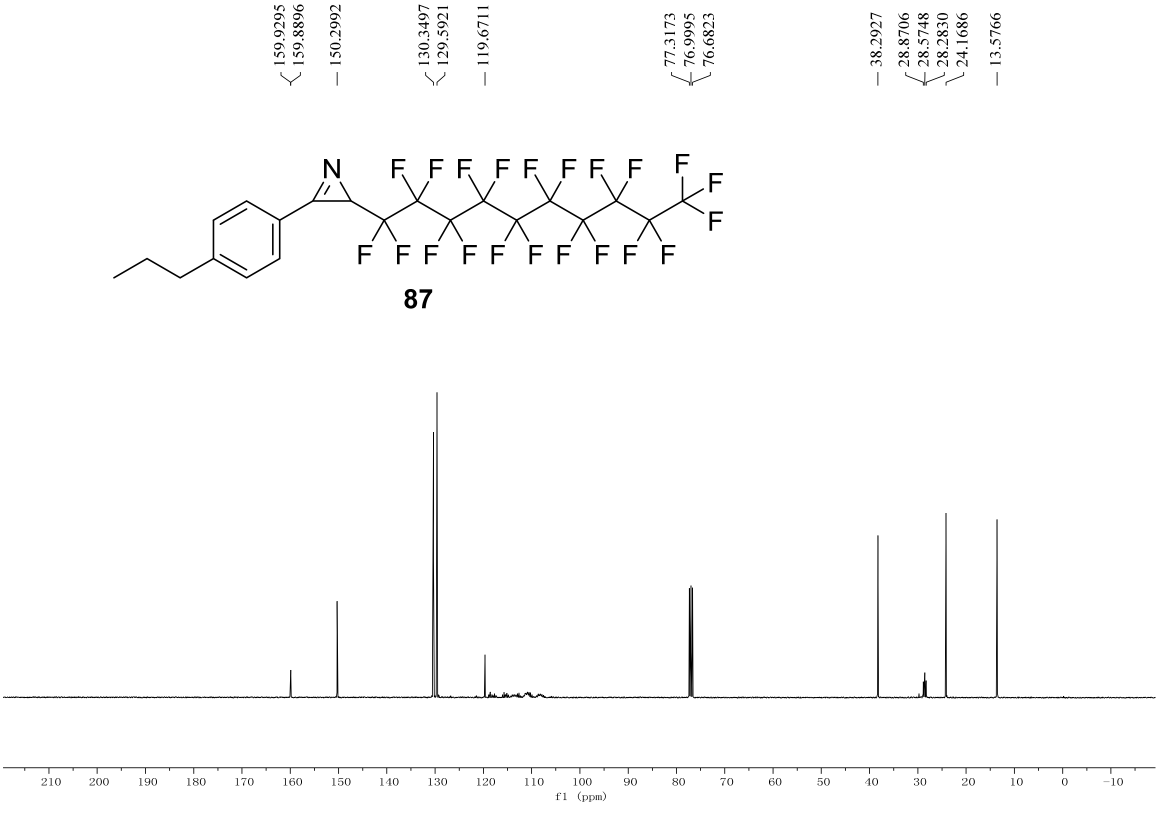

Supplementary Figure 223. ^{13}C NMR spectrum for compound **87**

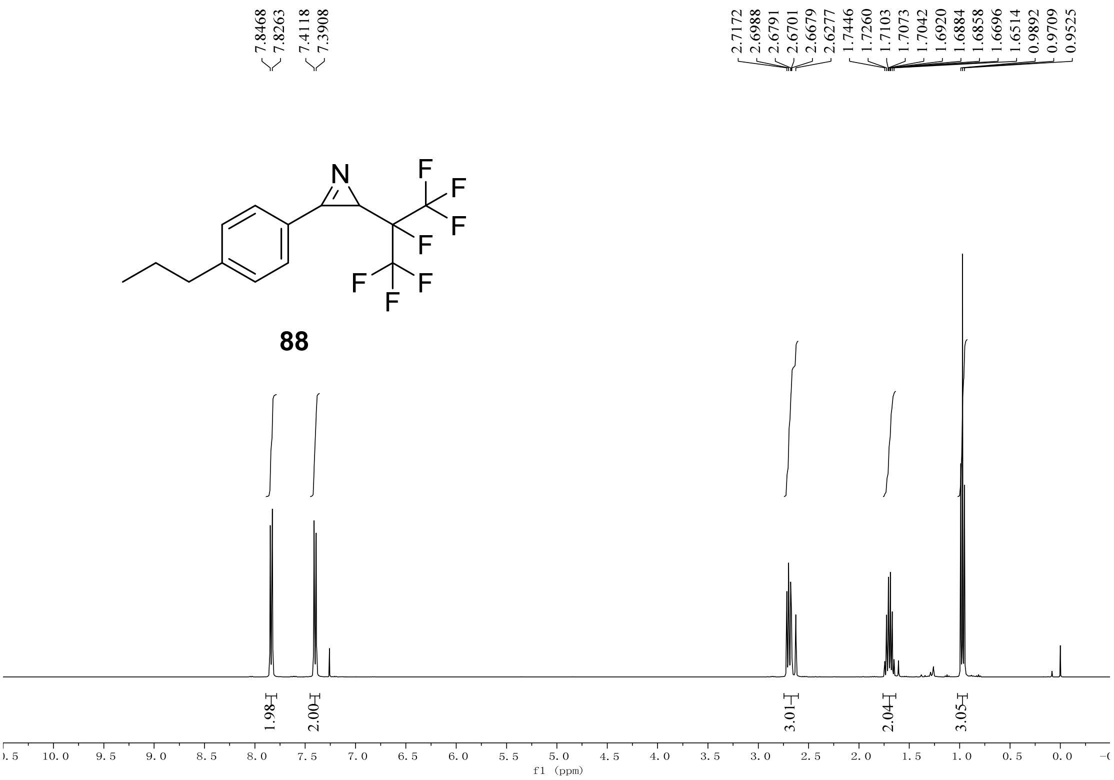

Supplementary Figure 224. ^{1}H NMR spectrum for compound **88**

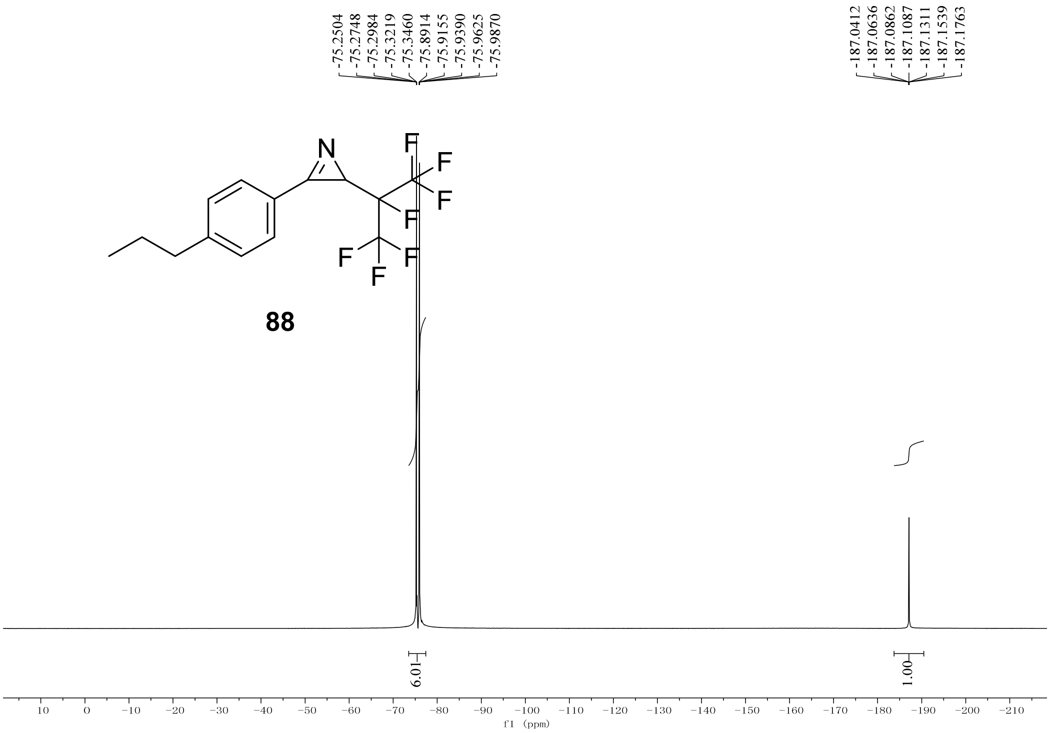

Supplementary Figure 225. ^{19}F NMR spectrum for compound **88**

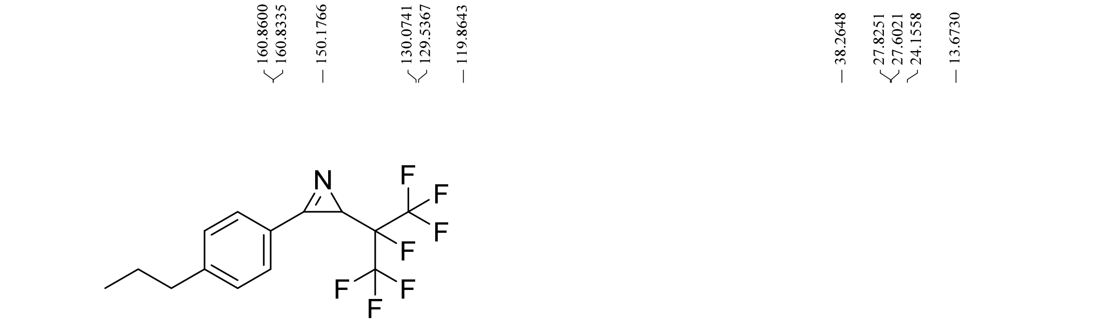

88

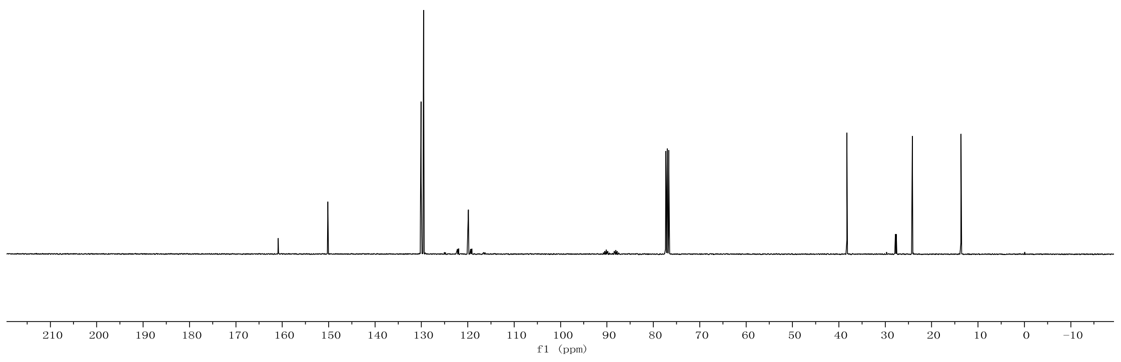

Supplementary Figure 226. ^{13}C NMR spectrum for compound **88**

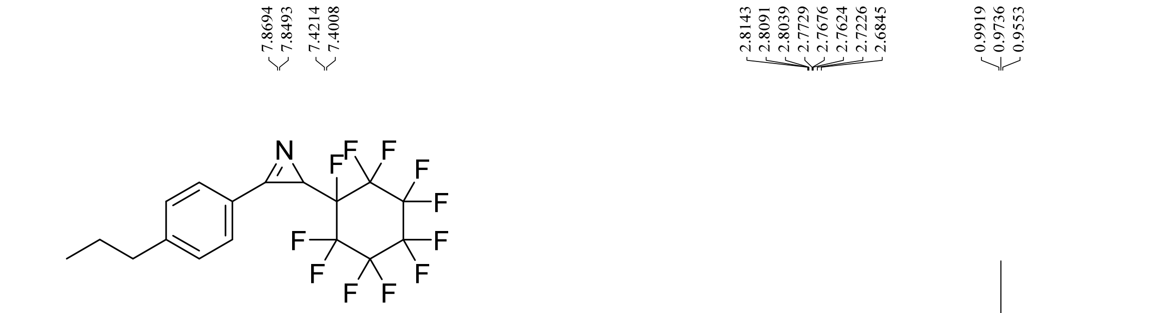

89

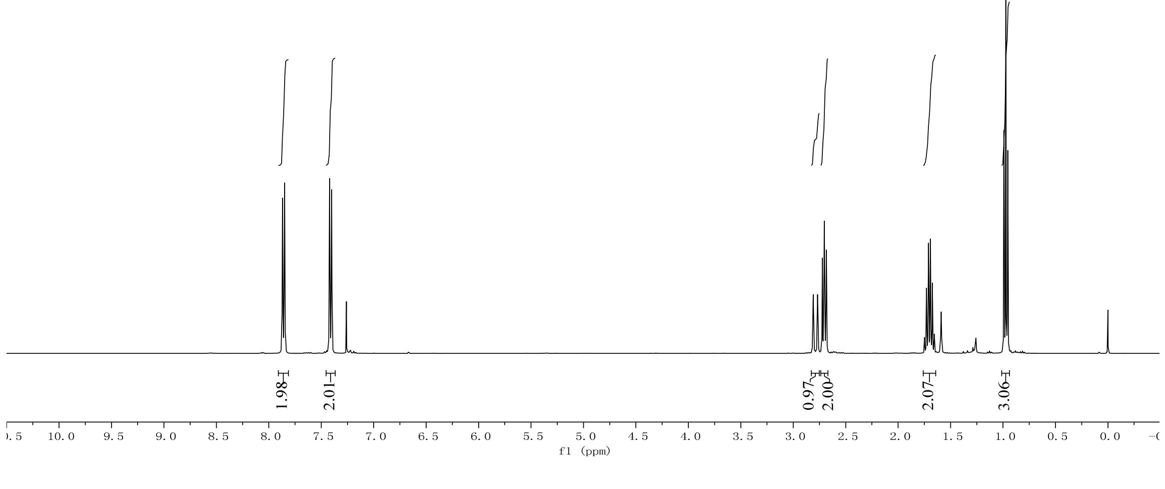

Supplementary Figure 227. ^{1}H NMR spectrum for compound **89**

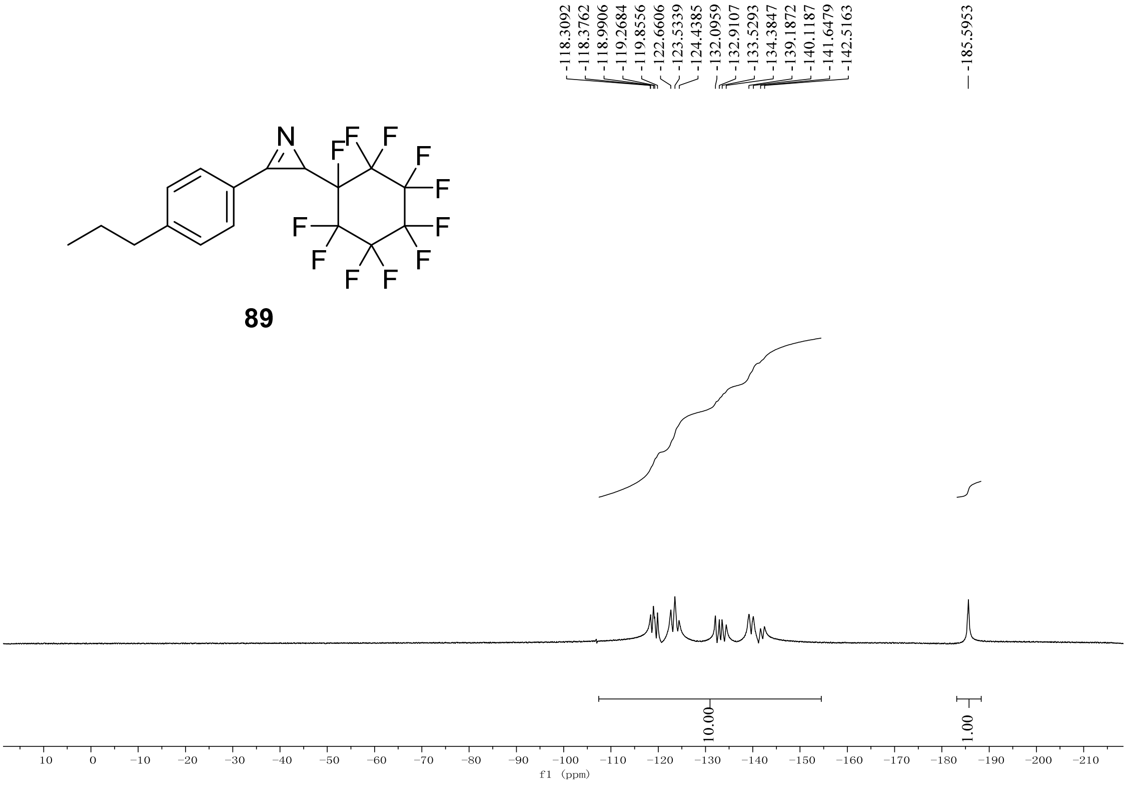

Supplementary Figure 228. ^{19}F NMR spectrum for compound **89**

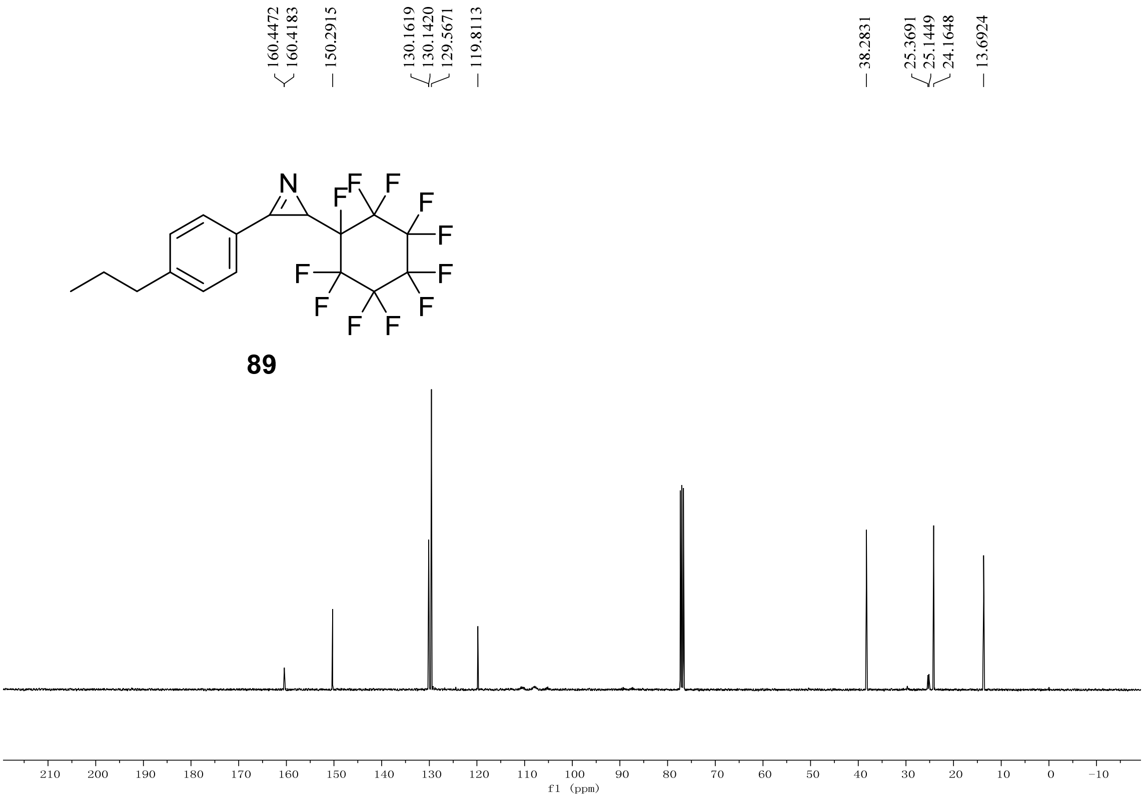

Supplementary Figure 229. ^{13}C NMR spectrum for compound **89**

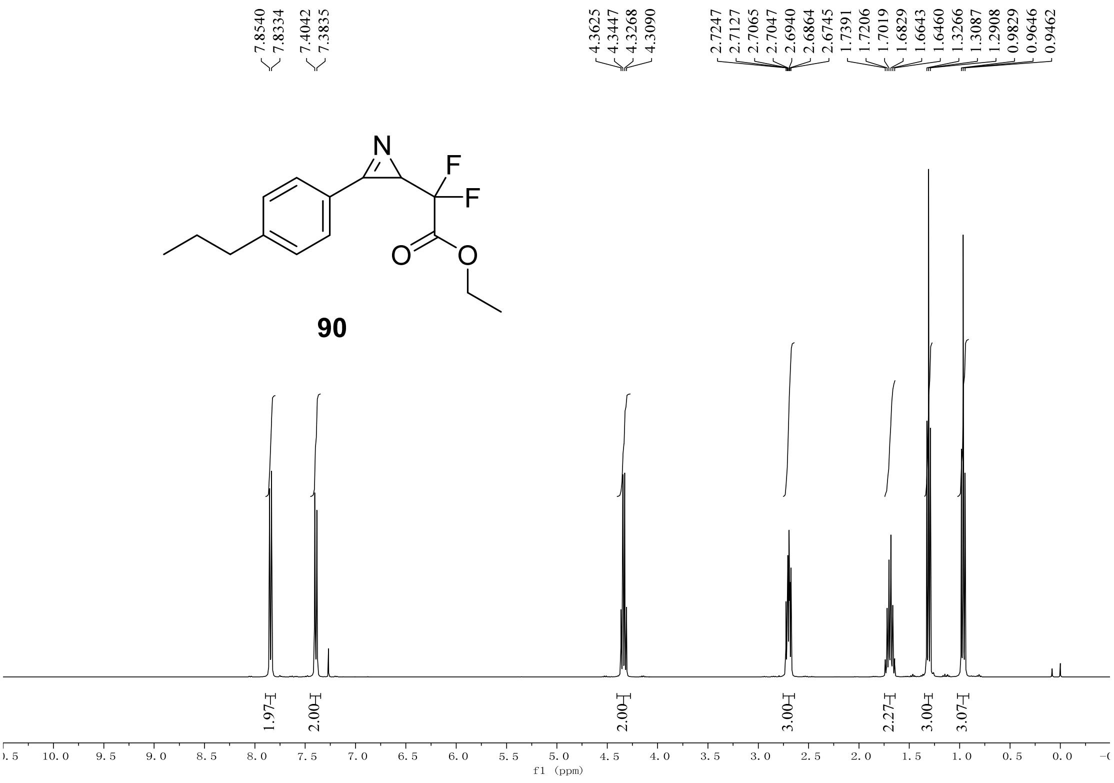

Supplementary Figure 230. ^{1}H NMR spectrum for compound **90**

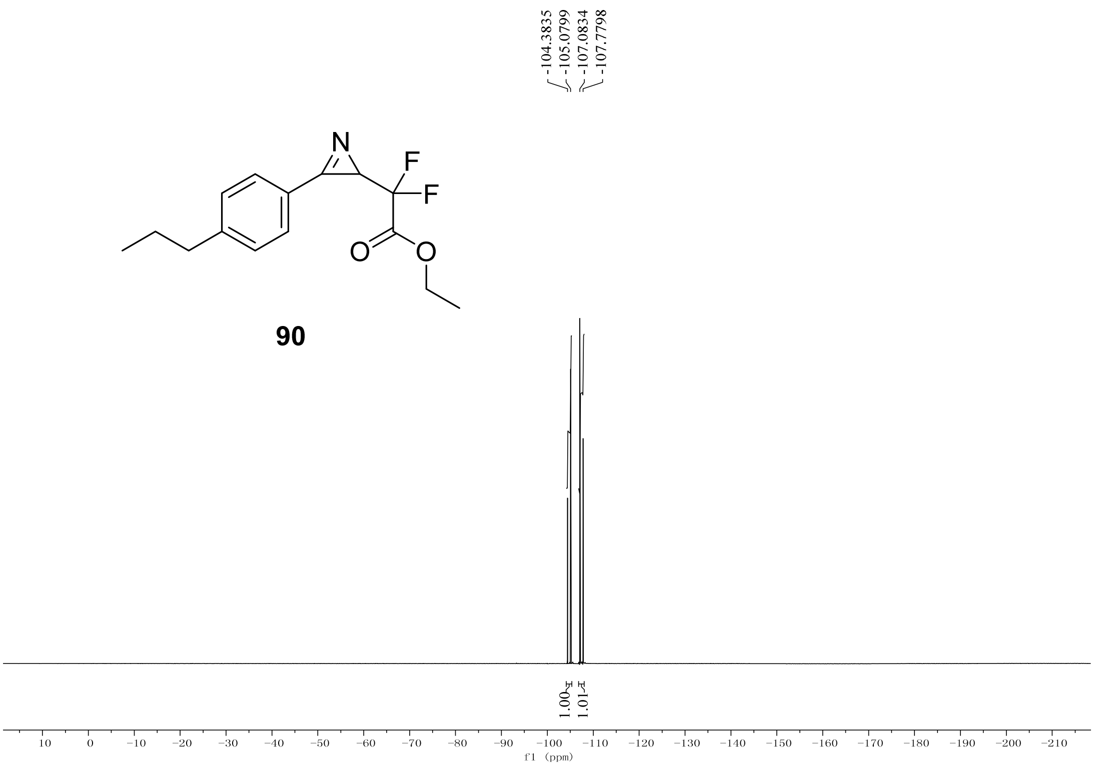

Supplementary Figure 231. ^{19}F NMR spectrum for compound **90**

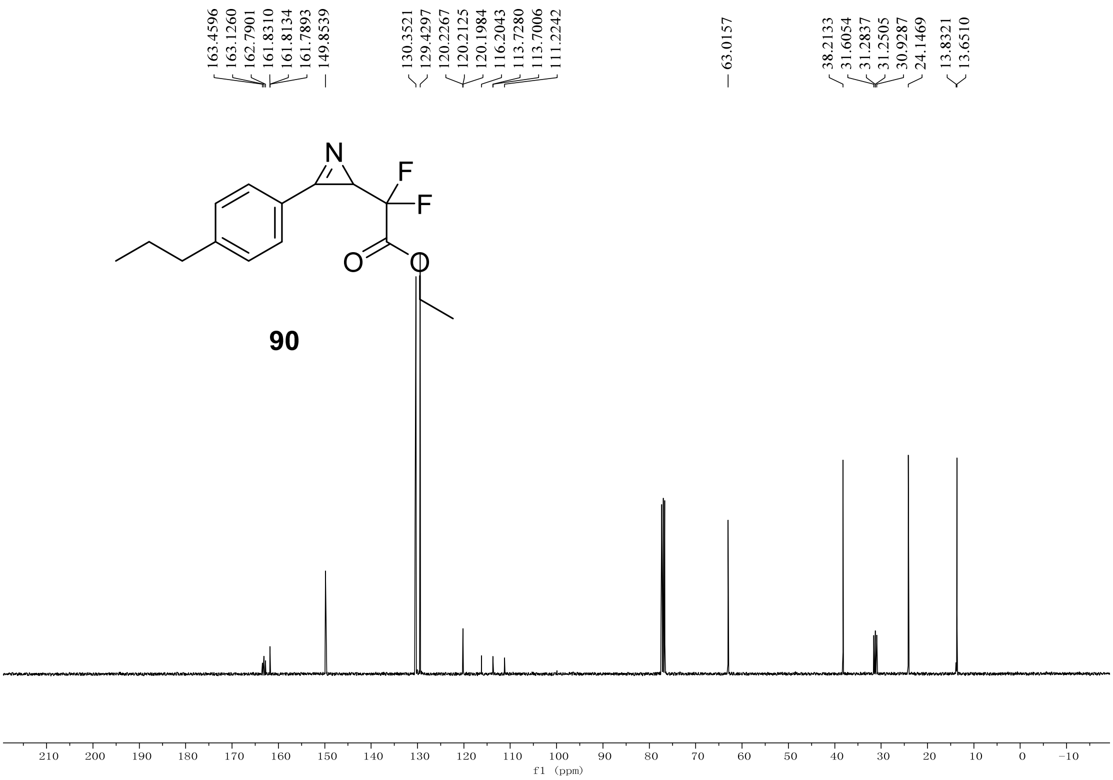

Supplementary Figure 232. ^{13}C NMR spectrum for compound **90**

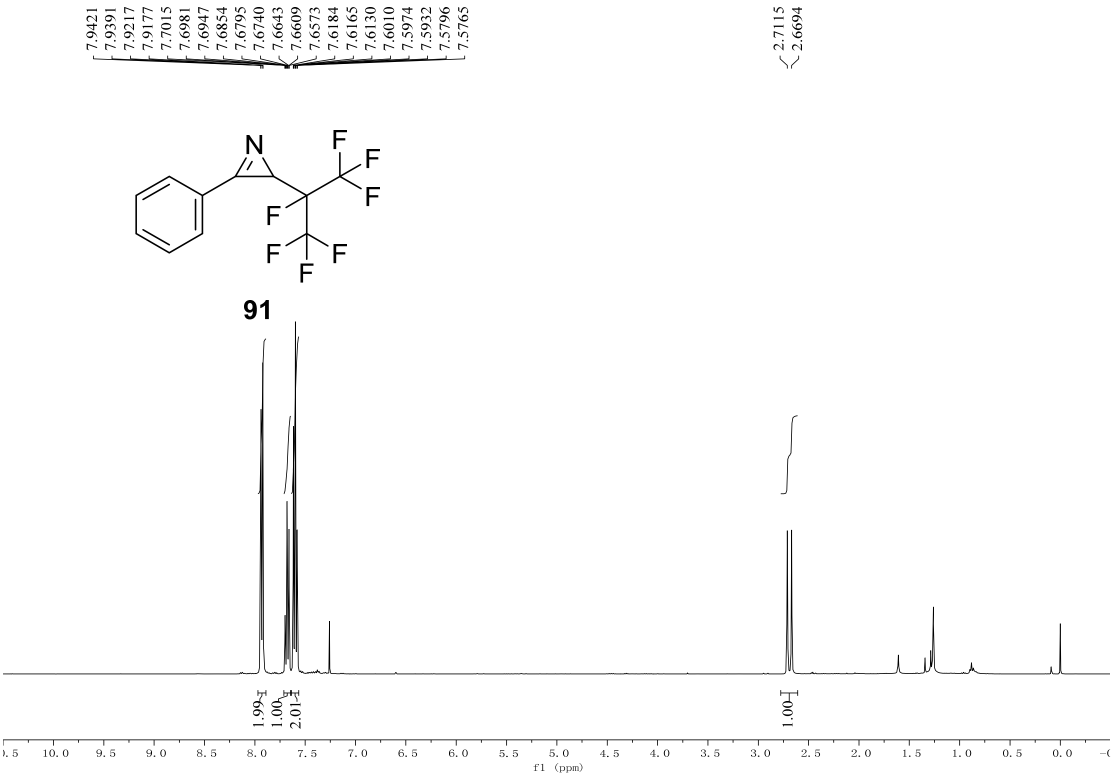

Supplementary Figure 233. ^{1}H NMR spectrum for compound **91**

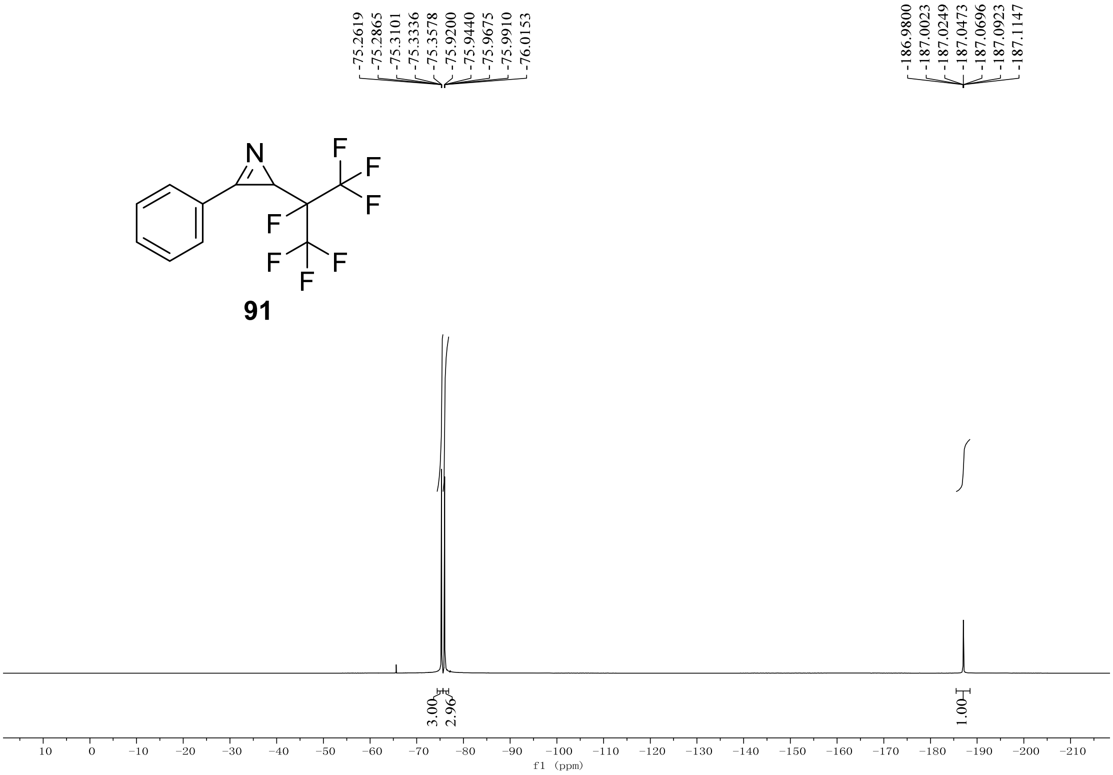

Supplementary Figure 234. ^{19}F NMR spectrum for compound **91**

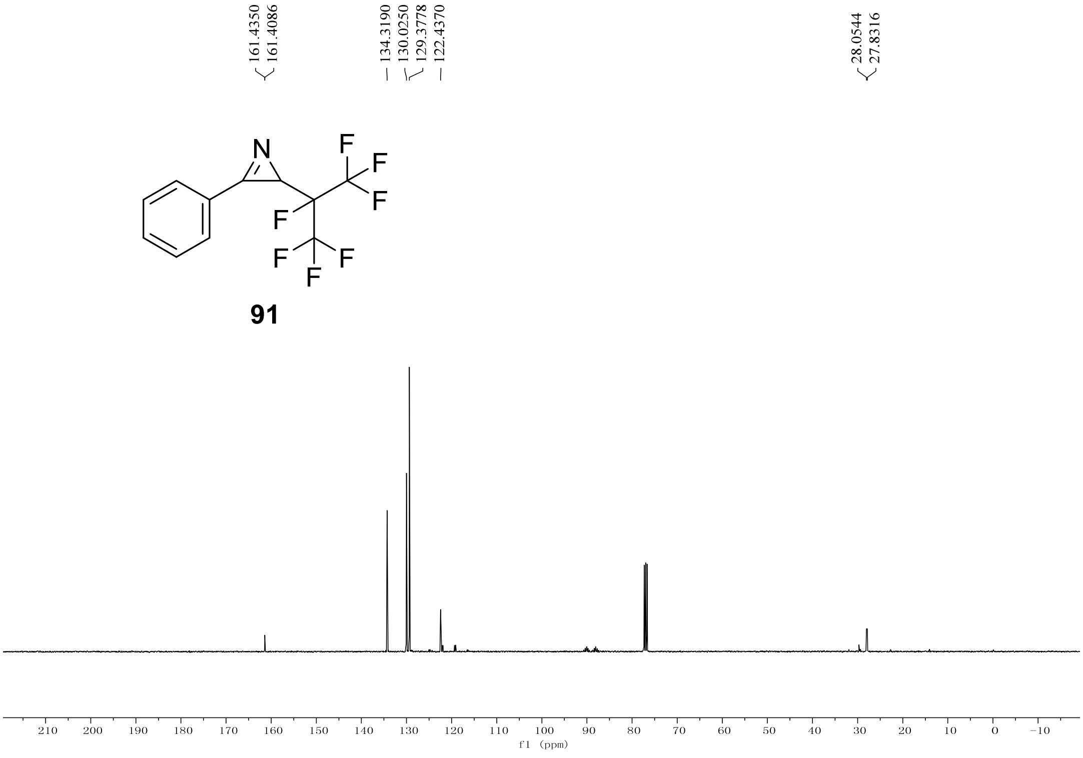

Supplementary Figure 235. ^{13}C NMR spectrum for compound **91**

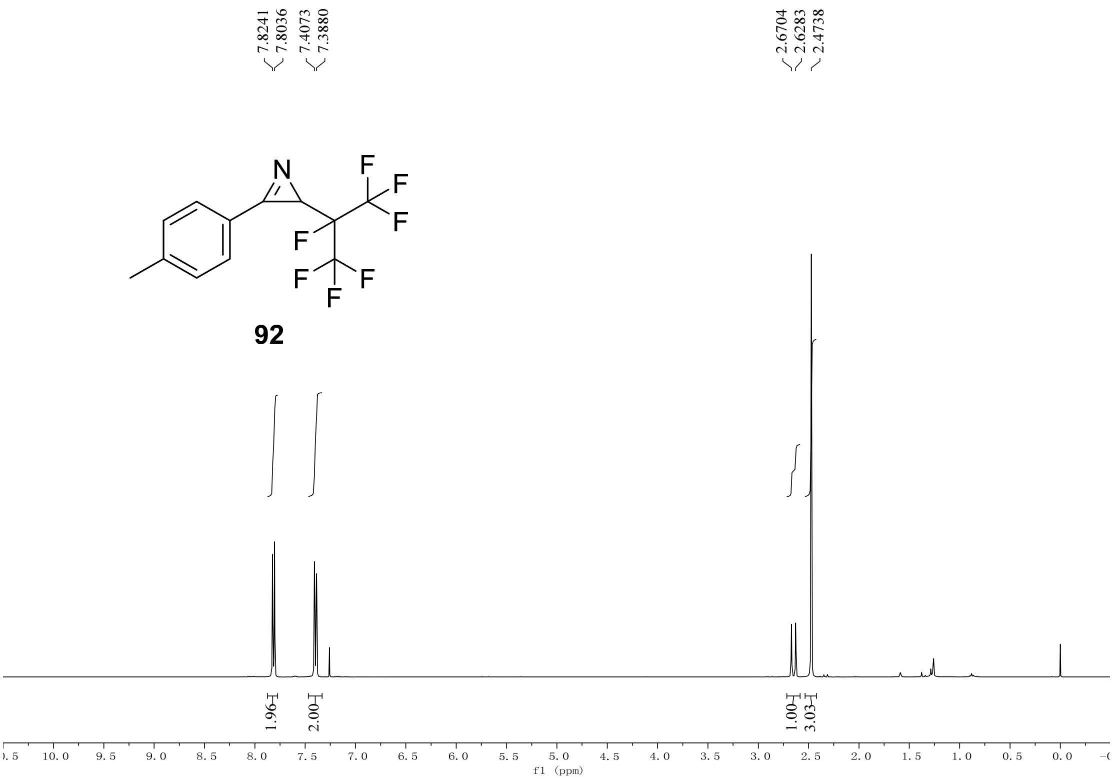

Supplementary Figure 236. ^{1}H NMR spectrum for compound **92**

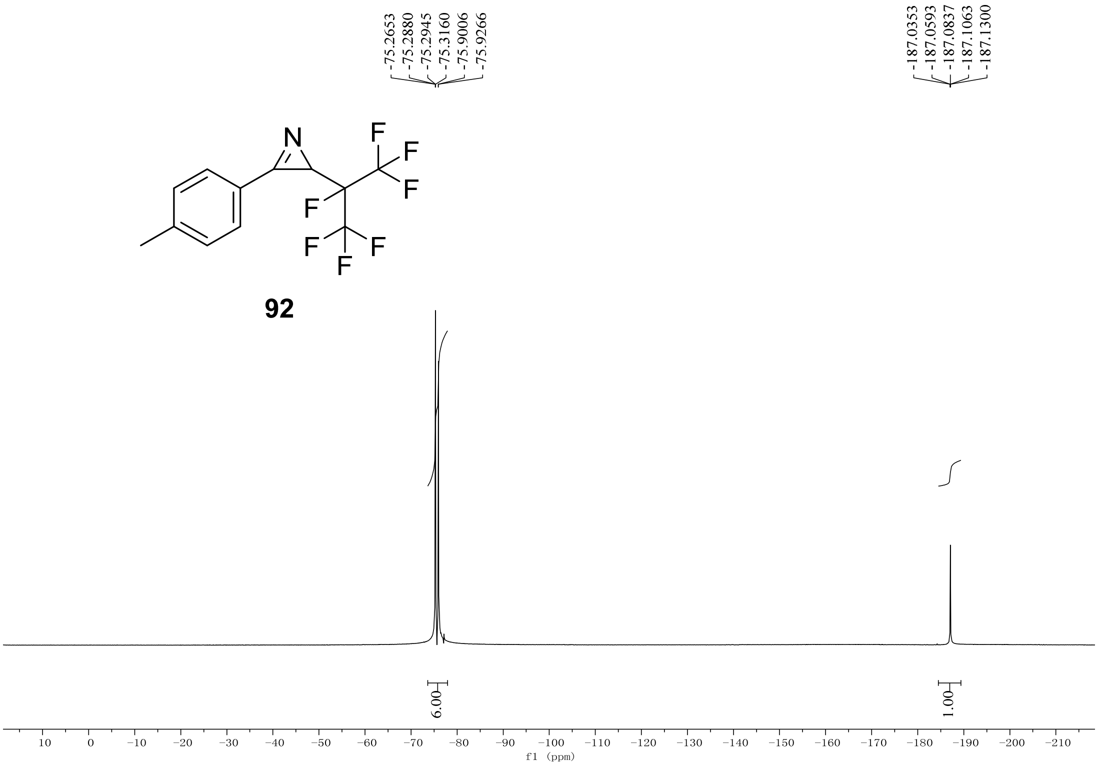

Supplementary Figure 237. ^{19}F NMR spectrum for compound **92**

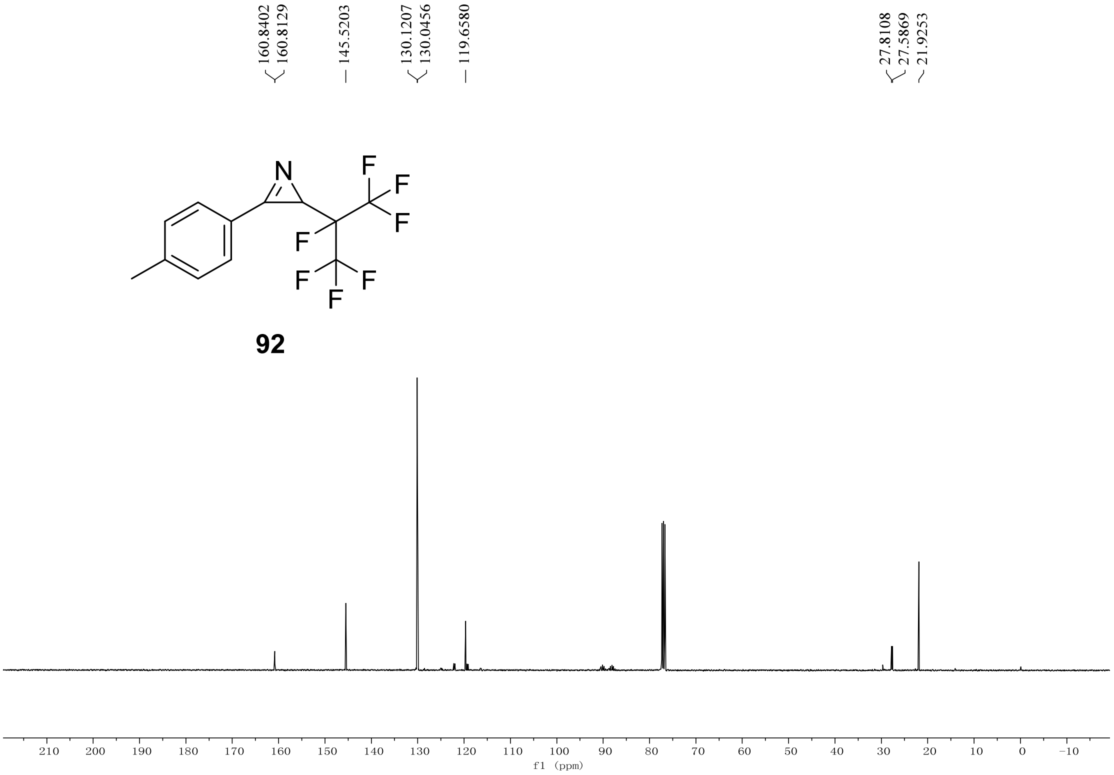

Supplementary Figure 238. ^{13}C NMR spectrum for compound **92**

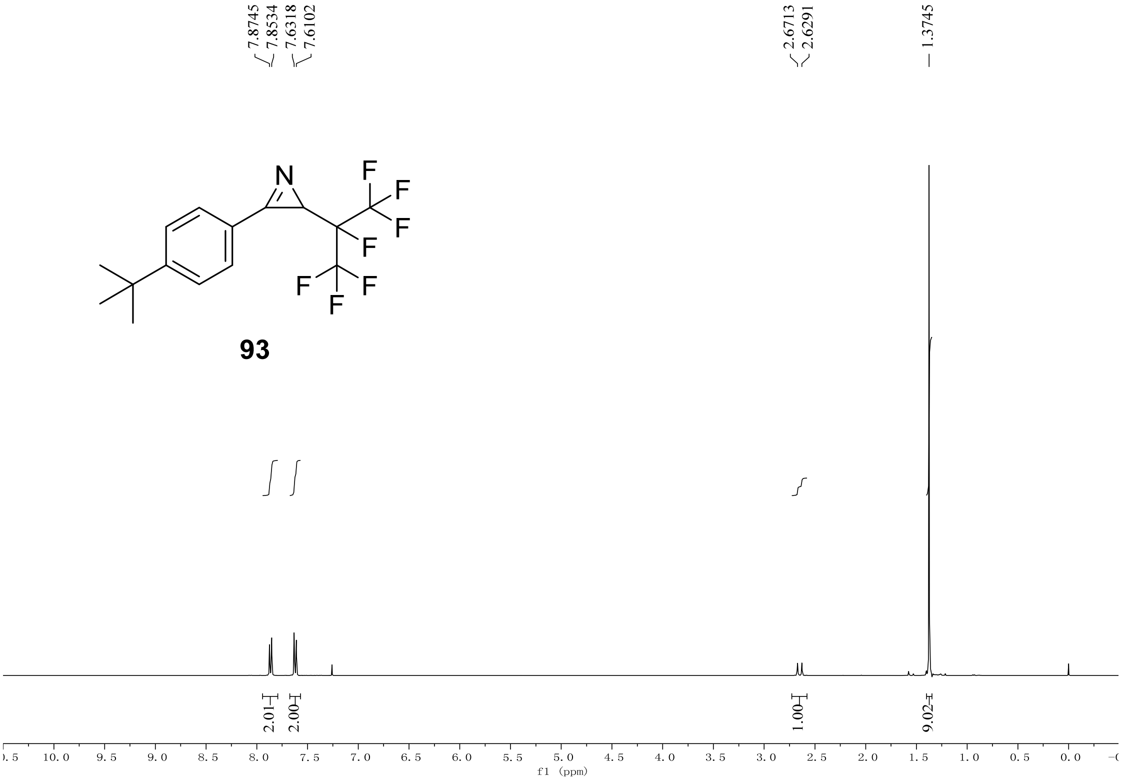

Supplementary Figure 239. ^{1}H NMR spectrum for compound **93**

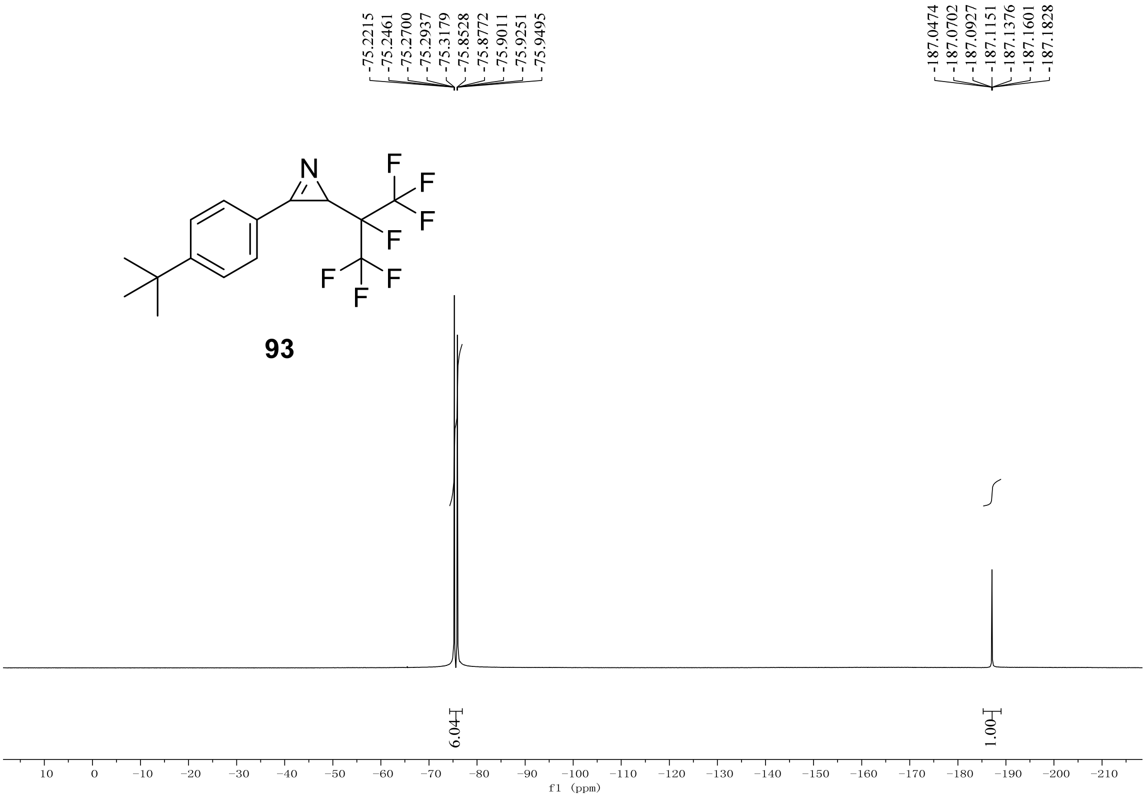

Supplementary Figure 240. ^{19}F NMR spectrum for compound **93**

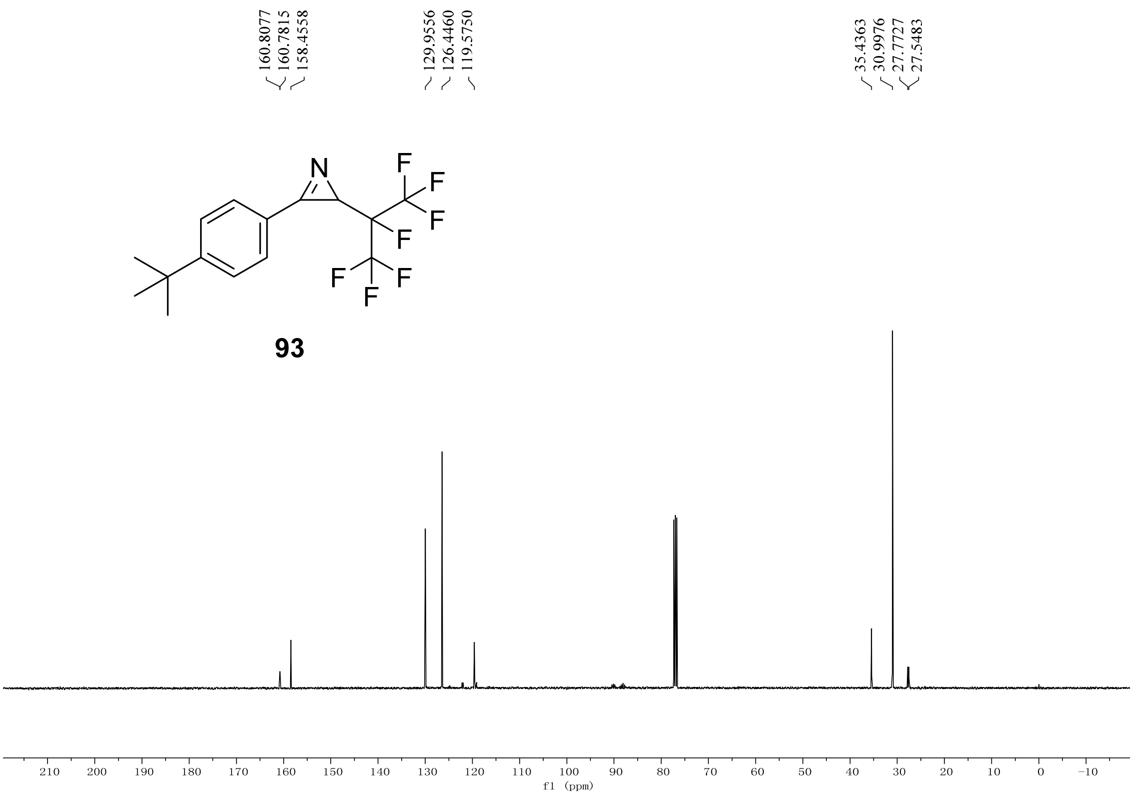

Supplementary Figure 241. ^{13}C NMR spectrum for compound **93**

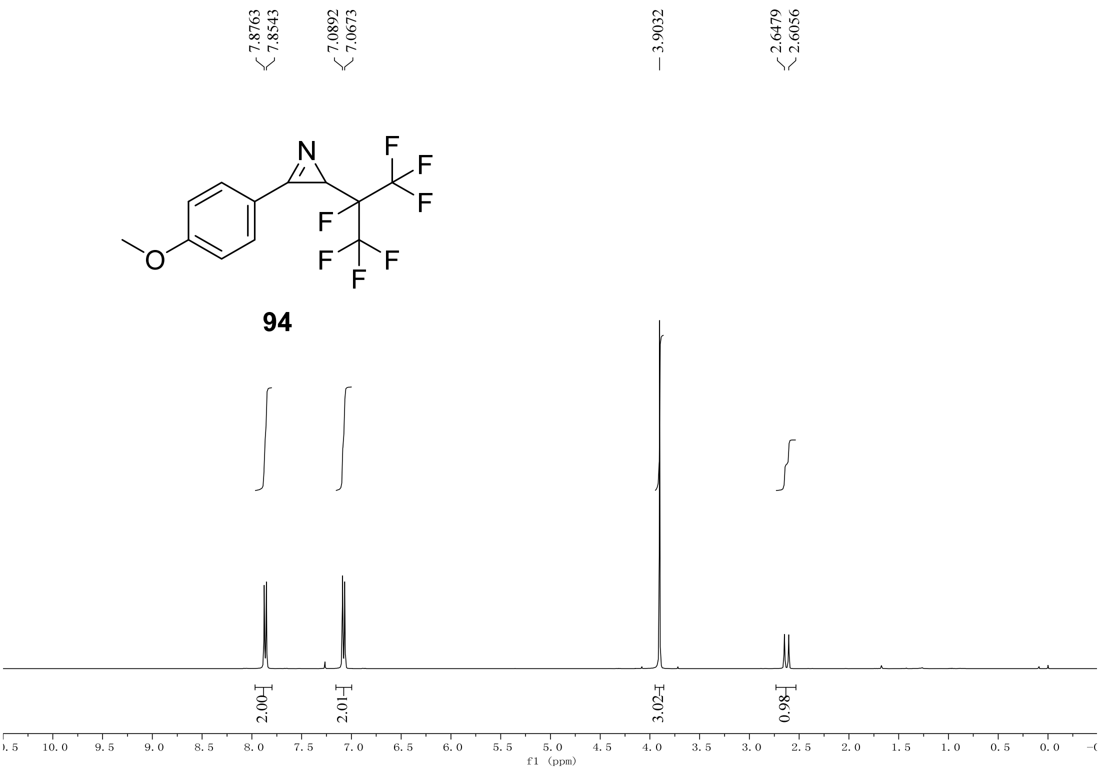

Supplementary Figure 242. ^{1}H NMR spectrum for compound **94**

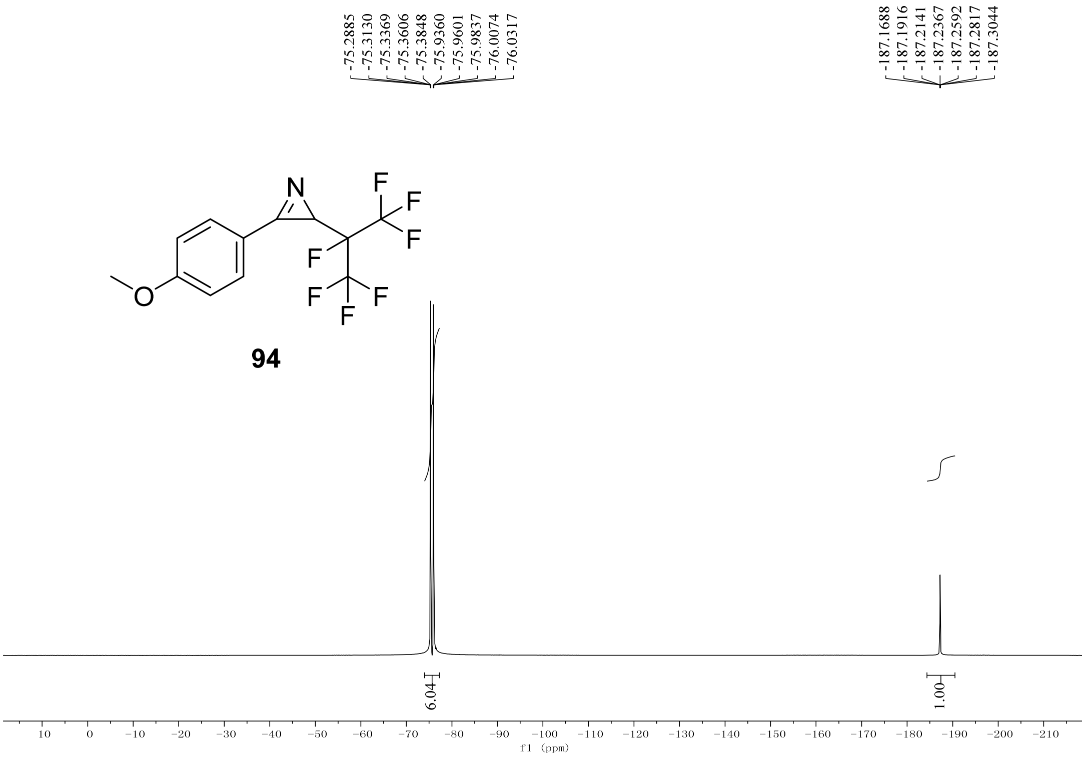

Supplementary Figure 243. ^{19}F NMR spectrum for compound **94**

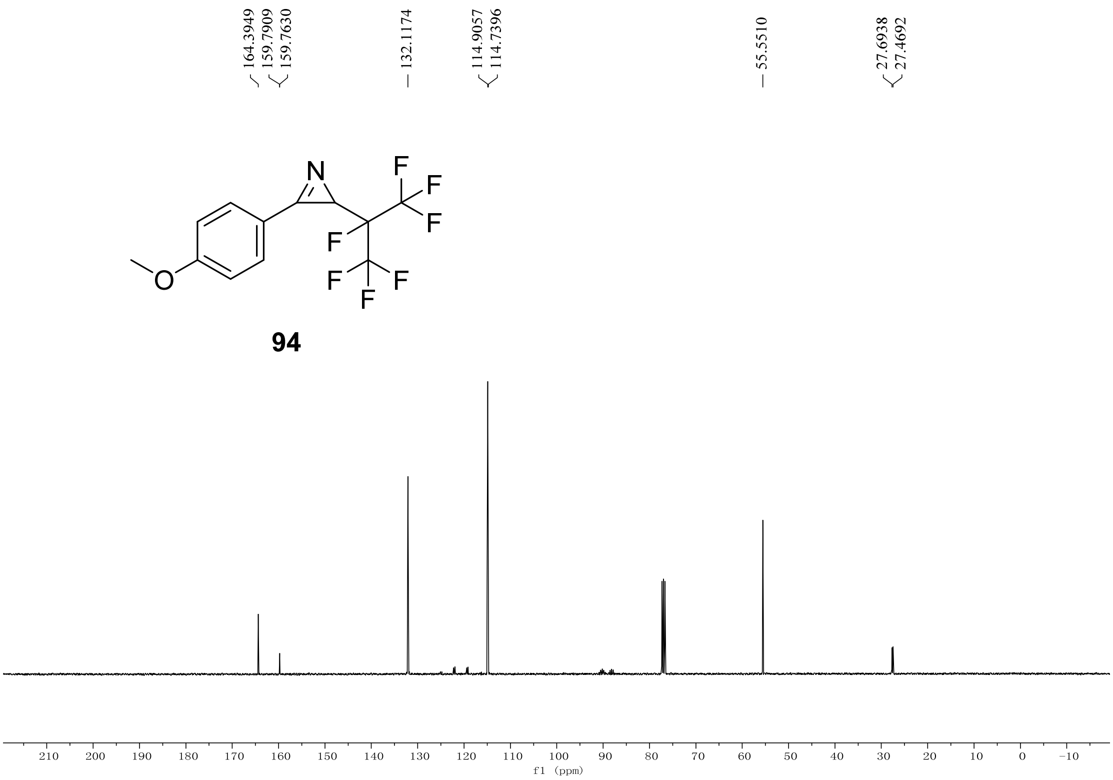

Supplementary Figure 244. ^{13}C NMR spectrum for compound **94**

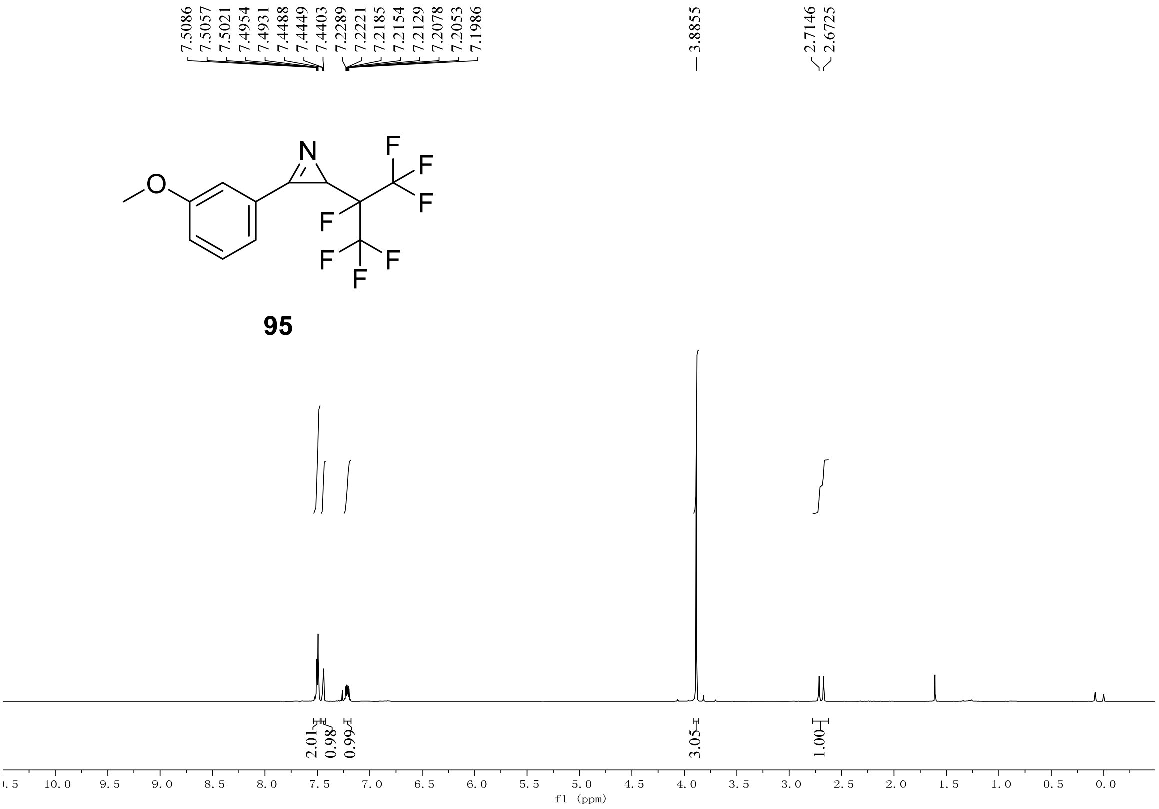

Supplementary Figure 245. ^{1}H NMR spectrum for compound **95**

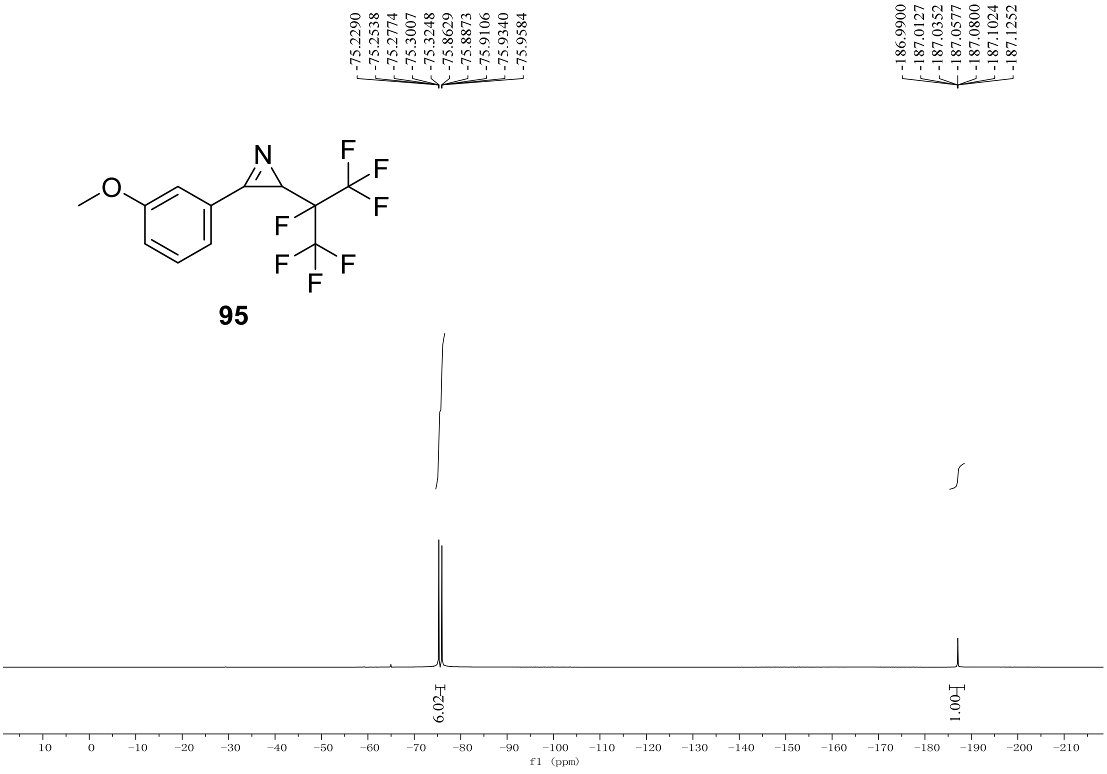

Supplementary Figure 246. ^{19}F NMR spectrum for compound **95**

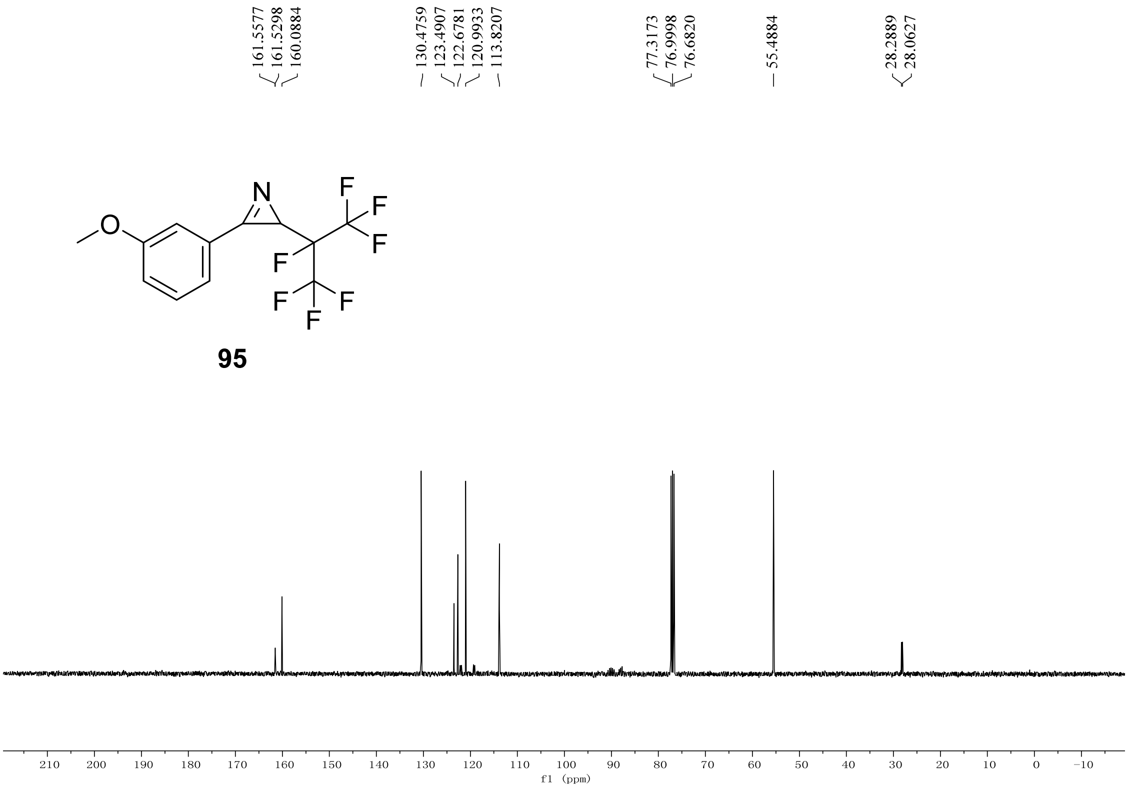

Supplementary Figure 247. ^{13}C NMR spectrum for compound **95**

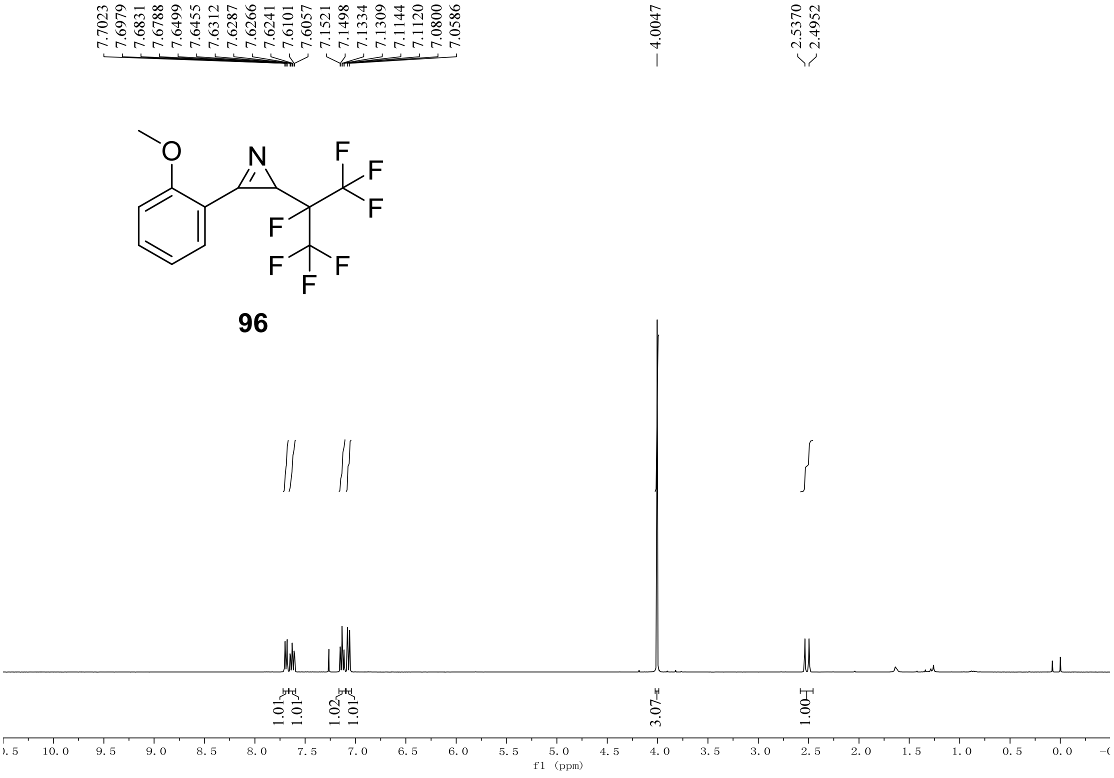

Supplementary Figure 248. ^{1}H NMR spectrum for compound **96**

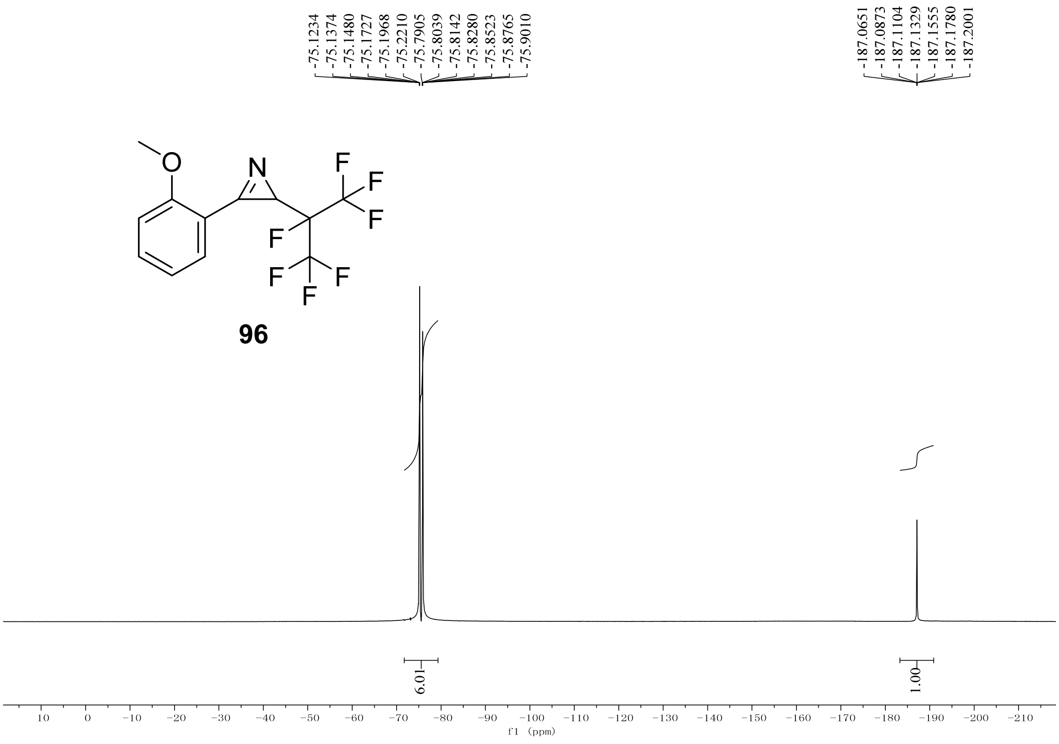

Supplementary Figure 249. ^{19}F NMR spectrum for compound **96**

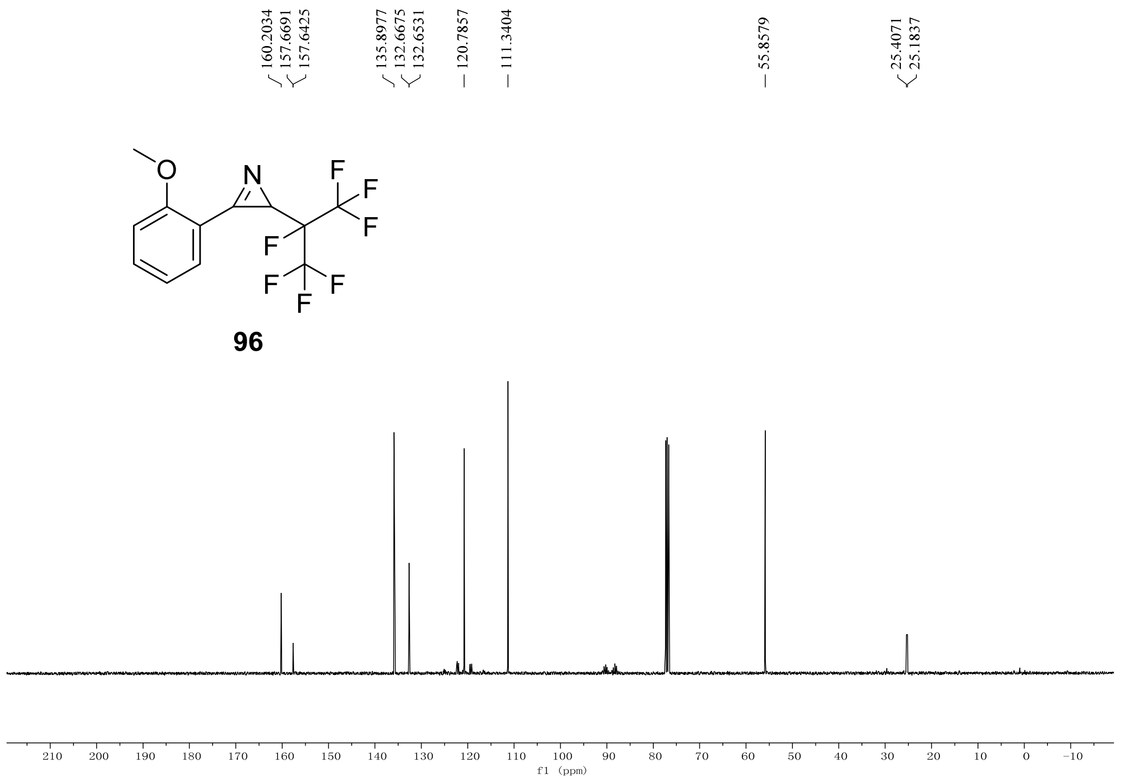

Supplementary Figure 250. ^{13}C NMR spectrum for compound **96**

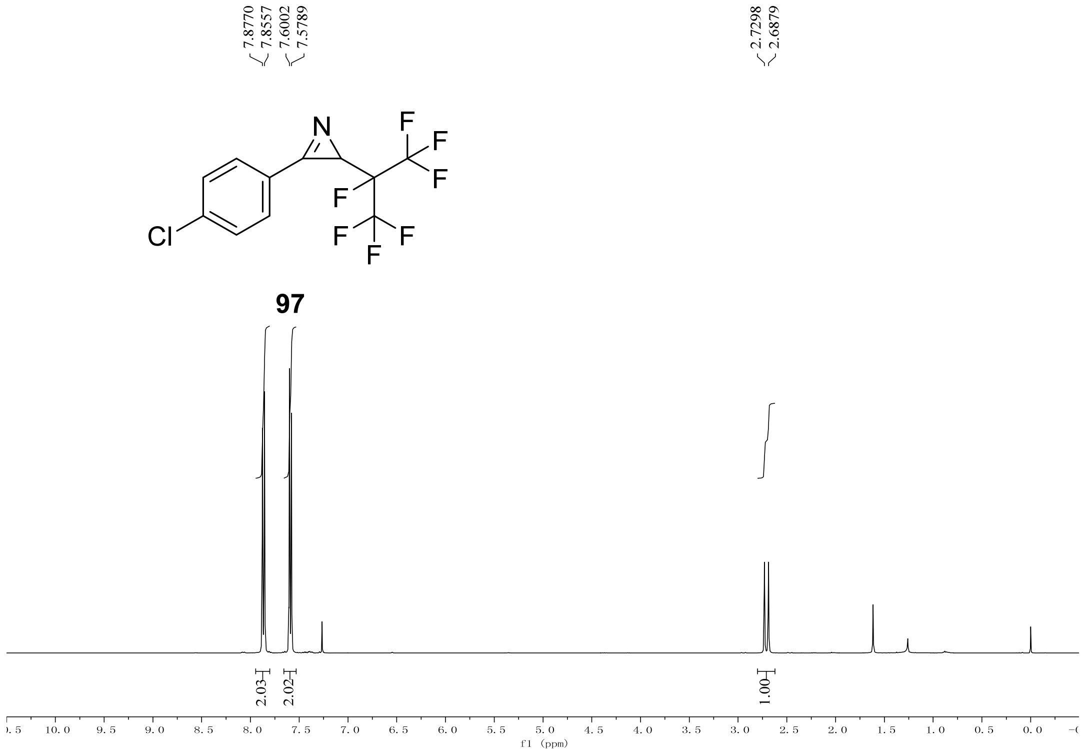

Supplementary Figure 251. ^{1}H NMR spectrum for compound **97**

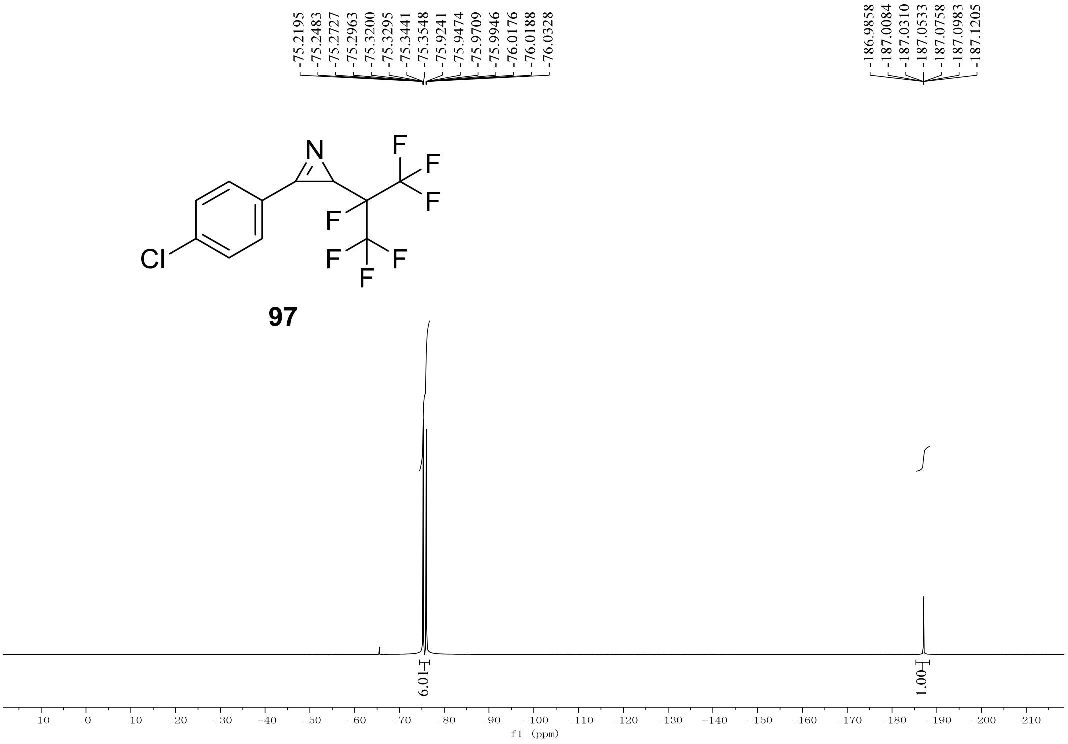

Supplementary Figure 252. ^{19}F NMR spectrum for compound **97**

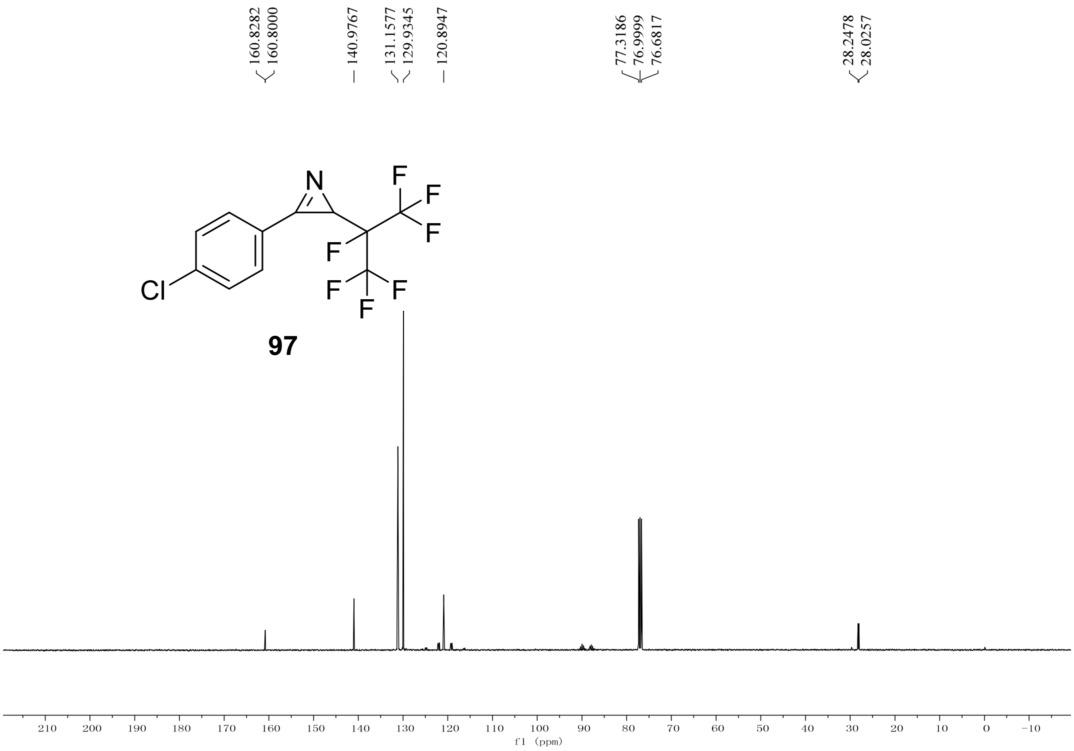

Supplementary Figure 253. ^{13}C NMR spectrum for compound **97**

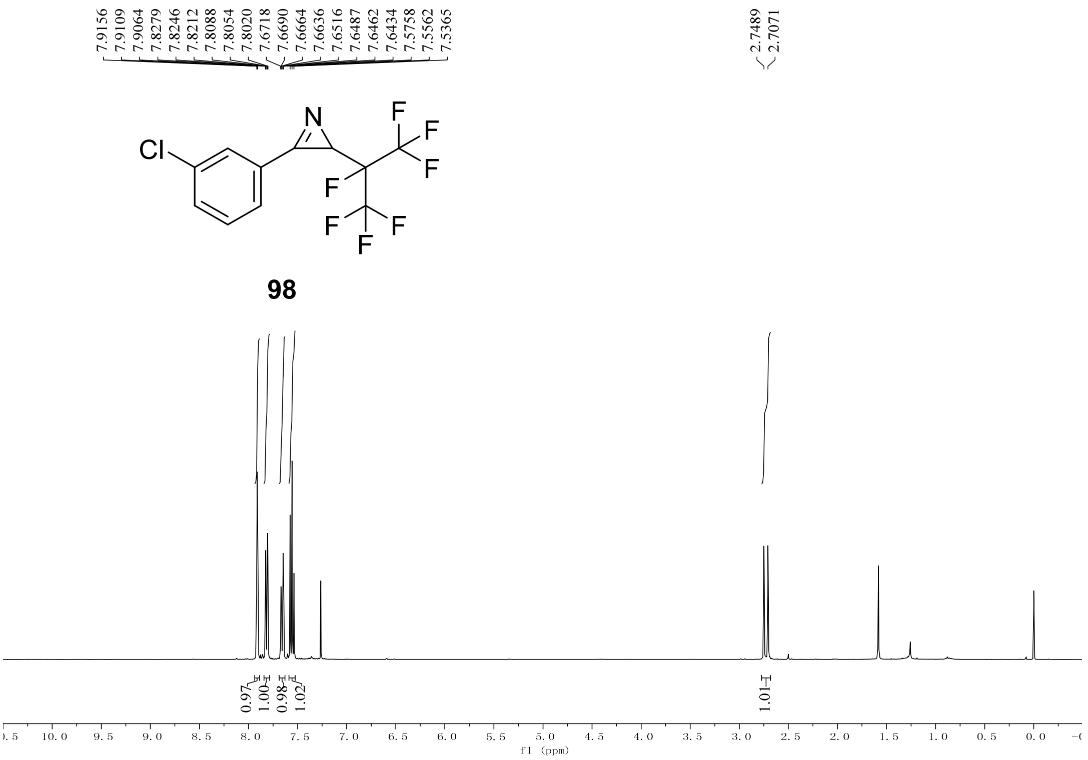

Supplementary Figure 254. ^{1}H NMR spectrum for compound **98**

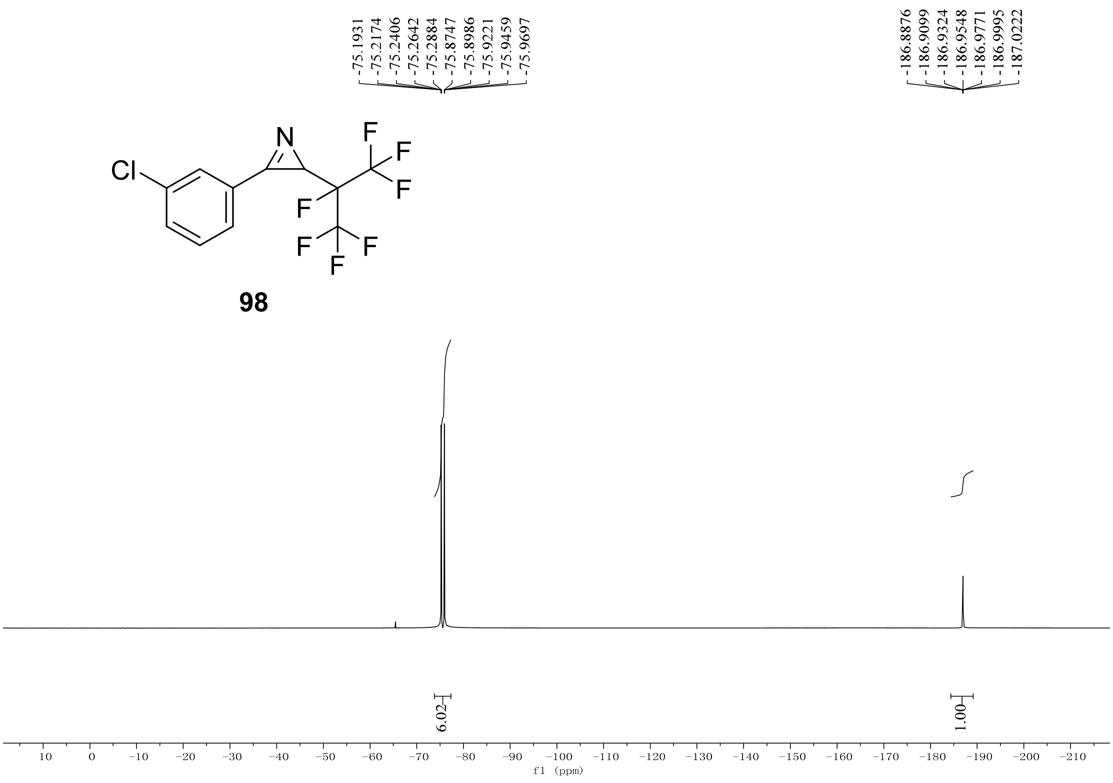

Supplementary Figure 255. ^{19}F NMR spectrum for compound **98**

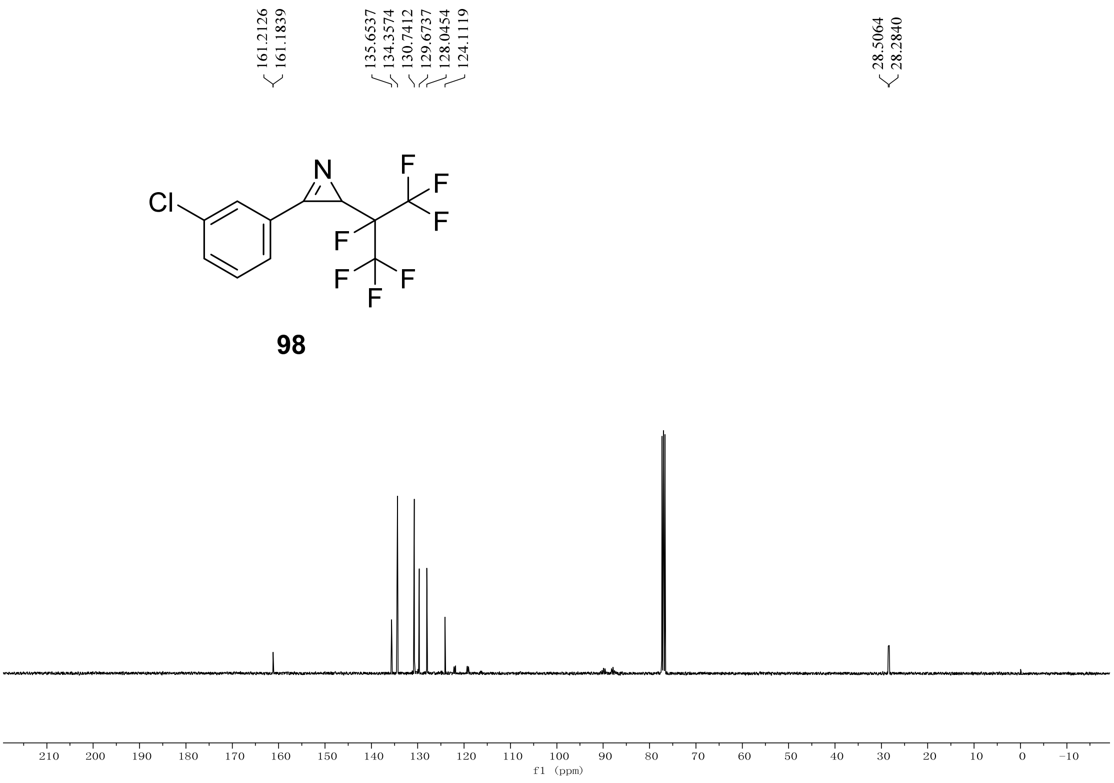

Supplementary Figure 256. ^{13}C NMR spectrum for compound **98**

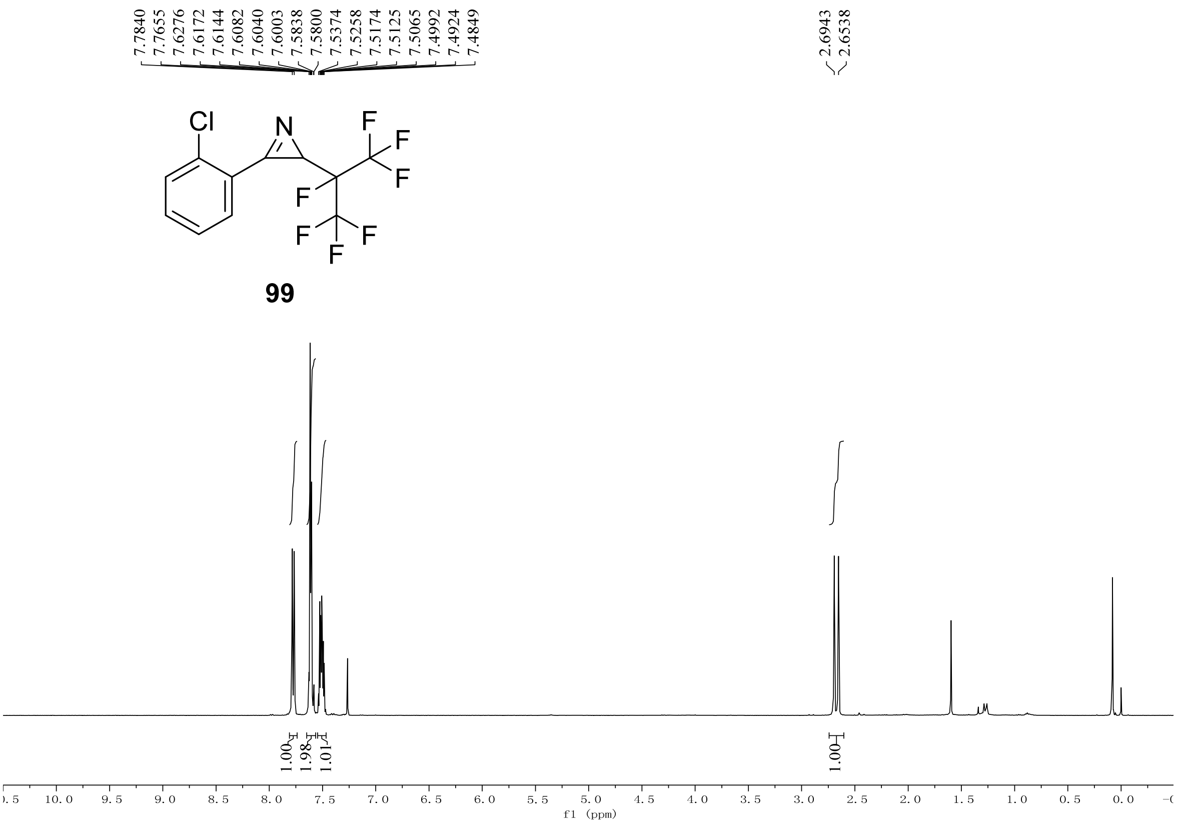

Supplementary Figure 257. ^{1}H NMR spectrum for compound **99**

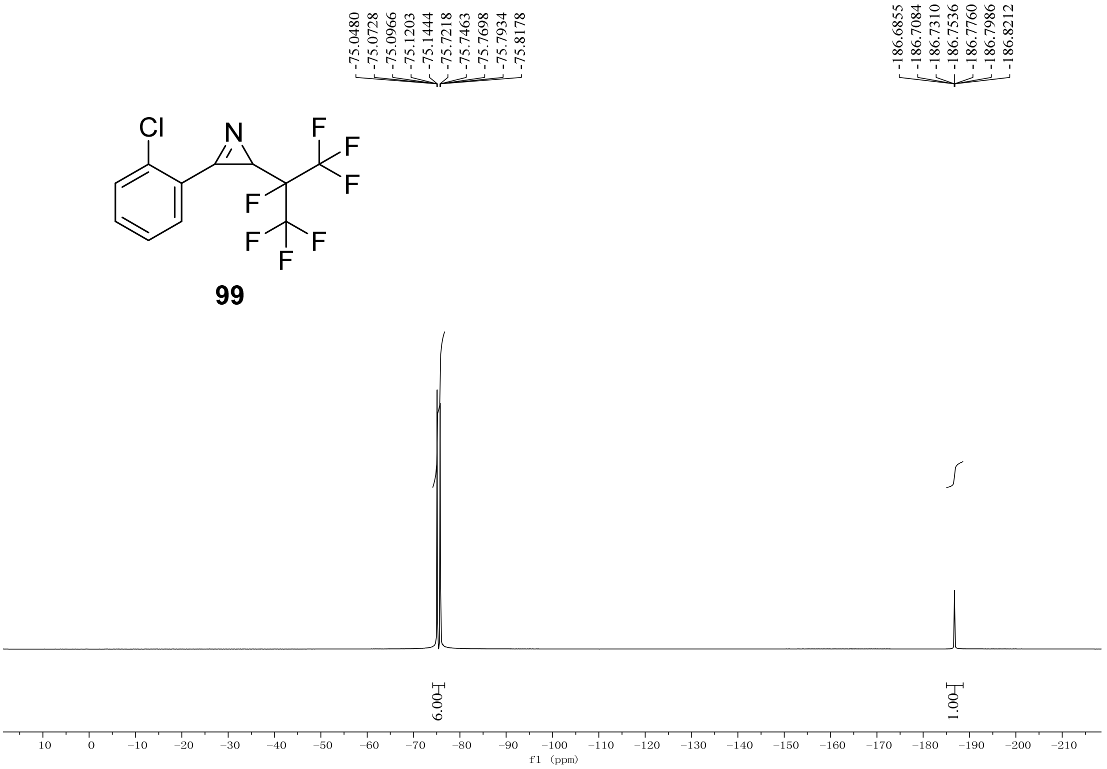

Supplementary Figure 258. ^{19}F NMR spectrum for compound **99**

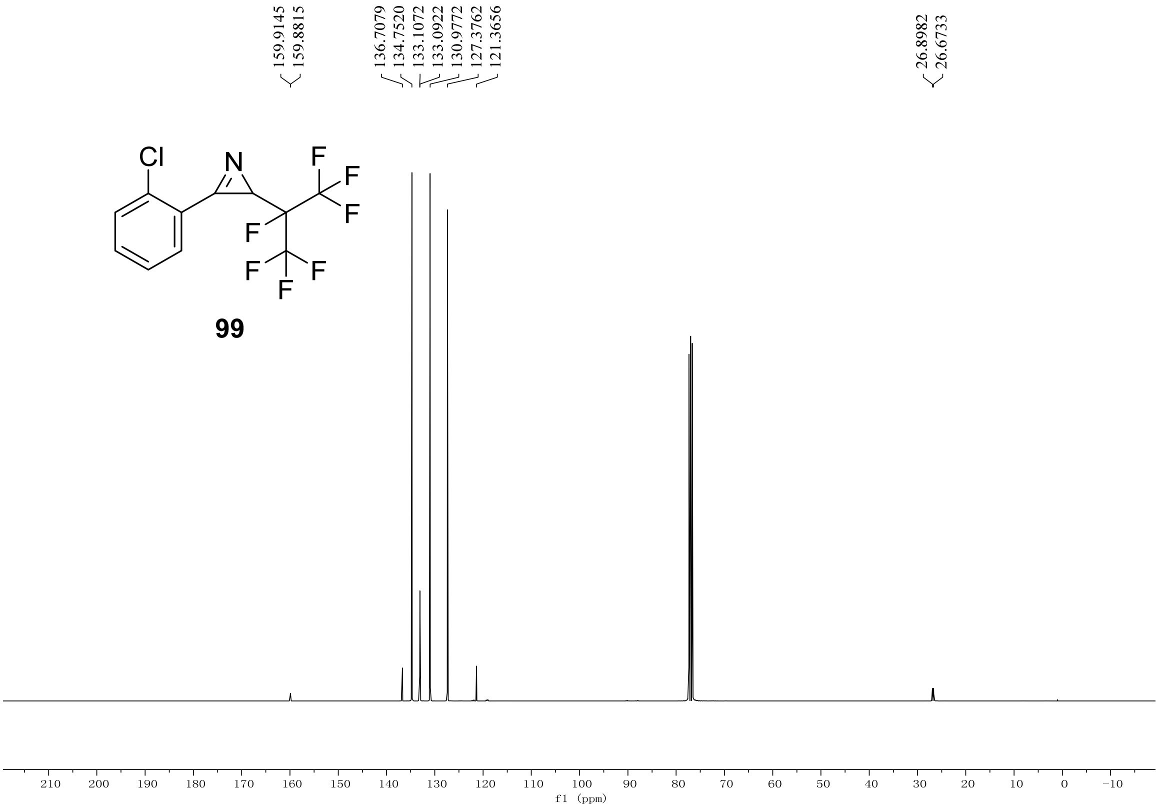

Supplementary Figure 259. ^{13}C NMR spectrum for compound **99**

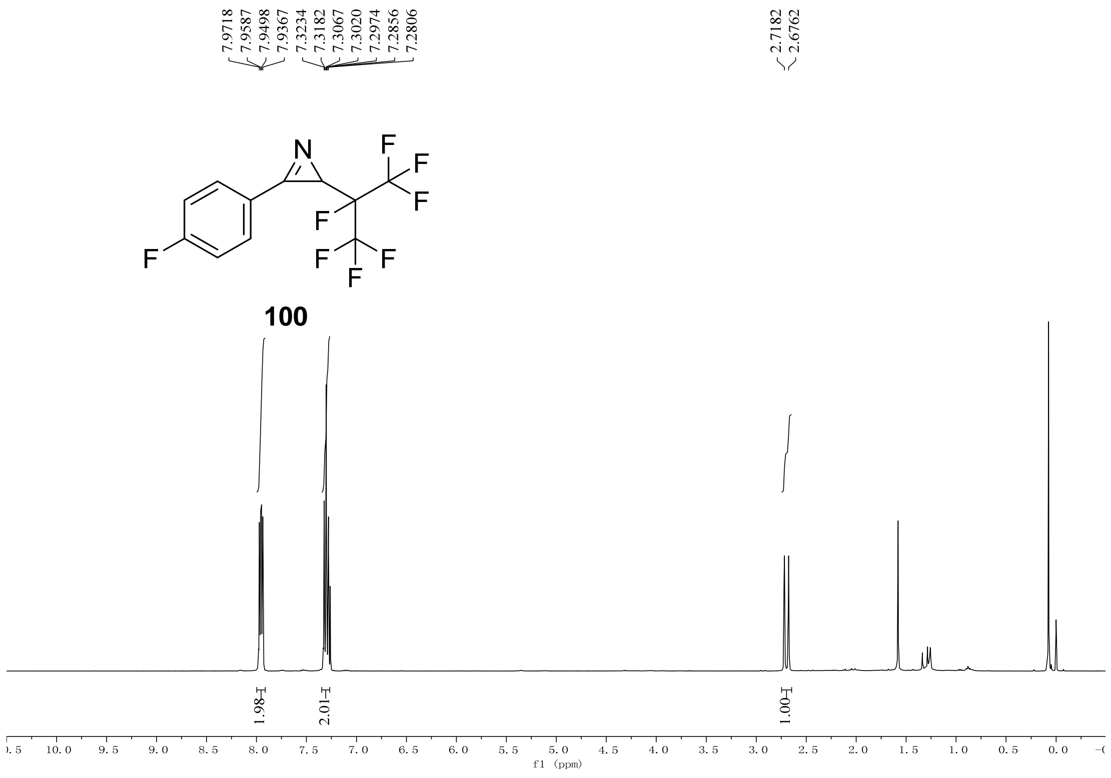

Supplementary Figure 260. ^{1}H NMR spectrum for compound **100**

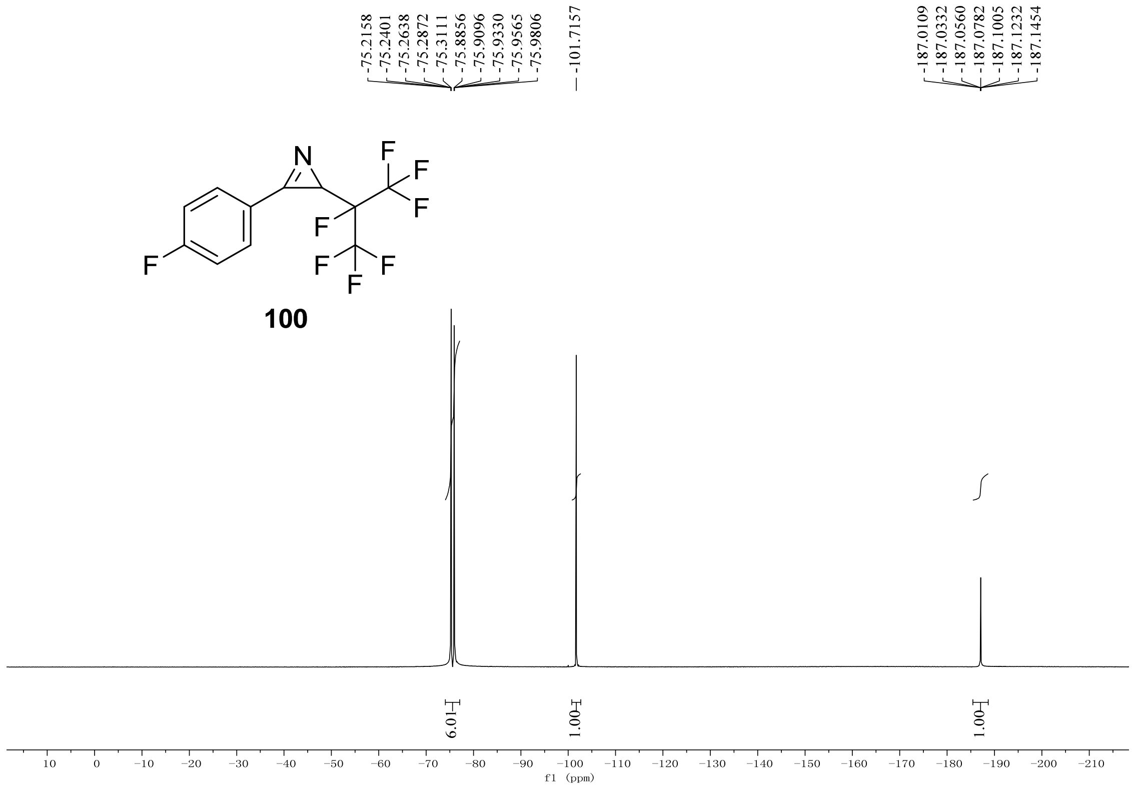

Supplementary Figure 261. ^{19}F NMR spectrum for compound **100**

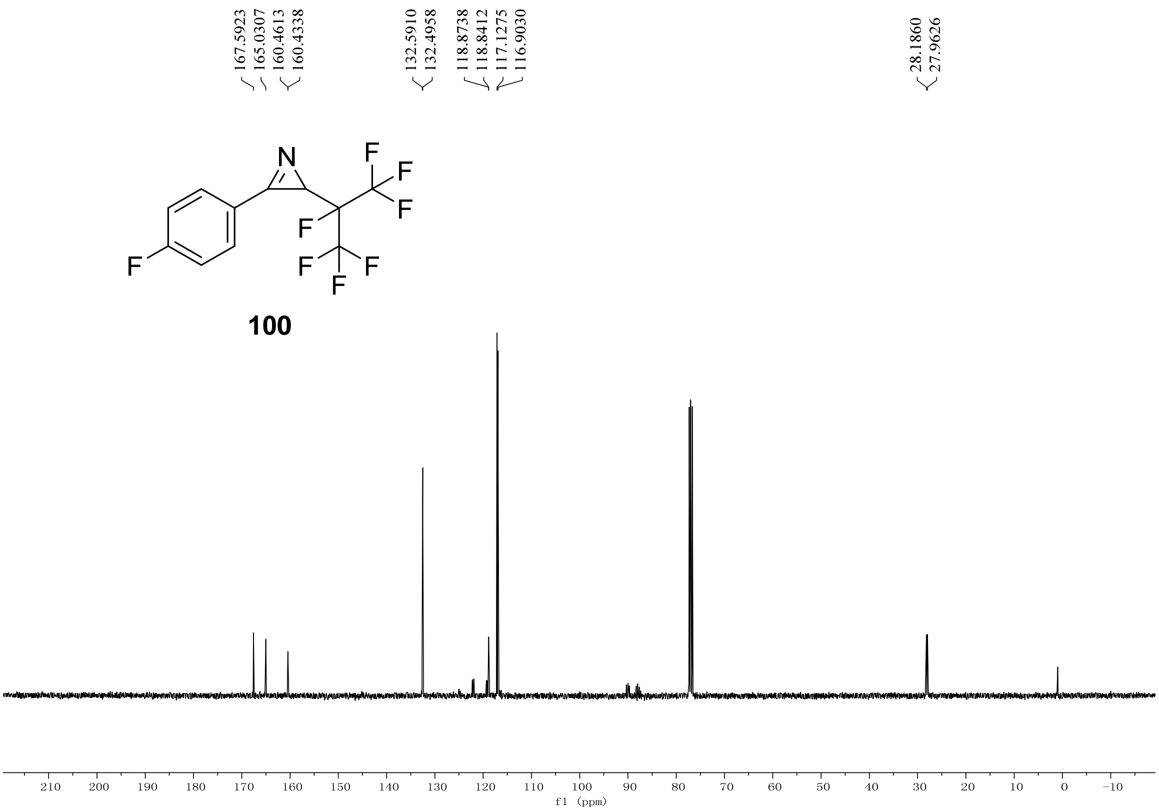

Supplementary Figure 262. ^{13}C NMR spectrum for compound **100**

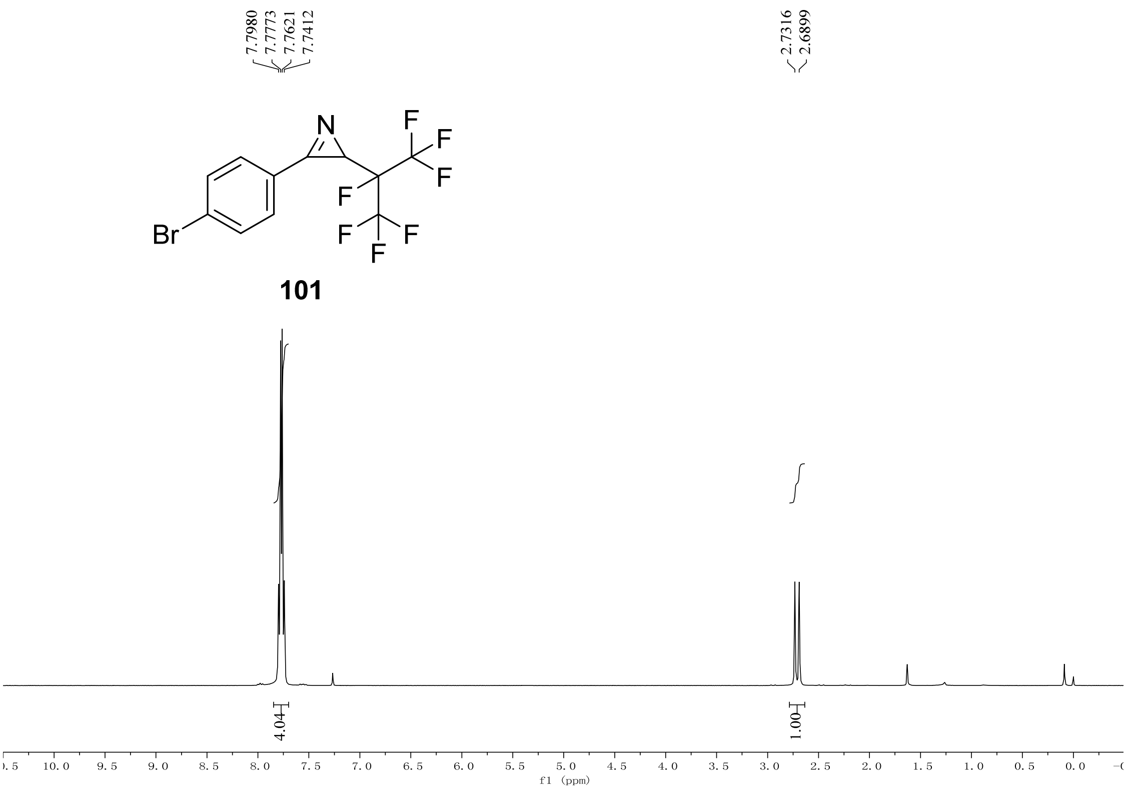

Supplementary Figure 263. ^{1}H NMR spectrum for compound **101**

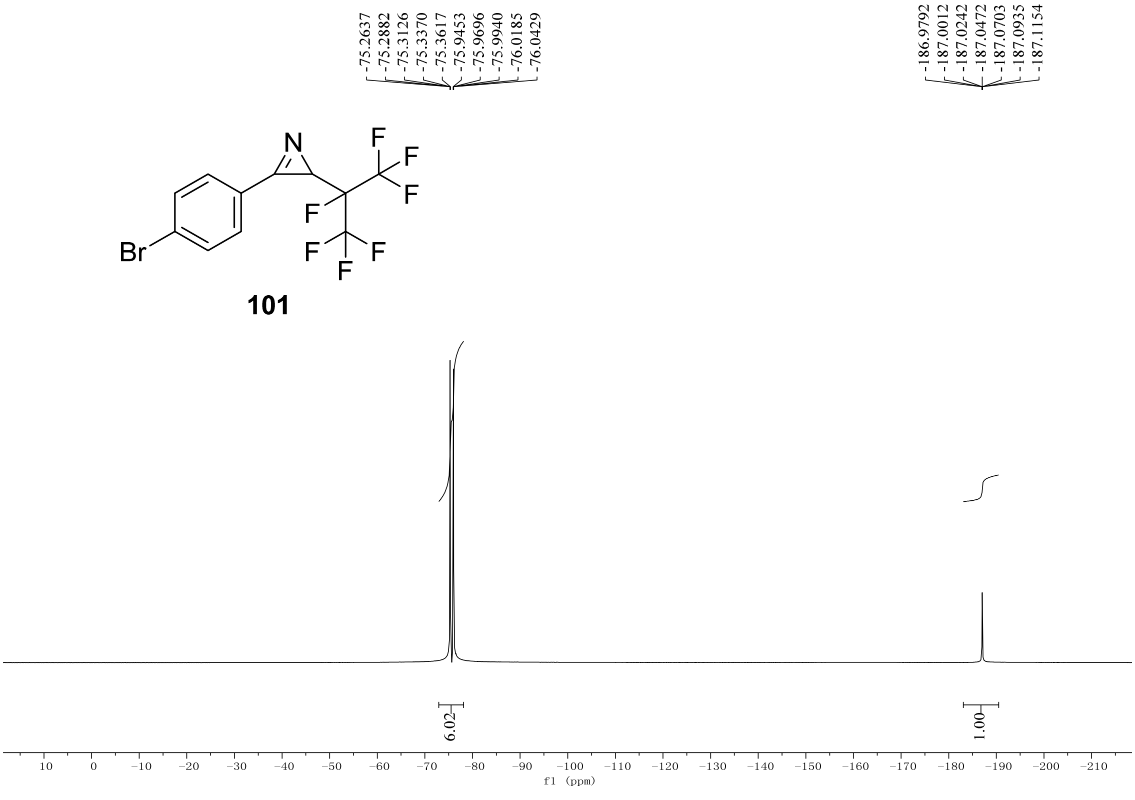

Supplementary Figure 264. ^{19}F NMR spectrum for compound **101**

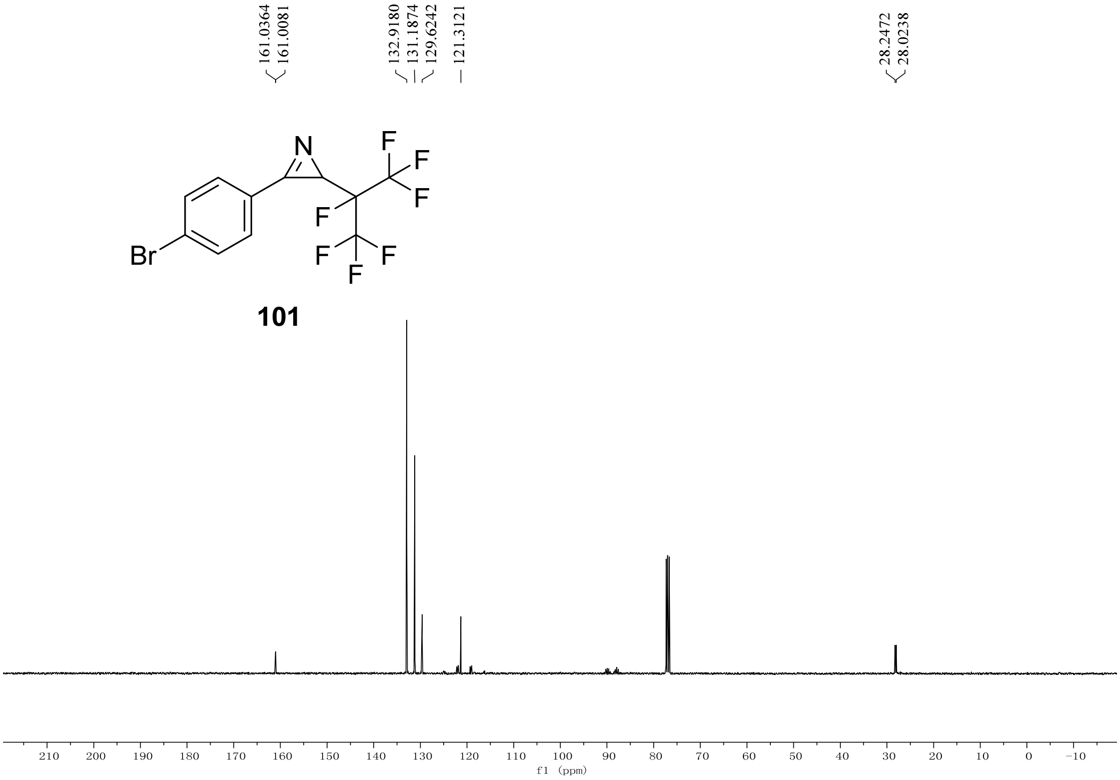

Supplementary Figure 265. ^{13}C NMR spectrum for compound **101**

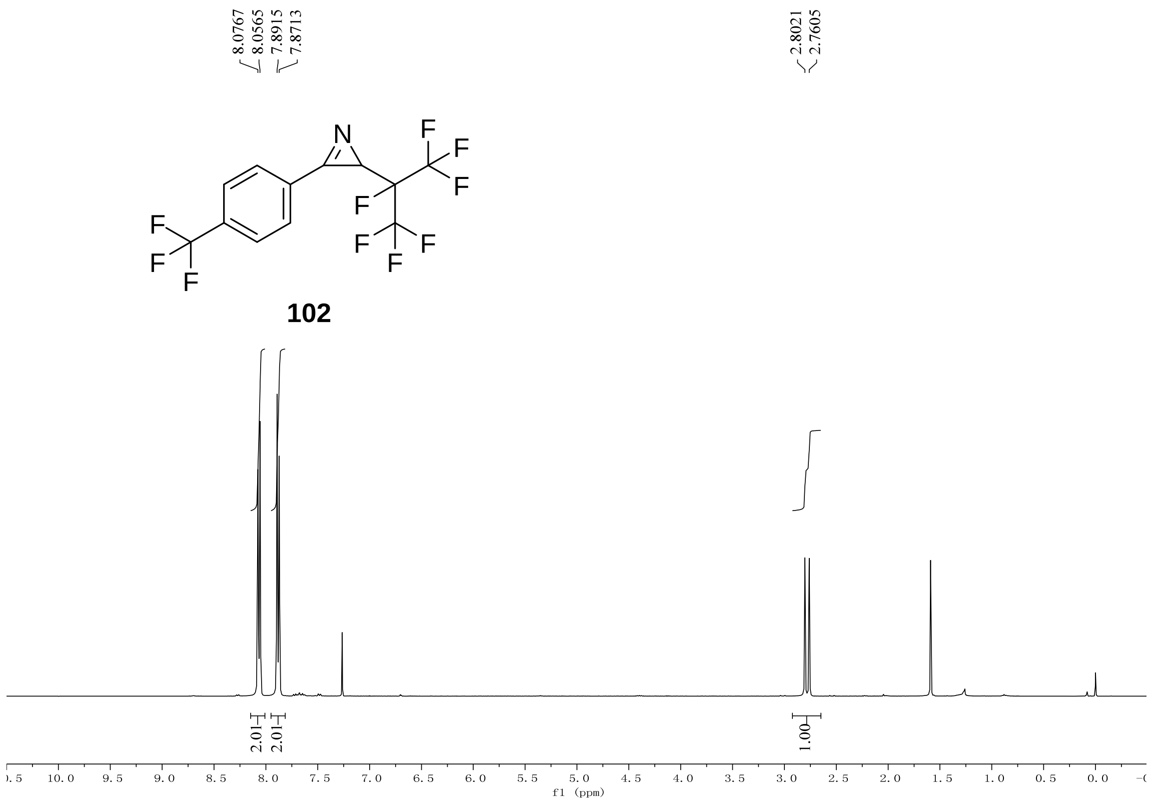

Supplementary Figure 266. ^{1}H NMR spectrum for compound **102**

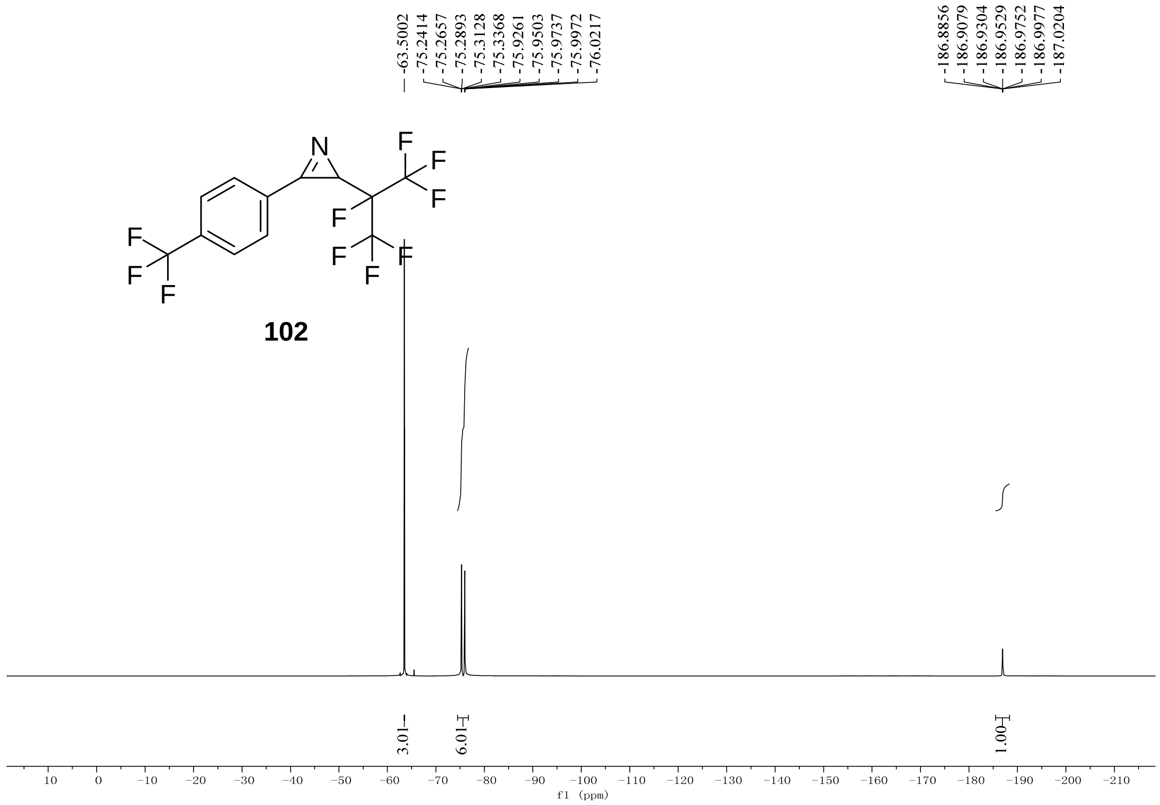

Supplementary Figure 267. ^{19}F NMR spectrum for compound **102**

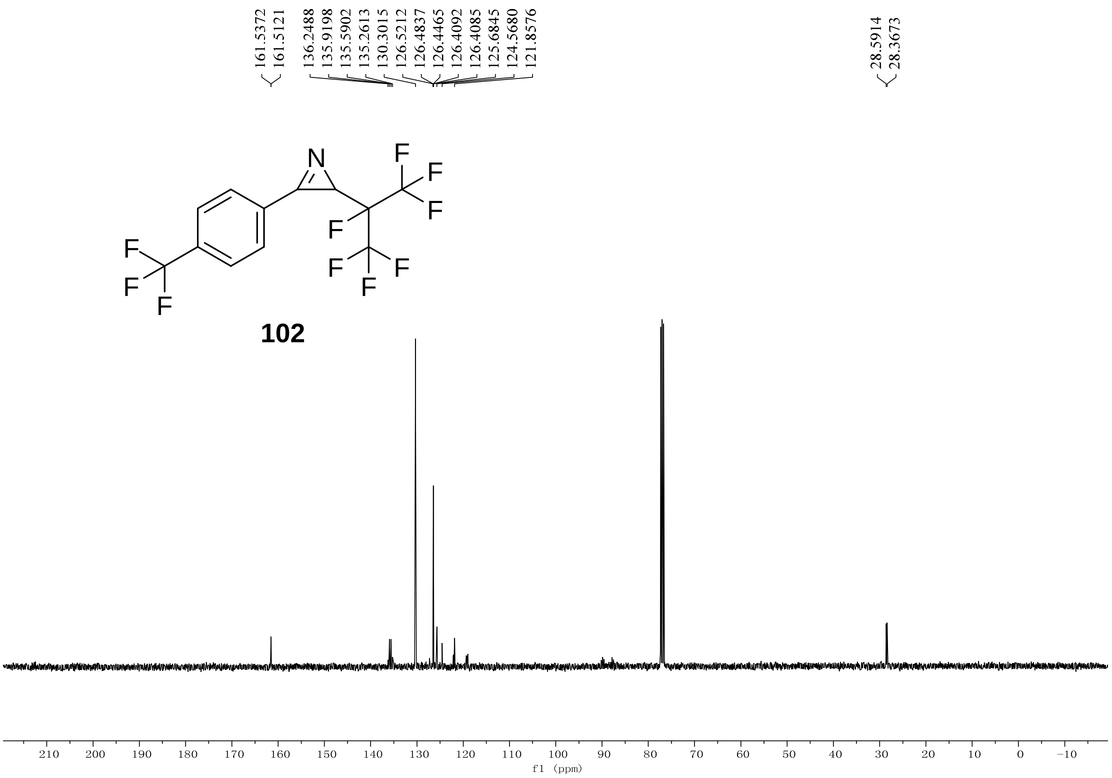

Supplementary Figure 268. ^{13}C NMR spectrum for compound **102**

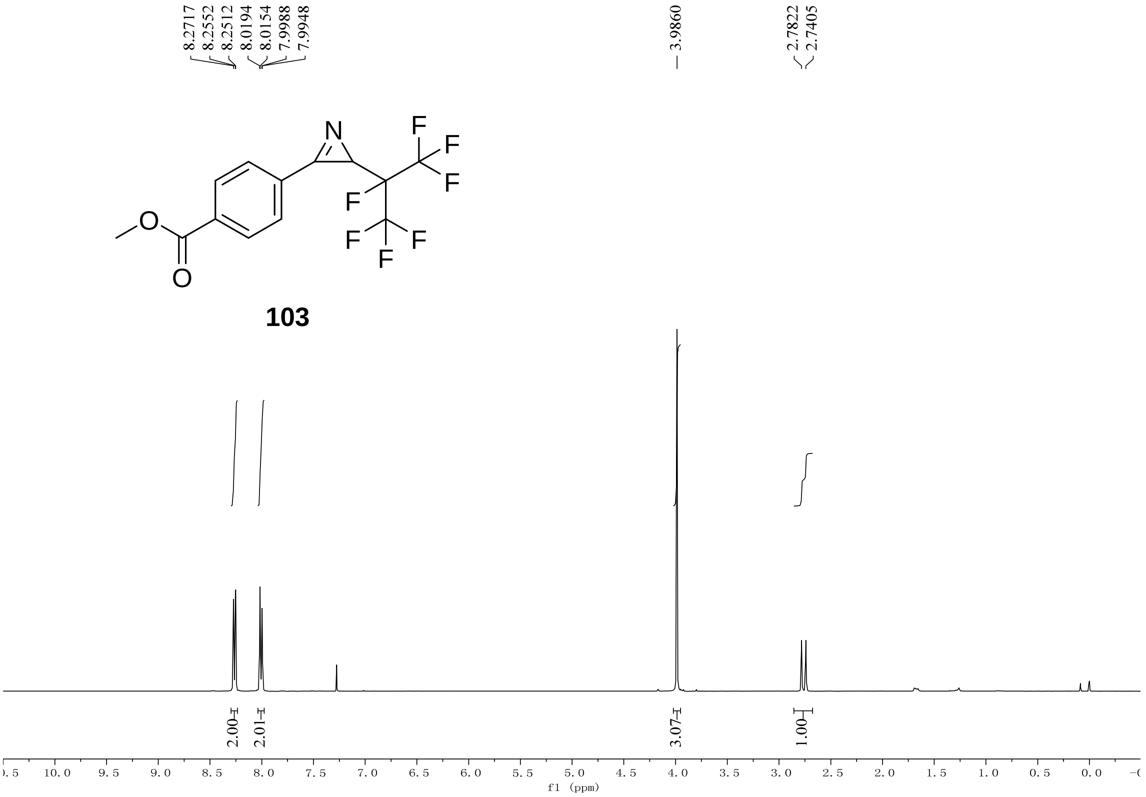

Supplementary Figure 269. ^{1}H NMR spectrum for compound **103**

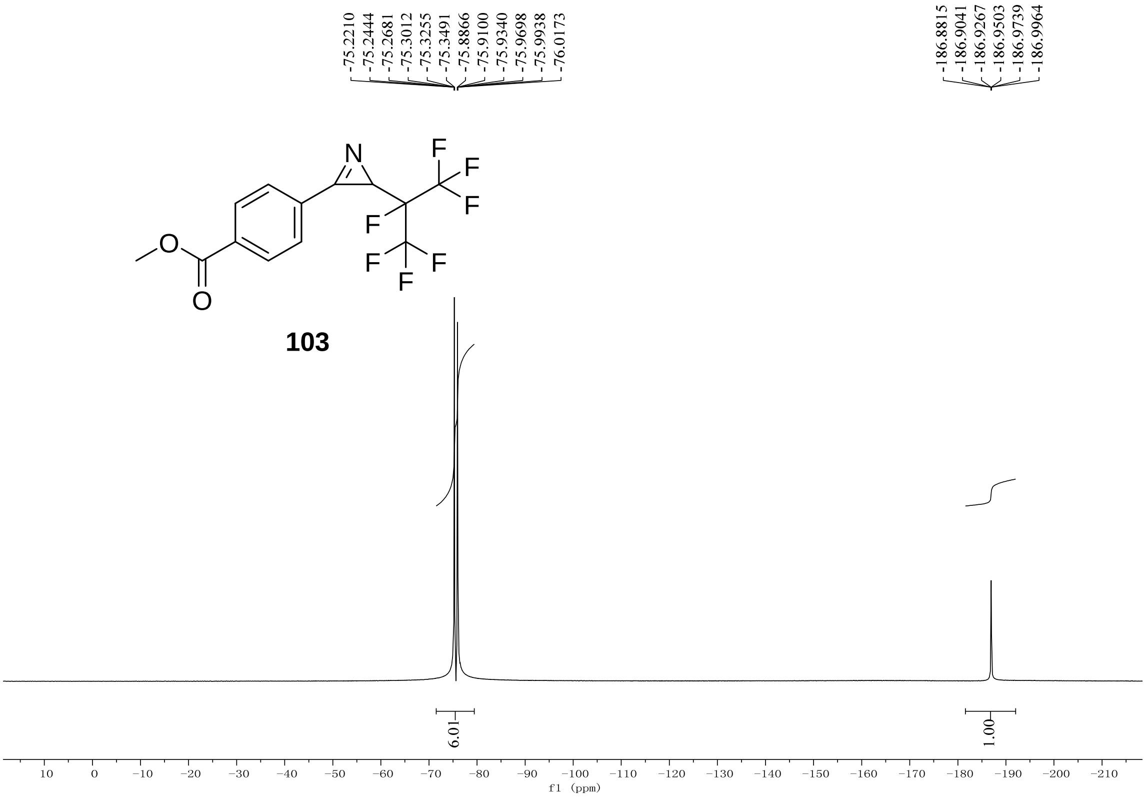

Supplementary Figure 270. ^{19}F NMR spectrum for compound **103**

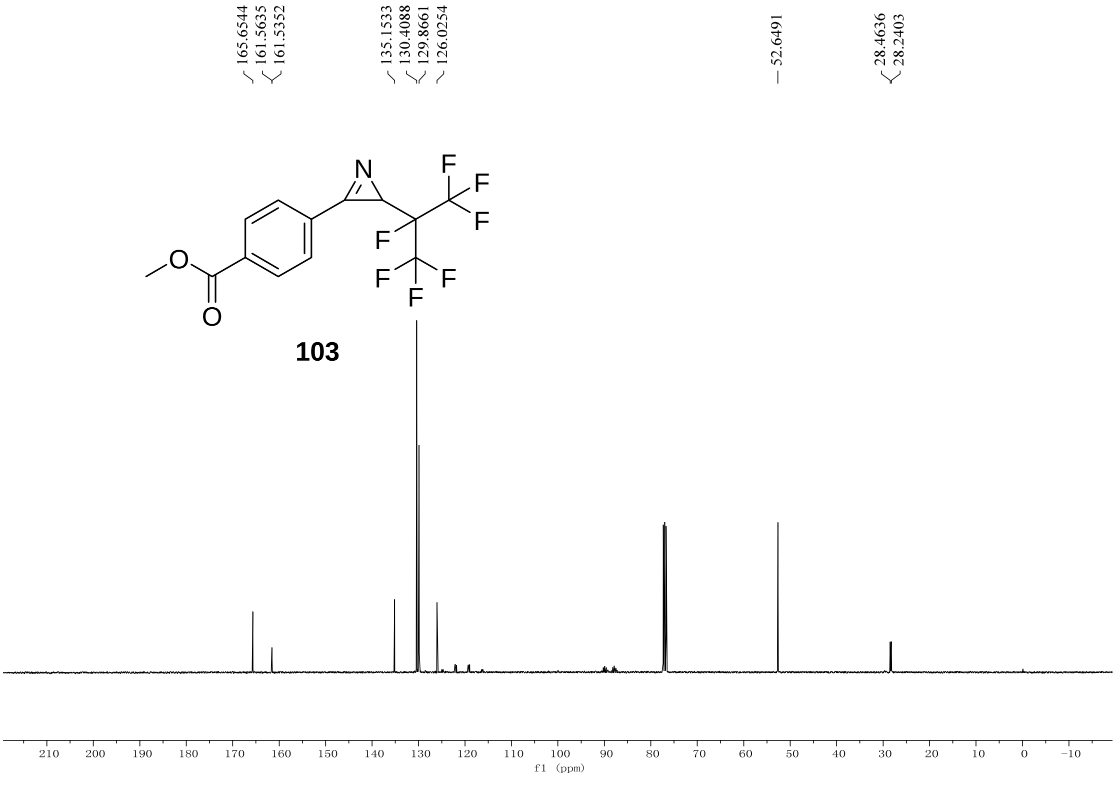

Supplementary Figure 271. ^{13}C NMR spectrum for compound **103**

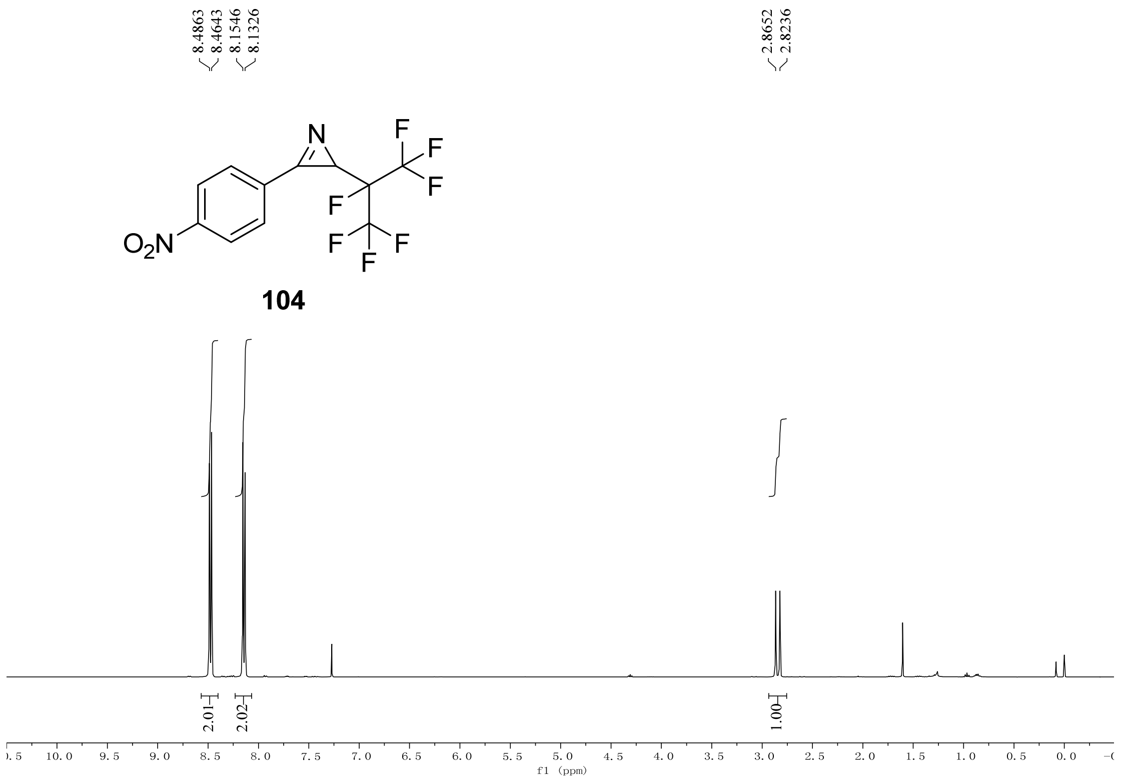

Supplementary Figure 272. ^{1}H NMR spectrum for compound **104**

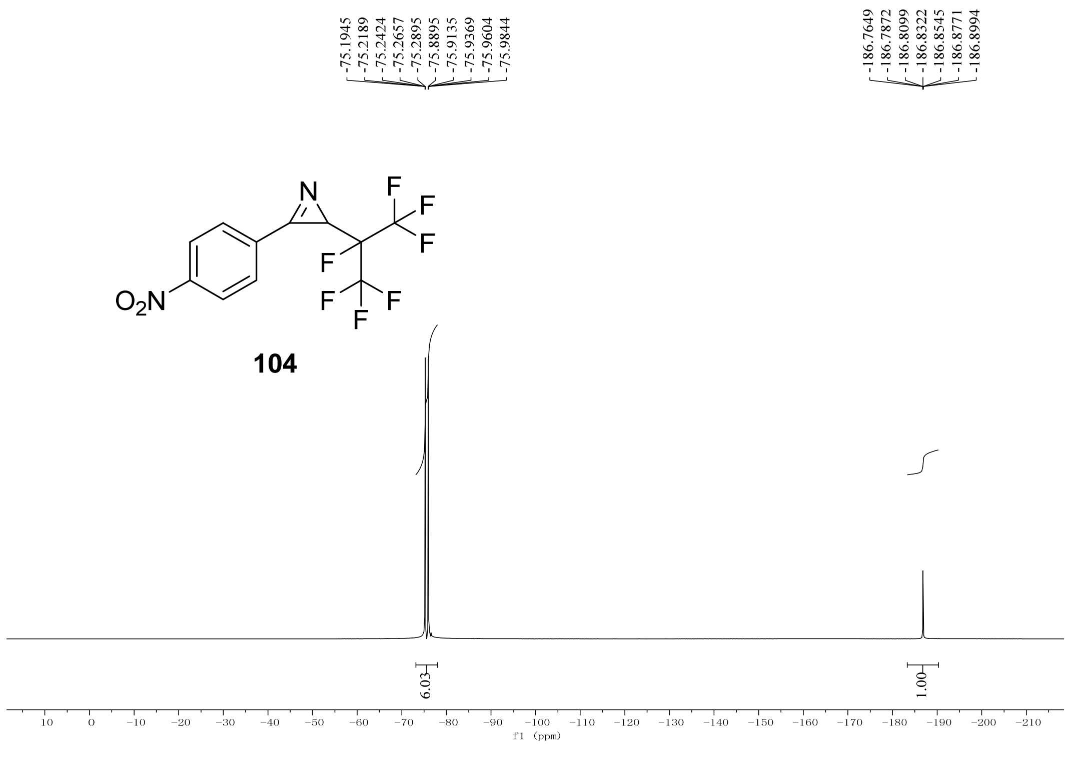

Supplementary Figure 273. ^{19}F NMR spectrum for compound **104**

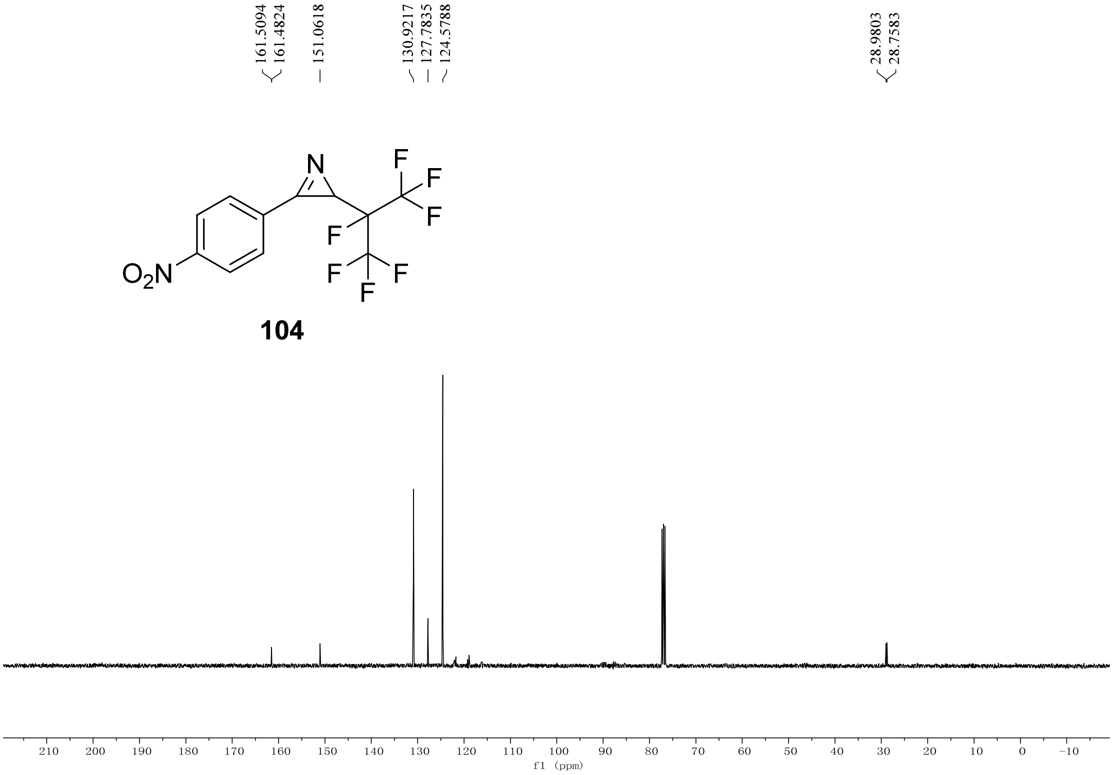

Supplementary Figure 274. ^{13}C NMR spectrum for compound **104**

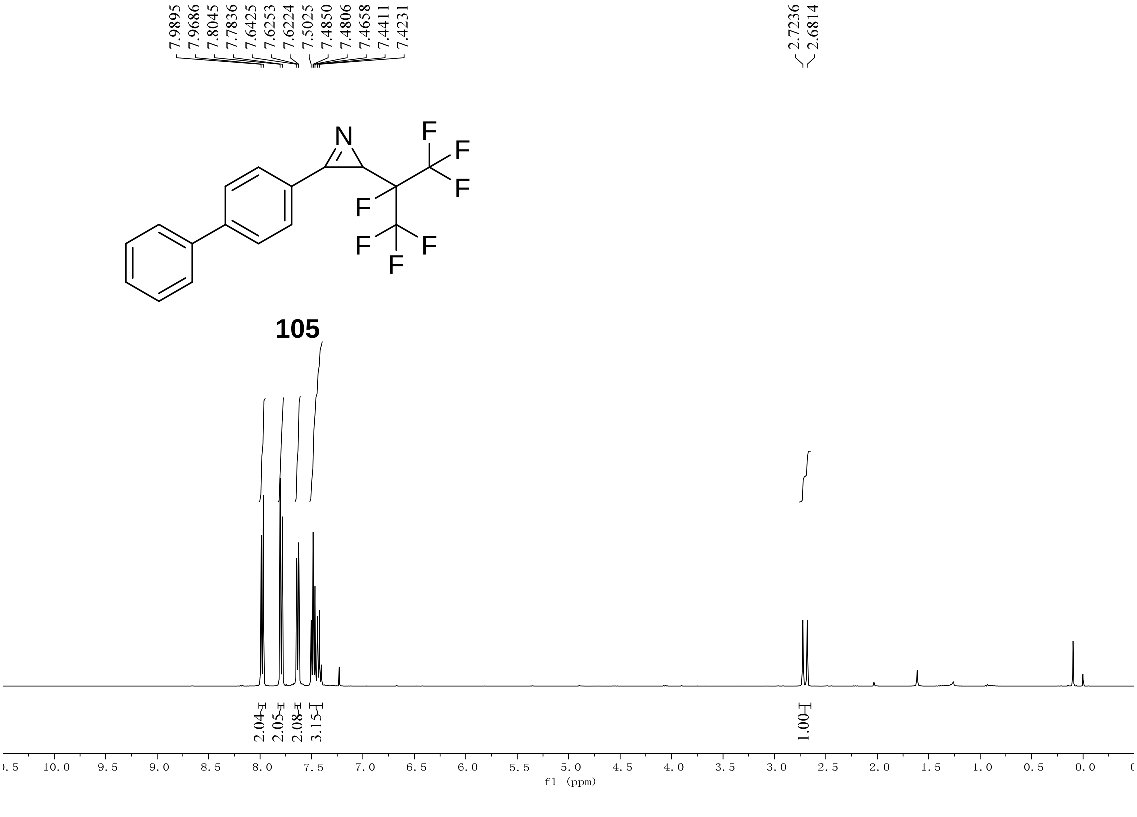

Supplementary Figure 275. ^{1}H NMR spectrum for compound **105**

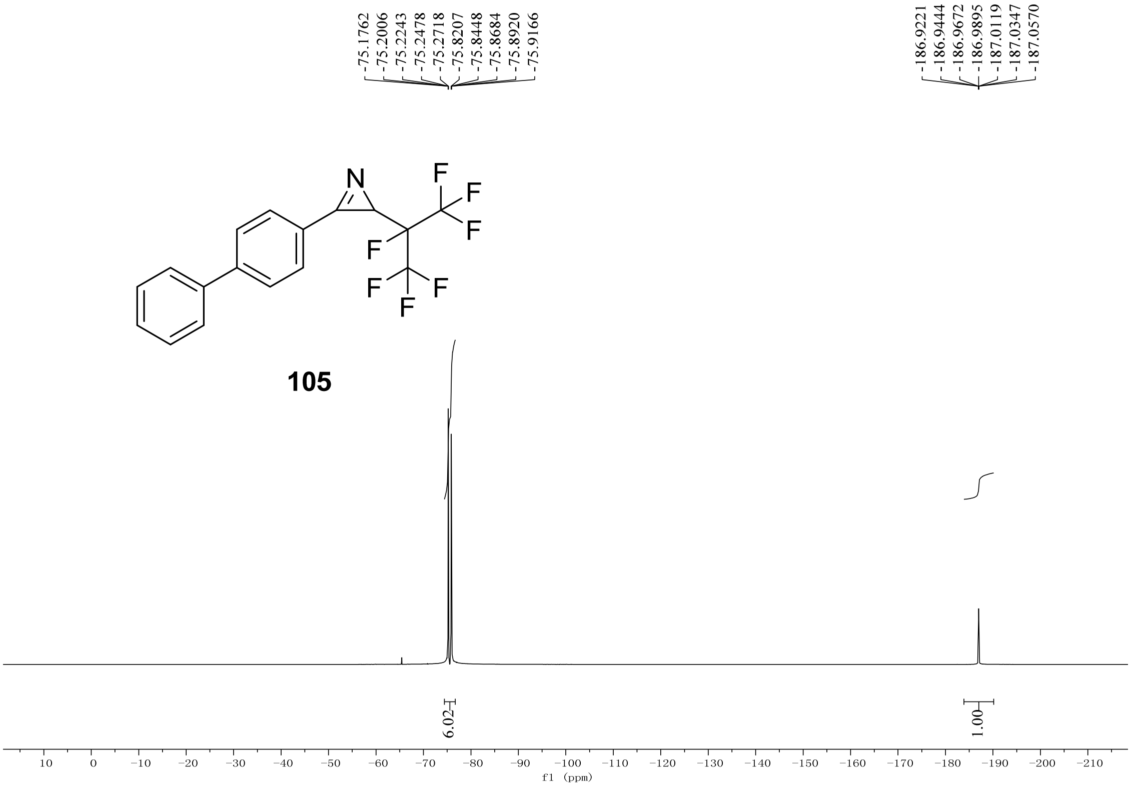

Supplementary Figure 276. ^{19}F NMR spectrum for compound **105**

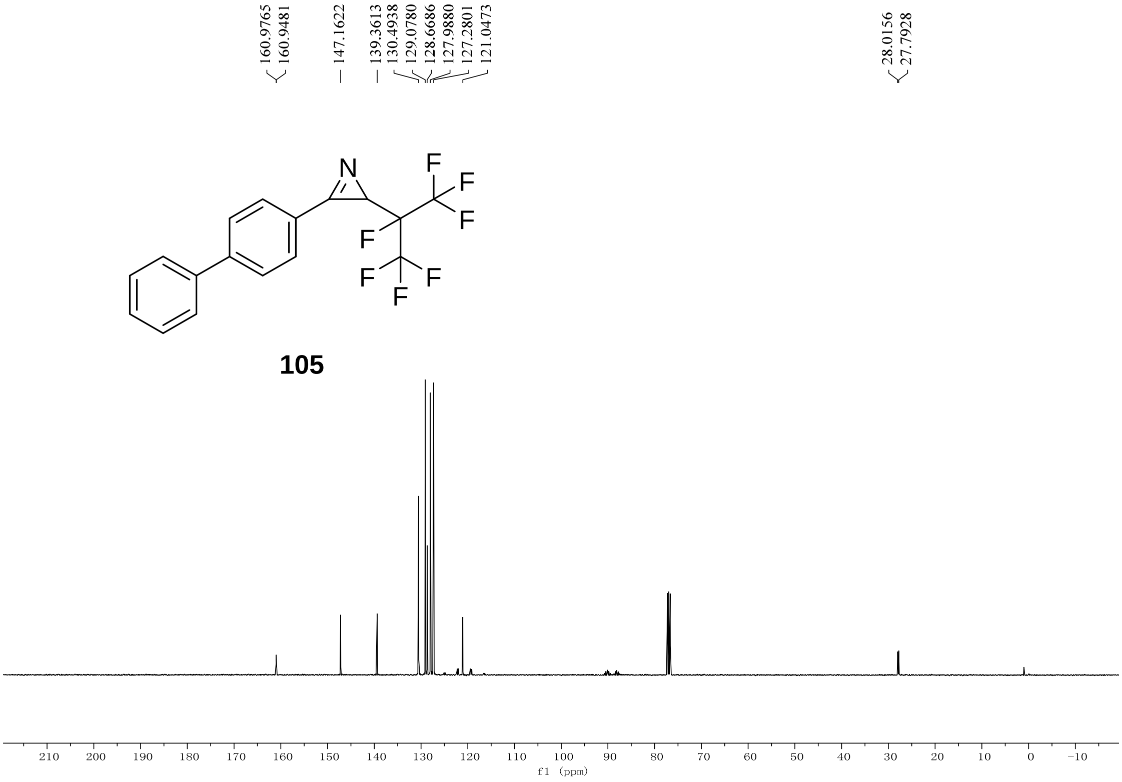

Supplementary Figure 277. ^{13}C NMR spectrum for compound **105**

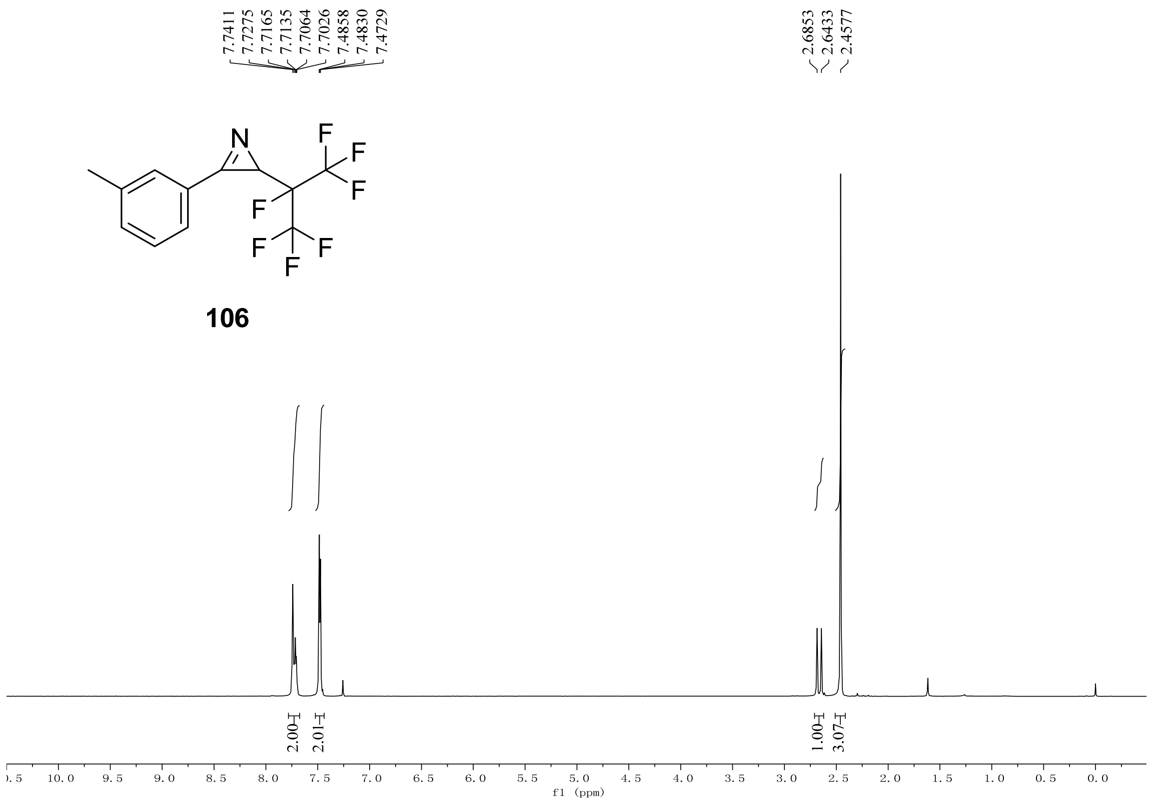

Supplementary Figure 278. ^{1}H NMR spectrum for compound **106**

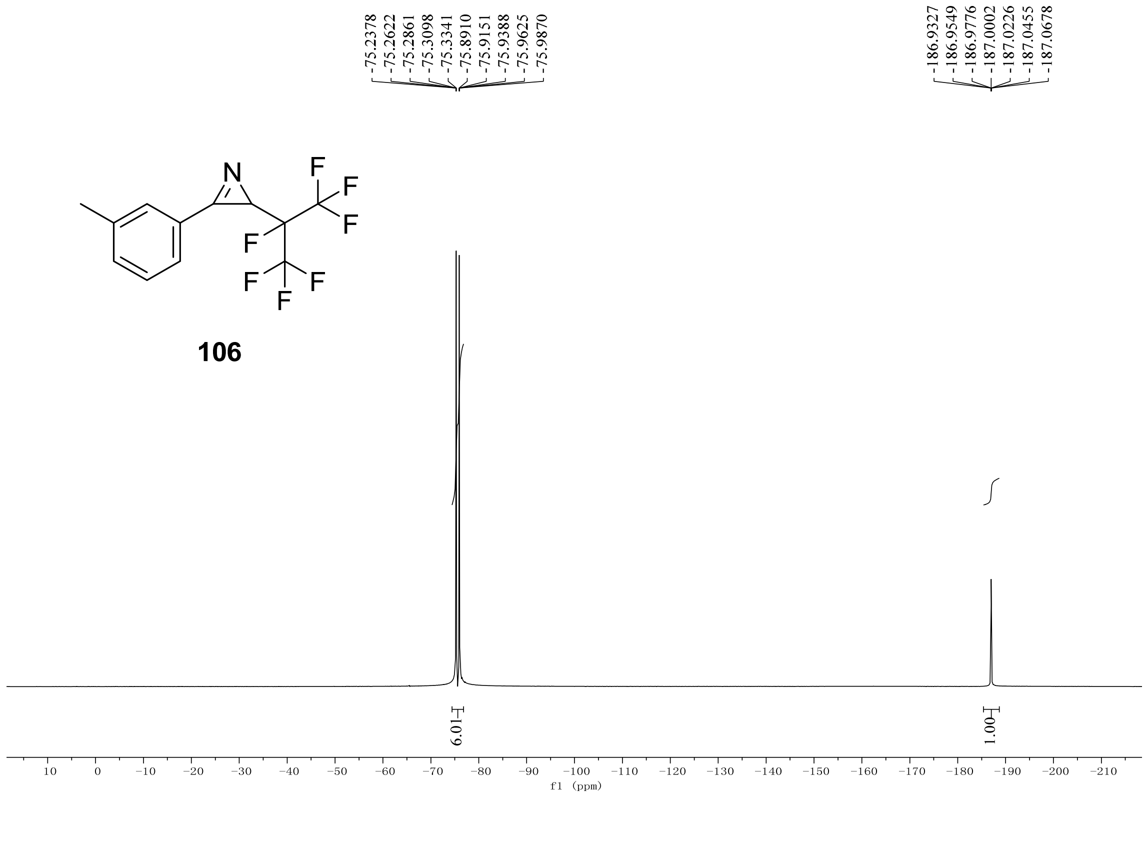

Supplementary Figure 279. ^{19}F NMR spectrum for compound **106**

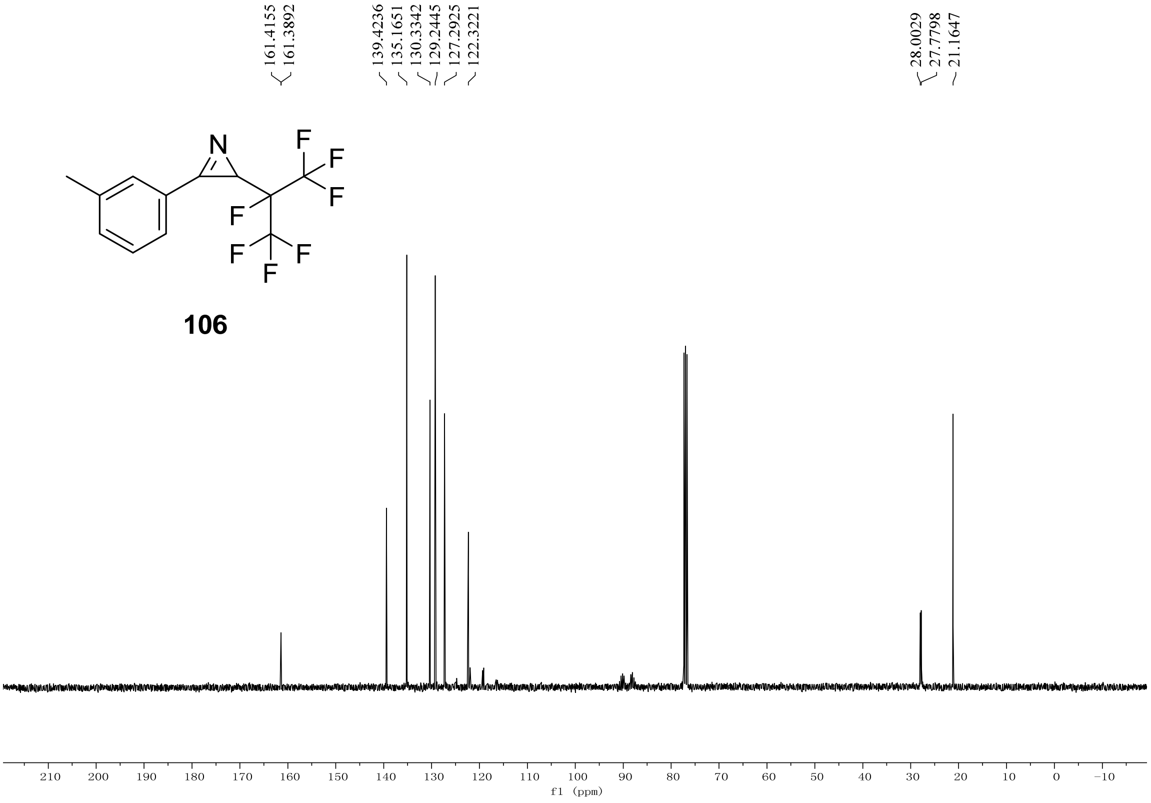

Supplementary Figure 280. ^{13}C NMR spectrum for compound **106**

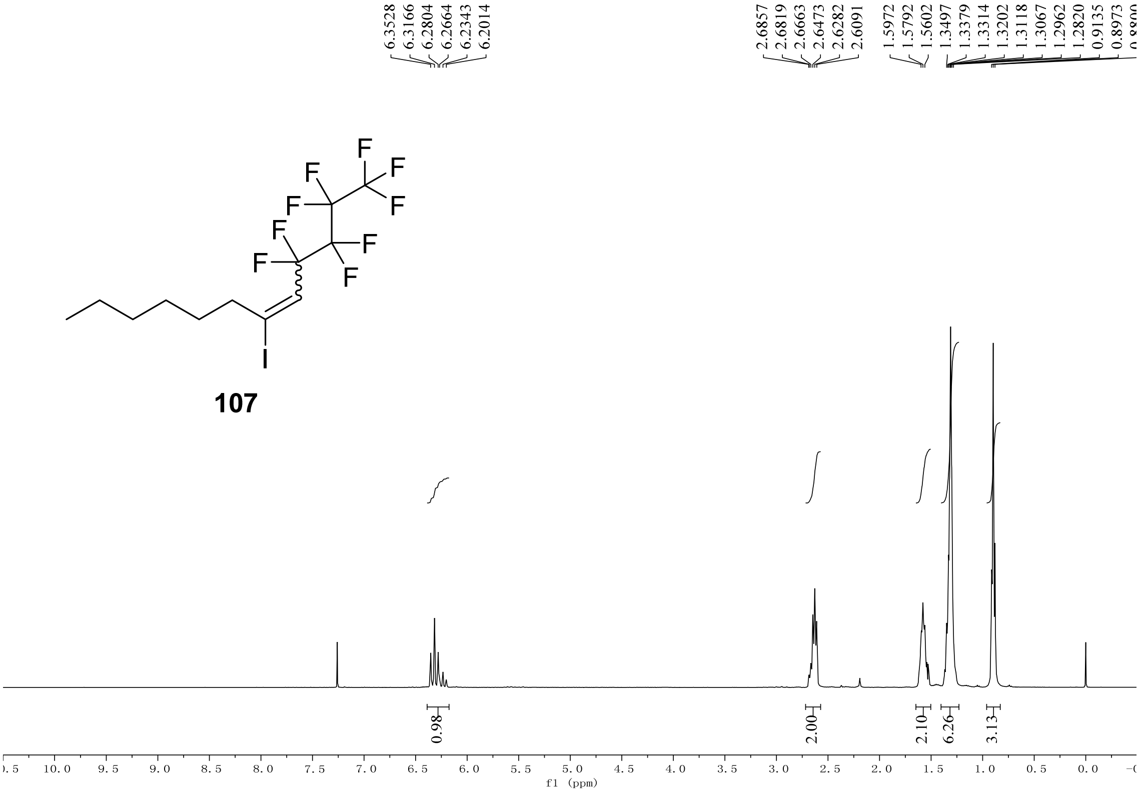

Supplementary Figure 281. ^{1}H NMR spectrum for compound **107**

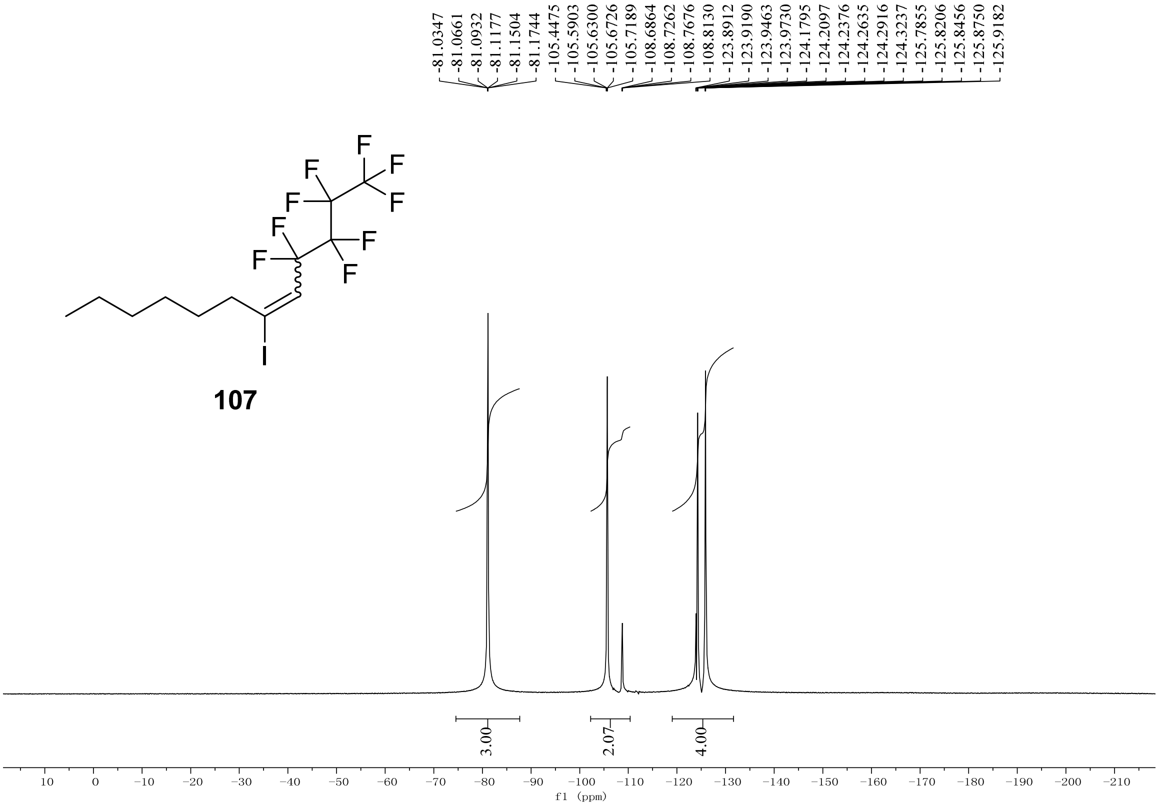

Supplementary Figure 282. ^{19}F NMR spectrum for compound **107**

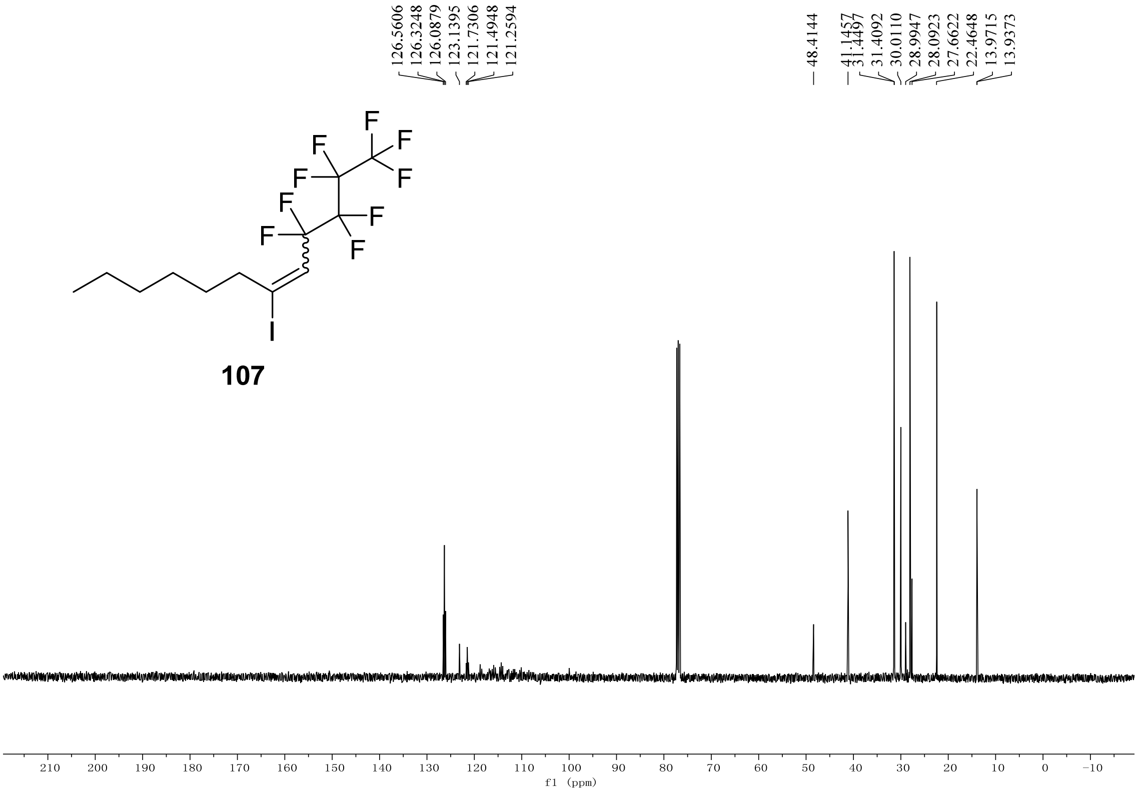

Supplementary Figure 283. ^{13}C NMR spectrum for compound **107**

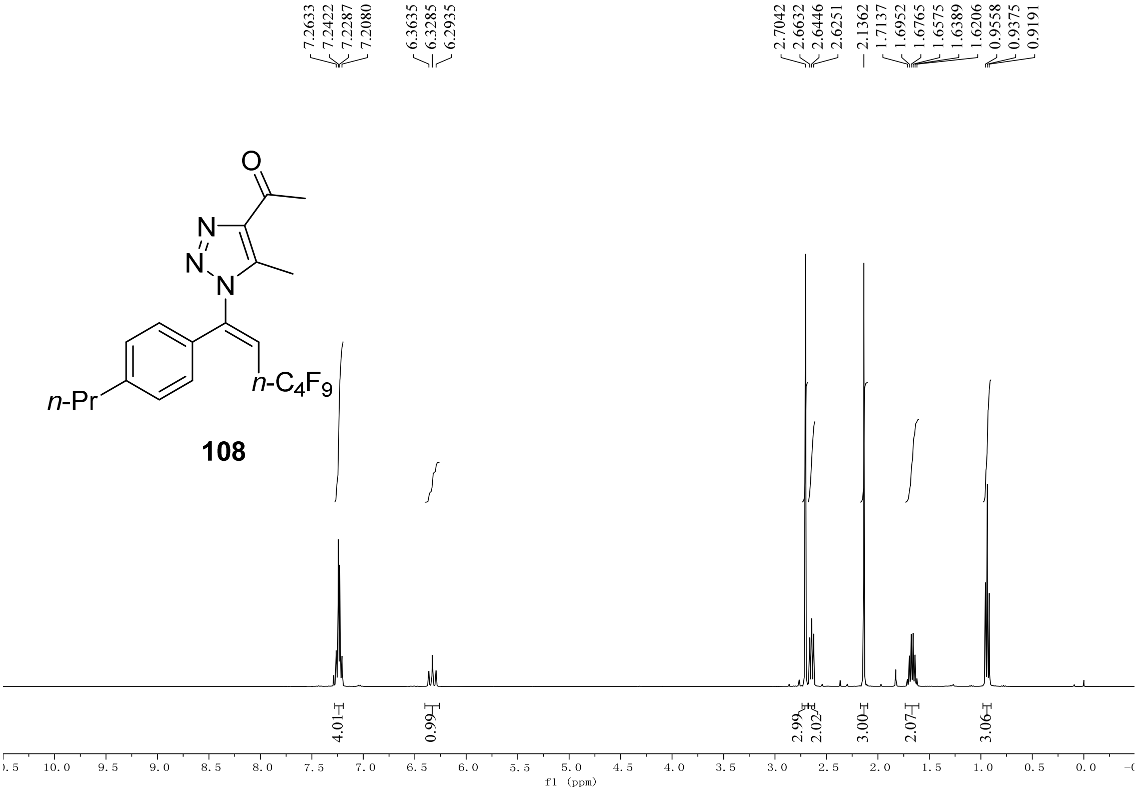

Supplementary Figure 284. ^{1}H NMR spectrum for compound **108**

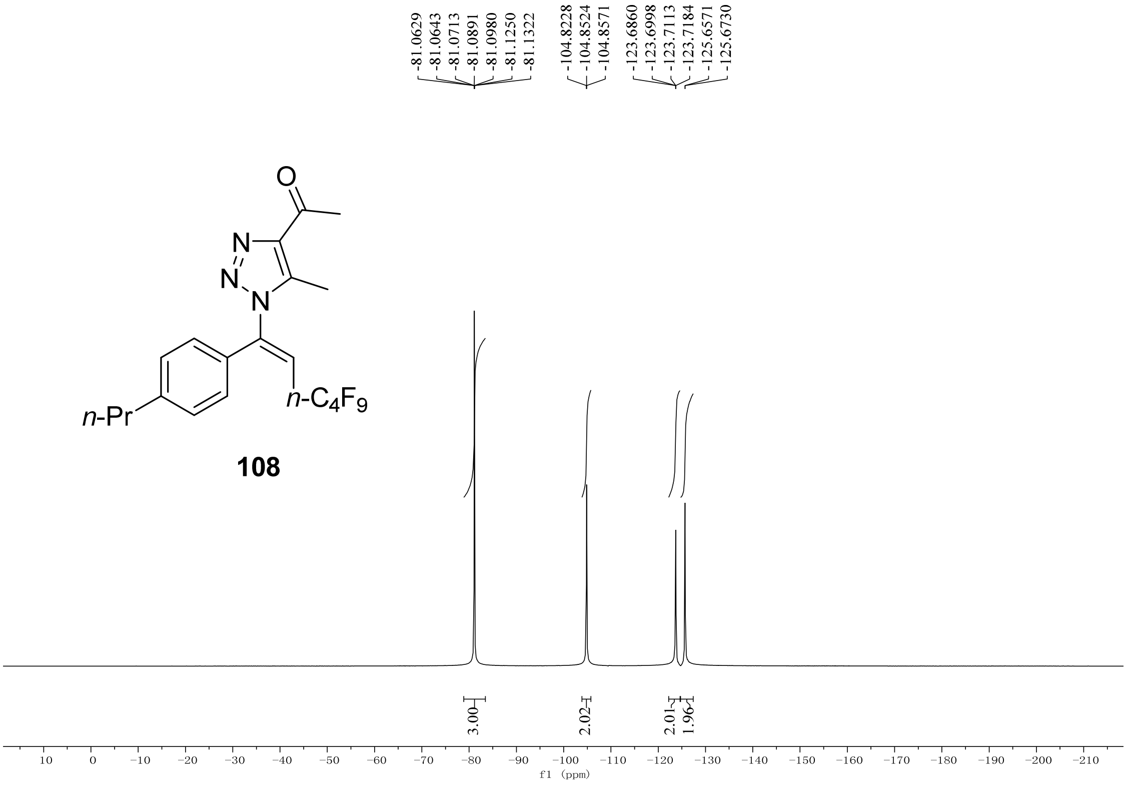

Supplementary Figure 285. ^{19}F NMR spectrum for compound **108**

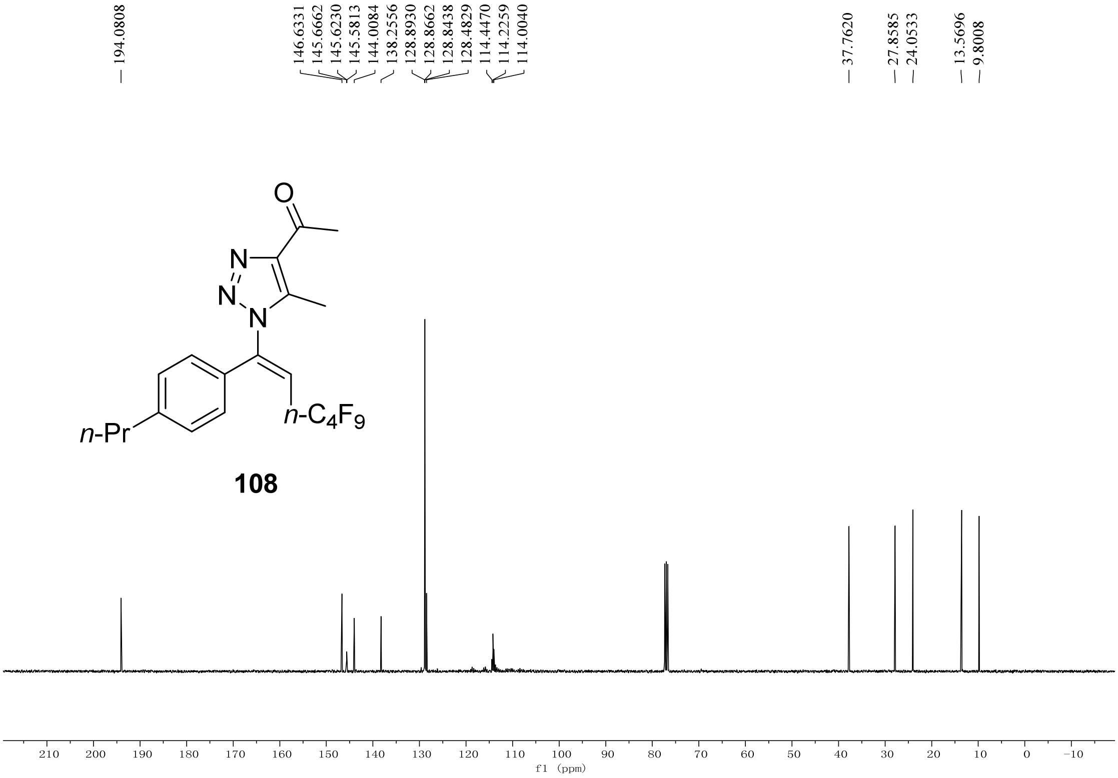

Supplementary Figure 286. ^{13}C NMR spectrum for compound **108**

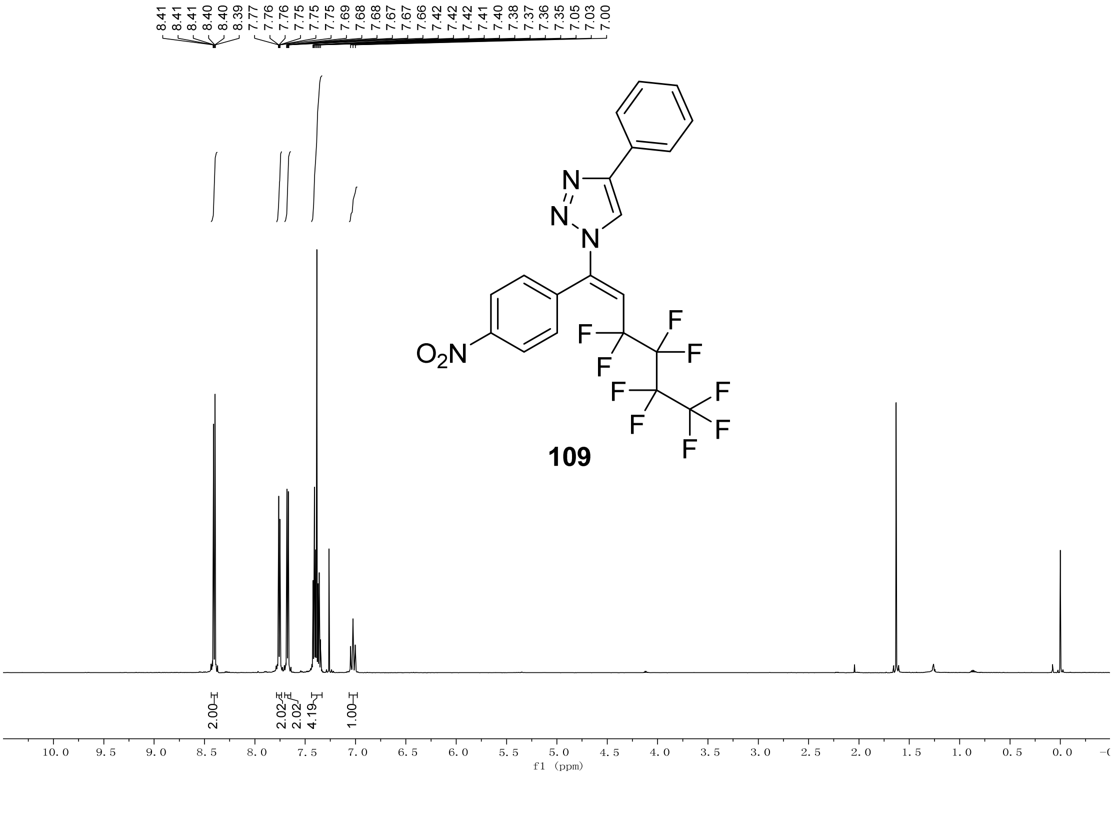

Supplementary Figure 287. ^{1}H NMR spectrum for compound **109**

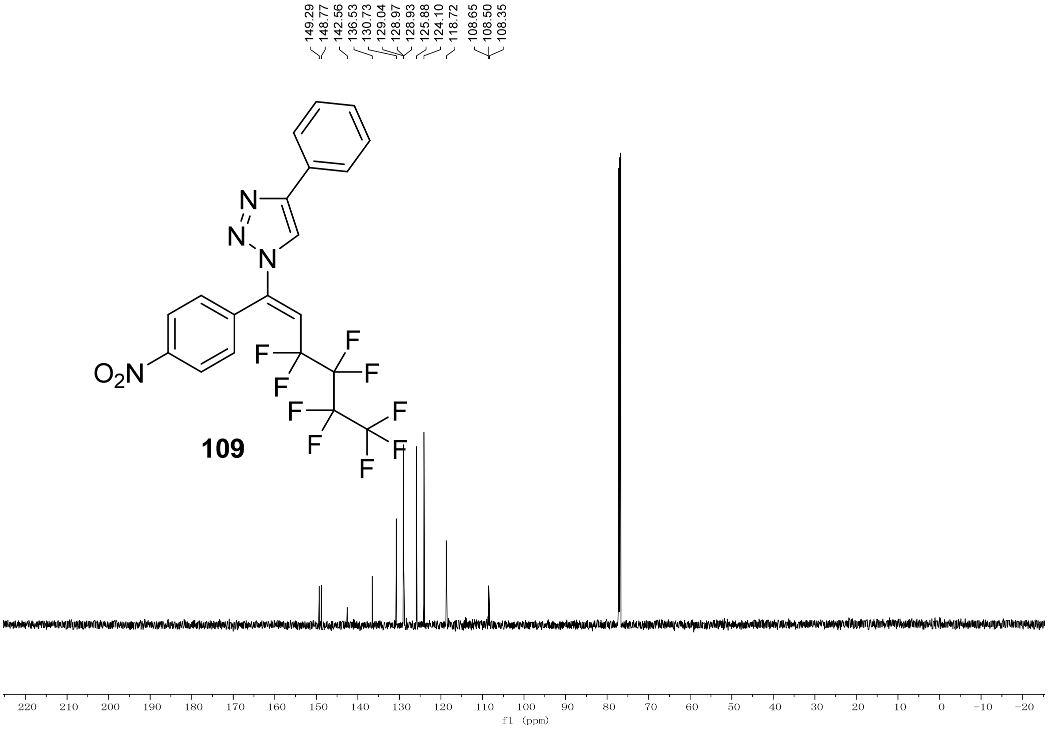

Supplementary Figure 288. ^{13}C NMR spectrum for compound **109**

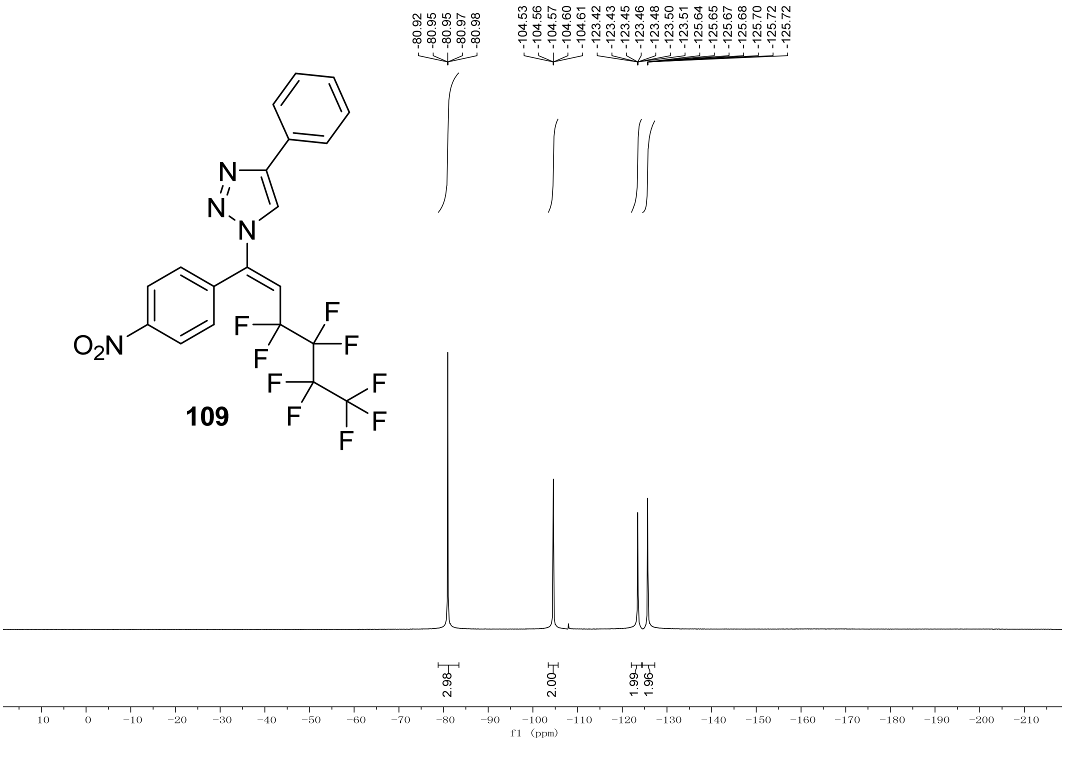

Supplementary Figure 289. ^{19}F NMR spectrum for compound **109**

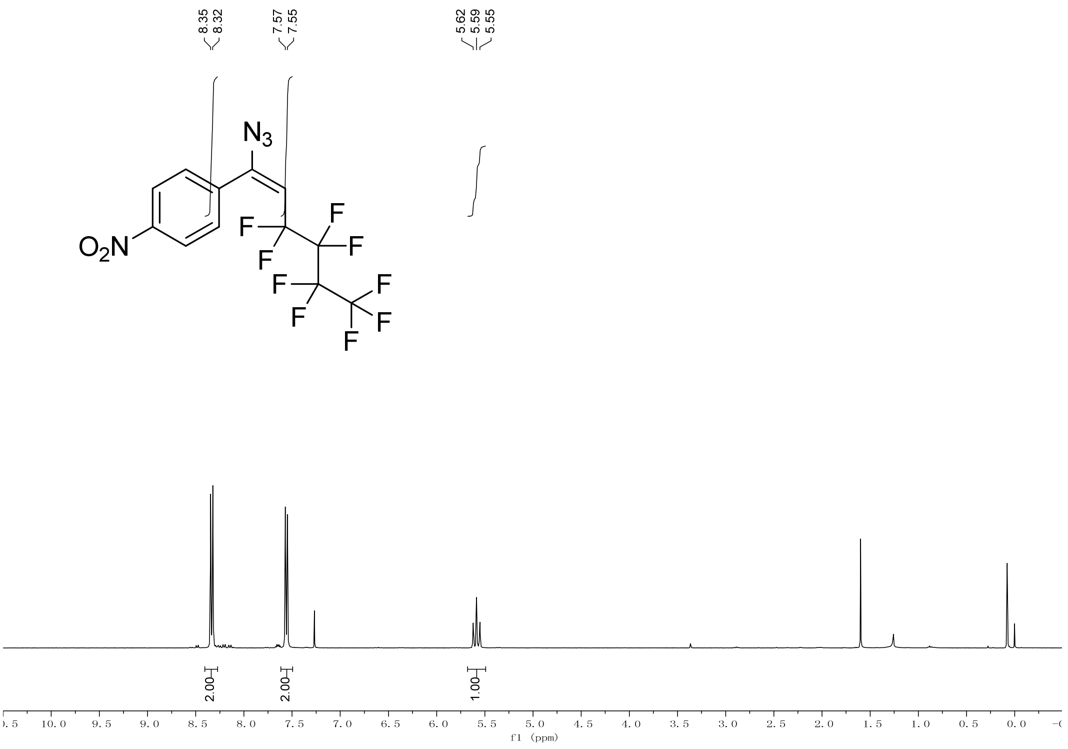

Supplementary Figure 290. ^{1}H NMR spectrum for compound (*E*)-1-(1-azido-3,3,4,4,5,5,6,6,6-nonafluorohex-1-en-1-yl)-4-nitrobenzene

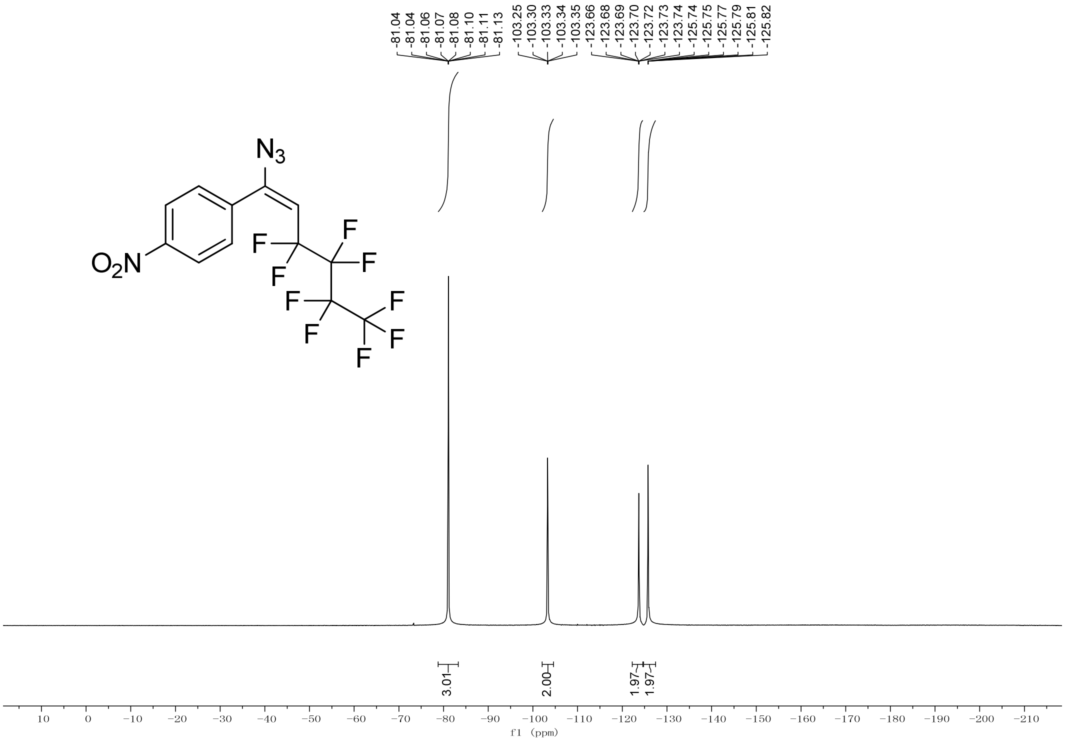

Supplementary Figure 291. ^{19}F NMR spectrum for compound (*E*)-1-(1-azido-3,3,4,4,5,5,6,6,6-nonafluorohex-1-en-1-yl)-4-nitrobenzene

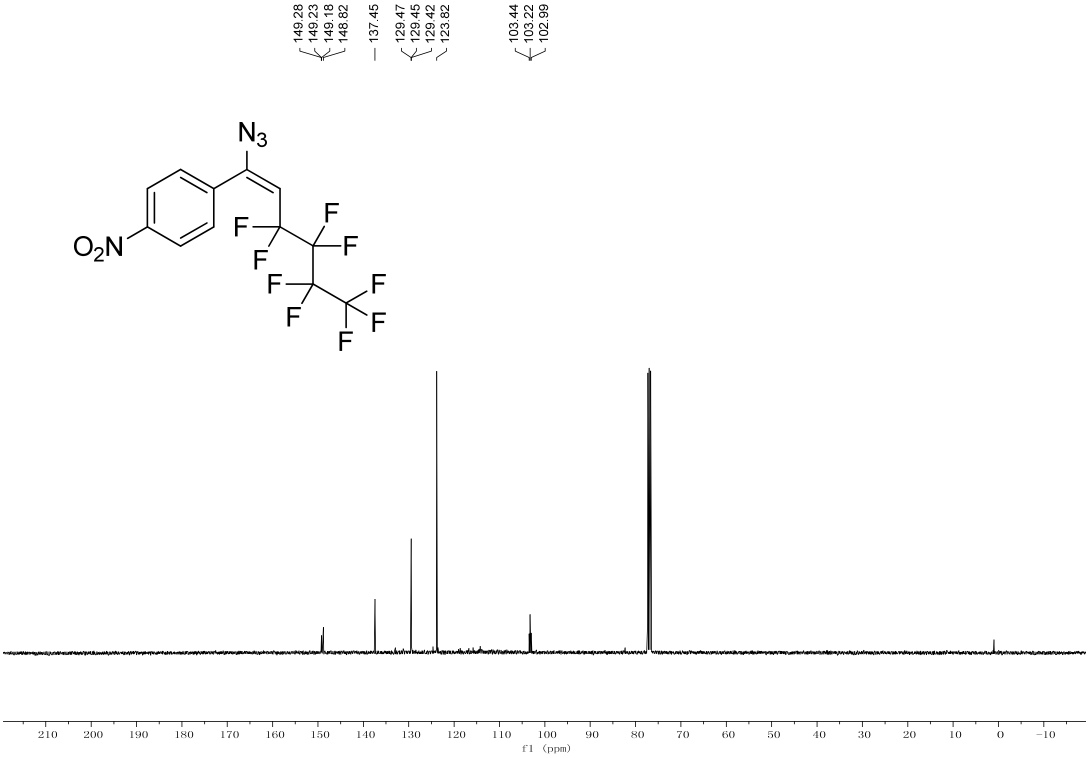

Supplementary Figure 292. ^{13}C NMR spectrum for compound (E)-1-(1-azido-3,3,4,4,5,5,6,6,6-nonafluorohex-1-en-1-yl)-4-nitrobenzene

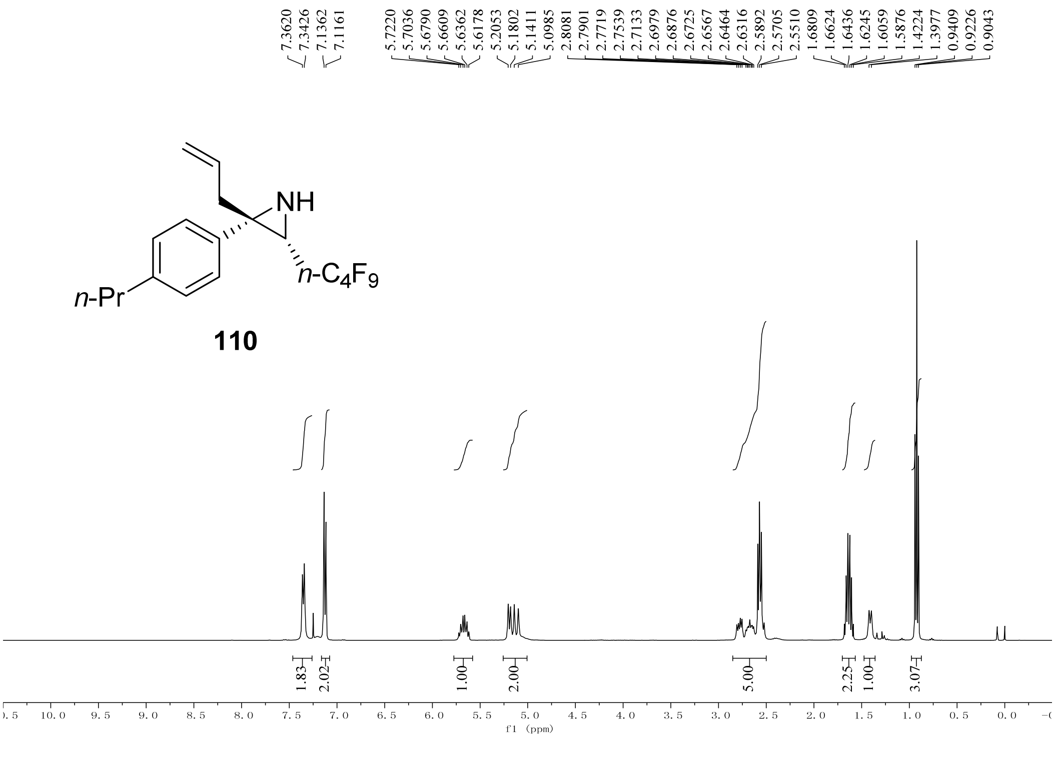

Supplementary Figure 293. ^{1}H NMR spectrum for compound **110**

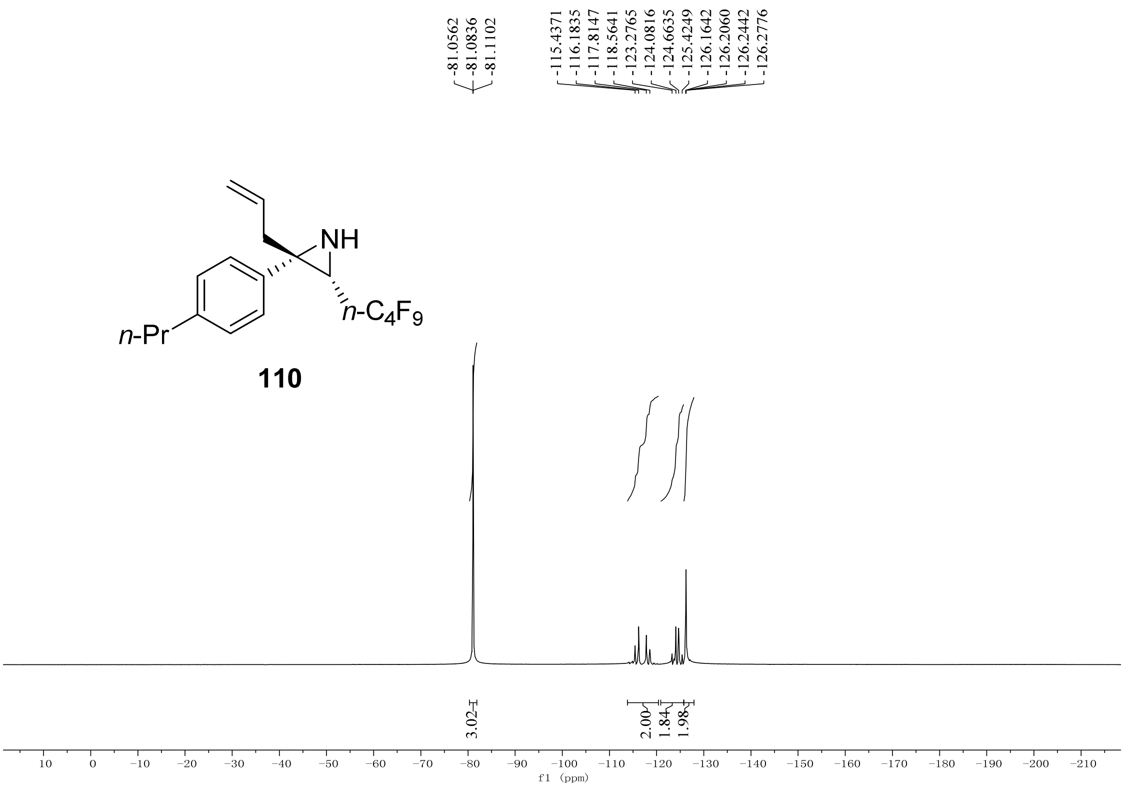

Supplementary Figure 294. ^{19}F NMR spectrum for compound **110**

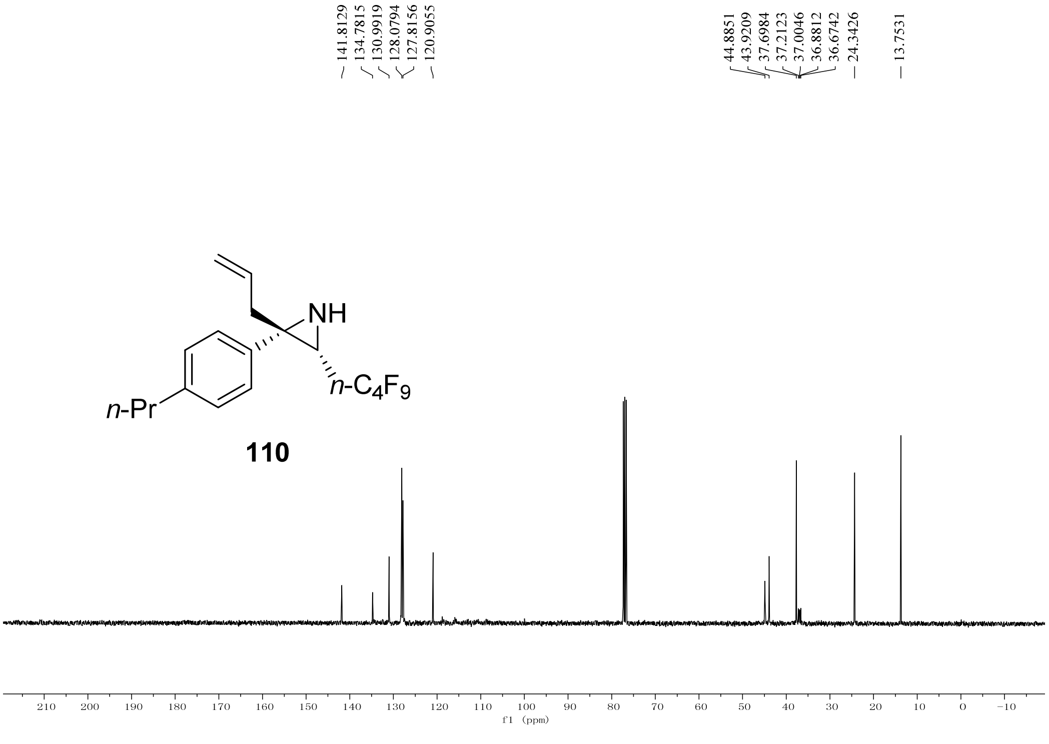

Supplementary Figure 295. ^{13}C NMR spectrum for compound **110**

Supplementary References

1. Wang, F., Qi, X., Liang, Z., Chen, P., Liu, G. Copper-Catalyzed Intermolecular Trifluoromethylazidation of Alkenes: Convenient Access to CF3-Containing Alkyl Azides. *Angew. Chem. Int. Ed.* **53**, 1881-1886 (2014).

2. Geng, X., Lin, F., Wang, X., Jiao, N. Azidofluoroalkylation of Alkenes with Simple Fluoroalkyl Iodides Enabled by Photoredox Catalysis. *Org. Lett.* **19**, 4738-4741 (2017).

3. Green, J. E., Bender, D. M., Jackson, S., O'donnell, M. J., Mccarthy, J. R. Mitsunobu Approach to the Synthesis of Optically Active alpha,alpha-Disubstituted Amino Acids. *Org. Lett.* **11**, 807-810 (2009).

4. Camps, P., *et al.* Synthesis and absolute configuration of novel N,O-psiconucleosides using (R)-N-phenylpantolactam as a resolution agent. *J. Org. Chem.* **73**, 6657-6665 (2008).

5. Konno, T., *et al.* Facile syntheses of various per- or polyfluoroalkylated internal acetylene derivatives. *Tetrahedron* **59**, 7571-7580 (2003).

6. Ng, E. P. J., Wang, Y.-F., Hui, B. W.-Q., Lapointe, G., Chiba, S. Orthogonal synthesis of pyrroles and 1,2,3-triazoles from vinyl azides and 1,3-dicarbonyl compounds. *Tetrahedron* **67**, 7728-7737 (2011).

7. Liu, Z., Liu, J., Zhang, L., Liao, P., Song, J., Bi, X. Silver(I)-Catalyzed Hydroazidation of Ethynyl Carbinols: Synthesis of 2-Azidoallyl Alcohols. *Angew. Chem. Int. Ed.* **53**, 5305-5309 (2014).

8. Hirashita, T., Toumatsu, S., Imagawa, Y., Araki, S., Setsune, J. Stereoselective allylation of azirines with allylindium reagents. *Tetrahedron Lett.* **47**, 1613-1616 (2006).
